# Stereocontrolled Synthesis of Dimethylamino Phosphorochloridate Monomers toward Stereopure Phosphorodiamidate Morpholino Oligonucleotides

**DOI:** 10.1021/acs.joc.5c02914

**Published:** 2026-03-12

**Authors:** Ryuichi Inutake, Hironao Hasegawa, Taiki Tsurusaki, Taiichi Sakamoto, Kazuki Sato, Takeshi Wada

**Affiliations:** a Department of Medicinal and Life Sciences, Faculty of Pharmaceutical Sciences, Tokyo University of Science, Niijuku, Katsushika, Tokyo 125−8585, Japan; b Discovery Research Laboratories, Nippon Shinyaku Co., Ltd., 3−14−1, Sakura, Tsukuba, Ibaraki 305−0003, Japan; c Department of Life Science, Faculty of Advanced Engineering, Chiba Institute of Technology, Tsudanuma, Narashino, Chiba 275-0016, Japan

## Abstract

As antisense oligonucleotides, phosphorodiamidate morpholino oligonucleotides (PMOs) exhibit excellent properties. However, they possess two stereoisomers for each phosphorus atom, and these stereoisomers exhibit different physicochemical and biological properties. In this study, we developed a stereocontrolled synthesis method of dimethylamino phosphorochloridate monomers, which was used for a practical synthesis method of PMOs, from oxazaphospholidine derivatives. However, the condensation of a 5′-oxazaphospholidine derivative with an amine under acidic conditions is challenging because the resulting phosphoramidite intermediate can be activated under such conditions. To address this challenge, in the proposed synthesis method, a morpholino nucleoside 5′-oxazaphospholidine derivative was condensed with a phenol derivative with a low p*K*_*a*_ value under acidic conditions. Subsequently, the resulting aryl phosphite was reacted with dimethylamine to yield a phosphoramidite, thereby liberating the phenol derivative as a leaving group. Chlorination of the phosphoramidite yielded a phosphorochloridate monomer in a highly stereoselective manner (dr = 93:7–97:3). Subsequently, the resulting chloridate monomer was stereospecifically condensed with the amino group of the morpholino nucleoside. The stereochemistry of the phosphorodiamidate morpholino dimers was unambiguously determined by nuclear magnetic resonance analysis. The results of this study facilitate the synthesis of stereocontrolled PMOs and the elucidation of their properties.

## Graphical abstract

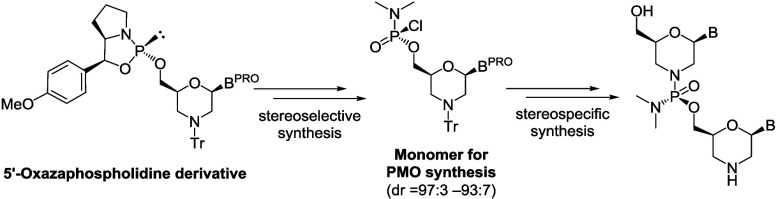

## Introduction

Antisense oligonucleotides (ASOs) are a novel type of therapeutics that can control protein expression via the formation of a duplex with a target mRNA.^1^ Phosphorodiamidate morpholino oligonucleotides (PMOs) are promising ASOs because of their high duplex-forming ability,^2^ stability in vivo,^2,3^ and low cytotoxicity.^4^ To date, four PMO drugs (Exondys 51,^5^ Vyondys 53,^6^ Viltepso,^7^ and Amondys 45 ^8^) for Duchenne muscular dystrophy have been approved by the U.S. Food and Drug Administration, with additional PMO drugs expected to be approved in the future. However, PMOs exhibit poor pharmacokinetic properties because of their low membrane permeability and blood retentivity.^4^ A solution to this problem is the stereocontrolled synthesis of phosphorus centers. PMOs possess two stereoisomers for each phosphorus atom, and currently available PMO drugs comprise mixtures of stereoisomers ranging from millions to hundreds of millions. In the case of phosphorothioate-modified oligonucleotides, properties such as lipophilicity, RNaseH activity, and cytotoxicity vary among stereoisomers.^9^ The properties such as lipophilicity are thought to be related to membrane permeability and blood retentivity; thus, the stereocontrol of PMOs is expected to address their disadvantages. However, insights into the properties of PMO stereoisomers have not been investigated, probably due to the lack of methods for the stereocontrolled synthesis of PMOs.

Thus far, several synthesis approaches for PMO diastereomixtures have been developed. The first example of PMO synthesis was reported by Summerton and Weller in 1993.^10^ In their report, PMOs were synthesized via the condensation of a morpholino nucleoside 5′-dimethylamino phosphorochloridate derivative with an amino group of another morpholino nucleoside. However, the reactivity of the monomers is marginal, and the condensation reaction requires a considerable amount of time. The condensation reaction rate is improved by adding activators, such as LiBr^11^ and ethylthiotetrazole,^12^ and increasing the reaction temperature.^13^ Recently, to address the low reactivity issue of P­(V) derivatives, PMO synthesis using reactive P­(III) compounds has been intensively investigated. For example, the research group led by Sinha used a 5′-*H*-phosphonate derivative as a monomer unit for the solid-phase synthesis of PMOs.^14^ Recently, we reported the solution-phase synthesis of PMOs using 5′-*H*-phosphonate derivatives.^15^ As another approach, Ghosh et al. reported the synthesis of PMOs, thiophosphoramidate morpholino oligonucleotides (TMOs), which are other promising antisense candidates, and PMO–TMO chimera using 5′-phosphoramidite derivatives.^16^ In their method, a 5′-phosphoramidite monomer was condensed with an amino group of a morpholino nucleoside in the presence of an acidic activator. After coupling, the resulting internucleotidic linkage is converted to a phosphorodiamidate or thiophosphoramidate counterpart via oxidative amination with I_2_ and Me_2_NH or sulfurization with 3-((dimethylamino-methylidene)­amino)-3*H*-1,2,4-dithiazole-3-thione (DDTT), respectively. Subsequent detritylation, followed by repetitive synthetic cycles, yielded PMOs and TMOs. Although this is a sophisticated method, the reaction intermediate is also a phosphoramidite derivative, which is activated under acidic conditions. Thus, the condensation reaction is inevitably reversible, resulting in low efficiency. They addressed this problem by performing condensation and *P*-modification repeatedly.

The stereocontrolled synthesis of PMOs was reported in a patent application by Endo et al.^17^ In their report, the stereoisomers of a dimethylamino phosphorochloridate monomer were separated via high-performance liquid chromatography using a chiral column. Subsequently, the stereopure monomer was condensed with an amino group of a morpholino nucleotide to afford stereocontrolled PMOs (Scheme 1A). The condensation reaction proceeds in a stereoselective manner. Thus, the development of a stereoselective synthesis method for dimethylamino phosphorochloridate monomers results in easy access to stereodefined PMOs. Using this strategy, the synthesis and the properties of gapmer ASOs with stereocontrolled PMOs in the wing region were reported by Kanatsu et al.^18^ They investigated the thermal stability of duplexes formed between stereodefined PMO 10 mers, namely, homoadenylate, cytidylate, thymidylate, and guanylate, and their complementary RNAs. The *T*_m_ values of the PMO 10 mers *S*p homoadenylate, cytidylate, thymidylate, and guanylate duplexes with their complementary RNAs were 68.5, 77.3, 31.3, and 88.8 °C, whereas the *T*_m_ values of the *R*p PMO 10 mers duplexes with their complementary RNAs were 37.0, 63.5, 19.3, and 73.1 °C, respectively. These results suggest that all *S*p-PMOs exhibit greater duplex-forming ability than the corresponding *R*p-PMOs, indicating that the stereocontrol of PMOs is effective for enhancing PMO properties. In addition, the stereocontrolled synthesis of dimethylamino phosphorochloridate monomers using an oxathiaphospholane derivative was reported in a patent application by Choi et al.^19^

**Scheme 1:**
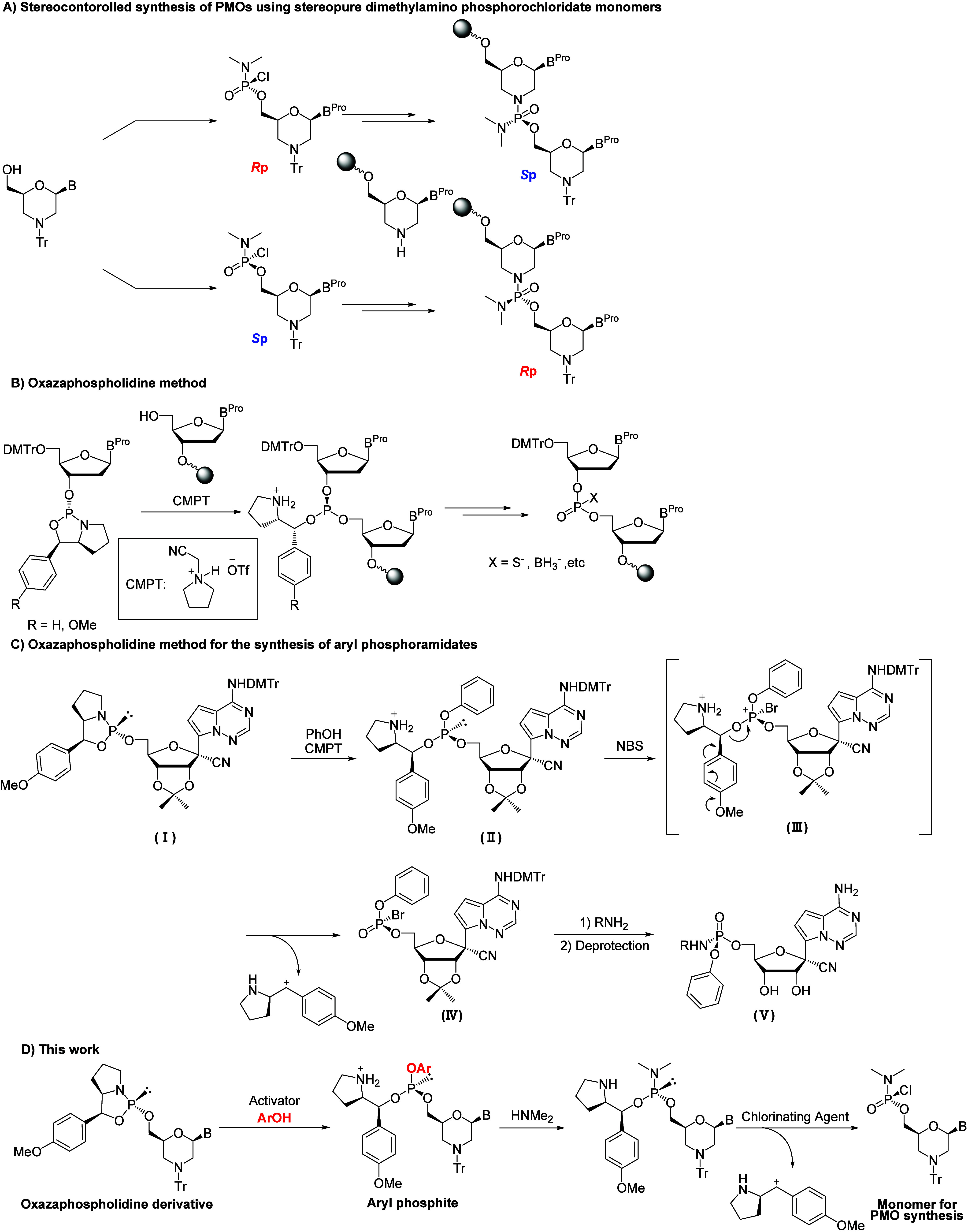
Strategies Adopted in This Study

In this study, we attempted the stereocontrolled synthesis of dimethylamino phosphorochloridate monomers using the oxazaphospholidine method, which has been used for the stereocontrolled synthesis of *P*-modified DNA and RNA derivatives, such as a phosphorothioate and boranophosphate (Scheme 1B).^20–22^ Oxazaphospholidine is a phosphoramidite derivative with a chiral auxiliary. Oxazaphospholidine derivatives can condense with a hydroxy group in the presence of *N*-(cyanomethyl)­pyrrolidinium triflate (CMPT)^22^ as a non-nucleophilic acid activator to stereospecifically afford phosphite intermediates. Subsequently, the modification of the intermediates on phosphorus atoms affords *P*-modified DNAs and RNAs. Therefore, the oxazaphospholidine method is expected to be effective for the stereocontrolled synthesis of dimethylamino phosphorochloridate monomers. However, to apply this method to the synthesis of phosphorochloridate monomers, an amine must be condensed with an oxazaphospholidine derivative under acidic conditions to afford a phosphoramidite intermediate. The resulting phosphoramidite intermediate can be activated under acidic conditions; thus, the reaction will be reversible, resulting in low efficiency. Thus, the stereocontrolled synthesis of dimethylamino phosphorochloridate monomers using the oxazaphospholidine method is not straightforward.

Meanwhile, Kers et al. synthesized *H*-phosphonamidate derivatives from 2,4,6-trichlorophenyl trimethylsilyl phosphite intermediates.^23,24^ They attempted the synthesis of *H*-phosphonamidate derivatives using an *H*-phosphonate monoester, an amine, and pivaloyl chloride (PivCl) as a condensing reagent. However, the amine preferentially reacted with the carbonyl groups of PivCl and the activated *H*-phosphonate monomer, resulting in the acylation of the amino group. They solved this problem using an aryl phosphite intermediate. The condensation of the *H*-phosphonate monoester and 2,4,6-trichlorophenol yielded an aryl phosphite. Subsequently, the reaction with the amine in the presence of a silylating reagent afforded a phosphoramidite. This result demonstrates that a highly acidic phenol derivative of an aryl phosphite can serve as a good leaving group for nucleophilic substitution with an amine. In addition, we previously reported the stereocontrolled synthesis of nucleotide analog prodrugs (ProTides) such as Remdesivir using oxazaphospholidine derivatives (I) bearing a methoxyphenyl group on the 5-position of the oxazaphospholidine ring (Scheme 1C).^25^ In the previous report, monoaryl phosphite triester (II) was synthesized via the condensation of an oxazaphospholidine derivative (I) with phenol. Subsequently, oxidative bromination and amination were successively performed to stereoselectively afford aryl phosphoramidate derivatives. The condensation reaction of oxazaphospholidine derivatives with phenol proceeded efficiently. The chiral auxiliary bearing a methoxyphenyl group was removed by treatment with an oxidative bromination reagent via a phosphonium intermediate (III). Subsequently, the amination on the resulting phosphorobromidate (IV) proceeded with the inversion of phosphorus stereochemistry, and the deprotection of the nucleobase and sugar backbone afforded ProTides (V). Chlorination rather than bromination is also expected to proceed with the removal of the chiral auxiliary, liberating stabilized benzyl cation species.

In the present study, the stereocontrolled synthesis of the chloridate monomer using the oxazaphospholidine method via an aryl phosphite as the key intermediate was investigated (Scheme 1D). Furthermore, the resulting chloridate monomers were condensed with the amino group of a morpholino nucleoside. The dimers were stereospecifically obtained, and the stereochemistry of the phosphorus atoms was successfully determined by nuclear magnetic resonance (NMR) analysis using NOESY or ROESY experiments.

## Results and Discussion

### Synthesis of Morpholino Nucleoside 5′-Oxazaphospholidine Derivatives

First, we synthesized morpholino nucleoside 5′-oxazaphospholidine derivatives via the coupling of a phosphitylating reagent (4*R*,5*S*) or (4*S*,5*R*)-**2** with one of four 5′-hydroxy-*N*-trityl (Tr)-morpholino nucleoside: thymidine **3** (Th), *N* ^4^-benzoylcytidine **4** (Cy^bz^), *N* ^6^-benzoyladenosine **5** (Ad^bz^), or *N* ^2^-isobutyryl-*O* ^6^-cyanoethylguanosine **6** (Gu^ce, ibu^) (Table 1). (α*S*,2*R*) or (α*R*,2*S*)-**1** was reacted with phosphorus trichloride to yield a phosphitylation reagent (4*R*,5*S*)- or (4*S*,5*R*)-2-chloro-1,3,2-oxazaphospholidine ((4*R*,5*S*) or (4*S*,5*R*)-**2**).^21^ Subsequently, the phosphitylation reagent was reacted with the 5′-hydroxy group of a morpholino nucleoside (either **3**, **4**, **5**, or **6**) to obtain an oxazaphospholidine (either **7**, **8**, **9**, or **10**). As shown in Table 1, all oxazaphospholidine derivatives were synthesized with high stereopurity and moderate isolated yields. Thin-layer chromatography (TLC) analysis of the reaction mixture indicated almost complete consumption of the 5′-hydroxy morpholino nucleoside in all cases. However, the ^31^P NMR spectra of the 5′-oxazaphospholidine derivatives after silica gel column chromatography purification indicated the presence of an oxidized counterpart (δ_P_ = 26.3). The separation of the byproduct was challenging, resulting in the reduced isolated yields of compounds **7**–**10**.

**Table 1:**
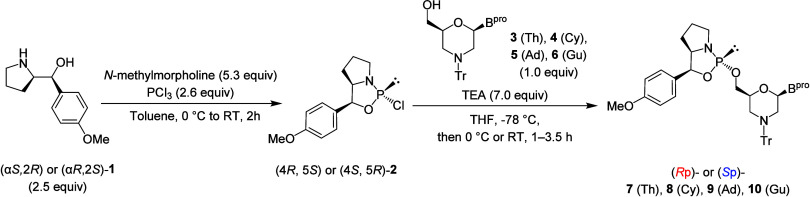
Synthesis of Oxazaphospholidine Derivatives

entry	2	nucleobase	product	isolated yield (%)	*R*p/*S*p^a^
1	(4*R*,5*S*)	Th	(*R*p)-**7**	62	>99:1
2	(4*R*,5*S*)	Cy^bz^	(*R*p)-**8**	76	>99:1
3	(4*R*,5*S*)	Ad^bz^	(*R*p)-**9**	31	>99:1
4	(4*R*,5*S*)	Gu^ce, ibu^	(*R*p)-**10**	33	>99:1
5	(4*S*,5*R*)	Th	(*S*p)-**7**	66	>1:99
6	(4*S*,5*R*)	Cy^bz^	(*S*p)-**8**	71	>1:99
7	(4*S*,5*R*)	Ad^bz^	(*S*p)-**9**	26	>1:99
8	(4*S*,5*R*)	Gu^ce, ibu^	(*S*p)-**10**	42	>1:99

a Determined by ^31^P NMR.

### Investigations of Dimethylamination Conditions

The stereocontrolled synthesis of phosphorochloridate monomers was investigated using the oxazaphospholidine derivatives. First, 4-nitrophenol was selected as the leaving group of an aryl phosphite and condensed with the (*R*p)-5′-oxazaphospholidine thymidine derivative ((*R*p)-**7**, 1.05 equiv) in the presence of CMPT as an acidic activator in MeCN at 0 °C (Scheme 2). Then, the partial solution was transferred to an NMR tube and analyzed by ^31^P NMR. As shown in the ^31^P NMR spectrum of the reaction mixture, major signals are observed at 133.0 and 12.2 ppm, which can be attributed to an arylphosphite intermediate **11** and an *H*-phosphonate diester, which was the hydrolyzed product of **11**, respectively (Figure 1a). We assumed that the hydrolysis of the intermediate **11** proceeded at the time of the transference of the reaction mixture. Subsequently, dimethylamine was added to the residue solution to replace nitrophenol as a leaving group. However, the ^31^P NMR spectrum of the reaction mixture indicated that the oxazaphospholidine derivative (*R*p)-**7** was regenerated (Figure 1b, δ_P_ = 154.6). This regeneration can be attributed to a reverse reaction in which the pyrrolidine moiety of the chiral auxiliary attacks the phosphorus atom under basic conditions.

**Scheme 2:**
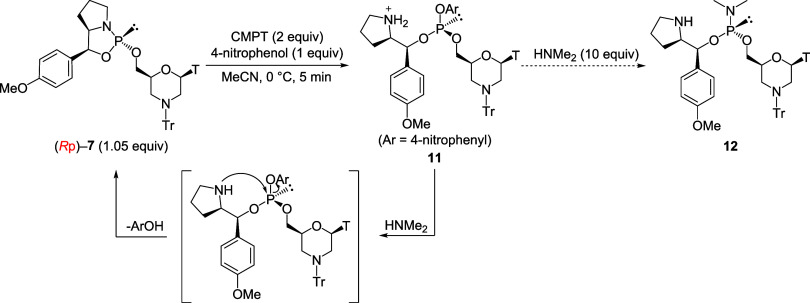
Synthesis of Arylphosphite and Plausible Mechanism of Recyclization

**Figure 1:**
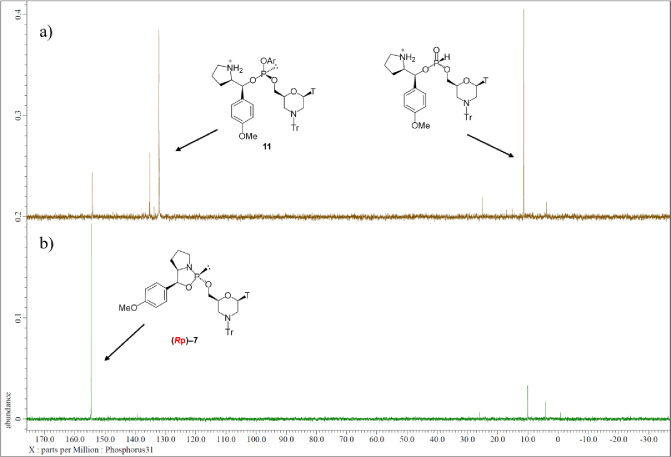
^31^P NMR spectra of reaction mixtures (a) after condensation with 4-nitrophenol; (b) after dimethylamination.

The capping of the pyrrolidine moiety was necessary to prevent this reverse reaction. Feathers et al. reported that phenyl isocyanate (PhNCO) is an effective scavenger of diisopropylamine in the phosphoramidite method.^26^ Therefore, we selected PhNCO as the acylating reagent of the pyrrolidine moiety in the condensation reaction. After the condensation reaction of (*R*p)-**7** and 4-nitrophenol in the presence of CMPT and PhNCO at 0 °C, the reaction with dimethylamine at room temperature (RT) and the oxidative chlorination of the resulting phosphoramidite by treatment with *N*-chlorosuccinimide (NCS) at 0 °C were performed (Scheme 3). A plausible mechanism for the removal of the chiral auxiliary is discussed in Supporting Information (Figures S28 and S29 and Scheme S9). According to the ^31^P NMR spectrum after the workup, the chloridate monomer **13** was successfully obtained; however, the diastereomer ratio of **13** (*S*p:*R*p) was low, approximately 31:69 (Table 2, entry 1). Therefore, each reaction was monitored via ^31^P NMR to elucidate which step caused a decrease in stereoselectivity. In the ^31^P NMR spectrum of the reaction mixture after the condensation of (*R*p)-**7** with 4-nitrophenol and the acylation by PhNCO, a primary signal that corresponded to the aryl phosphite **14** (δ_P_ = 136.2) was observed, along with a minor signal at 134.9 ppm in the phosphite region and a signal at 9.3 ppm (^1^ *J*_PH_ = 716 Hz), corresponding to an *H*-phosphonate diester, which was the hydrolyzed product of **14**. Although the signal at 134.9 ppm was not assigned, even if it originated from the diastereomer of **14**, it would imply high stereoselectivity (dr >97:3) (Figure 2a). However, the ^31^P NMR spectrum of the reaction mixture after the oxidative chlorination with NCS showed two signals corresponding to the two diastereomers of the chloridate monomer **13** (Figure 2b, *S*p:*R*p = 31:69). The stereochemistry of **13** was assigned based on the NMR analysis results, as described later. A signal corresponding to the *R*p-isomer was observed upfield compared with the *S*p counterpart.

**Scheme 3:**
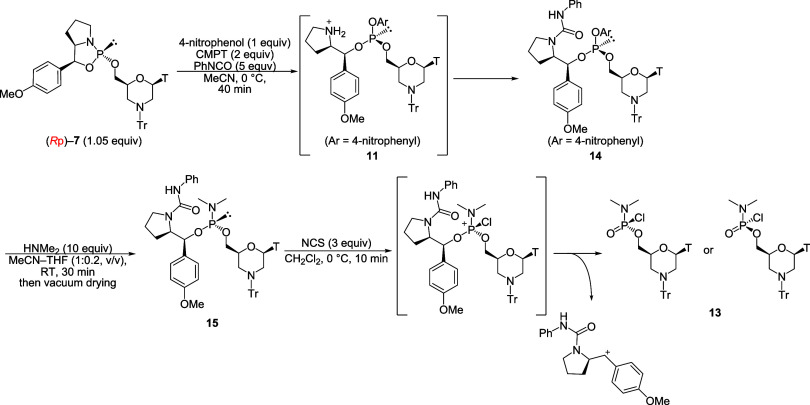
Synthesis of Dimethylamino Phosphorochloridate Monomer

**Figure 2:**
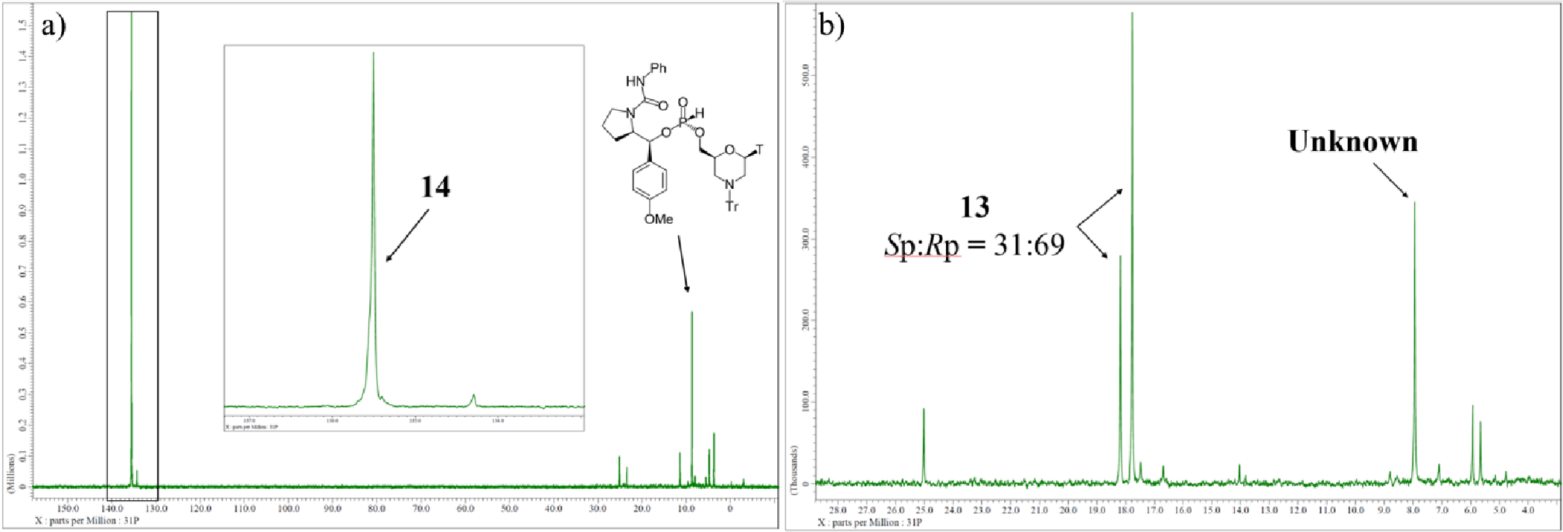
^31^P NMR spectra of reaction mixtures (a) after condensation with 4-nitrophenol and PhNCO; (b) after dimethylamination and chlorination.

**Table 2:**
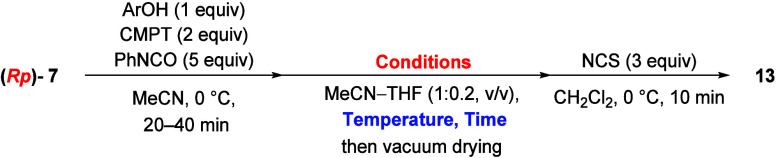
Investigation of Dimethylamination Conditions

entry	conditions	temperature (°C)	time	dr of **13** *S*p:*R*p^a^
1	HNMe_2_ (10 equiv)	RT	30 min	31:69
2	HNMe_2_ (10 equiv)	0	30 min	21:79
3	TMSNMe_2_ (5 equiv) HNMe_2_ (10 equiv)	0	1 h	6:94
4	TMSNMe_2_ (5 equiv) HNMe_2_ (10 equiv)	–20	1 h	3:97
5	TMSNMe_2_ (5 equiv) HNMe_2_ (10 equiv)	–40	1 h	3:97
6	BSA (2 equiv) HNMe_2_ (10 equiv)	–20	1 h	4:96

a Determined by the ^31^P NMR spectrum of the crude mixture.

According to these results, the stereoselectivity loss was attributed to the dimethylamination or oxidative chlorination step. Thus, we investigated the dimethylamination conditions (Table 2). In entry 2, the temperature of the dimethylamination reaction was lowered to 0 °C. This approach improved stereoselectivity; however, the results were not satisfactory (*S*p:*R*p = 21:79). We hypothesized that the epimerization was caused by the nucleophilic attack of the phenolate ion on the arylphosphite intermediate. Therefore, TMSNMe_2_ was added along with dimethylamine to silylate the liberated phenolate, further improving stereoselectivity (entry 3, *S*p:*R*p = 6:94). The investigation of the reaction temperature (entries 3–5) demonstrated that stereoselectivity plateaued at −20 °C (entry 4, *S*p:*R*p = 3:97).

Based on these investigations, stereoselectivity loss was suppressed by the conversion of phenolate ions into silyl ethers by the addition of TMSNMe_2_ (Scheme 4). Additionally, BSA was used for this reaction as a silylation reagent (entry 6). The result was comparable to that obtained with TMSNMe_2_, giving a similar diastereomer ratio (*S*p:*R*p = 4:96). Since TMSNMe_2_ gives HNMe_2_ upon silylation, which is a reagent of this step, and both TMSNMe_2_ and HNMe_2_ are volatile and easily removed under reduced pressure before the chlorination step, we selected the conditions shown in entry 4 as the optimal conditions.

**Scheme 4:**
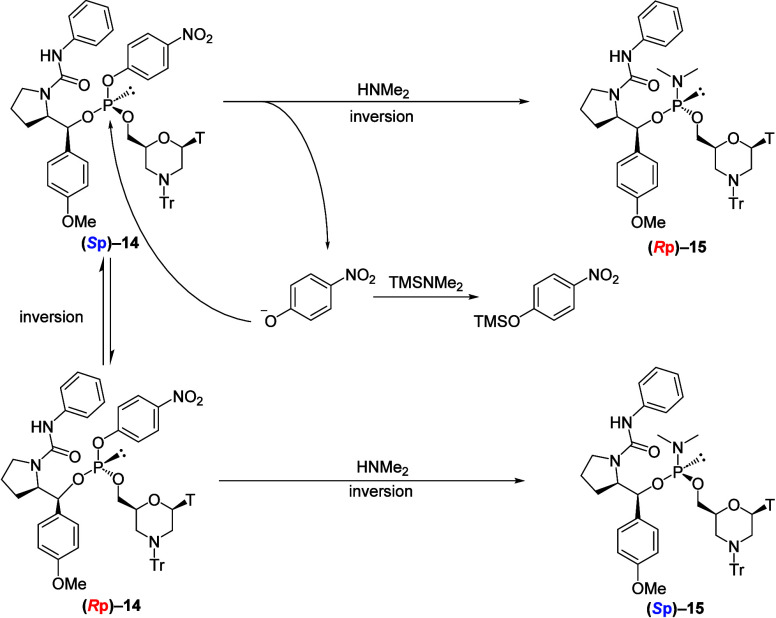
Plausible Mechanism Driving Stereoselectivity Loss

### Investigation of Phenol Derivatives

Subsequently, we investigated phenols with different substituents to improve stereoselectivity (Table 3). In entry 1, 4-cyanopheol, which exhibited the lowest acidity among the phenols under investigation, was used. However, the NMR yield of the desired product was low because the dimethylamination process did not proceed efficiently. Using the phenols with modest p*K*_a_ values (entries 2, 4, and 5) resulted in high stereoselectivity, with 3-methyl-4-nitrophenol and 4-nitrophenol producing preferable results. Although 2-nitrophenol exhibited a p*K*_a_ value similar to that of 4-nitrophenol, a lack of stereoselectivity was observed (entry 3, *S*p:*R*p = 38:62). The use of 2,4,6-trichlorophenol, which exhibited the highest acidity, resulted in a considerable decrease in stereoselectivity (entry 6, *S*p:*R*p = 43:57). According to these investigations, phenol derivatives with p*K*_a_ values ranging from 7.1 to 7.3 and no substituents on the ortho position yielded favorable results. Although 4-cyanophenol (p*K*_a_ = 7.97) appeared to exhibit a low leaving ability, which hampered dimethylamination, the excessive leaving ability of a phenol derivative with a low p*K*_a_ value may decrease in stereoselectivity. Furthermore, the steric hindrance of phenols with substitutions at the ortho position may result in low stereoselectivity or NMR yields. Based on these results, we selected 4-nitrophenol as the optimal phenol derivative.

**Table 3:**
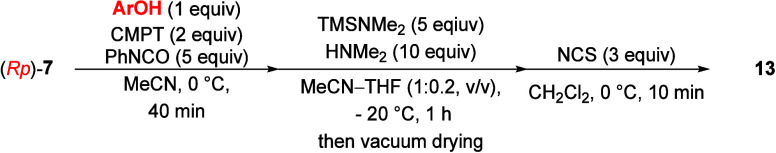
Investigation of Phenol Derivatives

entry	ArOH	p*K*_a_ of ArOH	dr of **13** *S*p:*R*p^a^	NMR yield (%)^a^
1	4-cyanophenol	7.97		22
2	3-methyl-4-nitrophenol	7.33	6:94	87
3	2-nitrophenol	7.23	38:62	46
4	4-nitrophenol	7.15	3:97	93
5	2,4,6-trifluorophenol	7.12	4:96	54
6	2,4,6-trichlorophenol	6.15	43:57	55

a Determined by ^31^P NMR after chlorination.

### Investigation of PhNCO Derivatives

The synthesis and isolation of the chloridate monomer were attempted under optimized conditions. However, the isolated yield of **13** was 16% even though the NMR yield was substantial (seeSupporting Information (SI), Figure S3). The low isolated yield was attributed to the difficulty in separating the chloridate monomer from the byproducts and the low stability of the product on neutral silica gel. Through the optimization of the purification conditions, we found that using diol-silica gel rather than neutral silica gel suppressed product decomposition. However, the separation of the chloridate monomers from the byproduct, whose chemical shift in the ^31^P NMR spectrum was 14 ppm, remained challenging. Through a control experiment, we demonstrated that the byproduct was formed via the reaction between the oxazaphospholidine derivative and NCS in the presence of CMPT (SI, Figure S6). We hypothesized that aryl phosphite derivatives without acylation on the pyrrolidine ring were converted into oxazaphospholidine derivatives under basic conditions in a dimethylamination step (Scheme 5). Subsequently, the chlorination by NCS yielded the byproduct. However, the ^31^P NMR spectrum of the reaction mixture after the condensation reaction (Figure 2a) shows complete acylation of the pyrrolidine ring. We hypothesized that acylation proceeded during NMR monitoring at RT, and the reaction was not completed at 0 °C.

**Scheme 5:**
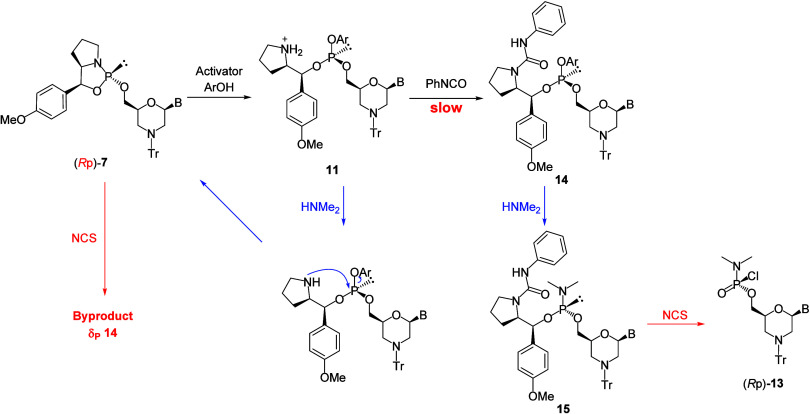
Plausible Mechanism for Byproduct Formation

Therefore, we anticipated that acylation with PhNCO would be sluggish and that more efficient acylating reagents would be required. To this end, four acylating reagents, namely, phenyl, 4-nitrophenyl, 2,4,6-trichlorophenyl, and 3,4-dichlorophenyl isocyanates, were investigated. The acylating rate of the pyrrolidine moiety was estimated from the integral ratios of **A** (*N*-free) and **B** (*N*-acylated) in the ^31^P NMR spectra using 1 equiv of the acylating reagent. The reactions were performed at 0 °C for 10, 20, and 60 min and monitored via ^31^P NMR at RT (Scheme 6). The results are presented in Figure 3. The acylation rate in the case of using PhNCO was only 28% after 1 h, and PhNCO exhibited the lowest reactivity among the four reagents. 3,4-Dichlorophenyl isocyanate was the most efficient reagent among the four reagents; thus, it was selected as the optimal reagent. According to the results, **13** was synthesized using 5 equiv of 3,4-dichlorophenyl isocyanate in the condensation step. The ^31^P NMR spectrum of the crude mixture indicated that changing the acylating reagent suppressed the formation of the byproduct (δ_P_ = 14) (SI, Figure S4). With the combination of an improved purification procedure, the isolated yield of **13** was improved to 36% (*S*p:*R*p = 4:96, Table 4, entry 1).

**Scheme 6:**
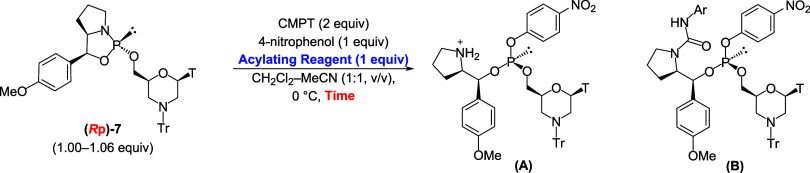
Investigation of Acylating Reagent

**Figure 3:**
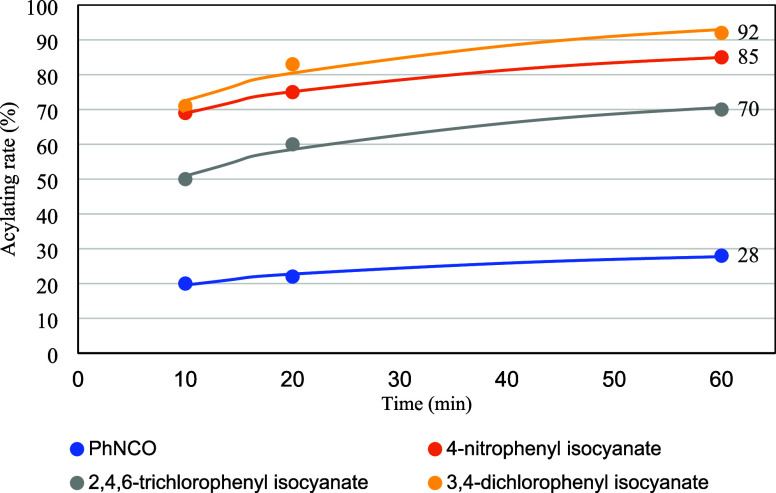
Investigation of acylating reagents.

**Table 4:**
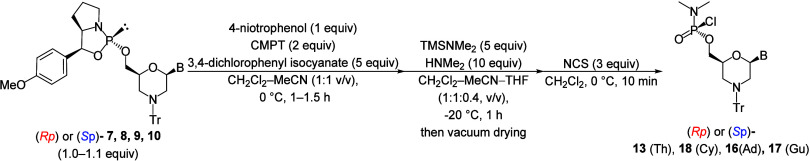
Synthesis of Chloridate Monomers under Optimal Conditions

entry	nucleobase	product	scale (mmol)	isolated yield (%)	dr of **13** *S*p:*R*p
1	Th	(*R*p)-**13**	0.30	36	4:96
2	Th	(*S*p)-**13**	0.30	51	95:5
3	Ad^bz^	(*R*p)-**16**	0.20	47	4:96
4	Ad^bz^	(*R*p)-**16**	1.0	52	5:95
5	Ad^bz^	(*S*p)-**16**	0.20	32	96:4
6	Gu^ce, ibu^	(*R*p)-**17**	0.20	39	5:95
7	Gu^ce, ibu^	(*S*p)-**17**	0.20	31	93:7
8	Cy^bz^	(*R*p)-**18**	0.20	–^b^	–^b^
9^a^	Cy^bz^	(*R*p)-**18**	0.20	47	3:97
10^a^	Cy^bz^	(*S*p)-**18**	0.20	46	97:3

a Dimethylketene methyl trimethylsilyl acetal was used instead of TMSNMe_2_.

b Byproduct was generated.

### Synthesis of Chloridate Monomers under Optimal Conditions

Furthermore, we synthesized the other stereoisomer **13** from the (*S*p)-oxazaphospholidine derivative (*S*p)-**7** with a high diastereomer ratio in a moderate yield (51% yield, *S*p:*R*p = 95:5, Table 4, entry 2). Subsequently, the same method was applied to other nucleotides. The chloridate monomers, namely, morpholino adenosine and guanosine derivatives, were obtained in moderate isolated yields (31–47%) with high stereoselectivity (dr = 93:7–96:4, Table 4, entries 3 and 5–7). Furthermore, the synthesis of (*R*p)-**16** on a 1.0 mmol scale was successfully performed, and the diastereomer ratio was comparable with that of the synthesis on a 0.20 mmol scale (52% yield, *S*p:*R*p = 5:95, entry 4), indicating that the present method is applicable for large-scale synthesis. For the cytidine derivatives, the main product was a byproduct with an amidine structure on the nucleobase protecting group (SI, Scheme S2). After rigorous investigation, we found that the silylating reagent played a pivotal role and that replacing TMSNMe_2_ with dimethylketene methyl trimethylsilyl acetal inhibited the side reaction (SI, Tables S1 and S2). Thus, dimethylketene methyl trimethylsilyl acetal was used for the synthesis of chloridate monomers with high stereoselectivity and moderate yields using cytidine derivatives (entries 9 and 10). As described, an efficient stereocontrolled method for the synthesis of chloridate monomers was developed. However, partial decomposition of the resulting chloridate monomers persisted during silica gel column chromatography. Therefore, further investigation into the purification conditions is required to improve the isolated yields.

### Synthesis of Stereocontrolled Dimers and Stereochemical Assignment of Their Phosphorus Stereochemistry

Subsequently, to determine the stereochemistry of chloridate monomers, the condensation reaction of chloridate monomers with an amino group of a 5′-*O*-TBDPS morpholino nucleoside was performed to obtain stereocontrolled 5′-N_PN_T-3′ (T_PN_T, A_PN_T, C_PN_T, and G_PN_T) dimers in the presence of a non-nucleophilic base, namely, *N*-ethylmorpholine or *i*Pr_2_NEt. The morpholino thymidine chloridate monomer (*R*p)-**13** (Table 5, entries 1, 3, 5, and 7) or (*S*p)-**13** (Table 5, entries 2, 4, 6, and 8) was used in the condensation reaction. Protected N_PN_T dimers ((*S*p)-**23**–**26**, (*R*p)-**23**–**26**) were obtained in moderate to high yields (58%–89%) with good stereoselectivity (dr = 91:9–98:2), indicating that the condensation reaction proceeded in a stereospecific manner. To obtain fully deprotected dimers, the deprotecting conditions were investigated using T_PN_T. First, the trityl protecting group on the amino group was removed under acidic conditions. Subsequently, the TBDPS group on the 5′-hydroxy group was removed by treatment with tetrabutylammonium fluoride (TBAF) (Scheme 7). However, the separation of a tetrabutylammonium salt from T_PN_T ((*S*p)-**27** or (*R*p)-**27**) was difficult. Consequently, multiple purifications were required, resulting in the low isolated yields of (*S*p)-**27** and (*R*p)-**27** (29%, Table 5, entry 1 and 49%, Table 5, entry 2, respectively). Based on these results, HF-Et_3_N was used in the desilylation of A_PN_T, C_PN_T, and G_PN_T rather than TBAF. After the removal of the TBDPS group, the deprotection of the acyl protecting groups on the nucleobases by ammonia treatment was performed. During this step, partial cleavage of the internucleotidic linkage was observed. This side reaction was notable, especially for C_PN_T dimers (SI, Figure S5). The difficulty in separating these byproducts from the desired dimers resulted in low isolated yields. Therefore, optimization of the deprotection conditions is necessary. Despite the low to moderate isolated yields (5%–55%, Table 5, entries 3–8), fully deprotected N_PN_T dimers ((*S*p)-**27**–**30**, (*R*p)-**27**–**30**) were obtained with high stereoselectivity (dr = 95:5–>99:1).

**Scheme 7:**
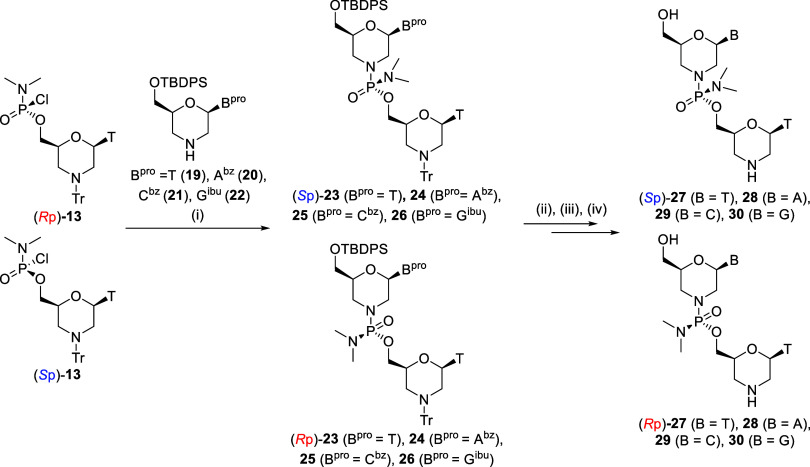
Synthesis of Dimers^a^

a (i) Chloridate monomer ((*R*p)-13 or (*S*p)-13) (1.0 equiv), morpholino nucleoside 19–22 (0.9–2.0 equiv), *N*-ethylmorpholine or *i*Pr_2_NEt (2.0 equiv), MeCN or THF, 1.5–17 h; (ii) 3-cyanopyridine (40 equiv), CF_3_COOH (40 equiv), CH_2_Cl_2_–CF_3_CH_2_OH (8:2, v/v), RT, 30–60 min; (iii) TBAF (40 equiv), THF, RT, 2 h, for (*S*p)-27 and (*R*p)-27, 3HF·Et_3_N (100 equiv), Et_3_N (200 equiv), THF, RT, 14–20 h, for (*S*p)-28–30 and (*R*p)-28–30; (iv) conc NH_3_aq–EtOH (3:1, v/v), 55 °C, 8–24 h, for (*S*p)-28–30 and (*R*p)-28–30.

**Table 5:** Synthesis of N_PN_T Dimer

	dr of **13**	protected N_PN_T dimer			fully deprotected N_PN_T dimer		
entry	*S*p:*R*p^a^	N_PN_T	*S*p:*R*p^a^	isolated yield (%)	N_PN_T	*S*p:*R*p^a^	isolated yield (%)
1	10:90	**23**	91:9	89	**27**	95:5	29
2	98:2	**23**	2:98	83	**27**	<1:99	49
3	3:97	**24**	94:6	58	**28**	97:3	25
4	98:2	**24**	2:98	89	**28**	2:98	55
5	3:97	**25**	95:5	75	**29**	97:3	30
6	97:3	**25**	4:96	74	**29**	<1:99	16
7	3:97	**26**	96:4	86	**30**	99:1	12
8	97:3	**26**	5:95	87	**30**	<1:99	5

a Determined by ^31^P NMR.

Finally, the stereochemistry of the fully deprotected dimer was determined via NMR analysis. The fully deprotected N_PN_T dimers were analyzed by 2D ^1^H–^1^H NOESY experiments for T_PN_T, A_PN_T, and G_PN_T or 2D-^1^H–^1^H ROESY experiments for C_PN_T in detail (SI, Figures S30–S37). A correlation between the signals of the 2′-H of the 5′-upstream nucleoside and the protons of the dimethylamino group was observed for the (*S*p)-**27**–**30** dimers. In contrast, a correlation between the 5′-H of the 3′-downstream nucleoside and the protons of the dimethylamino group was observed for the (*R*p)-**27**–**30** dimers. Molecular models of the stereodefined T_PN_T dimers were prepared based on the X-ray crystal structural analysis data of the T_PN_A dimers reported by Endo et al.^27^ (Figure 4). The models demonstrated that the dimethylamino groups of the *S*p- and *R*p-isomers were located near the 2′-H of the 5′-upstream nucleoside and the 5′-H of the 3′-downstream nucleoside, respectively. Referring to these models, the former and latter diastereomers were assigned as the *S*p- and *R*p-isomers, respectively. Based on the assumption that the condensation reaction of chlorophosphoramidate with an amino group proceeded with an inversion of the phosphorus stereochemistry, the phosphorus stereochemistry of the chloridate monomers was assigned *R*p and *S*p, respectively. Consequently, *R*p and *S*p chloridate monomers were synthesized from *R*p and *S*p 5′-oxazaphospholidine derivatives, respectively. The plausible stereocourse of the synthesis of the chloridate monomers was as follows: both the condensation reaction of the oxazaphospholidine derivative with the phenol derivative and dimethylamination proceeded with inversion, whereas oxidative chlorination resulted in the retention of phosphorus stereochemistry (Scheme 8).

**Figure 4:**
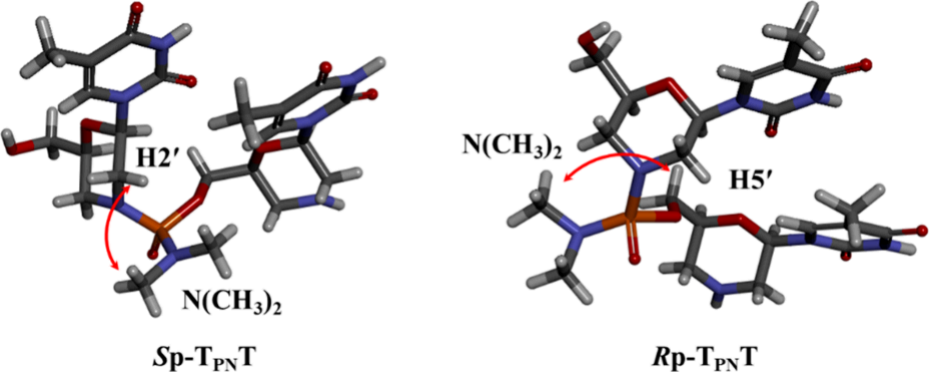
Molecular models of T_PN_T dimers (created from X-ray structural analysis data of T_PN_A dimers).

**Scheme 8:**
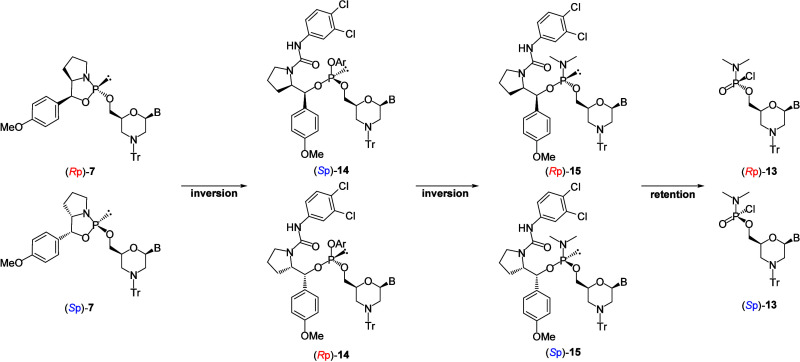
Stereocourse of Reactions

## Conclusions

The stereocontrolled synthesis of morpholino nucleoside 5′-dimethylamino phosphorochloridate monomers for the synthesis of stereopure PMO was achieved using 5′-oxazaphospholidine derivatives. The reaction yielded dimethylamino phosphorochloridate monomers for four morpholino nucleotides in moderate isolated yields with high stereoselectivity (>93:7). Although improvement of the isolated yield was necessary for practical use, this is the first example of the stereocontrolled synthesis of chloridate monomers from an oxazaphospholidine derivative. Furthermore, the proposed method affords stereocontrolled phosphoramidite derivatives; thus, it may be applicable for the stereocontrolled synthesis of other *P*-chiral nucleotide analogs. Moreover, the stereocontrolled synthesis of dimers was performed using the resulting chloridate monomers. Subsequently, the phosphorus stereochemistry was determined through NOESY and ROESY experiments. The stereochemistry of the chloridate monomers synthesized from *R*p and *S*p oxazaphospholidine derivatives was *R*p and *S*p stereoisomers, respectively. The NOESY and ROESY experiments can be readily conducted; thus, the proposed method can serve as a convenient means for assigning the phosphorus stereochemistry of PMOs. The results of this study contribute to the elucidation of the effect of phosphorus stereochemistry on the physicochemical and biological properties of PMOs.

## Experimental Section

### General Information

All reactions were conducted under an argon atmosphere. ^1^H NMR spectra were recorded using at 500 or 600 MHz with tetramethylsilane (TMS) as an internal standard (δ 0.00) in CDCl_3_ or deuterated solvent signal; D_2_O (δ 4.79). ^31^P NMR spectra were recorded at 162 or 202 MHz with 85% H_3_PO_4_ as an external standard (δ 0.0). ^13^C NMR spectra were recorded at 126 MHz with trimethylsilyl propanoic acid (δ 0.0) as an external standard, or the deuterated solvent signal; CDCl_3_ (δ 77.0) as an internal standard. HRMS data were obtained using a QTOF analyzer. Infrared (IR) spectra were recorded using attenuated total reflectance IR spectroscopy. Analytical TLC was performed on a commercial 0.25 mm-thick glass-plated silica gel layer. Reagents were purchased from Wako Pure Chemical Industries, Tokyo Chemical Industry, Sigma-Aldrich, and Sapala Organics. Manual silica gel column chromatography was performed on NH-silica gel (Chromatorex NH-DM1020) or DNH-silica gel (Fuji Silisia Chemical Ltd. CHROMATOREX (DNH MB 100–75/200)). Automated flash chromatography was performed on neutral silica gel (Yamazen UNIVERSAL Premium column (30 μm 60Å)), NH-silica gel (Yamazen UNIVERSAL Premium column (30 μm 60Å)), diol-silica gel (Yamazen UNIVERSAL column (40 μm 60Å)) (Yamazen Corporation), or octadecyl silica gel (Yamazen UNIVERSAL column (50 μm 120Å)) using an automated flash chromatography system W-prep 2XY (Yamazen Corporation). The organic solvents were purified and dried using the appropriate procedures.

### Synthesis of Oxazaphospholidine Derivatives

#### Synthesis of (4*R*,5*S*)*-* or (4*S,*5*R*)*-* **2**

The compounds were synthesized according to the method of ref 21 and used in the phosphitylation reactions without purification.

#### General Procedure for Synthesis of 5′-Oxazaphospholidine Morpholino Derivatives ((*R*p)-**7**–**10**, (*S*p)-**7**–**10**)

*N*-Trityl-morpholino thymidine **3** (2.43 g, 5.0 mmol for (*R*p)-**7**; 2.47 g, 5.1 mmol for (*S*p)-**7**), *N* ^4^-benzoyl-*N*-trityl-morpholino cytidine **4** (2.57 g, 4.5 mmol for (*R*p)-**8**; 2.50 g, 4.4 mmol, for (*S*p)-**8**), *N* ^6^-benzoyl-*N*-trityl-morpholino adenosine **5** (2.98 g, 5.0 mmol for (*R*p)-**9**; 2.98 g, 5.0 mmol for (*S*p)-**9**), or *N* ^2^-isobutyryl-*O* ^6^-cyanoethyl-*N*-trityl-morpholino guanosine **6** (1.46 g, 2.4 mmol for (*R*p)-**10**; 1.15 g, 1.8 mmol for (*S*p)-**10**) was dried by repeated coevaporation with pyridine, toluene, and THF and then dissolved in THF (10 mL for (*R*p)-**7**–**9** and (*S*p)-**7**–**9**; 5 mL for (*R*p)-**10**; 2.7 mL for (*S*p)-**10**). Et_3_N (4.9 mL, 35 mmol for (*R*p)-**7**–**9** and (*S*p)-**7**–**9**; 2.4 mL, 17 mmol for (*R*p)-**10**; 1.8 mL, 13 mmol for (*S*p)-**10**) was added to the mixture, and the mixture was cooled to −78 °C. A 1.3 M THF solution of (4*R*,5*S*)-**2** (10 mL, 13 mmol for (*R*p)-**7**–**9**; 5.0 mL, 6.5 mmol for (*R*p)-**10**), and (4*S*,5*R*)-**2** (10 mL, 13 mmol for (*S*p)-**8**, or **9**), or a 1.0 M THF solution of (4*S*,5*R*)-**2** (12 mL, 12 mmol for (*S*p)-**7**; 4.5 mL, 4.5 mmol for (Sp)-**10**) was added dropwise to the mixture and the mixture was gradually warmed to RT for (*R*p)-**7** or (*S*p)-**7**, or to 0 °C for (*R*p)-**8**–**10** or (*S*p)-**8**–**10**.

#### *R*p-T Oxazaphospholidine Derivative: (*R*p)-**7**

After 2.5 h, the mixture was diluted with CHCl_3_ (300 mL) and washed with saturated aqueous solutions of NaHCO_3_ (2 × 100 mL) and the aqueous layers were extracted using CHCl_3_ (200 mL). All organic layers were combined and washed with brine (100 mL). The aqueous layer was extracted with CHCl_3_ (100 mL), and the collected organic layers were combined, dried over Na_2_SO_4_ and filtered. The resulting mixture was diluted with toluene (5 mL) and concentrated under reduced pressure. The crude mixture containing (*R*p)-**7** was purified by automated silica gel column chromatography (NH-silica gel, 40 g, L size) using hexane–EtOAc (21:79–0:100, v/v) containing 1% Et_3_N as an eluent to afford (*R*p)-**7** (colorless foam, 2.24 g, 3.1 mmol, 62% yield).

^1^H NMR (500 MHz, CDCl_3_) δ 8.41 (s, 1H, H-3), 7.5–7.3 (br, 6H, Ar), 7.26 (d, *J* = 1.2 Hz, 1H, H-6), 7.23 (t, *J* = 7.7 Hz, 6H, Ar), 7.12 (t, *J* = 7.2 Hz, 3H, Ar), 7.06–7.04 (m, 2H, Ar), 6.87–6.82 (m, 2H, Ar), 6.12 (dd, *J* = 9.8, 2.3 Hz, 1H, H-1′), 5.37 (d, *J* = 6.2 Hz, 1H, 5-position of oxazaphospholidine), 4.29–4.25 (m, 1H, H-4′), 3.92–3.85 (m, 2H, H-5′, H-5″), 3.82 (s, 3H, OCH_3_), 3.80–3.76 (m, 1H, 4-position of oxazaphospholidine), 3.58–3.50 (m, 1H, pyrrolidine ring), 3.31 (dt, *J* = 11.2, 2.2 Hz, 1H, H-2′), 3.21–3.11 (m, 2H, H-3′, pyrrolidine ring), 1.79 (d, *J* = 1.1 Hz, 3H, 5-CH_3_), 1.66–1.60 (m, 2H, pyrrolidine ring), 1.53 (dd, *J* = 11.6, 10.8 Hz, 1H, H-3″), 1.39 (dd, *J* = 11.1, 9.8 Hz, 1H, H-2″), 1.21–1.15 (m, 1H, pyrrolidine ring), 0.97–0.89 (m, 1H, pyrrolidine ring); ^13^C­{^1^H} NMR (126 MHz, CDCl_3_) δ 163.4 (C-4), 158.9, 149.6 (C-2), 135.9 (C-6), 130.3, (d, ^3^ *J*_PC_ = 4.0 Hz, Ar) 129.1, 127.8, 126.6, 126.4, 113.5, 110.4 (C-5), 81.7 (d, ^2^ *J*_PC_ = 10.1 Hz, 5-position of oxazaphospholidine), 80.7 (C-1′), 77.2 (−C­(Ar)_3_), 75.9 (d, ^3^ *J*_PC_ = 2.0 Hz, C-4′), 67.5 (d, ^2^ *J*_PC_ = 2.9 Hz, 4-position of oxazaphospholidine), 64.8 (d, ^2^ *J*_PC_ = 14.7 Hz, C-5′), 55.3 (OCH_3_), 51.9 (C-2′), 49.2 (C-3′), 47.2 (d, ^2^ *J*_PC_ = 36.0 Hz, pyrrolidine ring), 28.0 (pyrrolidine ring), 26.0 (d, ^3^ *J*_PC_ = 3.5 Hz, pyrrolidine ring), 12.3 (5-CH_3_); ^31^P­{^1^H} NMR (202 MHz, CDCl_3_) δ 158.7; IR (neat, cm^–1^) 2940, 1682, 1513, 1447, 1247, 1032, 959, 744, 630, 575, 476, 418; high-resolution mass spectrometry (HRMS) (ESI-QTOF) *m*/*z*: [M + H]^+^ Calcd for C_41_H_44_N_4_O_6_P^+^, 719.2993; Found, 719.2994.

#### *S*p-T Oxazaphospholidine Derivative: (*S*p)-**7**

After 2 h, the mixture was diluted with CHCl_3_ (100 mL) and washed with saturated aqueous solutions of NaHCO_3_ (2 × 30 mL). The aqueous layers were combined and extracted using CHCl_3_ (100 mL), and the collected organic layers were combined, dried over Na_2_SO_4_, and filtered. The resulting mixture was diluted with toluene (5 mL) and concentrated under reduced pressure. The crude mixture containing (*S*p)-**7** was purified by automated silica gel column chromatography (NH-silica gel, 40 g, L size) using hexane–EtOAc (2:98–0:100, v/v) containing 1% Et_3_N as an eluent to afford (*S*p)-**7** (colorless foam, 2.42 g, 3.4 mmol, 66% yield).

^1^H NMR (500 MHz,CDCl_3_) δ 8.39 (s, 1H, H-3), 7.5–7.4 (br, 6H, Ar), 7.27–7.24 (m, 7H, Ar), 7.19–7.13 (m, 5H, Ar), 6.99 (d, *J* = 1.2 Hz, 1H, H-6), 6.90–6.87 (m, 2H, Ar), 6.10 (dd, *J* = 9.6, 2.3 Hz, 1H, H-1′), 5.67 (d, *J* = 6.3 Hz, 1H, 5-position of oxazaphospholidine), 4.30–4.25 (m, 1H, H-4′), 3.91–3.86 (m, 1H, H-5′), 3.81 (s, 3H, OCH_3_), 3.81–3.74 (m, 1H, 4-position of oxazaphospholidine), 3.69–3.64 (m, 1H, H-5″), 3.59–3.51 (m, 1H, pyrrolidine ring), 3.32 (dt, *J* = 9.1, 2.2 Hz, 1H, H-2′), 3.24 (dt, *J* = 11.9, 2.2 Hz, 1H, H-3′), 3.18–3.11 (m, 1H, pyrrolidine ring), 1.75 (d, *J* = 1.0 Hz, 3H, 5-CH_3_), 1.66–1.60 (m, 2H, pyrrolidine ring), 1.43–1.36 (m, 2H, H-2″, H-3′′), 1.20–1.14 (m, 1H, pyrrolidine ring), 0.99–0.91 (m, 1H, pyrrolidine ring); ^13^C­{^1^H} NMR (126 MHz, CDCl_3_) δ163.3 (C-4), 158.9 (Ar), 149.6 (C-2), 135.5 (C-6), 130.4 (d, ^3^ *J*_PC_ = 3.9 Hz), 129.2, 129.0, 127.8, 126.5, 126.4, 113.6, 110.4 (C-5), 82.0 (d, ^2^ *J*_PC_ = 9.4 Hz, 5-position of oxazaphospholidine), 80.5 (C-1′), 77.2 (−C­(Ar)_3_), 76.2 (d, ^3^ *J*_PC_ = 3.0 Hz, C-4′), 67.5 (d, ^2^ *J*_PC_ = 3.2 Hz, 4-positon of oxazaphospholidine), 63.9 (d, ^2^ *J*_PC_ = 12.2 Hz, C-5′), 55.3 (OCH_3_), 52.0 (C-2′), 49.9 (C-3′), 47.2 (d, ^2^ *J*_PC_ = 34.9 Hz, pyrrolidine ring), 28.0 (pyrrolidine ring), 26.0 (d, ^3^ *J*_PC_ = 3.2 Hz, pyrrolidine ring), 12.3 (5-CH_3_); ^31^P­{^1^H} NMR (202 MHz, CDCl_3_) δ 156.5; IR (neat, cm^–1^) 2936, 1683, 1514, 1448, 1247, 1033, 959, 744, 710, 630, 575, 478, 439, 416; HRMS (ESI-QTOF) *m*/*z*: [M + H]^+^Calcd for C_41_H_44_N_4_O_6_P^+^, 719.2993; Found, 719.2994.

#### *R*p-C^bz^ Oxazaphospholidine Derivative: (*R*p)-**8**

After 1 h, the mixture was diluted with toluene (10 mL) at 0 °C and washed with saturated aqueous solutions of NaHCO_3_. The organic layers were evaporated to remove THF at 91 mbar. Then, the mixture was diluted using CH_2_Cl_2_ and washed with saturated aqueous solutions of NaHCO_3_. The aqueous layers were extracted with CH_2_Cl_2_, and the collected organic layers were combined, washed with brine, dried over Na_2_SO_4_, and filtered. The resulting mixture was diluted with toluene (5 mL) and concentrated under reduced pressure. The crude mixture containing (*R*p)-**8** was purified by silica gel column chromatography (DNH-silica gel, 101 g) using hexane–EtOAc (1:3–1:5, v/v) containing 1% Et_3_N as an eluent to afford (*R*p)-**8** (colorless foam, 2.75 g, 3.4 mmol, 76% yield).

^1^H NMR (500 MHz,CDCl_3_) δ 7.94 (d, *J* = 7.4 Hz, 1H, H-6), 7.86 (d, *J* = 7.5 Hz, 2H, Ar), 7.58 (tt, *J* = 7.4, 1.1 Hz, 1H, Ar), 7.52–7.34 (m, 7H, Ar), 7.26–7.23 (m, 8H, Ar, H-5), 7.16–7.13 (m, 3H, Ar), 7.07–7.04 (m, 2H, Ar), 6.90 (dt, *J* = 8.7, 2.4 Hz, 2H, Ar), 6.27 (dd, *J* = 9.2, 2.2 Hz, 1H, H-1′), 5.31 (d, *J* = 6.2 Hz, 1H, 5-position of oxazaphospholidine), 4.28–4.24 (m, 1H, H-4′), 4.03–3.98 (m, 1H, H-5′), 3.91–3.86 (m, 1H, H-5″), 3.82 (s, 3H, OCH_3_), 3.80–3.72 (m, 1H, 4-position of oxazaphospholidine), 3.59–3.51 (m, 1H, pyrrolidine ring), 3.47 (dt, *J* = 11.1, 2.3 Hz, 1H, H-2′), 3.22–3.15 (m, 1H, pyrrolidine ring), 3.12 (dt, *J* = 12.0, 2.3 Hz, 1H, H-3′), 1.68–1.62 (m, 3H, pyrrolidine ring, H-3′), 1.21–1.11 (m, 2H, H-2′, pyrrolidine ring), 1.01–0.93 (m, 1H, pyrrolidine ring); ^13^C­{^1^H} NMR (126 MHz, CDCl_3_) δ 166.4 (−NHCO−), 161.9 (C-4), 159.0, 154.1 (C-2), 145.4 (C-6), 133.1, 129.9 (d, ^3^ *J*_PC_ = 4.0 Hz), 129.2, 129.0, 127.7, 127.5, 126.7, 126.4, 125.3, 113.6 (Ar), 96.4 (C-5), 82.1 (C-1′), 81.4 (d, ^2^ *J*_PC_ = 9.5 Hz, 5-position of oxazaphospholidine), 76.8 (−C­(Ar)_3_), 75.9 (d, ^3^ *J*_PC_ = 1.1 Hz, C-4′), 67.6 (d, ^3^ *J*_PC_ = 2.9 Hz, 4-position of oxazaphospholidine), 65.2 (d, ^2^ *J*_PC_ = 16.4 Hz, C-5′), 55.2 (OCH_3_), 52.6 (C-2′), 49.0 (C-3′), 47.3 (d, ^2^ *J*_PC_ = 35.1 Hz, pyrrolidine ring), 27.9 (pyrrolidine ring), 26.0 (d, ^3^ *J*_PC_ = 3.5 Hz, pyrrolidine ring); ^31^P­{^1^H} NMR (202 MHz, CDCl_3_) δ 159.6; IR (neat, cm^–1^) 2937, 1683, 1514, 1448, 1247, 1033, 959, 746, 710, 630, 576, 437, 417; HRMS (ESI-QTOF) *m*/*z*: [M + H]^+^ Calcd for C_47_H_47_N_5_O_6_P^+^, 808.3259; Found, 808.3256.

#### *S*p-C^bz^ Oxazaphospholidine Derivative: (*S*p)-**8**

After 1 h, the mixture was diluted with toluene (100 mL) at 0 °C and washed with saturated aqueous solutions of NaHCO_3_ (250 mL). The organic layers were evaporated to remove THF at 91 mbar. Then, the mixture was diluted with CH_2_Cl_2_ (100 mL) and washed with saturated aqueous solutions of NaHCO_3_ (250 mL). The aqueous layers were extracted with CH_2_Cl_2_ (50 mL), and the collected organic layers were combined, washed with brine (250 mL), dried over Na_2_SO_4_, and filtered. The resulting mixture was diluted with toluene (5 mL) and concentrated under reduced pressure. The crude mixture containing (*S*p)-**8** was purified by silica gel column chromatography (DNH-silica gel, 107 g) using hexane–EtOAc (1:3–1:5, v/v) containing 1% Et_3_N as an eluent to afford (*S*p)-**8** (colorless foam, 2.49 g, 3.1 mmol, 71% yield).

^1^H NMR (500 MHz,CDCl_3_) δ 7.87 (d, *J* = 7.5 Hz, 2H, Ar), 7.64 (d, *J* = 7.5 Hz, 1H, H-6), 7.61–7.58 (tt, *J* = 7.4, 1.2 Hz, 1H, Ar), 7.51–7.36 (m, 7H, Ar), 7.27–7.24 (m, 8H, Ar, H-5), 7.16–7.14 (m, 6H, Ar), 6.85 (dt, *J* = 8.8, 2.4 Hz, 2H, Ar), 6.24 (dd, *J* = 9.2, 2.1 Hz, 1H, H-1′), 5.67 (d, *J* = 6.3 Hz, 1H, 5-position of oxazaphospholidine), 4.35–4.30 (m, 1H, H-4′), 3.90–3.85 (m, 1H, H-5′), 3.82–3.69 (m, 5H, H-5″, 4-position of oxazaphospholidine, OCH_3_), 3.60–3.52 (m, 2H, H-2′, pyrrolidine ring), 3.22 (dt, *J* = 11.9, 2.1 Hz, 1H, H-3′), 3.18–3.12 (m, 1H, pyrrolidine ring), 1.66–1.61 (m, pyrrolidine ring), 1.47–1.42 (dd, *J* = 11.6, 10.4 Hz, 1H, H-3″), 1.26–1.22 (dd, *J* = 11.1, 9.3 Hz, 1H, H-2″), 1.19–1.12 (m, 1H, pyrrolidine ring), 0.99–0.91 (m, 1H, pyrrolidine ring);^13^C­{^1^H} NMR (126 MHz, CDCl_3_) δ 166.2 (−NHCO−), 161.9 (C-4), 158.9, 154.0 (C-2), 144.5 (C-6), 133.1, 130.3 (d, ^3^ *J*_PC_ = 3.7. Hz), 129.2, 129.0, 127.8, 127.5, 126.6, 126.4, 113.6 (Ar), 96.2 (C-5), 82.0 (C-1′), 81.8 (d, ^2^ *J*_PC_ = 9.5 Hz, 5-position of oxazaphospholidine), 76.9 (−C­(Ar)_3_), 76.2 (d, ^3^ *J*_PC_ = 2.5 Hz, C-4′), 67.5 (d, ^3^ *J*_PC_ = 3.2 Hz, 4-position of oxazaphospholidine), 64.0 (d, ^2^ *J*_PC_ = 12.5 Hz, C-5′), 55.1 (OCH_3_), 52.8 (C-2′), 49.8 (C-3′), 47.2 (d, ^2^ *J*_PC_ = 34.8 Hz, pyrrolidine ring), 28.0 (pyrrolidine ring), 26.0 (d, ^3^ *J*_PC_ = 3.5 Hz, pyrrolidine ring); ^31^P­{^1^H} NMR (202 MHz, CDCl_3_) δ 156.1; IR (neat, cm^–1^) 2932, 1682, 1514, 1448, 1247, 1025, 959, 744, 710, 630, 574, 438, 417; HRMS (ESI-QTOF) *m*/*z*: [M + H]^+^ Calcd for C_47_H_47_N_5_O_6_P^+^, 808.3259; Found, 808.3264.

#### *R*p-A^bz^ Oxazaphospholidine Derivative: (*R*p)-**9**

After 2 h, the mixture was diluted with CHCl_3_ (100 mL) and washed with a saturated aqueous solution of NaHCO_3_ (50 mL) and brine (50 mL). The aqueous layers were combined and extracted using CHCl_3_ (100 mL), and the collected organic layers were combined, dried over Na_2_SO_4_ and filtered. The resulting mixture was diluted with toluene (5 mL) and concentrated under reduced pressure. The crude mixture containing (*R*p)-**9** was purified by silica gel column chromatography (NH-silica gel, 95 g) using hexane–EtOAc (1:3 v/v) containing 1% Et_3_N as an eluent to afford (*R*p)-**9** (colorless foam, 1.30 g, 1.6 mmol, 31% yield).

^1^H NMR (500 MHz,CDCl_3_) δ 9.02 (br, 1H, −CONH−), 8.79 (s, 1H, H-2), 8.16 (s, 1H, H-8), 8.00 (d, *J* = 7.3 Hz, 2H, Ar), 7.58 (tt, *J* = 7.4, 1.2 Hz, 1H, Ar), 7.52–7.38 (m, 8H, Ar), 7.26–7.23 (m, 6H, Ar), 7.14 (t,, *J* = 7.3 Hz, 3H, Ar), 7.09–7.06 (d, *J* = 8.6 Hz, 2H, Ar), 6.87 (dt, *J* = 8.6, 2.5 Hz, 2H, Ar), 6.42 (dd, *J* = 9.8, 2.1 Hz, 1H, H-1′), 5.41 (d, *J* = 6.2 Hz, 1H, 5-position of oxazaphospholidine), 4.41–4.37 (m, 1H, H-4′), 3.89 (dd, *J* = 10.8, 4.7 Hz, 2H, H-5′, H-5″), 3.81 (s, 3H, OCH_3_), 3.78–3.74 (m, 1H, 4-position of oxazaphospholidine), 3.56–3.49 (m, 1H, pyrrolidine ring), 3.45 (dt, *J* = 11.3, 1.4 Hz, 1H, H-2′), 3.23 (dt, *J* = 12.1, 2.1 Hz, 1H, H-3′), 3.17–3.10 (m, 1H, pyrrolidine ring), 1.77 (dd, *J* = 11.0, 10.1 Hz, 1H, H-2″), 1.67–1.58 (m, 3H, H-3′′, pyrrolidine ring), 1.16–1.10 (m, 1H, pyrrolidine ring), 0.96–0.88 (m, 1H, pyrrolidine ring); ^13^C­{^1^H} NMR (126 MHz, CDCl_3_) δ 164.5 (−NHCO−), 158.9, 152.7 (C-2), 151.1 (C-4), 149.3 (C-6), 141.1 (C-8), 133.7, 132.7, 130.2 (d, ^3^ *J*_PC_ = 4.2 Hz), 129.1, 128.8, 127.8, 126.6, 126.5, 122.6 (C-5), 113.5 (Ar), 81.6 (d, ^2^ *J*_PC_ = 9.4 Hz, 5-position of oxazaphospholidine), 80.2 (C-1′), 76.8 (−C­(Ar)_3_), 75.9 (d, ^3^ *J*_PC_ = 2.2 Hz, C-4′), 67.6 (d, ^3^ *J*_PC_ = 2.9 Hz, 4-position of oxazaphospholidine), 64.6 (d, ^2^ *J*_PC_ = 14.3 Hz, C-5′), 55.2 (OCH_3_), 53.2 (C-2′), 49.4 (C-3′), 47.2 (d, ^2^ *J*_PC_ = 35.2 Hz, pyrrolidine ring), 27.9 (pyrrolidine ring), 25.9 (d, ^3^ *J*_PC_ = 3.5 Hz, pyrrolidine ring); ^31^P­{^1^H} NMR (202 MHz, CDCl_3_) δ 158.8; IR (neat, cm^–1^) 2933, 1683, 1613, 1514, 1448, 1303, 1246, 1175, 1100, 1026, 959, 744, 710, 645, 630, 563, 437, 418; HRMS (ESI-QTOF) *m*/*z*: [M + H]^+^ Calcd for C_48_H_47_N_7_O_5_P^+^, 832.3371; Found, 832.3374.

#### *S*p-A^bz^ Oxazaphospholidine Derivative: (*S*p)-**9**

After 3.5 h, the mixture was diluted with CHCl_3_ (100 mL) and washed with a saturated aqueous solution of NaHCO_3_ (50 mL) and brine (50 mL). The aqueous layers were combined and extracted with CHCl_3_ (100 mL), and the collected organic layers were combined, dried over Na_2_SO_4_, and filtered. The resulting mixture was diluted with toluene (5 mL) and concentrated under reduced pressure. The crude mixture containing (*S*p)-**9** was purified by silica gel column chromatography twice (NH-silica gel, 80 g) using hexane–EtOAc (1:2 v/v) containing 1% Et_3_N as an eluent for the first time, (NH-silica gel, 40 g) using hexane–EtOAc (2:3 v/v) containing 1% Et_3_N as an eluent for the second time to afford (*S*p)-**9** (colorless foam, 1.1 g, 1.3 mmol, 26% yield).

^1^H NMR (500 MHz,CDCl_3_) δ 8.98 (br, 1H, −CONH−), 8.80 (s, 1H, H-2), 8.00 (d, *J* = 7.4 Hz, 2H, Ar), 7.96 (s, 1H, H-8), 7.58 (tt, *J* = 7.4, 1.8 Hz, 1H, Ar), 7.52–7.42 (m, 7H, Ar), 7.28–7.25 (m, 8H, Ar), 7.18–7.13 (m, 5H, Ar), 6.86 (dt, *J* = 8.8, 2.4 Hz, 2H, Ar), 6.39 (dd, *J* = 9.9, 2.3 Hz, 1H, H-1′), 5.65 (d, *J* = 6.3 Hz, 1H, 5-position of oxazaphospholidine), 4.40–4.36 (m, 1H, H-4′), 3.97–3.91 (m, 1H, H-5′), 3.79 (s, 3H, OCH_3_), 3.78–3.75 (m, 1H, 4-position of oxazaphospholidine), 3.73–3.67 (m, 1H, H-5″), 3.58–3.50 (m, 1H, pyrrolidine ring), 3.48 (dt, *J* = 11.3, 2.2 Hz, 1H, H-2′), 3.35 (dt, *J* = 12.0, 2.2 Hz, 1H, H-3′), 3.17–3.10 (m, 1H, pyrrolidine ring), 1.81 (dd, *J* = 11.1, 10.2 Hz, 1H, H-2″), 1.64–1.54 (m, 3H, pyrrolidine ring, H-3′′), 1.18–1.12 (m, 1H, pyrrolidine ring), 0.96–0.88 (m, 1H, pyrrolidine ring); ^13^C­{^1^H} NMR (126 MHz, CDCl_3_) δ 164.5 (−NHCO−), 158.8 (Ar), 152.7 (C-2), 151.1 (C-4), 149.3 (C-6), 140.6 (C-8), 133.6, 132.7, 130.4 (d, ^3^ *J*_PC_ = 3.8 Hz), 129.2, 128.8, 127.9, 127.8, 127.0, 126.5 126.4, 122.6 (C-5), 113.5 (Ar), 82.0 (d, ^2^ *J*_PC_ = 9.5 Hz, 5-position of oxazaphospholidine), 80.0 (C-1′), 76.9 (−C­(Ar)_3_), 76.2 (d, ^3^ *J*_PC_ = 2.7 Hz, C-4′), 67.5 (d, ^3^ *J*_PC_ = 3.0 Hz, 4-position of oxazaphospholidine), 64.0 (d, ^2^ *J*_PC_ = 13.4 Hz, C-5′), 55.2 (OCH_3_), 53.2 (C-2′), 50.0 (C-3′), 47.2 (d, ^2^ *J*_PC_ = 34.9 Hz, pyrrolidine ring), 28.0 (pyrrolidine ring), 25.9 (d, ^3^ *J*_PC_ = 3.1 Hz, pyrrolidine ring); ^31^P­{^1^H} NMR (202 MHz, CDCl_3_) δ 156.9; IR (neat, cm^–1^) 2928, 1683, 1613, 1513, 1448, 1246, 1175, 1099, 1026, 959, 744, 710, 630, 562, 437, 408; HRMS (ESI-QTOF) *m*/*z*: [M + H]^+^ Calcd for C_48_H_47_N_7_O_5_P^+^, 832.3371; Found, 832.3372.

#### *R*p-G^ce, ibu^ Oxazaphospholidine Derivative: (*R*p)-**10**

After 2.5 h, the mixture was diluted with CHCl_3_ (100 mL) and washed with saturated aqueous solutions of NaHCO_3_ (2 × 30 mL). The aqueous layers were combined and extracted using CHCl_3_ (100 mL), and the collected organic layers were combined, dried over Na_2_SO_4_, and filtered. The resultant mixture was diluted with toluene (5 mL) and concentrated under reduced pressure. The crude mixture containing (*R*p)-**10** was purified by silica gel column chromatography (NH-silica gel) using hexane–EtOAc (1:3 v/v) as an eluent to afford (*R*p)-**10** (colorless foam, 0.66 g, 0.76 mmol, 33% yield).

^1^H NMR (500 MHz,CDCl_3_) δ 7.99 (s, 1H, H-8), 7.79 (s, 1H, −CONH−), 7.5–7.4 (br, 6H, Ar), 7.24 (t, *J* = 7.7 Hz, 6H, Ar), 7.16–7.13 (m, 3H, Ar), 7.09–7.06 (m, 2H, Ar), 6.86 (dt, *J* = 8.8, 2.3 Hz, 2H, Ar), 6.22 (dd, *J* = 9.8, 2.3 Hz, 1H, H-1′), 5.40 (d, *J* = 6.2 Hz, 1H, 5-position of oxazaphospholidine), 4.81–4.72 (m, 2H, −OCH_2_CH_2_−), 4.37–4.33 (m, 1H, H-4′), 3.87 (dd, *J* = 10.8, 4.8 Hz, 2H, H-5′, H-5″), 3.81 (s, 3H, OCH_3_), 3.78–3.73 (m, 1H, 4-position of oxazaphospholidine), 3.56–3.48 (m, 1H, pyrrolidine ring), 3.41 (dt, *J* = 11.2, 2.3 Hz, 1H, H-2′), 3.21 (dt, *J* = 12.1, 2.2 Hz, 1H, H-3′), 3.17–3.10 (m, 1H, pyrrolidine ring), 3.08–3.04 (m, 1H, −CH­(CH_3_)_2_), 3.01–2.99 (td, 6.7, 1.1 Hz, 2H, −OCH_2_CH_2_−), 1.70 (dd, *J* = 1.1, 10.1 Hz, 1H, H-2″), 1.64–1.58 (m, 3H, H-3′′, pyrrolidine ring), 1.36 (d, *J* = 6.9 Hz, 3H, −CH­(CH_3_)_2_), 1.34 (d, *J* = 6.9 Hz, 3H, −CH­(CH_3_)_2_), 1.15–1.09 (m, 1H, pyrrolidine ring), 0.96–0.88 (m, 1H, pyrrolidine ring); ^13^C­{^1^H} NMR (126 MHz, CDCl_3_) δ 175.6 (−NHCO−), 159.5 (C-2), 158.9 (C-6), 152.4 (C-4), 151.6, 140.1 (C-8), 130.1 (d, ^3^ *J*_PC_ = 3.9 Hz), 129.1, 127.8, 127.0, 126.6, 126.5, 117.4 (C-5), 116.8 (CN), 113.7, 113.5 (Ar), 81.7 (d, ^2^ *J*_PC_ = 9.5 Hz, 5-position of oxazaphospholidine), 80.5 (C-1′), 76.8 (−C­(Ar)_3_), 75.8 (d, ^3^ *J*_PC_ = 2.8 Hz, C-4′), 67.6 (d, ^3^ *J*_PC_ = 3.6 Hz, 4-position of oxazaphospholidine), 64.6 (d, ^2^ *J*_PC_ = 14.3 Hz, C-5′), 61.5 (−OCH_2_CH_2_CN), 55.2 (OCH_3_), 53.0 (C-2′), 49.4 (C-3′), 47.2 (d, ^2^ *J*_PC_ = 35.4 Hz, pyrrolidine ring), 35.8 (−CH­(CH_3_)_2_), 27.9 (pyrrolidine ring), 25.9 (d, ^3^ *J*_PC_ = 3.5 Hz, pyrrolidine ring), 19.4 (−CH­(CH_3_)_2_−), 19.3 (−CH­(CH_3_)_2_), 18.1 (−OCH_2_ CH_2_CN); ^31^P­{^1^H} NMR (202 MHz, CDCl_3_) δ 158.9; IR (neat, cm^–1^) 2966, 1688, 1610, 1513, 1447, 1385, 1244, 1100, 1026, 960, 745, 710, 630, 437, 408; HRMS (ESI-QTOF) *m*/*z*: [M + H]^+^ Calcd for C_48_H_52_N_8_O_6_P^+^, 867.3742; Found, 867.3741.

#### *S*p-G^ce, ibu^ Oxazaphospholidine Derivative: (*S*p)-**10**

After 2.5 h, the mixture was diluted with CHCl_3_ (100 mL) and washed with a saturated aqueous solution of NaHCO_3_ (50 mL). The aqueous layer was extracted using CHCl_3_ (50 mL), and the collected organic layers were combined, dried over Na_2_SO_4_, and filtered. The resulting mixture was diluted with toluene (5 mL) and concentrated under reduced pressure. The crude mixture containing (*S*p)-**10** was purified by silica gel column chromatography (NH-silica gel) using hexane–EtOAc (1:3 v/v) as an eluent to afford (*S*p)-**10** (colorless foam, 0.66 g, 0.76 mmol, 42% yield).

^1^H NMR (500 MHz,CDCl_3_) δ 7.81 (s, 1H, H-8), 7.79 (s, 1H, −CONH−), 7.5–7.4 (br, 6H, Ar), 7.25 (t, *J* = 7.7 Hz, 6H, Ar), 7.19–7.13 (m, 5H, Ar), 6.89 (dt, *J* = 8.7, 2.4 Hz, 2H, Ar), 6.19 (dd, *J* = 9.8, 2.3 Hz, 1H, H-1′), 5.66 (d, *J* = 6.4 Hz, 1H, 5-position of oxazaphospholidine), 4.82–4.71 (m, 2H, −OCH_2_CH_2_−), 4.36–4.31 (m, 1H, H-4′), 3.96–3.91 (m, 1H, H-5′), 3.82 (s, 3H, OCH_3_), 3.81–3.73 (m, 1H, 4-position of oxazaphospholidine), 3.70–3.64 (m, 1H, H-5″), 3.59–3.51 (m, 1H, pyrrolidine ring), 3.44 (dt, *J* = 11.3, 2.1 Hz, 1H, H-2′), 3.34 (dt, *J* = 12.0, 2.1 Hz, 1H, H-3′), 3.22–3.05 (m, 2H, pyrrolidine ring, −CH­(CH_3_)_2_), 3.00 (td, *J* = 6.6, 1.3 Hz, 2H, −OCH_2_CH_2_−), 1.77 (dd, *J* = 11.1, 10.2 Hz, 1H, H-2″), 1.67–1.58 (m, 2H, pyrrolidine ring), 1.52 (dd, *J* = 11.8, 10.6 Hz, 1H, H-3″), 1.36 (d, *J* = 6.8 Hz, 3H, −CH­(CH_3_)_2_), 1.34 (d, *J* = 6.8 Hz, 3H, −CH­(CH_3_)_2_), 1.20–1.13 (m, 1H, pyrrolidine ring), 0.97–0.90 (m, 1H, pyrrolidine ring); ^13^C­{^1^H} NMR (126 MHz, CDCl_3_) δ 175.6 (−NHCO−), 159.5 (C-2), 158.9 (C-6), 152.5 (C-4), 151.7, 139.7 (C-8), 130.4 (d, ^3^ *J*_PC_ = 3.9 Hz), 129.2, 127.8, 127.0, 126.5, 117.5 (C-5), 116.8 (CN), 113.6 (Ar), 82.1 (d, ^2^ *J*_PC_ = 9.6 Hz, 5-position of oxazaphospholidine), 80.4 (C-1′), 76.9 (−C­(Ar)_3_), 76.1 (d, ^3^ *J*_PC_ = 2.8 Hz, C-4′), 67.5 (d, ^3^ *J*_PC_ = 3.2 Hz, 4-position of oxazaphospholidine), 64.0 (d, ^2^ *J*_PC_ = 13.5 Hz, C-5′), 61.5 (−OCH_2_CH_2_CN), 55.3 (OCH_3_), 53.0 (C-2′), 50.1 (C-3′), 47.2 (d, ^2^ *J*_PC_ = 34.9 Hz, pyrrolidine ring), 35.8 (−CH­(CH_3_)_2_), 28.0 (pyrrolidine ring), 25.9 (d, ^3^ *J*_PC_ = 3.2 Hz, pyrrolidine ring), 19.4 (−CH­(CH_3_)_2_), 19.3 (−CH­(CH_3_)_2_), 18.1 (−OCH_2_ CH_2_CN); ^31^P­{^1^H} NMR (202 MHz, CDCl_3_) δ 157.1; IR (neat, cm^–1^) 2966, 1610, 1513, 1440, 1384, 1243, 1100, 1020, 960, 744, 710, 629, 444, 417, 405; HRMS (ESI-QTOF) *m*/*z*: [M + H]^+^ Calcd for C_48_H_52_N_8_O_6_P^+^, 867.3742; Found, 867.3750.

### Synthesis of Dimethylamino Phosphorochloridate Monomers

#### General Procedure for Synthesis of Dimethylamino Phosphorochloridate Monomers ((*R*p)-**13**, (*S*p)-**13**)

The 5′-oxazaphospholidine derivative (*R*p)-**7** (0.26 g, 0.36 mmol) or (*S*p)-**7** (0.25 g, 0.35 mmol) was dissolved in CH_2_Cl_2_ (3.5 mL for (*R*p)-**13** or (*S*p)-**13**) and dried over MS 4A for 1 d (solution A). In another vessel, a mixture of 4-nitrophenol (54 mg, 0.39 mmol for (*R*p)-**13** or 0.11 g, 0.81 mmol for (*S*p)-**13**) and CMPT (0.21 g, 0.79 mmol for (*R*p)-**13** or 0.42 g, 1.6 mmol for (*S*p)-**13**) in MeCN (4.0 mL for (*R*p)-**13** or 8.0 mL for (*S*p)-**13**) was dried over MS 3A for 1 d (solution B). Solution A (3.0 mL) was transferred to a round-bottom flask and cooled to 0 °C. Subsequently, 3,4-dichlorophenyl isocyanate (0.28 g, 1.5 mmol) and solution B (3.0 mL) were added to the mixture. After stirring at 0 °C for 1 h, the mixture was cooled to −20 °C. TMSNMe_2_ (0.24 mL, 1.5 mmol), and a 2.0 M THF solution of Me_2_NH (1.5 mL, 3.0 mmol) was added to the reaction mixture and stirred at −20 °C for 1 h. Subsequently, the volatiles were removed under reduced pressure at 0 °C. The residue was dissolved in CH_2_Cl_2_ (6.0 mL) and cooled to 0 °C. Subsequently, NCS (0.12 g, 0.86 mmol) was added to the mixture.

#### *R*p-T-Chloridate Monomer (*R*p)-**13**

After stirring for 10 min, the mixture was diluted with CH_2_Cl_2_ (50 mL) and washed with a 1.0 M NaH_2_PO_4_ aqueous solution (50 mL) and brine (50 mL). The aqueous layers were combined and extracted with CH_2_Cl_2_ (100 mL), and the collected organic layers were combined, dried over Na_2_SO_4_, filtered, and concentrated under reduced pressure. CH_2_Cl_2_ (2.0 mL) was added to the residue. The insoluble residue was removed via suction filtration and washed with CH_2_Cl_2_ (4.0 mL). Subsequently, the filtrate was concentrated under reduced pressure. The crude product was purified by automated silica gel column chromatography. An initial column (diol-silica gel, 37 g, L size) eluted with hexane–EtOAc (70:30–50:50, v/v) was followed by a second column (diol-silica gel, 14 g, M size) eluted with the same solvent gradient, affording (*R*p)-**13** (colorless foam, 63.9 mg, 0.10 mmol, 36% yield, dr = 4:96).

^1^H NMR (500 MHz, CDCl_3_) δ 8.76 (s, 1H, H-3), 7.5–7.4 (br, 6H, Ar), 7.30 (t, *J* = 7.6 Hz, 6H, Ar), 7.20 (t, *J* = 6.9 Hz, 3H, Ar), 7.05 (d, *J* = 1.2 Hz, 1H, H-6), 6.14 (dd, *J* = 9.6, 2.3 Hz, 1H, H-1′), 4.43–4.38 (m, 1H, H-4′), 4.13–4.10 (m, 2H, H-5′, H-5″), 3.38 (dt, *J* = 11.3, 2.3 Hz, 1H, H-2′), 3.16 (dt, *J* = 11.8, 2.5 Hz, 1H, H-3′), 2.66 (s, 3H, −N­(CH_3_)_2_), 2.64 (s, 3H, −N­(CH_3_)_2_), 1.84 (d, *J* = 1.0 Hz, 3H, 5-CH_3_), 1.50 (dd, *J* = 11.6, 10.8 Hz, 1H, H-3″), 1.43–1.39 (dd, *J* = 11.3, 9.7 Hz, 1H, H-2″); ^13^C­{^1^H} NMR (126 MHz, CDCl_3_) δ 163.4 (C-4), 149.7 (C-2), 135.3 (C-6), 129.1, 127.9, 126.6 (Ar), 110.6 (C-5), 80.5 (C-1′), 74.6 (d, ^3^ *J*_PC_ = 8.4 Hz, C-4′), 67.1 (d, ^2^ *J*_PC_ = 6.4 Hz, C-5′), 51.8 (C-2′), 48.8 (C-3′), 36.6 (d, ^2^ *J*_PC_ = 2.8 Hz, −N­(CH_3_)_2_) 12.3 (5-CH_3_); ^31^P­{^1^H} NMR (202 MHz, CDCl_3_) δ 18.9 (minor, *S*p), 18.6 (major, *R*p); IR (neat, cm^–1^) 2945, 1688, 1448, 1259, 1224, 1033, 996, 748, 709, 527, 452, 419; HRMS (ESI-QTOF) *m*/*z*: [M + Na]^+^ Calcd for C_31_H_34_ClN_4_O_5_PNa^+^, 631.1848; Found, 631.1849.

#### *S*p-T-Chloridate Monomer (*S*p)-**13**

After stirring for 10 min, the mixture was diluted with CH_2_Cl_2_ (100 mL) and washed with a 1.0 M NaH_2_PO_4_ aqueous solution (100 mL). The aqueous layer was extracted with CH_2_Cl_2,_ and the organic layers were combined. The organic layer was washed with brine (100 mL), and the aqueous layer was extracted with CH_2_Cl_2_. Then, the collected organic layers were combined, dried over Na_2_SO_4_, filtered, and concentrated under reduced pressure. CH_2_Cl_2_ (5.0 mL) was added to the residue. The insoluble residue was removed via suction filtration and washed with CH_2_Cl_2_. Subsequently, the filtrate was concentrated under reduced pressure. The crude product was purified by automated silica gel column chromatography (diol-silica gel, 37g, L size) using hexane–EtOAc (90:10–50:50, v/v) as an eluent to afford (*S*p)-**13** (colorless foam, 93.1 mg, 0.15 mmol, 51% yield, dr = 95:5).

^1^H NMR (500 MHz, CDCl_3_) δ 8.57 (s, 1H, H-3), 7.5–7.4 (br, 6H, Ar), 7.31 (t, *J* = 7.6 Hz, 6H, Ar), 7.20 (t, *J* = 6.9 Hz, 3H, Ar), 7.04 (d, *J* = 1.2 Hz, 1H, H-6), 6.14 (dd, *J* = 9.6, 2.4 Hz, 1H, H-1′), 4.43–4.38 (m, 1H, H-4′), 4.15–4.05 (m, 2H, H-5′, H-5″), 3.38 (dt, *J* = 11.3, 2.5 Hz, 1H, H-2′), 3.16 (dt, *J* = 11.8, 2.3 Hz, 1H, H-3′), 2.66 (s, 3H, −N­(CH_3_)_2_), 2.63 (s, 3H, −N­(CH_3_)_2_), 1.83 (d, *J* = 1.0 Hz, 3H, 5-CH_3_), 1.48 (dd, *J* = 11.5, 10.8 Hz, 1H, H-3″), 1.40 (dd, *J* = 11.3, 9.7 Hz, 1H, H-2″); ^13^C­{^1^H} NMR (126 MHz, CDCl_3_) δ 163.3 (C-4), 149.7 (C-2), 135.3 (C-6), 129.1, 127.9, 126.6 (Ar), 110.6 (C-5), 80.5 (C-1′), 74.7 (d, ^3^ *J*_PC_ = 8.5 Hz, C-4′), 67.1 (d, ^2^ *J*_PC_ = 5.6 Hz, C-5′), 51.8 (C-2′), 48.9 (C-3′), 36.6 (d, ^2^ *J*_PC_ = 2.9 Hz, −N­(CH_3_)_2_) 12.4 (5-CH_3_); ^31^P­{^1^H} NMR (202 MHz, CDCl_3_) δ 18.9 (major, *S*p), 18.6 (minor, *R*p); IR (neat, cm^–1^) 2945, 1690, 1448, 1265, 1033, 999, 748, 711, 527, 424; HRMS (ESI-QTOF) *m*/*z*: [M + Na]^+^ Calcd for C_31_H_34_ClN_4_O_5_PNa^+^, 631.1848; Found, 631.1847.

#### General Procedure for Synthesis of Dimethylamino Phosphorochloridate Monomers ((*R*p)-**16**, **17**, (*S*p)-**16**, **17**)

The 5′-oxazaphospholidine derivative (*R*p)-**9** (0.18 g, 0.22 mmol), (*R*p)-**10** (0.18 g, 0.20 mmol), (*S*p)-**9** (0.19 g, 0.22 mmol), or (*S*p)-**10** (0.19 g, 0.22 mmol) was dissolved in CH_2_Cl_2_ (2.0 mL for (*R*p)-**16** or (*R*p)-**17** or 2.2 mL for (*S*p)-**16** or (*S*p)-**17**) dried over MS 4A for 1 d (solution A). In another vessel, a mixture of 4-nitrophenol (42 mg, 0.30 mmol for (*R*p)-**16**, 41 mg, 0.30 mmol for (*R*p)-**17**, 56 mg, 0.40 mmol for (*S*p)-**16**, 42 mg, 0.30 mmol for (*S*p)-**17**) and CMPT (0.16 g, 0.60 mmol for (*R*p)-**16**, 0.16 g, 0.60 mmol for (*R*p)-**17**, 0.21 g, 0.79 mmol for (*S*p)-**16**, 0.16 g, 0.60 mmol for (*S*p)-**17**) in MeCN (4.0 mL for (*S*p)-**16**, 3.0 mL for (*R*p)-**16**, (*R*p)-**17**, or (*S*p)-**17**) was dried over MS 3A for 1 d (solution B). Solution A (2.0 mL for (*R*p)-**16** or (*R*p)-**17** or 2.2 mL for (*S*p)-**16**, or (*S*p)-**17**) was transferred to a round-bottom flask and cooled to 0 °C. 3,4-dichlorophenyl isocyanate (0.18 g, 0.97 mmol for (*R*p)-**16**, 0.19 g, 1.0 mmol for (*R*p)-**17** or (*S*p)-**17**, 0.18 g, 0.97 mmol for (*S*p)-**16**), and solution B (2.0 mL for (*R*p)-**16,** (*R*p)-**17**, (*S*p)-**16**, or (*S*p)-**17**) were added to the mixture. After stirring at 0 °C for 1 h for (*R*p)-**16**, **17**, or (*S*p)-**16**, or at 0 °C for 1.5 h for (*S*p)-**17** at 0 °C for 1 h, the mixture was cooled to −20 °C. TMSNMe_2_ (0.16 mL, 1.0 mmol for (*R*p)-**16,** (*R*p)-**17**, (*S*p)-**16**, or (*S*p)-**17**) and a 2.0 M THF solution of HNMe_2_ (1.0 mL, 2.0 mmol for (*R*p)-**16,** (*R*p)-**17**, (*S*p)-**16**, or (*S*p)-**17**) were added to the reaction mixture successively and stirred at −20 °C for 1 h. Then, the volatiles were removed under reduced pressure at 0 °C. The residue was dissolved in CH_2_Cl_2_ (4.0 mL for (*R*p)-**16,** (*R*p)-**17**, (*S*p)-**16**, or (*S*p)-**17**) and cooled to 0 °C. Subsequently, NCS (84 mg, 0.63 mmol for (*R*p)-**16**, 75 mg, 0.56 mmol for (*R*p)-**17**, 87 mg, 0.65 mmol for (*S*p)-**16**, 81 mg, 0.60 mmol for (*S*p)-**17**)) was added to the mixture.

#### *R*p-A-Chloridate Monomer (*R*p)-**16**

After stirring for 10 min, the mixture was diluted with CH_2_Cl_2_ (50 mL) and washed with 1.0 M NaH_2_PO_4_ aqueous solutions (50 mL) and brine (50 mL). The collected organic layer was dried over Na_2_SO_4_, filtered, and concentrated under reduced pressure. CH_2_Cl_2_ (2.0 mL) was added to the residue, and the insoluble residue was removed via suction filtration and washed with CH_2_Cl_2_ (4.0 mL). Subsequently, the filtrate was concentrated under reduced pressure. The crude product was purified by automated silica gel column chromatography (diol-silica gel, 37 g, L size) using hexane–EtOAc (70:30–20:80, v/v) as an eluent to afford (*R*p)-**16** (colorless foam, 68 mg, 94 μmol, 47% yield, dr = 4:96).

^1^H NMR (500 MHz, CDCl_3_) δ 8.86 (s, 1H, −CONH−), 8.81 (s, 1H, H-2), 8.01 (s, 1H, H-8), 8.00 (d, *J* = 7.4 Hz, 2H, Ar), 7.60 (tt, *J* = 7.4, 1.4 Hz, 1H, Ar), 7.53–7.45 (m, 8H, Ar), 7.32 (t, *J* = 7.7 Hz, 6H, Ar), 7.21 (t, *J* = 7.6 Hz, 3H, Ar), 6.42 (dd, *J* = 9.9, 2.4 Hz, 1H, H-1′), 4.55–4.50 (m, 1H, H-4′), 4.16–4.13 (m, 2H, H-5′, H-5″), 3.55 (dt, *J* = 11.4, 2.3 Hz, 1H, H-2′), 3.26 (dt, *J* = 11.9, 2.4 Hz, 1H, H-3′), 2.65 (s, 3H, −N­(CH_3_)_2_), 2.62 (s, 3H, −N­(CH_3_)_2_), 1.83 (dd, *J* = 11.3, 10.1 Hz, 1H, H-2″), 1.63 (dd, *J* = 11.7, 10.4 Hz, 1H, H-3″); ^13^C­{^1^H} NMR (126 MHz, CDCl_3_) δ 164.4 (−NHCO−),152.8 (C-2), 151.3 (C-4), 149.3 (C-6), 140.5 (C-8), 133.5, 132.8, 129.1, 128.9, 128.0, 127.8, 126.7 (Ar), 122.7 (C-5), 80.2 (C-1′), 74.7 (d, ^3^ *J*_PC_ = 8.4 Hz, C-4′), 67.1 (d, ^2^ *J*_PC_ = 5.5 Hz, C-5′), 53.0 (C-2′), 48.9 (C-3′), 36.6 (d, ^2^ *J*_PC_ = 2.8 Hz, −N­(CH_3_)_2_); ^31^P­{^1^H} NMR (202 MHz, CDCl_3_) δ 19.1 (minor, *S*p), 18.7 (major, *R*p); IR (neat, cm^–1^) 2919, 1691, 1581, 1448, 1247, 1027, 998, 745, 707, 547, 532, 453; HRMS (ESI-QTOF) *m*/*z*: [M + H]^+^ Calcd for C_38_H_38_ClN_7_O_4_P^+^, 722.2406.; Found, 722.2400.

#### *S*p-A-Chloridate Monomer (*S*p)-**16**

After stirring for 10 min, the mixture was diluted with CH_2_Cl_2_ (50 mL) and washed with a 1.0 M NaH_2_PO_4_ aqueous solution (50 mL) and brine (50 mL). The collected organic layer was dried over Na_2_SO_4_, filtered, and concentrated under reduced pressure. CH_2_Cl_2_ (2.0 mL) was added to the residue, and the insoluble residue was removed via suction filtration and washed with CH_2_Cl_2_ (4.0 mL). Subsequently, the filtrate was concentrated under reduced pressure. The crude product was purified by automated silica gel column chromatography (diol-silica gel, 37 g, L size) using hexane–EtOAc (70:30–20:80, v/v) as an eluent to afford (*S*p)-**16** (colorless foam, 46 mg, 64 μmol, 32% yield, dr = 96:4).

^1^H NMR (500 MHz, CDCl_3_) δ 9.00 (s, 1H, −CONH−), 8.80 (s, 1H, H-2), 8.01–7.99 (m, 3H, Ar, H-8), 7.59 (tt, *J* = 7.4, 1.3 Hz, 1H, Ar), 7.52–7.44 (m, 8H, Ar), 7.32 (t, *J* = 7.7 Hz, 6H, Ar), 7.21 (t, *J* = 7.5 Hz, 3H, Ar), 6.42 (dd, *J* = 9.9, 2.3 Hz, 1H, H-1′), 4.55–4.50 (m, 1H, H-4′), 4.19–4.09 (m, 2H, H-5′, H-5″), 3.54 (dt, *J* = 11.4, 2.3 Hz, 1H, H-2′), 3.26 (dt, *J* = 11.9, 2.2 Hz, 1H, H-3′), 2.63 (s, 3H, −N­(CH_3_)_2_), 2.60 (s, 3H, −N­(CH_3_)_2_), 1.82 (dd, *J* = 11.2, 9.6 Hz, 1H, H-2″), 1.63 (dd, *J* = 11.6, 10.2 Hz, 1H, H-3″); ^13^C­{^1^H} NMR (126 MHz, CDCl_3_) δ 164.5 (−NHCO−),152.8 (C-2), 151.2 (C-4), 149.4 (C-6), 140.5 (C-8), 133.5, 132.8, 129.1, 128.8, 128.0, 127.8, 126.7 (Ar), 122.7 (C-5), 80.2 (C-1′), 74.7 (d, ^3^ *J*_PC_ = 8.8 Hz, C-4′), 67.0 (d, ^2^ *J*_PC_ = 5.8 Hz, C-5′), 53.0 (C-2′), 48.9 (C-3′), 36.6 (d, ^2^ *J*_PC_ = 3.0 Hz, −N­(CH_3_)_2_); ^31^P­{^1^H} NMR (202 MHz, CDCl_3_) δ 19.1 (major, *S*p), 18.7 (minor, *R*p); IR (neat, cm^–1^) 2901, 1695, 1581, 1448, 1247, 1025, 998, 744, 710, 646, 525, 457, 420; HRMS (ESI-QTOF) *m*/*z*: [M + H]^+^ Calcd for C_38_H_38_ClN_7_O_4_P^+^, 722.2406.; Found, 722.2408.

#### *R*p-G-Chloridate Monomer (*R*p)-**17**

After stirring for 10 min, the mixture was diluted with CH_2_Cl_2_ (50 mL) and washed with a 1.0 M NaH_2_PO_4_ aqueous solution (50 mL). The aqueous layer was extracted with CH_2_Cl_2_ (50 mL), and the organic layers were combined. The organic layer was washed with brine (100 mL), and the aqueous layer was extracted with CH_2_Cl_2_ (100 mL). Then, the collected organic layers were combined, dried over Na_2_SO_4_, filtered, and concentrated under reduced pressure. CH_2_Cl_2_ (2.0 mL) was added to the residue. The insoluble residue was removed via suction filtration and washed with CH_2_Cl_2_ (4.0 mL). Subsequently, the filtrate was concentrated under reduced pressure. The crude product was purified by automated silica gel column chromatography (diol-silica gel, 37 g, L size) using hexane–EtOAc (70:30–30:70, v/v) as an eluent to afford (*R*p)-**17** (colorless foam, 58 mg, 77 μmol, 39% yield, dr = 5:95).

^1^H NMR (500 MHz, CDCl_3_) δ 7.82 (s, 1H, H-8), 7.79 (s, 1H, −CONH−), 7.5–7.4 (s, 6H, Ar), 7.31 (t, *J* = 7.7 Hz, 6H, Ar), 7.20 (t, *J* = 7.2 Hz, 3H, Ar), 6.24 (dd, *J* = 9.8, 2.4 Hz, 1H, H-1′), 4.82–4.73 (m, 2H, −OCH_2_CH_2_CN), 4.51–4.47 (m, 1H, H-4′), 4.14–4.11 (m, 2H, H-5′, H-5″), 3.50 (dt, *J* = 11.4, 2.3 Hz, 1H, H-2′), 3.23 (dt, *J* = 11.9, 2.2 Hz, 1H, H-3′), 3.05–2.95 (m, 3H, −CH­(CH_3_)_2_, −CH_2_CH_2_CN), 2.65 (s, 3H, −N­(CH_3_)_2_), 2.62 (s, 3H, −N­(CH_3_)_2_), 1.77 (dd, *J* = 11.2, 9.7 Hz, 1H, H-2″), 1.59 (dd, *J* = 11.6, 10.7 Hz, 1H, H-3″), 1.36 (t, *J* = 4.3 Hz, 6H, −CH­(CH_3_)_2_); ^13^C­{^1^H} NMR (126 MHz, CDCl_3_) δ 175.4 (−NHCO−),159.6 (C-6), 152.5 (C-4), 151.8 (C-2), 139.5 (C-8), 129.1, 128.0, 126.7, (Ar), 117.5 (C-5), 116.8 (−CH_2_ CN), 80.4 (C-1′), 74.6 (d, ^3^ *J*_PC_ = 8.6 Hz, C-4′), 67.1 (d, ^2^ *J*_PC_ = 5.5 Hz, C-5′), 61.6 (−OCH_2_CH_2_CN), 52.8 (C-2′), 48.9 (C-3′), 36.6 (d, ^2^ *J*_PC_ = 3.2 Hz, −N­(CH_3_)_2_), 36.0 (−CH­(CH_3_)_2_), 19.4 (−CH­(CH_3_)_2_), 19.3 (−CH­(CH_3_)_2_), 18.1 (−OCH_2_ CH_2_CN); ^31^P­{^1^H} NMR (202 MHz, CDCl_3_) δ 19.2 (minor, *S*p), 18.8 (major, *R*p); IR (neat, cm^–1^) 2962, 1721, 1593, 1384, 1242, 1027, 996, 747, 711, 637, 529, 420; HRMS (ESI-QTOF) *m*/*z*: [M + H]^+^ Calcd for C_38_H_43_ClN_8_O_5_P^+^, 757.2778.; Found, 757.2776.

#### *S*p-G-Chloridate Monomer (*S*p)-**17**

After stirring for 10 min, the mixture was diluted with CH_2_Cl_2_ (50 mL) and washed with a 1.0 M NaH_2_PO_4_ aqueous solution (50 mL). The aqueous layer was extracted with CH_2_Cl_2_ (50 mL)_,_ and the organic layers were combined. The organic layer was washed with brine (100 mL), and the aqueous layer was extracted with CH_2_Cl_2_ (100 mL). Then, the collected organic layers were combined, dried over Na_2_SO_4_, filtered, and concentrated under reduced pressure. CH_2_Cl_2_ (2.0 mL) was added to the residue. The insoluble residue was removed via suction filtration and washed with CH_2_Cl_2_ (4.0 mL). Subsequently, the filtrate was concentrated under reduced pressure. The crude product was purified by automated silica gel column chromatography (diol-silica gel, 37 g, L size) using hexane–EtOAc (70:30–30:70, v/v) as an eluent to afford (*S*p)-**17** (colorless foam, 47 mg, 62 μmol, 31% yield, dr = 93:7).

^1^H NMR (500 MHz, CDCl_3_) δ 7.86 (s, 1H, −CONH−), 7.81 (s, 1H, H-8), 7.5–7.4 (s, 6H, Ar), 7.31 (t, *J* = 7.6 Hz, 6H, Ar), 7.20 (t, *J* = 7.3 Hz, 3H, Ar), 6.25 (dd, *J* = 9.9, 2.3 Hz, 1H, H-1′), 4.82–4.72 (m, 2H, −OCH_2_CH_2_CN), 4.52–4.47 (m, 1H, H-4′), 4.17–4.05 (m, 2H, H-5′, H-5″), 3.50 (dt, *J* = 11.2, 2.4 Hz, 1H, H-2′), 3.24 (dt, *J* = 11.9, 2.3 Hz, 1H, H-3′), 3.06–2.96 (m, 3H, −CH­(CH_3_)_2_, −OCH_2_CH_2_CN−), 2.63 (s, 3H, −N­(CH_3_)_2_), 2.60 (s, 3H, −N­(CH_3_)_2_), 1.76 (dd, *J* = 10.8, 9.5 Hz, 1H, H-2″), 1.59 (dd, *J* = 11.3, 10.9 Hz, 1H, H-3″), 1.35 (t, *J* = 6.7 Hz, 6H, −CH­(CH_3_)_2_); ^13^C­{^1^H} NMR (126 MHz, CDCl_3_) δ 175.4 (−NHCO−), 159.6 (C-6), 152.5 (C-4), 151.8 (C-2), 139.5 (C-8), 129.1, 128.0, 127.9, 127.9, 127.2, 126.7, (Ar), 117.5 (C-5), 116.8 (−CH_2_ CN), 80.4 (C-1′), 74.6 (d, ^3^ *J*_PC_ = 8.4 Hz, C-4′), 67.1 (d, ^2^ *J*_PC_ = 5.6 Hz, C-5′), 61.6 (−OCH_2_CH_2_CN), 52.9 (C-2′), 49.0 (C-3′), 36.6 (d, ^2^ *J*_PC_ = 2.7 Hz, −N­(CH_3_)_2_), 36.0 (−CH­(CH_3_)_2_), 19.4 (−CH­(CH_3_)_2_), 19.4 (−CH­(CH_3_)_2_), 18.1 (−CH_2_ CH_2_CN); ^31^P­{^1^H} NMR (202 MHz, CDCl_3_) δ 19.2 (major, *S*p), 18.8 (minor, *R*p); IR (neat, cm^–1^) 2967, 1715, 1609, 1385, 1241, 996, 746, 709, 638, 532, 442; HRMS (ESI-QTOF) *m*/*z*: [M + H]^+^ Calcd for C_38_H_43_ClN_8_O_5_P^+^, 757.2778.; Found, 757.2776.

#### Large-Scale Synthesis of (*R*p)-**16**

The 5′-oxazaphospholidine derivative (*R*p)-**9** (0.83 g, 1.0 mmol) was dissolved in CH_2_Cl_2_ (10 mL), dried over MS 4A for 1 d (solution A). In another vessel, a mixture of 4-nitrophenol (0.15 g, 1.09 mmol) and CMPT (0.58 g, 2.2 mmol) in MeCN (11 mL) was dried over MS 3A for 1 d (solution B). Solution A (10 mL) was transferred to a round-bottom flask, and cooled to 0 °C. Subsequently, 3,4-dichlorophenyl isocyanate (0.94 g, 5.0 mmol) and solution B (10 mL) were added to the mixture. After stirring at 0 °C for 1 h, the mixture was cooled to −20 °C. TMSNMe_2_ (0.80 mL, 5.0 mmol) and a 2.0 M THF solution of Me_2_NH (5.0 mL, 10 mmol) were added to the reaction mixture successively and stirred at −20 °C for 1 h. Then, the volatiles were removed under reduced pressure at 0 °C. The residue was dissolved in CH_2_Cl_2_ (20 mL) and cooled to 0 °C. Subsequently, NCS (0.41 g, 3.1 mmol) was added to the mixture. After stirring for 10 min, the mixture was diluted using CH_2_Cl_2_ (100 mL) and washed with a 1.0 M NaH_2_PO_4_ aqueous solution (100 mL). The aqueous layer was extracted with CH_2_Cl_2_ (100 mL)_,_ and the organic layers were combined. The organic layer was washed with brine (100 mL), and the aqueous layer was extracted with CH_2_Cl_2_ (100 mL). Subsequently, the collected organic layers were combined, dried over Na_2_SO_4_, filtered, and concentrated under reduced pressure. CH_2_Cl_2_ (5.0 mL) was added to the residue. The insoluble residue was removed by suction filtration and washed with CH_2_Cl_2_ (25 mL). Subsequently, the filtrate was concentrated under reduced pressure. The crude product was purified by automated silica gel column chromatography (diol-silica gel, 37 g, L size) using hexane–EtOAc (70:30–20:80, v/v) as an eluent to afford (*R*p)-**16** (colorless foam, 0.38 g, 0.52 mmol, 52%, yield, dr = 5:95).

#### General Procedure for Synthesis of Dimethylamino Phosphorochloridate Monomers ((*R*p)-**18** or (*S*p)-**18**)

The 5′-oxazaphospholidine derivative (*R*p)-**8** or (*S*p)-**8** (0.18 g, 0.22 mmol) was dissolved in CH_2_Cl_2_ (2.0 mL) and dried over MS 4A for 1 d (solution C). In another vessel, a mixture of 4-nitrophenol (42 mg, 0.31 mmol for (*R*p)-**8**; 56 mg, 0.41 mmol for (*S*p)-**8**) and CMPT (0.16 g, 0.61 mmol for (*R*p)-**8**; 0.21 g, 0.79 mmol for (*S*p)-**8**) in MeCN (3.0 mL for (*R*p)-**8**; 4.0 mL for (*S*p)-**8**) was dried over MS 3A for 1 d (solution D). Solution C (2.0 mL) was transferred to a round-bottom flask and cooled to 0 °C. Subsequently, 3,4-dichlorophenyl isocyanate (0.19 g, 1.0 mmol for (*R*p)-**8**; 0.20 g, 1.1 mmol for (*S*p)-**8**) was added, and solution D (2.0 mL) was added to the solution. After stirring at 0 °C for 1 h, the mixture was cooled to −20 °C. Dimethylketene methyl trimethylsilyl acetal (0.12 mL, 0.59 mmol) and a 2.0 M THF solution of Me_2_NH (2.0 mL, 4.0 mmol) were added to the reaction mixture and stirred at −20 °C for 1 h. Subsequently, the volatiles were removed under reduced pressure at 0 °C. The residue was dissolved in CH_2_Cl_2_ (4.0 mL) and cooled to 0 °C. Subsequently, NCS (84 mg, 0.63 mmol for (*R*p)-**8**; 75 mg, 0.56 mmol for (*S*p)-**8**) was added to the solution.

#### *R*p-C-Chloridate Monomer (*R*p)-**18**

After stirring for 10 min, the mixture was diluted with CH_2_Cl_2_ (50 mL) and washed with a 1.0 M NaH_2_PO_4_ aqueous solution (50 mL). The aqueous layer was extracted with CH_2_Cl_2_ (50 mL)_,_ and the organic layers were combined. The organic layer was washed with brine (100 mL), and the aqueous layer was extracted with CH_2_Cl_2_ (100 mL). Then, the collected organic layers were combined, dried over Na_2_SO_4_, filtered, and concentrated under reduced pressure. CH_2_Cl_2_ (2.0 mL) was added to the residue. The insoluble residue was removed via suction filtration and washed with CH_2_Cl_2_ (4.0 mL). Subsequently, the filtrate was concentrated under reduced pressure. The crude product was purified by automated silica gel column chromatography twice (diol-silica gel, 37 g, L size) using hexane–EtOAc (70:30–20:80, v/v) as an eluent, for the first time, (diol-silica gel, 37 g, L size) using hexane–EtOAc (50:50–20:80, v/v) as an eluent, for the second time to afford (*R*p)-**18** (yellow foam, 66 mg, 95 μmol, 47% yield, dr = 3:97).

^1^H NMR (500 MHz, CDCl_3_) δ 8.6–8.5 (br, 1H, −CONH−), 7.87 (d, *J* = 6.7 Hz, 2H, Ar), 7.73 (d, *J* = 7.3 Hz, 1H, H-6), 7.60 (tt, *J* = 7.4, 1.1 Hz, 1H, Ar), 7.52–7.40 (m, 9H, Ar, H-5), 7.30 (t, *J* = 7.5 Hz, 6H, Ar), 7.19 (t, *J* = 6.9 Hz, 3H, Ar), 6.28 (dd, *J* = 9.2, 2.1 Hz, 1H, H-1′), 4.46–4.41 (m, 1H, H-4′), 4.20–4.10 (m, 2H, H-5′, H-5″), 3.60 (dt, *J* = 11.3, 2.2 Hz, 1H, H-2′), 3.17 (dt, *J* = 11.9, 2.3 Hz, 1H, H-3′), 2.66 (s, 3H, −N­(CH_3_)_2_), 2.63 (s, 3H, −N­(CH_3_)_2_), 1.54 (dd, *J* = 11.5, 10.6 Hz, 1H, H-3″), 1.29 (dd, *J* = 11.2, 9.4 Hz, 1H, H-2″); ^13^C­{^1^H} NMR (126 MHz, CDCl_3_) δ 162.1 (C-4), 154.2 (C-2), 144.6 (C-6), 133.2, 129.0, 127.9, 127.5, 126.6, 125.8 (Ar), 96.3 (C-5), 82.0 (C-1′), 74.7 (d, ^3^ *J*_PC_ = 8.3 Hz, C-4′), 67.2 (d, ^2^ *J*_PC_ = 5.5 Hz, C-5′), 52.6 (C-2′), 48.8 (C-3′), 36.6 (d, ^2^ *J*_PC_ = 2.8 Hz, −N­(CH_3_)_2_); ^31^P­{^1^H} NMR (202 MHz, CDCl_3_) δ 19.1 (minor, *S*p), 18.7 (major, *R*p); IR (neat, cm^–1^) 2953, 1668, 1552, 1479, 1348, 1306, 1256, 1031, 995, 745, 705, 523, 445; HRMS (ESI-QTOF) *m*/*z*: [M + H]^+^ Calcd for C_37_H_38_ClN_5_O_5_P^+^, 698.2294.; Found, 698.2294.

#### *S*p-C-Chloridate Monomer (*S*p)-**18**

After stirring for 10 min, the mixture was diluted with CH_2_Cl_2_ (50 mL) and washed with a 1.0 M NaH_2_PO_4_ aqueous solution (50 mL) and brine (50 mL). The aqueous layers were combined and extracted with CH_2_Cl_2_ (100 mL), and the collected organic layers were combined, dried over Na_2_SO_4_, filtered, and concentrated under reduced pressure. CH_2_Cl_2_ (2.0 mL) was added to the residue. The insoluble residue was removed via suction filtration and washed with CH_2_Cl_2_ (4.0 mL). Then, the filtrate was concentrated under reduced pressure. The crude product was purified by automated silica gel column chromatography (diol-silica gel, 37 g, L size) using hexane–EtOAc (70:30–20:80, v/v) as an eluent to afford (*S*p)-**18** (colorless foam, 65 mg, 93 μmol, 46% yield, 97:3).

^1^H NMR (500 MHz, CDCl_3_) δ 8.9–8.6 (br, 1H, −CONH−), 7.88 (d, *J* = 7.5 Hz, 2H, Ar), 7.72 (d, *J* = 7.4 Hz, 1H, H-6), 7.59 (tt, *J* = 7.4, 2.0 Hz, 1H, Ar), 7.51–7.41 (m, 8H, Ar, H-5), 7.30 (t, *J* = 7.6 Hz, 6H, Ar), 7.19 (t, *J* = 7.2 Hz, 3H, Ar), 6.28 (dd, *J* = 9.2, 2.1 Hz, 1H, H-1′), 4.46–4.42 (m, 1H, H-4′), 4.18–4.08 (m, 2H, H-5′, H-5″), 3.60 (dt, *J* = 11.3, 2.5 Hz, 1H, H-2′), 3.17 (dt, *J* = 11.9, 2.4 Hz, 1H, H-3′), 2.66 (s, 3H, −N­(CH_3_)_2_), 2.63 (s, 3H, −N­(CH_3_)_2_), 1.52 (dd, *J* = 11.5, 10.8 Hz, 1H, H-3″), 1.28 (dd, *J* = 11.2, 9.4 Hz, 1H, H-2″); ^13^C­{^1^H} NMR (126 MHz, CDCl_3_) δ 162.1 (C-4), 154.1 (C-2), 144.4 (C-6), 133.2, 132.9, 129.1, 129.0, 127.9, 127.6, 126.6 (Ar), 96.6 (C-5), 82.0 (C-1′), 74.8 (d, ^3^ *J*_PC_ = 8.5 Hz, C-4′), 67.2 (d, ^2^ *J*_PC_ = 5.6 Hz, C-5′), 52.6 (C-2′), 48.9 (C-3′), 36.6 (d, ^2^ *J*_PC_ = 2.8 Hz, −N­(CH_3_)_2_); ^31^P­{^1^H} NMR (202 MHz, CDCl_3_) δ 19.1 (major, *S*p), 18.7 (minor, *R*p); IR (neat, cm^–1^) 2936, 1665, 1480, 1305, 1256, 1022, 996, 702, 526, 453, 421; HRMS (ESI-QTOF) *m*/*z*: [M + H]^+^ Calcd for C_37_H_38_ClN_5_O_5_P^+^, 698.2294.; Found, 698.2293.

#### Investigation of Dimethylamination Conditions (Table 2)

The 5′-oxazaphospholidine derivative (*R*p)-**7** (0.14 g, 0.20 mmol for entries 1, 5, and 6; 0.11 g, 0.15 mmol, for entry 2; 0.22 g, 0.30 mmol, for entry 3; 0.19 g, 0.26 mmol for entry 4) was dissolved in MeCN (2.0 mL, for entries 1, 5, and 6; 1.5 mL for entry 2; 3.0 mL for entry 3; 2.5 mL for entry 4) with MS 3A for 1 d, and the mixture (1.5 mL for entries 1–3; 5 and 6; 1.0 mL for entry 4) was transferred to a bottom flask and cooled to 0 °C. PhNCO (80 μL, 0.75 mmol for entries 1–3; 5, and 6; 55 μL, 0.51 mmol for entry 4) was added. A mixture of 4-nitrophenol (29 mg, 0.21 mmol for entries 1 and 4; 28 mg, 0.20 mmol for entries 2, 5, and 6; 56 mg, 0.41 mmol for entry 3) and CMPT (0.10 g, 0.40 mmol for entries 1, 4, 5, and 6; 0.11 g, 0.41 mmol, for entry 2; 0.21 g, 0.80 mmol for entry 3) in MeCN (2.0 mL for entries 1, 2, 4, 5, and 6; 4.0 mL for entries 3) were dried over MS 3A for 1 d, and the mixture (1.5 mL for entries 1–3, 5, and 6; 1.0 mL for entry 4) was added to the solution. After stirring for 40 min for entries 1–4 or 20 min for entries 5 and 6 at 0 °C, dimethylamination was conducted as shown below. For entry 1, a 2.0 M THF solution of HNMe_2_ (0.75 mL, 1.5 mmol) was added to the reaction mixture and stirred at RT for 30 min. For entry 2, a 2.0 M THF solution of HNMe_2_ (0.75 mL, 1.5 mmol) was added to the reaction mixture and stirred at 0 °C for 30 min. For entry 3, TMSNMe_2_ (0.12 mL, 0.75 mmol) and a 2.0 M THF solution of HNMe_2_ (0.75 mL, 1.5 mmol) were added to the reaction mixture and stirred at 0 °C for 1 h. For entry 4, the mixture (2.0 mL) was cooled to −20 °C. TMSNMe_2_ (80 μL, 0.50 mmol) and 2.0 M THF solution of HNMe_2_ (0.50 mL, 1.0 mmol) were added to the reaction mixture and stirred at −20 °C for 1 h. For entry 5, the mixture (2.0 mL) was cooled to −40 °C. TMSNMe_2_ (80 μL, 0.50 mmol) and a 2.0 M THF solution of HNMe_2_ (0.50 mL, 1.0 mmol) were added to the reaction mixture and stirred at −40 °C for 1 h. For entry 6, the mixture (2.5 mL) was cooled to −20 °C. BSA (61 μL, 0.25 mmol) and a 2.0 M THF solution of HNMe_2_ (0.63 mL, 1.3 mmol) were added to the reaction mixture and stirred at −20 °C for 1 h. Subsequently, the solvent and the volatile residue were removed under reduced pressure at 0 °C. The reaction mixture was dissolved in CH_2_Cl_2_ (2.5 mL for entries 1, 5, and 6; 3.0 mL for entries 2 and 3; 2.0 mL for entry 4) and the mixture (2.0 mL for entries 1, 4, 5, and 6; 3.0 mL for entries 2 and 3) was cooled to 0 °C and NCS (41 mg, 0.30 mmol for entry 1; 63 mg, 0.47 mmol for entry 2; 60 mg, 0.45 mmol for entry 3; 40 mg, 0.30 mmol for entry 4 and 6; 42 mg, 0.31 mmol for entry 5) was added to the solution. After stirring for 10 min at 0 °C, the formation of **13** was monitored by ^31^P NMR analysis.

#### Investigation of the Phenol Derivatives (Table 3)

The 5′-oxazaphospholidine derivative (*R*p)-**7** (0.11 g, 0.15 mmol, for entries 1 and 6; 0.25 g, 0.35 mmol, for entries 2, 3 and 5; 0.19 g, 0.26 mmol for entry 4) was dissolved in MeCN (1.5 mL, for entries 2, 3 and 5; 3.5 mL, for entries 1 and 6; 0.19 g, 2.5 mL for entry 4) with MS 3A for 1 d; the mixture (1.5 mL, for entries 1–3, 5, and 6; 1.0 mL for entry 4) was transferred to a round-bottom flask and cooled to 0 °C. PhNCO (80 μL, 0.75 mmol for entries 1–3, 5, and 6; 55 μL, 0.51 mmol for entry 4) was added. A mixture of phenol (4-cyanophenol (24.4 mg, 0.21 mmol) for entry 1; 2-methyl-6-nitrophenol (31 mg, 0.21 mmol) for entry 2; 2-nitrophenol (28 mg, 0.20 mmol) for entry 3; 4-nitrophenol (29 mg, 0.21 mmol) for entry 4; 2,4,6-trifluorophenol (31 mg, 0.21 mmol) for entry 5; 2,4,6-trichlorophenol (39 mg, 0.20 mmol) for entry 6) was added. CMPT (0.10 g, 0.40 mmol) in MeCN (4.0 mL) was dried over MS 3A for 1 d, and the mixture (1.5 mL for entries 1–3, 5, and 6; 1.0 mL for entry 4) was added to the solution. After stirring for 40 min at 0 °C, the mixture (3.0 mL, for entries 1 and 6; 2.5 mL, for entries 2, 3, and 5; 2.0 mL for entry 4) was cooled to −20 °C. TMSNMe_2_ (0.12 mL, 0.75 mmol for entries 1 and 6; 0.10 mL, 0.62 mmol for entries 2, 3, and 5; 80 μL, 0.50 mmol for entry 4) and a 2.0 M THF solution of Me_2_NH (0.75 mL, 1.5 mmol, for entries 1 and 6; 0.63 mL, 1.3 mmol, for entries 2, 3, and 5; 0.50 mL, 1.0 mmol for entry 4) were added to the reaction mixture and stirred at −20 °C for 1 h. Subsequently, the solvent and the volatile residue were removed under reduced pressure at 0 °C. The reaction mixture was dissolved in CH_2_Cl_2_ (3.0 mL for entries 1 and 6; 2.5 mL for entries 2, 3, and 5; 2.0 mL for entry 4). The mixture (3.0 mL for entries 1 and 6; 2.0 mL for entries 2, 3, 4, and 5) was cooled to 0 °C, and NCS (59 mg, 0.44 mmol for entry 1; 43 mg, 0.32 mmol for entry 2; 50 mg, 0.37 mmol for entry 3; 40 mg, 0.30 mmol for entry 4 and 5; 61 mg, 0.46 mmol for entry 6) was added to the solution. After stirring for 10 min at 0 °C, the formation of **13** was monitored by ^31^P NMR analysis.

#### Investigation of PhNCO Derivative

The 5′-oxazaphospholidine derivative (*R*p)-**7** (0.11 g, 0.15 mmol for entry 1; 0.18 g, 0.25 mmol for entry 2 and 3; 0.18 g, 0.25 mmol for entry 4) was dissolved in CH_2_Cl_2_ (1.5 mL for entry 1; 2.5 mL for entries 2–4) with MS 4A for 1 d. The mixture (1.0 mL) was transferred to a round-bottom flask and cooled to 0 °C. PhNCO derivative (phenyl isocyanate (11 μL, 0.10 mol) for entry 1, 4-nitrophenylisocyanate (17 mg, 0.10 mmol) for entry 2, 2,4,6-trichlorophenylisocyanate (23 mg, 0.10 mmol) for entry 3, and 3,4-dichlorophenylisocyanate (19 mg, 0.10 mmol) for entry 4) was added. A mixture of 4-nitrophenol (20 mg, 0.14 mmol, for entry 1; 35 mg, 0.25 mmol for entries 2 and 3; 42 mg, 0.30 mmol for entry 4) and CMPT (78 mg, 0.30 mmol for entry 1; 0.13 g, 0.50 mmol for entry 2 and 3; 0.16 g, 0.60 mmol for entry 4) in MeCN (1.5 mL for entry 1; 2.5 mL for entries 2 and 3; 3.0 mL for entry 4) was dried over MS 3A for 1 d, and the mixture (1.0 mL) was added to the solution. After stirring at 0 °C for 20, 40, and 60 min, the mixture was monitored by ^31^P NMR analysis at RT, respectively.

### Synthesis of Phosphorodiamidate Morpholino Dimers

#### T_PN_T Dimer ((*S*p)-**23** and (*R*p)-**23**)

A chloridate monomer (*R*p)-**13** (34 mg, 55 μmol, dr = 10:90) or (*S*p)-**13** (67 mg, 110 μmol, dr = 98:2) was dissolved in MeCN (1.0 mL for (*S*p)-**23** or 2.0 mL for (*R*p)-**23**), and a 1.0 M MeCN solution of *N*-ethylmorpholine (14 μL, 0.11 mmol for (*S*p)-**23** or 28 μL, 0.22 mmol for (*R*p)-**23**) was added to the solution. Then, a morpholino thymidine derivative **19** (53 mg, 0.11 mmol for (*S*p)-**23** or 48 mg, 0.10 mmol for (*R*p)-**23**) bearing a free amino group was added to the solution at RT, and the mixture was stirred for 1 h (for (*S*p)-**23**) or 2 h (for (*R*p)-**23**) at RT. Then, the mixture was diluted with EtOAc (20 mL) and washed with brine (3 × 20 mL). The organic layer was dried over Na_2_SO_4_, filtered, and concentrated under reduced pressure.

#### *S*p-T_PN_T Dimer (*S*p)-**23**

The residue was purified by automated silica gel chromatography twice (neutral silica gel, 16 g, M size) using CHCl_3_–MeOH (98:2–91:9, v/v) as an eluent, for the first time, (neutral silica gel, 16 g, M size) using CHCl_3_–MeOH (98:2–91:9, v/v) as an eluent, for the second time to afford (*S*p)**-23** (colorless foam, 52 mg, 49 μmol, 89%, dr = 91:9).

^1^H NMR (500 MHz, CDCl_3_) δ 8.14 (s, 1H, Th-H-3 (5′-upstream)), 8.06 (s, 1H, Th-H-3 (3′-downstream)), 7.67–7.63 (m, 4H, Ar), 7.5–7.4 (m, 7H, Ar), 7.39–7.36 (m, 4H, Ar), 7.29–7.26 (m, 7H, Ar), 7.19–7.15 (m, 4H, Th-H-6 (5′-upstream), Ar), 7.04 (d, *J* = 1.2 Hz, 1H, Th-H-6 (3′-downstream)), 6.15 (dd, *J* = 9.6, 2.3 Hz, 1H, Th-H-1′ (3′-downstream)), 5.60 (dd, *J* = 10.0, 2.7 Hz, 1H, Th-H-1′ (5′-upstream)), 4.41–4.36 (m, 1H, Th-H-4′ (3′-downstream)), 3.96–3.80 (m, 3H, Th-H-5′ (3′-downstream), Th-H-5″ (3′-downstream), Th-H-4′ (5′-upstream)), 3.70 (d, *J* = 4.4 Hz, 2H, Th-H-5′ (5′-upstream), Th-H-5″ (5′-upstream)), 3.41–3.34 (m, 3H, Th-H-2′ (3′-downstream), Th-H-2′ (5′-upstream), Th-H-3′ (5′-upstream)), 3.14 (dt, *J* = 11.8, 2.4 Hz, 1H, Th-H-3′ (3′-downstream)), 2.84–2.78 (m, 1H, Th-H-3″ (5′-upstream)), 2.65–2.55 (m, 7H, N­(CH_3_)_2_, Th-H-2″ (5′-upstream)), 1.88 (d, *J* = 1.1 Hz, 3H, Me (5′-upstream)), 1.81 (d, *J* = 1.0 Hz, 3H, Me (3′-downstream)), 1.45–1.40 (m, 2H, Th-H-2″ (3′-downstream)), Th-H-3′′ (3′-downstream)), 1.07 (s, 9H, C­(CH_3_)_3_); ^13^C­{^1^H} NMR (126 MHz, CDCl_3_) δ 163.8 (Th-C-4 (3′-downstream)), 163.6 (Th-C-4 (5′-upstream)), 149.9 (Th-C-2 (3′-downstream)), 149.7 (Th-C-2 (5′-upstream)), 135.5, 135.4, (Th-C-6 (3′-downstream)), 135.0 (Th-C-6 (5′-upstream)), 132.8, 132.8, 129.9, 129.9, 127.8, 127.7, 126.6 (Ar), 110.9 (Th-C-5 (5′-upstream)), 110.6 (Th-C-5 (3′-downstream), 80.4 (Th-C-1′ (3′-downsteam)), 79.6 (d, ^3^ *J*_PC_ = 8.7 Hz, Th-C-1′ (5′-upstream)), 77.7 (d, ^3^ *J*_PC_ = 7.1 Hz, Th-C-4′ (5′-upstream)), 75.2 (d, ^3^ *J*_PC_ = 6.7 Hz, (3′-downstream)), 65.3 (d, ^2^ *J*_PC_ = 3.7 Hz, Th-C-5′ (3′-downstream)), 64.0 (Th-C-5′ (5′-upstream)), 51.7 (Th-C-2′ (3′-downstream)), 49.1 (Th-C-3′ (3′-downstream)), 47.2 (Th-C-2′ (5′-upstream)), 45.1 (Th-C-3′ (5′-upstream)), 36.7, 36.6 (N­(CH_3_)_2_), 26.7 (C­(CH_3_)_3_), 19.2 (C­(CH_3_)_3_), 12.5 (Th-C-5-CH_3_ (5′-upstream)), 12.3 (Th-C-5-CH_3_ (3′-downstream)); ^3^ ^1^P­{^1^H} NMR (202 MHz, CDCl_3_) δ 17.1 (major, *S*p), 16.8 (minor, *R*p); HRMS (ESI/QTOF) *m*/*z*: [M + H]^+^ Calcd for C_55_H_67_N_7_O_9_PSi^+^, 1052.4502; Found, 1052.4506.

#### *R*p-T_PN_T Dimer (*R*p)-**23**

The residue was purified by automated silica gel chromatography (neutral silica gel, 16 g, M size) using CHCl_3_–MeOH (98:2–91:9, v/v) as an eluent to afford (*R*p)-**23** (colorless foam, 88 mg, 84 μmol, 83%, dr = 2:98).

^1^H NMR (500 MHz, CDCl_3_) δ 8.20 (s, 1H, Th-H-3 (5′-upstream)), 8.11 (s, 1H, Th-H-3 (3′-downstream)), 7.65–7.61 (m, 4H, Ar), 7.51–7.40 (m, 6H, Ar), 7.39–7.36 (m, 4H, Ar), 7.29–7.26 (m, 8H, Ar), 7.16–7.13 (m, 4H, Th-H-6 (5′-upstream), Ar), 7.06 (d, *J* = 1.2 Hz, 1H, Th-H-6 (3′-downstream)), 6.12 (dd, *J* = 9.6, 2.3 Hz, 1H, Th-H-1′ (3′-downstream)), 5.55 (dd, *J* = 9.8, 2.7 Hz, 1H, Th-H-1′ (5′-upstream)), 4.37–4.33 (m, 1H, Th-H-4′ (3′-downstream)), 3.97–3.85 (m, 2H, Th-H-5′, Th-H-5″ (3′-downstream)), 3.81–3.76 (m, 1H, Th-H-4′ (5′-upstream)), 3.73–3.65 (m, 2H, Th-H-5′, Th-H-5′′ (5′-upstream)), 3.40–3.28 (m, 3H, Th-H-2′ (3′-downstream), Th-H-2′ (5′-upstream), Th-H-3′ (5′-upstream)), 3.14 (dt, *J* = 11.8, 2.4 Hz, Th-H-3′ (3′-downstream)), 2.75–2.70 (m, 1H, Th-H-3″ (5′-upstream)), 2.64 (d, *J* = 9.6 Hz, 6H, N­(CH_3_)_2_), 2.54–2.49 (m, 1H, Th-H-2″ (5′-upstream)), 1.90 (d, *J* = 1.1 Hz, 3H, Me (5′-upstream)), 1.79 (d, *J* = 1.1 Hz, 3H, Me (3′-downstream)), 1.48–1.42 (m, 2H, Th-H-2″ (3′-downstream), Th-H-3′′ (3′-downstream)), 1.05 (s, 9H, C­(CH_3_)_3_); ^13^C­{^1^H} NMR (126 MHz, CDCl_3_) δ 163.7 (Th-C-4 (5′-upstream)), 163.6 (Th-C-4 (3′-downstream)), 149.9 (Th-C-2 (5′-upstream)), 149.8 (Th-C-2 (3′-downstream)), 135.5 (Th-C-6 (3′-downstream)), 135.5, 135.4, 135.0 (Th-C-6 (5′-upstream)), 132.9, 132.8, 129.9, 127.9, 127.8, 127.7, 126.5 (Ar), 110.8 (Th-C-5 (5′-upstream)), 110.6 (Th-C-5 (3′-downstream), 80.3 (Th-C-1′ (3′-downsteam)), 79.7 (d, *J* = 7.7 Hz, Th-C-1′ (5′-upsteam)), 77.7 (d, ^3^ *J*_PC_ = 5.5 Hz, Th-C-4′ (5′-upstream)), 75.4 (d, ^3^ *J*_PC_ = 6.4 Hz, Th-C-4′ (3′-downstream)), 65.5 (d, ^2^ *J*_PC_ = 4.3 Hz, Th-C-5′ (3′-downstream)), 64.1 (Th-C-5′ (5′-upstream)), 51.9 (Th-C-2′ (3′-downstream)), 49.0 (Th-C-3′ (3′-downstream)), 47.3 (Th-C-2′ (5′-upstream)), 45.1 (Th-C-3′ (5′-upstream)), 36.6, 36.6 (N­(CH_3_)_2_), 26.7 (C­(CH_3_)_3_), 19.3 (C­(CH_3_)_3_), 12.5 (Th-C-5-CH_3_ (5′-upstream)), 12.4 (Th-C-5-CH_3_ (3′-downstream)); ^3^ ^1^P­{^1^H} NMR (202 MHz, CDCl_3_) δ 17.1 (minor, *S*p), 16.8 (major, *R*p); HRMS (ESI/QTOF) *m*/*z*: [M + Na]^+^ Calcd for C_55_H_66_N_7_O_9_PSiNa^+^, 1074.4322; Found, 1074.4338.

#### A_PN_T Dimer ((*S*p)-**24** and (*R*p)-**24**)

A chloridate monomers (*R*p)-**13** (67 mg, 0.11 mmol, dr = 3:97 for (*S*p)-**24**) or (*S*p)-**13** (67 mg, 0.11 μmol, dr = 98:2 for (*R*p)-**24**) and a morpholino adenosine derivative **20** (59 mg, 0.10 mmol for (*S*p)-**24**; 60 mg, 0.10 mmol for (*R*p)-**24**) bearing a free amino group were dissolved in THF (1.0 mL), and (*i*Pr)_2_NEt (33 μL, 0.22 mmol) was added to the mixture at RT. The mixture was stirred for 17 h at RT, and the mixture was diluted with EtOAc (20 mL) and washed with brine (3 × 20 mL). The aqueous layers were combined and extracted with EtOAc (50 mL). The organic layers were combined, dried over Na_2_SO_4_, filtered, and concentrated under reduced pressure.

#### *S*p-A_PN_T dimer (*S*p)-**24**

The residue was purified by automated silica gel chromatography (neutral silica gel, 16 g, M size) using CHCl_3_–MeOH (98:2–91:9) as an eluent to afford (*S*p)-**24** (colorless foam, 68 mg, 58 μmol, 58%, dr = 94:6).

^1^H NMR (500 MHz, CDCl_3_) δ 9.07 (s, 1H, −NHCO−), 8.80 (s, 1H, Ad-H-2), 8.21 (s, 1H, Th-H-3), 8.08 (s, 1H, Ad-H-8), 8.06–8.05 (m, 2H, Ar), 7.63 (tt, *J* = 7.6, 1.2 Hz, 5H, Ar), 7.54 (t, *J* = 7.6 Hz, 2H, Ar), 7.50–7.40 (m, 7H, Ar), 7.35 (td, *J* = 7.3, 1.4 Hz, 4H, Ar), 7.27–7.24 (m, 7H, Ar), 7.14 (t, *J* = 6.7 Hz, 3H, Ar), 7.01 (d, *J* = 1.2 Hz, 1H, Th-H-6), 6.13 (dd, *J* = 9.6, 2.2 Hz, 1H, Th-H-1′), 5.86 (dd, *J* = 10.0, 2.6 Hz, 1H, Ad-H-1′), 4.42–4.37 (m, 1H, Th-H-4′), 4.00–3.91 (m, 3H, Th-C-5′, Th-H-5″, Ad-H-4′), 3.78–3.65 (m, 3H, Ad-H-5′, Ad-H-2′), 3.49–3.44 (m, 1H, Ad-H-3′), 3.34 (dt, *J* = 11.3, 2.2 Hz, 1H, Th-H-2′), 3.15 (dd, *J* = 11.8, 2.3 Hz, 1H, Th-H-3′), 3.10–3.04 (ddd, *J* = 16.5, 10.2, 6.4 Hz, 1H, Ad-H-2″), 2.91–2.84 (m, 1H, Ad-H-3′′), 2.65 (d, *J* = 9.8 Hz, 6H, −N­(CH_3_)_2_), 1.76 (d, *J* = 0.9 Hz, 3H, Th-H-5-CH_3_), 1.48–1.40 (m, 2H, Th-H-2″, Th-H-3′′), 1.05 (s, 9H, C­(CH_3_)_3_); ^13^C­{^1^H} NMR (126 MHz, CDCl_3_) δ 164.6 (−NHCO−), 163.0 (Th-C-4), 152.8 (Ad-C-2), 151.2 (Ad-C-4), 149.6 (Ad-C-6), 149.5 (Th-C-2), 140.5 (Ad-C-8), 135.5, 135.5, 135.3 (Th-C-6), 133.5, 132.9, 132.8, 129.9, 129.9, 128.9, 127.9, 127.8, 126.6 (Ar), 122.8 (Ad-C-5), 110.5 (Th-C-5), 80.4 (Th-C-1′), 80.0 (d, ^3^ *J*_PC_ = 7.4 Hz, Ad-C-1′), 77.6 (d, ^3^ *J*_PC_ = 5.1 Hz, Ad-C-4′), 75.4 (d, ^3^ *J*_PC_ = 7.0 Hz, Th-C-4′), 65.5 (d, ^2^ *J*_PC_ = 4.1 Hz, Th-C-5′), 64.1 (Ad-C-5′), 51.8 (Th-C-2′), 49.0 (Th-C-3′), 48.3 (Ad-C-2′), 45.4 (Ad-C-3′), 36.7, 36.7 (−N­(CH_3_)_2_), 26.8 (C­(CH_3_)_3_), 19.3 (−C­(CH_3_)_3_), 12.4 (Th-C-5-CH_3_); ^31^P­{^1^H} NMR (202 MHz, CDCl_3_) δ 17.1 (major, *S*p), 17.0 (minor, *R*p); HRMS (ESI-QTOF) *m*/*z*: [M + H]^+^ Calcd for C_64_H_70_N_10_O_8_PSi^+^, 1165.4880; Found, 1165.4878.

#### *R*p-A_PN_T Dimer (*R*p)-**24**

The residue was purified by automated silica gel chromatography (neutral silica gel, 16 g, M size) using CHCl_3_–MeOH (98:2–91:9) as an eluent to afford (*R*p)-**24** (colorless foam, 105 mg, 90 μmol, 89%, dr = 2:98).

^1^H NMR (500 MHz, CDCl_3_) δ 9.18 (s, 1H, −NHCO−), 8.75 (s, 1H, Ad-H-2), 8.47 (s, 1H, Th-H-3), 8.09–8.07 (m, 3H, Ar, Ad-H-8), 7.61 (tt, *J* = 6.3, 1.6 Hz, 5H, Ar), 7.54 (t, *J* = 7.6 Hz, 2H, Ar), 7.50–7.38 (m, 7H, Ar), 7.35 (dt, *J* = 7.0, 1.2 Hz, 5H, Ar), 7.23 (t, *J* = 7.7 Hz, 6H), 7.11 (t, *J* = 7.2 Hz, 3H, Ar), 7.00 (d, *J* = 1.1 Hz, 1H, Th-H-6), 6.08 (dd, *J* = 9.6, 2.2 Hz, 1H, Th-H-1′), 5.83 (dd, *J* = 10.0, 2.6 Hz, 1H, Ad-H-1′), 4.38–4.34 (m, 1H, Th-H-4′), 4.00–3.95 (m, 1H, Th-H-5′), 3.93–3.88 (m, 3H, Th-H-5′, Ad-H-4′), 3.75 (dd, *J* = 11.0, 4.6 Hz, 1H, Ad-H-5′), 3.70–3.65 (m, 2H, Ad-H-5′, Ad-H-2′), 3.39 (dd, *J* = 12.3, 8.1 Hz, 1H, Ad-H-3′), 3.30 (dt, *J* = 11.3, 8.1 Hz, 1H, Th-H-2′), 3.12 (dt, *J* = 11.8, 2.0 Hz, 1H, Th-H-3′), 3.05–3.00 (m, 1H, Ad-H-2′), 2.87–2.81 (m, 1H, Ad-H-3′), 2.68 (d, *J* = 9.7 Hz, 6H, −N­(CH_3_)_2_), 1.71 (d, *J* = 0.8 Hz, 3H, Th-H-5-CH_3_), 1.47–1.39 (m, 2H, Th-H-2′, Th-H-3′), 1.04 (s, 9H, C­(CH_3_)_3_); ^13^C­{^1^H} NMR (126 MHz, CDCl_3_) δ 164.7 (−NHCO−), 163.0 (Th-C-4), 152.7 (Ad-C-2), 151.2 (Ad-C-4), 149.7 (Ad-C-6), 149.5 (Th-C-2), 140.3 (Ad-C-8), 135.5, 135.5, 135.1 (Th-C-6), 133.5, 132.9, 132.8, 129.9, 129.9, 128.8, 128.0, 127.9, 127.8, 127.8, 126.5 (Ar), 122.8 (Ad-C-5), 110.6 (Th-C-5), 80.4 (Th-C-1′), 79.7 (d, ^3^ *J*_PC_ = 8.3 Hz, Ad-C-1′), 77.6 (d, ^3^ *J*_PC_ = 8.3 Hz, Ad-C-4′), 75.3 (d, ^3^ *J*_PC_ = 8.3 Hz, Th-C-4′), 65.5 (d, ^2^ *J*_PC_ = 4.6 Hz, Th-C-5′), 64.0 (Ad-C-5′), 51.7 (Th-C-2′), 49.0 (Th-C-3′), 48.3 (Ad-C-2′), 45.4 (Ad-C-3′), 36.7, 36.7 (−N­(CH_3_)_2_), 26.8 (C­(CH_3_)_3_), 19.2 (−C­(CH_3_)_3_), 12.3 (Th-C-5-CH_3_); ^31^P­{^1^H} NMR (202 MHz, CDCl_3_) δ 17.1 (minor, *S*p), 17.0 (major, *R*p); HRMS (ESI-QTOF) *m*/*z*: [M + H]^+^ Calcd for C_64_H_70_N_10_O_8_PSi^+^, 1165.4880; Found, 1165.4882.

#### C_PN_T Dimer ((*S*p)-**25** and (*R*p)-**25**)

A chloridate monomers (*R*p)-**13** (67 mg, 0.11 mmol, dr = 3:97 for (*S*p)-**25**) or (*S*p)-**13** (67 mg, 0.11 mmol, dr = 97:3 for (*R*p)-**25**) and a morpholino cytidine derivative **21** (58 mg, 0.10 mmol for (*S*p)-**25**; 59 mg, 0.10 mmol for (*R*p)-**25**) bearing a free amino group were dissolved in THF (1.0 mL), and (*i*Pr)_2_NEt (33 μL, 0.22 mmol) was added to the mixture at RT. The mixture was stirred for 4 h at RT, and the mixture was diluted with EtOAc (10 mL) and washed with brine (3 × 10 mL). The aqueous layers were combined and extracted with EtOAc (30 mL). The organic layers were combined, dried over Na_2_SO_4_, filtered, and concentrated under reduced pressure.

#### *S*p-C_PN_T Dimer (*S*p)-**25**

The residue was purified by automated silica gel chromatography (neutral silica gel, 16 g, M size) using CHCl_3_–MeOH (98:2–91:9) as an eluent to afford (*S*p)-**25** (colorless foam, 88 mg, 77 μmol, 75%, dr = 95:5).

^1^H NMR (500 MHz, CDCl_3_) δ 8.89 (s, 1H, −NHCO−), 8.72 (s, 1H, Th-H-3), 7.93 (d, *J* = 7.6 Hz, 2H, Ar), 7.72 (d, *J* = 7.5 Hz, 1H, Cy-H-6), 7.68–7.64 (m, 4H, Ar), 7.61 (dt, *J* = 7.4, 1.6 Hz, 1H, Ar), 7.54–7.37 (m, 14H, Cy-H-5, Ar), 7.29–7.26 (m, 6H, Ar), 7.15 (t, *J* = 6.9 Hz, 3H, Ar), 7.08 (d, *J* = 1.2 Hz, 1H, Th-H-6), 6.14 (dd, *J* = 9.5, 2.2 Hz, 1H, Th-H-1′), 5.66 (dd, *J* = 9.4, 2.5 Hz, 1H, Cy-H-1′), 4.43–4.38 (m, 1H, Th-H-4′), 4.01–3.96 (m, 1H, Th-H-5′), 3.93–3.89 (m, 1H, Th-H-5″), 3.86–3.82 (m, 1H, Cy-H-4′), 3.74 (d, *J* = 4.4 Hz, 2H, Cy-H-5′, Cy-H-5″), 3.62 (t, *J* = 10.1 Hz, Cy-H-2′), 3.44 (dd, *J* = 12.1, 7.5 Hz, 1H, Cy-H-3′), 3.37 (dt, *J* = 11.3, 2.2 Hz, 1H, Th-H-2′), 3.15 (dt, *J* = 11.8, 2.1 Hz, 1H, Th-H-3′), 2.87–2.81 (m, 1H, Cy-H-3″), 2.62 (d, *J* = 9.8 Hz, 6H, −N­(CH_3_)_2_), 2.44–2.39 (m, 1H, Cy-H-2′), 1.81 (d, *J* = 1.0 Hz, 3H, Th-H-5-CH_3_), 1.48–1.39 (m, 2H, Th-H-2″, Th-H-3′′), 1.07 (s, 9H, C­(CH_3_)_3_); ^13^C­{^1^H} NMR (126 MHz, CDCl_3_) δ 166.4 (−NHCO−), 163.5 (Th-C-4), 162.3 (Cy-C-4), 154.0 (Cy-C-2), 149.7 (Th-C-2), 144.0 (Cy-C-6), 135.5 (Th-C-6), 135.5, 133.2, 133.0, 132.9, 129.9, 129.0, 127.9, 127.8, 127.8, 127.6, 126.5, 110.4 (Th-C-5), 96.5 (Cy-C-5), 81.5 (d, ^3^ *J*_PC_ = 7.6 Hz, Cy-C-1′), 80.5 (Th-C-1′), 77.8 (d, ^3^ *J*_PC_ = 5.8 Hz, Cy-C-4′), 76.8 (C­(Ph)_3_), 75.3 (d, ^3^ *J*_PC_ = 6.7 Hz, Th-C-4′), 65.4 (d, ^2^ *J*_PC_ = 4.4 Hz, Th-C-5′), 64.2 (Cy-C-5′), 51.9 (Th-C-2′), 49.1 (Th-C-3′), 47.9 (Cy-C-2′), 45.1 (Cy-C-3′), 36.6, 36.6 (−N­(CH_3_)_2_), 26.7 (C­(CH_3_)_3_), 19.2 (−C­(CH_3_)_3_), 12.4 (Th-C-5-CH_3_); ^31^P­{^1^H} NMR (202 MHz, CDCl_3_) δ 17.2 (major, *S*p), 16.7 (minor, *R*p); HRMS (ESI-QTOF) *m*/*z*: [M + H]^+^ Calcd for C_63_H_70_N_8_O_9_PSi^+^,1141.4768; Found, 1141.4768.

#### *R*p-C_PN_T Dimer (*R*p)-**25**

The residue was purified by automated silica gel chromatography (neutral silica gel, 16 g, M size) using CHCl_3_–MeOH (98:2–91:9) as an eluent to afford (*R*p)-**25** (colorless foam, 87 mg, 76 μmol, 74%, dr = 4:96).

^1^H NMR (500 MHz, CDCl_3_) δ 9.05 (s, 1H, −NHCO−),8.86 (s, 1H, Th-H-3), 7.98 (d, *J* = 7.6 Hz, 2H, Ar), 7.70 (d, *J* = 7.5 Hz, 1H, Cy-H-6), 7.66–7.63 (m, 4H, Ar), 7.60 (dt, *J* = 7.4, 1.9 Hz, 1H, Ar), 7.52 (t, *J* = 7.6 Hz, 3H, Cy-H-5, Ar), 7.47–7.37 (m, 11 H, Ar, Cy-H-5), 7.28–7.25 (m, 6H, Ar), 7.13 (t, *J* = 7.1 Hz, 3H, Ar), 7.08 (d, *J* = 1.2 Hz, 1H, Th-H-6), 6.15 (dd, *J* = 9.6, 2.2 Hz, 1H, Th-H-1′), 5.64 (dd, *J* = 9.4, 2.5 Hz, 1H, Cy-H-1′), 4.38–4.33 (m, 1H, Th-H-4′), 3.96–3.91 (m, 1H, Th-H-5′), 3.89–3.81 (m, 2H, Th-H-5″, Cy-H-4′), 3.78–3.70 (m, 2H, Cy-H-5′, Cy-H-5′′), 3.61 (t, *J* = 9.1 Hz, Cy-H-2′), 3.38–3.32 (m, 2H, Cy-H-3′, Th-H-2′), 3.10 (dt, *J* = 11.8, 2.1 Hz, 1H, Th-H-3′), 2.75–2.70 (m, 1H, Cy-H-3″), 2.67 (d, *J* = 9.6 Hz, 6H, −N­(CH_3_)_2_), 2.36–2.31 (m, 1H, Cy-H-2″), 1.76 (d, *J* = 1.0 Hz, 3H, Th-H-5-CH_3_), 1.47–1.42 (m, 2H, Th-H-2″, Th-H-3′′), 1.06 (s, 9H, C­(CH_3_)_3_); ^13^C­{^1^H} NMR (126 MHz, CDCl_3_) δ 166.4 (−NHCO−), 163.7 (Th-C-4), 162.3 (Cy-C-4), 153.9 (Cy-C-2), 149.7 (Th-C-2), 143.9 (Cy-C-6), 135.6 (Th-C-6), 135.5, 135.5, 133.2, 132.9, 132.8, 129.9, 129.9, 129.1, 128.9, 127.8, 127.8, 127.8, 127.7, 126.4 (Ar), 110.5 (Th-C-5), 96.6 (Cy-C-5), 81.3 (d, ^3^ *J*_PC_ = 9.1 Hz, Cy-C-1′), 80.4 (Th-C-1′), 77.9 (d, ^3^ *J*_PC_ = 7.6 Hz, Cy-C-4′), 75.1 (d, ^3^ *J*_PC_ = 7.2 Hz, Th-C-4′), 65.3 (d, ^2^ *J*_PC_ = 3.7 Hz, Th-C-5′), 64.1 (Cy-C-5′), 51.7 (Th-C-2′), 49.0 (Th-C-3′), 47.8 (Cy-C-2′), 45.1 (Cy-C-3′), 36.7, 36.6 (−N­(CH_3_)_2_), 26.7 (C­(CH_3_)_3_), 19.2 (−C­(CH_3_)_3_), 12.3 (Th-C-5-CH_3_); ^31^P­{^1^H} NMR (202 MHz, CDCl_3_) δ 17.2 (minor, *S*p), 16.7 (major, *R*p); HRMS (ESI-QTOF) *m*/*z*: [M + H]^+^ Calcd for C_63_H_70_N_8_O_9_PSi^+^, 1141.4768; Found, 1141.4774.

#### G_PN_T Dimer ((*S*p)-**26** and (*R*p)-**26**)

A chloridate monomers (*R*p)-**13** (52 mg, 86 μmol, dr = 3:97 for (*S*p)-**26**) or (*S*p)-**13** (69 mg, 0.11 mmol, dr = 97:3 for (*R*p)-**26**) and a morpholino guanosine derivative **22** (44 mg, 77 μmol for (*S*p)-**26**, 58 mg, 0.10 mmol for (*R*p)-**26**) bearing a free amino group were dissolved in THF (1.0 mL), and (*i*Pr)_2_NEt (21 μL, 0.14 mmol for (*S*p)-**26**, 33 μL, 0.22 mmol for (*R*p)-**26**) was added to the mixture at RT. The mixture was stirred for 4 h at RT, and the mixture was diluted with EtOAc (10 mL) and washed with brine (3 × 10 mL). The aqueous layers were combined and extracted with EtOAc (30 mL). The organic layers were combined, dried over Na_2_SO_4_, filtered, and concentrated under reduced pressure.

#### *S*p-G_PN_T Dimer (*S*p)-**26**

The residue was purified by automated silica gel chromatography (neutral silica gel, 16 g, M size) using CHCl_3_–MeOH (98:2–91:9) as an eluent to afford (*S*p)-**26** (colorless foam, 75 mg, 66 μmol, 86%, dr = 96:4).

^1^H NMR (500 MHz, CDCl_3_) δ 12.0 (s, 1H, Gu-H-1), 8.59 (s, 1H, −NHCO), 8.00 (s, 1H, Th-H-3), 7.69 (s, 1H, Gu-H-8), 7.64–7.61 (m, 4H, Ar), 7.47–7.39 (m, 7H, Ar), 7.36 (td, *J* = 7.1, 1.2 Hz, 5H, Ar), 7.28–7.25 (m, 6H, Ar), 7.16 (t, *J* = 6.4 Hz, 3H, Ar), 6.99 (d, *J* = 1.2 Hz, 1H, Th-H-6), 6.12 (dd, *J* = 9.8, 2.5 Hz, 1H, Th-H-1′), 5.53 (dd, *J* = 10.1, 2.6 Hz, 1H, Gu-H-1′), 4.37–4.32 (m, 1H, Th-H-4′), 3.95–3.83 (m, 3H, Th-H-5′, Gu-H-4′), 3.80–3.66 (m, 2H, Gu-H-5′, Gu-H-2′), 3.36–3.30 (m, 2H, Th-H-2′, Gu-H-3′), 3.13 (dt, *J* = 11.9, 2.3 Hz, 1H, Th-H-3′), 2.94–2.88 (m, 1H, Gu-H-2″), 2.78–2.71 (m, 1H, Gu-H-3′′), 2.65 (d, *J* = 9.4 Hz, 6H, −N­(CH_3_)_2_), 2.60–2.54 (m, 1H, −CH­(CH_3_)_2_), 1.81 (d, *J* = 1.0 Hz, 3H, Th-H-5-CH_3_), 1.45–1.37 (m, 2H, Th-H-2″, Th-H-3′′), 1.27–1.25 (m, 6H, CH­(CH_3_)_2_), 1.05 (s, 9H, −C­(CH_3_)_3_); ^13^C­{^1^H} NMR (126 MHz, CDCl_3_) δ 178.6 (−NHCO−), 163.4 (Th-C-4), 155.4 (Gu-C-6), 149.8 (Th-C-2), 147.6 (Gu-C-2), 147.4 (Gu-C-4), 136.1 (Gu-C-8), 135.5, 135.5, 135.3 (Th-C-6), 132.8, 130.0, 129.9, 127.9, 127.8, 126.6 (Ar), 121.2 (Gu-C-5), 110.7 (Th-C-5), 80.3 (Th-C-1′), 80.1 (d, ^3^ *J*_PC_ = 7.4 Hz, Gu-C-1′), 77.4 (Gu-C-4′) 75.4 (d, ^3^ *J*_PC_ = 7.4 Hz, Th-C-4′), 65.7 (^2^ *J*_PC_ = 4.6 Hz, Th-C-5′), 64.1 (Gu-C-5′), 51.7 (Th-C-2′), 49.0 (Th-C-3′), 48.1 (Gu-C-2′), 45.4 (Gu-C-3′), 36.7, 36.7 (−N­(CH_3_)_2_), 36.4 (−CH­(CH_3_)_2_), 26.8 (−C­(CH_3_)_3_), 19.2 (−C­(CH_3_)_3_), 19.0, 18.8 (CH­(CH_3_)_2_), 12.4 (Th-C-5-CH_3_); ^31^P­{^1^H} NMR (202 MHz, CDCl_3_) δ 17.2 (major, *S*p), 16.6 (minor, *R*p); HRMS (ESI-QTOF) *m*/*z*: [M + H]^+^ Calcd for C_61_H_72_N_10_O_9_PSi^+^, 1147.4986; Found, 1147.4956.

#### *R*p-G_PN_T Dimer (*R*p)-**26**

The residue was purified by automated silica gel chromatography (neutral silica gel, 16 g, M size) using CHCl_3_–MeOH (98:2–91:9) as an eluent to afford (*R*p)-**26** (colorless foam, 100 mg, 87 μmol, 87%, dr = 5:95).

^1^H NMR (500 MHz, CDCl_3_) δ 9.71 (s, 1H, −NHCO), 8.05 (s, 1H, Th-H-3), 7.67–7.64 (m, 5H,Gu-H-8, Ar), 7.47–7.43 (m, 7H, Ar, Th-H-6), 7.41–7.37 (m, 5H, Ar), 7.30–7.26 (m, 7H, Ar), 7.19 (t, *J* = 7.0 Hz, 3H, Ar), 6.13 (dd, *J* = 9.5, 2.3 Hz, 1H, Th-H-1′), 5.35 (dd, *J* = 10.1, 2.6 Hz, 1H, Gu-H-1′), 4.43–4.38 (m, 1H, Th-H-4′), 4.14–4.08 (m, 1H, Th-H-5′), 3.94 (td, *J* = 11.3, 3.8 Hz, 1H, Th-H-5″), 3.71 (dd, J = 10.3, 4.2 Hz, 1H, Gu-H-5′), 3.66–3.54 (m, 4H, Gu-H-4′, Gu-H-2′, Gu-H-5′, Gu-H-3′), 3.45 (dt, *J* = 11.3, 2.4 Hz, 1H, Th-H-2′), 3.12 (dt, *J* = 11.7, 2.5 Hz, 1H, Th-H-2″), 2.97–2.91 (m, 1H, Gu-H-2′′), 2.80–2.67 (m, 2H, Gu-H-3′′, −CH­(CH_3_)_2_), 2.58 (d, *J* = 10.4 Hz, 6H, −N­(CH_3_)_2_), 1.87 (d, *J* = 1.0 Hz, 3H, Th-H-5-CH_3_), 1.45–1.36 (m, 2H, Th-H-2′, Th-H-3′), 1.26 (dd, *J* = 6.9, 2.5 Hz, 6H, −CH­(CH_3_)_2_), 1.12 (s, 9H, −C­(CH_3_)_3_); ^13^C­{^1^H} NMR (126 MHz, CDCl_3_) δ 179.2 (−NHCO−), 164.1 (Th-C-4), 155.5 (Gu-C-6), 149.6 (Th-C-2), 148.0 (Gu-C-2), 147.6 (Gu-C-4), 136.2 (Gu-C-8), 135.7, 135.5 (Th-C-6), 132.7, 132.6, 129.9, 129.8, 127.9, 127.8, 127.7, 126.6 (Ar), 120.8 (Gu-C-5), 110.4 (Th-C-5), 80.6 (Th-C-1′), 79.6 (d, ^3^ *J*_PC_ = 5.9 Hz, Gu-C-1′), 77.2 (Gu-C-4′), 75.0 (d, ^3^ *J*_PC_ = 4.6 Hz, Th-C-4′), 66.0 (^2^ *J*_PC_ = 5.5 Hz, Th-C-5′), 64.0 (Gu-C-5′), 51.8 (Th-C-2′), 48.8 (Th-C-3′), 47.5 (Gu-C-2′), 46.2 (Gu-C-3′), 36.4, 36.4 (−N­(CH_3_)_2_), 36.1 (−CH­(CH_3_)_2_), 26.8 (−C­(CH_3_)_3_), 19.2 (−C­(CH_3_)_3_), 19.0, 18.8 (CH­(CH_3_)_2_), 12.4 (Th-C-5-CH_3_); ^31^P­{^1^H} NMR (202 MHz, CDCl_3_) δ 17.2 (minor, *S*p), 16.6 (major, *R*p); HRMS (ESI-QTOF) *m*/*z*: [M + H]^+^ Calcd for C_61_H_72_N_10_O_9_PSi^+^, 1147.4986; Found, 1147.4967.

#### General Procedure for the Removal of the Protecting Group of the T_PN_T Dimers

The T_PN_T dimer (*S*p)-**23** (21 mg, 20 μmol) or (*R*p)-**23** (21 mg, 20 μmol) was dissolved in CH_2_Cl_2_ (2.0 mL) (solution E). 3-Cyanopyridine (83 mg, 0.80 mmol for (*S*p)-**27** or 87 mg, 0.80 mmol for (*R*p)-**27**) and CF_3_COOH (61 μL, 0.80 mmol) were dissolved in a mixture of CH_2_Cl_2_–CF_3_CH_2_OH (3:2, v/v) (2.0 mL) (solution F). To solution E, solution F was added at RT and allowed to stir for 1 h. The mixture was concentrated under reduced pressure. Then, the residue was dissolved in THF (0.8 mL). A 1.0 M tetrabutylammonium fluoride (TBAF) THF solution (0.80 mL, 0.80 mmol) was added to the solution at RT, and the mixture was stirred for 2 h at RT.

#### *S*p-T_PN_T Dimer ((*S*p)-**27**)

The mixture was diluted with Et_2_O (10 mL) and extracted with H_2_O (10 mL). The aqueous layer was concentrated under reduced pressure. The crude T_PN_T dimer was purified by automated silica gel column chromatography (ODS, 7g, S size) using H_2_O–MeCN (100:0–70:30) as an eluent. Then, the residue was dissolved in 0.25 M KPF_6_ aqueous solutions (10 mL) and washed with CH_2_Cl_2_ (3 × 10 mL). The aqueous layers were concentrated under reduced pressure. The T_PN_T dimer was purified by automated silica gel column chromatography (ODS, 7g, S size) using H_2_O–MeCN (100:0–70:30) as an eluent to afford (*S*p)-**27** (3.3 mg, 5.8 μmol, 29%, dr = 95:5).

^1^H NMR (600 MHz, D_2_O) δ 7.63 (s, 1H, Th-H-6), 7.58 (s, 1H, Th-H-6), 5.75 (d, *J* = 9.8 Hz, 1H, Th-H-1′ (3′-downstream)), 5.70 (d, *J* = 9.5 Hz, 1H, Th-H-1′ (5′-upstream)), 4.22–4.20 (m, 1H, Th-H-5′ (3′-downstream)), 4.12–4.05 (m, 2H, Th-H-4′ (3′-downstream), Th-H-5″ (3′-downstream)), 3.97–3.92 (br, 1H, Th-H-4′ (5′-upstream)), 3.70–3.64 (m, 2H, Th-H-5′, Th-H-5′′ (5′-upstream)), 3.50 (t, *J* = 10.2 Hz, 1H, Th-H-2′ (5′-upstream)), 3.38 (t, *J* = 10.5 Hz, 1H, Th-H-3′ (5′-upstream)), 3.07–2.86 (m, 4H, Th-H-2″ (5′-upstream), Th-H-2′ (3′-downstream), Th-H-3′ (3′-downstream), Th-H-3′′ (5′-upstream)), 2.80–2.67 (m, 8H, Th-H-3′′ (3′-downstream), Th-H-2′′ (3′-downstream), −N­(CH_3_)_2_), 1.87 (s, 3H, Th-H-5-CH_3_, (3′-downstream)), 1.80 (s, 3H, Th-H-5-CH_3_ (5′-upstream)); ^13^C­{^1^H} NMR (126 MHz, D_2_O) δ 169.3, 169.2 (Th-C-4), 154.3, 154.1 (Th-C-2), 140.2 (Th-C-6), 114.3, 114.0 (Th-C-5), 82.9 (Th-C-1′ (3′-downstream)), 82.5 (d, ^3^ *J*_PC_ = 6.5 Hz, Th-C-1′ (5′-upstream)), 80.5 (d, ^3^ *J*_PC_ = 4.9 Hz, Th-C-4′ (5′-upstream)), 78.8 (d, ^3^ *J*_PC_ = 6.9 Hz, Th-C-4′ (3′-downstream), 68.7 (d, ^2^ *J*_PC_ = 4.6 Hz, Th-C-5′ (3′-downstream)), 64.4 (Th-C-5′ (5′-upstream)), 49.3 (Th-C-2′ (3′-downstream)), 48.7 (Th-C-2′ (5′-upstream)), 46.8 (Th-C-3′ (5′-upstream)), 46.4 (Th-C-3′ (3′-downstream)), 38.6, 38.6 (−N­(CH_3_)_2_), 14.4 (Th-C-5-CH_3_), 14.3 (Th-C-5-CH_3_); ^31^P­{^1^H} NMR (202 MHz, D_2_O) δ 19.7 (major, *S*p), 19.6 (minor, *R*p); HRMS (ESI-QTOF) *m*/*z*: [M + H]^+^ Calcd for C_22_H_35_N_7_O_9_P^+^, 572.2229; Found, 572.2217.

#### *R*p-T_PN_T Dimer ((*R*p)-**27**)

The mixture was diluted with Et_2_O (10 mL) and extracted with H_2_O (2 × 10 mL). 0.25 M KPF_6_ aqueous solution (10 mL) was added to the aqueous layer and washed with CH_2_Cl_2_ (3 × 30 mL). The aqueous layers were concentrated under reduced pressure. The crude T_PN_T dimer was purified by automated silica gel column chromatography twice (ODS, 14g, M size, H_2_O–MeCN (99:1–70:30)) as an eluent, for the first time, (ODS, 14g, M size, H_2_O–MeCN (100:0–70:30)) as an eluent to afford (*R*p)-**27** (5.6 mg, 9.8 μmol, 49%, dr >1:99).

^1^H NMR (500 MHz, D_2_O) δ 7.63–7.62 (m, 2H, Th-H-6 (3′-downstream), Th-H-6 (5′-upstream)), 5.71 (d, *J* = 9.9, 2.6 Hz, 1H, Th-H-1′ (3′-downstream)), 5.65 (d, *J* = 10.1, 2.6 Hz, 1H, Th-H-1′ (5′-upstream)), 4.14–4.08 (m 3H, Th-H-4′ (3′-downstream), Th-H-5′, Th-H-5″ (3′-downstream)), 3.95–3.90 (m, 1H, Th-H-4′ (5′-upstream)), 3.65 (dd, *J* = 12.2, 4.0 Hz, 1H, Th-H-5′ (5′-upstream)), 3.60 (dd, *J* = 12.2, 5.4 Hz, 1H, Th-H-5″ (5′-upstream)), 3.52 (t, *J* = 10.4 Hz, 1H, Th-H-2′ (5′-upstream)), 3.27–3.23 (m, Th-H-3′ (5′-upstream)) 3.01 (dd, *J* = 12.8, 2.6 Hz, 1H, Th-H-2′ (3′-downstream)), 2.90–2.74 (m, 4H, Th-H-3′ (3′-downstream), Th-H-2″ (5′-upstream), Th-H-2′′ (3′-downstream), Th-H-3′ (5′-upstream)), 2.69 (d, *J* = 10.1 Hz, 6H, −N­(CH_3_)_2_), 2.61 (dd, *J* = 13.0, 10.8 Hz, Th-H-3″ (3′-downstream)), 1.87 (s, 3H, Th-H-5-CH_3_)), 1.80 (s, 3H, Th-H-5-CH_3_); ^13^C­{^1^H} NMR (126 MHz, D_2_O) δ 169.7, 169.5 (Th-C-4), 154.5, 154.2 (Th-C-2), 140.2, 140.0 (Th-C-6), 114.2, 114.1 (Th-C-5), 82.9 (Th-C-1′ (3′-downstream)), 82.7 (d, ^3^ *J*_PC_ = 7.1 Hz, Th-C-1′ (5′-upstream)), 80.2 (d, ^3^ *J*_PC_ = 5.6 Hz, Th-C-4′ (5′-upstream)), 79.3 (d, ^3^ *J*_PC_ = 7.1 Hz, Th-C-4′ (3′-downstream), 69.2 (d, ^2^ *J*_PC_ = 5.3 Hz, Th-C-5′ (3′-downstream)), 64.4 (Th-C-5′ (5′-upstream)), 49.3 (Th-C-2′ (3′-downstream)), 49.0 (d, ^2^ *J*_PC_ = 1.8 Hz, Th-C-2′ (5′-uptream)), 46.8 (Th-C-3′ (5′-upstream)), 46.4 (Th-C-3′ (3′-downstream)), 38.6, 38.6 (−N­(CH_3_)_2_), 14.4 (Th-C-5-CH_3_), 14.3 (Th-C-5-CH_3_); ^31^P­{^1^H} NMR (202 MHz, D_2_O) δ 19.3; HRMS (ESI-QTOF) *m*/*z*: [M + H]^+^ Calcd for C_22_H_35_N_7_O_9_P^+^, 572.2229; Found, 572.2201.

#### General Procedure for the Removal of Protecting Groups of the A_PN_T Dimers

The A_PN_T dimer (*S*p)-**24** (35 mg, 30 μmol) or (*R*p)-**24** (35 mg, 30 μmol) was dissolved in CH_2_Cl_2_ (3.0 mL) (solution G). 3-Cyanopyridine (0.38 g, 3.6 mmol for (*S*p)-**28**, 0.12 g, 1.2 mmol for (*R*p)-**28**) and CF_3_COOH (0.28 mL, 3.7 mmol for (*S*p)-**28**, 0.10 mL, 1.3 mmol for (*R*p)-**28**) were dissolved in a mixture of CH_2_Cl_2_–CF_3_CH_2_OH (3:2, v/v) (9.0 mL for (*S*p)-**28**, 3.0 mL for (*R*p)-**28**) (solution H). To solution G, solution H (3.0 mL) was added at RT and stirred for 50 min (for (*S*p)-**28**) or 25 min (for (*R*p)-**28**). Then, the mixture was diluted with CH_2_Cl_2_ (20 mL) and washed with a saturated aqueous solution of NaHCO_3_ (3 × 20 mL). The aqueous layers were combined and extracted with CH_2_Cl_2_ (50 mL), and the collected organic layers were combined, dried over Na_2_SO_4_, filtered, and concentrated under reduced pressure. For the synthesis of (*S*p)-**28**, the residue was dissolved in THF (4.0 mL) (solution I). Et_3_N-3HF (0.98 mL, 6.0 mmol) and Et_3_N (1.7 mL, 12 mmol) were dissolved in THF (15 mL) (solution J). To solution I, solution J (7.5 mL) was added at RT and stirred for 20 h. For the synthesis of (*R*p)-**28**, the residue was dissolved in THF (6.0 mL). To the mixture, Et_3_N (0.83 mL, 6.0 mmol) and Et_3_N-3HF (0.49 mL, 3.0 mmol) were added and stirred for 16 h. The mixture was concentrated under reduced pressure. The residue was treated with concentrated aqueous NH_3_–EtOH (3:1, v/v, 8 mL) at 55 °C for 18 h for (*S*p)-**28** or 22 h for (*R*p)-**28** in an oil bath. The mixture was diluted with H_2_O (10 mL) and washed with Et_2_O (2 × 10 mL for (*S*p)-**28** or 2 × 20 mL for (*R*p)-**28**). The aqueous layer was concentrated under reduced pressure.

#### *S*p-A_PN_T Dimer ((*S*p)-**28**)

The crude A_PN_T dimer was purified by automated silica gel column chromatography (ODS, 7 g, S size) using H_2_O–MeCN (100:0–70:30) as an eluent and recycle RP-HPLC (ODS, H_2_O–MeCN (75:25, v/v)) to afford (*S*p)-**28** (4.4 mg, 7.6 μmol, 25%, dr = 97:3).

^1^H NMR (600 MHz, D_2_O) δ 8.28 (s, 1H, Ad-H-8), 8.12 (s, 1H, Ad-H-2), 7.25 (s, 1H, Th-H-6), 5.84 (d, *J* = 8.7 Hz, 1H, Ad-H-1′), 5.61 (d, *J* = 8.9 Hz, 1H, Th-H-1′), 4.43–4.39 (br, 1H, Th-H-5′), 4.15–4.07 (m, 3H, Th-H-5″. Th-H-4′, Ad-H-4′), 3.72–3.58 (m, 4H, Ad-H-5′, Ad-H-5′′, Ad-H-2′, Ad-H-2′′), 3.53 (t, *J* = 10.7 Hz, 1H, Ad-H-3′), 3.04 (t, *J* = 11.9 Hz, 1H, Ad-H-3′), 2.95–2.86 (m, 3H, Th-H-3′, Th-H-3″, Th-H-2′), 2.71 (s, 3H, −N­(CH_3_)_2_), 2.69 (s, 3H, −N­(CH_3_)_2_), 2.53 (t, *J* = 11.2 Hz, 1H, Th-H-2″), 1.49 (s, 3H, −C­(CH_3_)_3_); ^13^C­{^1^H} NMR (126 MHz, D_2_O) δ 167.8 (Th-C-4), 158.5 (Ad-C-6), 155.8 (Ad-C-2), 153.0 (Th-C-2), 151.0 (Ad-C-4), 142.2 (Ad-C-8), 139.0 (Th-C-6), 120.9 (Ad-C-5), 113.5 (Th-C-5), 82.9 (Th-C-1′), 81.4 (d, ^3^ *J*_PC_ = 4.3 Hz, Ad-C-1′), 80.6 (d, ^3^ *J*_PC_ = 4.5 Hz, Ad-C-4′), 78.5 (d, ^3^ *J*_PC_ = 10.6 Hz, Th-C-4′), 68.3 (d, ^2^ *J*_PC_ = 4.8 Hz, Th-C-5′), 64.3 (Ad-C-5′), 49.6 (Th-C-2′), 48.6 (Ad-C-2′), 47.0 (Ad-C-3′), 46.3 (Th-C-3′), 38.6, 38.6 (−N­(CH_3_)_2_), 14.1 (Th-C-5-CH_3_); ^31^P­{^1^H} NMR (202 MHz, D_2_O) δ 19.6 (major, *S*p), 19.2 (minor, *R*p); HRMS (ESI-QTOF) *m*/*z*: [M + H]^+^ Calcd for C_22_H_34_N_10_O_7_P^+^, 581.2345; Found, 581.2343.

#### *R*p-A_PN_T Dimer ((*R*p)-**28**)

The crude A_PN_T dimer was purified by automated silica gel column chromatography (ODS, 7 g, S size) using H_2_O–MeCN (100:0–70:30) as an eluent and recycle RP-HPLC (ODS, H_2_O–MeCN (75:25, v/v)) to afford (*R*p)-**28** (9.7 mg, 17 μmol, 55%, dr = 2:98).

^1^H NMR (500 MHz, D_2_O) δ 8.22 (s, 1H, Ad-H-8), 8.06 (s, 1H, Ad-H-2), 7.43 (s, 1H, Th-H-6), 5.77 (d, *J* = 8.7 Hz, 1H, Ad-H-1′), 5.58 (d, *J* = 8.7 Hz, 1H, Th-H-1′), 4.23–4.12 (br, 3H, Th-H-4′, Th-H-5′, Th-H-5″), 4.10–4.04 (br, 1H, Ad-H-4′), 3.79 (t, *J* = 9.3 Hz, 1H, Ad-H-2′), 3.72–3.66 (m, 2H, Ad-H-5′, Ad-H-5″), 3.36–3.29 (m, 2H, Ad-H-2′′, Ad-H-3′), 2.96–2.89 (m, 3H, Ad-H-3′′, Th-H-2′, Th-H-3′), 2.77 (d, *J* = 9.4 Hz, 6H, −N­(CH_3_)_2_), 2.64–2.57 (m, 2H, Th-H-2″, Th-H-3′′), 1.56 (s, 3H, Th-H-5-CH_3_); ^13^C­{^1^H} NMR (126 MHz, D_2_O) δ 168.2 (Th-C-4), 158.1 (Ad-C-6), 155.6 (Ad-C-2), 153.3 (Th-C-2), 150.6 (Ad-C-4), 142.1 (Ad-C-8), 139.5 (Th-C-6), 120.7 (Ad-C-5), 120.7, 113.5 (Th-C-5), 83.0 (Th-C-1′), 81.9 (d, ^3^ *J*_PC_ = 8.7 Hz, Ad-C-1′), 80.1 (d, ^3^ *J*_PC_ = 7.2 Hz, Th-C-4′), 79.3 (d, ^3^ *J*_PC_ = 7.3 Hz, Ad-C-4′), 69.3 (d, ^2^ *J*_PC_ = 5.5 Hz, Th-C-5′), 64.4 (Ad-C-5′), 49.6 (Th-C-2′), 49.4 (Ad-C-2′), 46.7 (Ad-C-3′), 46.3 (Th-C-3′), 38.8, 38.8 (−N­(CH_3_)_2_), 14.0 (Th-C-5-CH_3_); ^31^P­{^1^H} NMR (202 MHz, D_2_O) δ 19.6 (minor, *S*p), 19.4 (major, *R*p).; HRMS (ESI-QTOF) *m*/*z*: [M + H]^+^ Calcd for C_22_H_34_N_10_O_7_P^+^, 581.2345; Found, 581.2344.

#### General Procedure for the Removal of Protecting Groups of C_PN_T Dimers

The C_PN_T dimer (*S*p)-**25** (34 mg, 30 μmol), (*R*p)-**25** (34 mg, 30 μmol) was dissolved in CH_2_Cl_2_ (3.0 mL) (solution G). 3-Cyanopyridine (0.13 g, 1.2 mmol, for (*S*p)-**29** or 0.38 g, 3.6 mmol for (*R*p)-**29**) and CF_3_COOH (90 μL, 1.2 mmol, for (*S*p)-**29**, 28 mL, 3.7 mmol for (*R*p)-**29**) were dissolved in a mixture of CH_2_Cl_2_–CF_3_CH_2_OH (4:1, v/v) (6.0 mL for (*S*p)-**29**) or CH_2_Cl_2_–CF_3_CH_2_OH (3:2, v/v) (9.0 mL for (*R*p)-**29**) (solution H). To solution G, solution H (3.0 mL) was added at RT and allowed to stir for 20 min for (*S*p)-**29**, 30 min for (*R*p)-**29**. Then, the mixture was diluted with CH_2_Cl_2_ (10 mL) and washed with a saturated aqueous solution of NaHCO_3_ (3 × 10 mL). The aqueous layers were combined and extracted with CH_2_Cl_2_ (30 mL), and the collected organic layers were combined, dried over Na_2_SO_4_, filtered, and concentrated under reduced pressure. For the synthesis of (*S*p)-**29**, the residue was dissolved in THF (10 mL), and Et_3_N (0.83 mL, 6.0 mmol) and Et_3_N-3HF (0.49 mL, 3.0 mmol) were added and stirred for 15 h. For the synthesis of (*R*p)-**29**, the residue was dissolved in THF (4.0 mL) (solution I). Et_3_N-3HF (0.98 mL, 6.0 mmol) and Et_3_N (1.7 mL, 12 mmol) were dissolved in THF (13 mL) (solution J). To solution I, solution J (7.5 mL) was added at RT and stirred for 15 h. The mixture was concentrated under reduced pressure. The residue was treated with concentrated aqueous NH_3_–EtOH (3:1, v/v, 8 mL) at 55 °C for 9 h ((*S*p)-**29**) or 24 h ((*R*p)-**29**) in an oil bath. The mixture was diluted with H_2_O (20 mL) and washed with Et_2_O (2 × 20 mL). The aqueous layers were combined and concentrated under reduced pressure.

#### *S*p-C_PN_T Dimer ((*S*p)-**29**)

The crude C_PN_T dimer was purified by automated silica gel column chromatography (ODS, 7 g, S size) using H_2_O–MeCN (100:0–70:30) as an eluent and recycle RP-HPLC (ODS, H_2_O–MeCN (75:25, v/v)) to afford (*S*p)-**29** (5.0 mg, 9.0 μmol, 30%, dr = 97:3).

^1^H NMR (600 MHz, D_2_O) δ 7.73 (d, *J* = 7.4 Hz, 1H, Cy-H-6), 7.56 (s, 1H, Th-H-6), 5.98 (d, *J* = 7.3 Hz, 1H, Cy-H-5), 5.78 (d, *J* = 9.7 Hz, 1H, Th-H-1′), 5.74 (d, *J* = 9.5 Hz, 1H, Cy-H-1′), 4.24–4.07 (m, 3H, Th-H-5′, Th-H-5″, Th-H-4′), 3.96–3.90 (br, 1H, Cy-H-4′), 3.70–3.64 (m, 2H, Cy-H-5′, Cy-H-5′′), 3.53 (t, *J* = 9.9 Hz, 1H, Cy-H-2′), 3.38 (t, *J* = 10.0 Hz, 1H, Cy-H-3′), 3.08 (d, *J* = 12.2 Hz, 1H, Th-H-2′), 3.02–2.94 (m, 2H, Th-H-3′. Cy-H-2″), 2.89–2.81 (m, 3H, Cy-H-3′′, Th-H-2′′, Th-H-3′′), 2.68 (d, *J* = 10.6 Hz, 6H, −N­(CH_3_)_2_), 1.79 (s, 3H, Th-H-5-CH_3_); ^13^C­{^1^H} NMR (126 MHz, D_2_O) δ 168.8 (Th-C-4), 168.7 (Cy-C-4), 159.3 (Cy-C-2), 153.8 (Th-C-2), 144.4 (Cy-C-6), 140.0 (Th-C-6), 114.1 (Th-C-5), 99.0 (Cy-C-5), 83.5 (d, ^3^ *J*_PC_ = 6.5 Hz, Cy-C-1′), 82.7 (Th-C-1′), 80.5 (d, ^3^ *J*_PC_ = 5.0 Hz, Cy-C-4′), 78.5 (d, ^3^ *J*_PC_ = 7.6 Hz, Th-C-4′), 68.6 (d, ^2^ *J*_PC_ = 6.4 Hz, Th-C-5′), 64.4 (Cy-C-5′), 49.2 (Cy-C-2′), 49.0 (Th-C-2′), 46.9 (d, ^2^ *J*_PC_ = 1.9 Hz, Cy-C-3′), 46.3 (Th-C-3′), 38.7, 38.6 (−N­(CH_3_)_2_), 14.3 (Th-C-5-CH_3_); ^31^P­{^1^H} NMR (202 MHz, D_2_O) δ 19.5 (major, *S*p), 19.1 (minor, *R*p); HRMS (ESI-QTOF) *m*/*z*: [M + H]^+^ Calcd for C_21_H_34_N_8_O_8_P^+^, 557.2232.; Found, 557.2218.

#### *R*p-C_PN_T Dimer ((*R*p)-**29**)

The crude C_PN_T dimer was purified by automated silica gel column chromatography (ODS, 7 g, S size) using H_2_O–MeCN (100:0–70:30) as an eluent and recycle RP-HPLC (ODS, H_2_O–MeCN (75:25, v/v)) to afford (*R*p)-**29** (2.7 mg, 4.8 μmol, 16%, dr >1:99).

^1^H NMR (600 MHz, D_2_O) δ 7.73 (d, *J* = 7.1 Hz, 1H, Cy-H-6), 7.65 (s, 1H, Th-H-6), 6.01 (d, *J* = 7.2 Hz, 1H, Cy-H-5), 5.74 (d, *J* = 9.6 Hz, 1H, Th-H-1′), 5.68 (d, *J* = 9.3 Hz, 1H, Cy-H-1′), 4.16–4.10 (m, 3H, Th-H-5′, Th-H-5″, Th-H-4′), 3.97–3.91 (br, 1H, Cy-H-4′), 3.68–3.57 (m, 3H, Cy-H-5′, Cy-H-5′′, Cy-H-2′), 3.28 (t, *J* = 9.8 Hz, 1H, Cy-H-3′), 3.06 (d, *J* = 12.4 Hz, 1H, Th-H-2′), 2.93 (d, *J* = 12.8 Hz, 1H, Th-H-3′), 2.81–2.62 (m, 10H, Th-H-2″, Cy-H-3′′, Cy-H-2′′, −N­(CH_3_)_2_, Th-H-3″), 1.74 (s, 3H, Th-H-5-CH_3_); ^13^C­{^1^H} NMR (126 MHz, D_2_O) δ 168.9 (Cy-C-4), 168.9 (Th-C-4), 159.4 (Cy-C-2), 153.9 (Th-C-2), 144.1 (Cy-C-6), 140.1 (Th-C-6), 114.2 (Th-C-5), 98.9 (Cy-C-5), 83.5 (d, ^3^ *J*_PC_ = 7.1 Hz, Cy-C-1′), 82.6 (Th-C-1′), 80.0 (d, ^3^ *J*_PC_ = 5.5 Hz, Cy-C-4′), 79.0 (d, ^3^ *J*_PC_ = 6.8 Hz, Th-C-4′), 69.1 (d, ^2^ *J*_PC_ = 5.1 Hz, Th-C-5′), 64.5 (Cy-C-5′), 49.5 (Cy-C-2′), 49.1 (Th-C-2′), 46.9 (Cy-C-3′), 46.2 (Th-C-3′), 38.6, 38.6 (−N­(CH_3_)_2_), 14.2 (Th-C-5-CH_3_); ^31^P­{^1^H} NMR (202 MHz, D_2_O) δ 19.2.; HRMS (ESI-QTOF) *m*/*z*: [M + H]^+^ Calcd for C_21_H_34_N_8_O_8_P^+^, 557.2232.; Found, 557.2231.

#### General Procedure for the Removal of Protecting Groups of G_PN_T Dimers

The G_PN_T dimer (*S*p)-**26** (33 mg, 29 μmol), or (*R*p)-**26** (34 mg, 30 μmol), was dissolved in CH_2_Cl_2_ (1.0 mL) (solution I). 3-Cyanopyridine (0.38 g, 3.6 mmol) and CF_3_COOH (0.28 mL, 3.7 mmol) were dissolved in a mixture of CH_2_Cl_2_–CF_3_CH_2_OH (3:2, v/v) (9.0 mL) (solution J). To solution I, solution J (3.0 mL) was added at RT and allowed to stir for 30 min (for (*S*p)-**30**) or 40 min (for (*R*p)-**30**). Then, the mixture was diluted with CH_2_Cl_2_ (10 mL) and washed with a saturated aqueous solution of NaHCO_3_ (3 × 10 mL). The aqueous layers were combined and extracted with CH_2_Cl_2_ (30 mL), and the collected organic layers were combined, dried over Na_2_SO_4_, filtered, and concentrated under reduced pressure. The residue was dissolved in THF (10 mL). To the mixture, Et_3_N (0.83 mL, 6.0 mmol) and Et_3_N-3HF (0.49 mL, 3.0 mmol) were added and stirred for 14 h. The mixture was concentrated under reduced pressure. The residue was treated with concentrated aqueous NH_3_–EtOH (3:1, v/v, 8 mL) at 55 °C for 8 h ((*S*p)-**30** or (*R*p)-**30**) in an oil bath. The mixture was diluted with H_2_O (20 mL) and washed with Et_2_O (2 × 20 mL). The aqueous layers were combined and concentrated under reduced pressure.

#### *S*p-G_PN_T Dimer ((*S*p)-**30**)

The crude G_PN_T dimer was purified by automated silica gel column chromatography (ODS, 7g, S size) using H_2_O–MeCN (100:0–70:30) as an eluent and recycle RP-HPLC (ODS, H_2_O–MeCN (75:25, v/v)). The residue was dissolved in the 20% MeCN in H_2_O (0.73 mL), and a part of the solution (0.39 mL) was purified by using an ACQUITY Premier UPLC system (Waters) with detection at 260 nm at 30 °C and a flow rate of 0.5 mL/min using a C18 column (1.7 μm, 2.1 × 50 nm). The UPLC purification was performed with a linear gradient of 1–7.5% MeCN in a 0.1 M TEAA buffer (pH 7.0) to afford (*S*p)-**30** (1.0 mg, 1.7 μmol, 12%, dr = 99:1).

^1^H NMR (600 MHz, D_2_O) δ 7.97 (s, 1H, Gu-H-8), 7.36 (s, 1H, Th-H-6), 5.74 (d, *J* = 9.4 Hz, 1H, Th-H-1′), 5.68 (d, *J* = 9.2 Hz, 1H, Gu-H-1′), 4.43–4.37 (br, 1H, Th-H-5′), 4.19 (d, *J* = 9.8 Hz, 1H, Gu-H-4′), 4.10 (d, *J* = 11.1 Hz, 1H, Th-H-5″), 4.06–4.00 (br, 1H, Th-H-4′), 3.71–3.61 (m, 3H, Gu-H-5′, Gu-H-5′′, Gu-H-2′), 3.54–3.50 (m, 2H, Gu-H-2′′, Gu-H-3′), 3.03–2.91 (m, 4H, Th-H-2′, Gu-H-3′′, Th-H-3′, Th-H-3′′), 2.72–2.65 (m, 7H, Th-H-2′′, −N­(CH_3_)_2_), 1.55 (s, 3H, Th-H-5-CH_3_); ^13^C­{^1^H} NMR (126 MHz, D_2_O) δ 168.0 (Th-C-4), 161.4 (Gu-C-6), 156.9 (Gu-C-2), 153.7 (Gu-C-4), 153.4 (Th-C-2), 139.5 (Gu-C-8), 139.1 (Th-C-6), 118.7 (Gu-C-5), 113.9 (Th-C-5), 82.5 (Th-C-1′), 81.2 (d, ^3^ *J*_PC_ = 6.1 Hz, Gu-C-1′), 80.4 (d, ^3^ *J*_PC_ = 4.1 Hz, Th-C-4′), 78.0 (d, ^3^ *J*_PC_ = 6.9 Hz, Gu-C-4′), 68.2 (d, ^2^ *J*_PC_ = 5.5 Hz, Th-C-5′), 64.3 (Gu-C-5′), 49.2 (Th-C-2′), 48.5 (Gu-C-2′), 47.0 (Gu-C-3′), 46.2 (Th-C-3′), 38.6, 38.5 (−N­(CH_3_)_2_), 14.0 (Th-C-5-CH_3_); ^31^P­{^1^H} NMR (202 MHz, D_2_O) δ 19.5; HRMS (ESI-QTOF) *m*/*z*: [M + H]^+^ Calcd for C_22_H_34_N_10_O_8_P^+^, 597.2294.; Found, 597.2295.

#### *R*p-G_PN_T Dimer ((*R*p)-**30**)

The crude G_PN_T dimer was purified by automated silica gel column chromatography (ODS, H_2_O–MeCN (100:0–70:30)) and recycled RP-HPLC (ODS, H_2_O–MeCN (75:25, v/v)). The residue was dissolved in the 20% MeCN in H_2_O (0.76 mL), and the solution (0.39 mL) was purified by using an ACQUITY Premier UPLC system (Waters) with detection at 260 nm at 30 °C and a flow rate of 0.5 mL/min using a C18 column (1.7 μm, 2.1 × 50 nm). The UPLC purification was performed with a linear gradient of 1–7.5% MeCN in a 0.1 M TEAA buffer (pH 7.0) to afford (*R*p)-**30** (0.39 mg, 0.65 μmol, 5%, dr > 1:99).

^1^H NMR (600 MHz, D_2_O) δ 7.96 (s, 1H, Gu-H-8), 7.55 (s, 1H, Th-H-6), 5.71 (d, *J* = 9.5 Hz, 1H, Th-H-1′), 5.67 (d, *J* = 9.5 Hz, 1H, Gu-H-1′), 4.24–4.16 (m, 3H, Th-H-5′, Th-H-5″, Th-H-4′), 4.07–4.01 (br, 1H, Gu-H-4′), 3.72–3.64 (m, 3H, Gu-H-5′, Gu-H-5′′, Gu-H-2′), 3.33–3.29 (m, 2H, Gu-H-3′, Gu-H-2′′), 3.12 (d, *J* = 12.3 Hz, 1H, Th-H-2′), 3.03 (d, *J* = 12.5 Hz, 1H, Th-H-3′), 2.94–2.89 (m, 1H, Gu-H-3″), 2.76–2.75 (m, 8H, Th-H-2′′, Th-H-3′′, −N­(CH_3_)_2_), 1.68 (s, 3H, Th-H-5-CH_3_); ^13^C­{^1^H} NMR (126 MHz, D_2_O) δ 168.4 (Th-C-4), 161.6 (Gu-C-6), 156.9 (Gu-C-2), 153.6 (Th-C-2), 153.5 (Gu-C-4), 139.6 (Gu-C-8), 139.5 (Th-C-6), 118.7 (Gu-C-5), 113.9 (Th-C-5), 82.8 (Th-C-1′), 81.8 (d, ^3^ *J*_PC_ = 7.8 Hz, Gu-C-1′), 80.0 (d, ^3^ *J*_PC_ = 6.4 Hz, Gu-C-4′), 78.5 (d, ^3^ *J*_PC_ = 9.6 Hz, Th-C-4′), 69.3 (d, ^2^ *J*_PC_ = 6.1 Hz, Th-C-5′), 64.4 (Gu-C-5′), 49.3 (Gu-C-2′), 49.2 (Th-C-2′), 46.9 (Gu-C-3′), 46.1 (Th-C-3′), 38.7, 38.7 (−N­(CH_3_)_2_), 14.0 (Th-C-5-CH_3_); ^31^P­{^1^H} NMR (202 MHz, D_2_O) δ 19.1; HRMS (ESI-QTOF) *m*/*z*: [M + H]^+^ Calcd for C_22_H_34_N_10_O_8_P^+^, 597.2294.; Found, 597.2295.

## Supplementary Material



## Data Availability

The data underlying this study are available in the published article and its online Supporting Information.

## Notes

Experimental details, copies of ^1^H, ^13^C, ^31^P NMR, COSY, HSQC, and HMBC spectra of new compounds, and final products (PDF)
