# Supplementary material for: Stereocontrolled Synthesis of Dimethylamino Phosphorochloridate Monomers toward Stereopure Phosphorodiamidate Morpholino Oligonucleotides
Source: J Org Chem. 2026 Mar 12;91(12):4170–90. doi: 10.1021/acs.joc.5c02914 (PMC13036775; doi:10.1021/acs.joc.5c02914)
Supplement: Supplementary file 1 [file jo5c02914_si_001.pdf]

Stereocontrolled Synthesis of Dimethylamino Phosphorochloridate Monomers toward Stereopure Phosphorodiamidate Morpholino Oligonucleotides

Ryuichi Inutake[a], Hironao Hasegawa[b], Taiki Tsurusaki[a], Taiichi Sakamoto[c], Kazuki Sato[a], Takeshi Wada[a]*

[a]Department of Medicinal and Life Sciences, Faculty of Pharmaceutical Sciences, Tokyo University of Science, Niijuku, Katsushika, Tokyo, 125–8585, Japan

[b]Discovery Research Laboratories, Nippon Shinyaku Co., Ltd., 3–14–1, Sakura, Tsukuba, Ibaraki, 305–0003, Japan

[c]Department of Life Science, Faculty of Advanced Engineering, Chiba Institute of Technology, Tsudanuma, Narashino, Chiba, 275-0016, Japan

Supporting Information

Table of Contents

1. General information S2
2. Experimental section S2
2.1 Synthesis of *N*²-isobutyryl-*O*⁶-cyanoethyl morpholino guanosine S2
2.2 Synthesis of 5′-*O* -*tert*-butyldiphenylsilyl-morpholino nucleosides S4
3. Additional Information S4
3.1.1 Determination of the structure of byproducts S4
3.1.2 Investigation of dimethylamination conditions for the morpholino cytidine derivative S6
3.2 NMR spectra of the crude mixture S8
3.3 Reaction monitoring by ^{31}P NMR S10
3.4 Elucidation of the mechanism for removal of chiral auxiliary S27
3.5 NOESY and ROESY experiments of $N_{PN}T$ dimers S32
4.1 Copies of ^{1}H, ^{13}C, ^{31}P NMR, COSY, HMBC, HMQC, and HSQC spectra S48
5. References S268

1. General information

All the reactions were conducted under Ar atmosphere. Dry organic solvents were prepared by the appropriate relevant procedures. The ^{1}H NMR spectra were recorded at 400 or 500 MHz and an internal standard was tetramethylsilane (δ 0.00). The ^{13}C NMR spectra were recorded at 126 MHz and an internal standard was a deuterated solvent signal; $CDCl_3$ (δ 77.0). The ^{31}P NMR spectra were recorded at 162 MHz or 202 MHz with 85% H_3PO_4 (δ 0.00) as an external standard in $CDCl_3$. COSY, HSQC, HMQC, and HMBC spectra were recorded on a 400 MHz or 500 MHz NMR spectrometer. IR spectra were obtained using an ATR-IR spectrometer. Analytical TLC was performed on commercial glass plated 0.25 mm thickness silica gel layer. Automated silica gel column chromatography was performed on neutral silica gel (Yamazen UNIVERSAL Premium column (30 μm)) (Yamazen Corporation) using automated flash chromatography system W-prep 2XY (Yamazen Corporation). Compound **S1** was purchased from Sapala Organics.

2. Experimental section

2.1 Synthesis of *N*²-isobutyryl-*O*⁶-cyanoethyl morpholino guanosine

Scheme S1. Synthesis of the *N*²-isobutyryl-*O*⁶-cyanoethyl morpholino guanosine

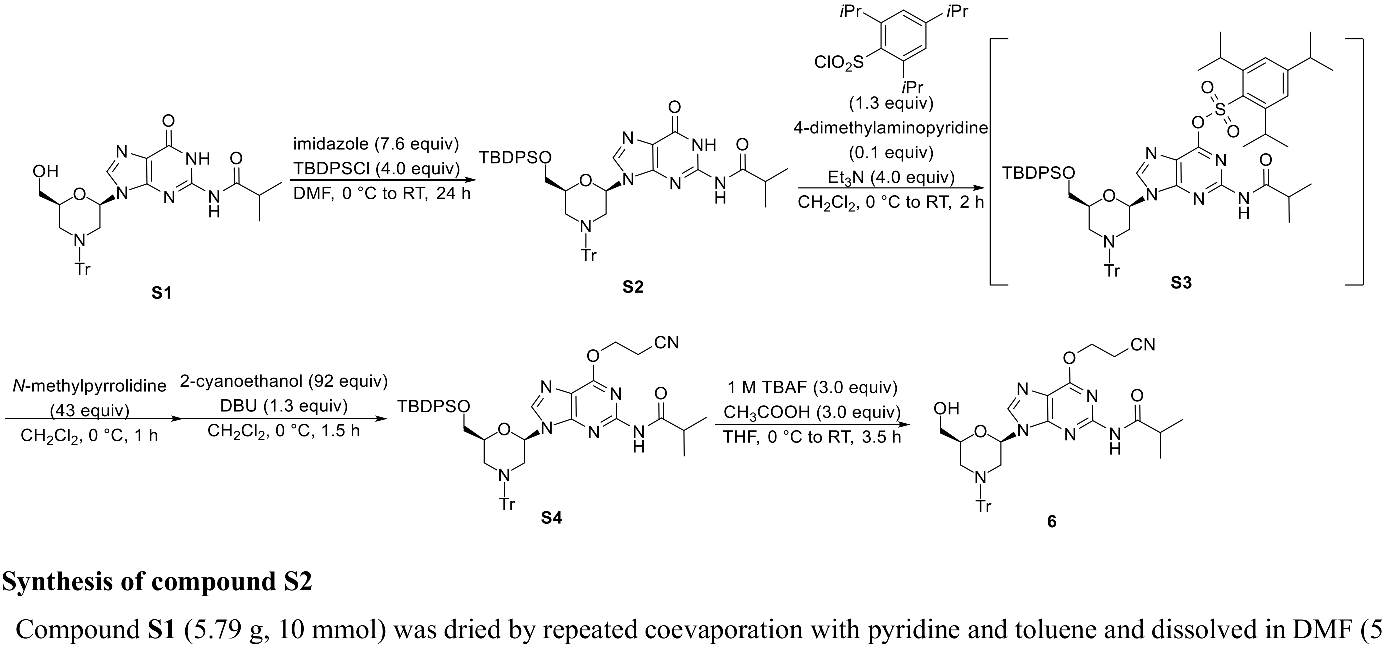

Synthesis of compound S2

Compound **S1** (5.79 g, 10 mmol) was dried by repeated coevaporation with pyridine and toluene and dissolved in DMF (50 mL). The mixture was cooled to 0 °C and imidazole (5.20 g, 76 mmol) and TBDPSCl (10 mL, 40 mmol) were added to the mixture at 0 °C. Then, the mixture was warmed to RT and stirred for 24 h, and the reaction was quenched with MeOH (50 mL). The mixture was concentrated under reduced pressure. The residue was diluted with EtOAc (250 mL) and washed with brines (3×250 mL). The aqueous layers were combined and back-extracted with EtOAc (250 mL). The organic layers were combined, dried over $MgSO_4$, filtered, and concentrated under reduced pressure. The residue was divided into the mixtures and each mixture was purified by automated silica gel column chromatography (neutral, 135 g, 3L) using $CHCl_3$–MeOH (100:0–95:5, v/v) as an eluent. The residue was dissolved in a mixed solvent of $CHCl_3$–toluene–EtOAc (400mL, 3:30:10, v/v/v) and washed with brine (1×300 mL). The aqueous layer was back-extracted with toluene–EtOAc (300 mL, 3:1, v/v). The organic layers were combined, dried over Na_2SO_4, filtered, and concentrated under reduced pressure to afford compound **S2** (colorless foam, 7.56 g, 92%). The ^{1}H NMR spectrum corresponded with literature data[1].

Synthesis of compound S4

Compound **S2** (4.09 g, 5.0 mmol) was dissolved in CH_2Cl_2 (30 mL) and the mixture was cooled to 0 °C (solution A). 4-Dimethylaminopyridine (63 mg, 0.51 mmol) was dissolved in CH_2Cl_2 (3.0 mL) and the mixture was added to the solution A. Et_3N (2.8 mL, 20 mmol) and 2,4,6-triisopropylbenzenesulfonyl chloride (1.97 g, 6.5 mmol) was added to the mixture, and the mixture was stirred for 2 h at RT. The mixture was diluted with CH_2Cl_2 (100 mL) and washed with 1.0 M NaH_2PO_4 aqueous solutions (3×100 mL). The aqueous layers were combined and back-extracted with CH_2Cl_2 (300 mL). The organic layers were combined, dried over Na_2SO_4, filtered, and concentrated under reduced pressure. This compound was used for the next reaction without further purification. The crude mixture of compound **S3** was dissolved in CH_2Cl_2 (25 mL) and cooled to 0 °C. *N*-Methylpyrrolidine (23 mL, 215 mmol) was added to the mixture and stirred for 1 h. Then, 2-cyanoethanol (16 mL, 230 mmol) and 1,8-diazabicyclo[5.4.0]-7-undecene (DBU) (0.97 mL, 6.5 mmol) were added to the mixture. The mixture was stirred for 1.5 h at 0 °C, diluted with CH_2Cl_2 (100 mL), and washed with 1.0 M NaH_2PO_4 aqueous solutions (3×100 mL) and a brine (1×100 mL). The aqueous layers were combined and back-extracted with CH_2Cl_2 (200 mL). The organic layers were combined, dried over Na_2SO_4, filtered, and concentrated under reduced pressure. The crude mixture containing compound **S4** was purified by automated silica gel column chromatography twice using neutral, 135 g, 3L size column and $CHCl_3$−MeOH (99:1−91:9, v/v) as an eluent, for the first time, neutral, 55 g, 2L size column and hexane−EtOAc (70:30−50:50, v/v) for the second time to afford compound **S4** (colorless foam, 3.47 g, 4.0 mmol, 80%).

^{1}H NMR (400 MHz, $CDCl_3$) δ 7.82 (s, 1H, -CONH-), 7.67 (s, 1H, H-8), 7.58–7.37 (m, 12H, Ar), 7.33–7.27 (m, 5H, Ar), 7.19 (t, *J* = 7.1 Hz, 3H, Ar), 6.21 (dd, *J* = 9.6, 2.3 Hz, 1H, H-1′), 4.81–4.69 (m, 2H, $-OCH_2CH_2CN$), 4.35–4.30 (m, 1H, H-4), 3.75 (dd, *J* = 11.0, 4.6 Hz, 1H, H-5′), 3.59 (dd, *J* = 11.0, 5.5 Hz, 1H, H-5″), 3.43 (dt, *J* = 11.0, 2.4 Hz, 1H, H-2′), 3.33 (dt, *J* =11.9, 1.2 Hz, 1H, H-3′), 3.06–2.95 (m, 3H, $-CH(CH_3)_2$, $-OCH_2CH_2CN$), 1.70 (dd, *J* = 11.0, 10.0 Hz, 1H, H-2″), 1.58 (dd, *J* = 11.4, 10.5 Hz, 1H, H-3″), 1.35 (t, *J* = 6.4 Hz, 6H, $-CH(CH_3)_2$), 0.95 (s, 9H, $-C(CH_3)_3$); ^{13}C{^{1}H} NMR (101 MHz, $CDCl_3$) δ 175.5 (-CONH-), 159.5 (C-6), 152.4 (C-4), 151.7(C-2), 139.6 (C-8), 135.5, 135.4, 133.1, 133.0, 129.7, 129.7, 129.1, 127.8, 127.6, 126.4 (Ar), 117.4 (C-5), 116.8 (-CN), 80.4 (C-1′), 77.1 (C-4′), 76.8 ($C(Ar)_3$), 64.4 (C-5′), 61.5 ($-CH_2CH_2CN$), 53.1 (C-2′), 49.7 (C-3′), 35.8 ($-CH(CH_3)_2$), 26.7 ($-C(CH_3)_3$), 19.3, 19.3 ($-CH(CH_3)_2$), 19.1 ($-C(CH_3)_3$), 18.0 ($-CH_2CH_2CN$); HRMS (ESI-QTOF) *m/z*: [M + H]$^+$ Calcd for $C_{52}H_{56}N_7O_4Si^+$,870.4158; Found, 870.4165.

Synthesis of Compound 6

Compound **S4** (1.74 g, 2.0 mmol) was dissolved in THF, and the mixture was cooled to 0 °C. AcOH (0.34 mL, 6.0 mmol) and 1.0 M TBAF (6.0 mL) were added to the mixture. Then, the mixture was stirred for 5 min, warmed to RT, and stirred for a further 3.5 h. Then, the mixture was diluted with EtOAc (100 mL) and washed with saturated $NaHCO_3$ aqueous solutions (3×100 mL). The aqueous layers were combined and back-extracted with EtOAc (150 mL). The organic layers were combined, dried over Na_2SO_4, filtered, and concentrated under reduced pressure. The crude mixture containing compound **6** was purified by automated silica gel column chromatography (neutral, 40 g, L size) using $CHCl_3$−MeOH (99:1−95:5, v/v) as an eluent to afford **6** (colorless foam, 1.11 g, 1.7 mmol, 87%).

^{1}H NMR (400 MHz, $CDCl_3$) δ 8.36 (s, 1H, -CONH-), 7.77 (s, 1H, H-8), 7.5–7.4 (br, 6H, Ar), 7.29 (t, *J* = 8.5 Hz, 6H, Ar), 7.17 (t, *J* = 7.1 Hz, 3H, Ar), 6.21 (dd, *J* = 9.6, 2.3 Hz, 1H, H-1′), 4.74–4.69 (m, 2H, $-OCH_2CH_2CN$), 4.28–4.23 (m, 1H, H-4′), 3.62–3.54 (m, 2H, H-5′, H-5″), 3.40 (dt, *J* = 11.4, 2.4 Hz, 1H, H-2′), 3.14–3.06 (m, 2H, H-3′, $-CH(CH_3)_2$), 2.94–2.85 (m, 3H, $-OCH_2CH_2CN$, OH), 1.76 (dd, *J* = 11.2, 10.8 Hz, 1H, H-2″), 1.56 (dd, *J* = 11.6, 11.2 Hz, 1H, H-3″), 1.35 (d, *J* = 6.9 Hz, 3H, $-CH(CH_3)_2$), 1.33 (d, *J* = 6.9 Hz, 3H, $-CH(CH_3)_2$); ^{13}C{^{1}H} NMR (101 MHz, $CDCl_3$), δ 176.1 (-CONH-), 159.5 (C-6), 152.3 (C-4), 151.8 (C-2), 139.5 (C-8), 129.1, 127.9, 126.5 (Ar), 117.2 (C-5), 116.8 (-CN), 80.4 (C-1′), 77.5 (C-4′), 76.7 ($C(Ar)_3$), 63.4 (C-5′), 61.5 ($-CH_2CH_2CN$), 52.8 (C-2′), 48.7 (C-3′), 35.7 ($-CH(CH_3)_2$), 19.3, 19.2 ($-CH(CH_3)_2$), 17.9 ($-CH_2CH_2CN$); HRMS (ESI-QTOF) *m/z*: [M + H]$^+$ Calcd for $C_{36}H_{38}N_7O_4^+$,632.2980 ; Found, 632.3007.

2.2 Synthesis of 5′-*O* -*tert*-butyldiphenylsilyl-morpholino nucleosides

Synthesis of compound 19−21

The compounds were synthesized according to the method of reference[2], and the ^{1}H NMR spectrum corresponded with literature data.

Synthesis of compound 22

Compound **S2** (0.76 g, 1.0 mmol) was dissolved in CH_2Cl_2 (10 mL) and the mixture was cool to 0 °C. 6% dichloroacetic acid in CH_2Cl_2 (10 mL) was added to the mixture and warmed to RT. The mixture was sttired for 20 min and diluted with MeOH (10 mL). The mixture was washed with saturated aqueous solutions of $NaHCO_3$ (3×20 mL), and the aqueous layers were combined and back-extracted with CH_2Cl_2 (50 mL). The combined organic layers were dried over with Na_2SO_4, filtered, and concentrated under reduced pressure. The crude mixture containing compound **22** was purified by automated silica gel column chromatography (neutral, 40 g, L size) using $CHCl_3$−MeOH (95:5−88:12, v/v) as an eluent to afford **22** (colorless foam, 0.43 g, 0.75 mmol, 76%).

^{1}H NMR (400 MHz, $CDCl_3$) δ 7.74 (s, 1H, H-8), 7.62 (td, *J* = 8.1, 1.5 Hz, 4H, Ar), 7.40–7.31 (m, 6H, Ar), 5.65 (dd, *J* = 9.8, 2.5 Hz, 1H, H-1′), 3.93–3.88 (m, 1H, H-4′), 3.75–3.65 (m, 2H, H-5′), 3.25 (dd, *J* = 12.3, 2.3 Hz, 1H, H-2′), 3.13 (dd, *J* = 12.8, 2.4 Hz, 1H, H-3′), 2.86–2.73 (m, 2H, H-2″, H-3″), 2.69–2.61 (m, 1H, -C<u>H</u>(CH_3)$_2$), 1.23 (t, *J* = 7.1 Hz, 6H, -CH(C<u>H</u>$_3$)$_2$), 1.02 (s, 9H, C(C<u>H</u>$_3$)$_3$)); ^{13}C{^{1}H} NMR (101 MHz, $CDCl_3$) δ 179.0 (-NH<u>C</u>O-), 155.7 (C-6), 147.9 (C-4), 147.6 (C-2), 136.4 (C-8), 135.5, 135.4, 133.0, 132.9, 129.8, 129.8, 127.7, 127.7 (Ar), 120.8 (C-5), 80.5 (C-1′), 78.4 (C-4′), 64.5 (C-5′), 50.2 (C-2′), 46.5 (C-3′), 36.3 (-<u>C</u>H(CH_3)$_2$), 26.7 (-C(<u>C</u>H$_3$)$_3$), 19.2 (-<u>C</u>(CH_3)$_3$), 19.0, 18.9 (CH(<u>C</u>H$_3$)$_2$); HRMS (ESI-QTOF) *m/z*: [M + H]$^+$ Calcd for $C_{30}H_{39}N_6O_4Si^+$, 575.2797 ; Found, 575.2798.

3. Additional Information

3.1.1 Determination of the structure of byproducts

The synthesis of a chloridate monomer of the morpholino cytidine derivative using the reaction conditions in Table 4, Entry 8 resulted in a side reaction on the nucleobase. Isolation of the byproduct was troublesome due to lability of the chloridate moiety. Thus, to elucidate the side reaction on the nucleobase, the obtained chloridate derivative was condensed with morpholine to convert to a stable phosphorodiamidate derivative.

Scheme S2. Synthesis of the chloridate derivative

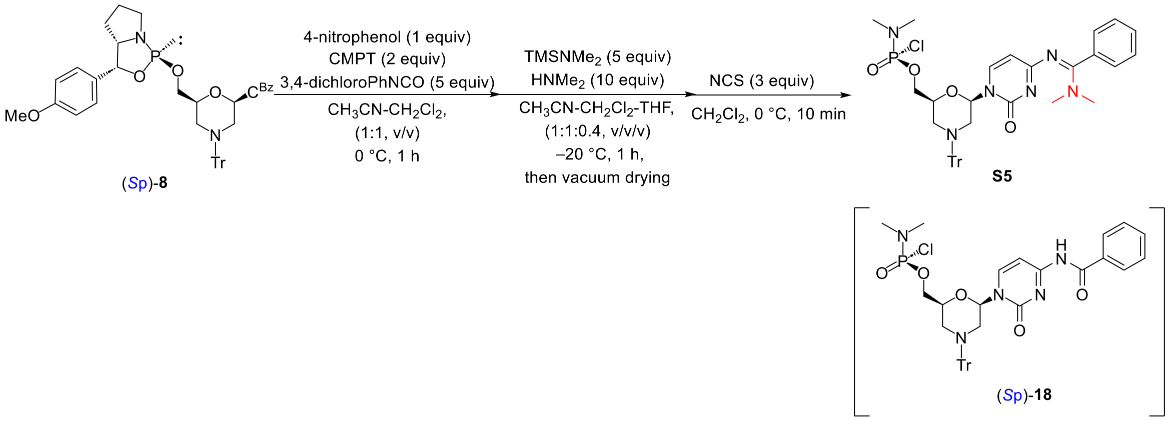

Scheme S3. Condensation of the chloridate derivative with morpholine

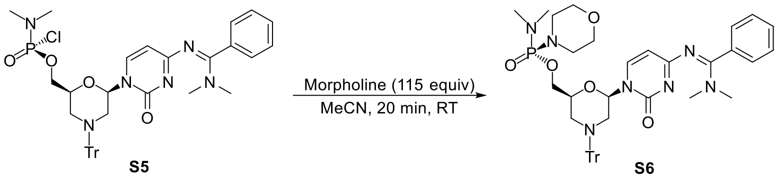

After the synthesis of the chloridate derivative, the crude mixture was dissolved in MeCN (2.0 mL) and added morpholine (1.0 mL, 11 mmol). The mixture was stirred for 20 min at RT and concentrated under reduced pressure. The residue was purified by automated silica gel column chromatography (neutral, 7 g, S size) using ($CHCl_3$:MeOH =99:1−80:20) as an eluent, and preparative TLC (neutral silica gel) using (hexane:EtOAc=1:1) as an eluent to afford to compound **S6**, and the structure of compound **S6** was determined by ^{1}H NMR and ESI-MS. In ^{1}H NMR spectrum, signals at 3.16 and 2.87 ppm, which were corresponded to protons of a dimethylamino group, were observed. Since the signal corresponded to the protons of the dimethylamino group attached to the phosphorus atom was detected at 2.55 and 2.52 ppm, indicating that the byproduct contained an additional dimethylamino group. In combination with the result from an HRMS analysis, the structure of the byproduct was assigned as compound **S6** bearing an amidine structure on the nucleobase.

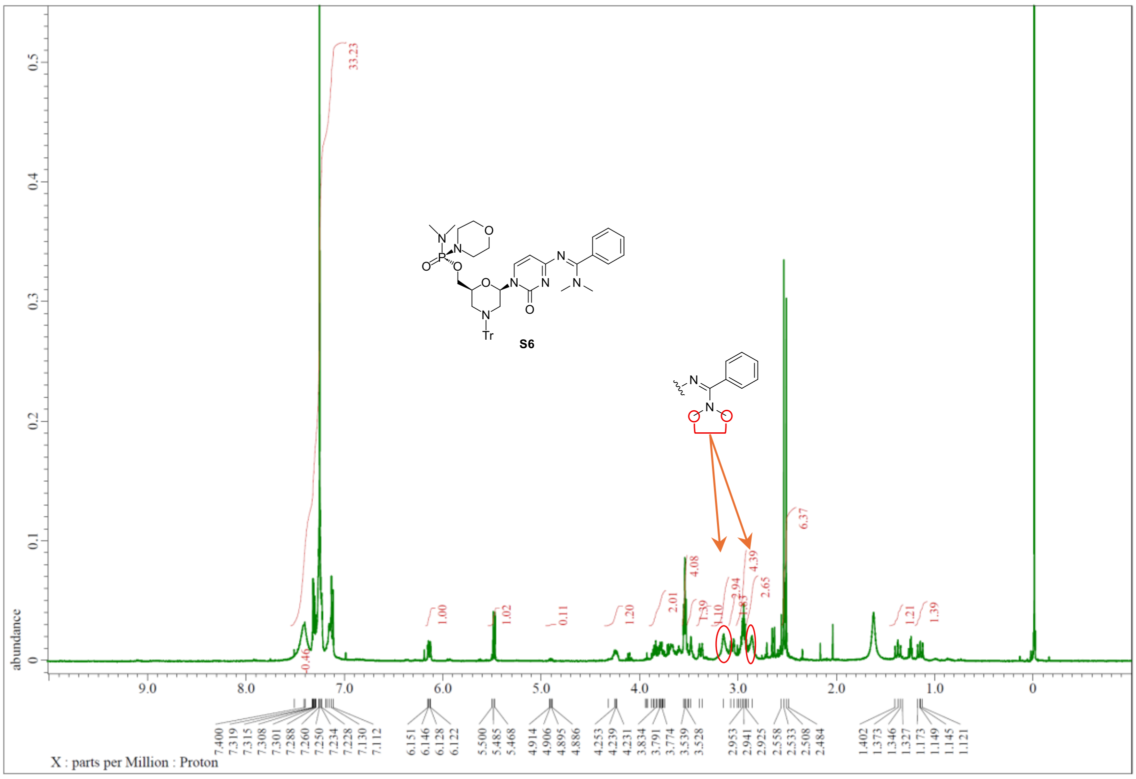

Figure S1. ^{1}H NMR of S6

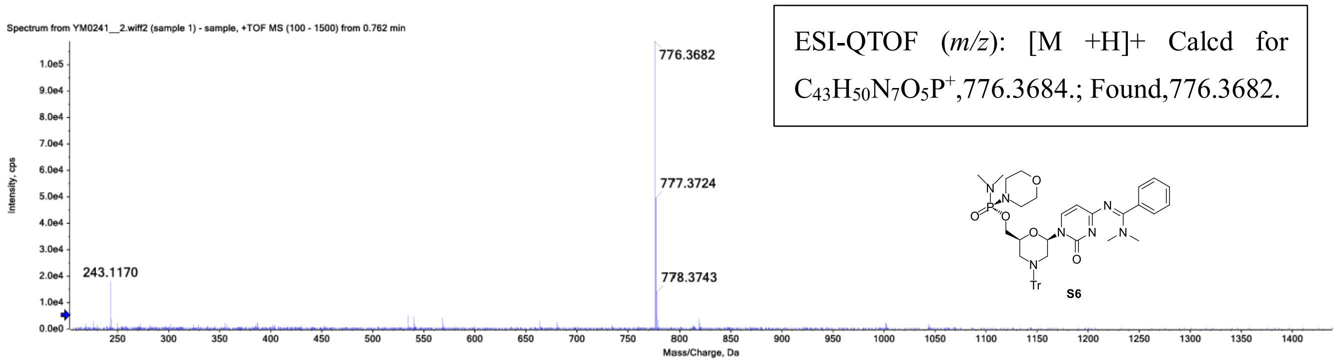

ESI-QTOF (*m/z*): [M +H]+ Calcd for $C_{43}H_{50}N_7O_5P^+$,776.3684.; Found,776.3682.

Figure S2. Mass spectra of S6

3.1.2 Investigation of dimethylamination conditions for the morpholino cytidine derivative

As discussed earlier, a byproduct with an amidine structure on a protecting group of the nucleobase was formed as a main product for the synthesis of the cytidine derivative (**Scheme S4**, **Table S1**, Entry 1). The byproduct was not generated in the absence of $TMSNMe_2$ (Entry 2). Therefore, we hypothesized that the oxygen atom of the amide was activated by $TMSNMe_2$ and replaced with a dimethylamine group (**Scheme S5**). Thus, we investigated dimethylamination conditions using a silylation reagent with a lower reactivity than $TMSNMe_2$.

Scheme S4. Synthesis of chloridate monomers by previous conditions

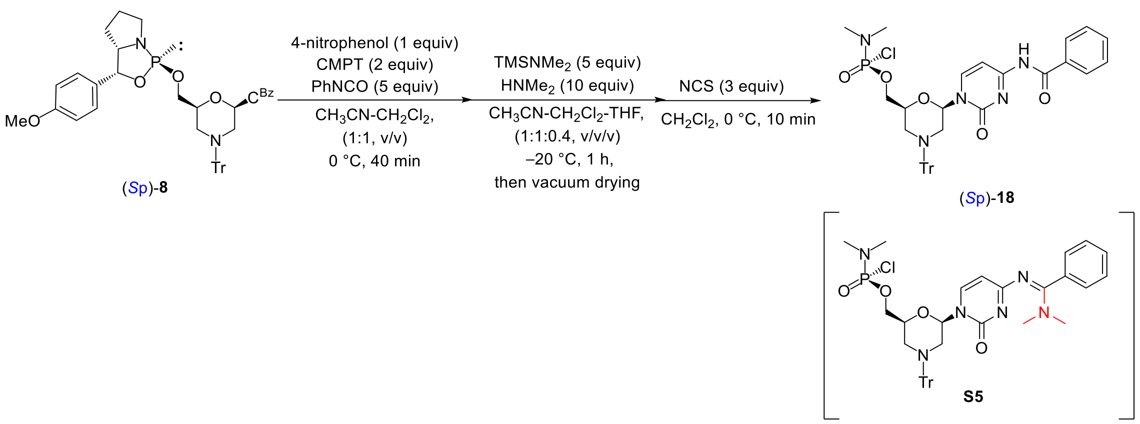

Table S1. Investigations of dimethylamination conditions for morpholino cytidine nucleotides

Entry	Silylation reagent	$HNMe_2$ (equiv)	Temperature (°C)	dr[a]	**18**:**S5**[a]
1	$TMSNMe_2$	10	0	97:3	<1:99
2	–	10	0	74:26	>99:1

a **Determined by ^{31}P NMR.**

Scheme S5. Plausible mechanism for amidination

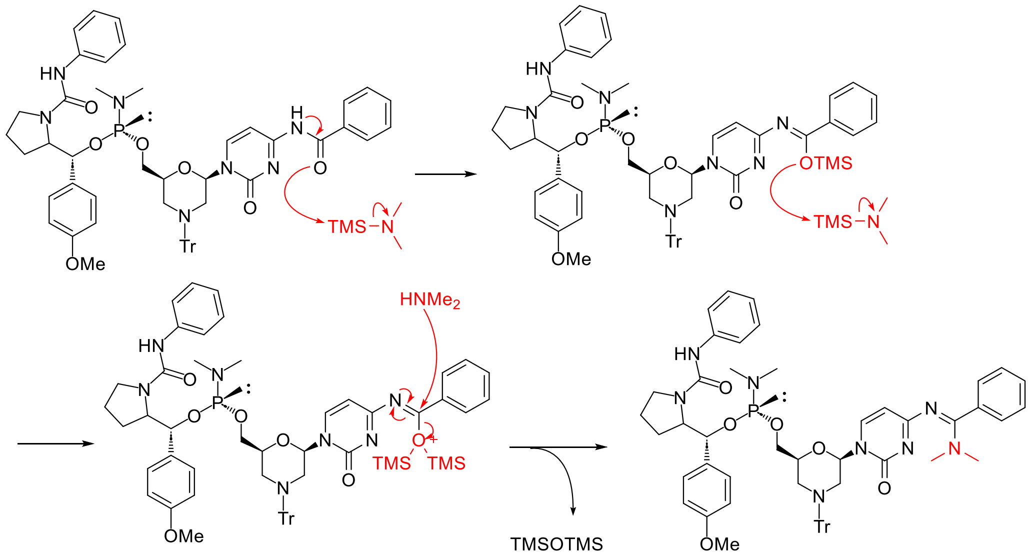

In **Table S2**, diastereomer ratio (dr) of phosphorochloridate and ratio of **18** and **S5** were determined by ^{31}P NMR. The ratio of **18** (desired product) and **S5** (byproduct) was calculated from ^{31}P NMR spectrum to convert the phosphorochloridate derivatives **18** and **S5** into stable phosphorodiamidate derivatives **S7** and **S8** by condensation with $HNMe_2$. Dimethylketene methyl trimethylsilyl acetal (DMTA) was used as a silylation reagent. In Entry 1, 5 equiv of DMTA and 20 equiv of dimethylamine were used in the dimethylamination step, but almost half of the amide moiety was converted to the amidine moiety. The equivalents of DMTA were reduced to 3 equiv, and the formation of the amidine structure was suppressed (S7:S8 = 70:30, Entry 2). Lowering the reaction temperature to -20 ˚C almost completely inhibited the formation of the byproduct (Entry 3). From these investigations, the conditions shown in entry 3 were found to be optimal.

Scheme S6. Investigations of dimethylamination conditions for morpholino cytidine nucleotides

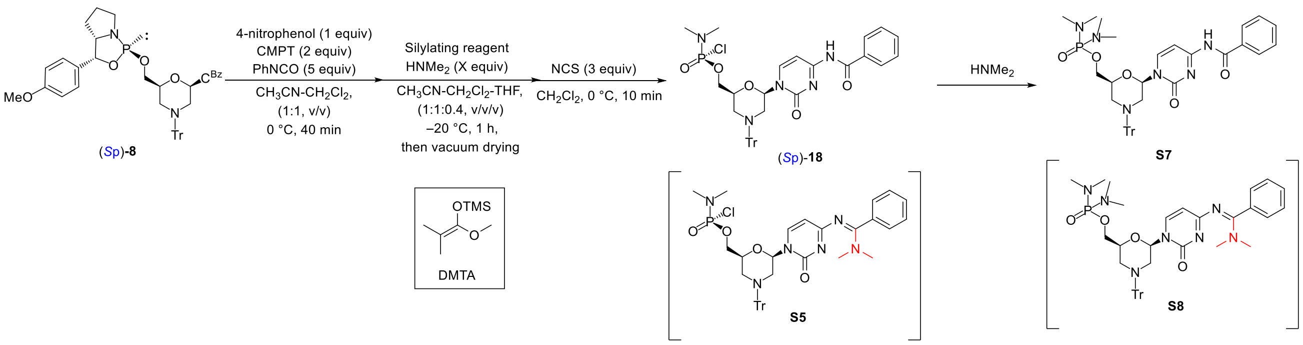

Table S2. Investigations of dimethylamination conditions for morpholino cytidine nucleotides

Entry	Silylation reagent	$HNMe_2$ (equiv)	Temperature (°C)	dr[a]	**S7**:**S8**[a]
1	DMTA (5 equiv)	20	0	95:5	52:48
2	DMTA (3 equiv)	20	0	95:5	70:30
3	DMTA (3 equiv)	20	-20	95:5	97:3

[a] **Determined by ^{31}P NMR.**

3.2 NMR spectra of the crude mixture

Crude mixture of (*R*p)-**13** synthesized under the conditions shown in **Table 3, Entry 4**

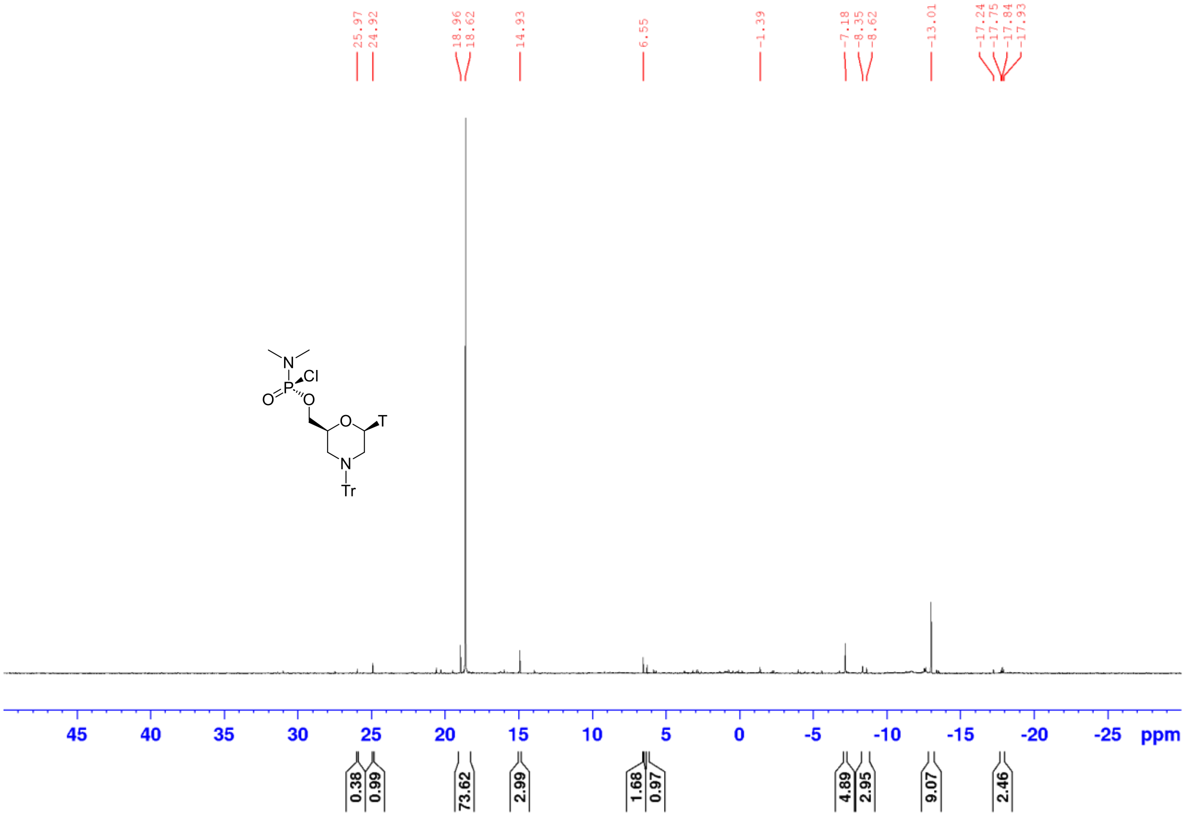

Figure S3. ^{31}P NMR spectrum of crude mixture

Crude mixture of (*R*p)-**13** synthesized under the conditions shown in **Table 4, Entry 1**

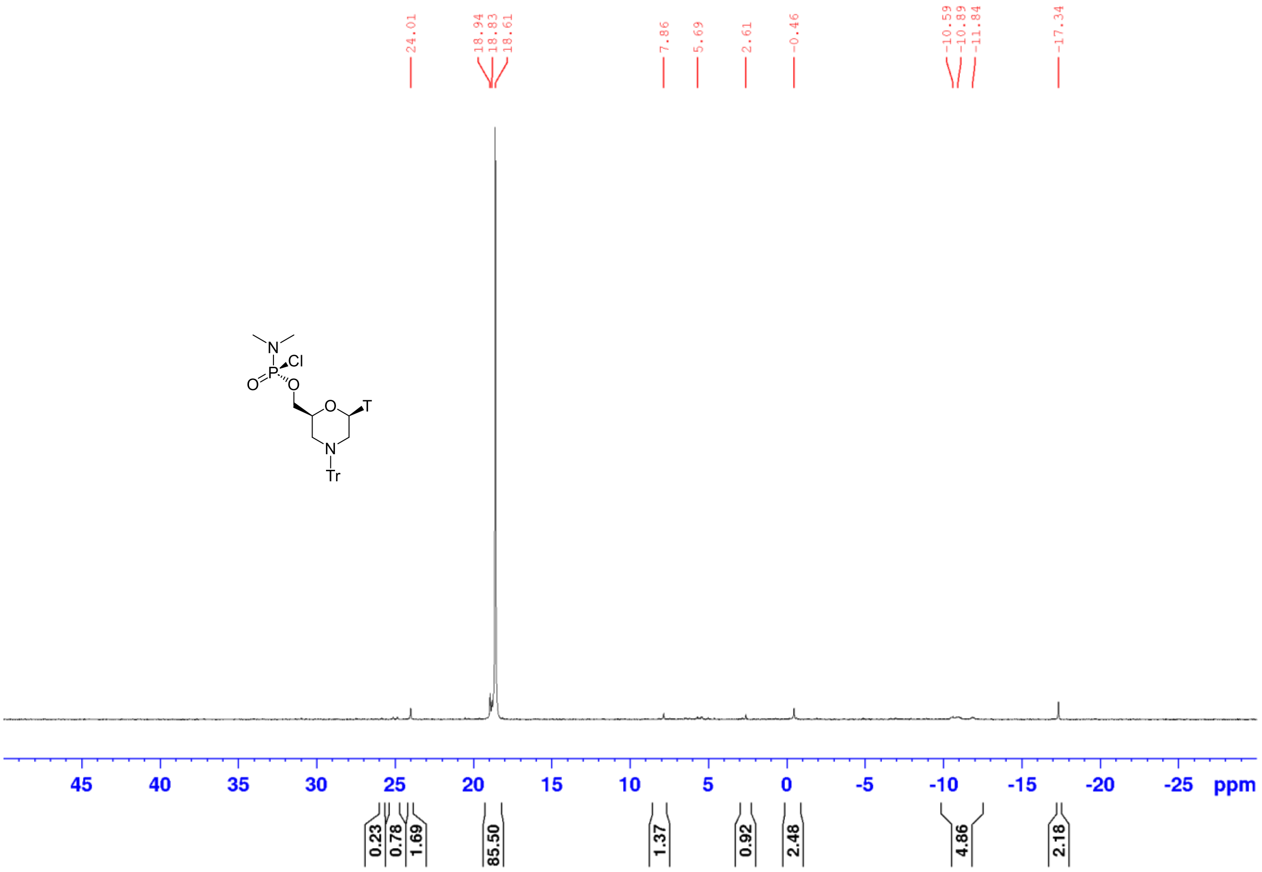

Figure S4. ^{31}**P NMR spectrum of crude mixture**

Crude mixture of fully deprotected C$_{PN}$T dimer (*R*p)-**29**

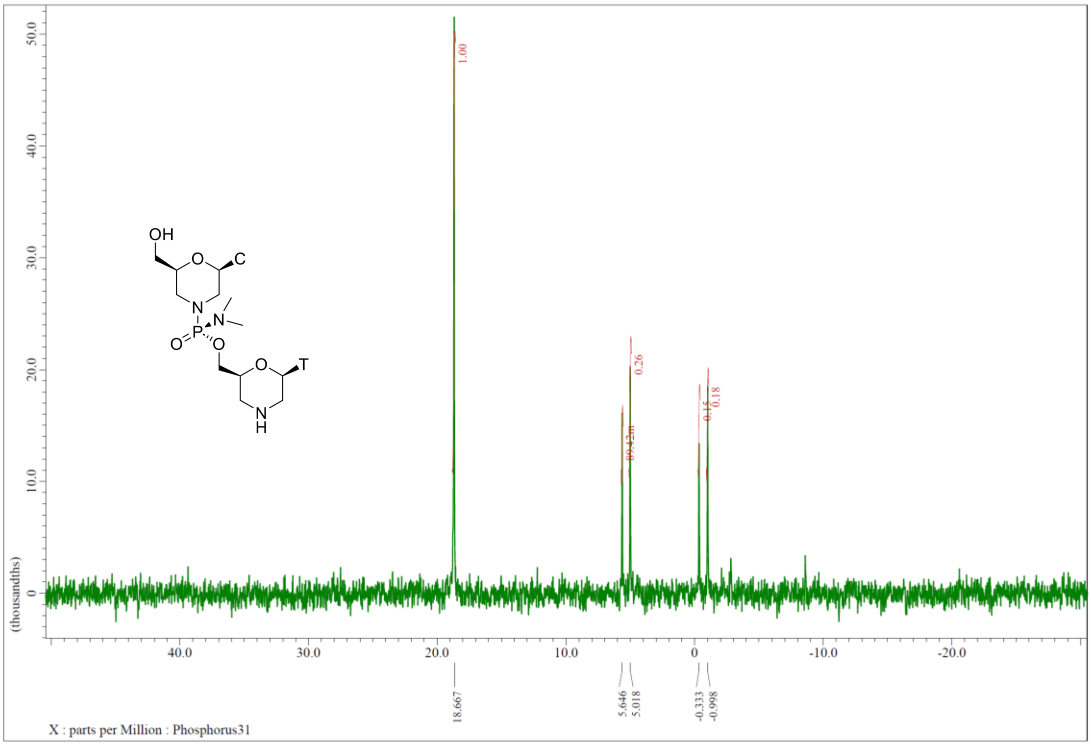

Figure S5. ^{31}**P NMR spectra of crude mixture**

3.3 Reaction monitoring by ^{31}P NMR

Scheme S7. Control experiment for the elucidation of formation mechanism of a byproduct (δ_P = 14)

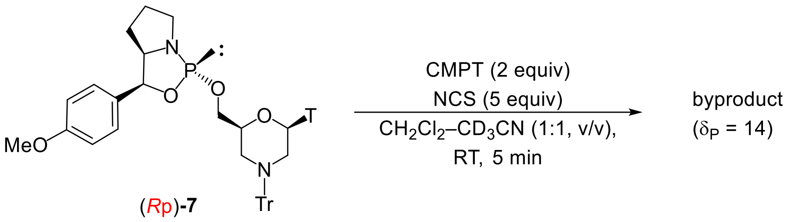

The reaction of (*R*p)-7 with CMPT and NCS resulted in the formation of a byproduct (δ_P = 14). The structure of byproduct was not determined.

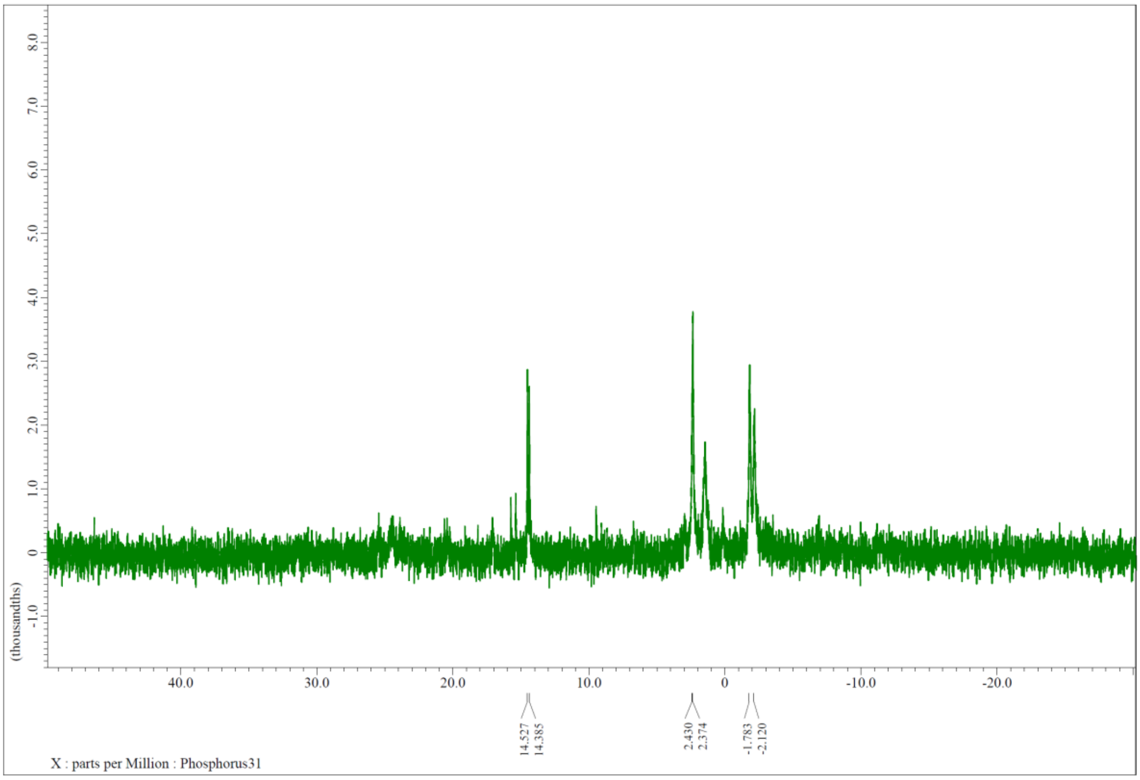

Figure S6. ^{31}P {^{1}H} NMR spectrum of the control experiment for the elucidation of the formation mechanism of a byproduct (δ_P = 14)

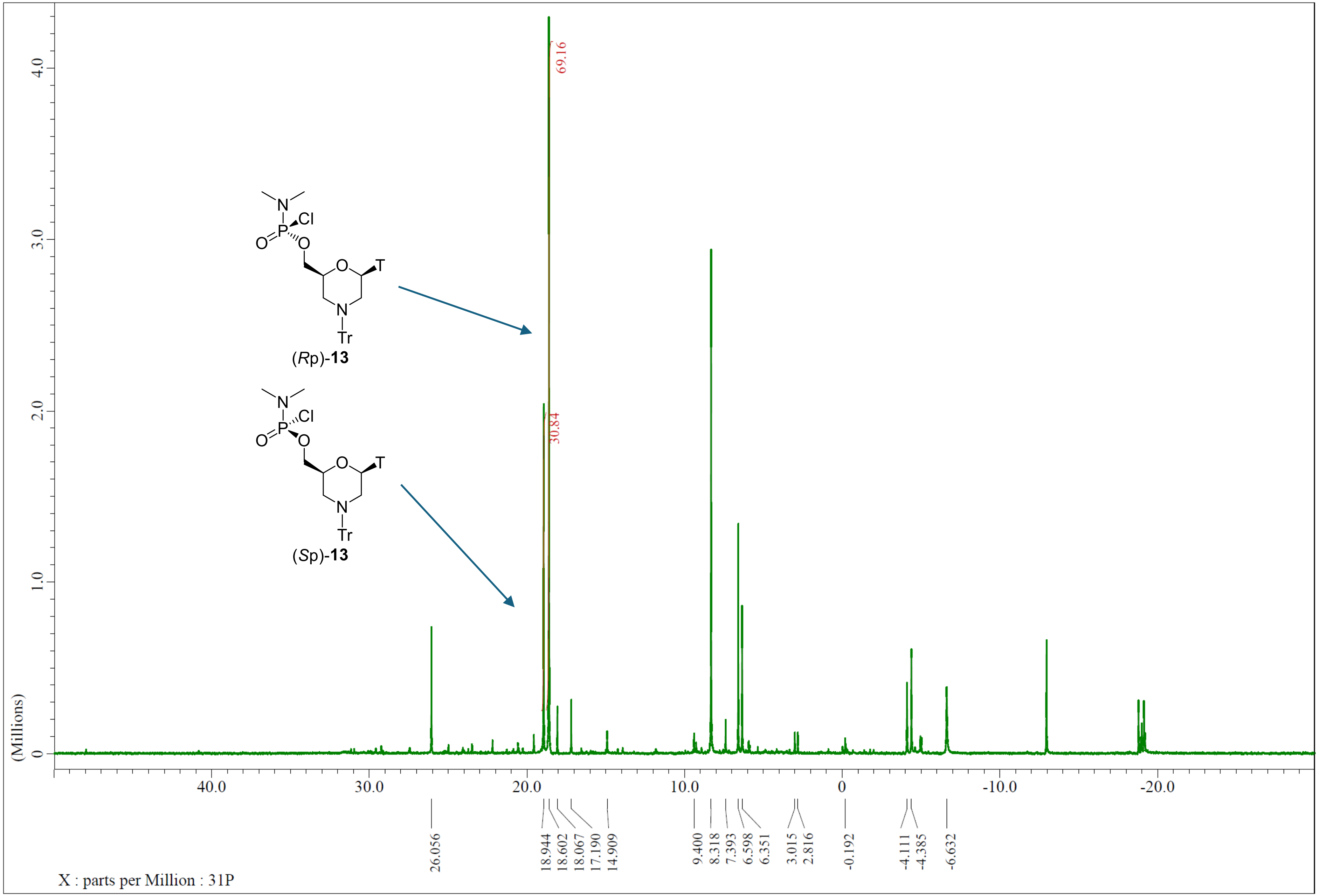

Figure S7. ^{31}**P NMR spectrum of the reaction mixture of Table 2, Entry 1**

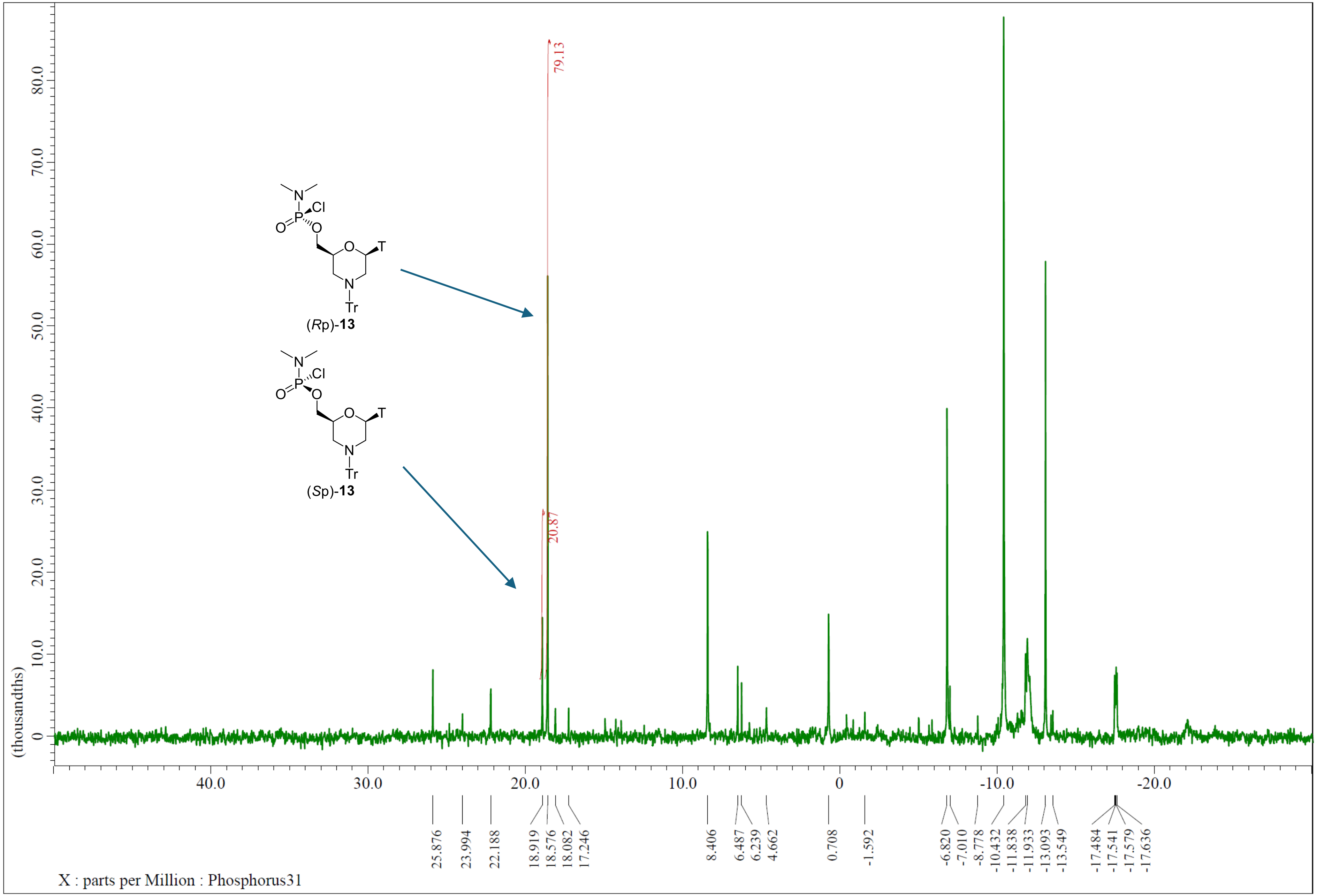

Figure S8. ^{31}P NMR spectrum of the reaction mixture of Table 2, Entry 2

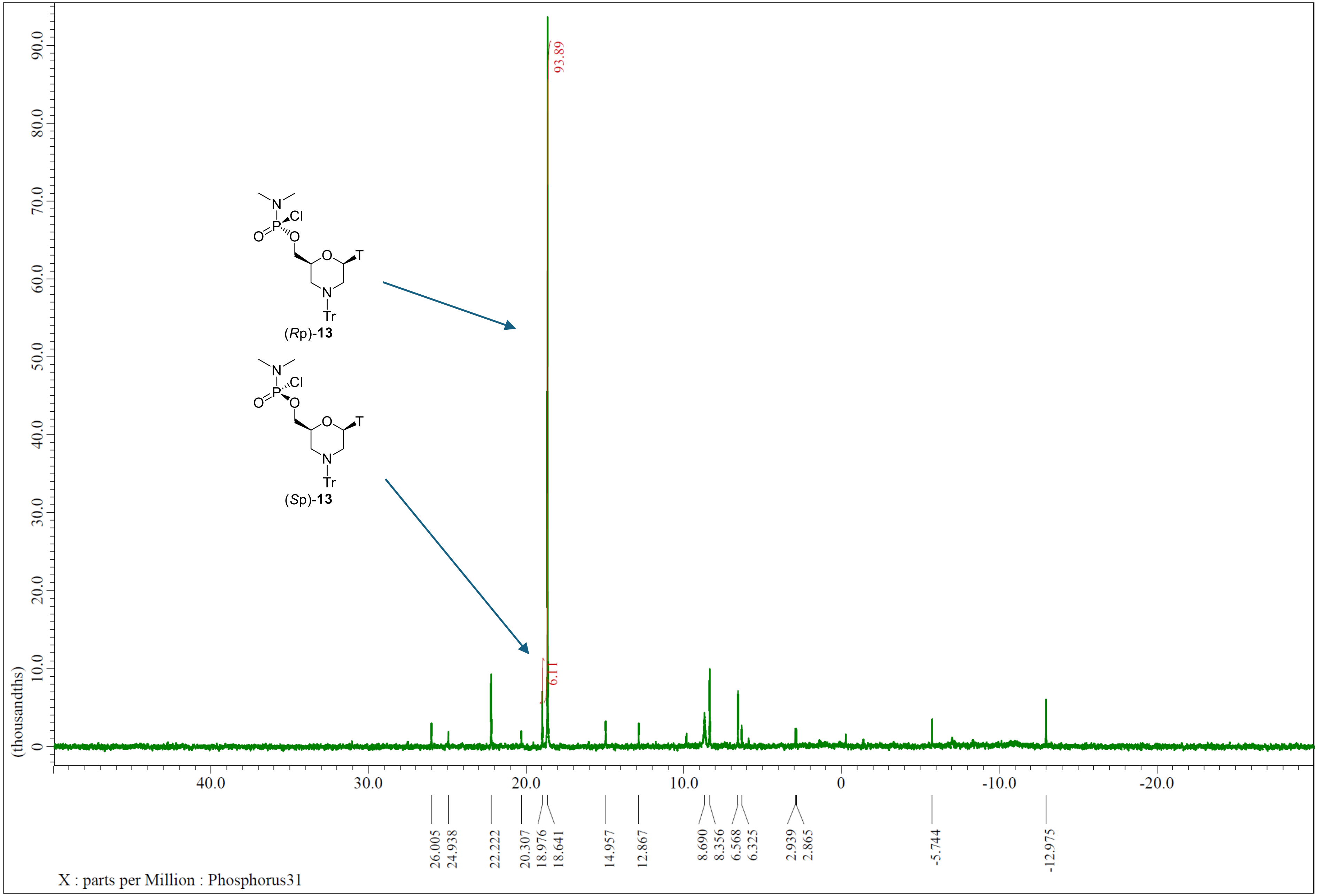

Figure S9. ^{31}P NMR spectrum of the reaction mixture of Table 2, Entry 3

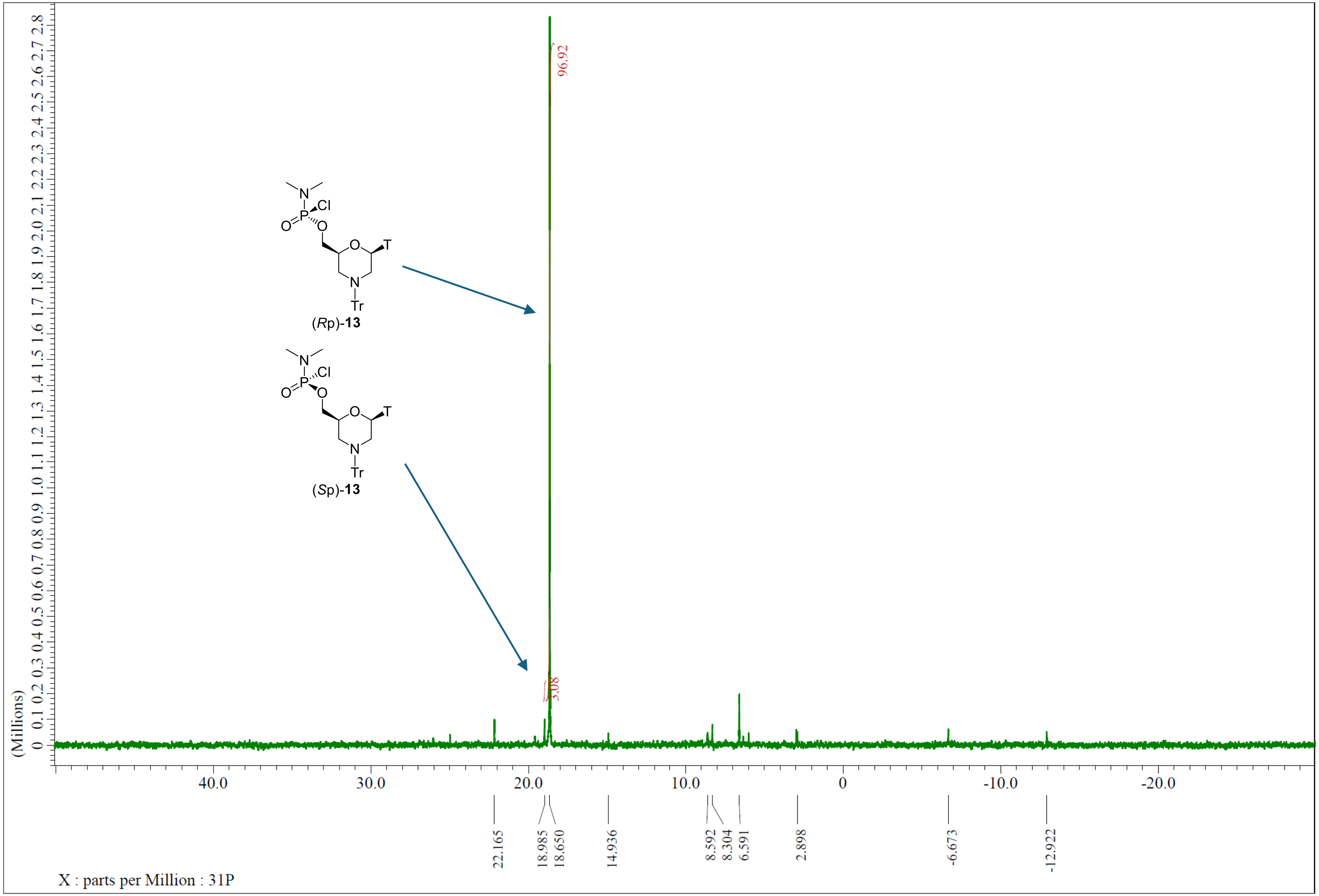

Figure S10. ^{31}**P NMR spectrum of the reaction mixture of Table 2, Entry 4**

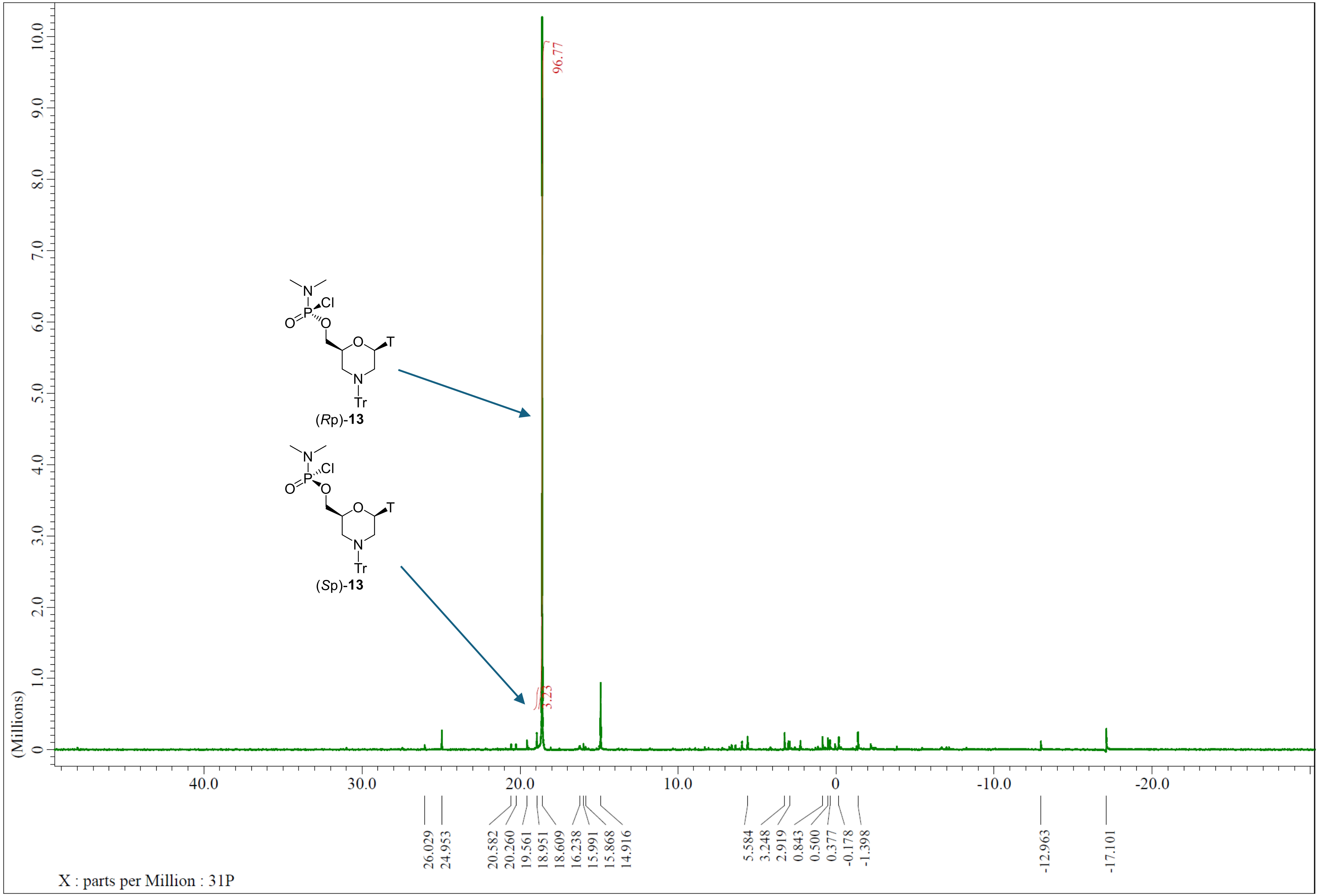

Figure S11. ^{31}**P NMR spectrum of the reaction mixture of Table 2, Entry 5**

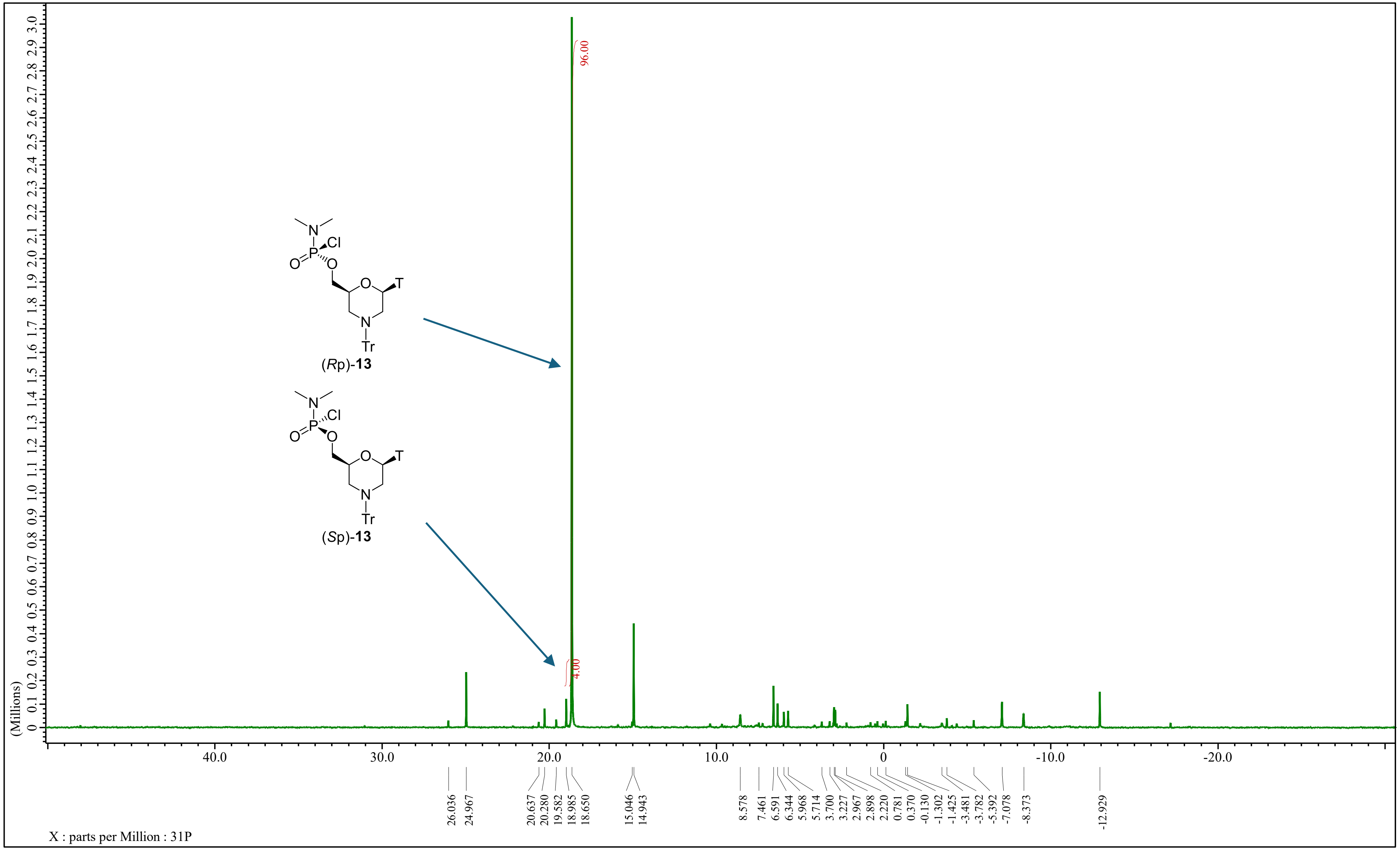

Figure S 12. ^{31}P NMR spectrum of the reaction mixture of Table 2, Entry 6

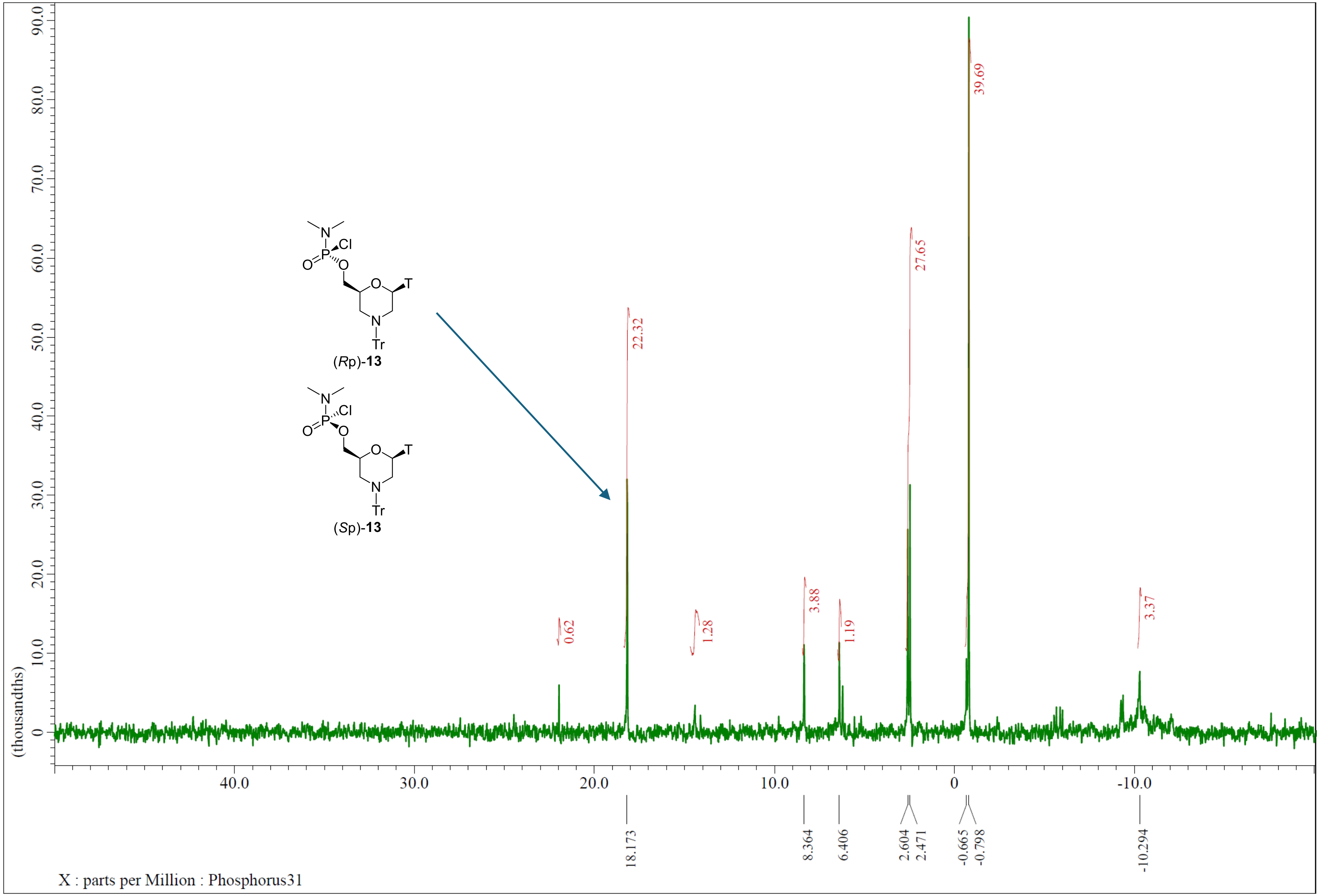

Figure S13. ^{31}**P NMR spectrum of the reaction mixture of Table 3, Entry 1**

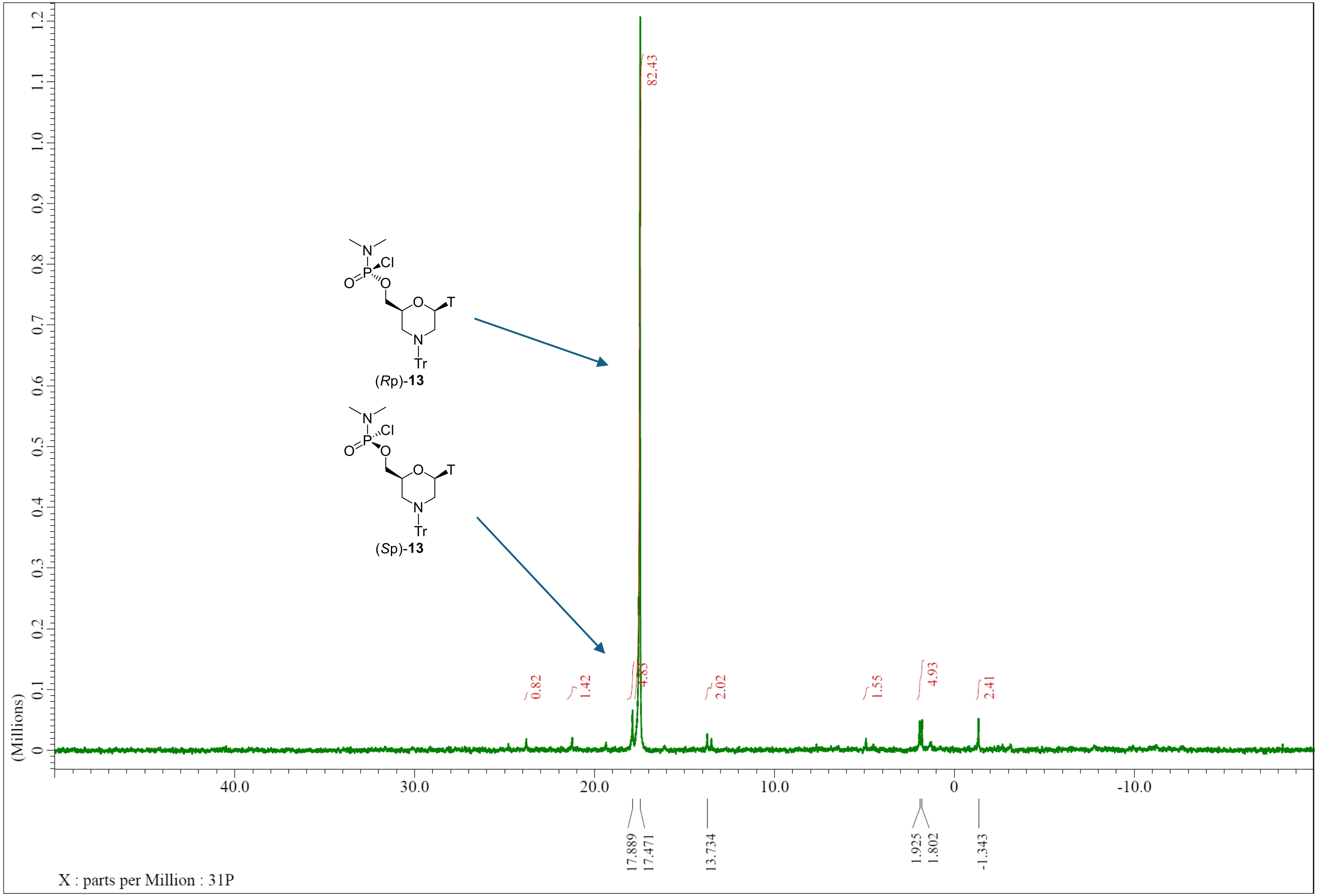

Figure S14. ^{31}**P NMR spectrum of the reaction mixture of Table 3, Entry 2**

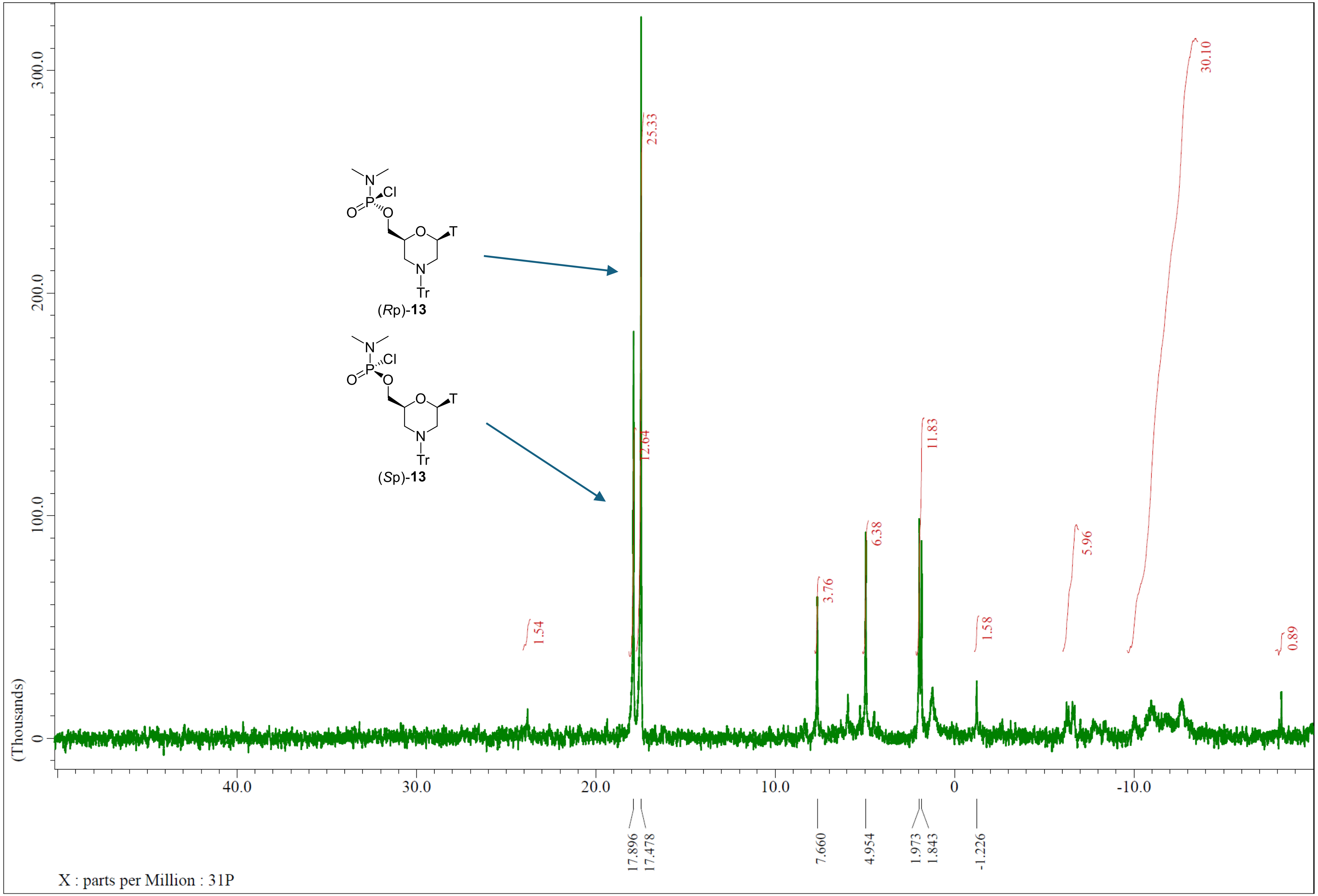

Figure S15. ^{31}**P NMR spectrum of the reaction mixture of Table 3, Entry 3**

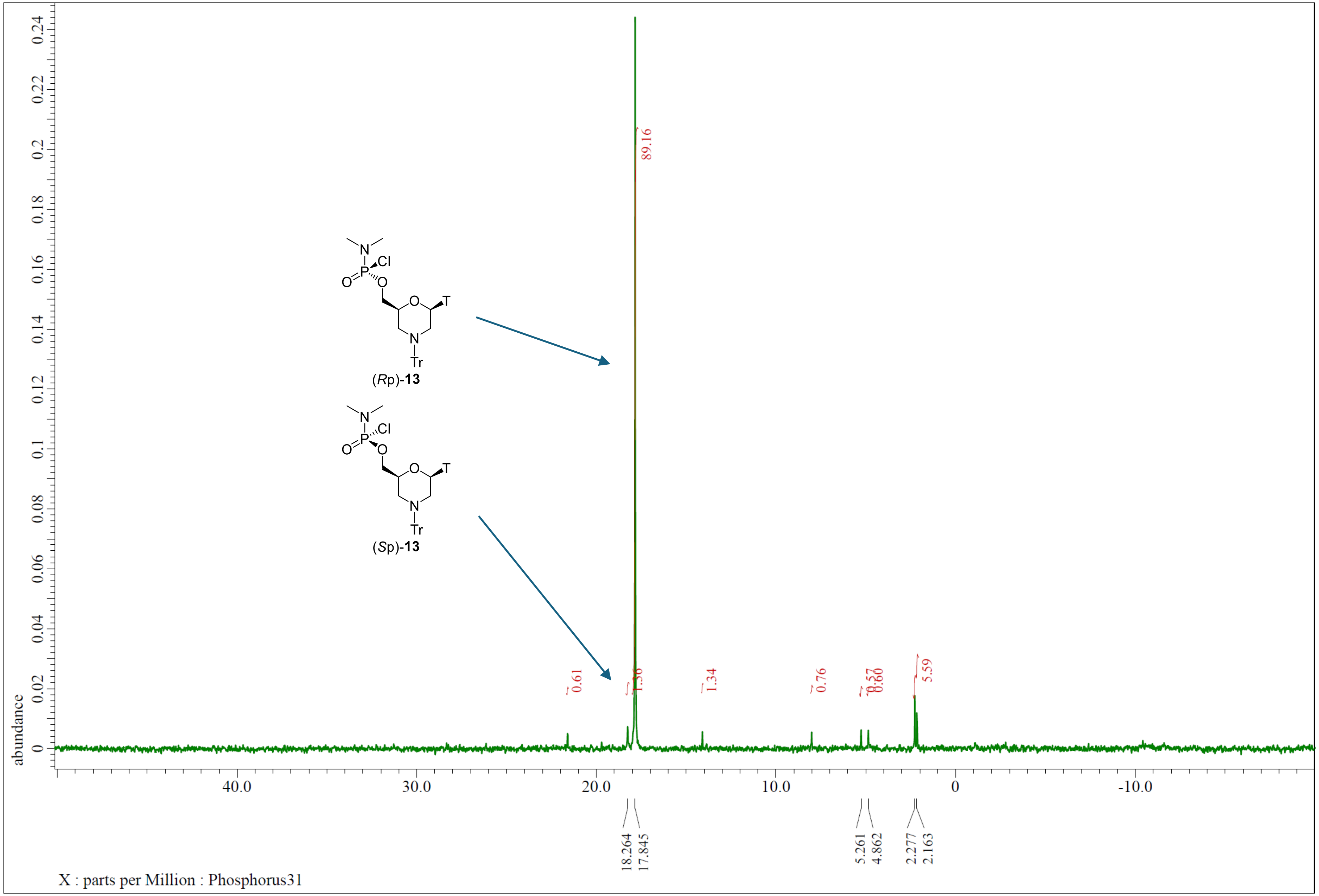

Figure S16. ^{31}**P NMR spectrum of the reaction mixture of Table 3, Entry 4**

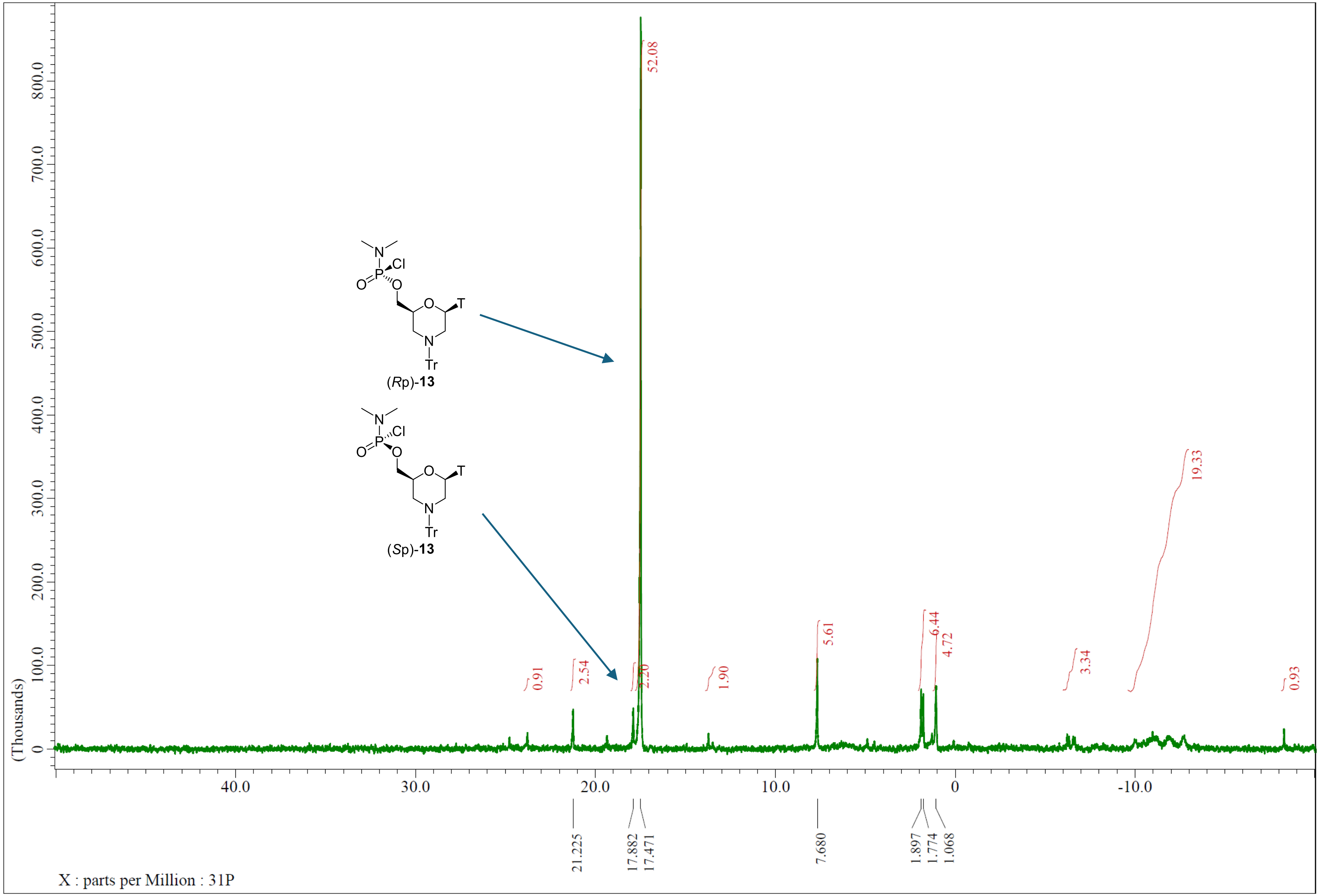

Figure S17. ^{31}P NMR spectrum of the reaction mixture of Table 3, Entry 5

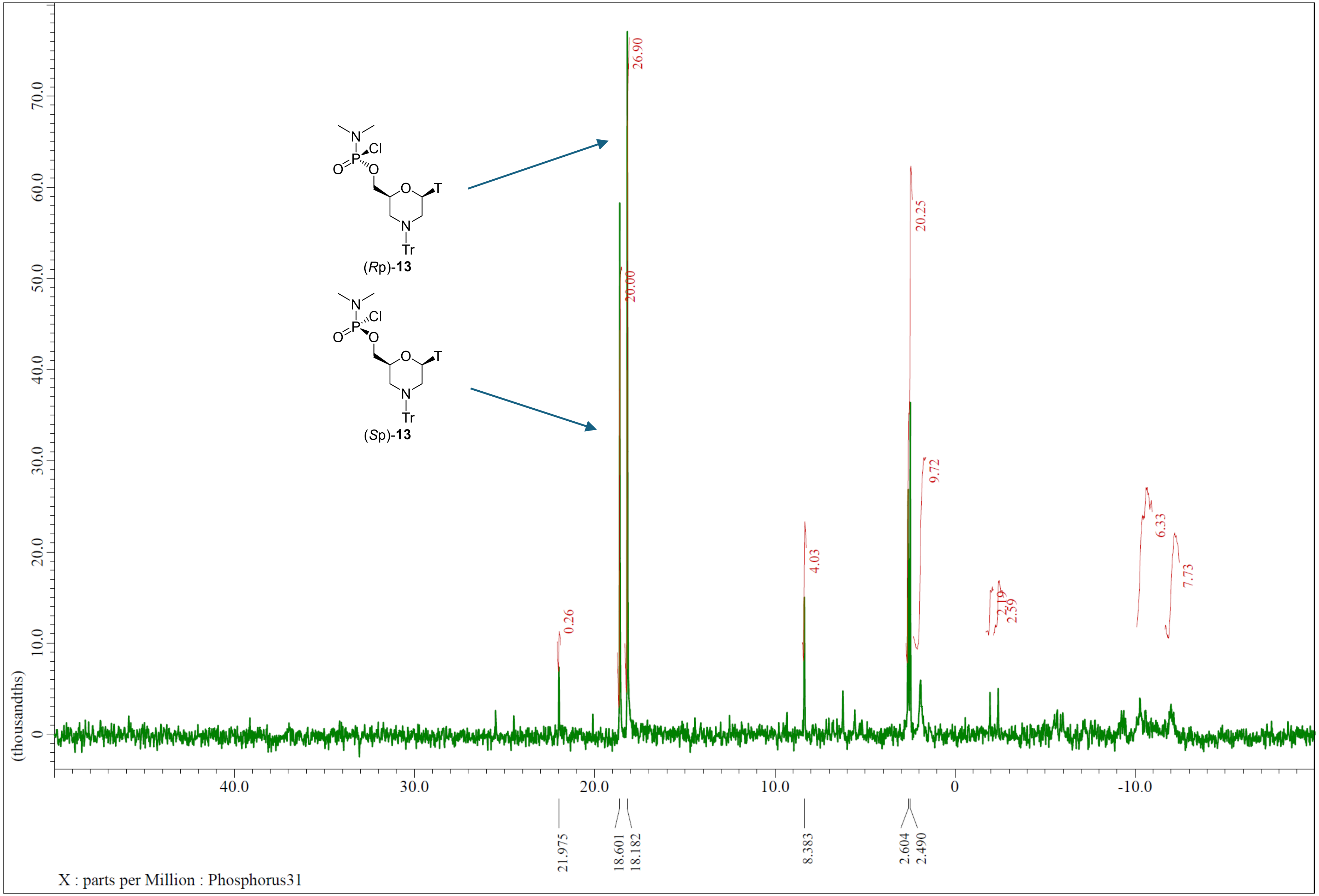

Figure S18. ^{31}**P NMR spectrum of the reaction mixture of Table 3, Entry 6**

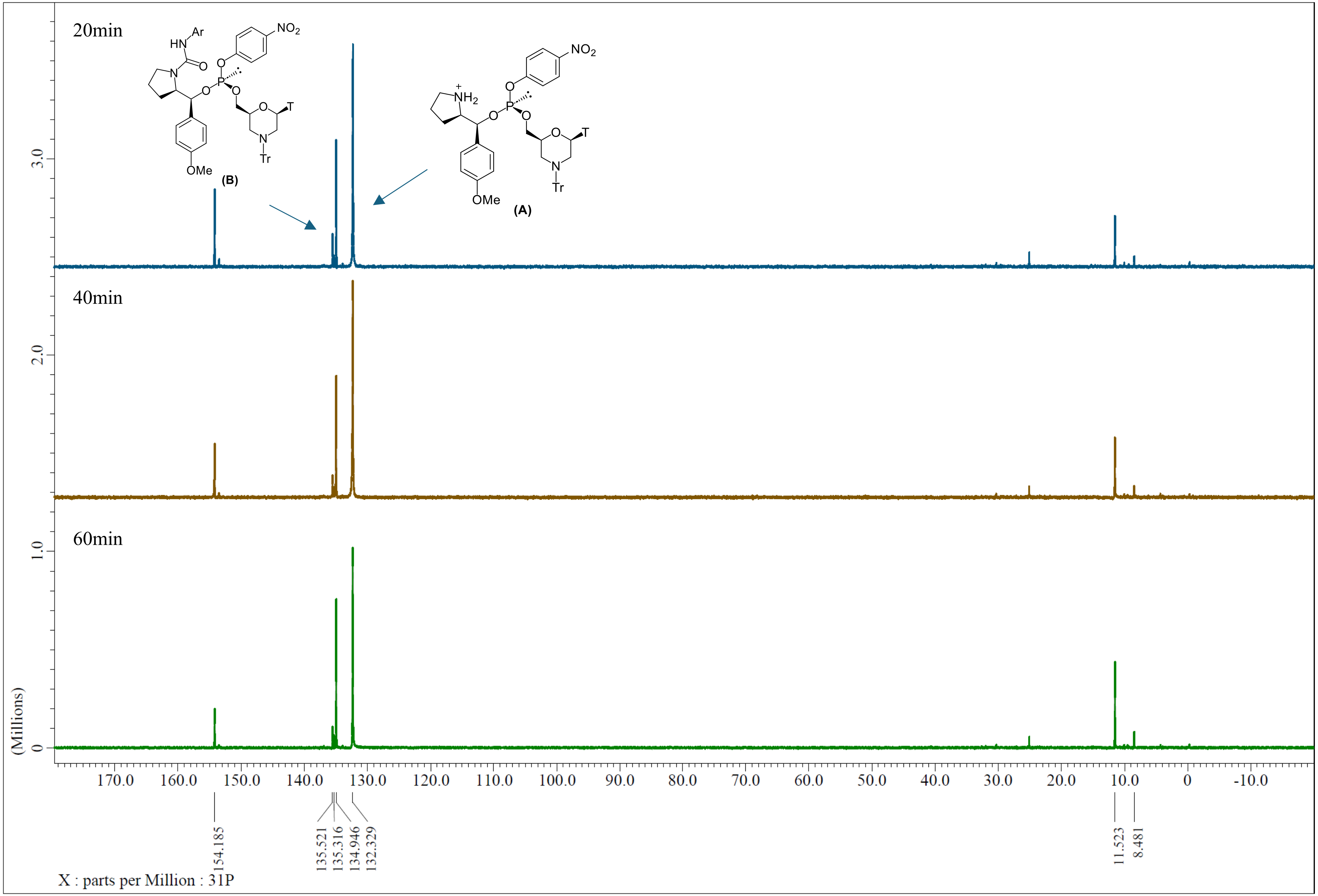

Figure S19. ^{31}P NMR spectra of the reaction mixture of Figure 3 (PhNCO, 20 min, 40 min, 60 min)

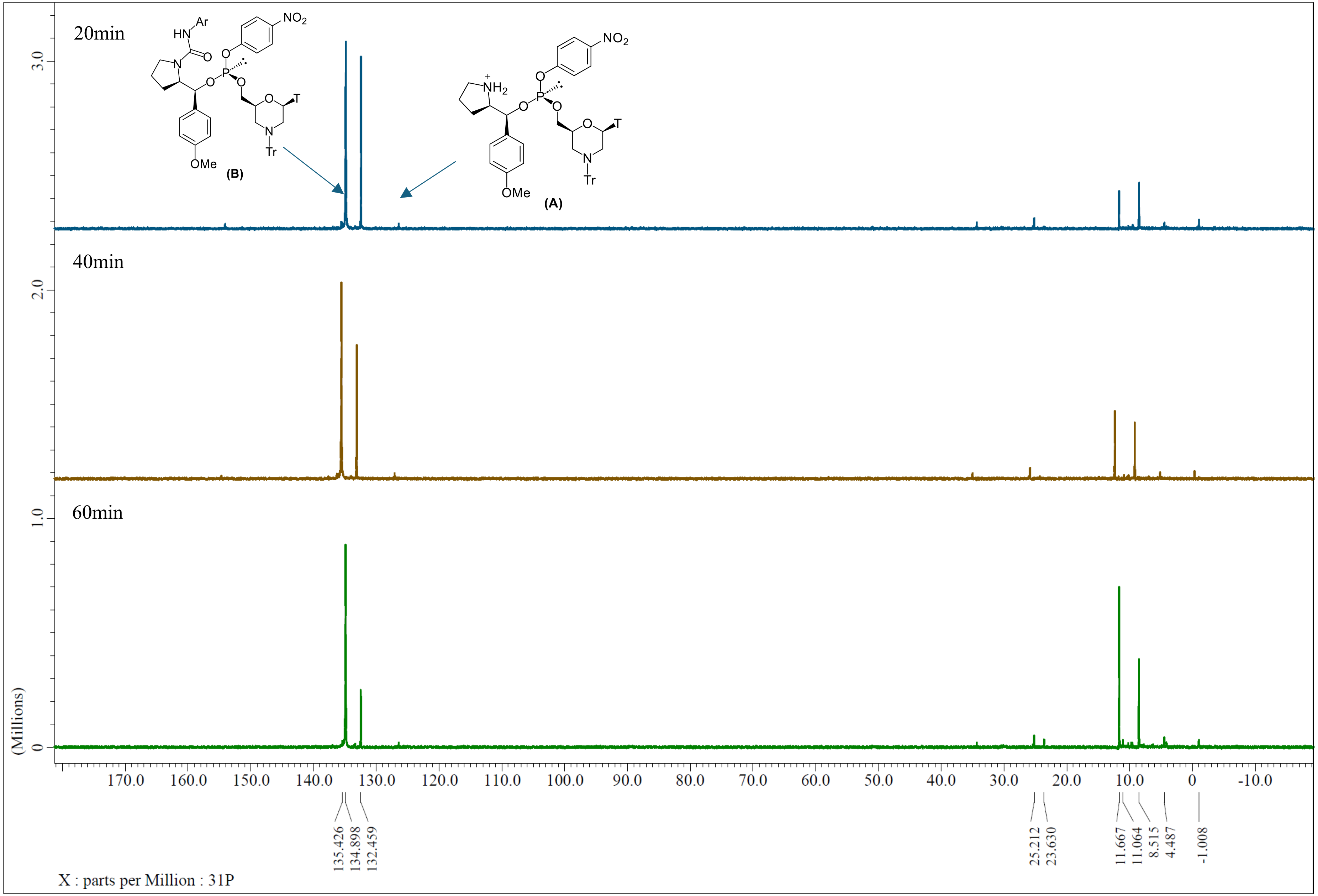

Figure S20. ^{31}**P NMR spectra of the reaction mixture of figure 3 (4-nitrophenyl isocyanate, 20 min, 40 min, 60 min)**

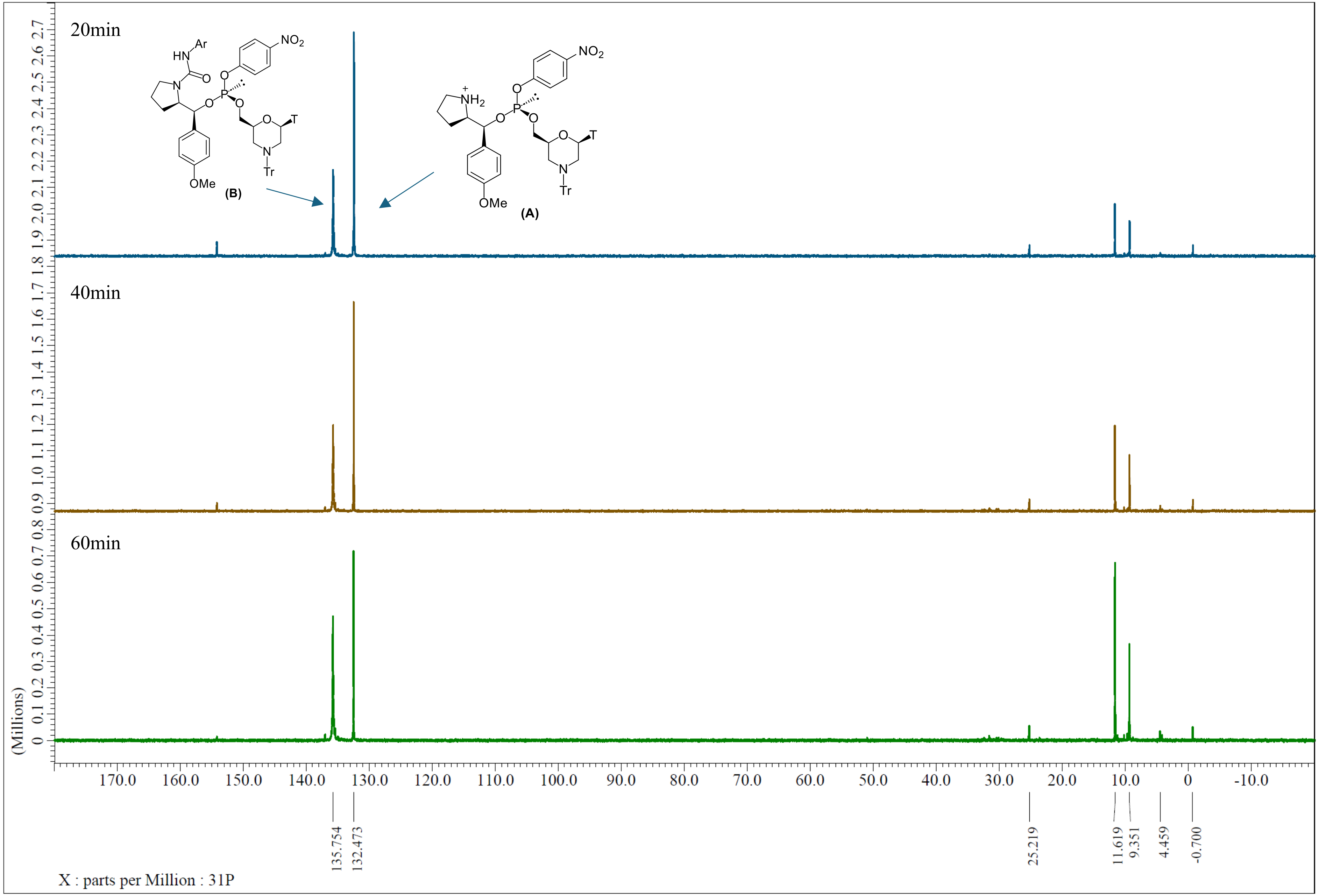

Figure S21. ^{31}**P NMR spectra of the reaction mixture of figure 3 (2,4,6-trichlorophenyl isocyanate, 20 min, 40 min, 60 min)**

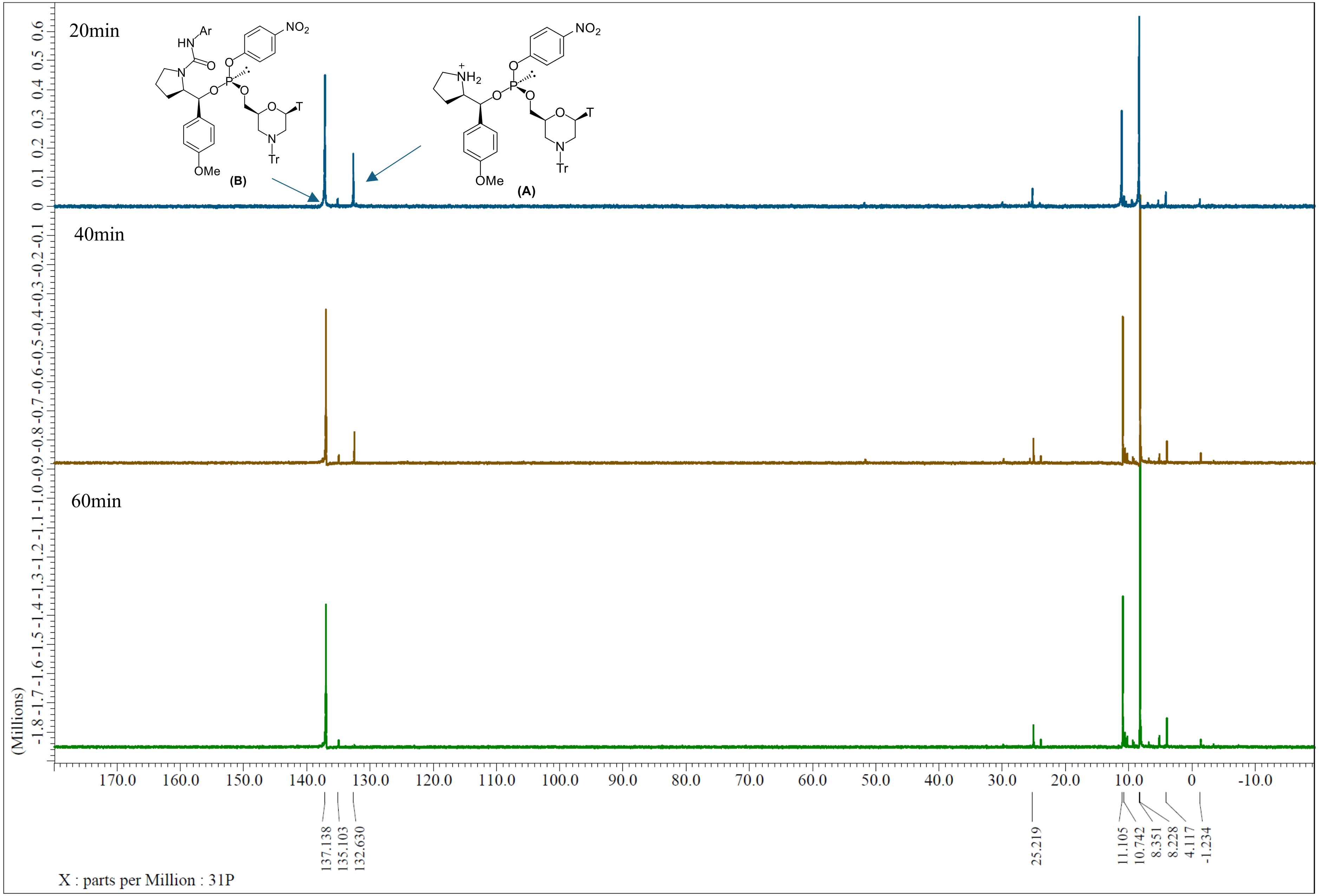

Figure S22. ^{31}P NMR spectra of the reaction mixture of figure 3 (3,4-dichlorophenyl isocyanate, 20 min, 40 min, 60 min)

3.4 Elucidation of the mechanism for removal of the chiral auxiliary

To identify the byproduct from the chiral auxiliary, we synthesized cyclized compound **S10** under the conditions shown in **Scheme S8**. The structure of **S10** was determined by ^{1}H NMR, ^{13}C NMR, COSY, HMBC, and ESI-MS (**Figure S23–S27**). In the ^{1}H NMR spectrum of the crude mixture of chloridate monomer **(*R*p)-13**, which was synthesized under the optimal conditions. We observed two signals at δ 5.36 (d, *J* = 9.0 Hz) and δ 4.97 (d, *J* = 2.6 Hz) in the crude mixture. These signals were consistent with the signals of the benzyl position of two diastereomers of compound **S10** (δ 5.35 (d, *J* = 8.9 Hz) and δ 4.94 (d, *J* = 2.7 Hz)). The ^{1}H NMR spectra indicated signals corresponding to a diastereomer mixture of **S10** (**Figure S28 and S29**). The result suggested that removal of the chiral auxiliary procced via the formation of a benzyl cation, followed by the formation of diastereomer mixtures of compound **S10** through nucleophilic attack of the carbonyl group of the urea moiety to the benzyl cation (**Scheme S9**).

Scheme S8. Synthesis of compound S10

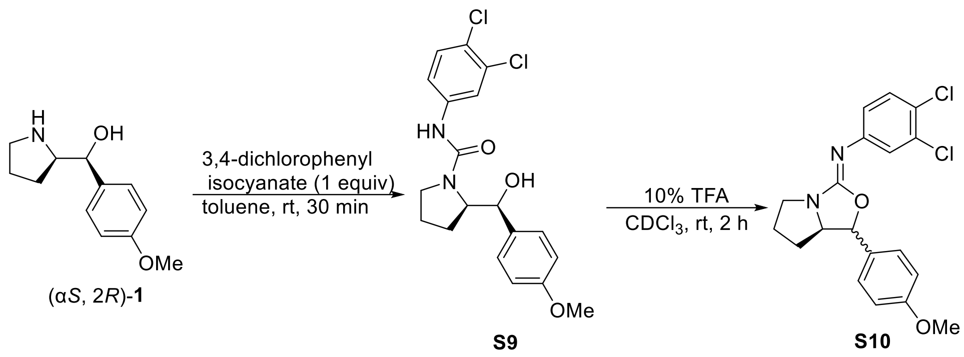

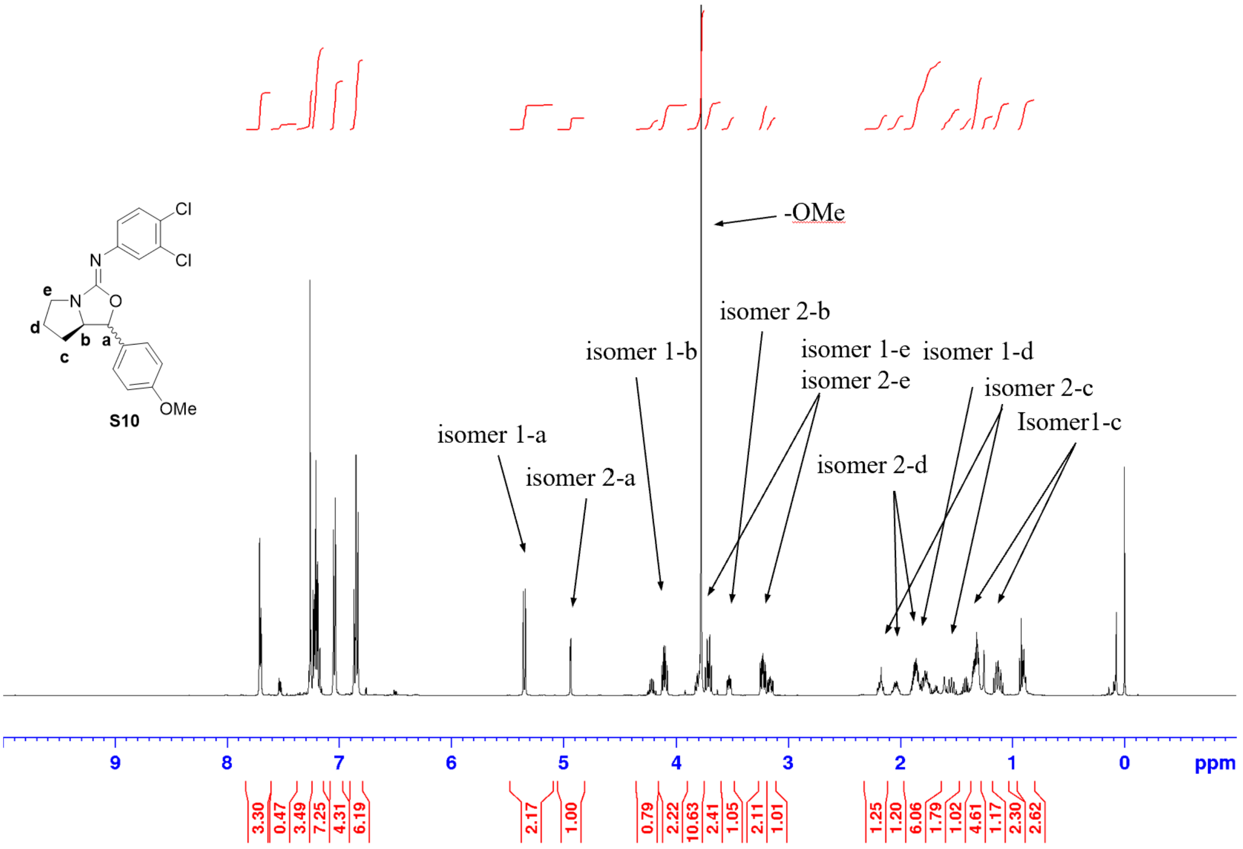

Figure S23. ^{1}H NMR spectrum of S10

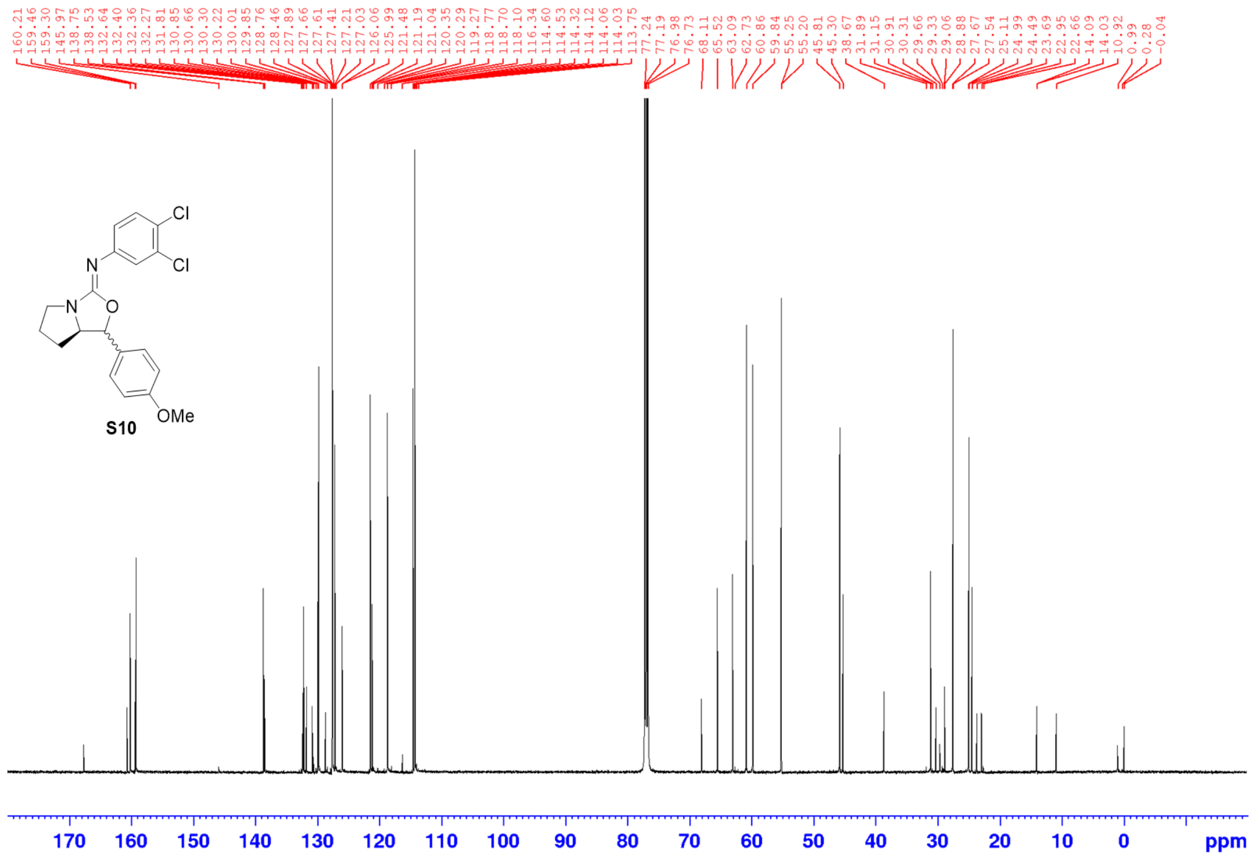

Figure S 24. ^{13}C NMR spectrum of S10

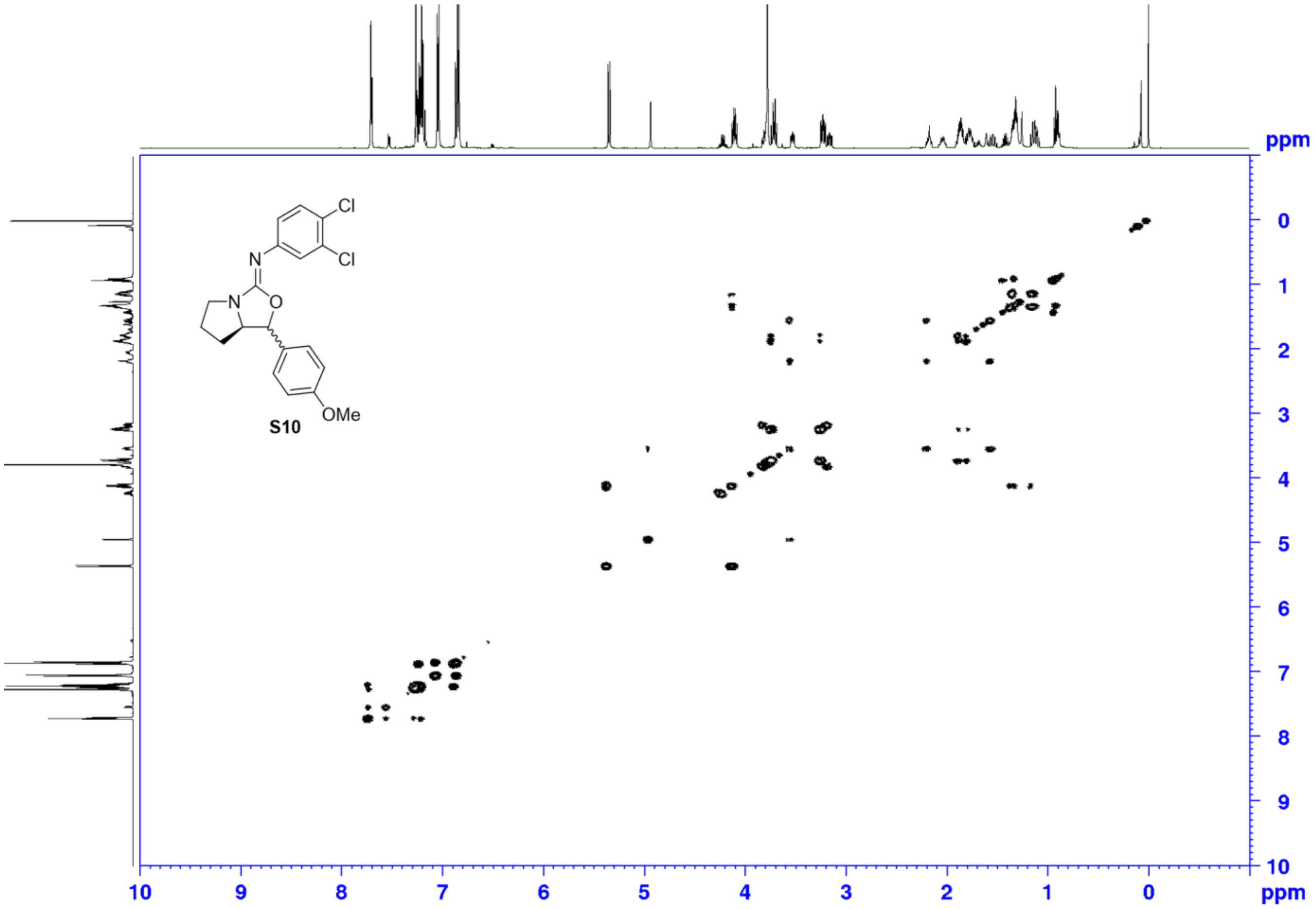

Figure S 25. COSY of S10

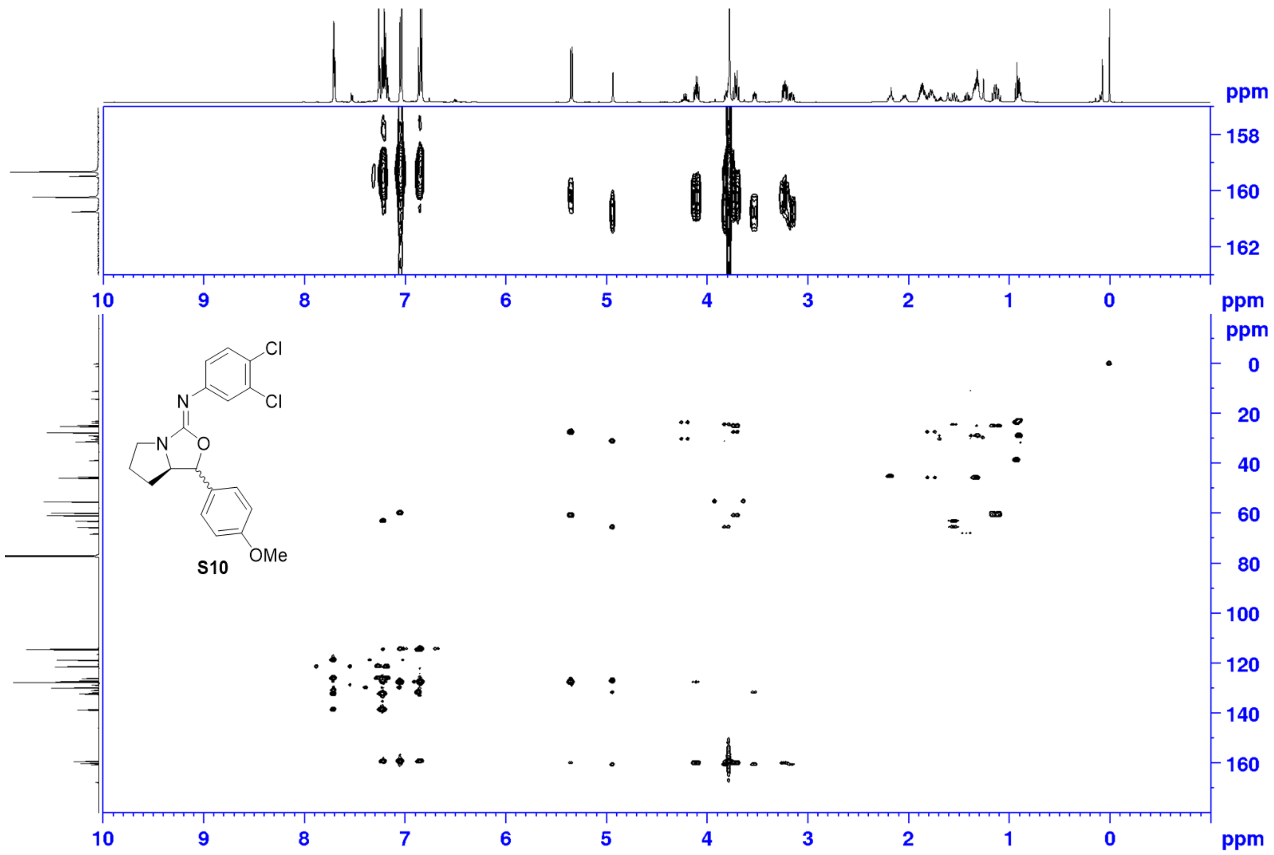

Figure S 26. HMBC of S10

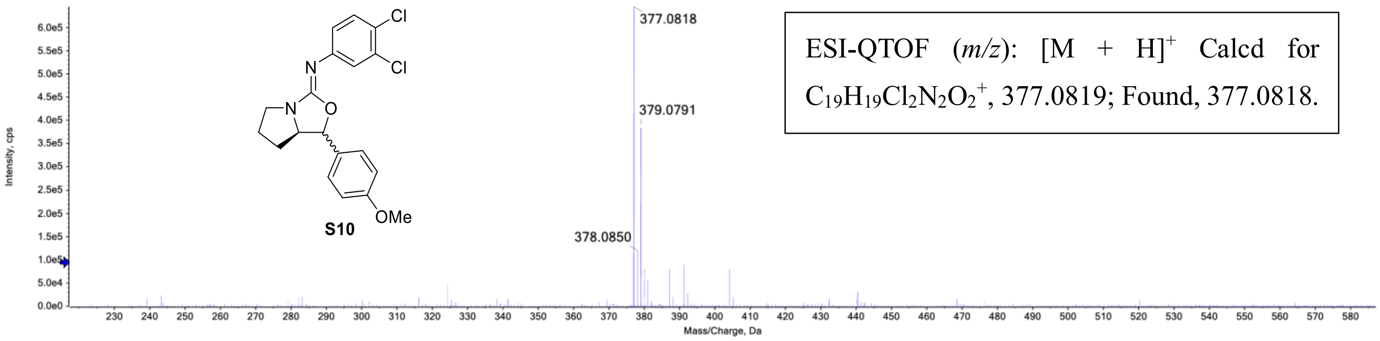

Figure S27. Mass spectra of S10

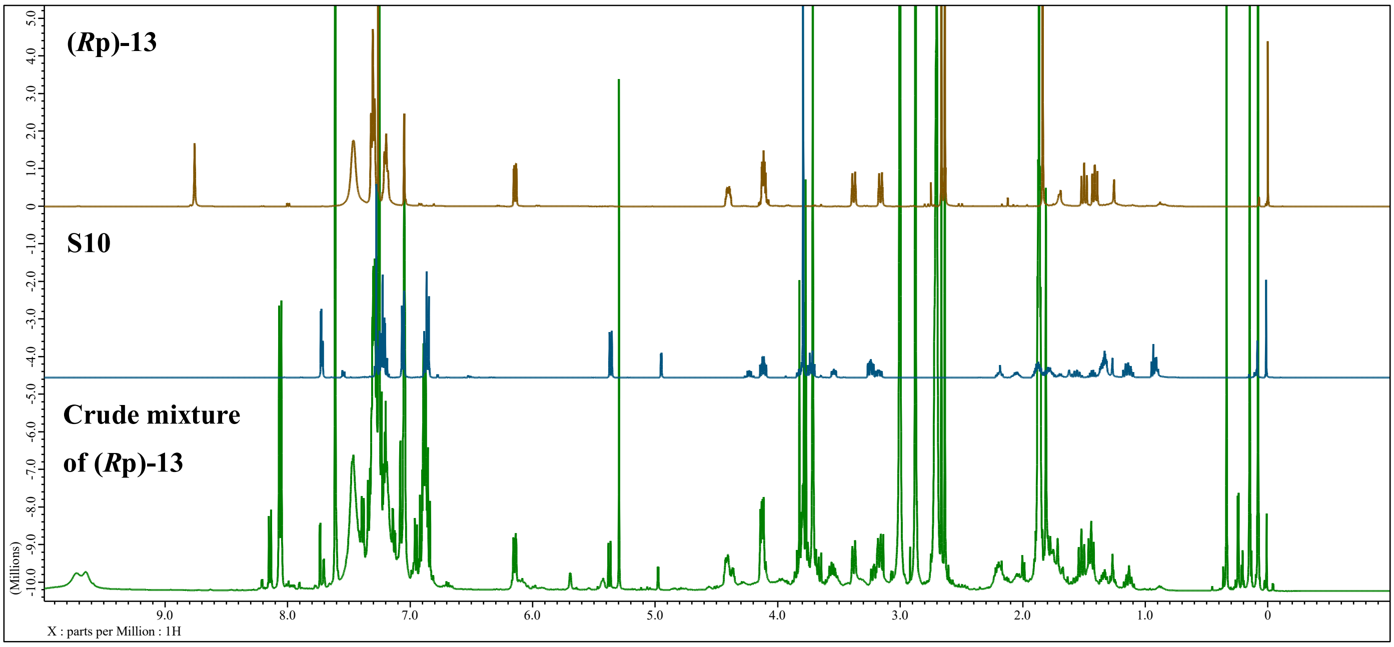

Figure S 28. [1]**H NMR spectra of crude mixture of (*R*p)-13**

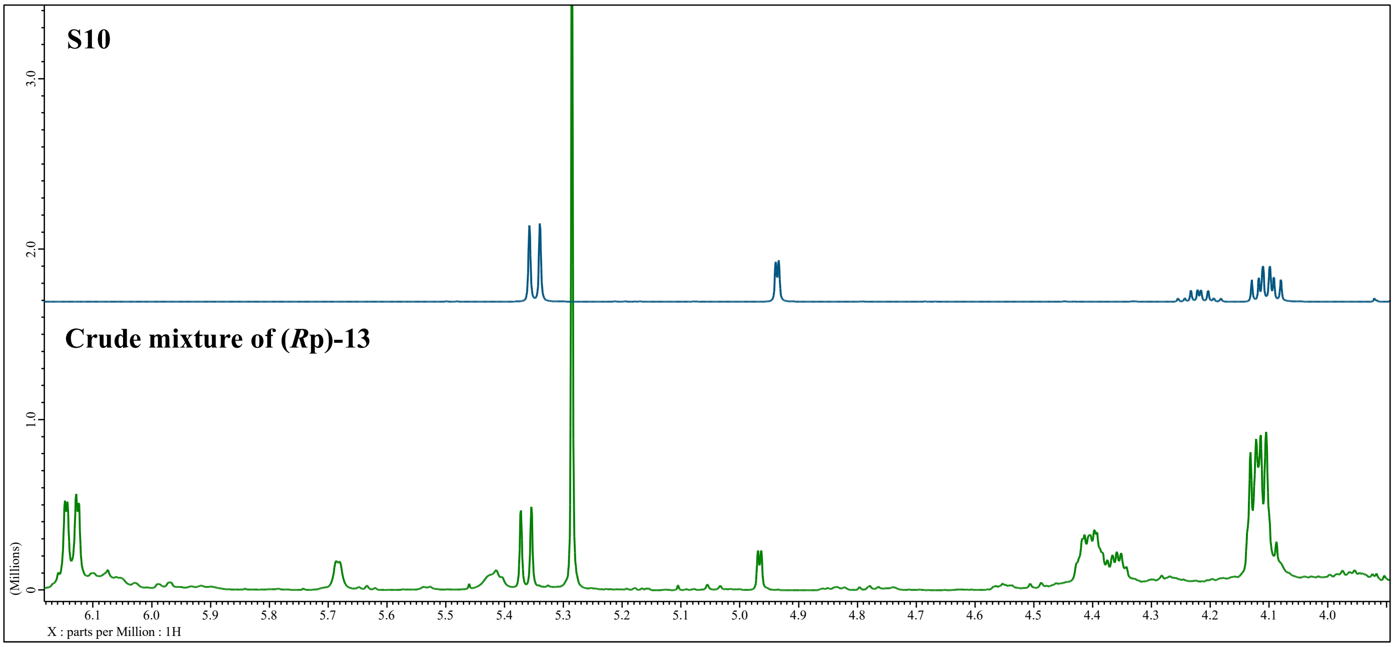

Figure S 29. [1]**H NMR spectra of S10 (blue) and crude mixture of (*R*p)-13 (green)**

Scheme S9. Plausible mechanism for removal of the chiral auxiliary

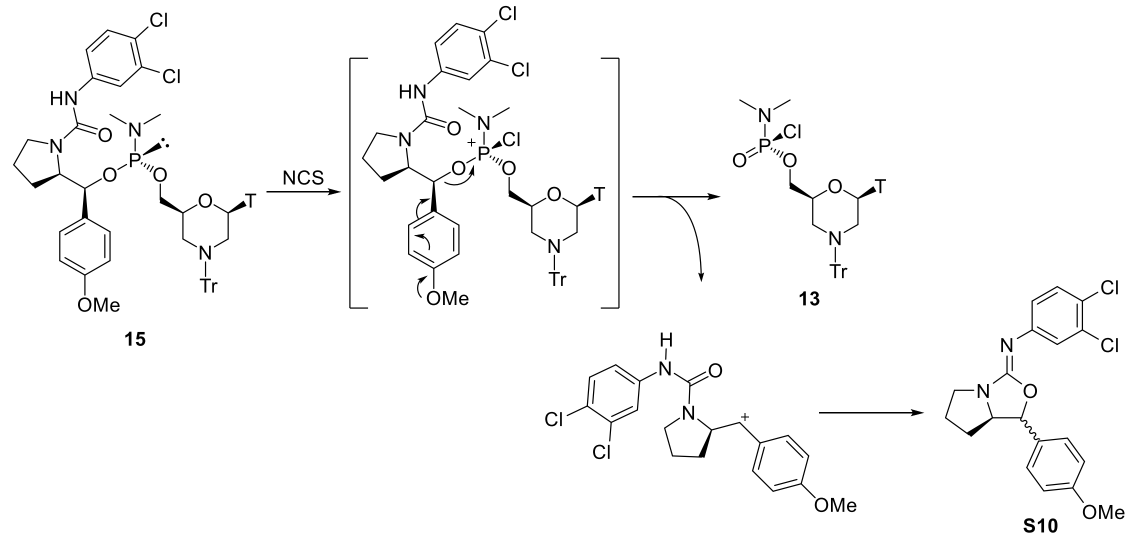

3.5 NOESY and ROESY experiments of $N_{PN}T$ dimers

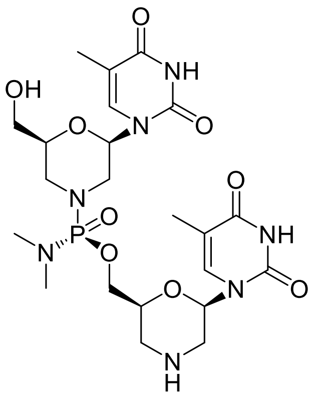

(*S*p)-**27**

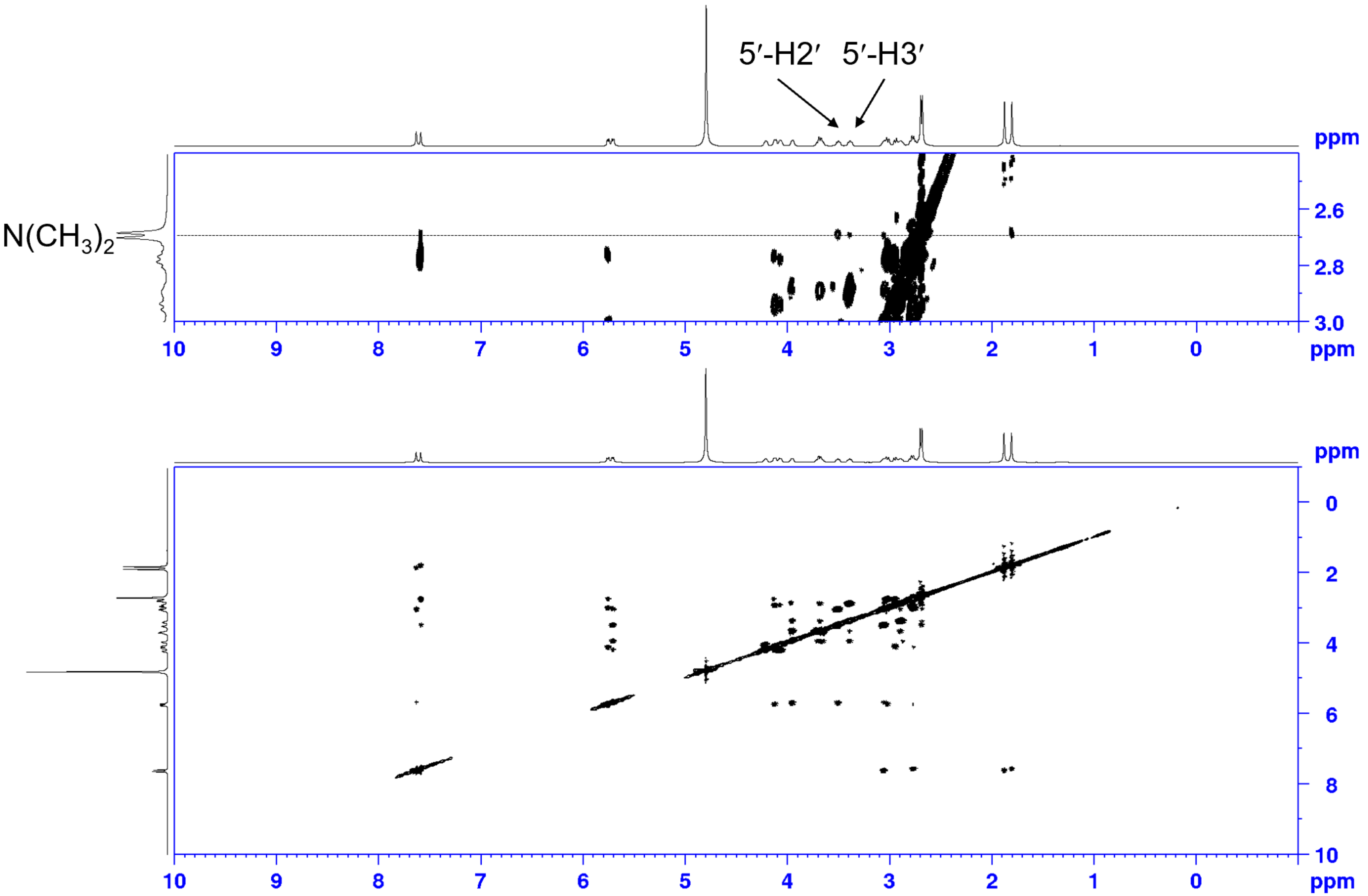

Figure S 30. NOESY spectrum of *S*p-TT dimer (*S*p)-**27**

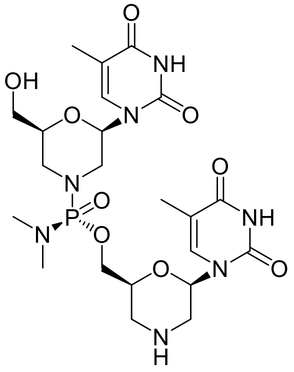

(*R*p)-**27**

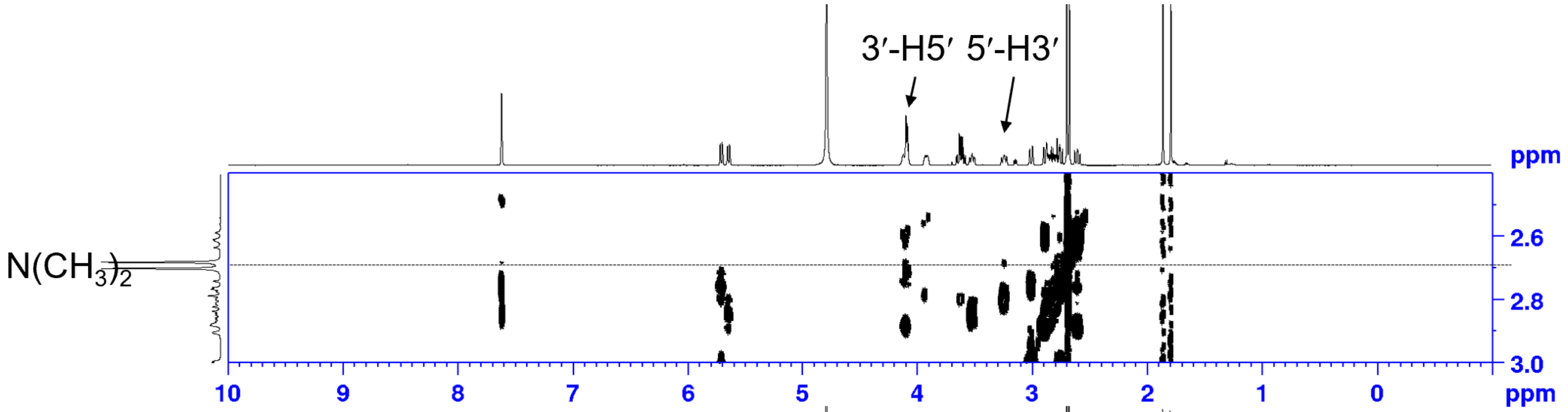

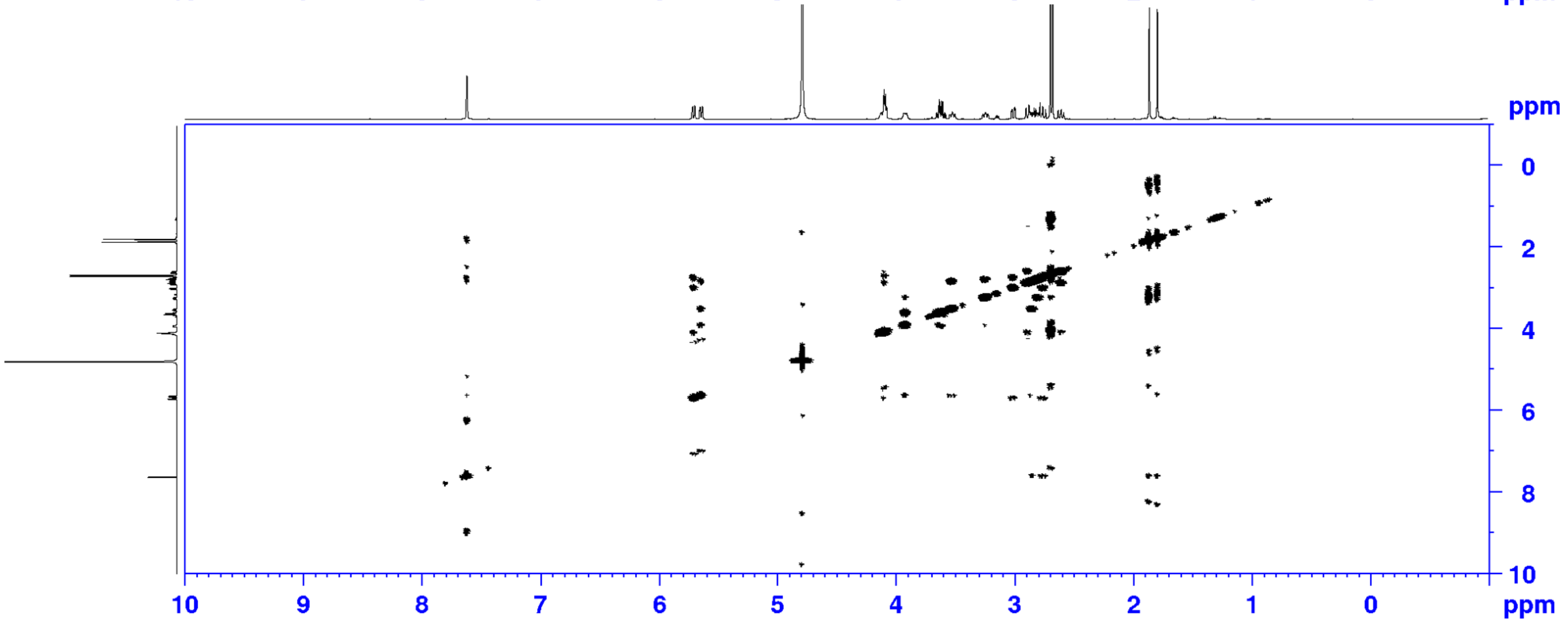

Figure S 31. NOESY spectrum of *R*p-TT dimer (*R*p)-**27**

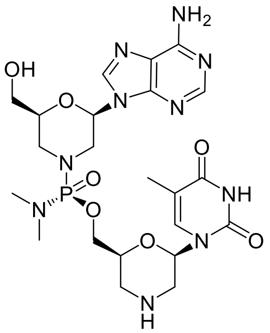

(*S*p)-**28**

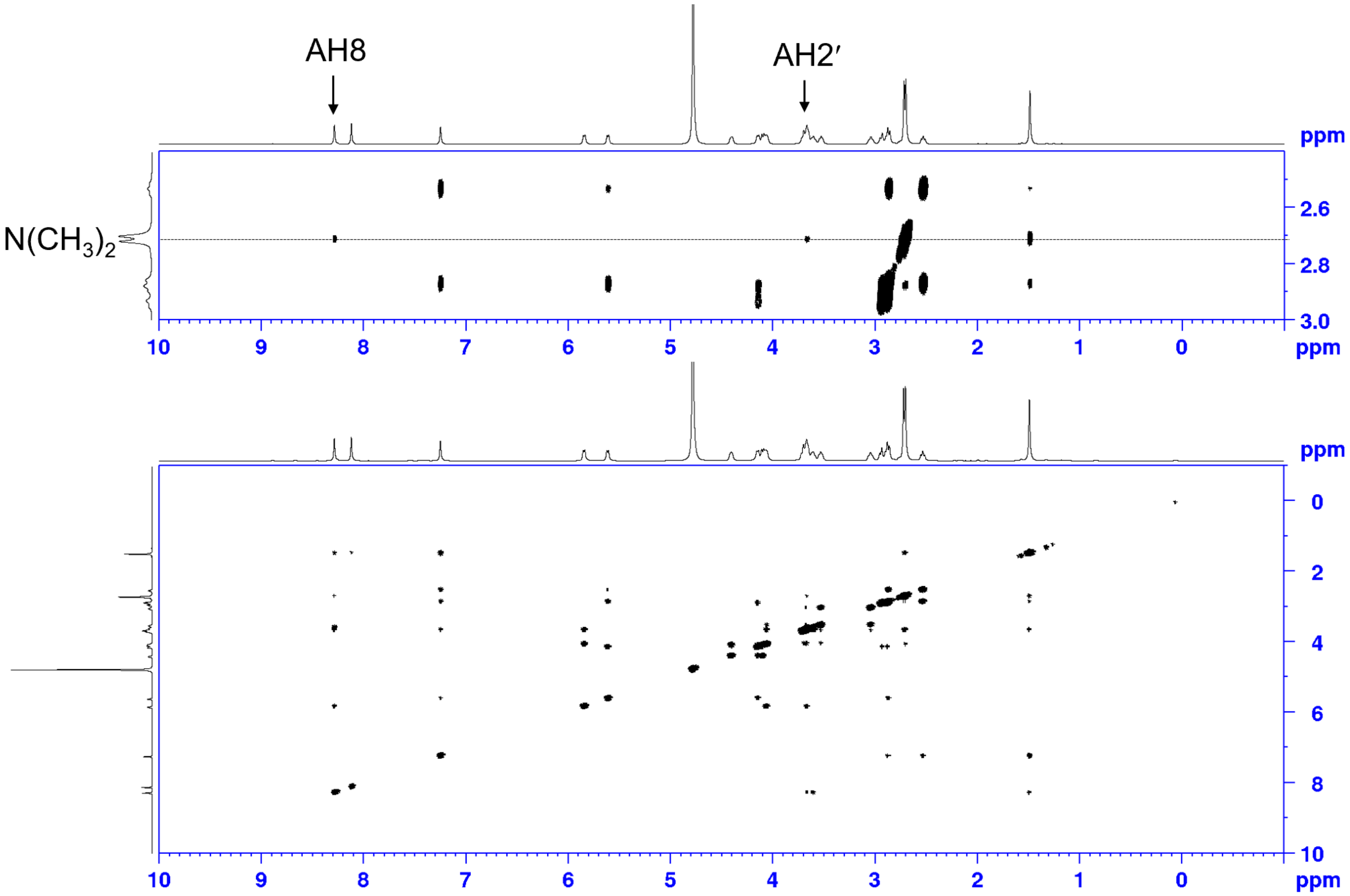

Figure S 32. NOESY spectrum of *S*p-AT dimer (*S*p)-**28**

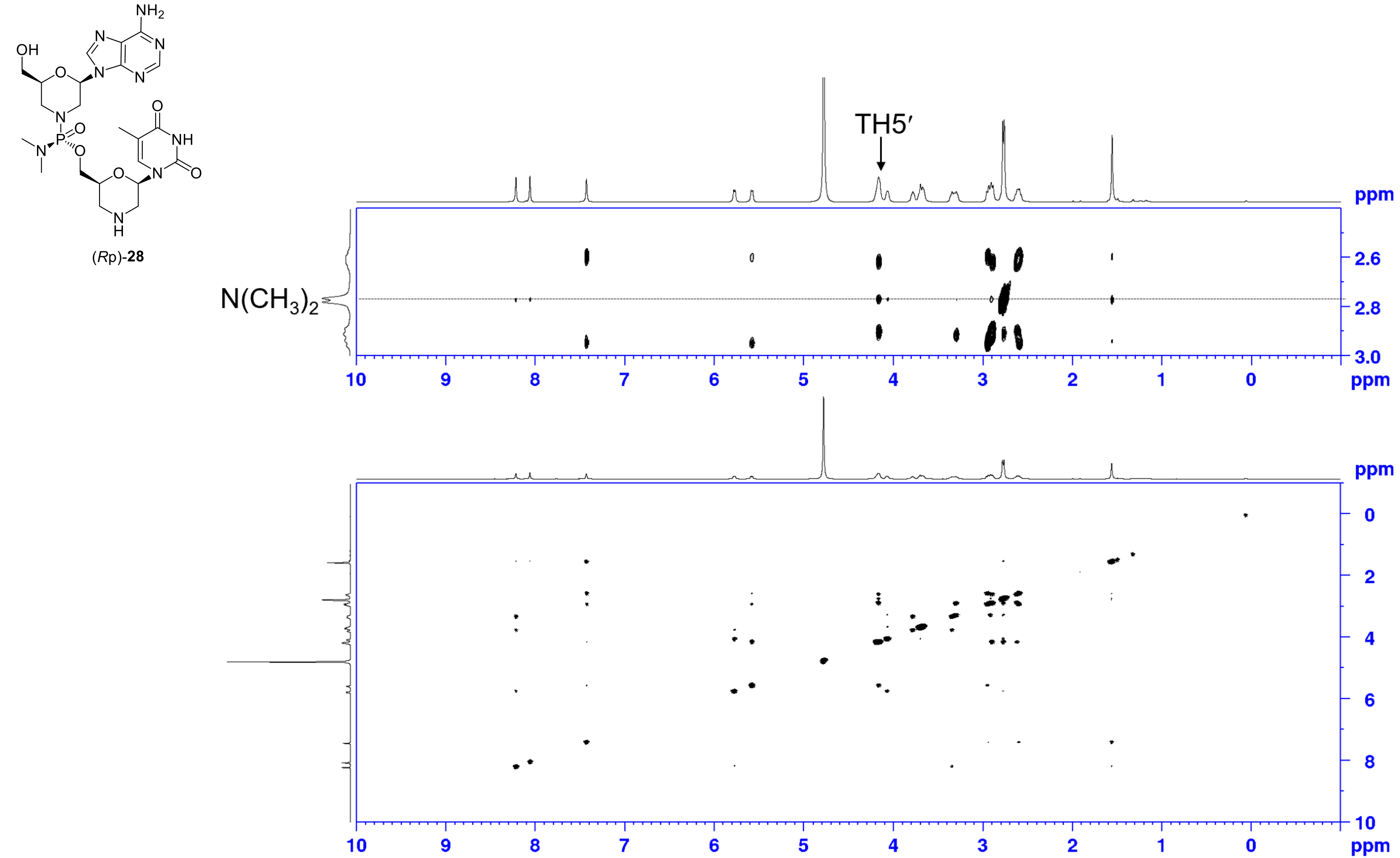

Figure S 33. NOESY spectrum of *R*p-AT dimer (*R*p)-**28**

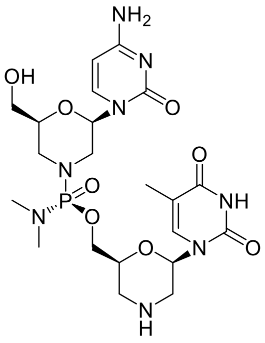

(*S*p)-**29**

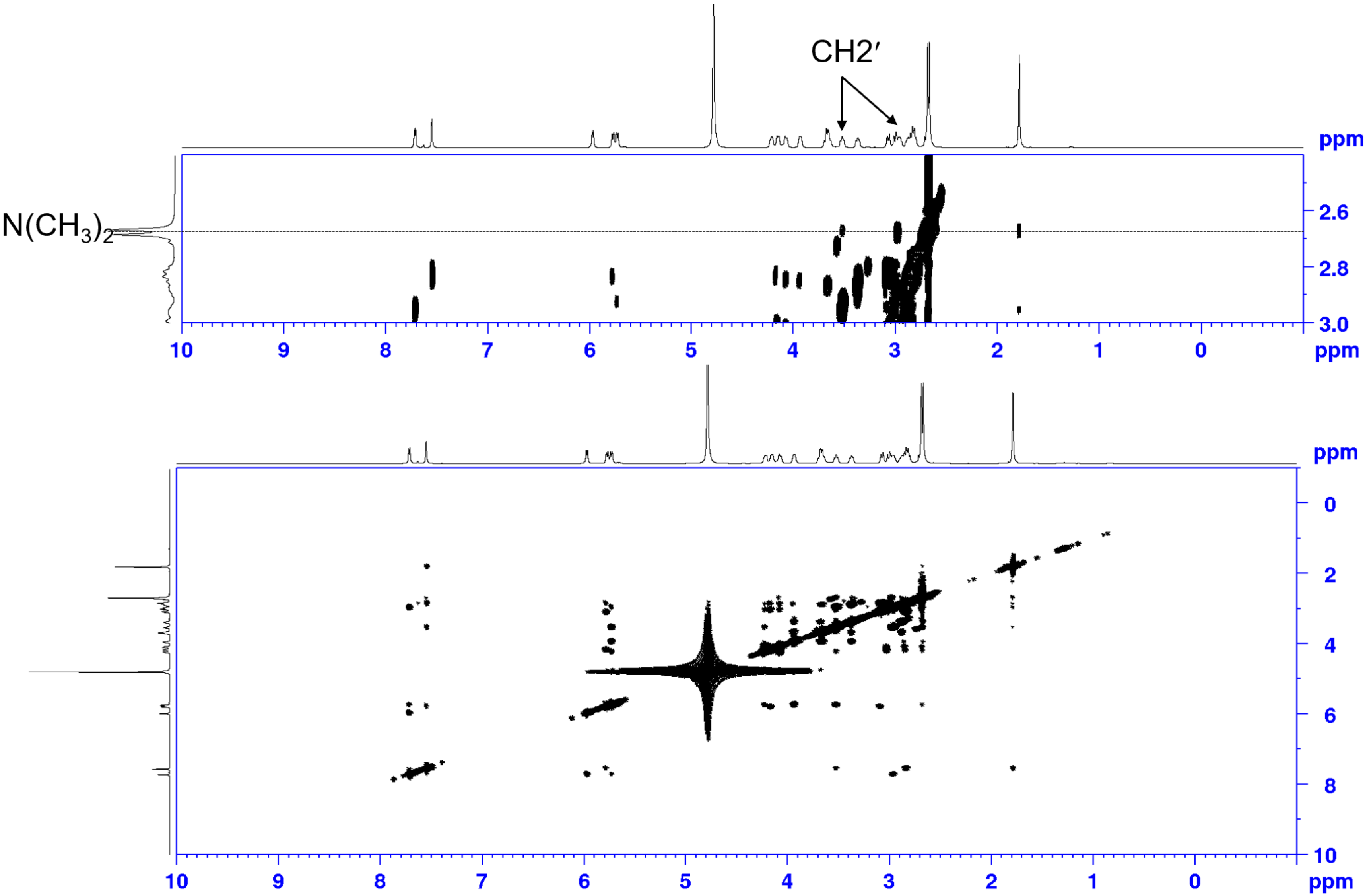

Figure S 34. ROESY spectrum of *S*p-CT dimer (*S*p)-**29**

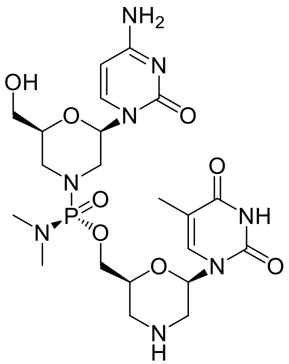

(*R*p)-**29**

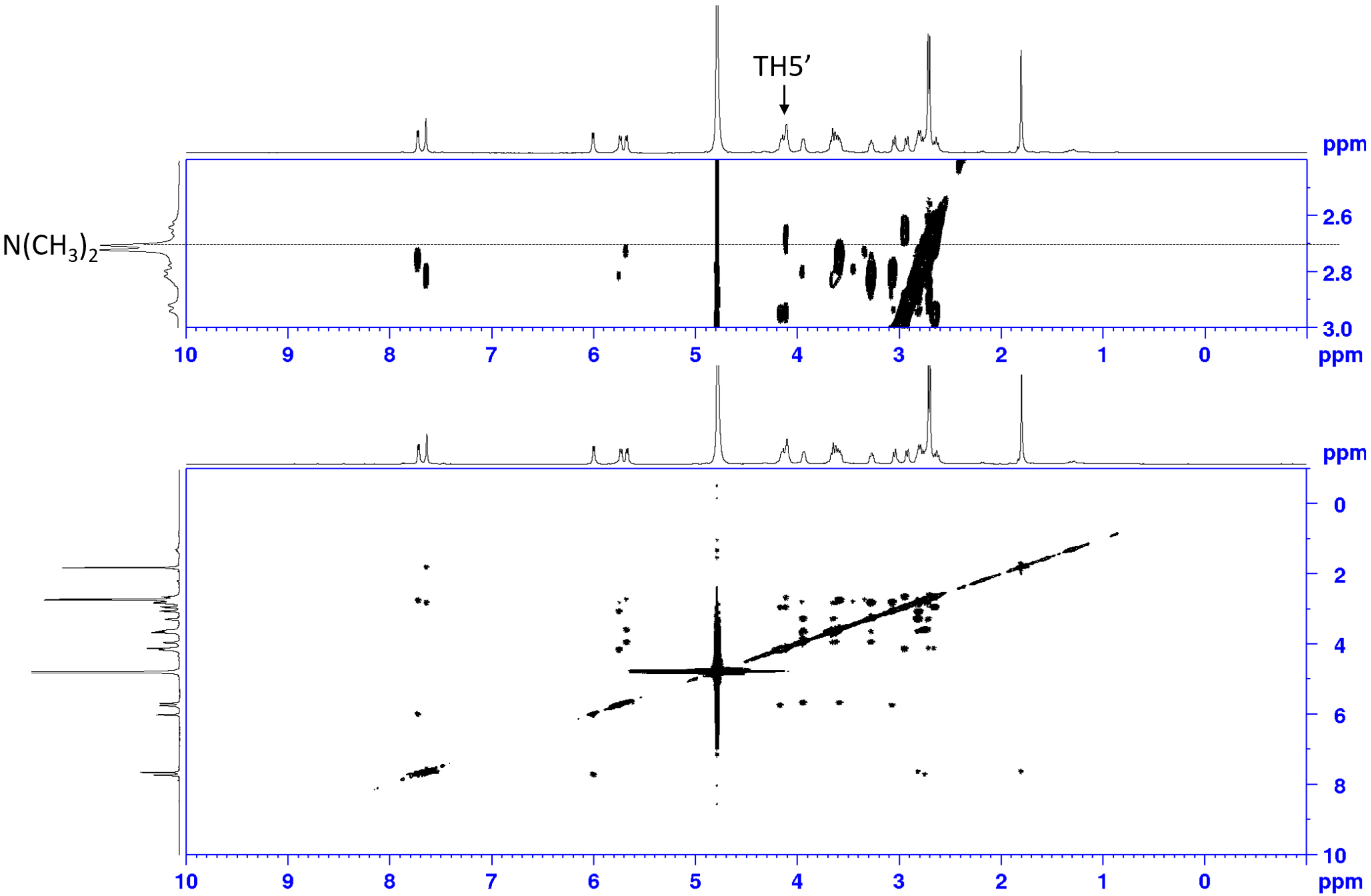

Figure S 35. ROESY spectrum of *R*p-CT dimer (*R*p)-**29**

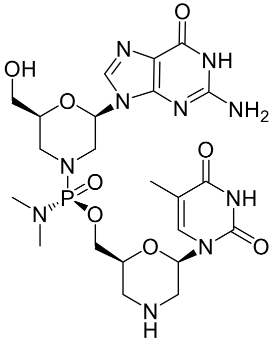

(*S*p)-**30**

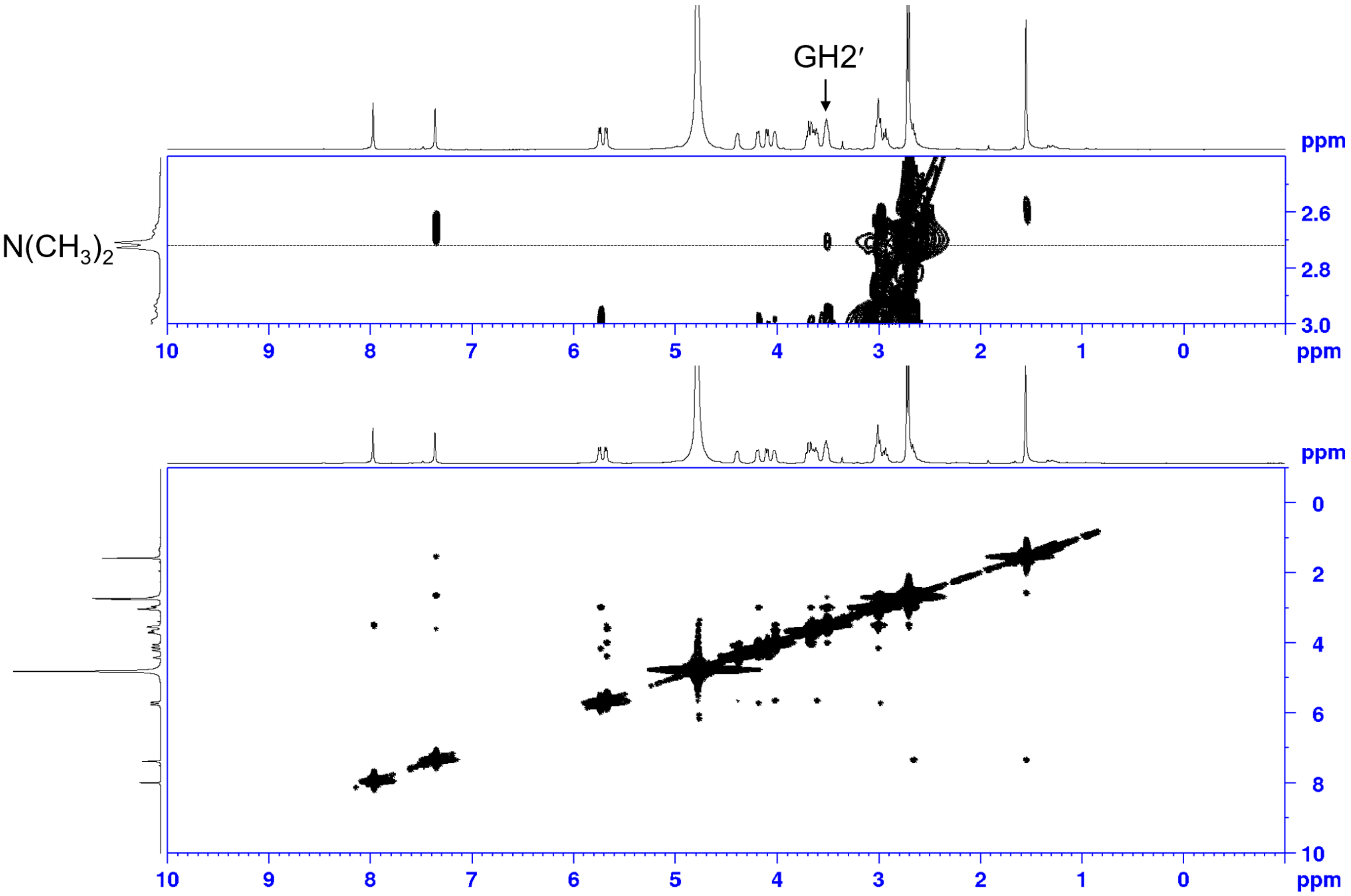

Figure S 36. NOESY spectrum of *S*p-GT dimer (*S*p)-**30**

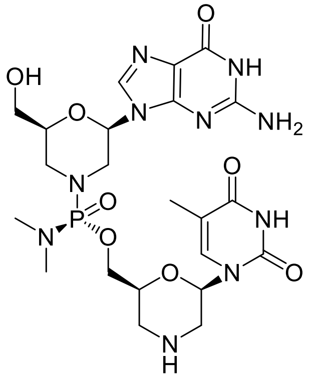

(*R*p)-**30**

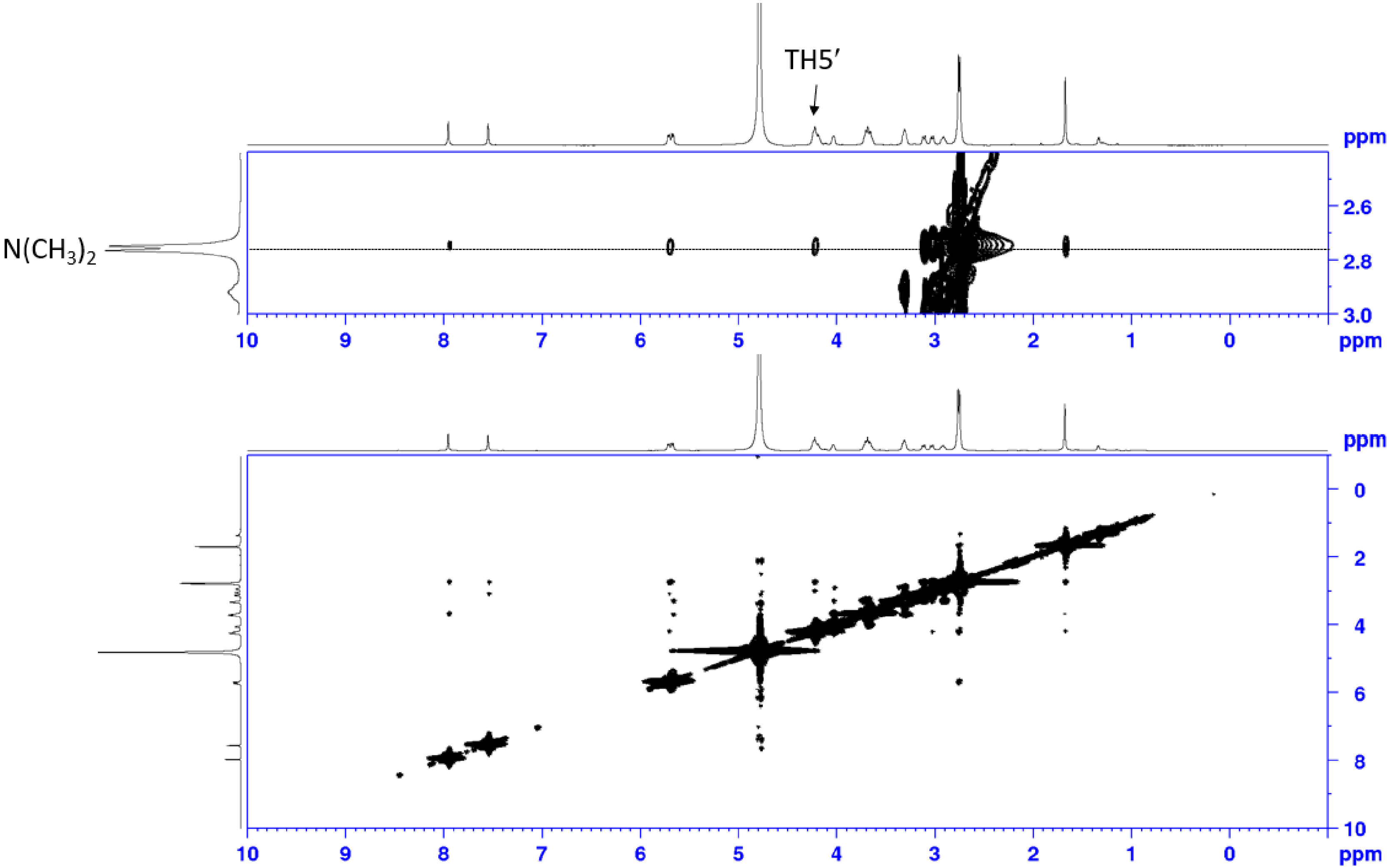

Figure S 37. NOESY spectrum of *R*p-GT dimer (*R*p)-**30**

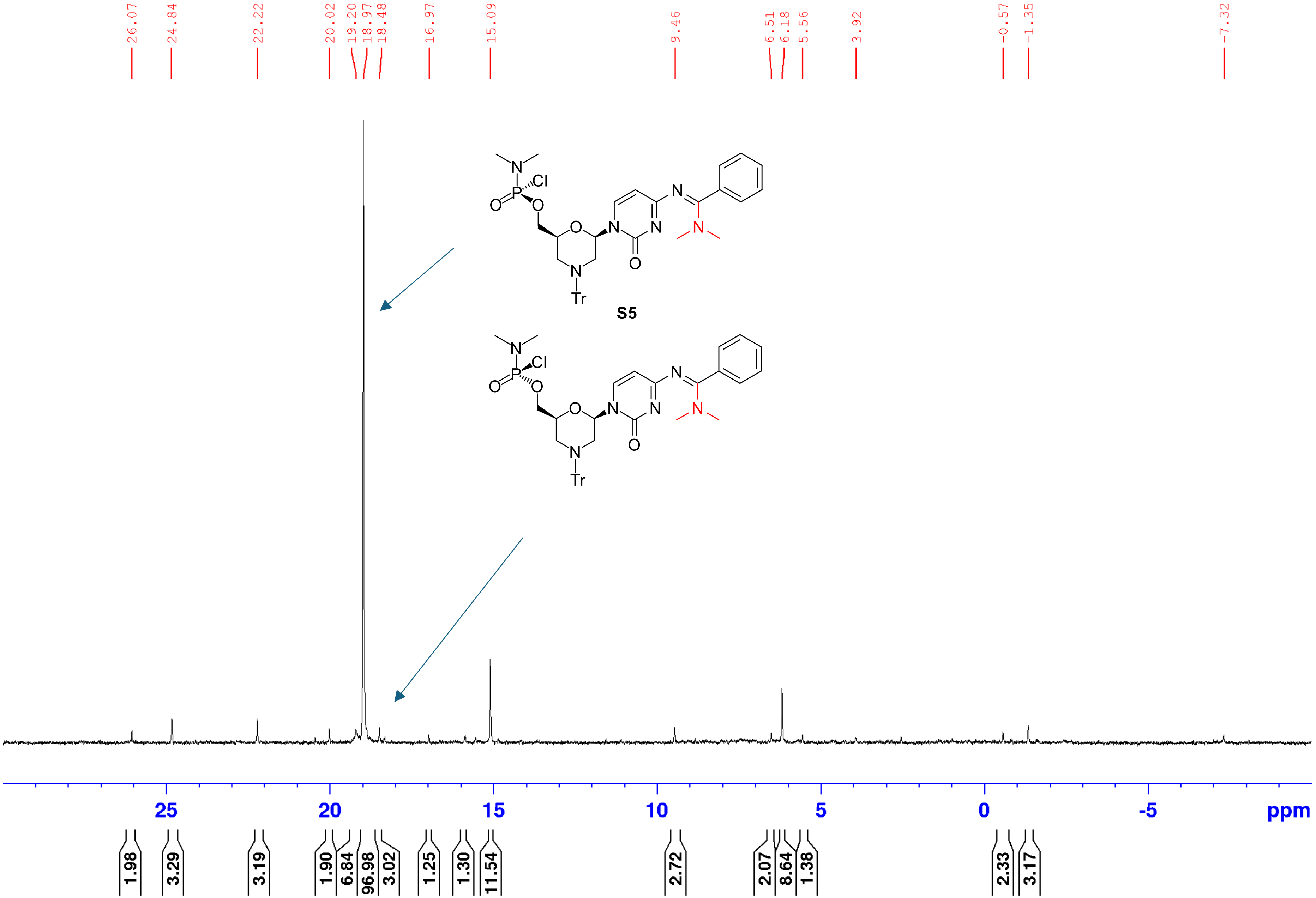

Figure S 38. **^{31}P NMR spectrum of the reaction mixture of Table S1, Entry 1**

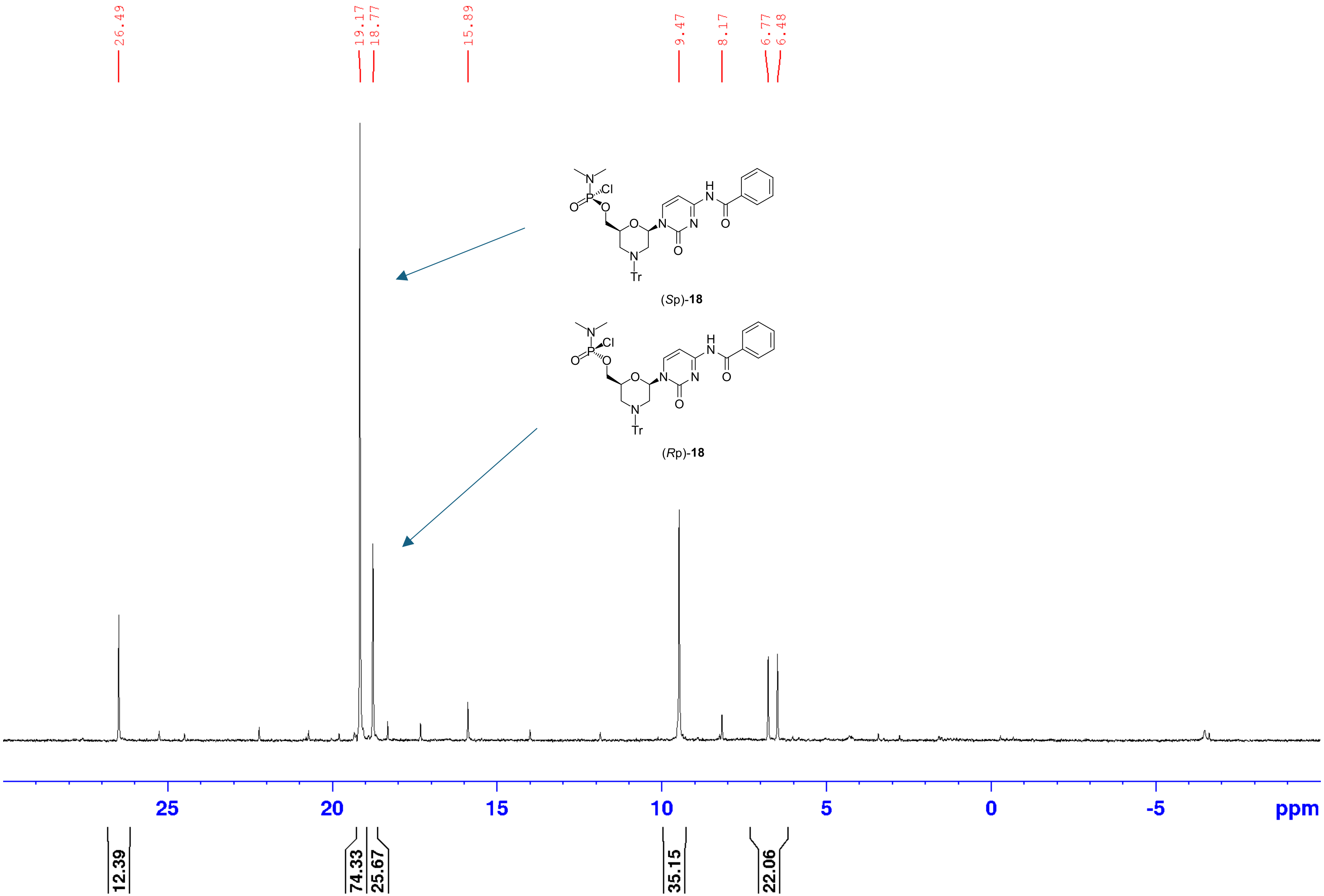

Figure S 39. ^{31}P NMR spectrum of the reaction mixture of Table S1, Entry 2

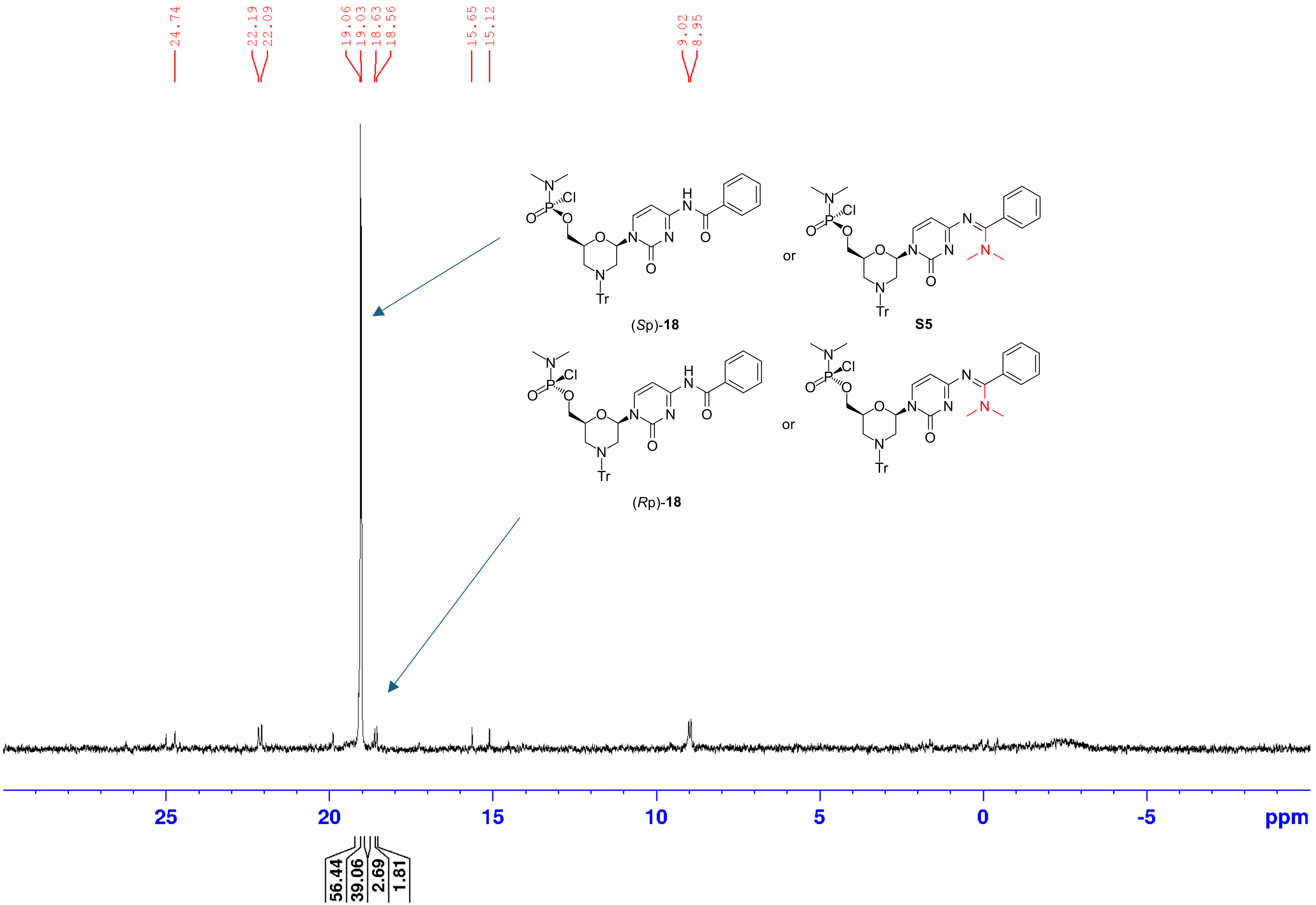

Figure S 40. **^{31}P NMR spectrum of the reaction mixture of Table S2, Entry 1**

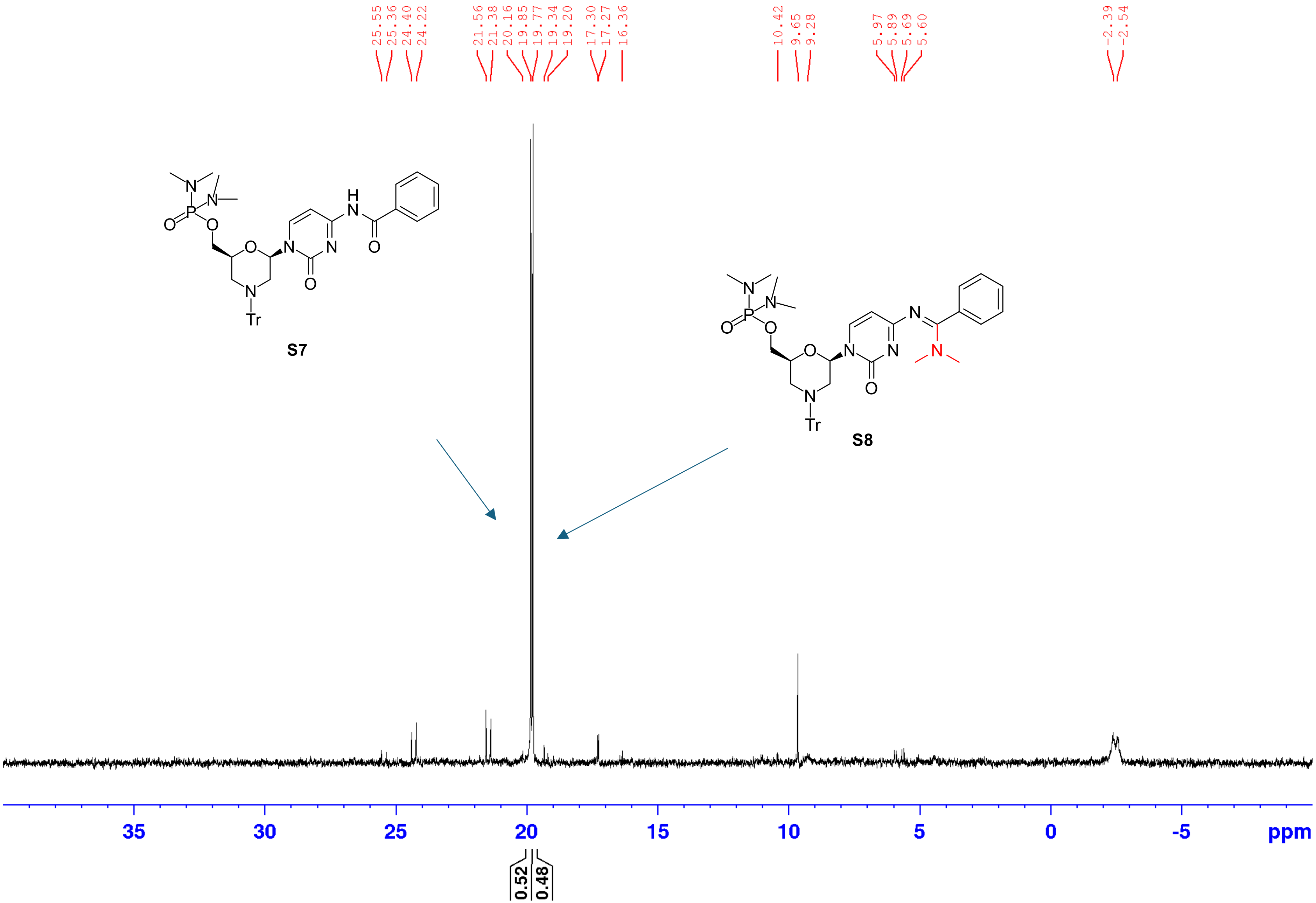

Figure S 41. ^{31}**P NMR spectrum after the condensation with chloridate and dimethylamine of Table S2, Entry 1**

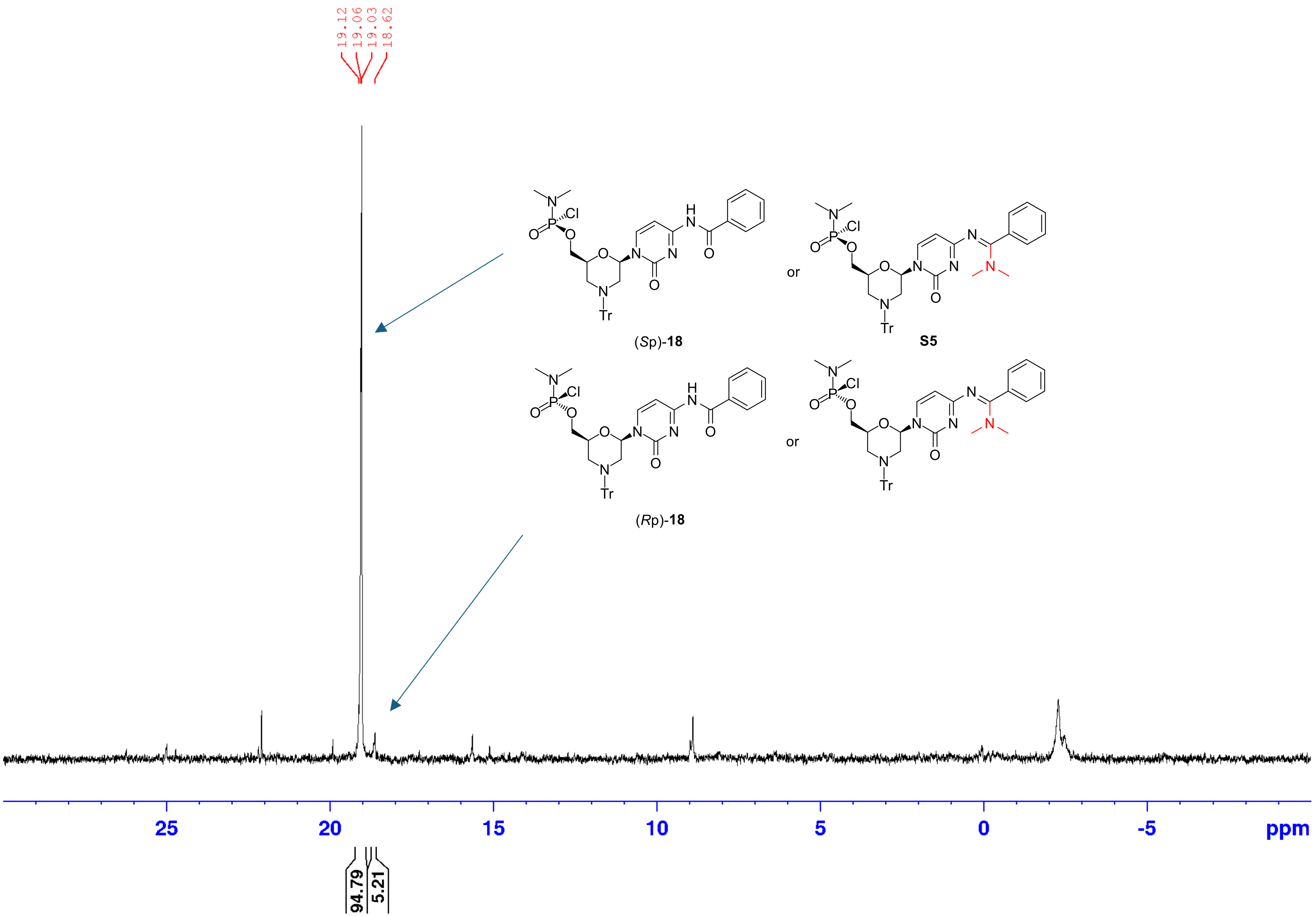

Figure S 42. **^{31}P NMR spectrum of the reaction mixture of Table S2, Entry 2**

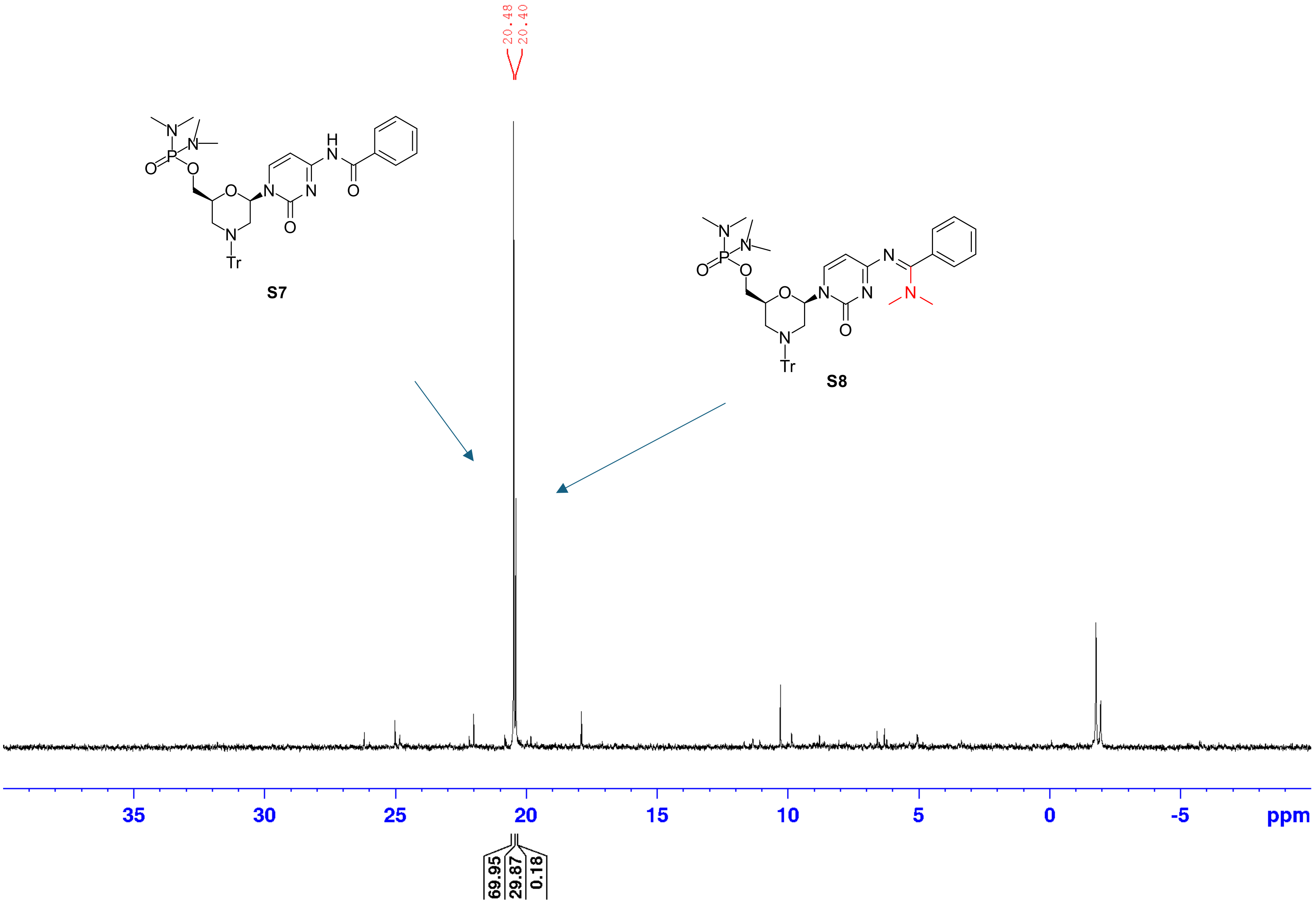

Figure S 43. ^{31}P NMR spectrum after the condensation with chloridate and dimethylamine of Table S2, Entry 2

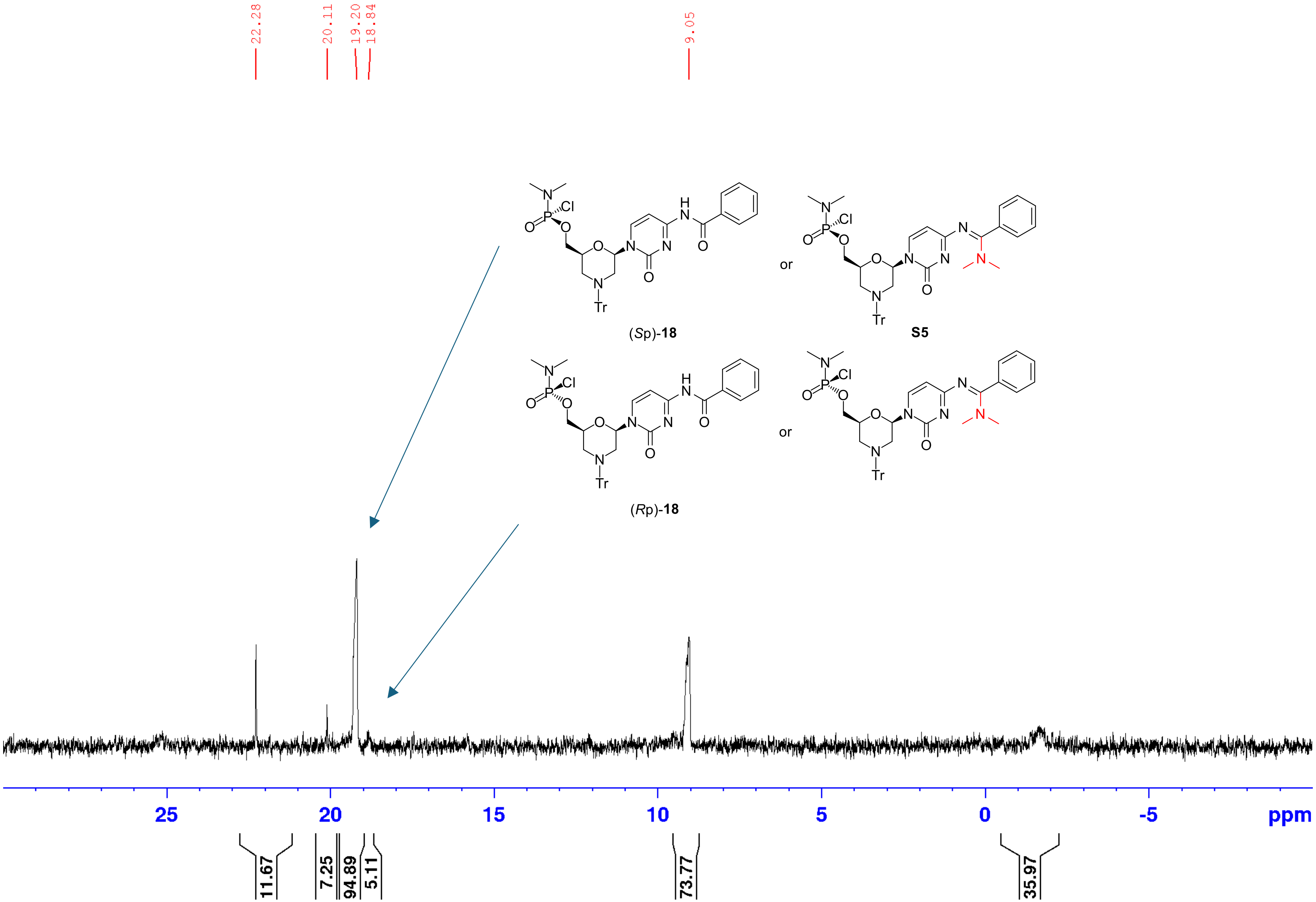

Figure S 44. ^{31}**P NMR spectrum of the reaction mixture of Table S2, Entry 3**

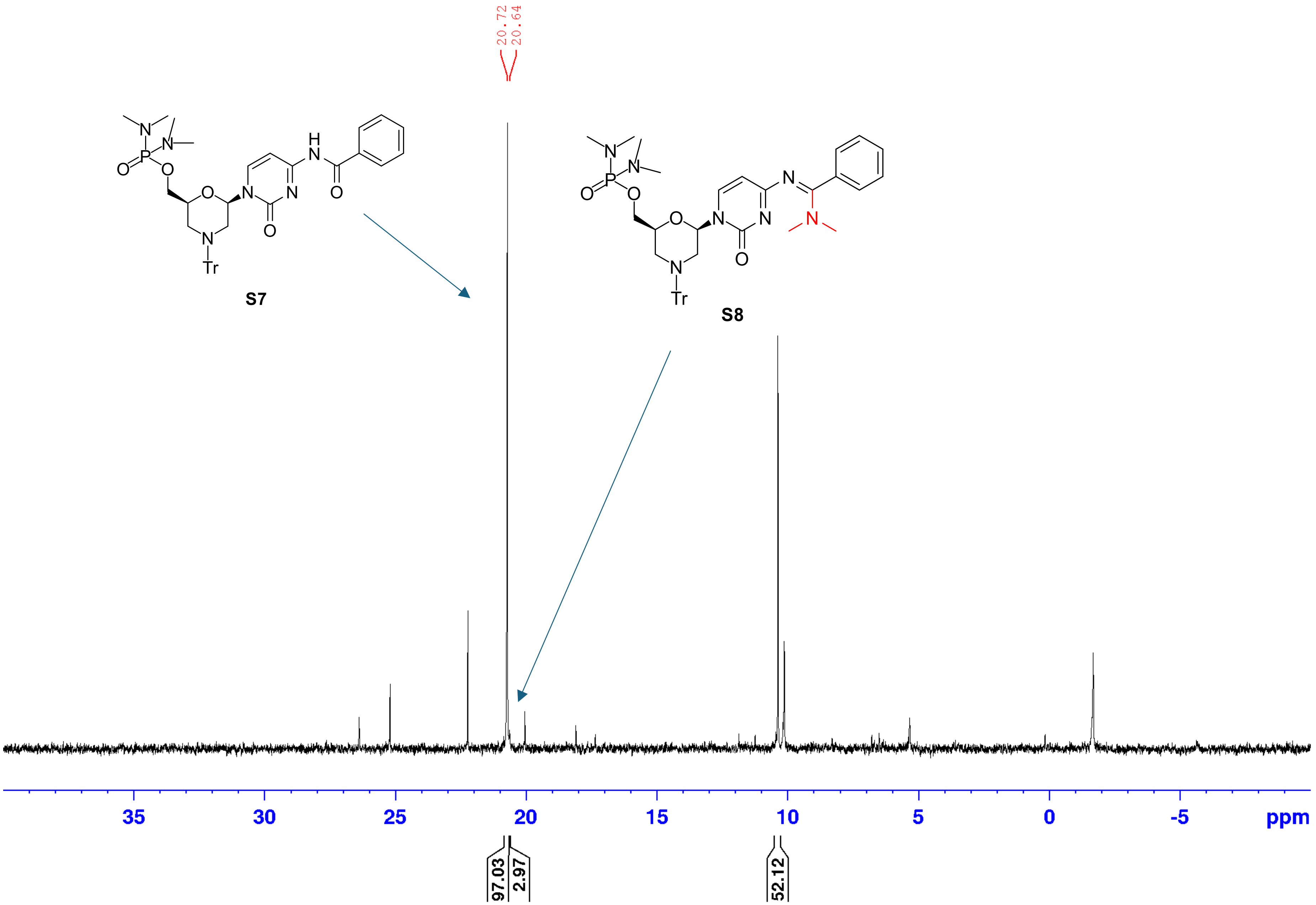

Figure S 45. ^{31}P NMR spectrum after the condensation with chloridate and dimethylamine of Table S2, Entry 3

4.1 Copies of ^{1}H, ^{13}C, ^{31}P NMR, COSY, HMBC, HMQC, and HSQC spectra

^{1}H NMR (400 MHz, $CDCl_3$) of **3**

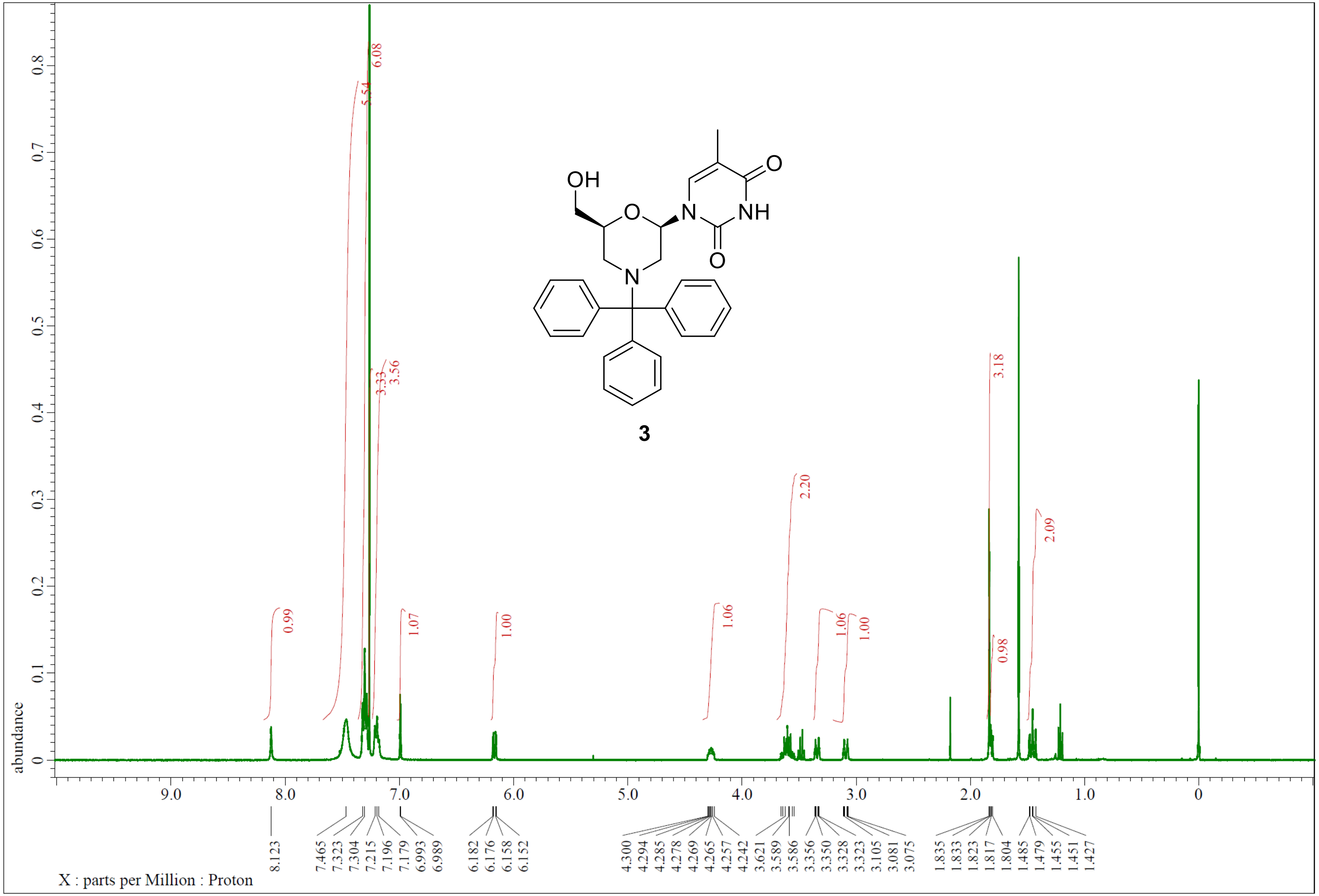

^{1}H NMR (400 MHz, CDCl3) of **4**

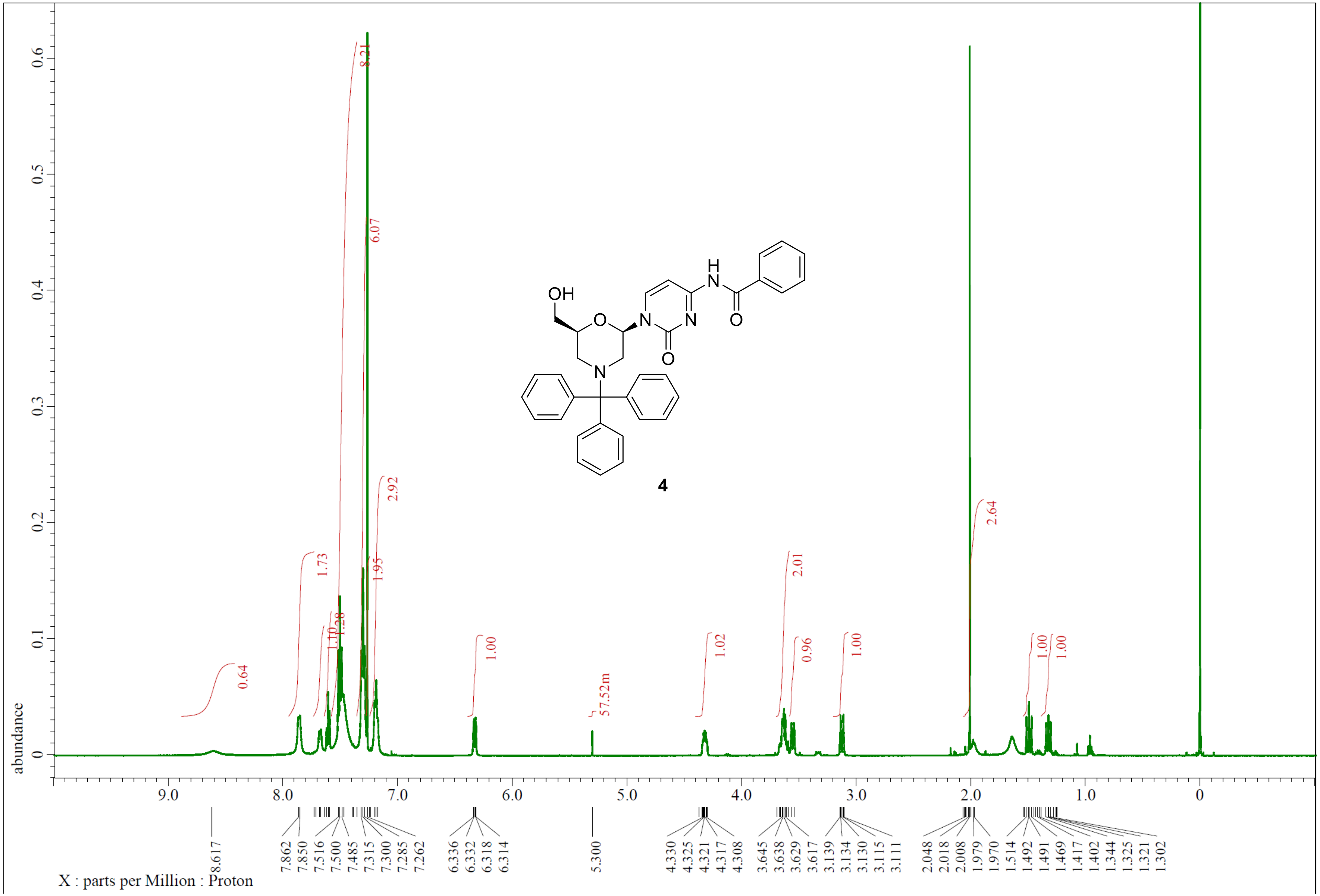

^{1}H NMR (400 MHz, $CDCl_3$) of **5**

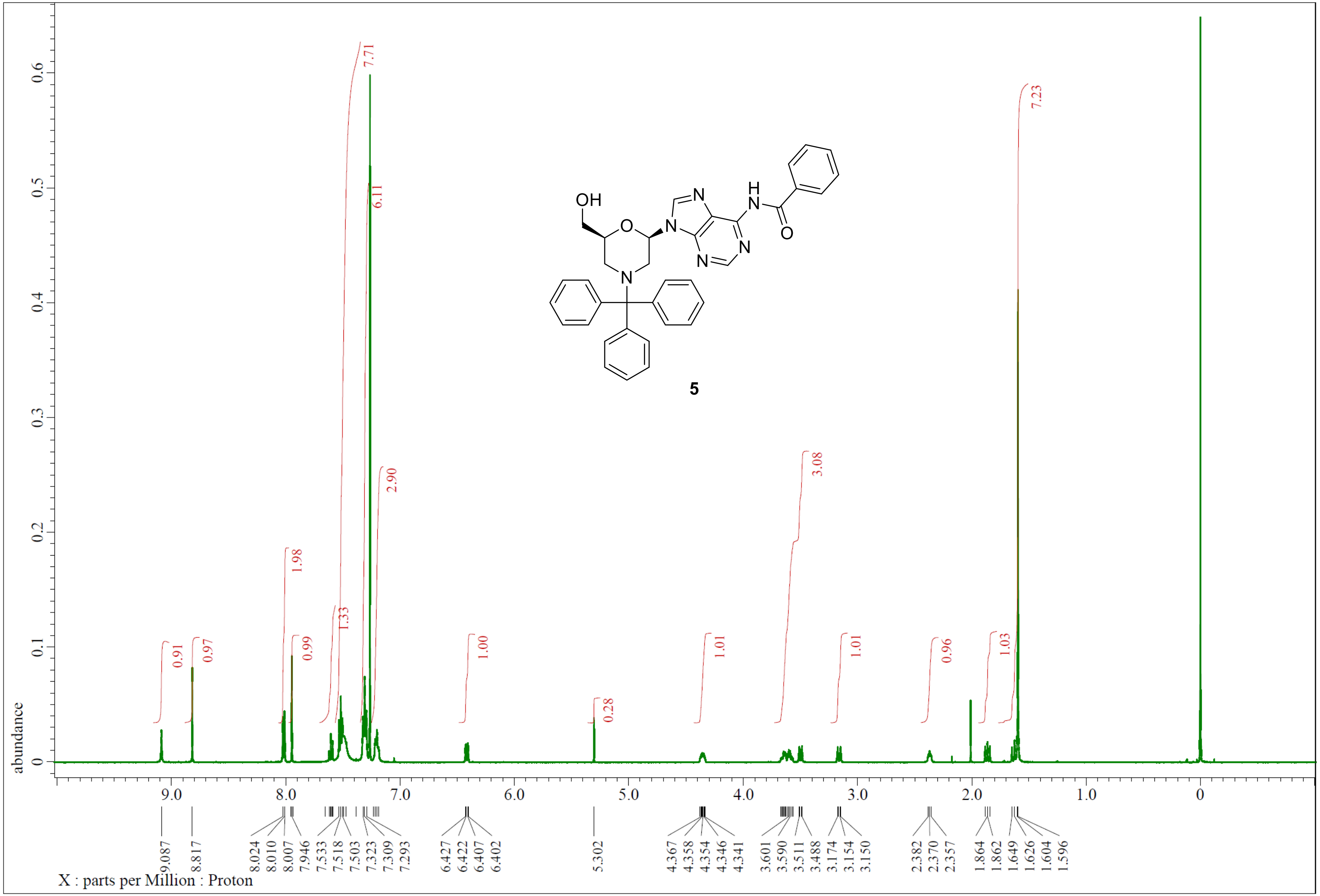

^{1}H NMR (400 MHz, $CDCl_3$) of **S2**

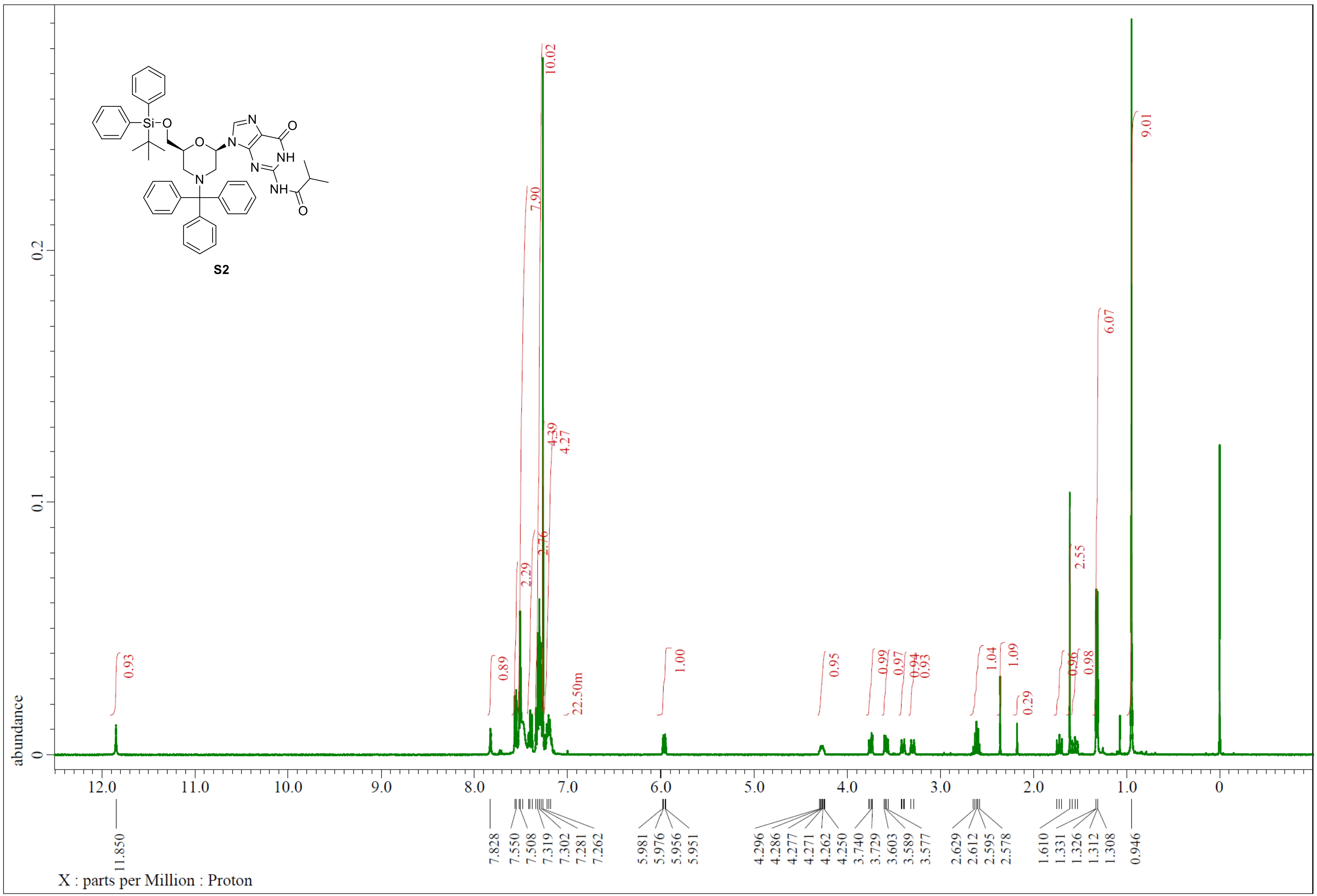

^{1}H NMR (400 MHz, $CDCl_3$) of **S4**

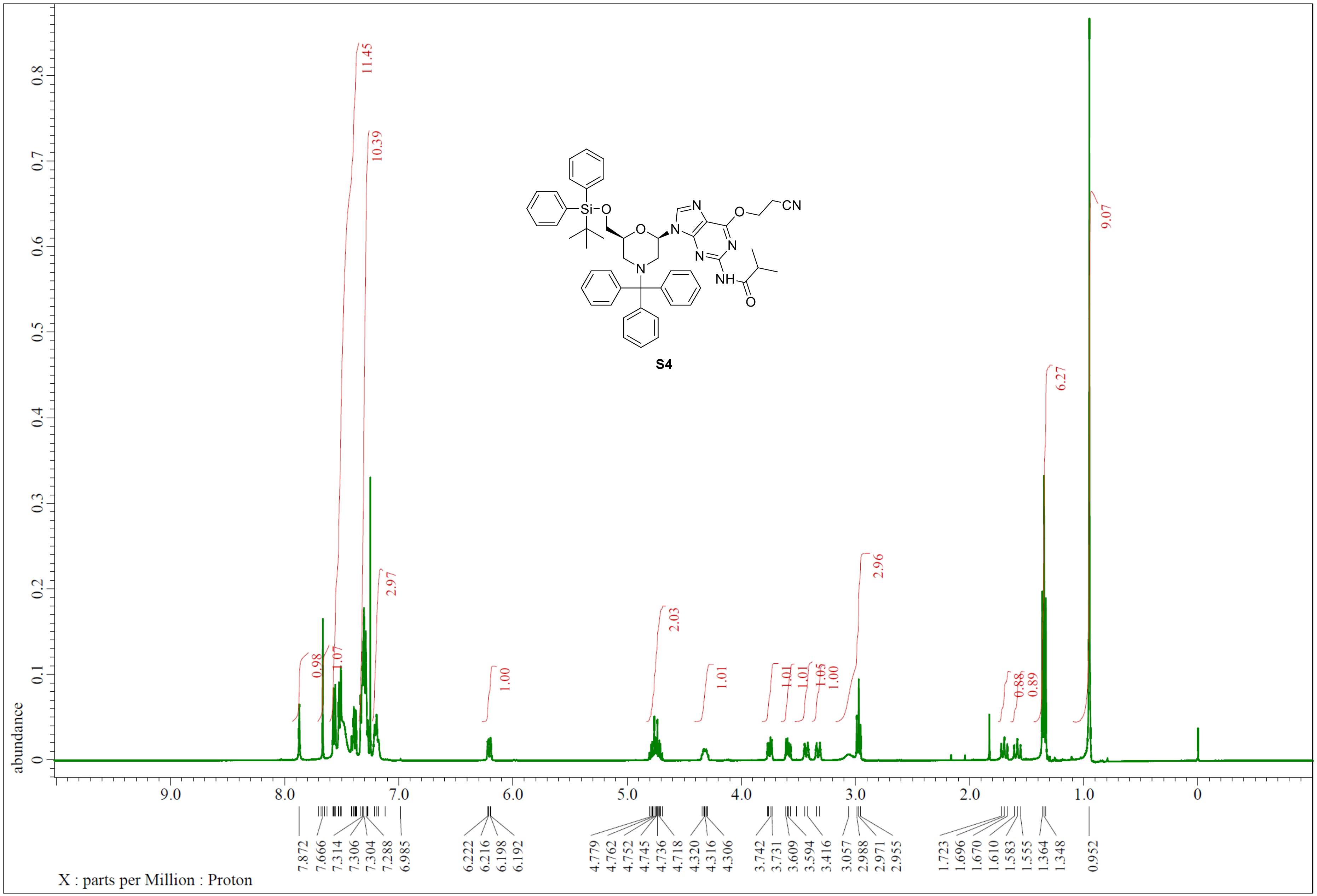

$^{13}C\{^1H\}$ NMR (101 MHz, CDCl$_3$) of **S4**

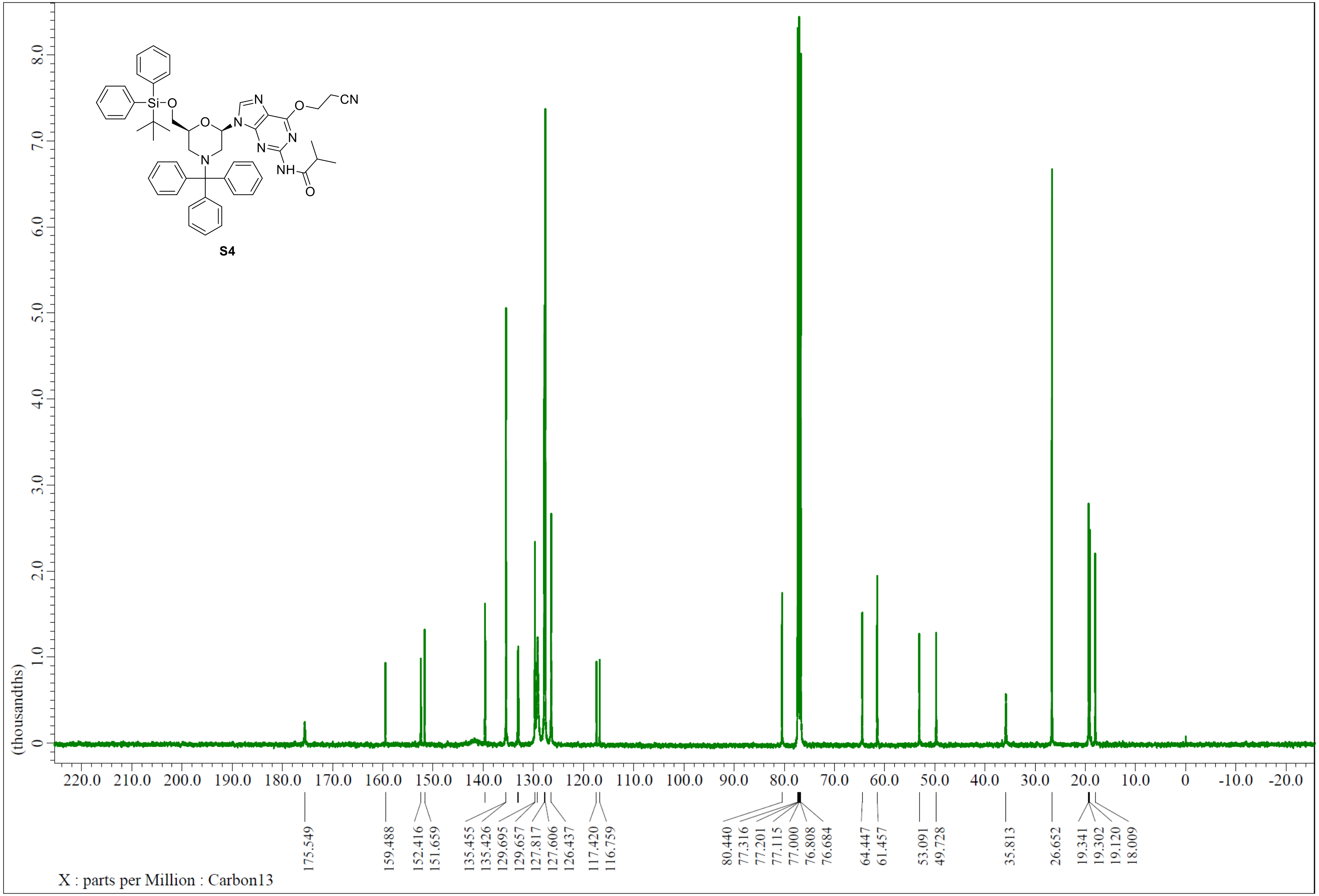

COSY ($CDCl_3$) of **S4**

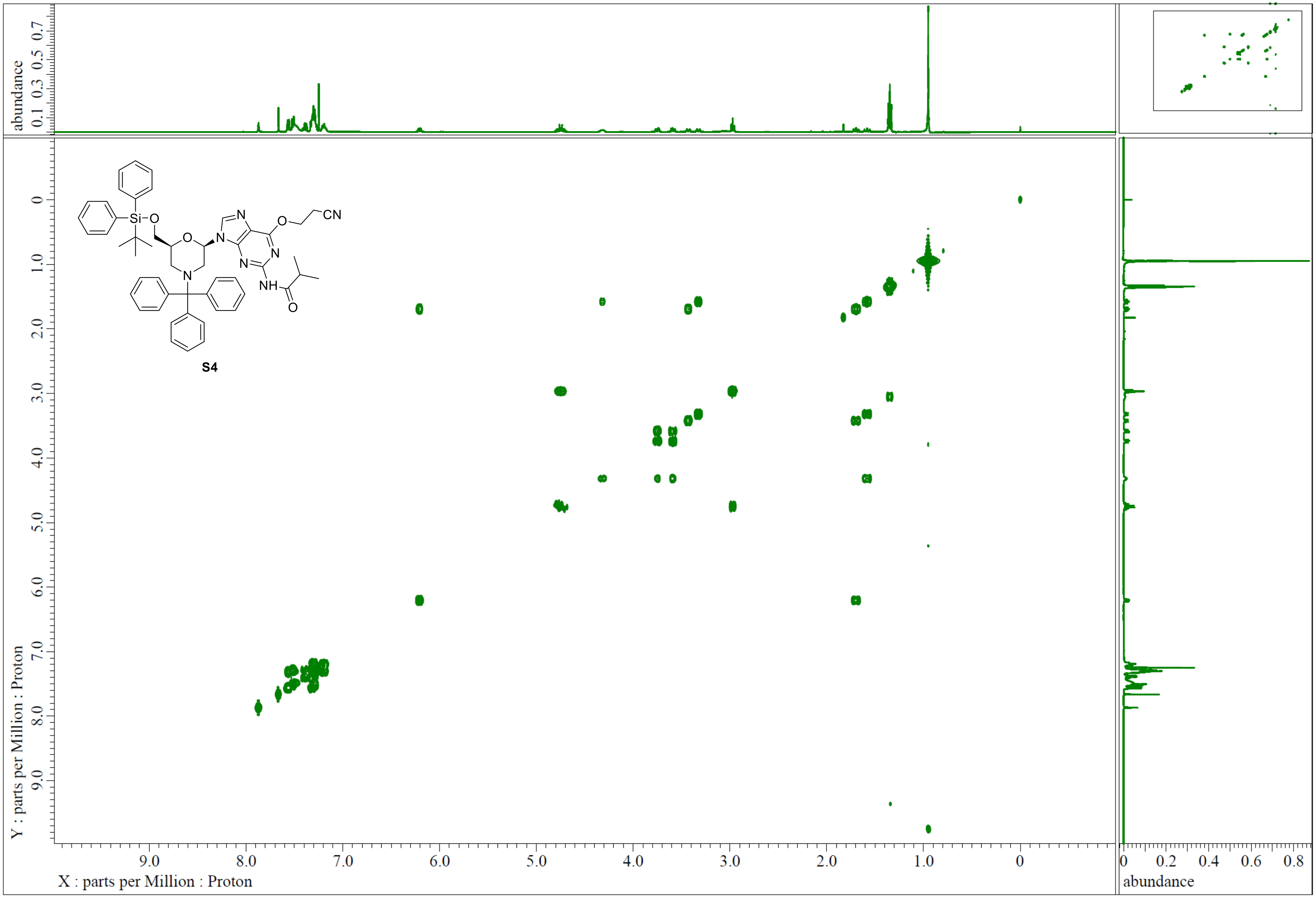

HMQC ($CDCl_3$) of **S4**

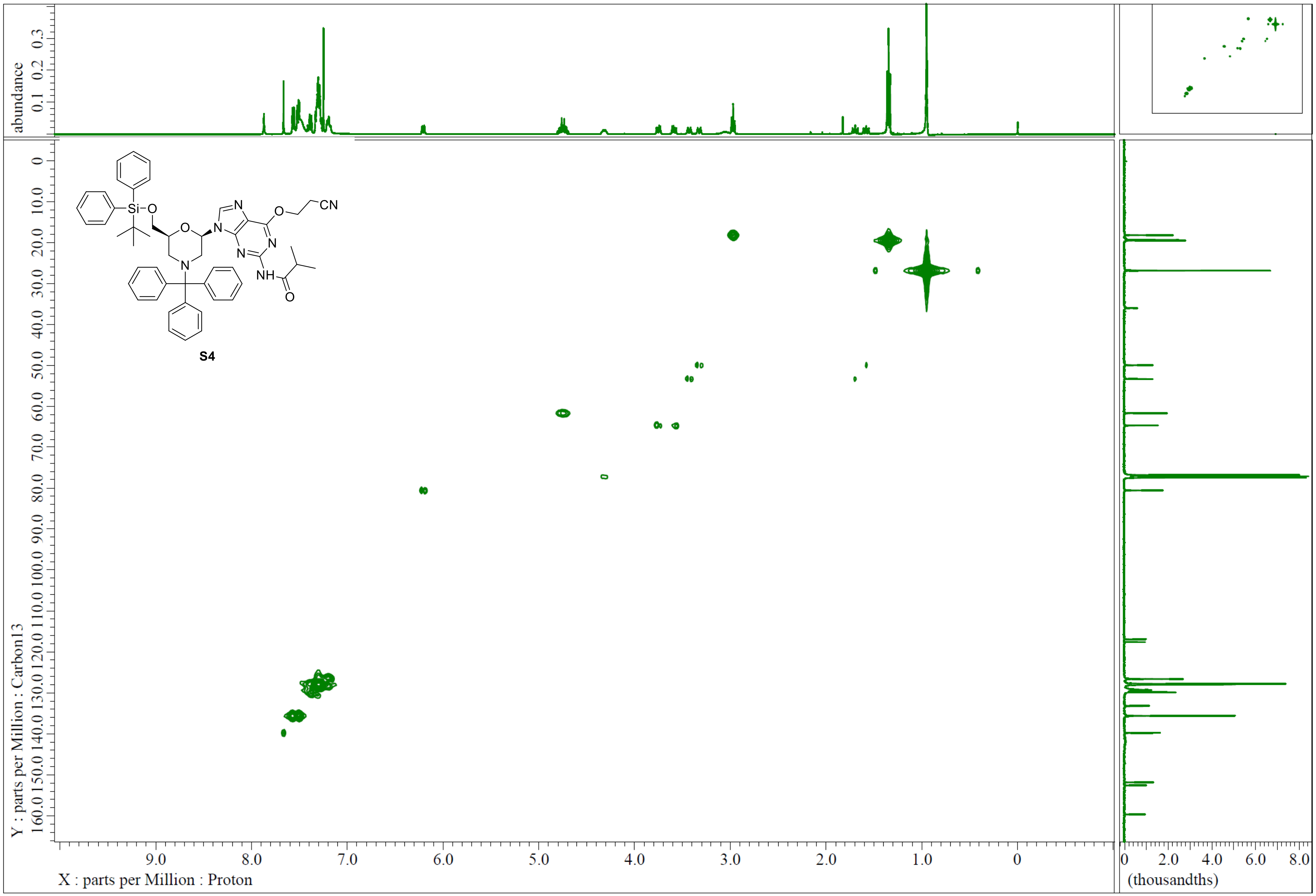

HMBC ($CDCl_3$) of **S4**

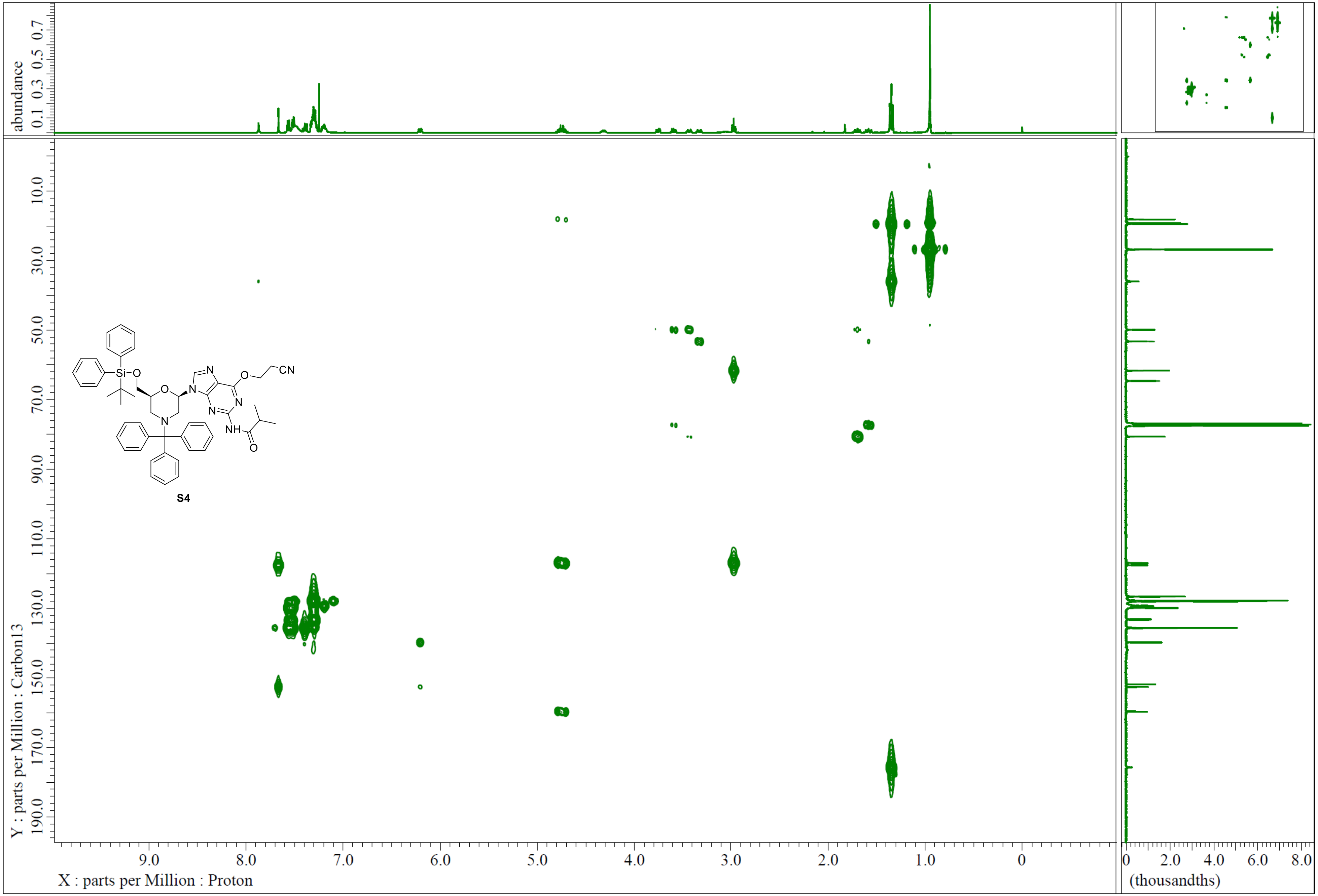

^{1}H NMR (400 MHz, $CDCl_3$) of **6**

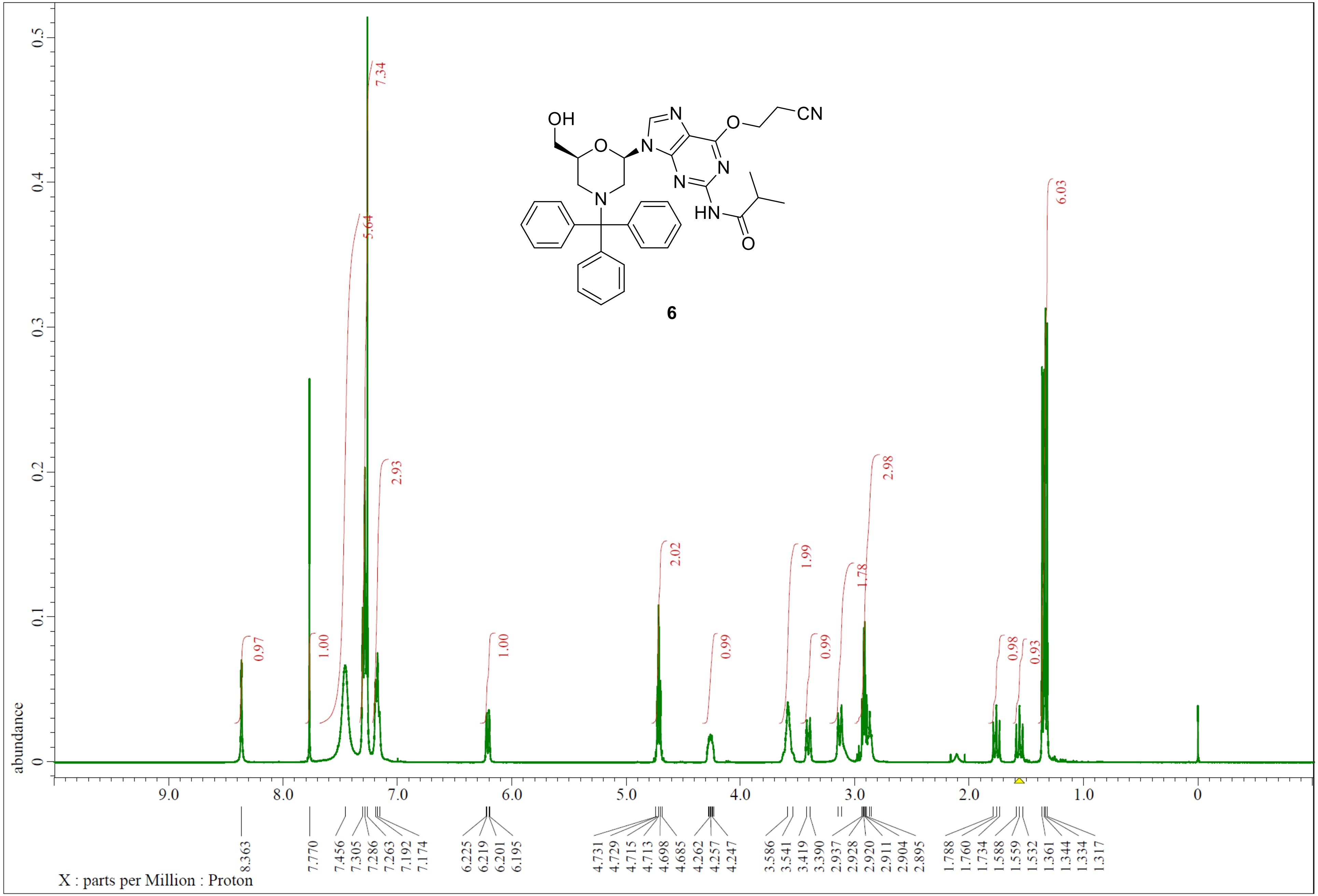

$^{13}C\{^{1}H\}$ NMR (101 MHz, $CDCl_3$) of **6**

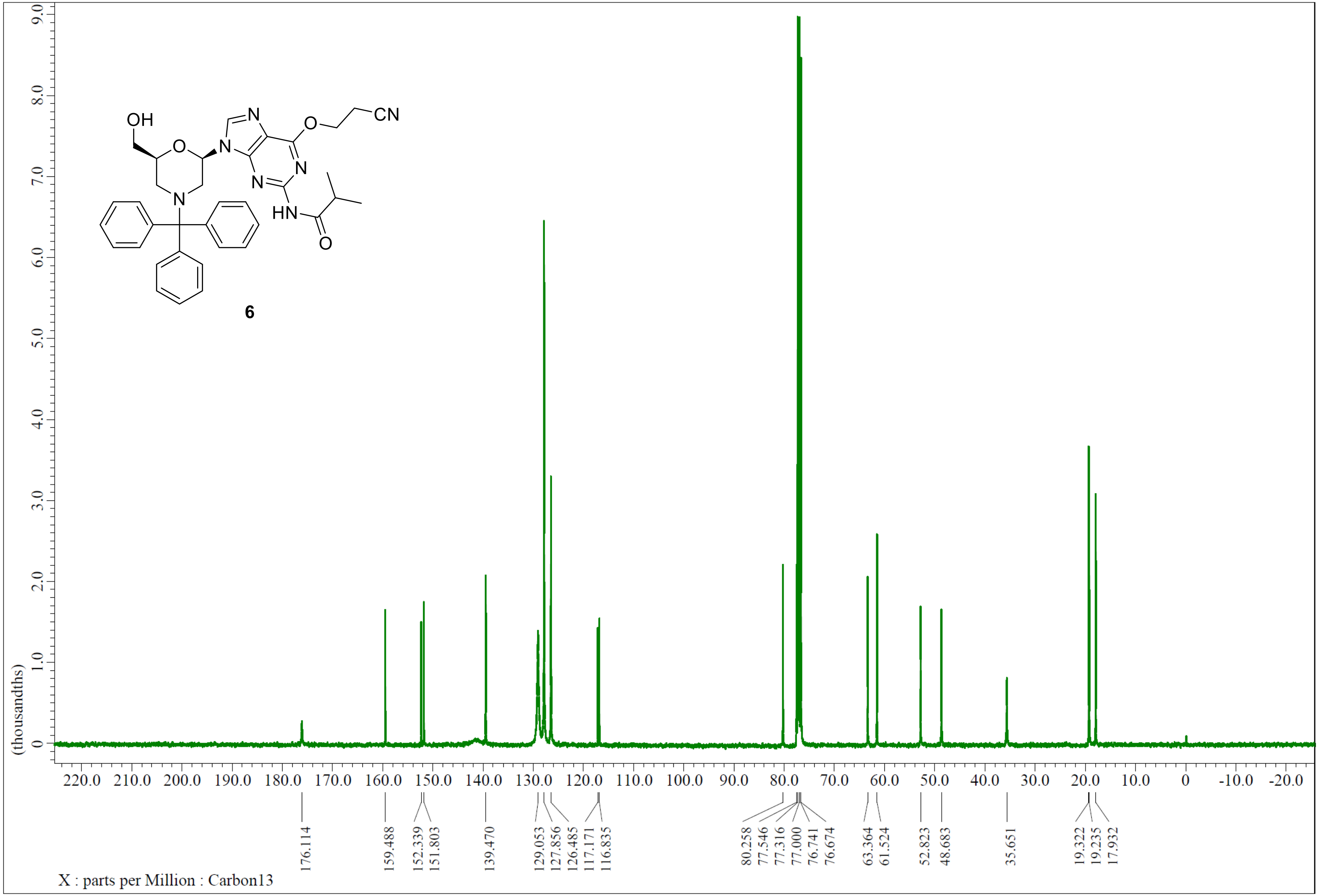

COSY ($CDCl_3$) of **6**

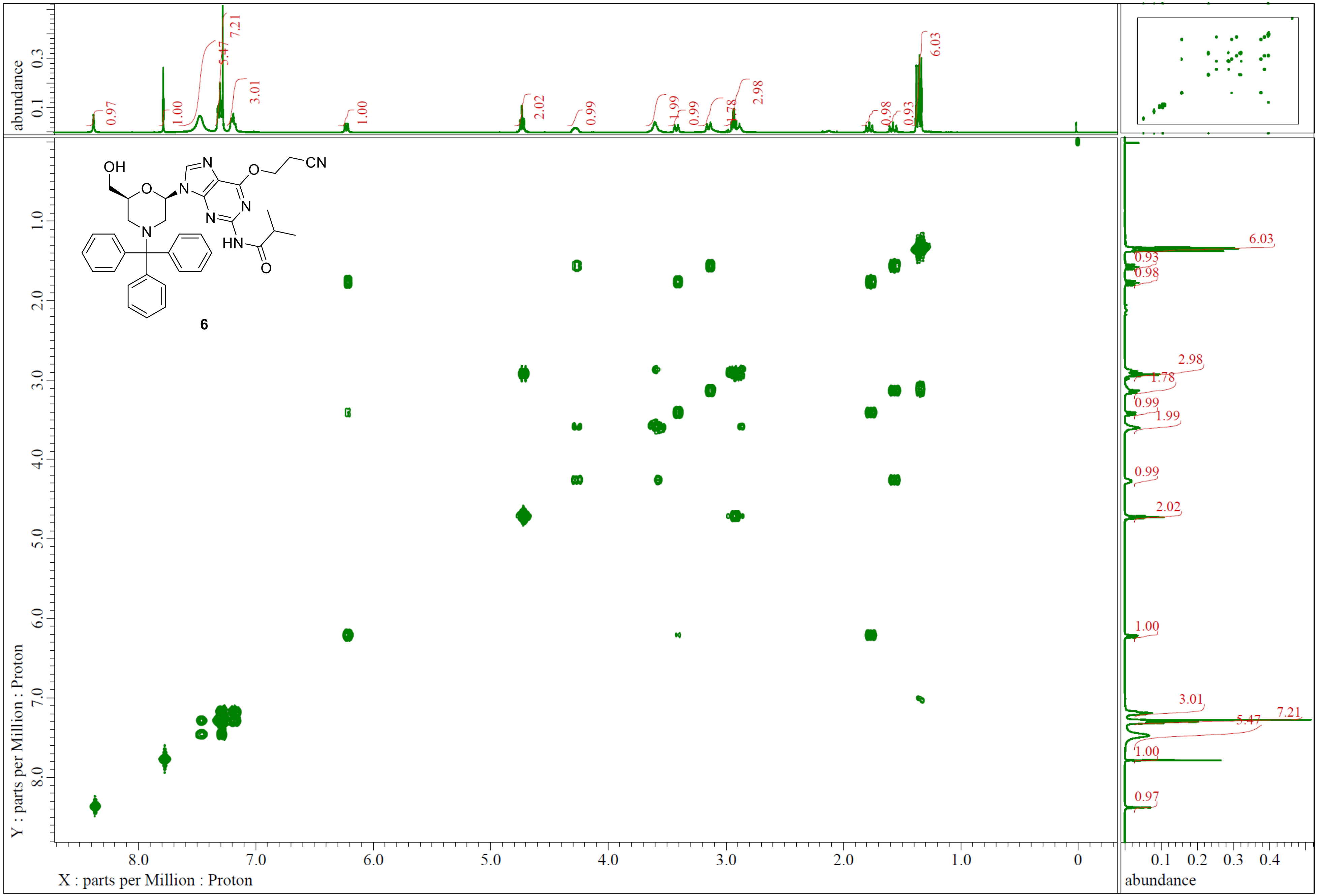

HMQC ($CDCl_3$) of **6**

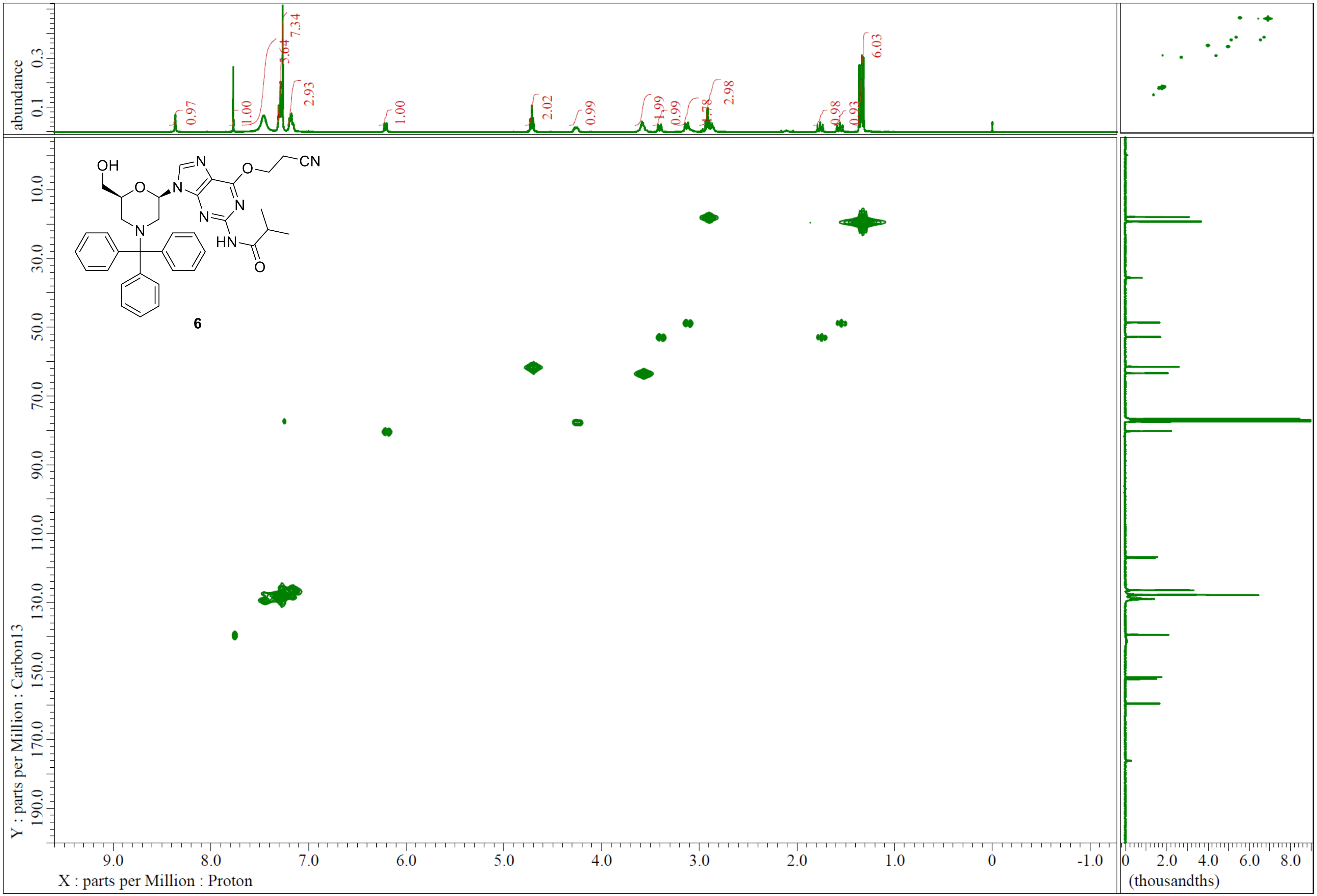

HMBC ($CDCl_3$) of **6**

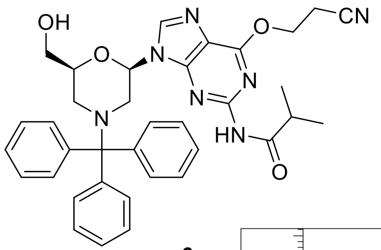

6

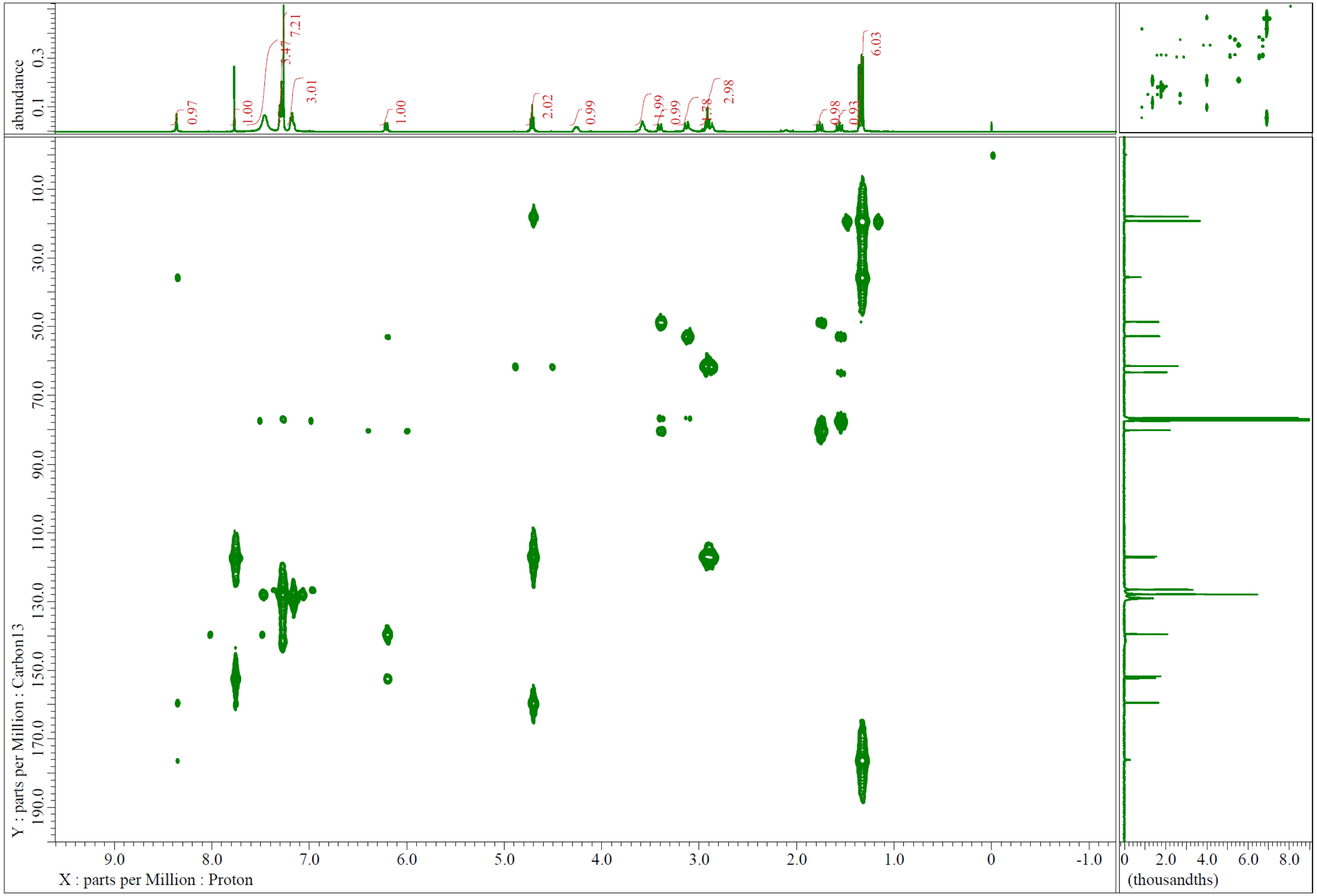

^{1}H NMR (500 MHz, $CDCl_3$) of (*R*p)-**7**

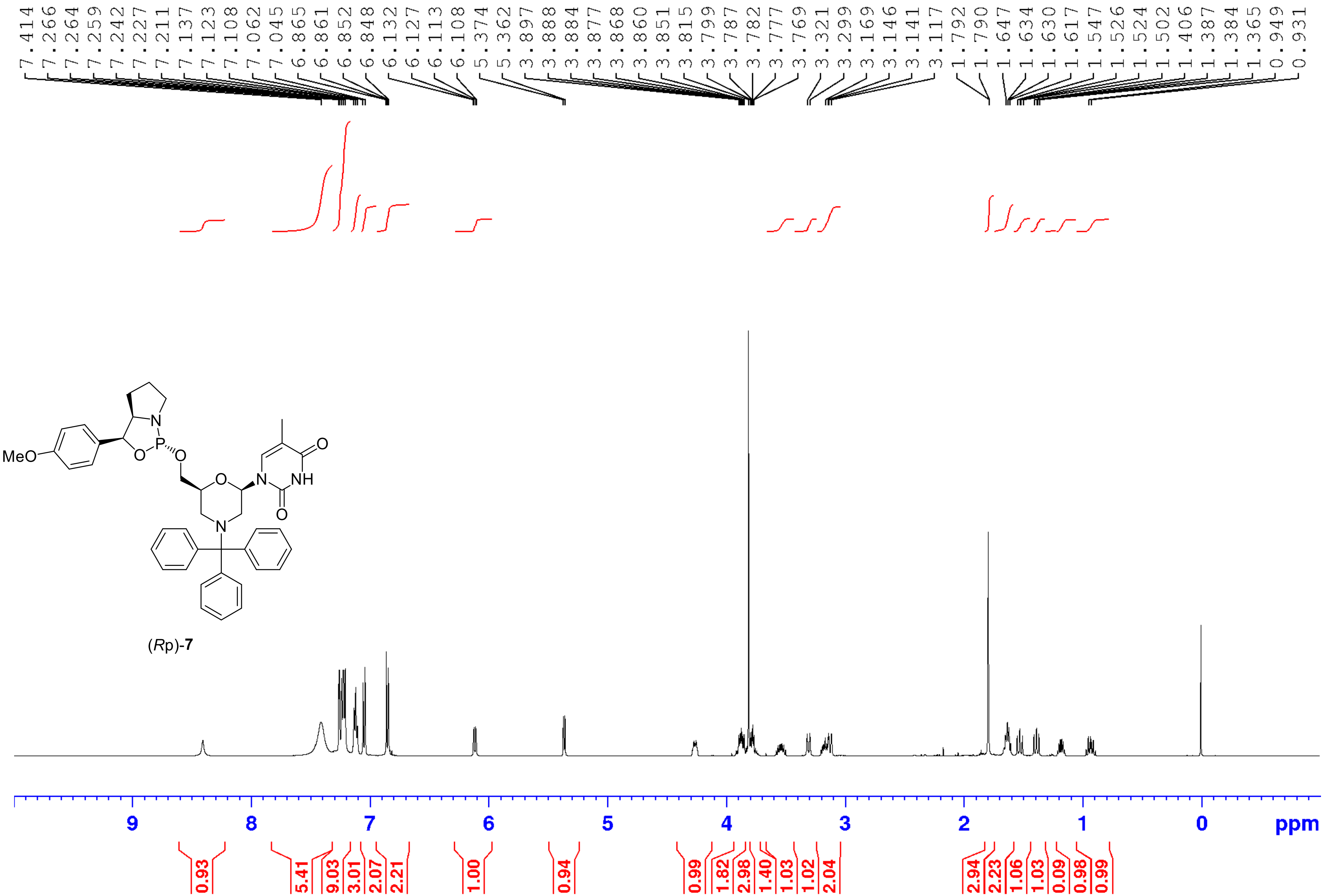

(*R*p)-**7**

^{13}C {^{1}H} NMR (126 MHz, $CDCl_3$) of (*R*p)-**7**

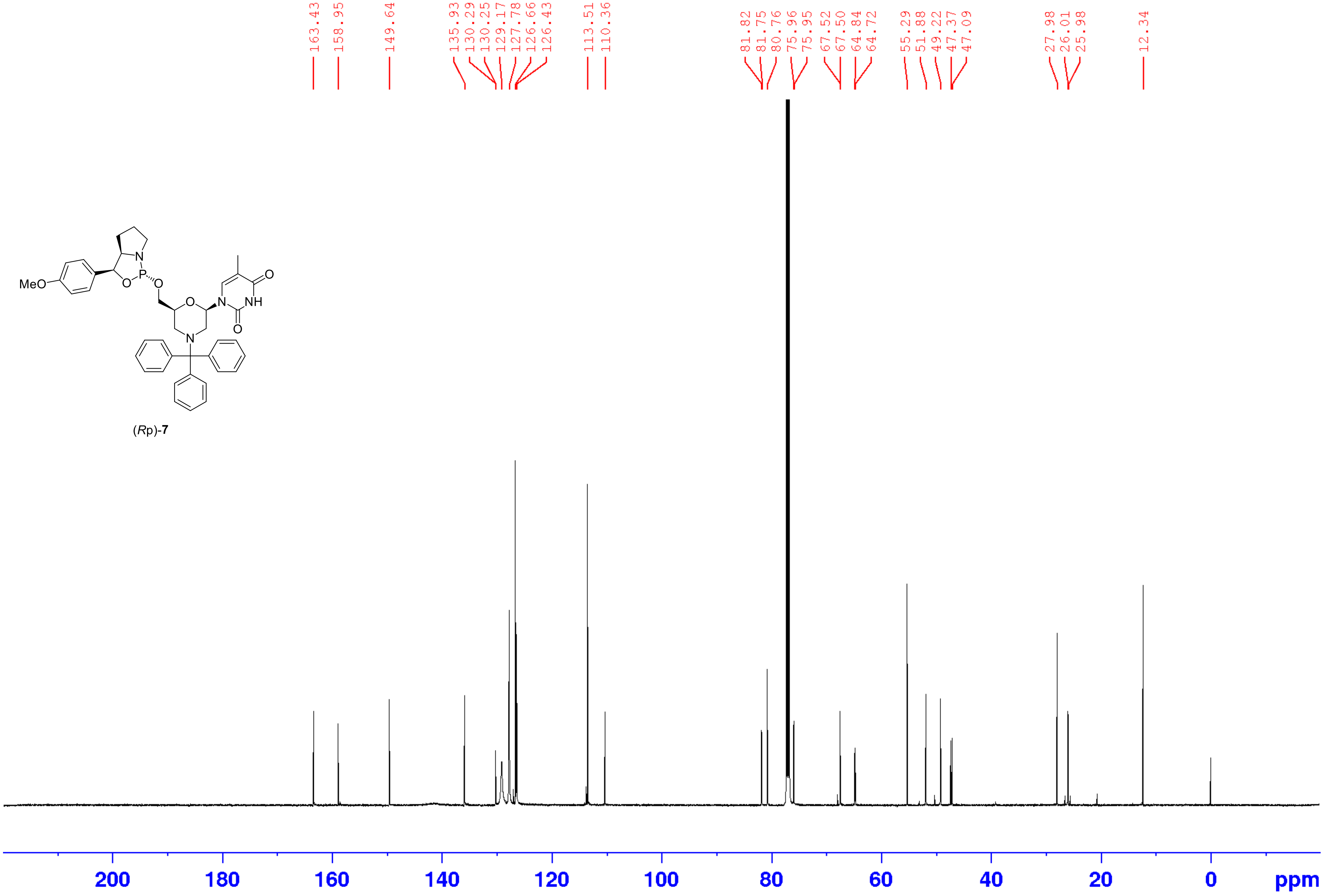

$^{31}P\{^{1}H\}$ NMR (202 MHz, $CDCl_3$) of (*R*p)-**7**

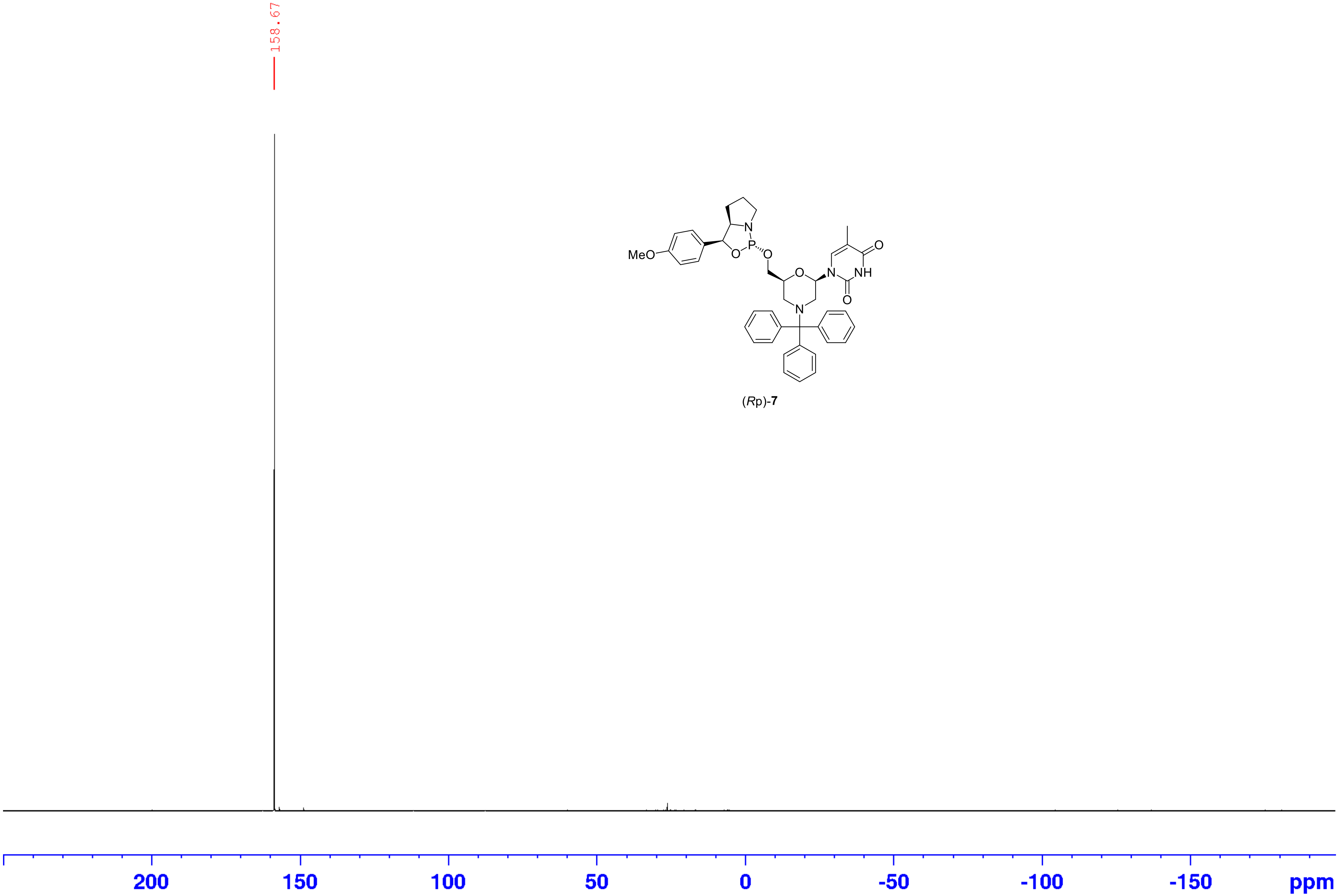

COSY ($CDCl_3$) of (*R*p)-**7**

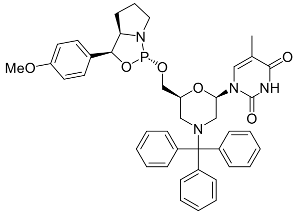

(*R*p)-**7**

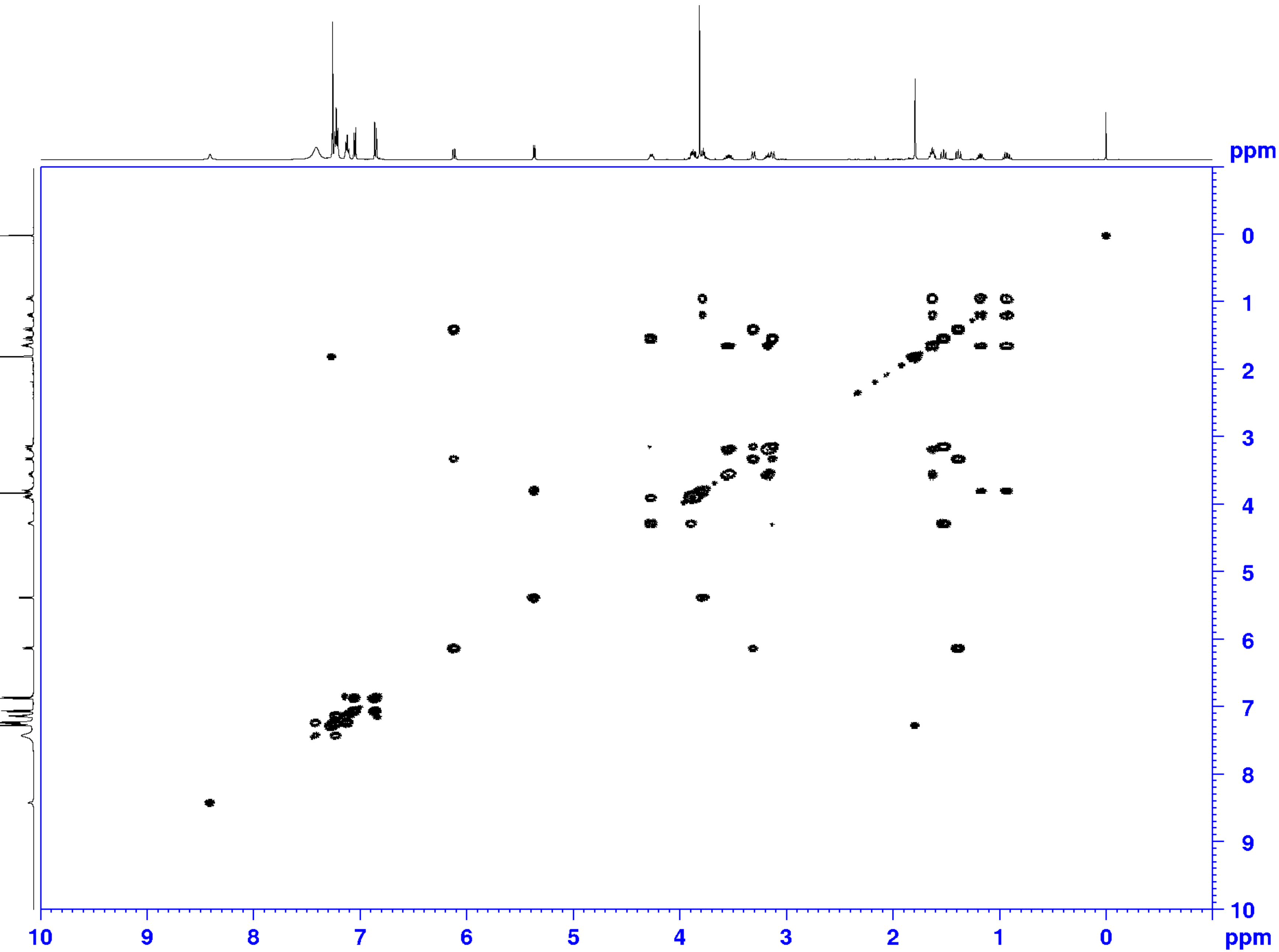

HSQC ($CDCl_3$) of (*R*p)-**7**

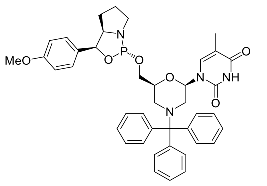

(*R*p)-**7**

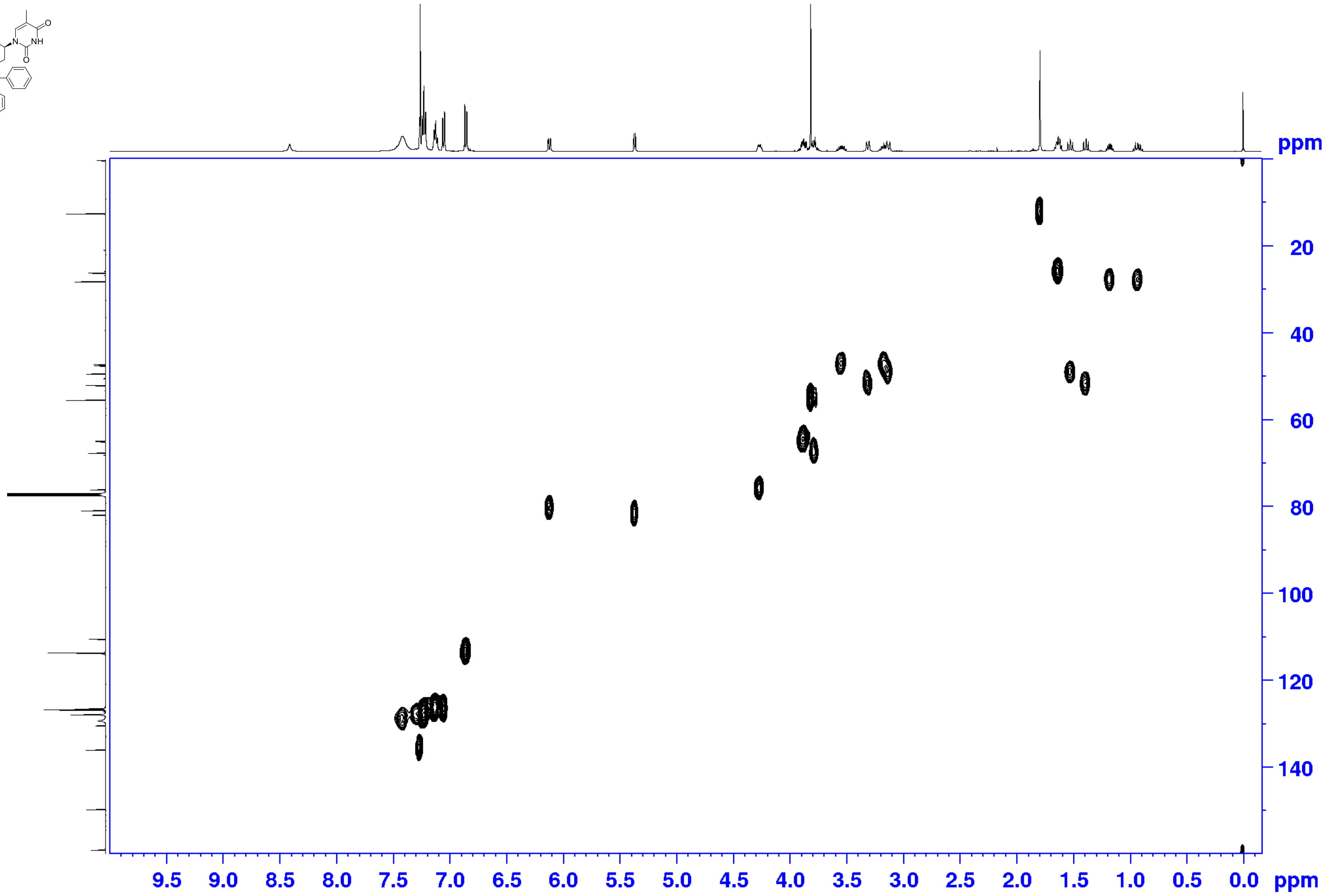

HMBC ($CDCl_3$) of (*R*p)-**7**

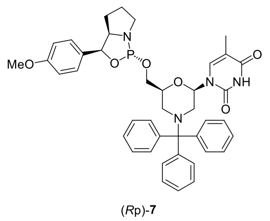

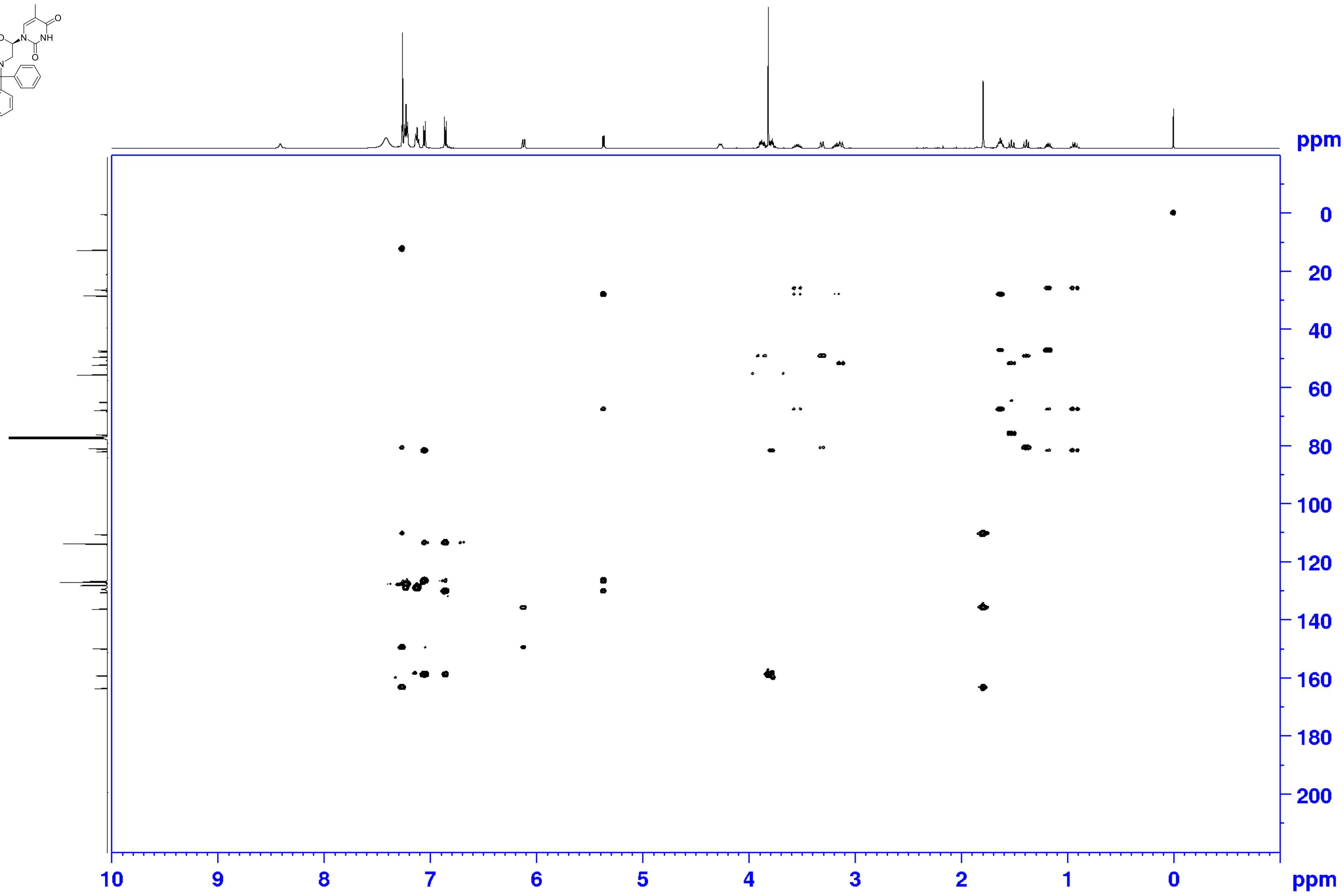

^{1}H NMR (500 MHz, $CDCl_3$) of (*S*p)-**7**

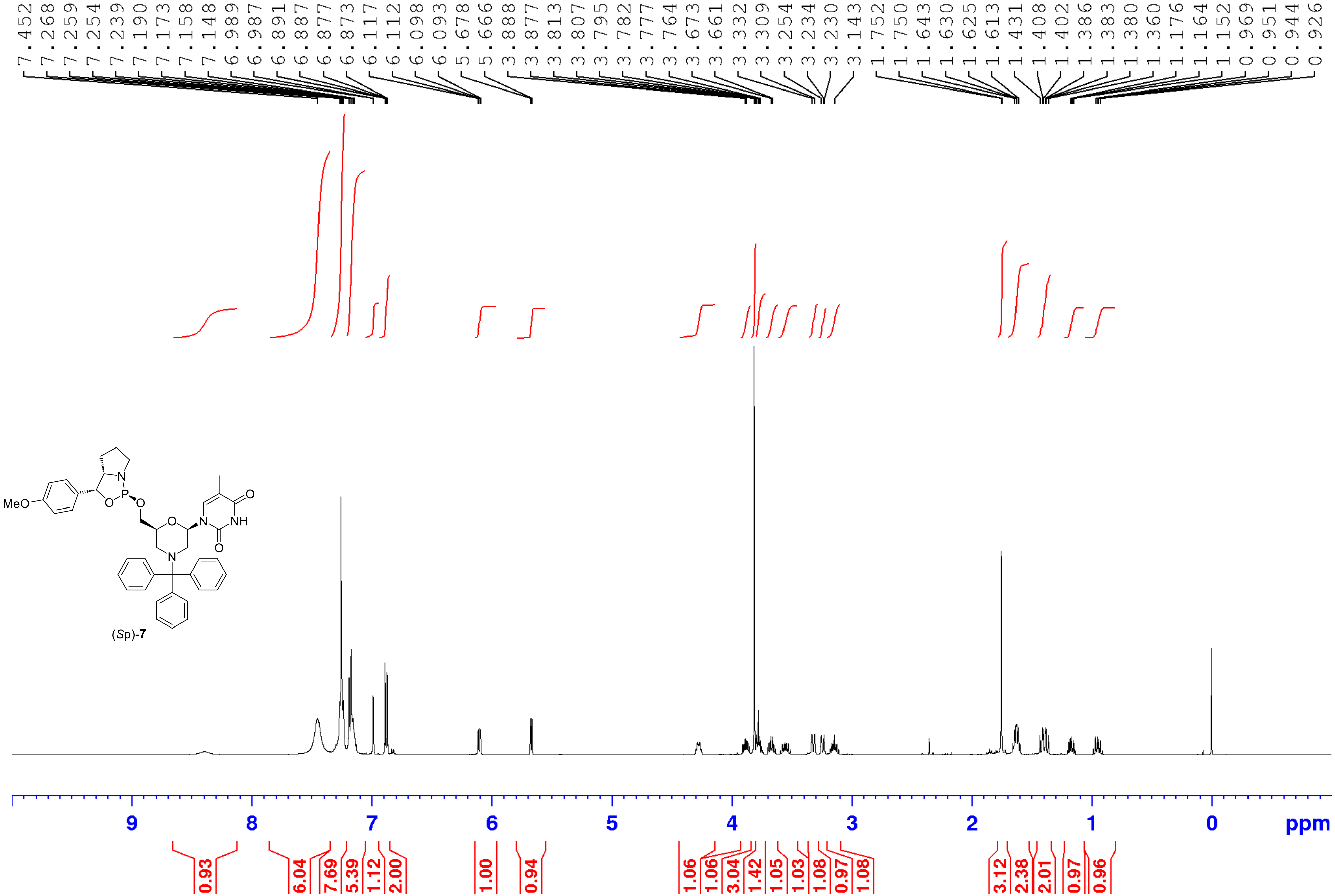

^{13}C {^{1}H} NMR (126 MHz, $CDCl_3$) of (*S*p)-**7**

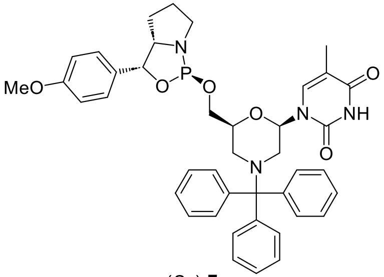

(*S*p)-**7**

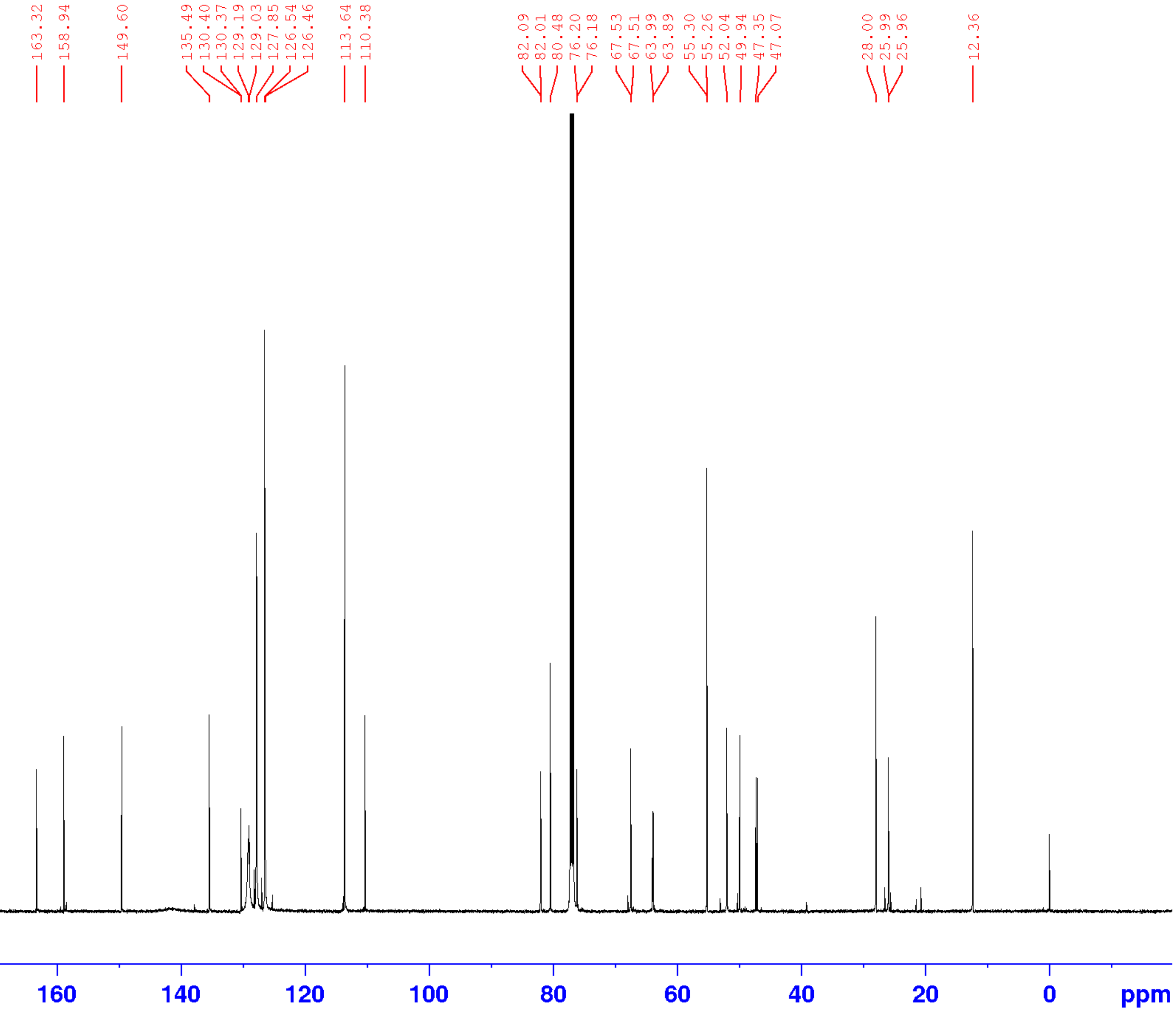

^{31}P {^{1}H} NMR (202 MHz, $CDCl_3$) of (*S*p)-**7**

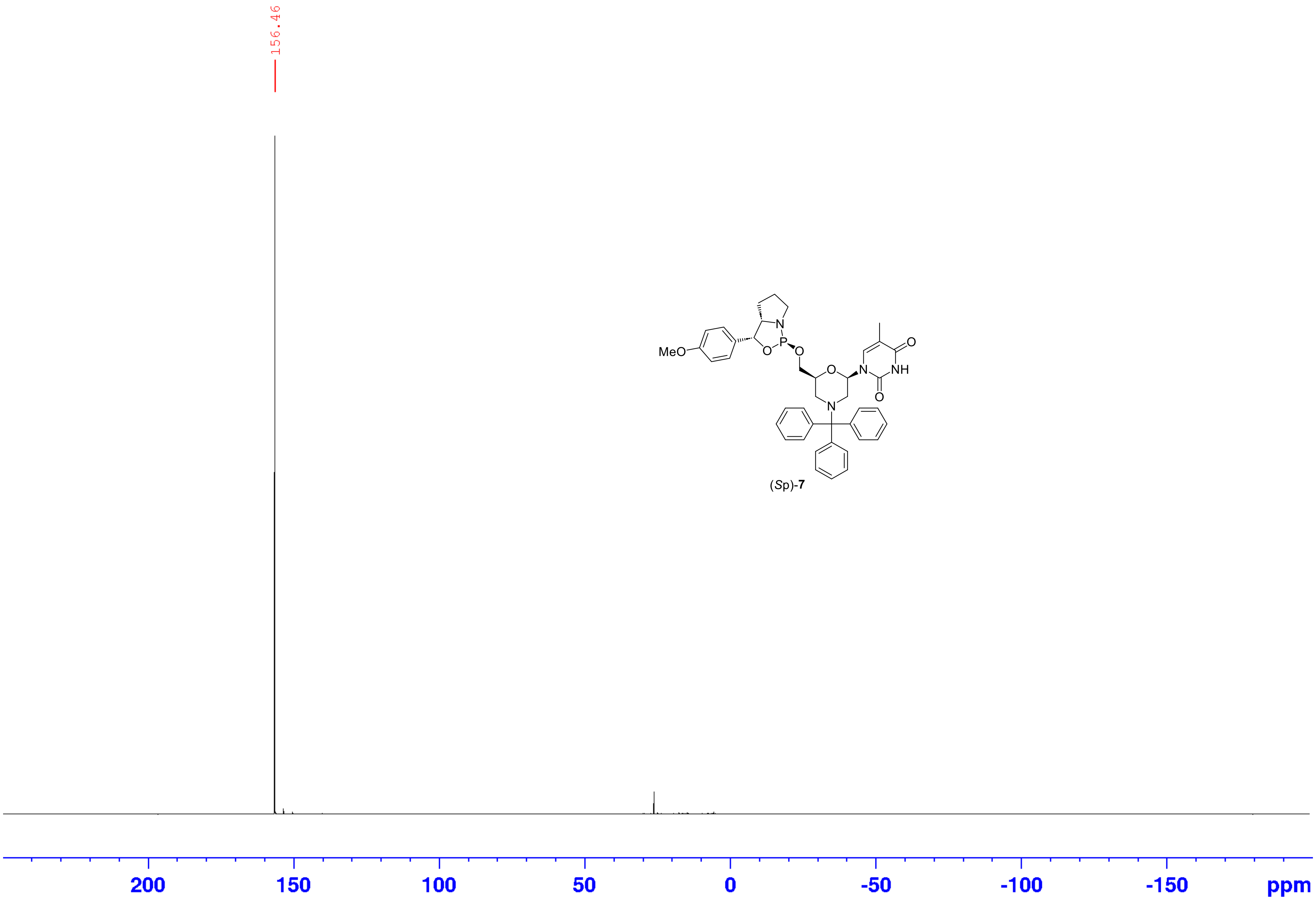

COSY ($CDCl_3$) of (*S*p)-**7**

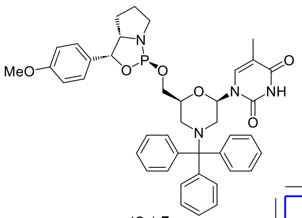

(*S*p)-**7**

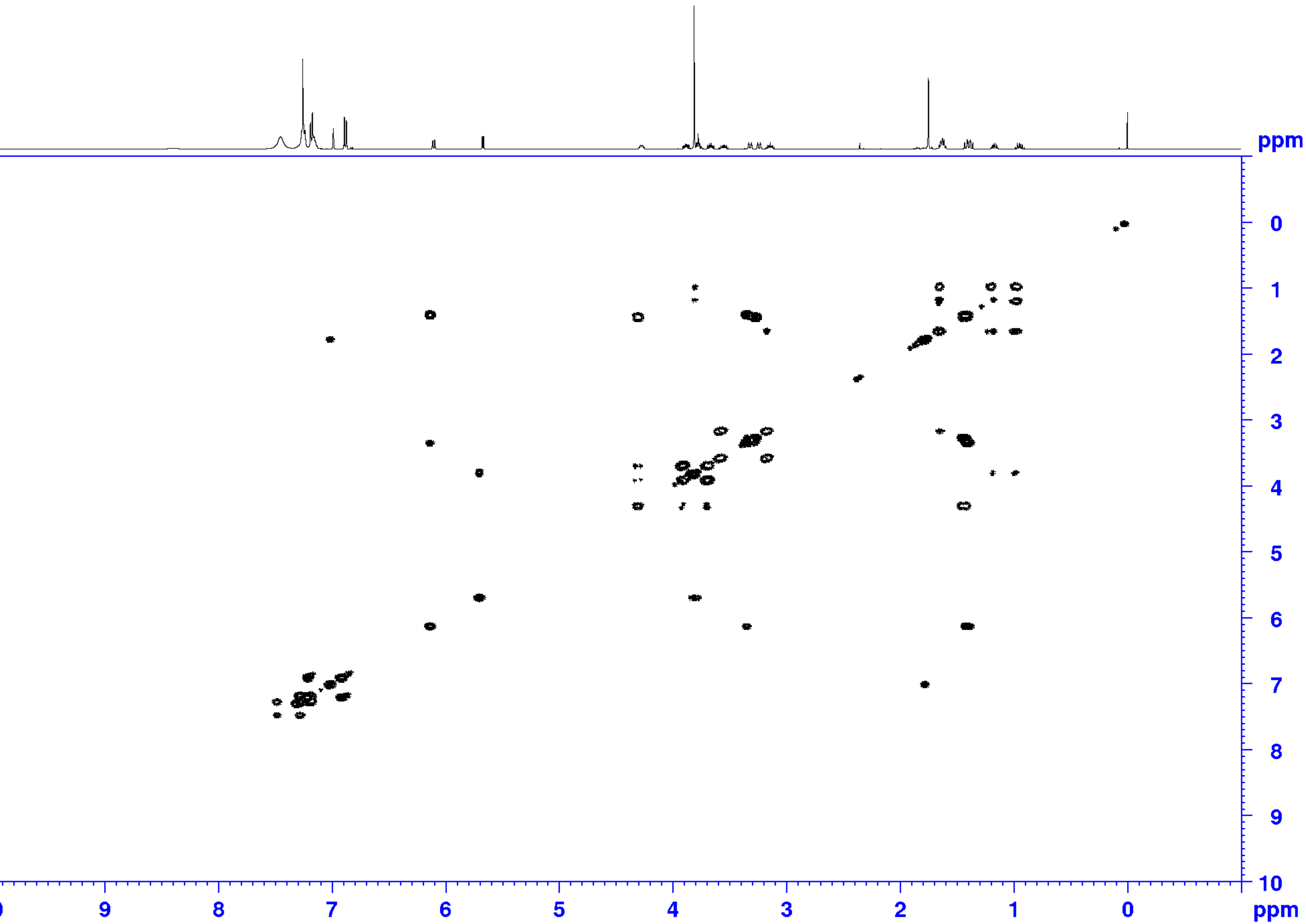

HSQC ($CDCl_3$) of (*S*p)-**7**

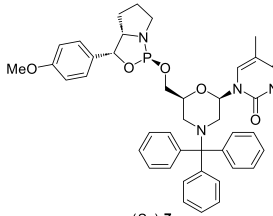

(*S*p)-**7**

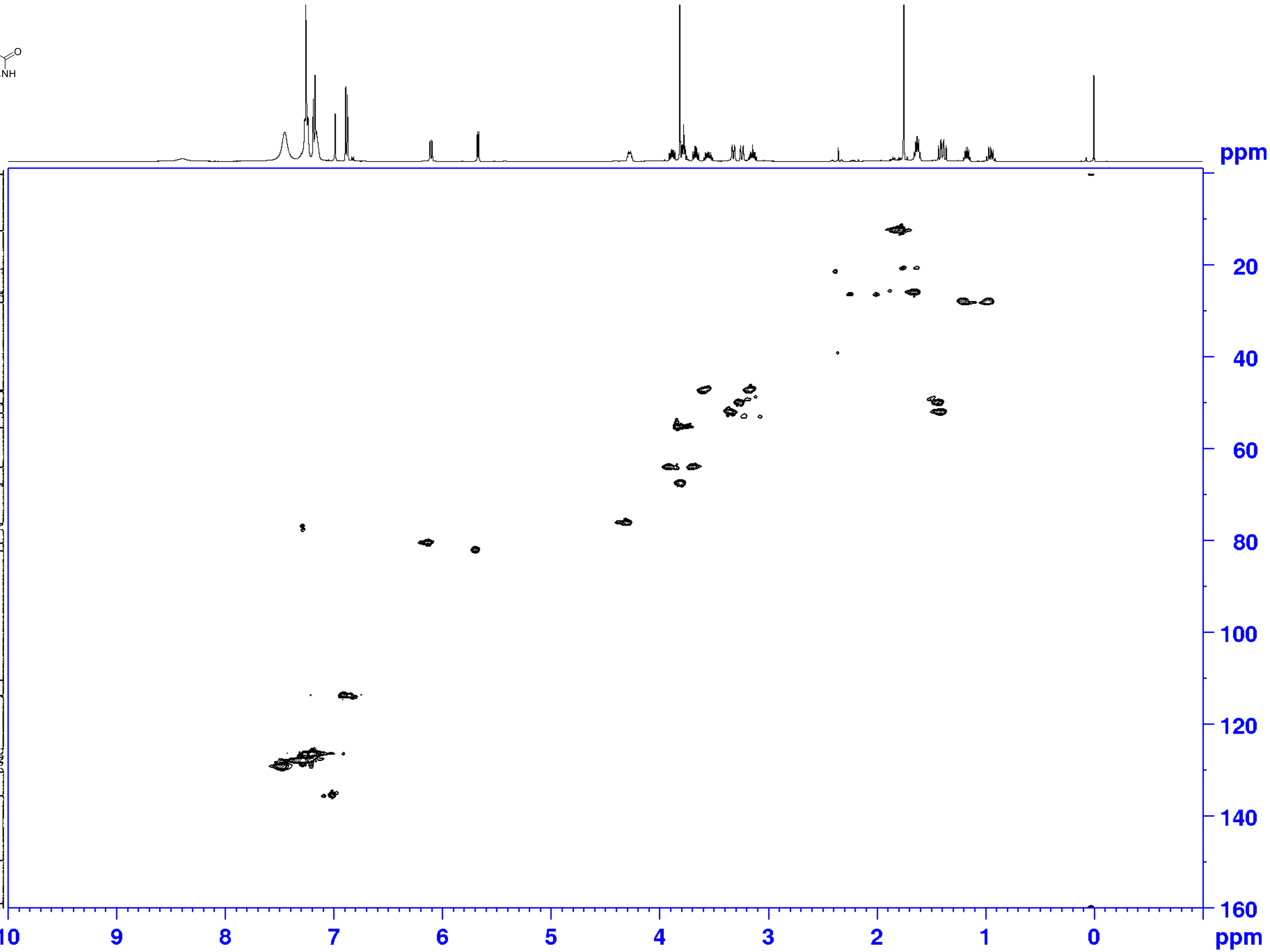

HMBC ($CDCl_3$) of (*S*p)-**7**

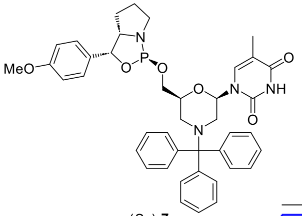

(*S*p)-**7**

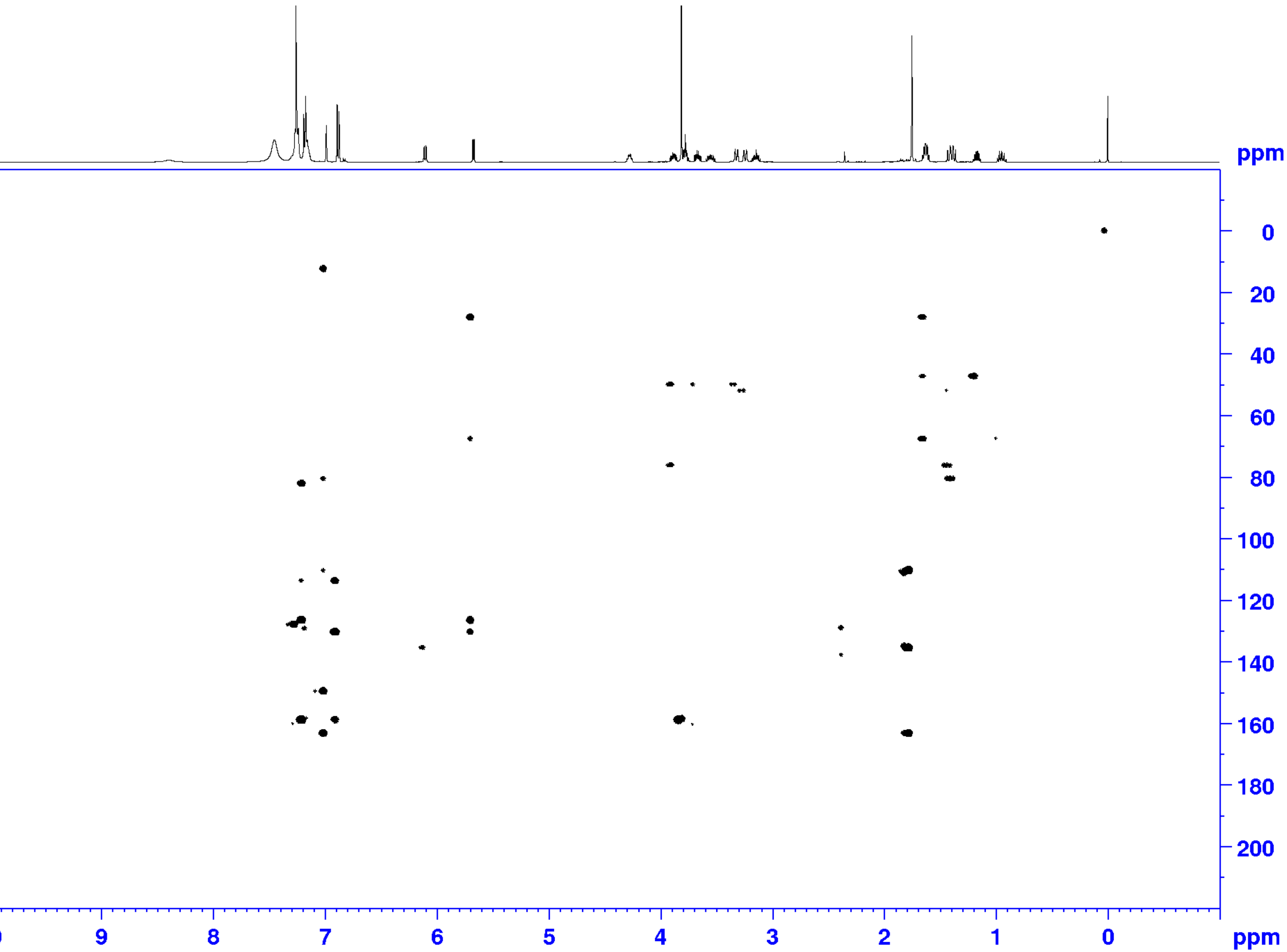

^{1}H NMR (500 MHz, CDCl$_3$) of (*R*p)-**8**

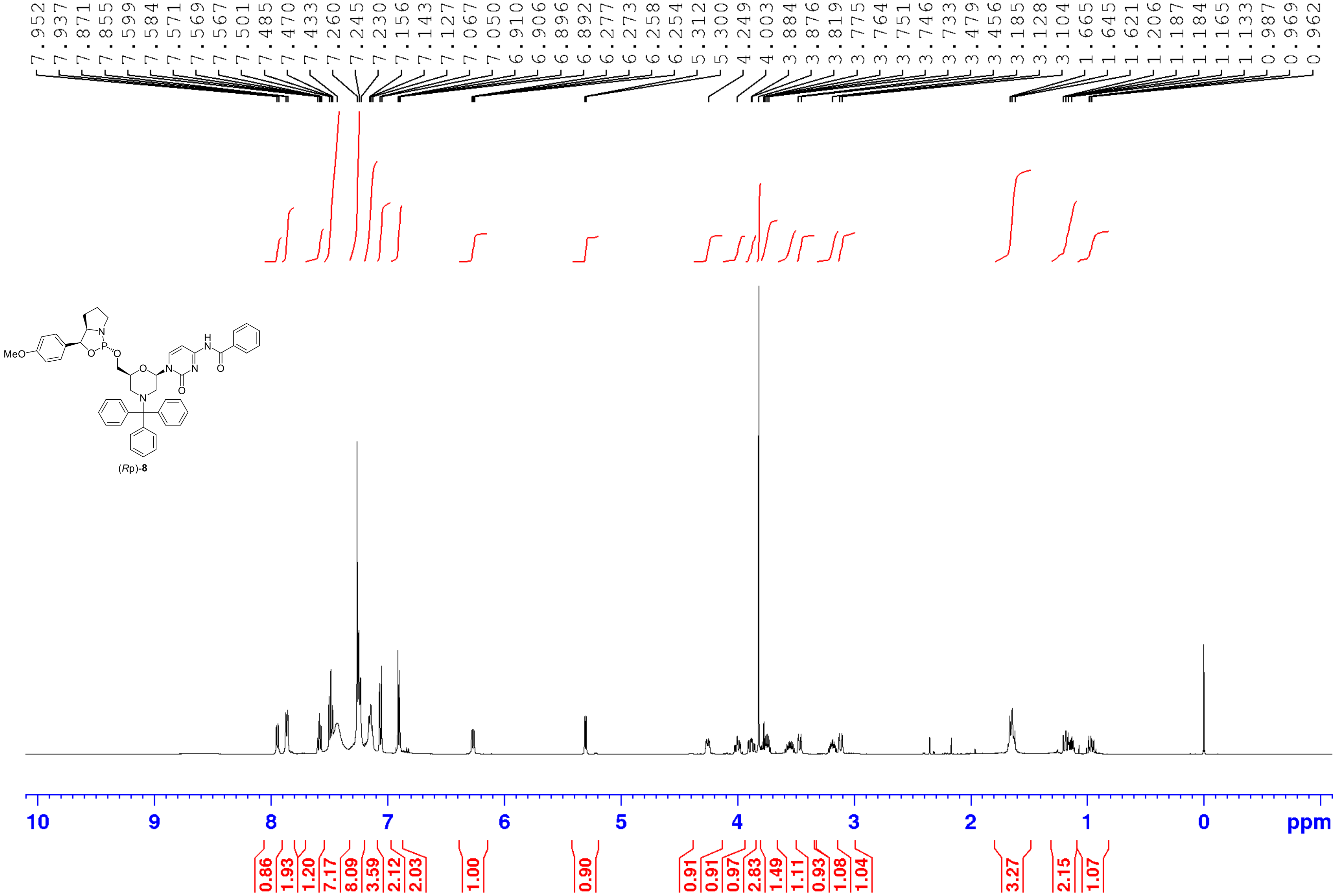

^{13}C {^{1}H} NMR (126 MHz, $CDCl_3$) of (*R*p)-**8**

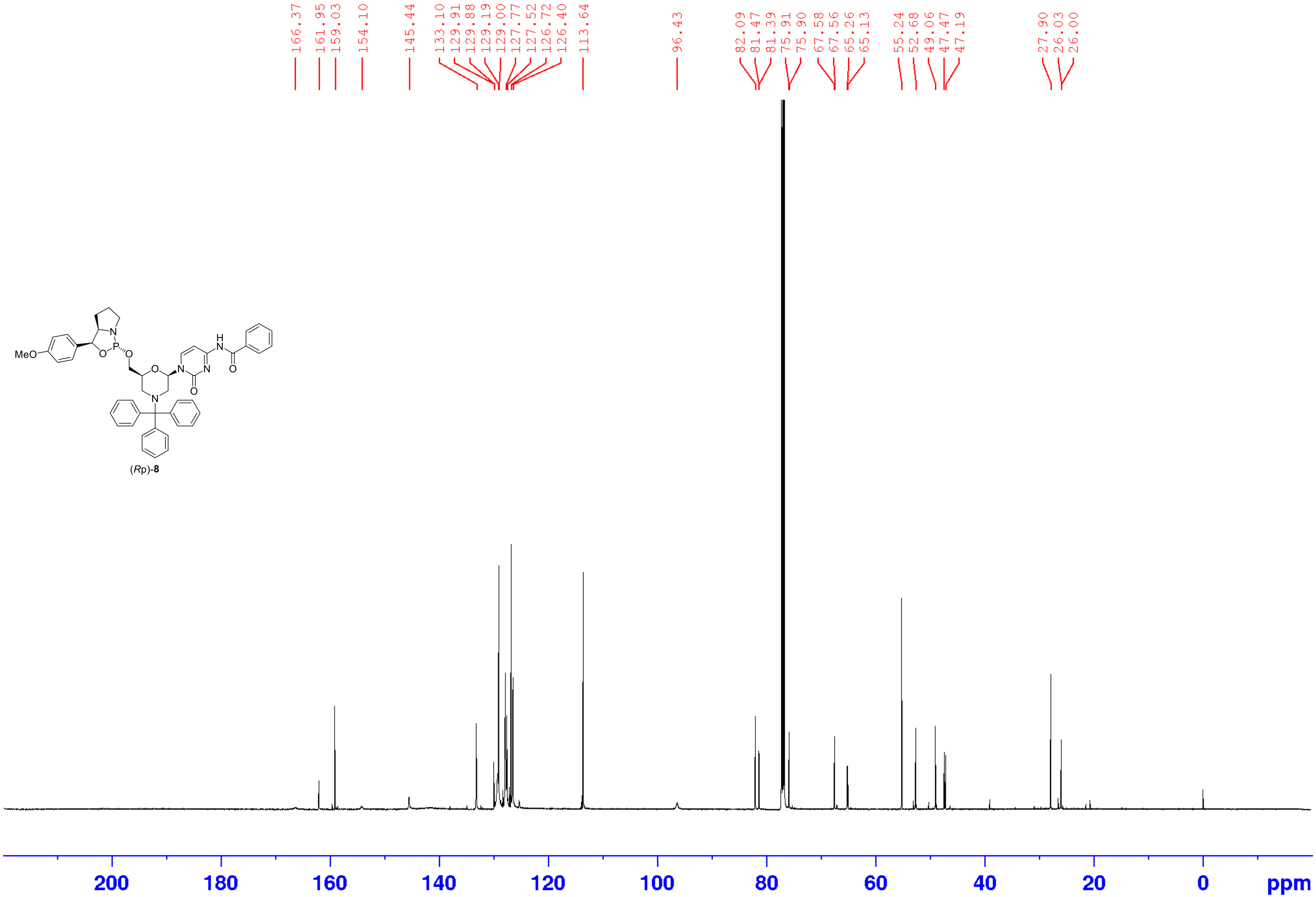

^{31}P {^{1}H} NMR (202 MHz, $CDCl_3$) of (*R*p)-**8**

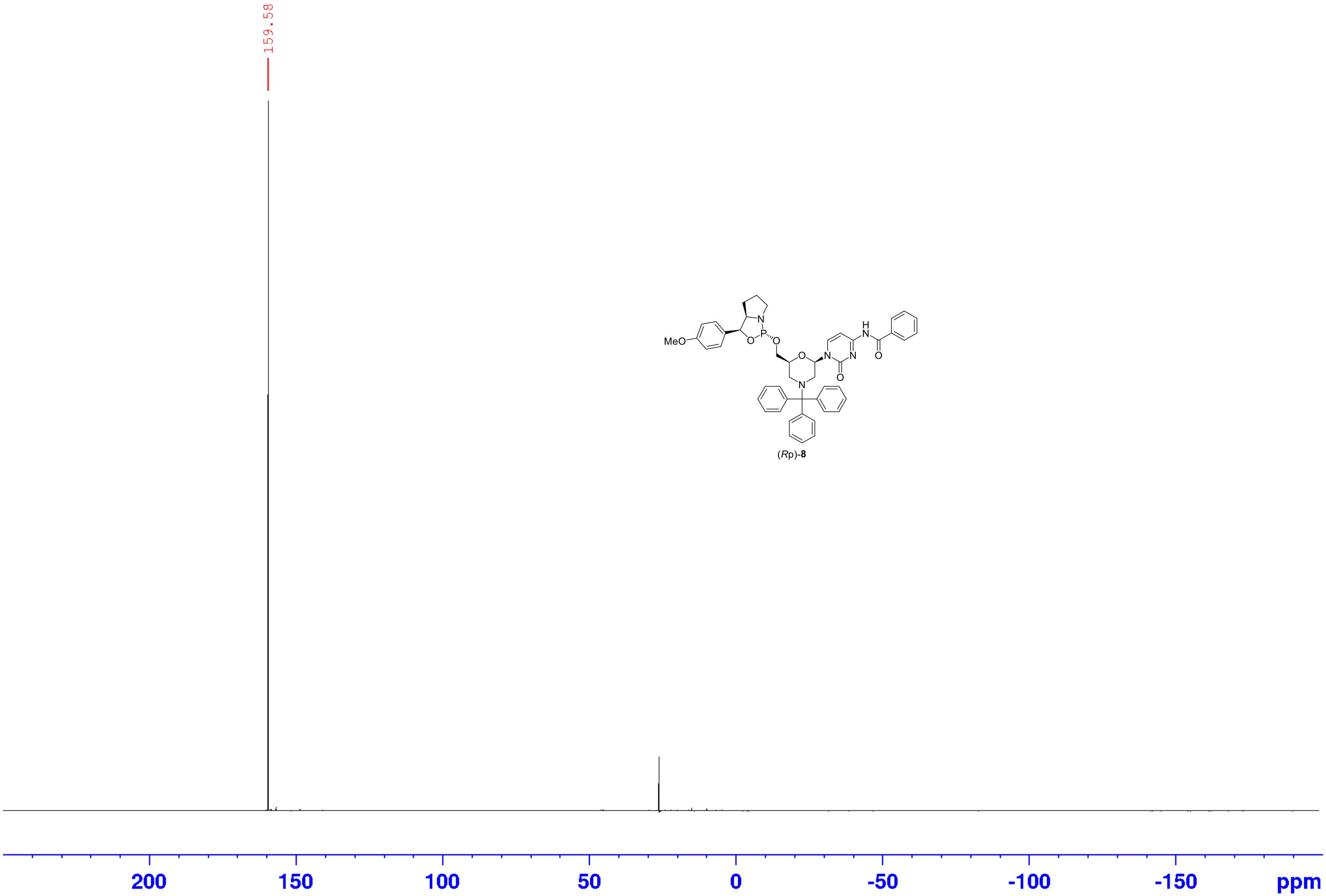

(*R*p)-**8**

COSY ($CDCl_3$) of (*R*p)-**8**

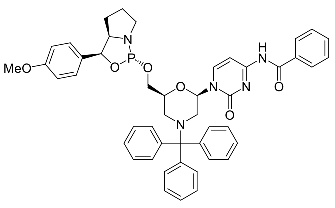
(*R*p)-**8**

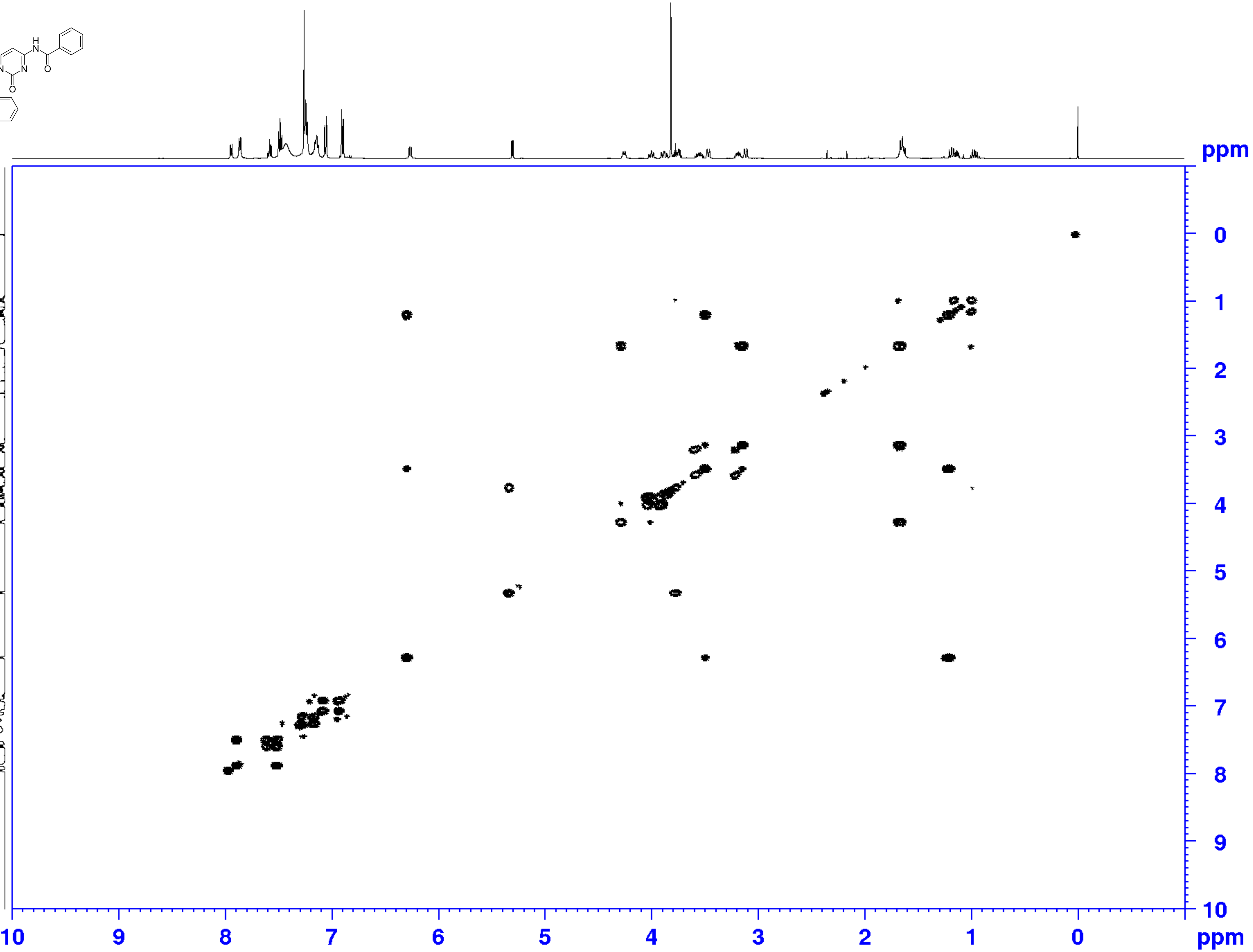

HSQC ($CDCl_3$) of (*R*p)-**8**

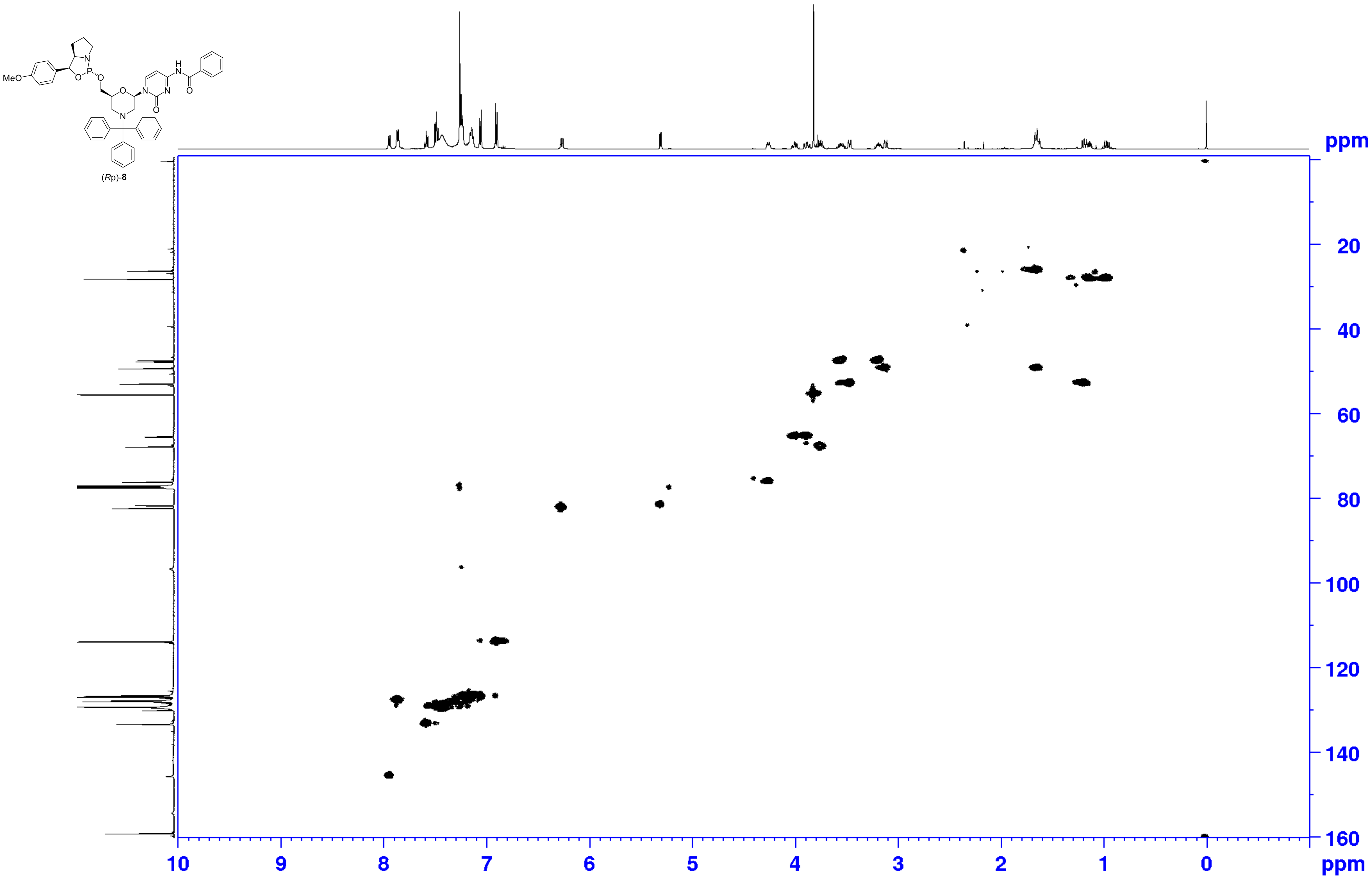

(*R*p)-**8**

HMBC ($CDCl_3$) of (*R*p)-**8**

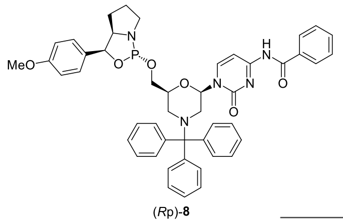

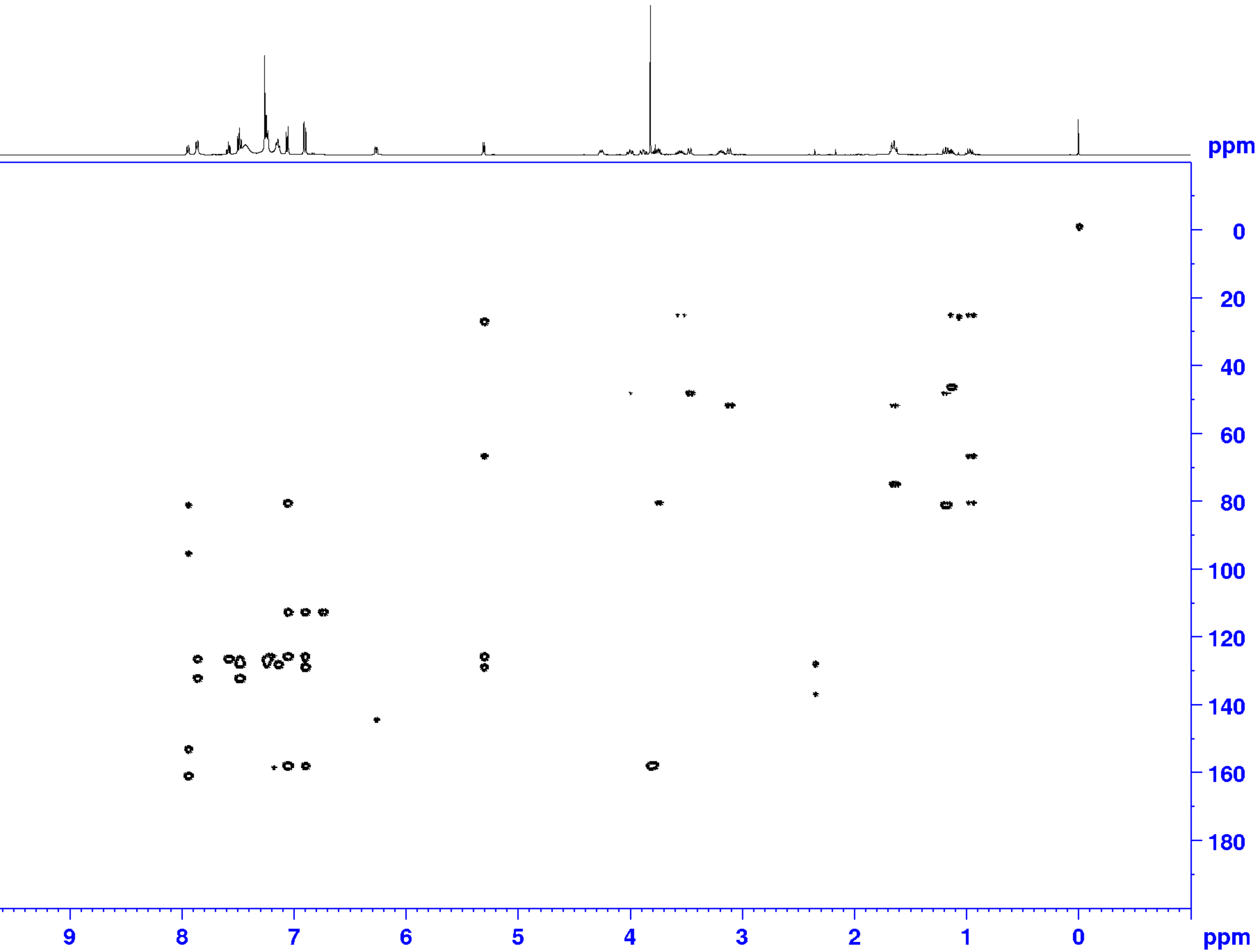

^{1}H NMR (500 MHz, $CDCl_3$) of (*S*p)-**8**

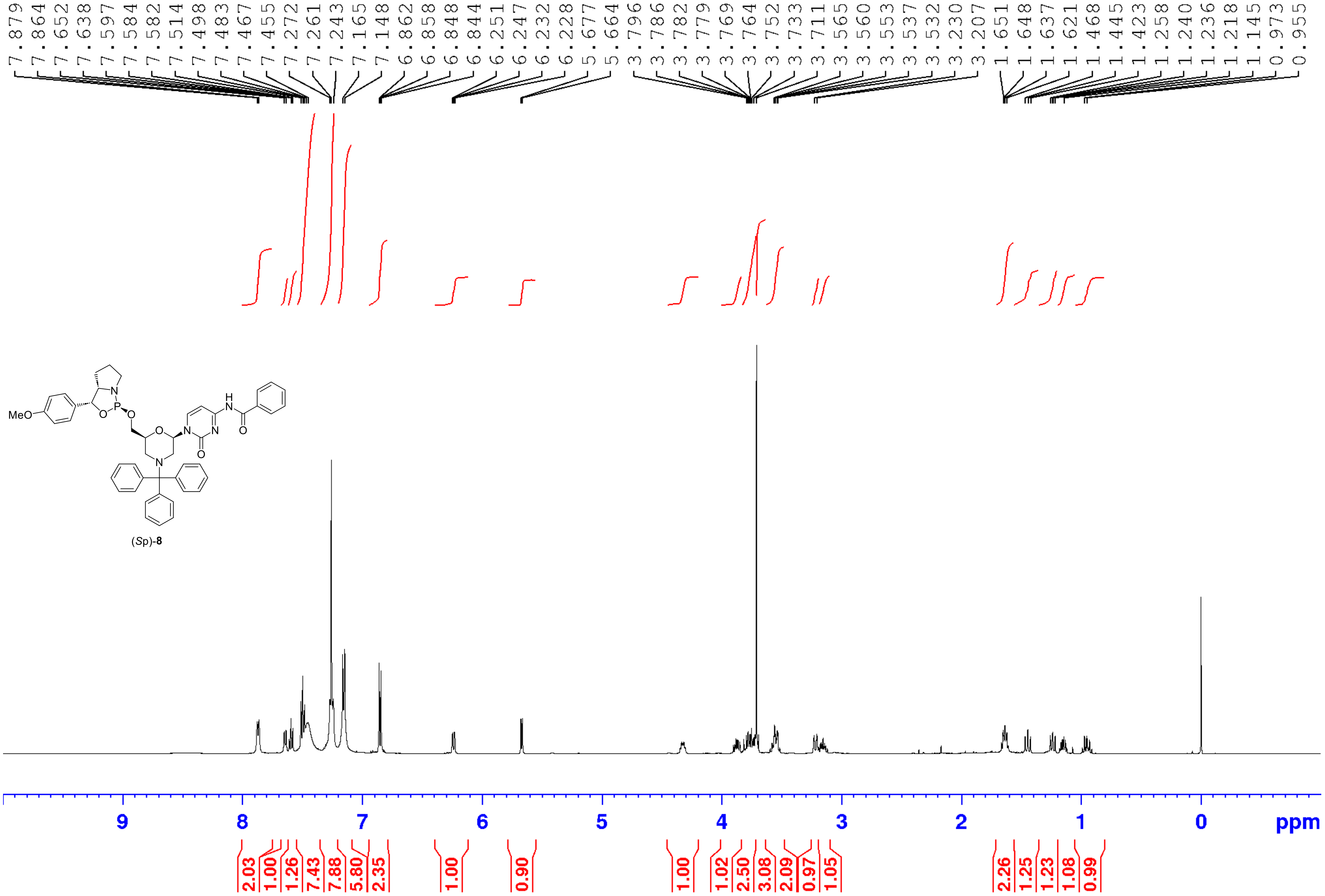

(*S*p)-**8**

$^{13}C\ \{^{1}H\}$ NMR (126 MHz, $CDCl_3$) of (*S*p)-**8**

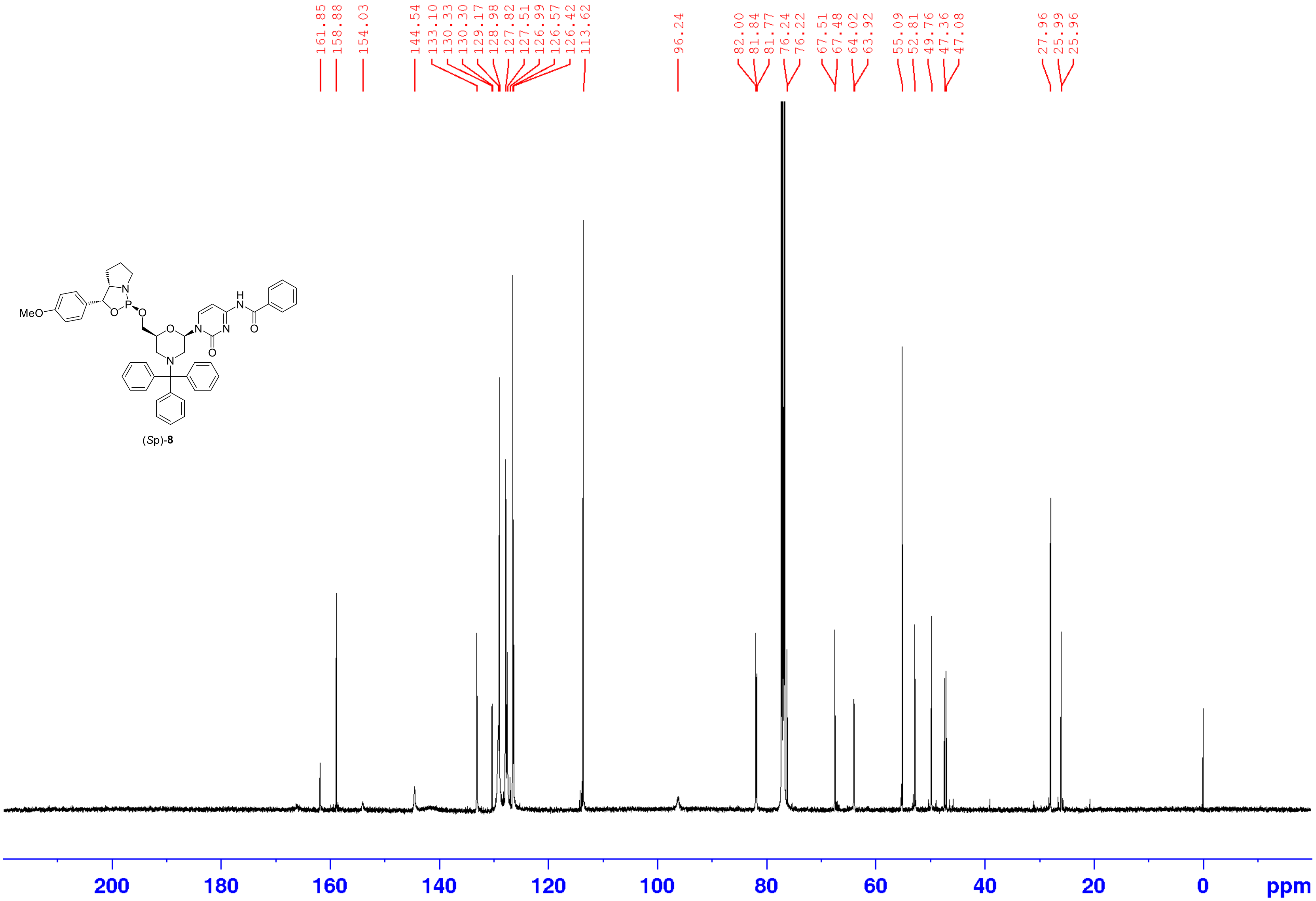

^{31}P {^{1}H} NMR (202 MHz, $CDCl_3$) of (*S*p)-**8**

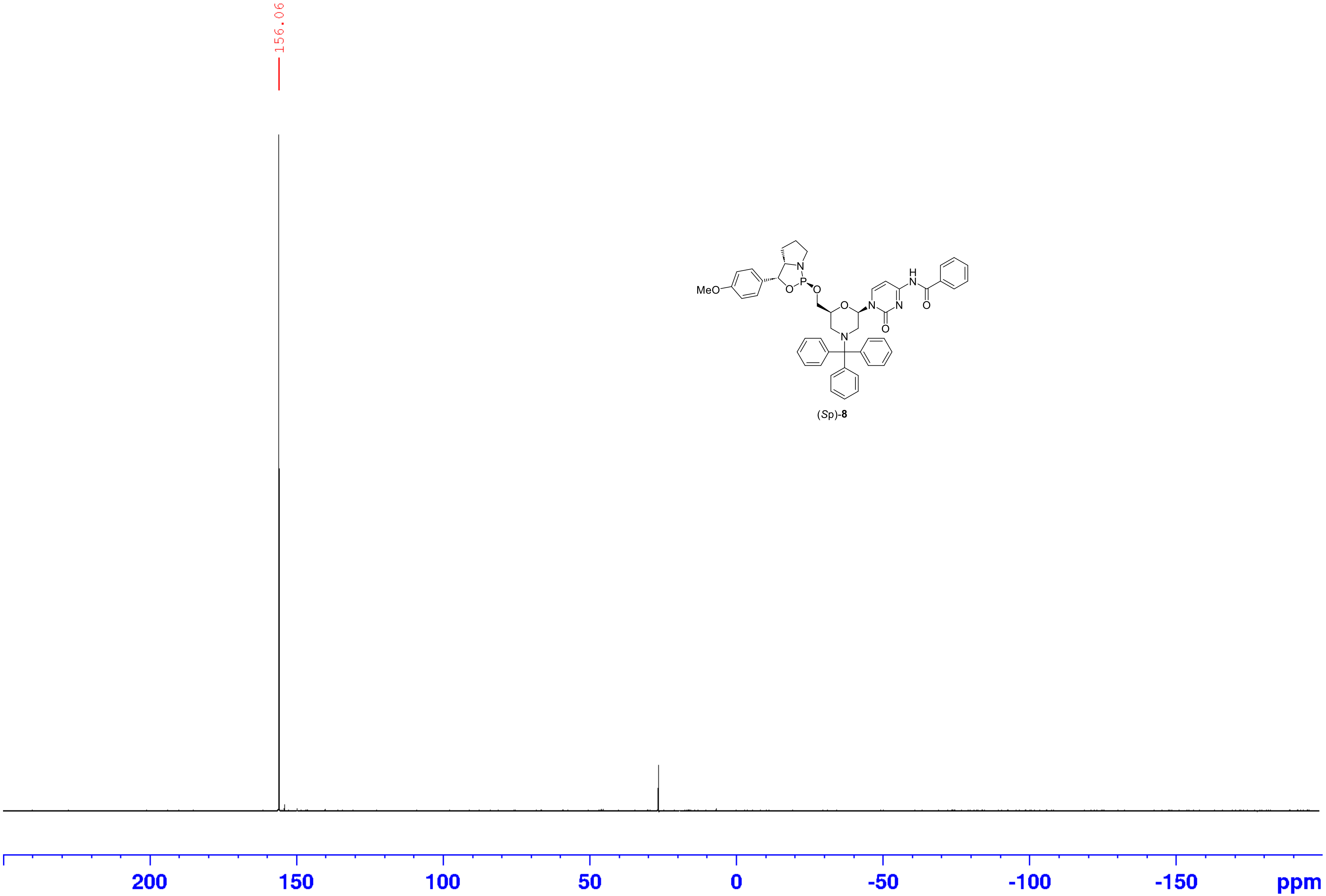

COSY ($CDCl_3$) of (*S*p)-**8**

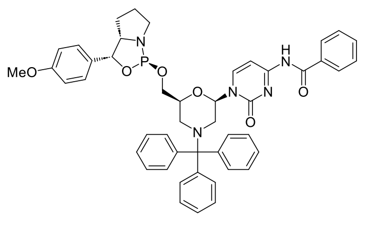

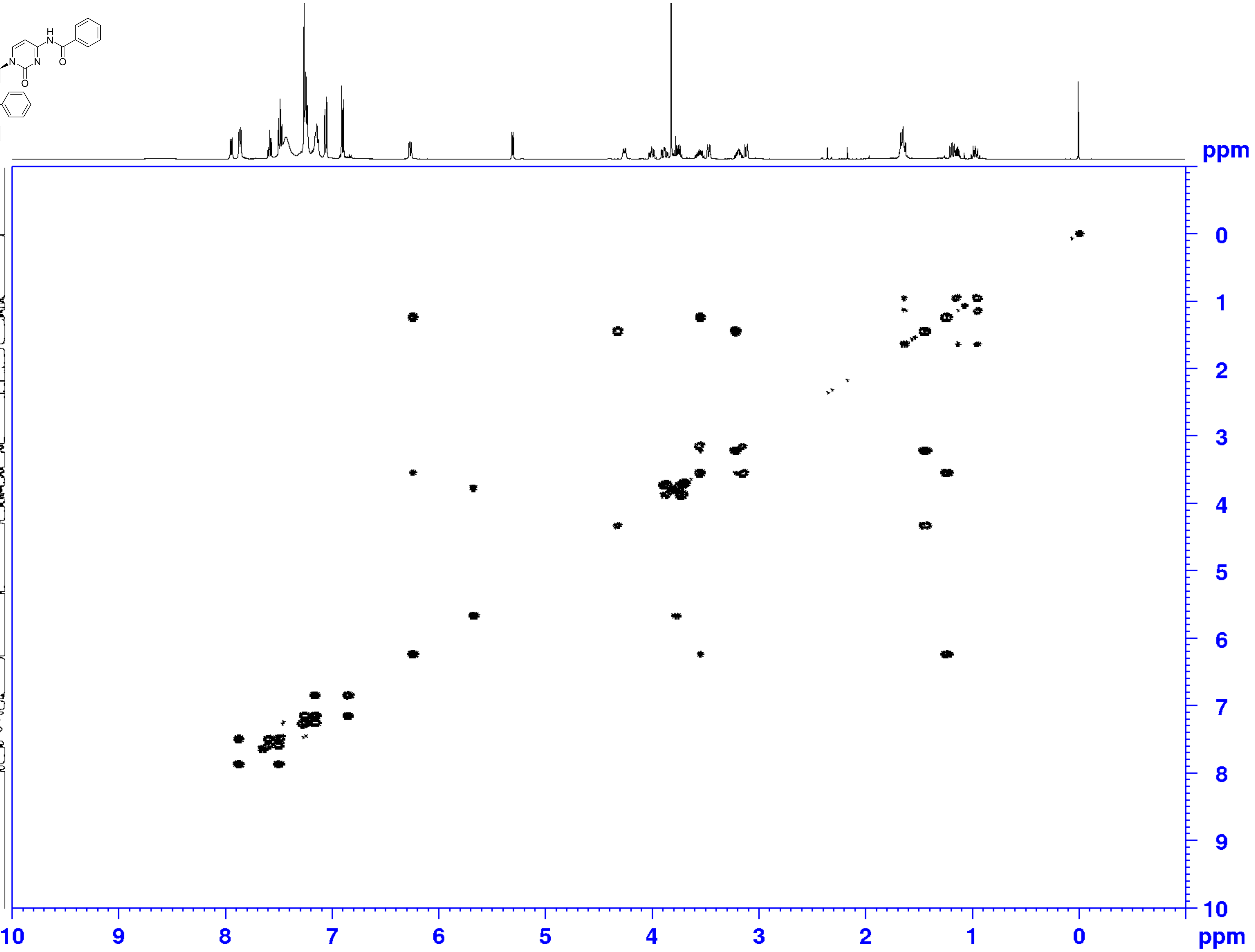

HSQC ($CDCl_3$) of (*S*p)-**8**

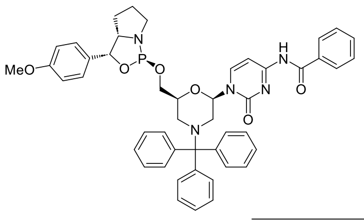

(*S*p)-**8**

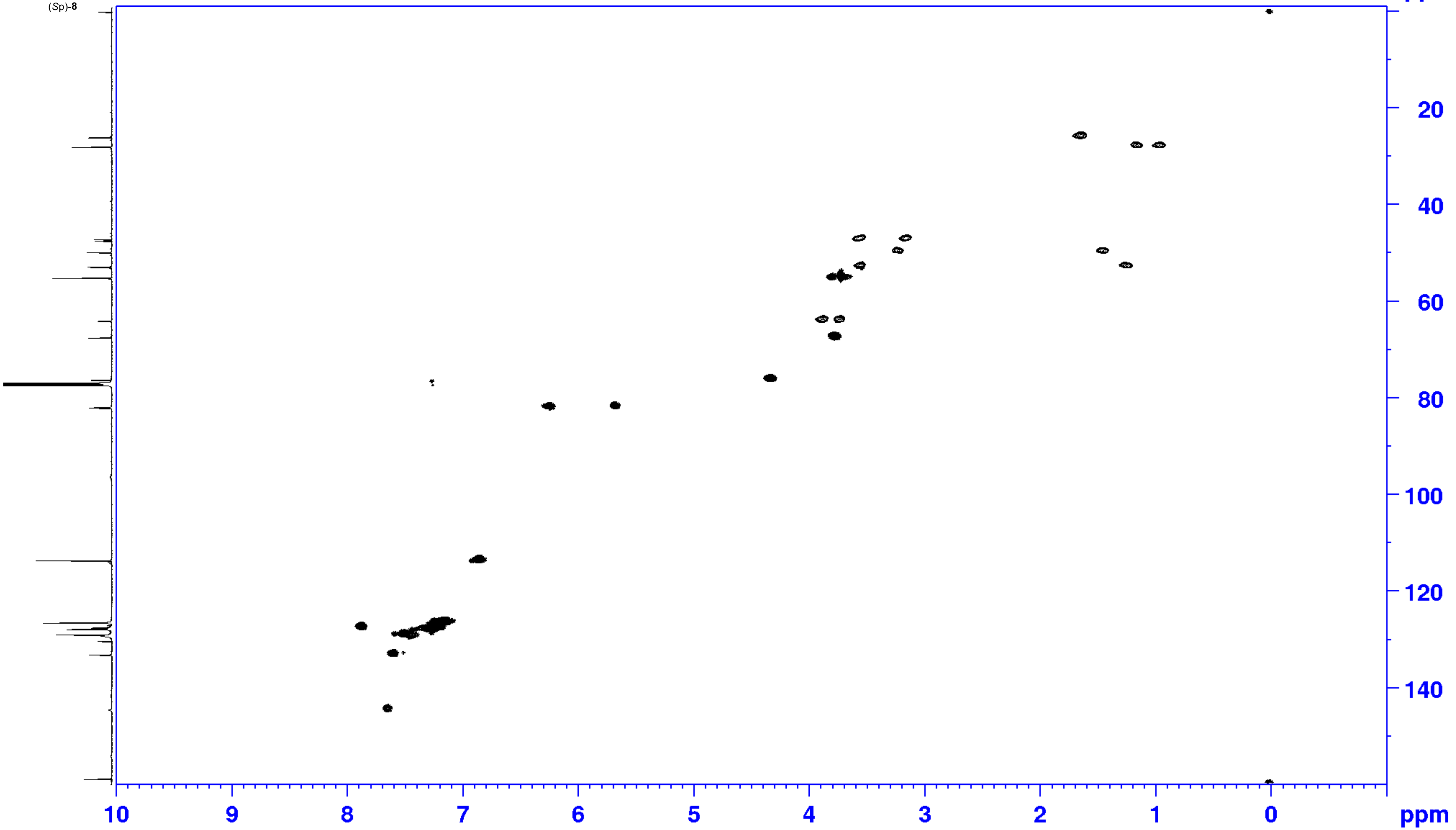

HMBC ($CDCl_3$) of (*S*p)-**8**

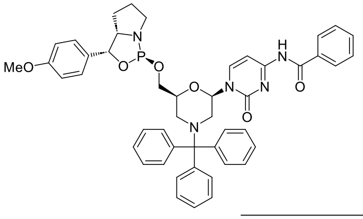

(*S*p)-**8**

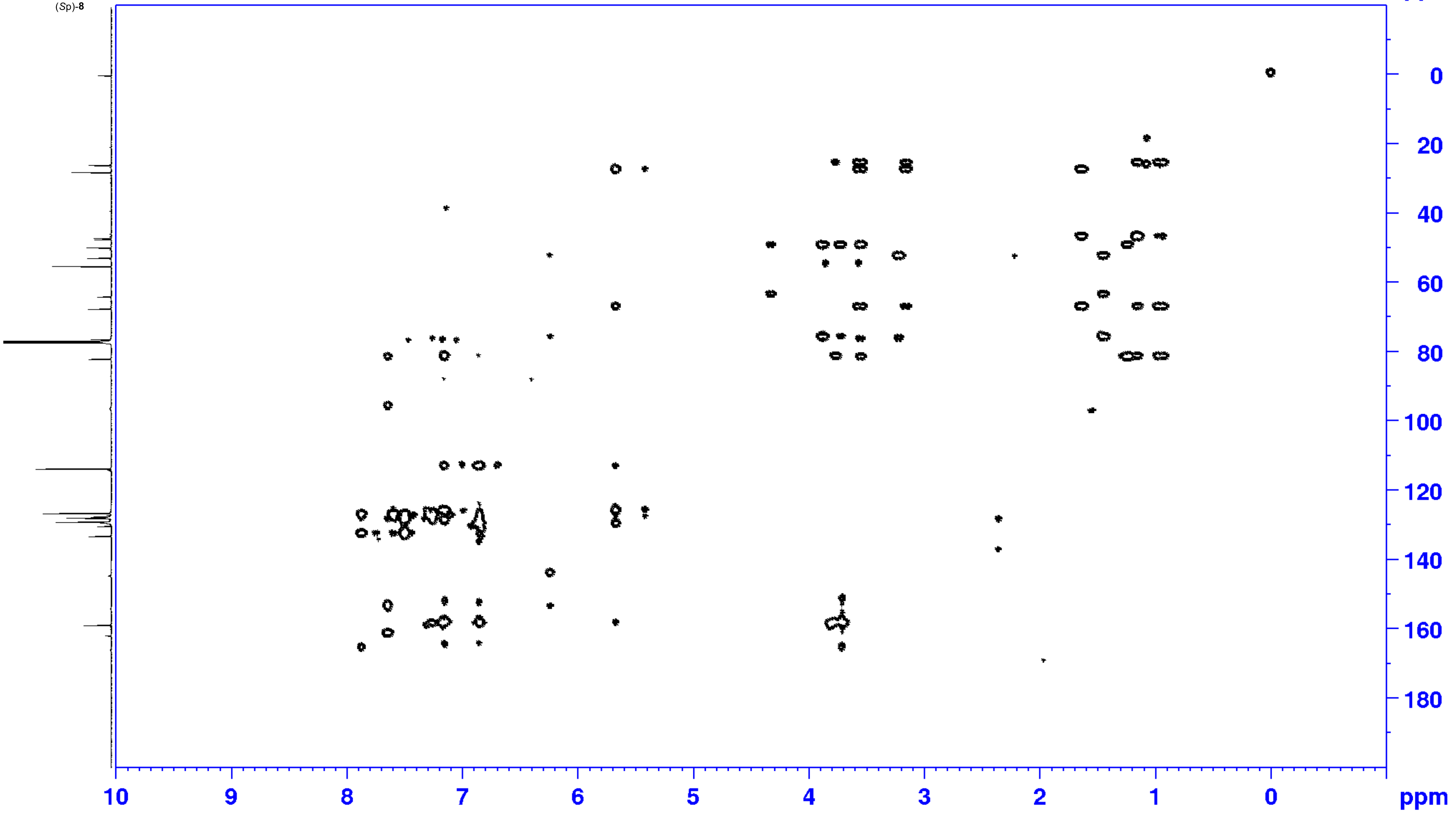

^{1}H NMR (500 MHz, $CDCl_3$) of (*R*p)-**9**

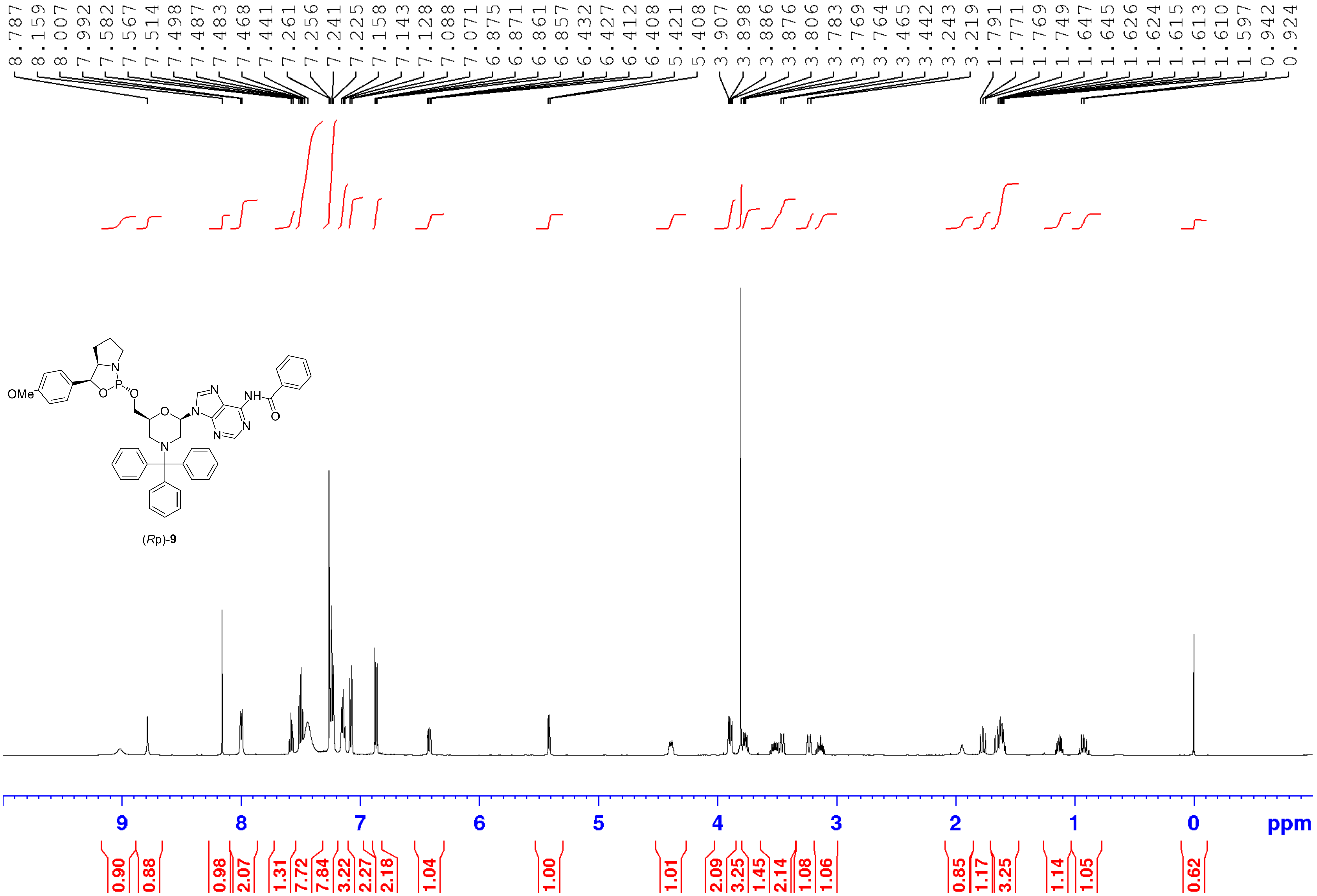

(*R*p)-**9**

$^{13}C\ \{^{1}H\}$ NMR (126 MHz, $CDCl_3$) of (*R*p)-**9**

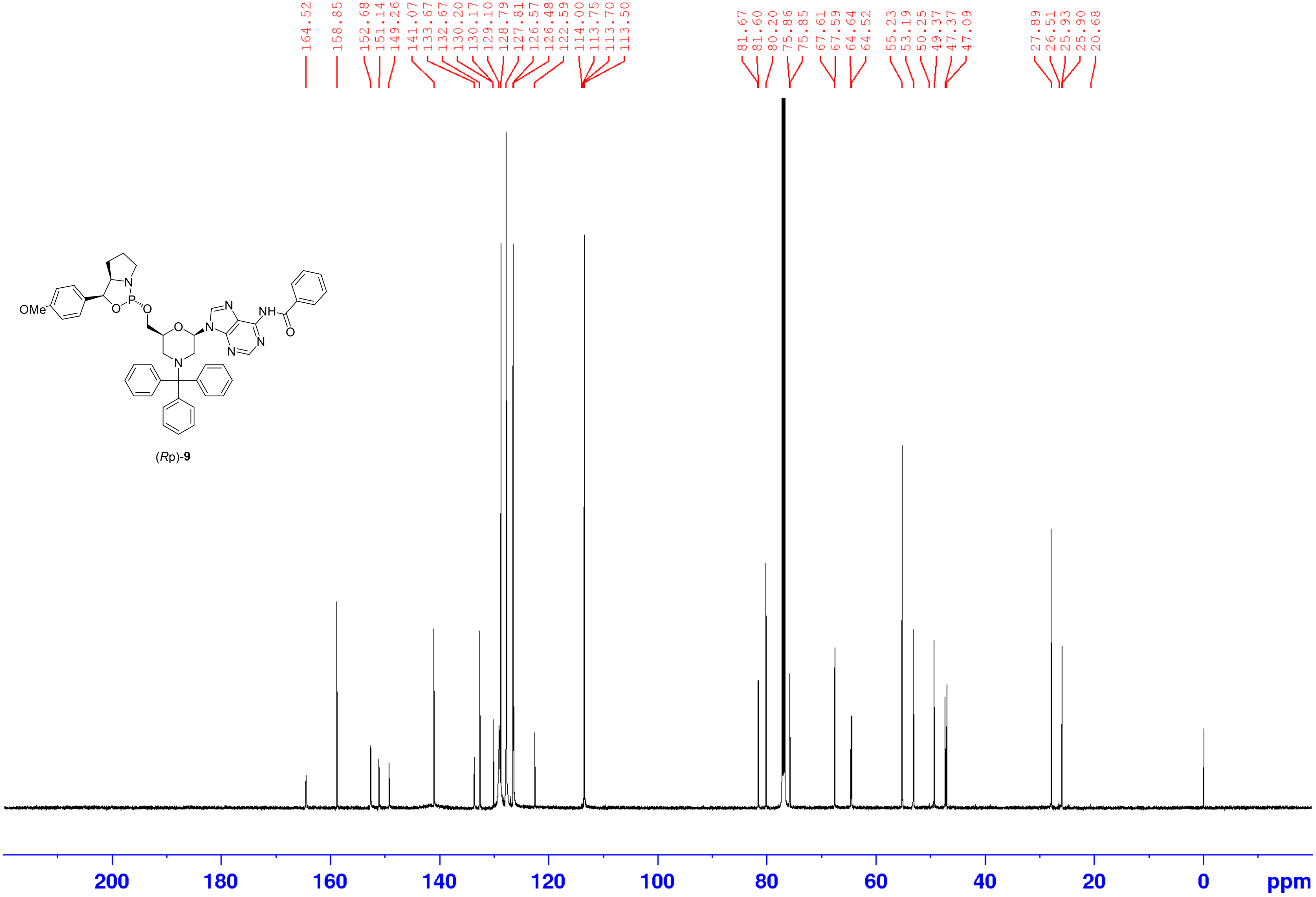

^{31}P {^{1}H} NMR (202 MHz, $CDCl_3$) of (*R*p)-**9**

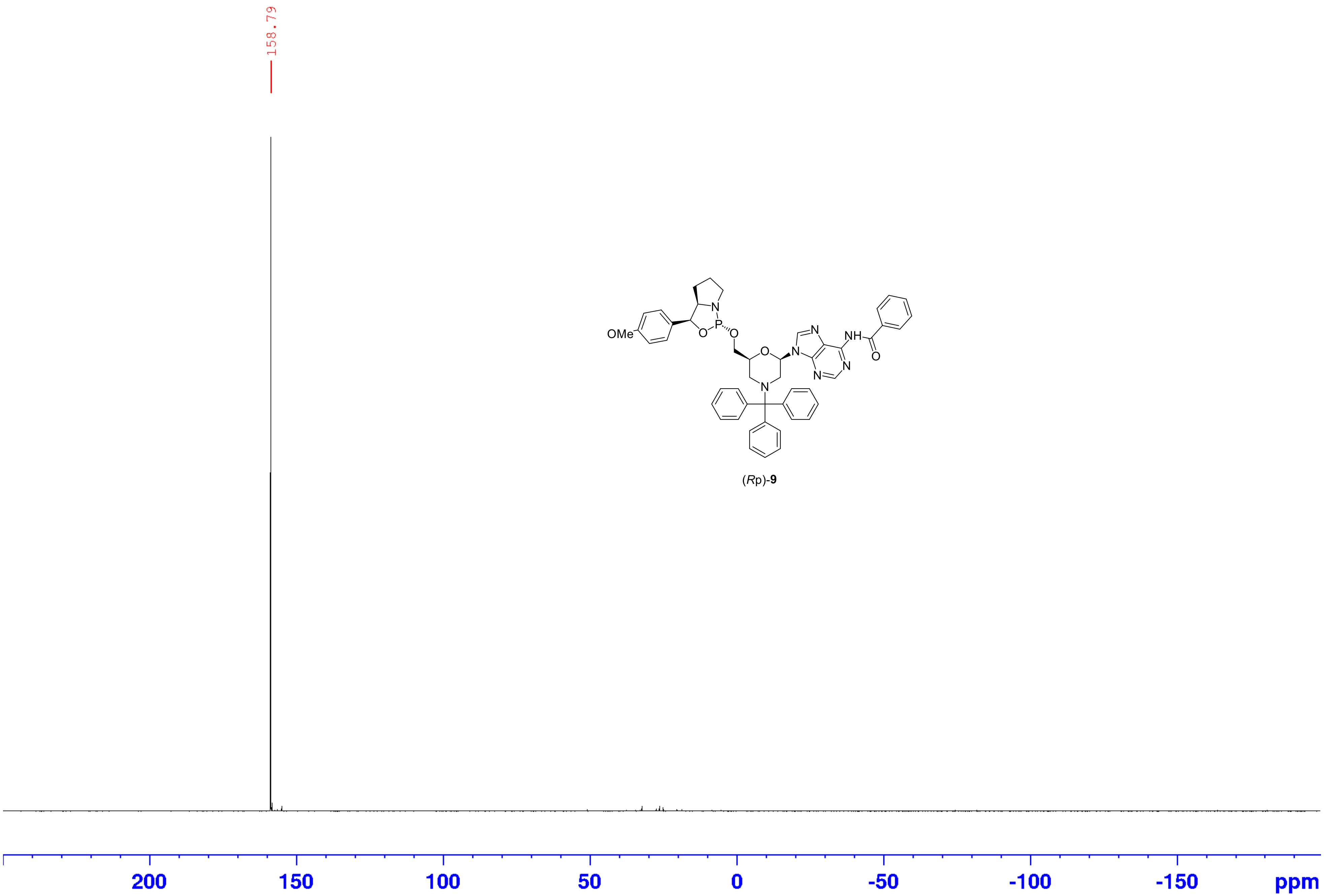

COSY ($CDCl_3$) of (*R*p)-**9**

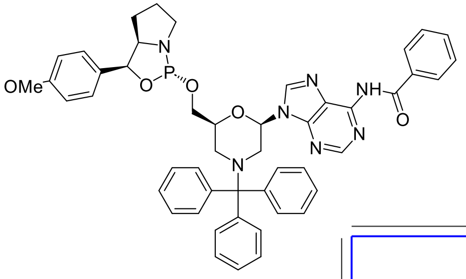

(*R*p)-**9**

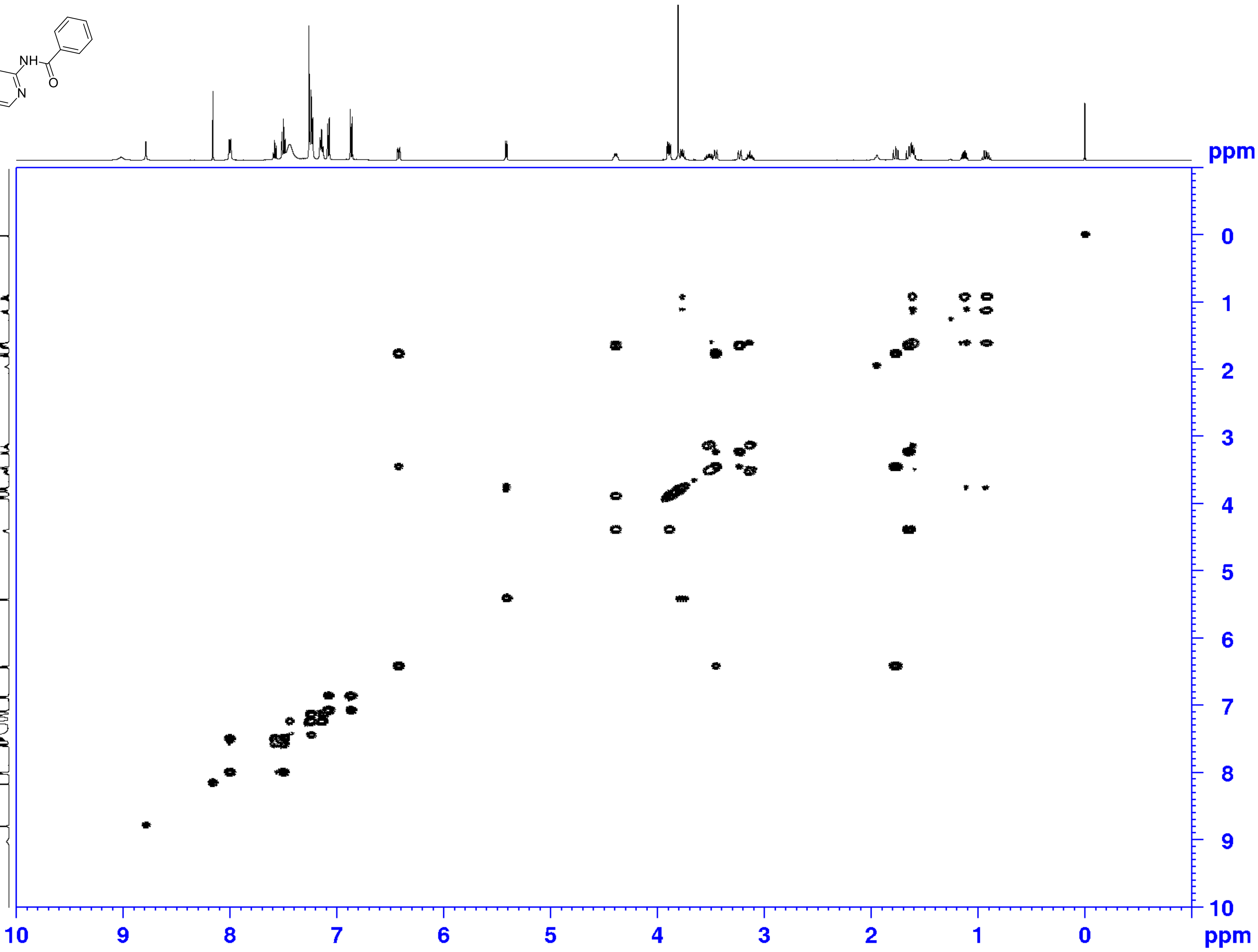

HSQC ($CDCl_3$) of (*R*p)-**9**

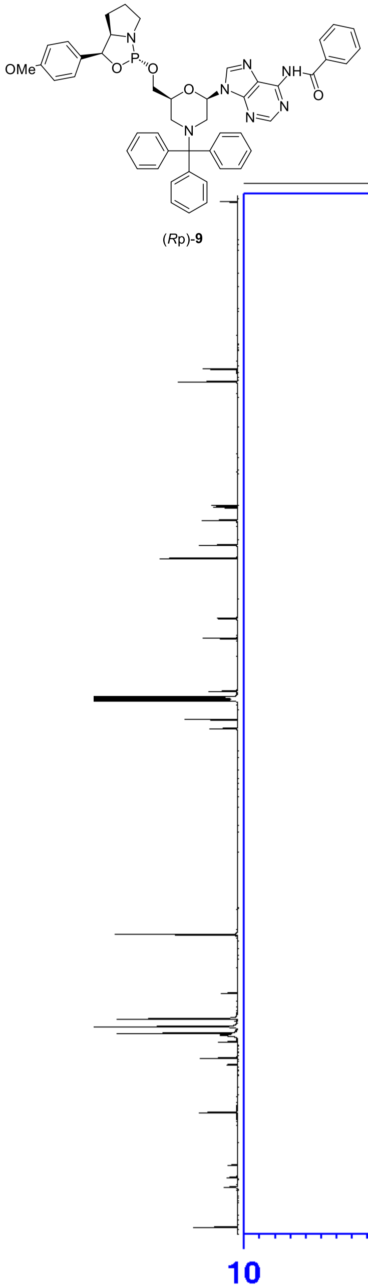

(*R*p)-**9**

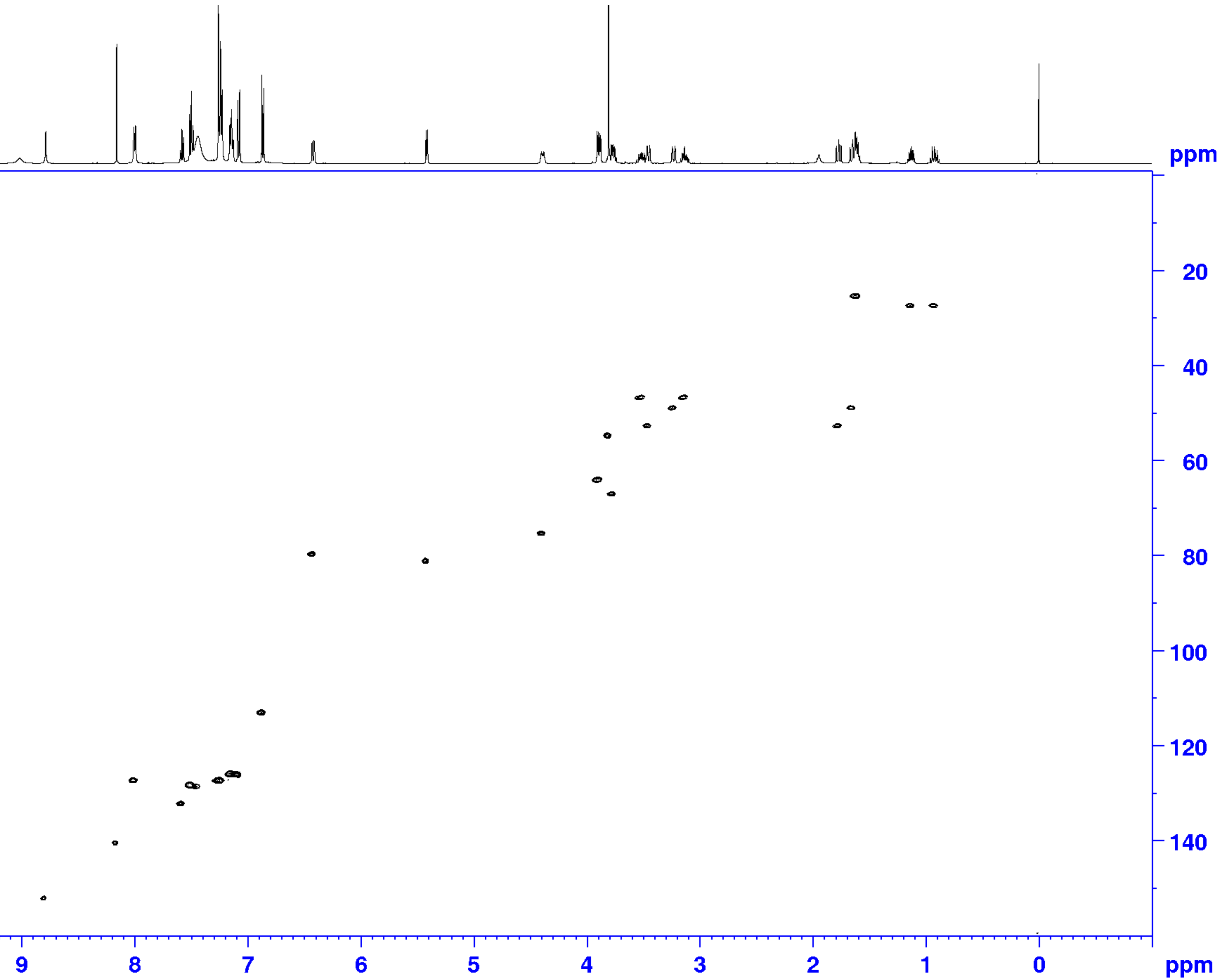

HMBC ($CDCl_3$) of (*R*p)-**9**

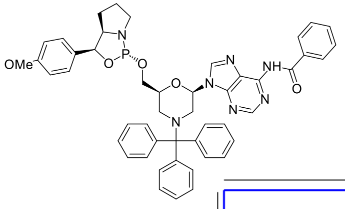

(*R*p)-9

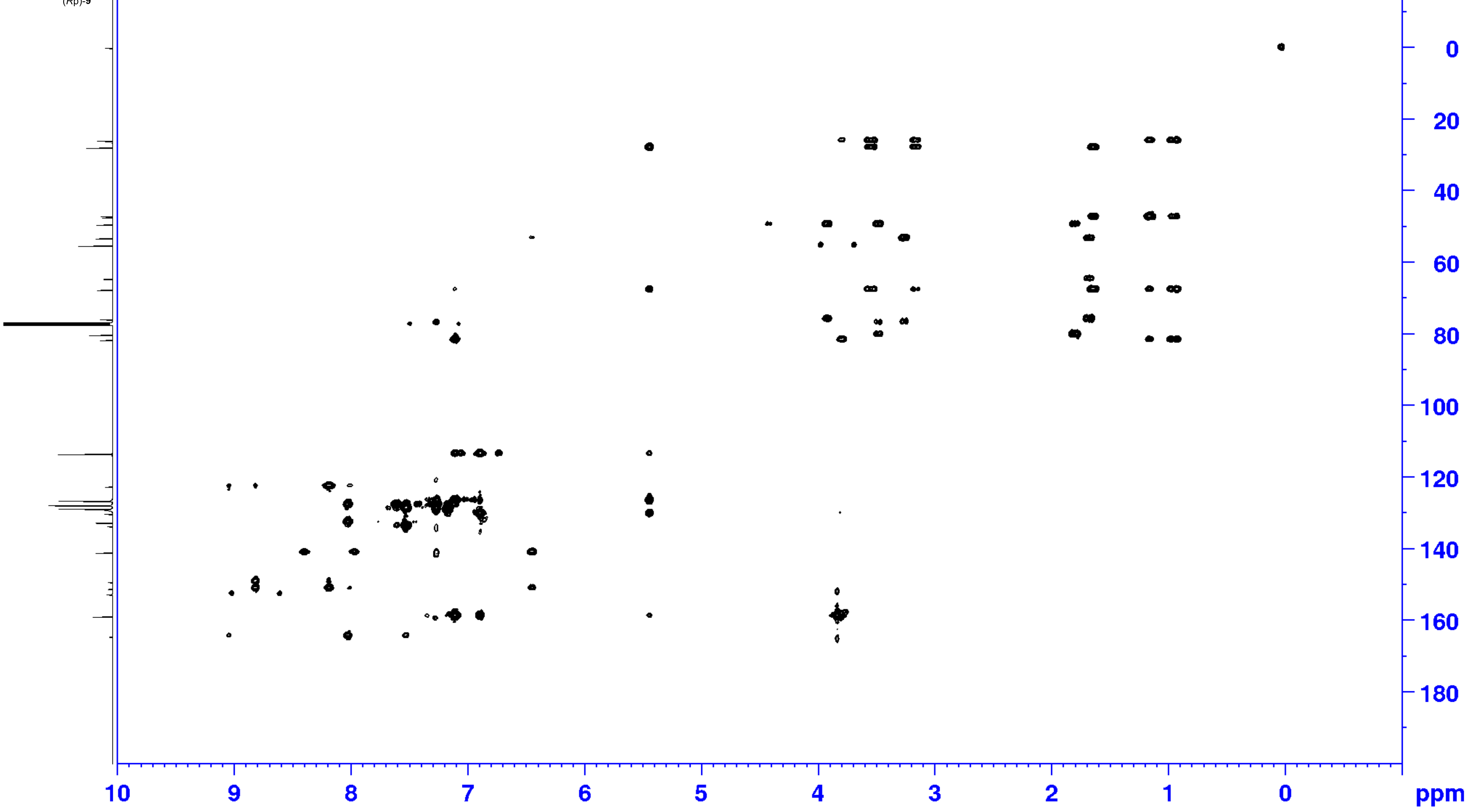

^{1}H NMR (500 MHz, $CDCl_3$) of (*S*p)-**9**

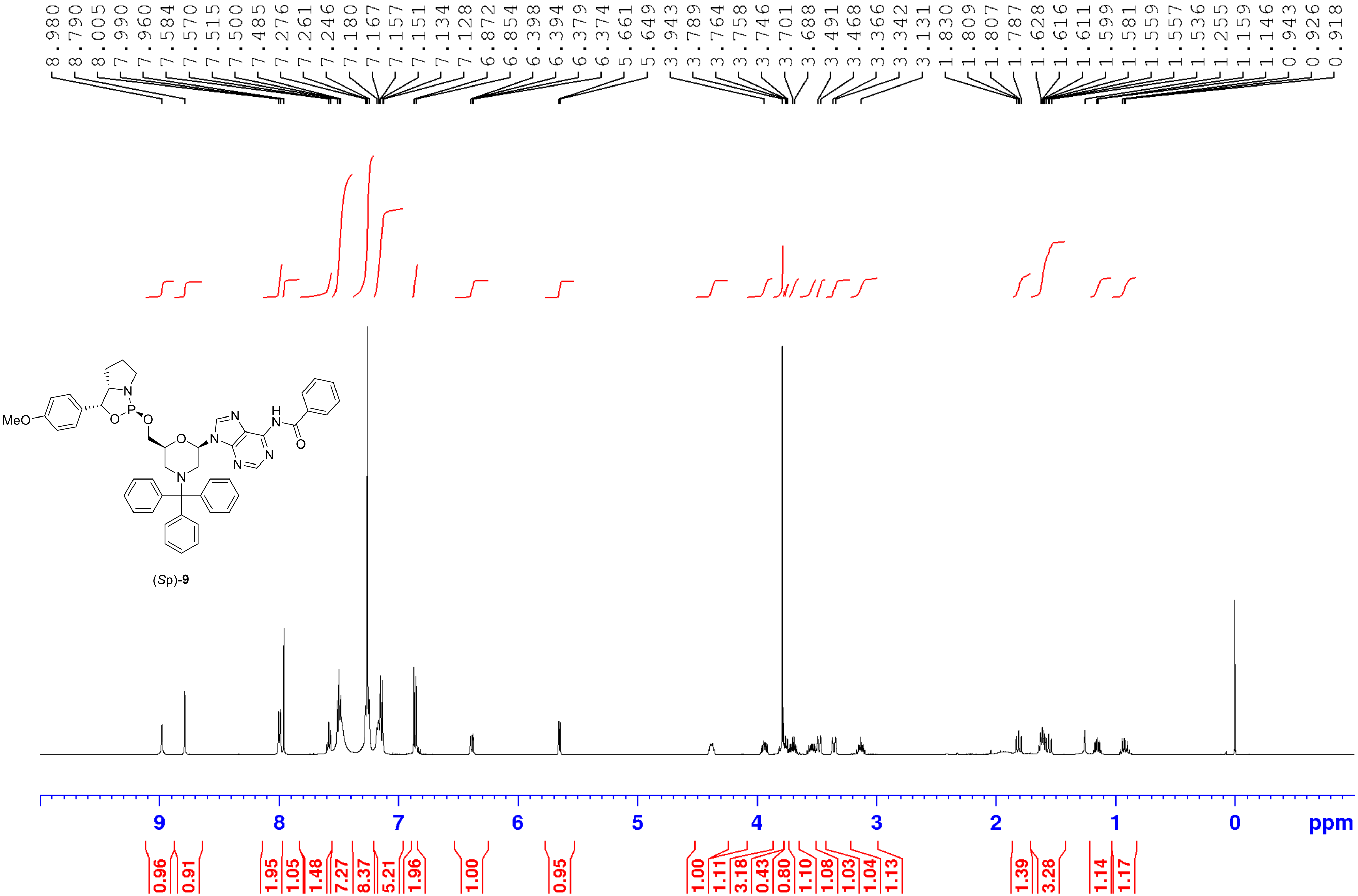

(*S*p)-**9**

$^{13}C\{^{1}H\}$ NMR (126 MHz, $CDCl_3$) of (*S*p)-**9**

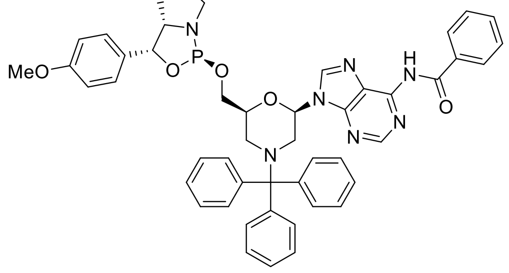

(*S*p)-**9**

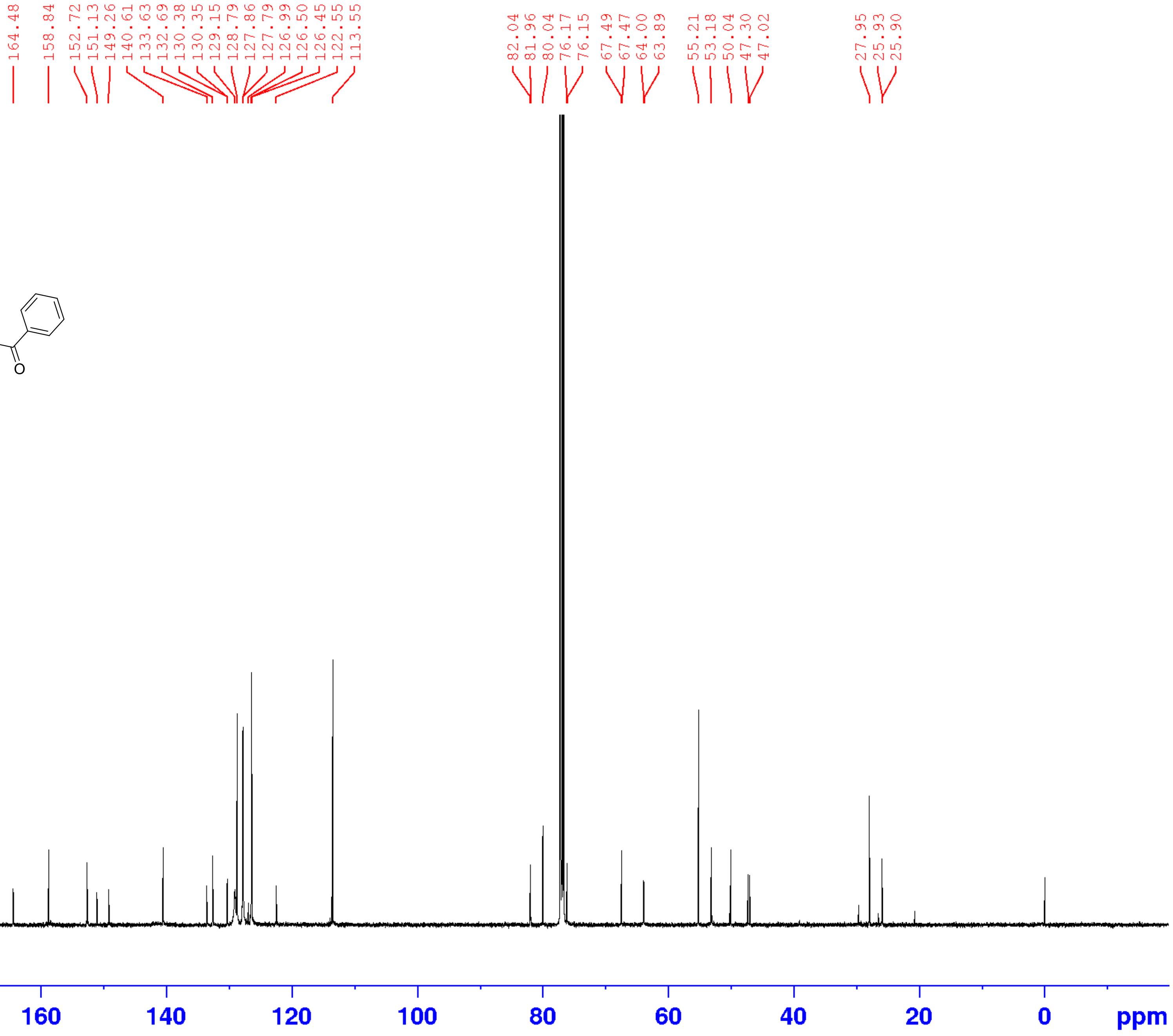

^{31}P {^{1}H} NMR (202 MHz, CDCl$_3$) of (*S*p)-**9**

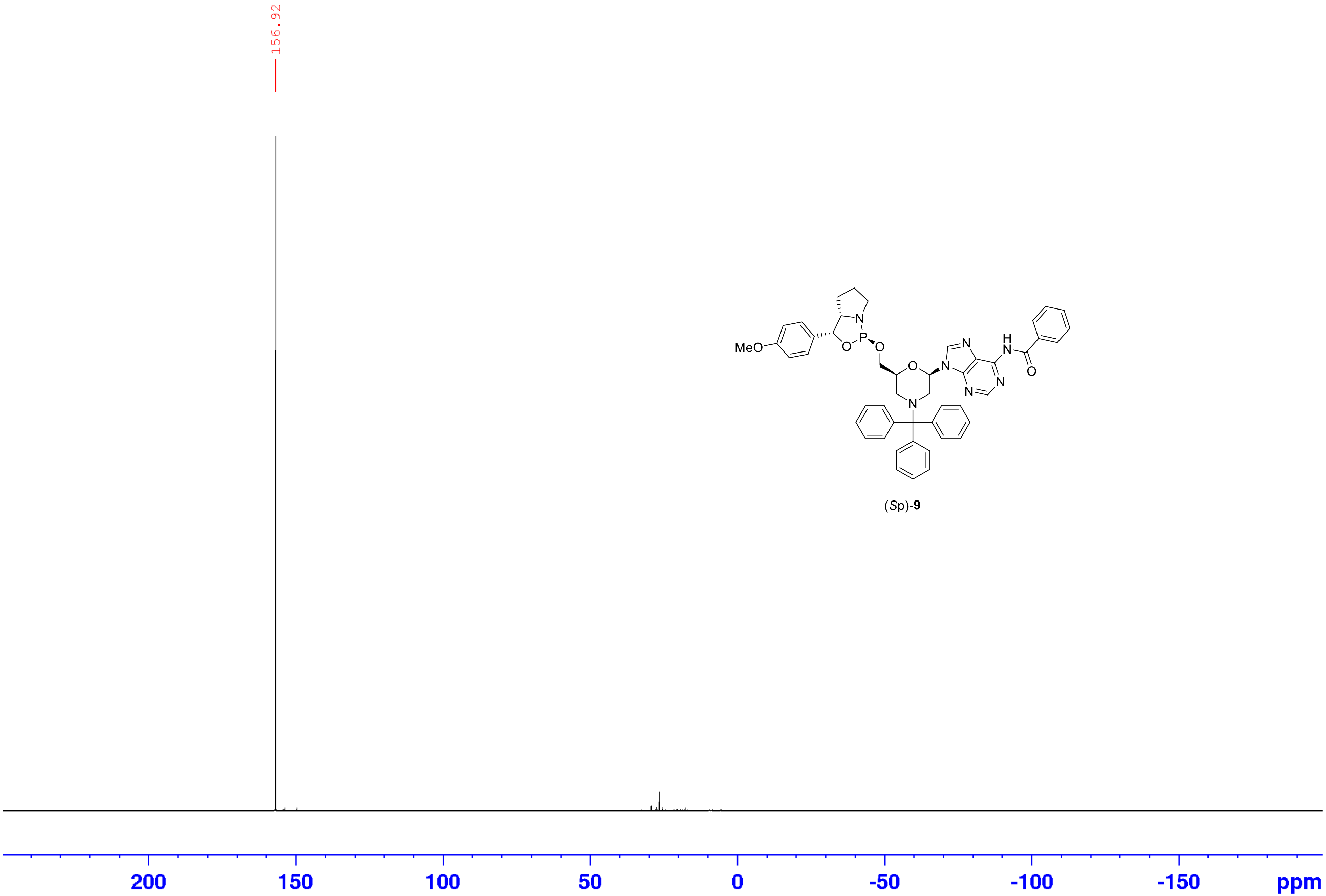

(*S*p)-**9**

COSY ($CDCl_3$) of (*S*p)-**9**

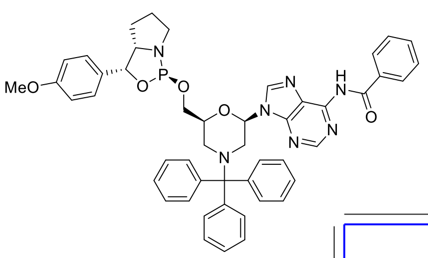

(*S*p)-**9**

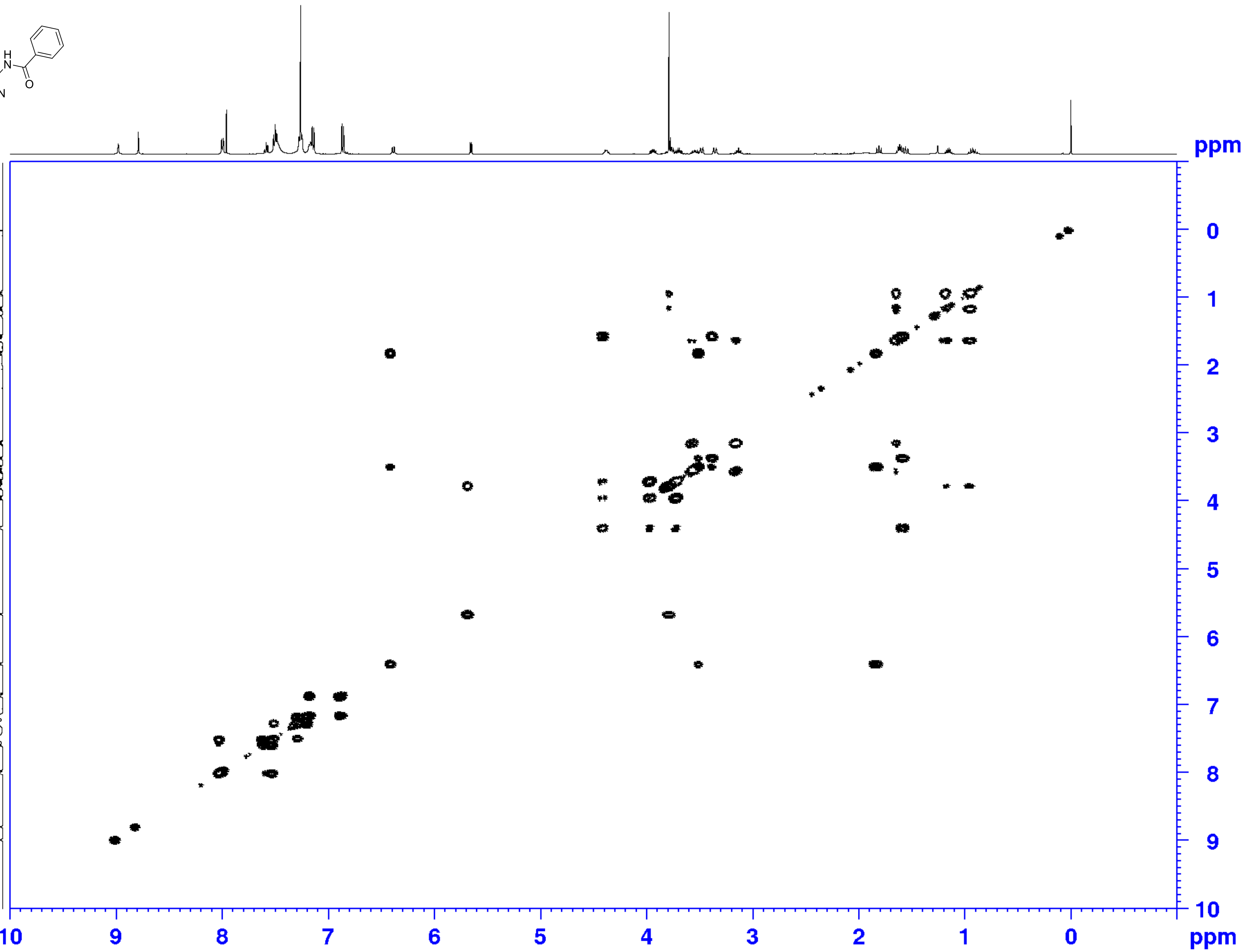

HSQC ($CDCl_3$) of (*S*p)-**9**

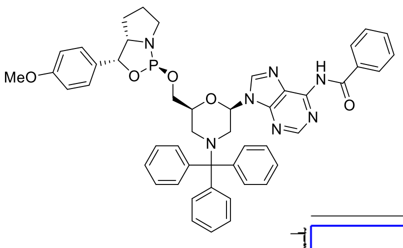

(Sp)-**9**

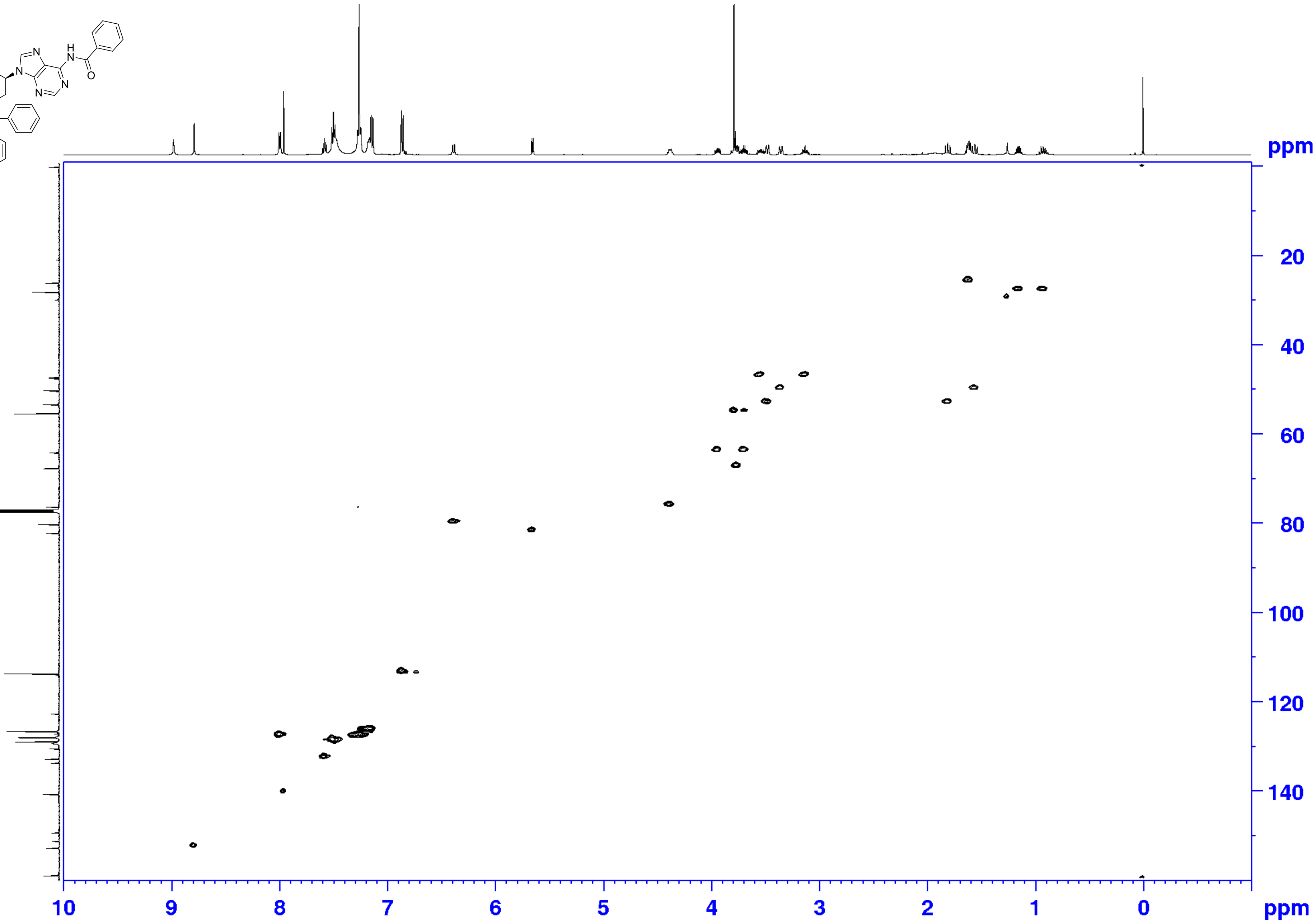

HMBC ($CDCl_3$) of (*S*p)-**9**

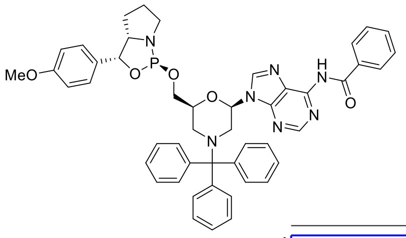

(*S*p)-**9**

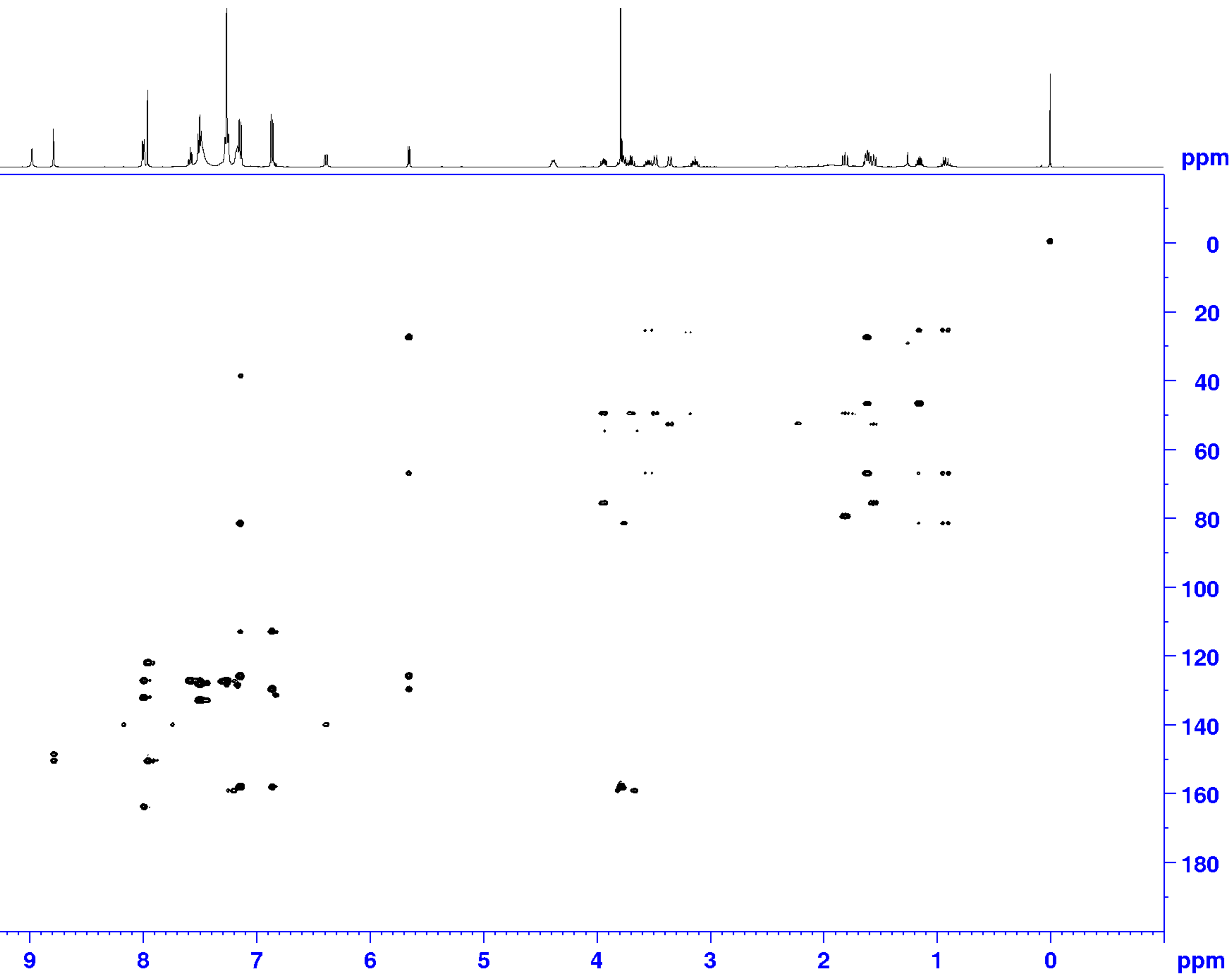

^{1}H NMR (500 MHz, $CDCl_3$) of (*R*p)-**10**

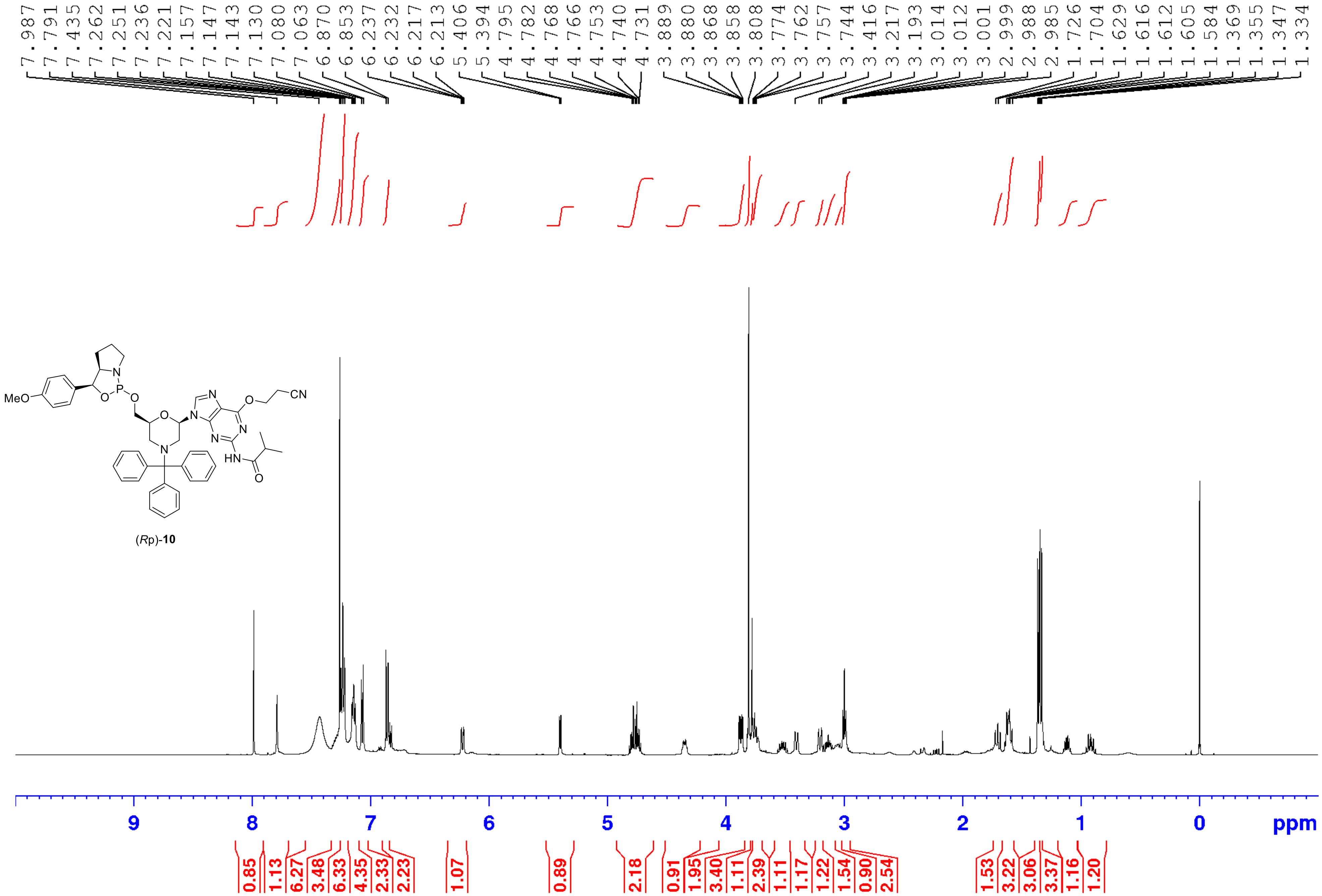

$^{13}C\ \{^{1}H\}$ NMR (126 MHz, $CDCl_3$) of (*R*p)-**10**

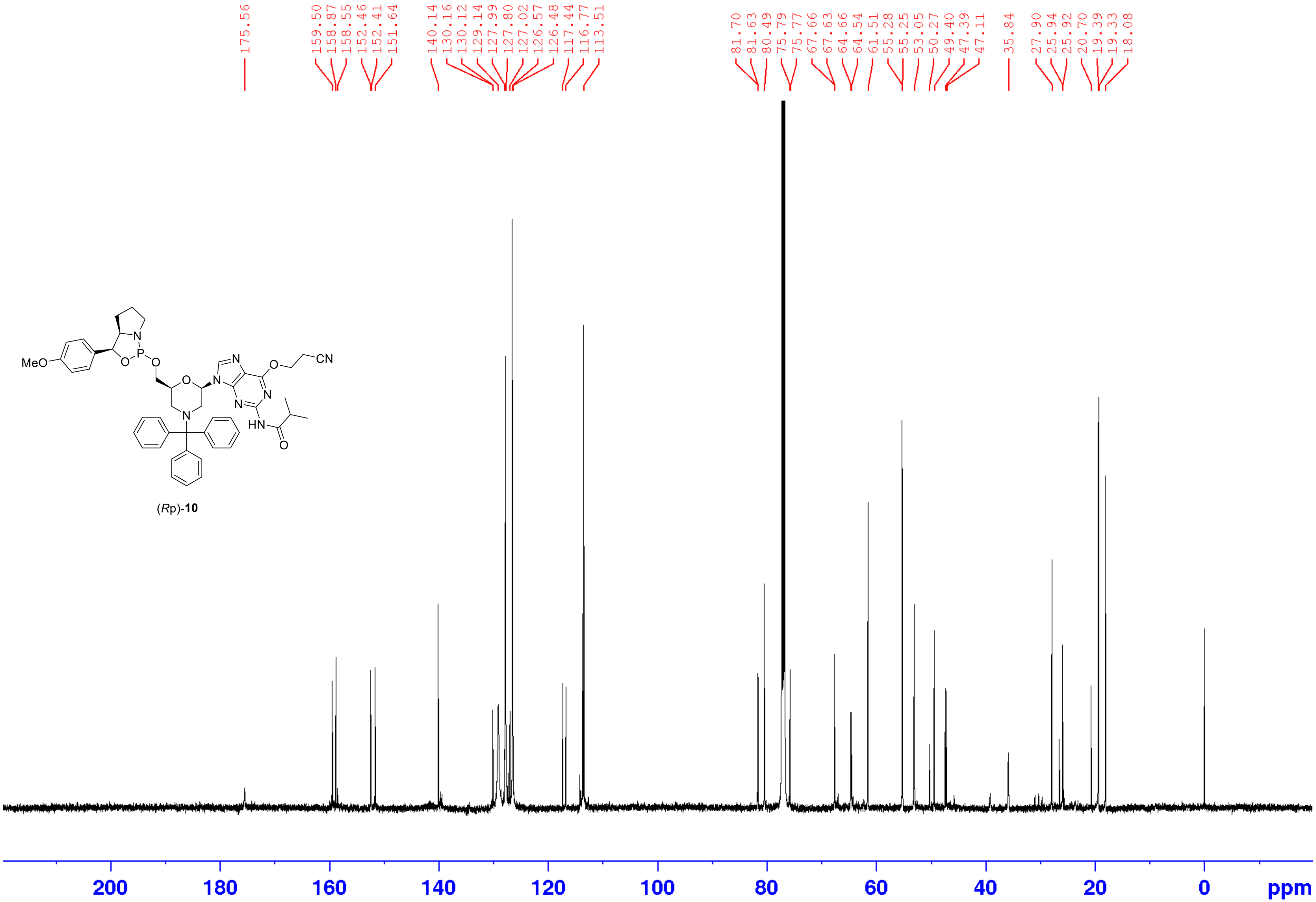

(*R*p)-**10**

^{31}P {^{1}H} NMR (202 MHz, $CDCl_3$) of (*R*p)-**10**

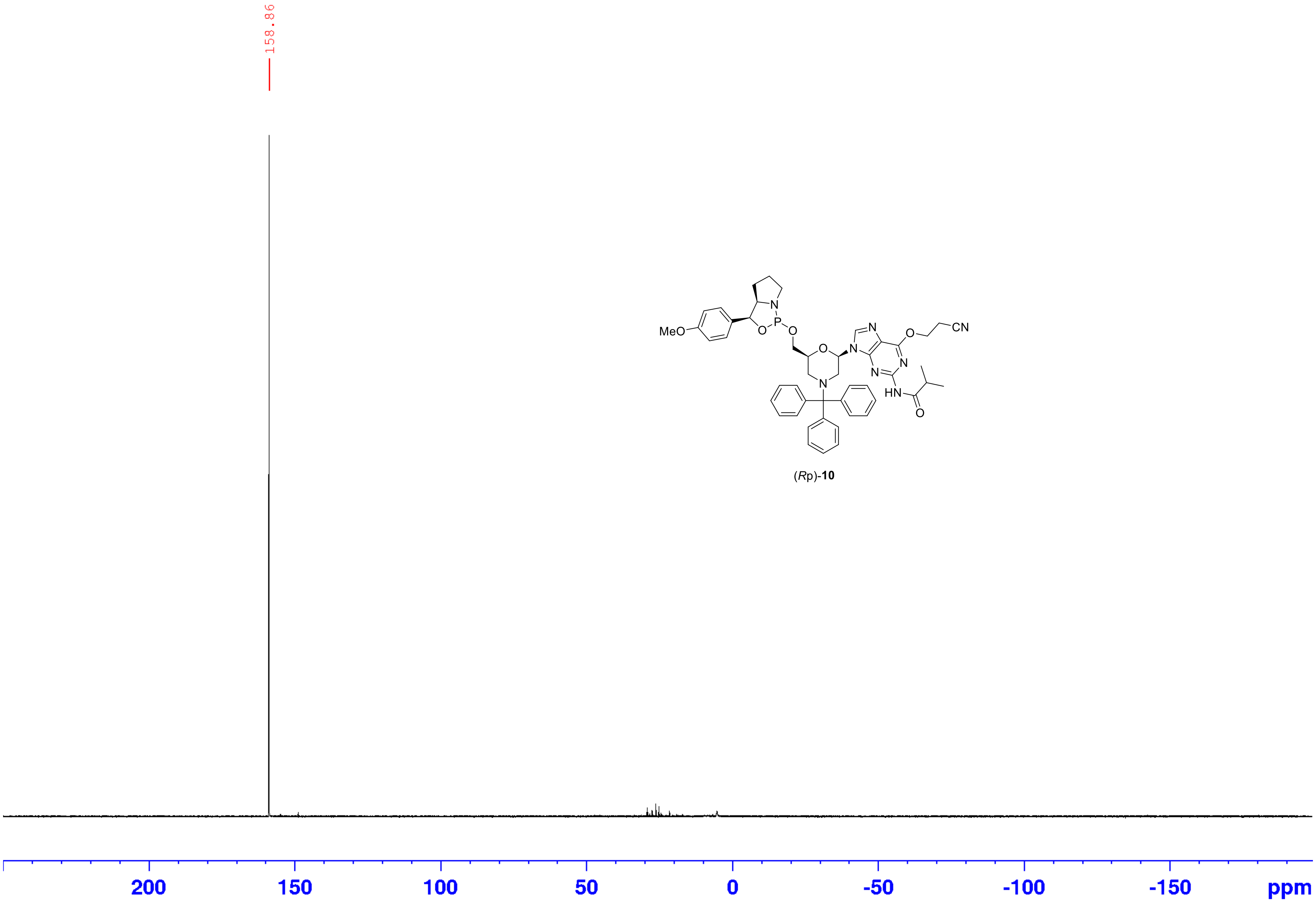

COSY ($CDCl_3$) of (*R*p)-**10**

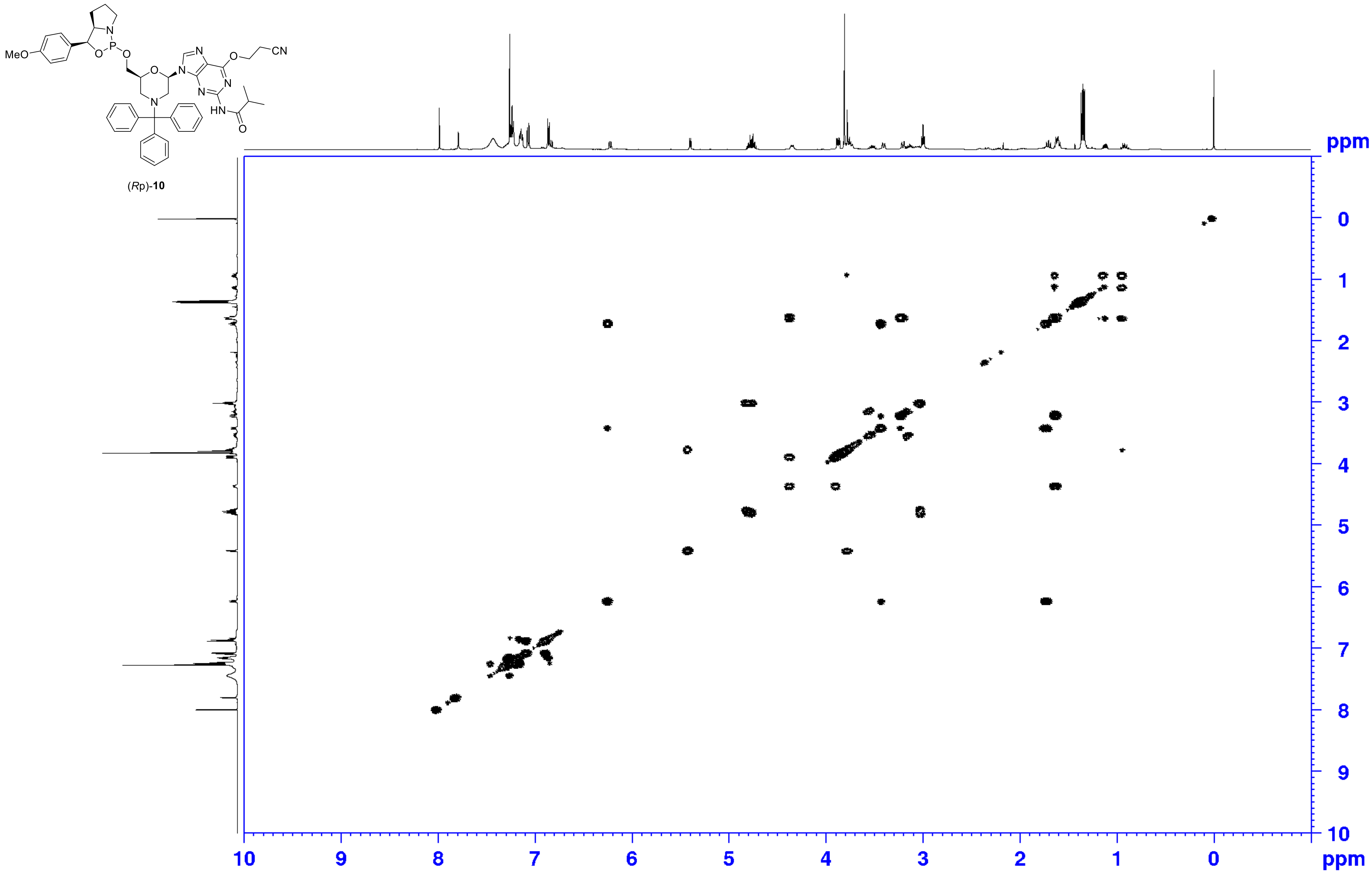

HSQC ($CDCl_3$) of (*R*p)-**10**

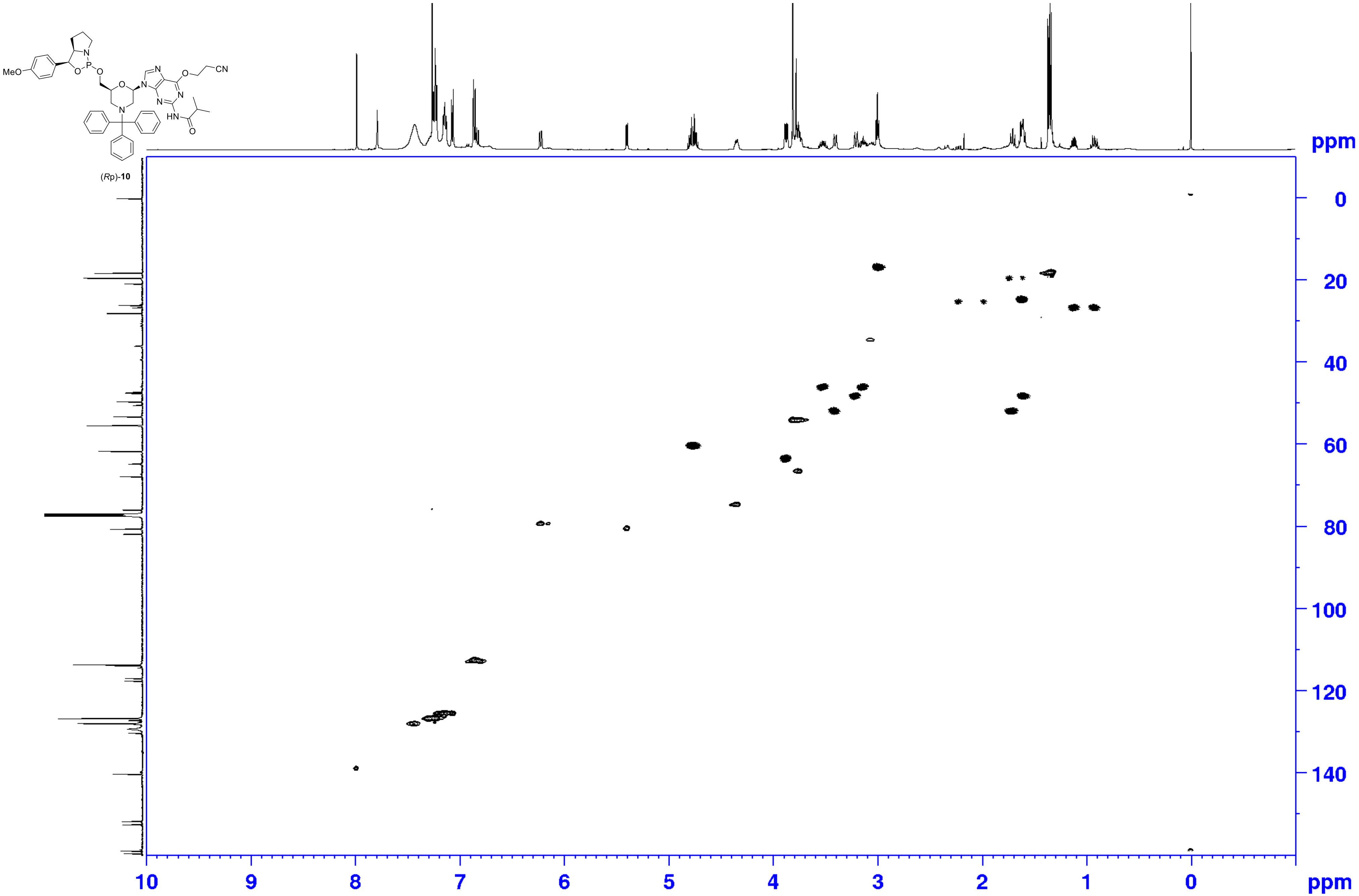

HMBC ($CDCl_3$) of (*R*p)-**10**

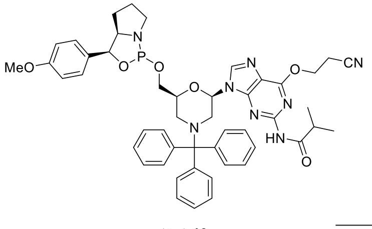

(*R*p)-**10**

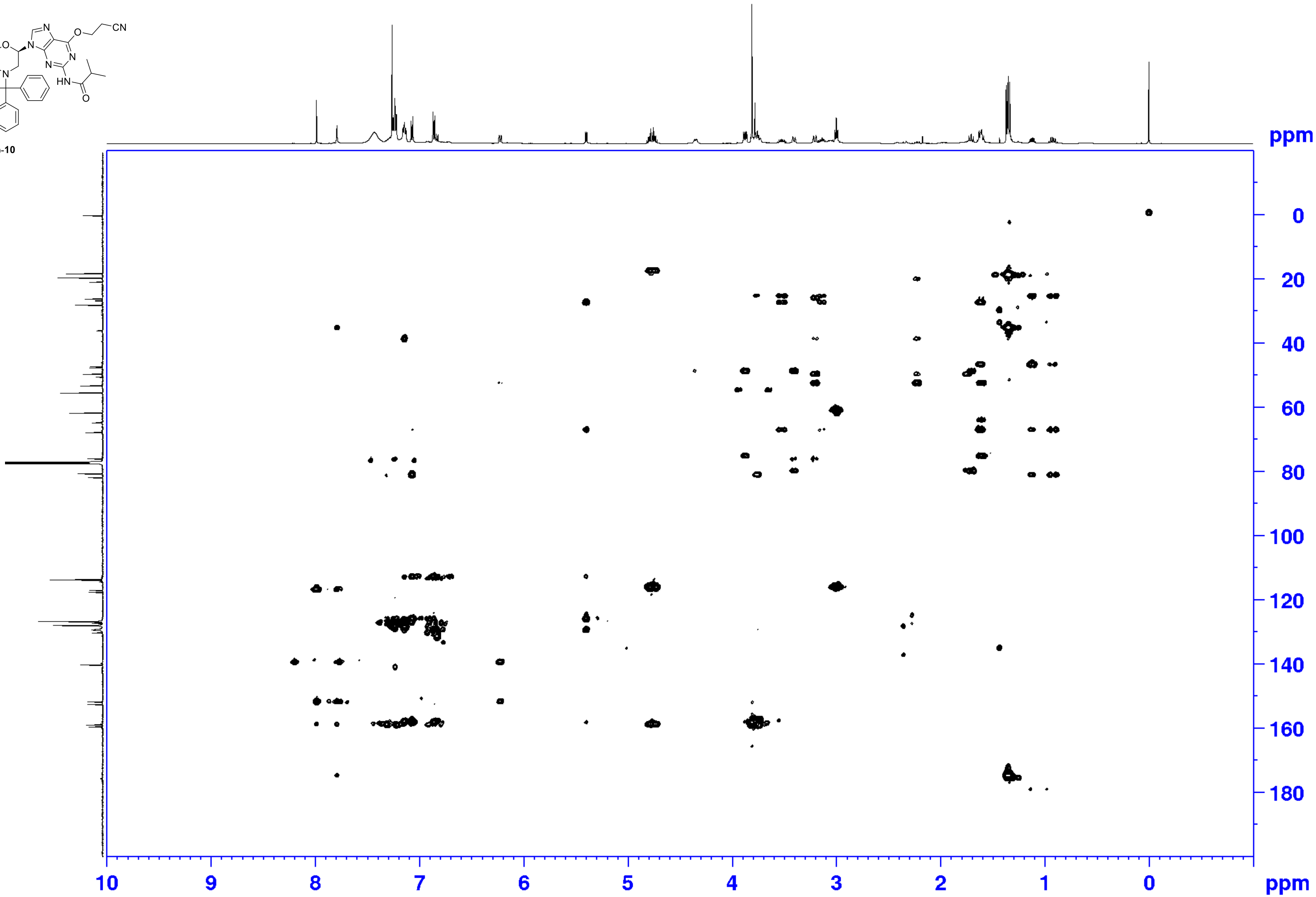

^{1}H NMR (500 MHz, $CDCl_3$) of (*S*p)-**10**

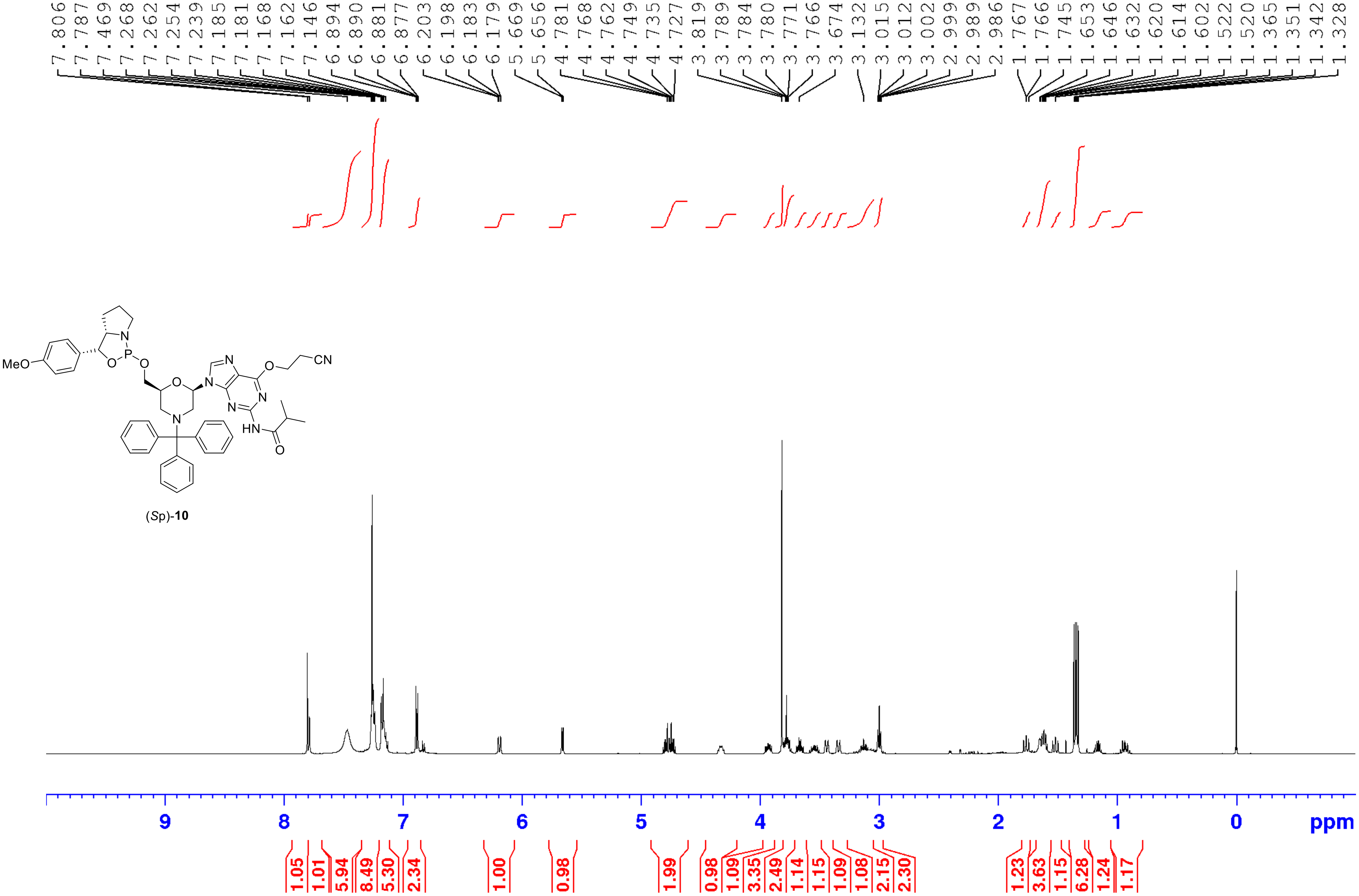

^{13}C {^{1}H} NMR (126 MHz, $CDCl_3$) of (*S*p)-**10**

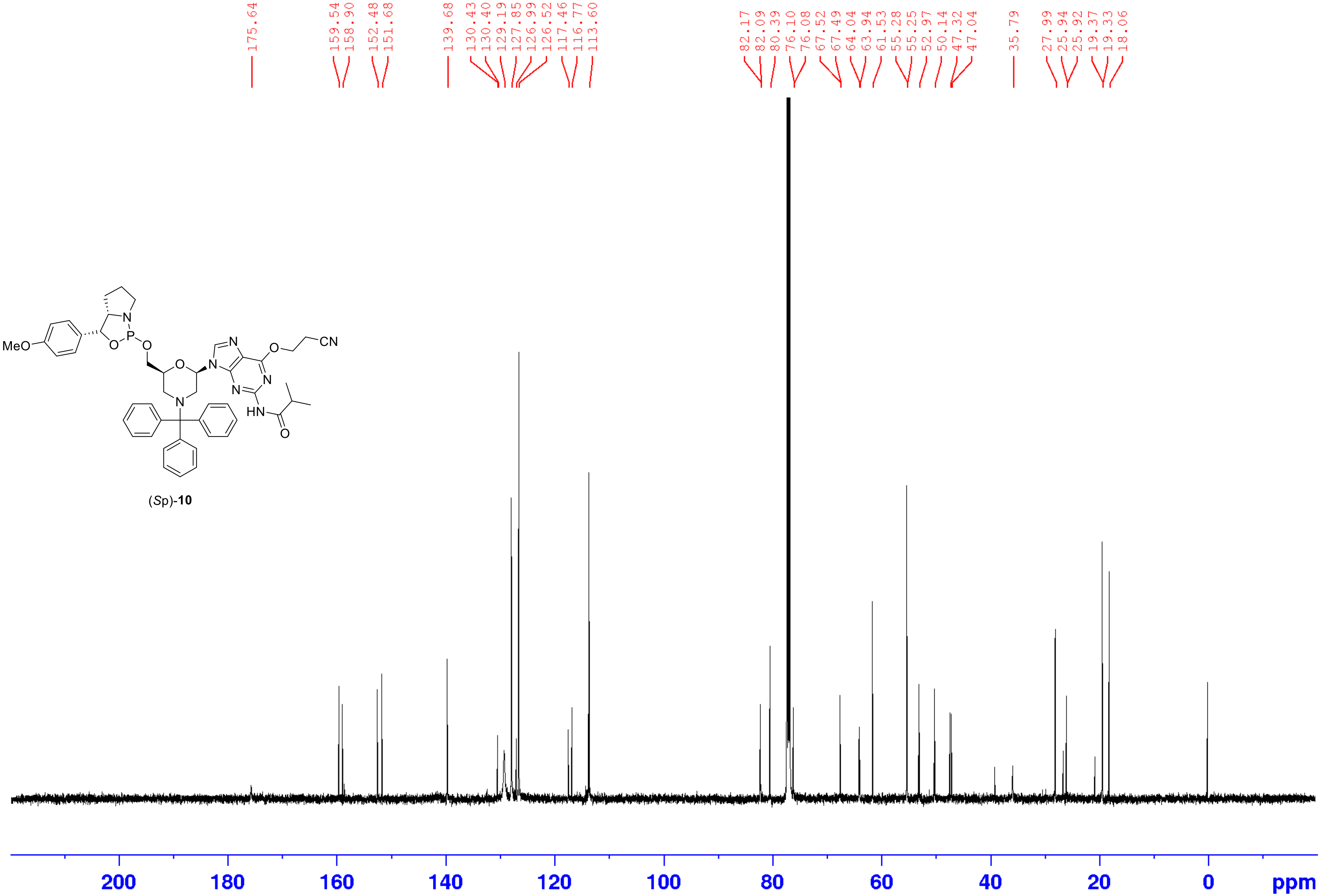

$^{31}P\ \{^{1}H\}$ NMR (202 MHz, $CDCl_3$) of (*S*p)-**10**

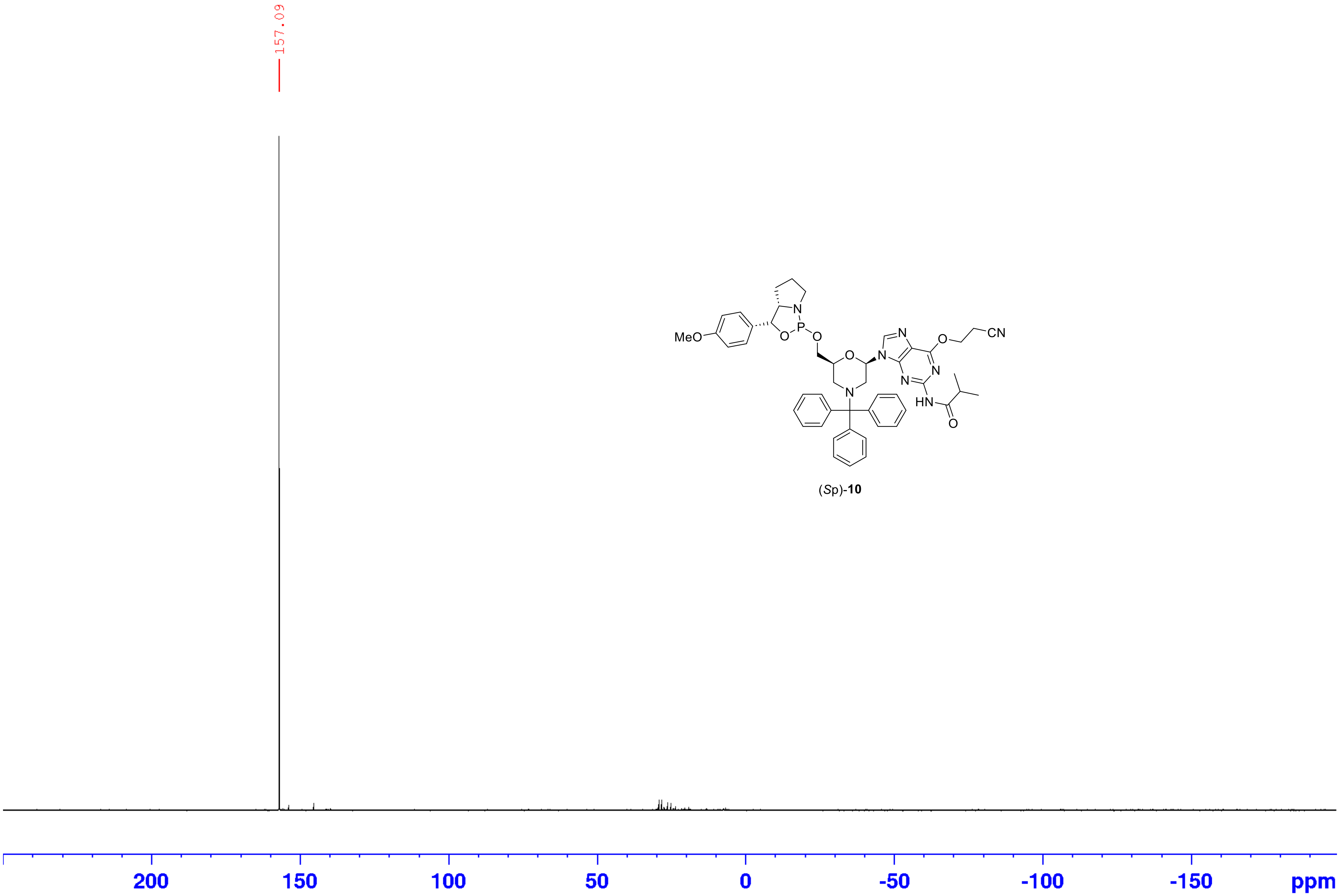

COSY ($CDCl_3$) of (*S*p)-**10**

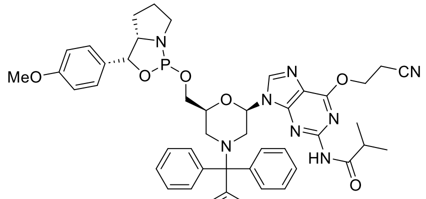

(*S*p)-**10**

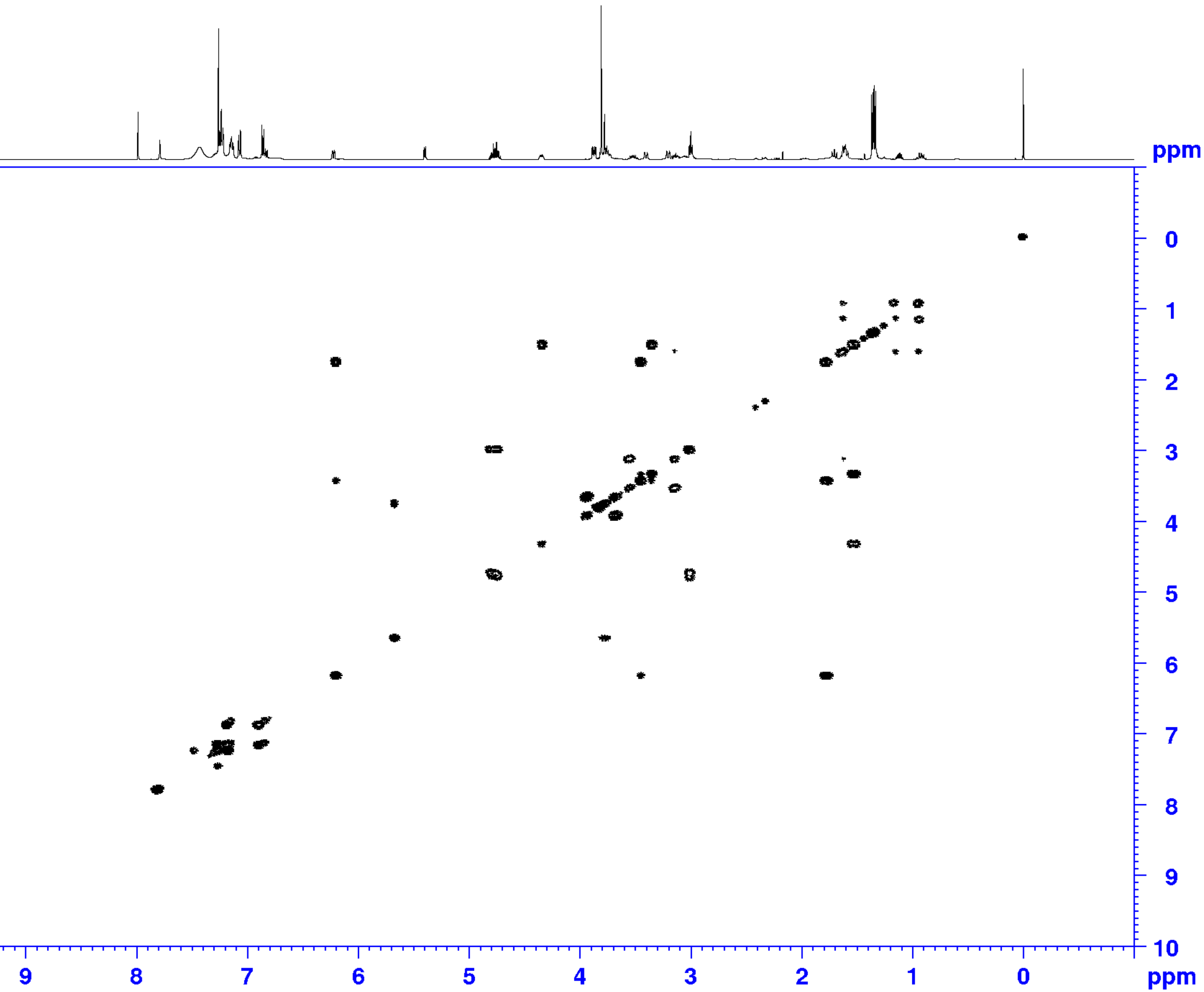

HSQC (CDCl$_3$) of (*S*p)-**10**

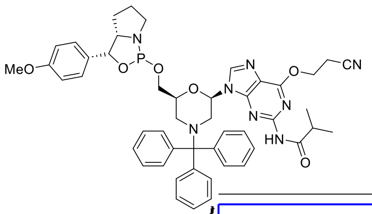

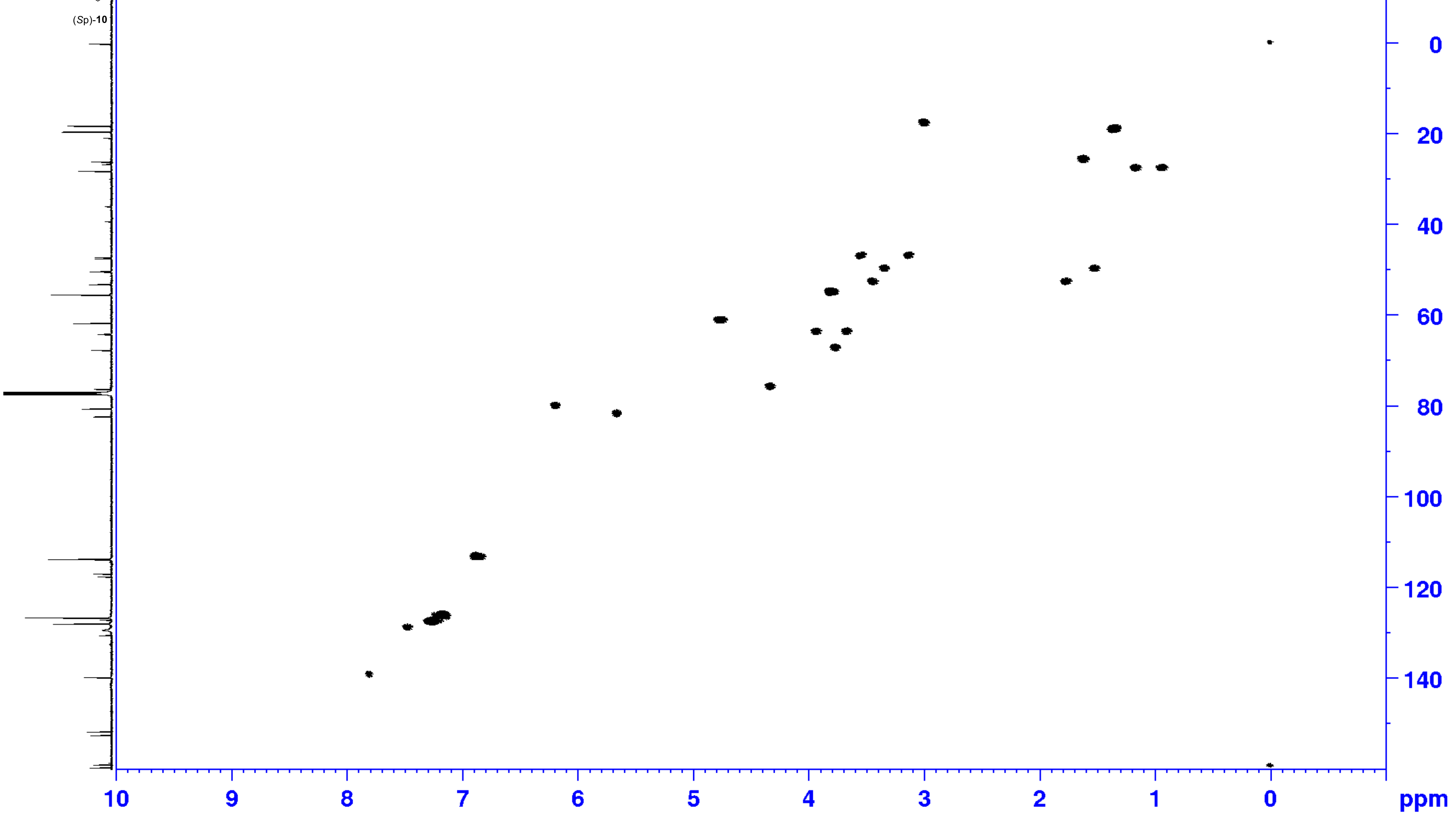

HMBC ($CDCl_3$) of (*S*p)-**10**

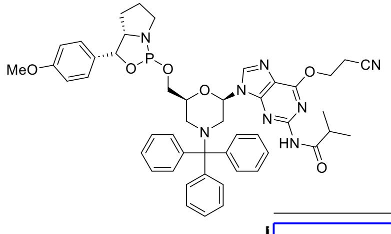

(*S*p)-**10**

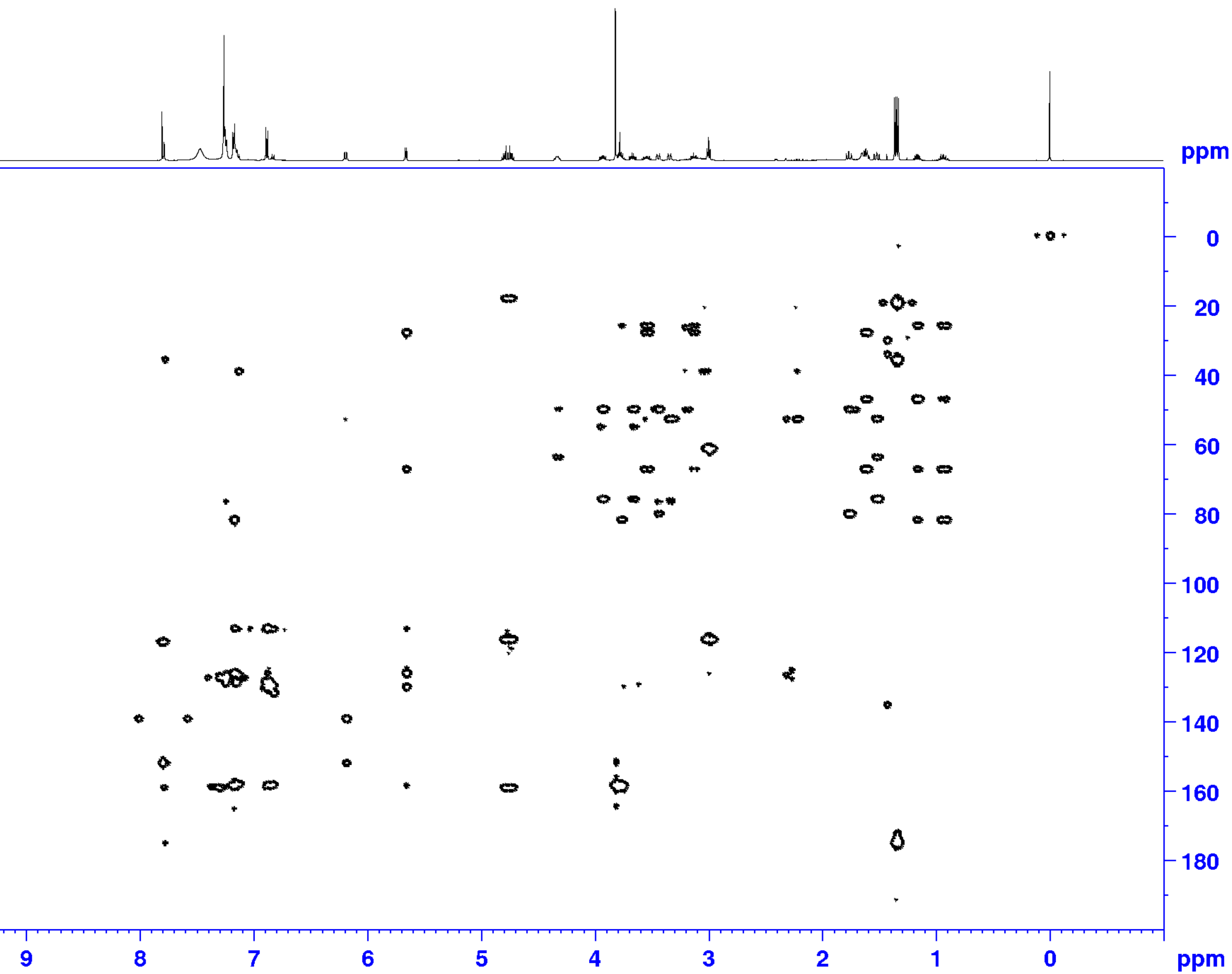

^{1}H NMR (500 MHz, $CDCl_3$) of (*R*p)-**13**

(*R*p)-**13**

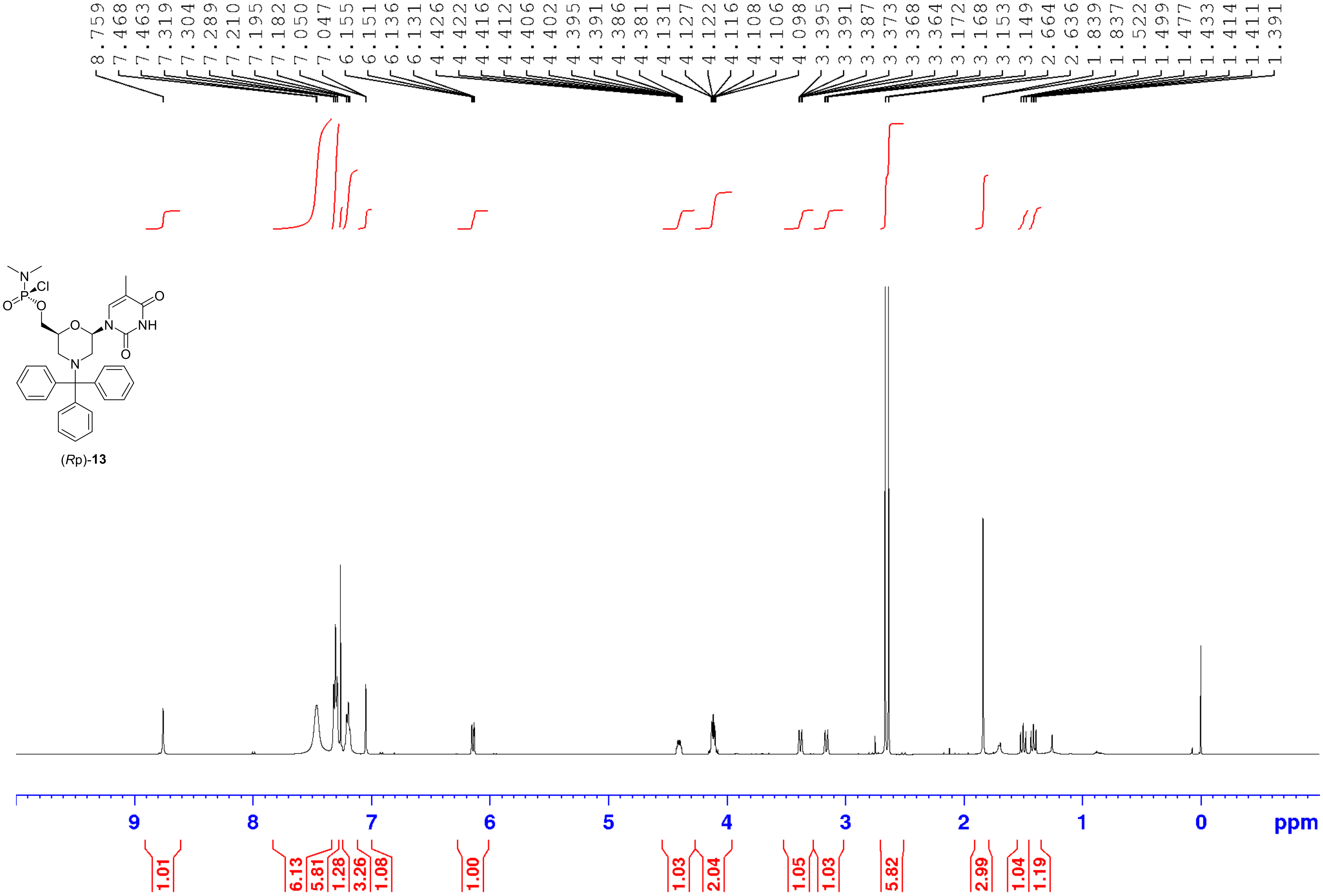

$^{13}C\ \{^{1}H\}$ NMR (126 MHz, $CDCl_3$) of (*R*p)-**13**

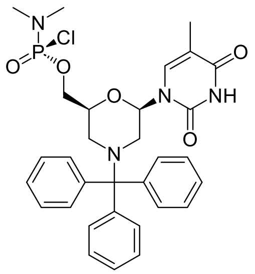

(*R*p)-**13**

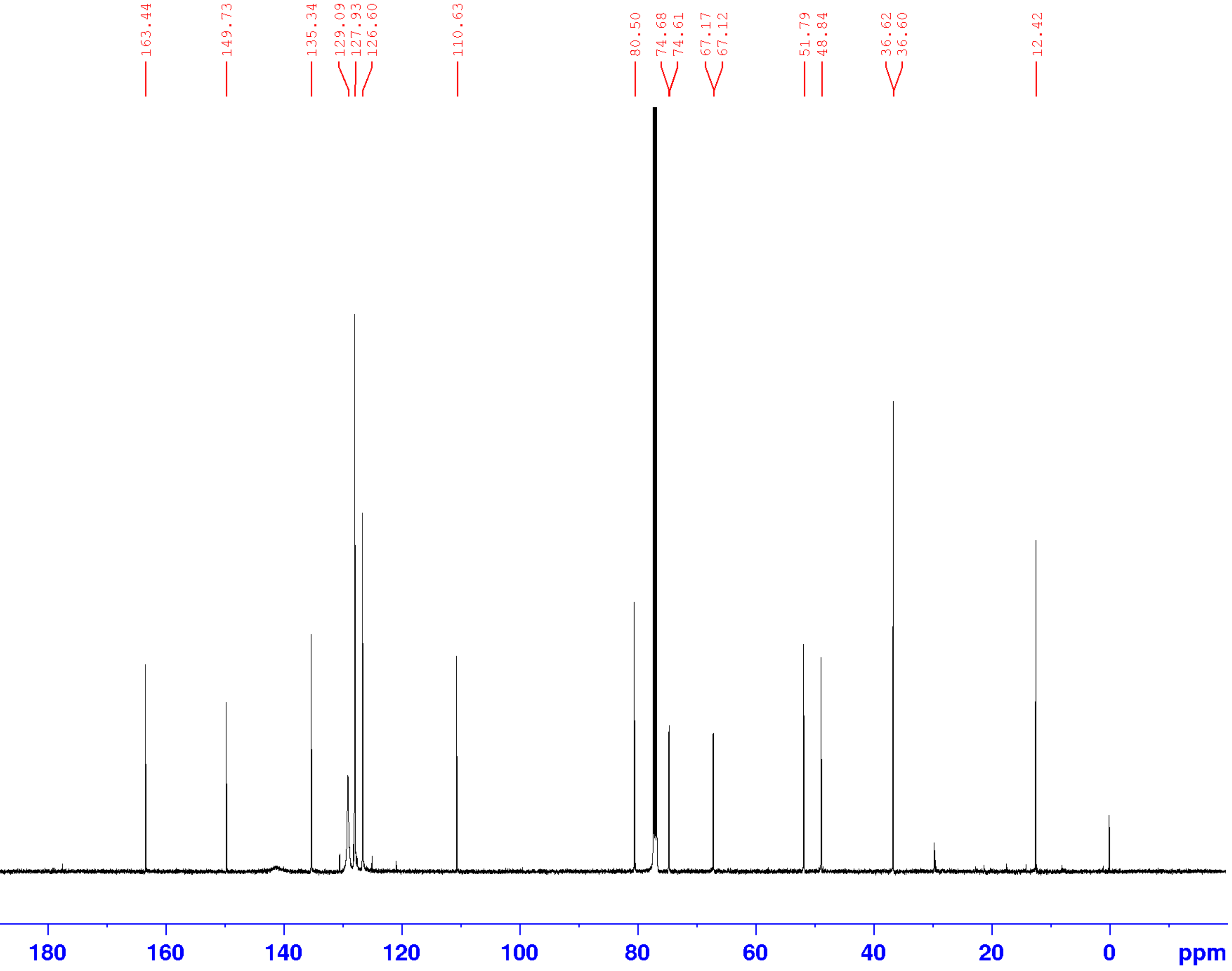

$^{31}P\ \{^{1}H\}$ NMR (202 MHz, CDCl$_3$) of (*R*p)-**13**

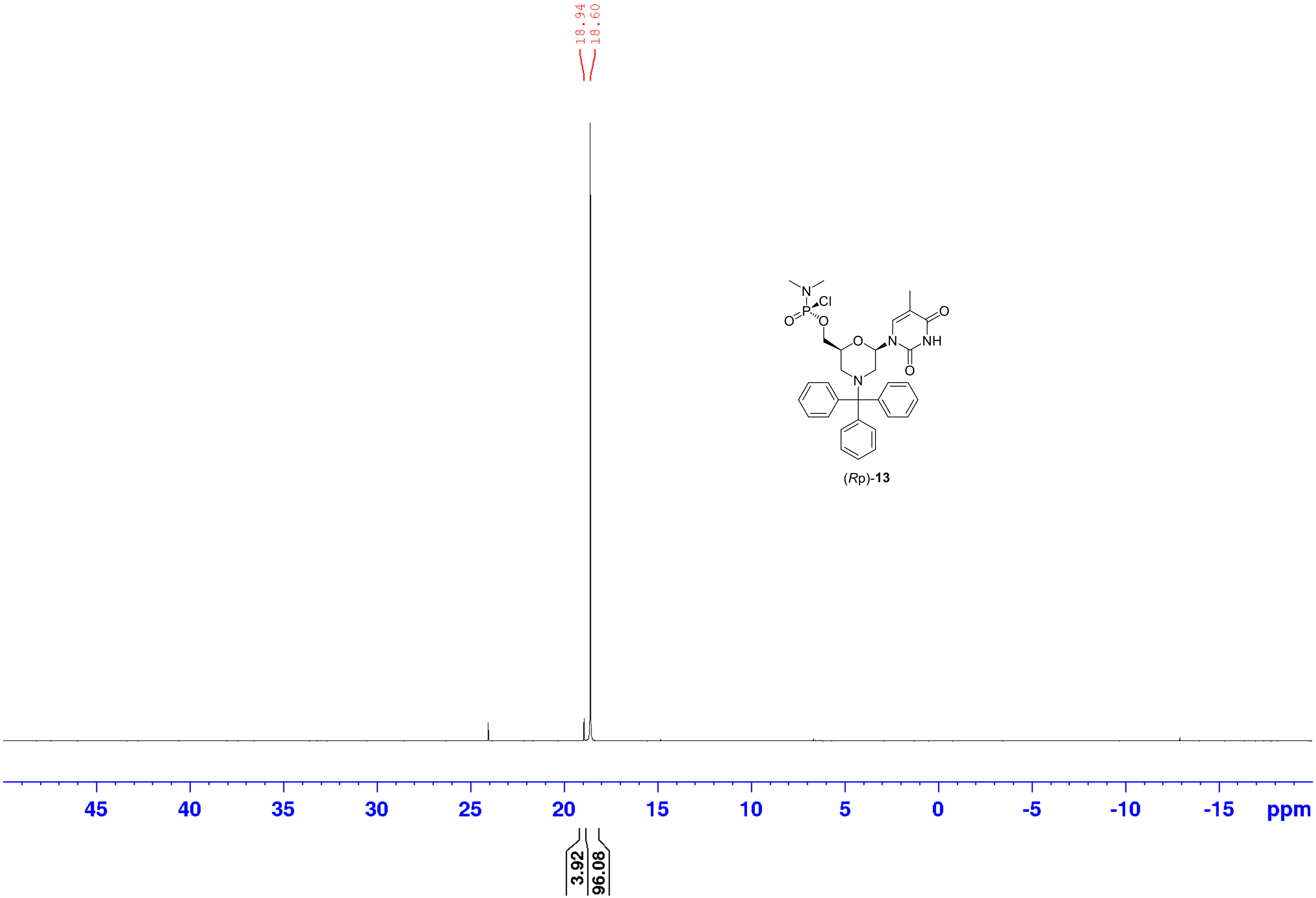

COSY ($CDCl_3$) of (*R*p)-**13**

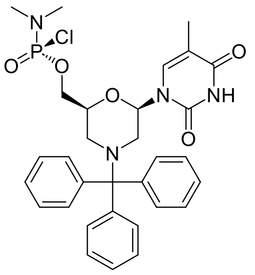

(*R*p)-**13**

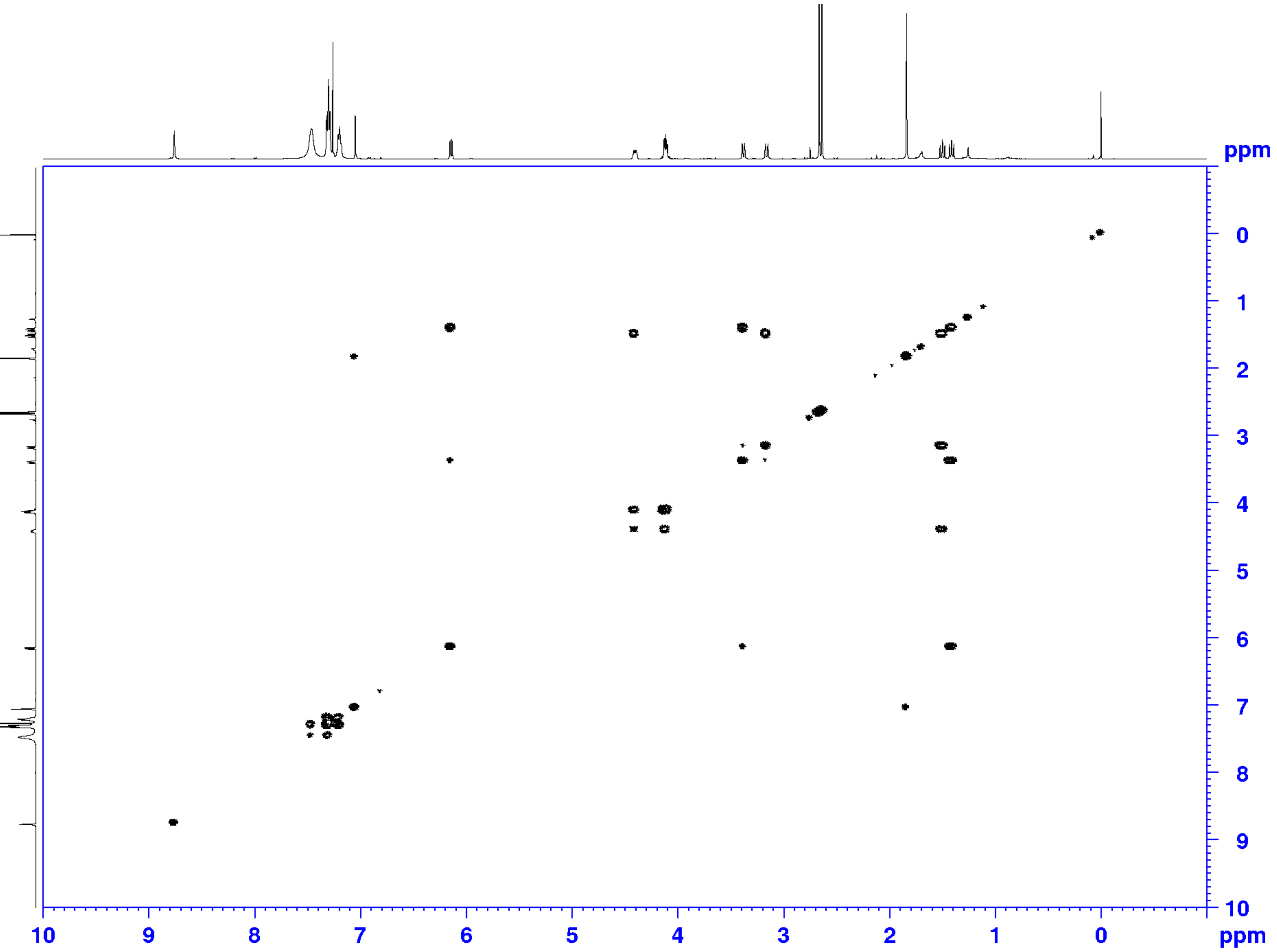

HSQC ($CDCl_3$) of (*R*p)-**13**

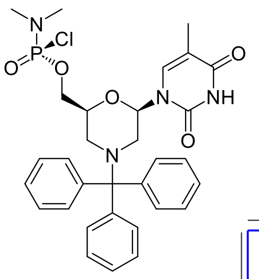

(*R*p)-**13**

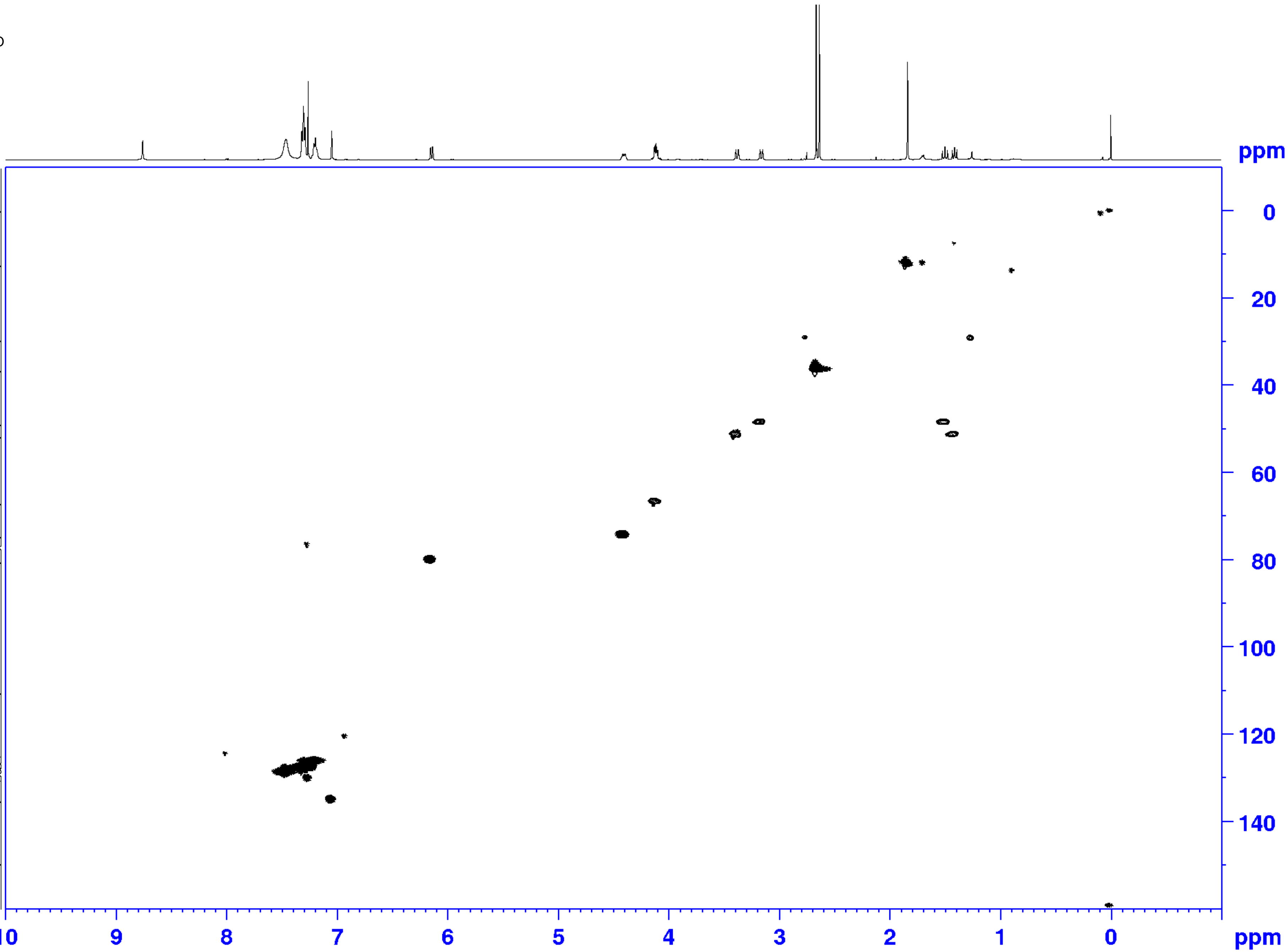

HMBC ($CDCl_3$) of (*R*p)-**13**

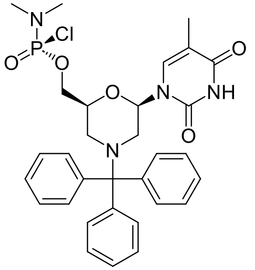

(*R*p)-**13**

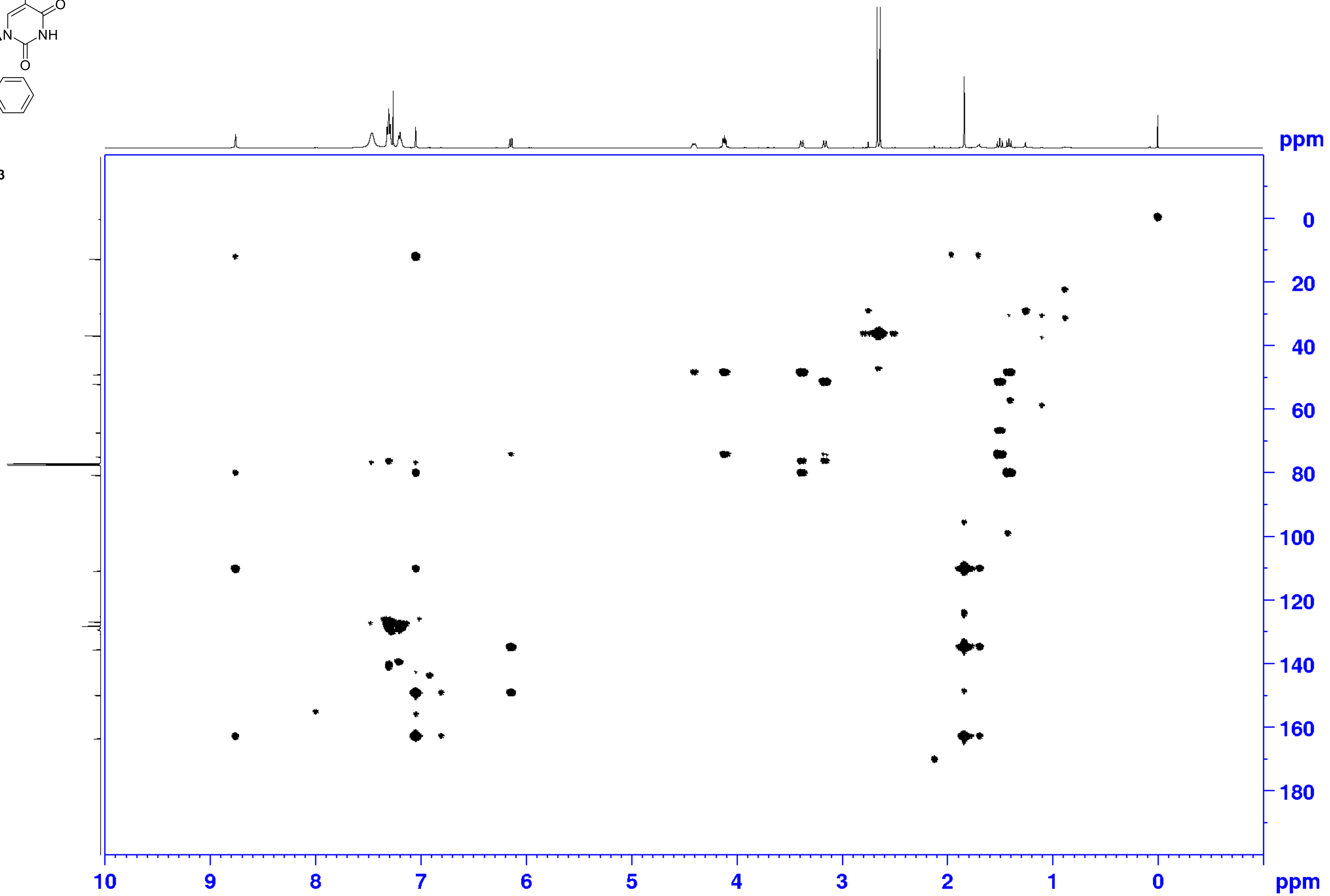

^{1}H NMR (500 MHz, $CDCl_3$) of (*S*p)-**13**

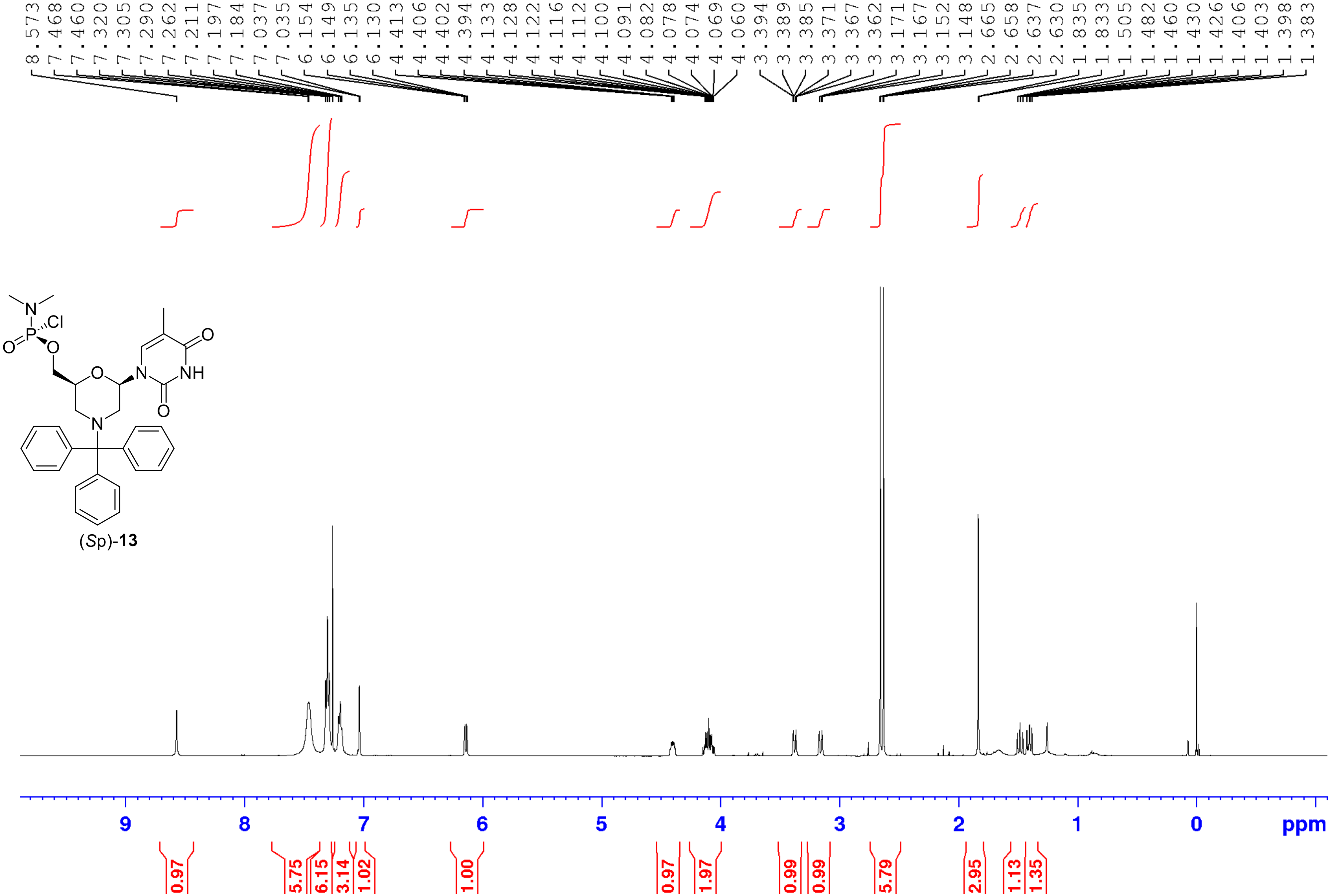

^{13}C {^{1}H} NMR (126 MHz, $CDCl_3$) of (*S*p)-**13**

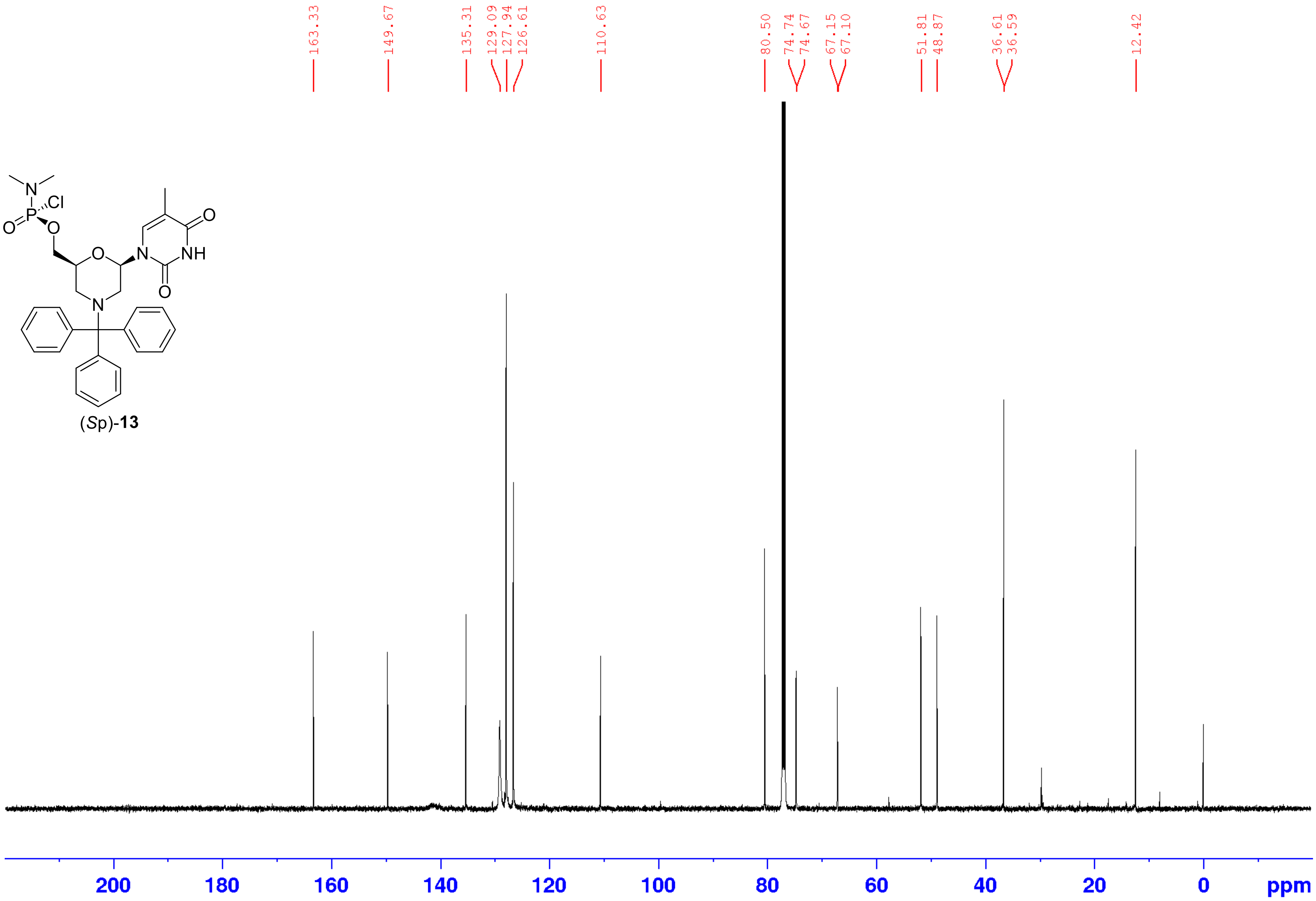

^{31}P {^{1}H} NMR (202 MHz, $CDCl_3$) of (*S*p)-**13**

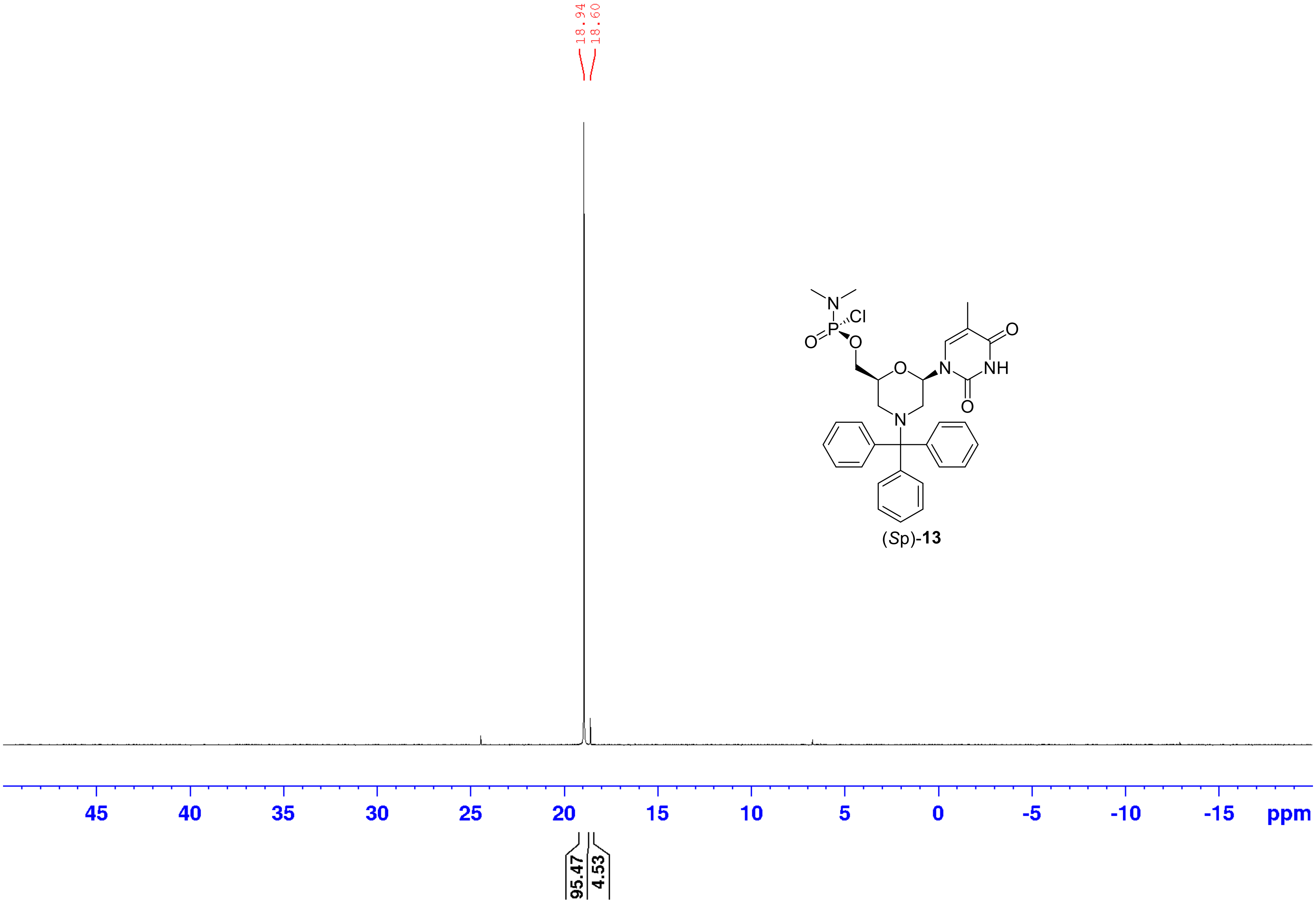

COSY ($CDCl_3$) of (*S*p)-**13**

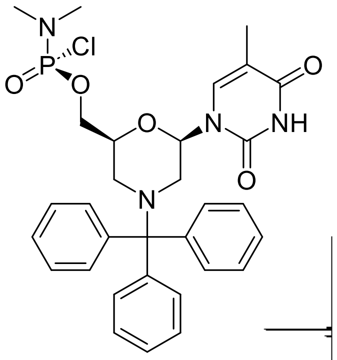

(*S*p)-**13**

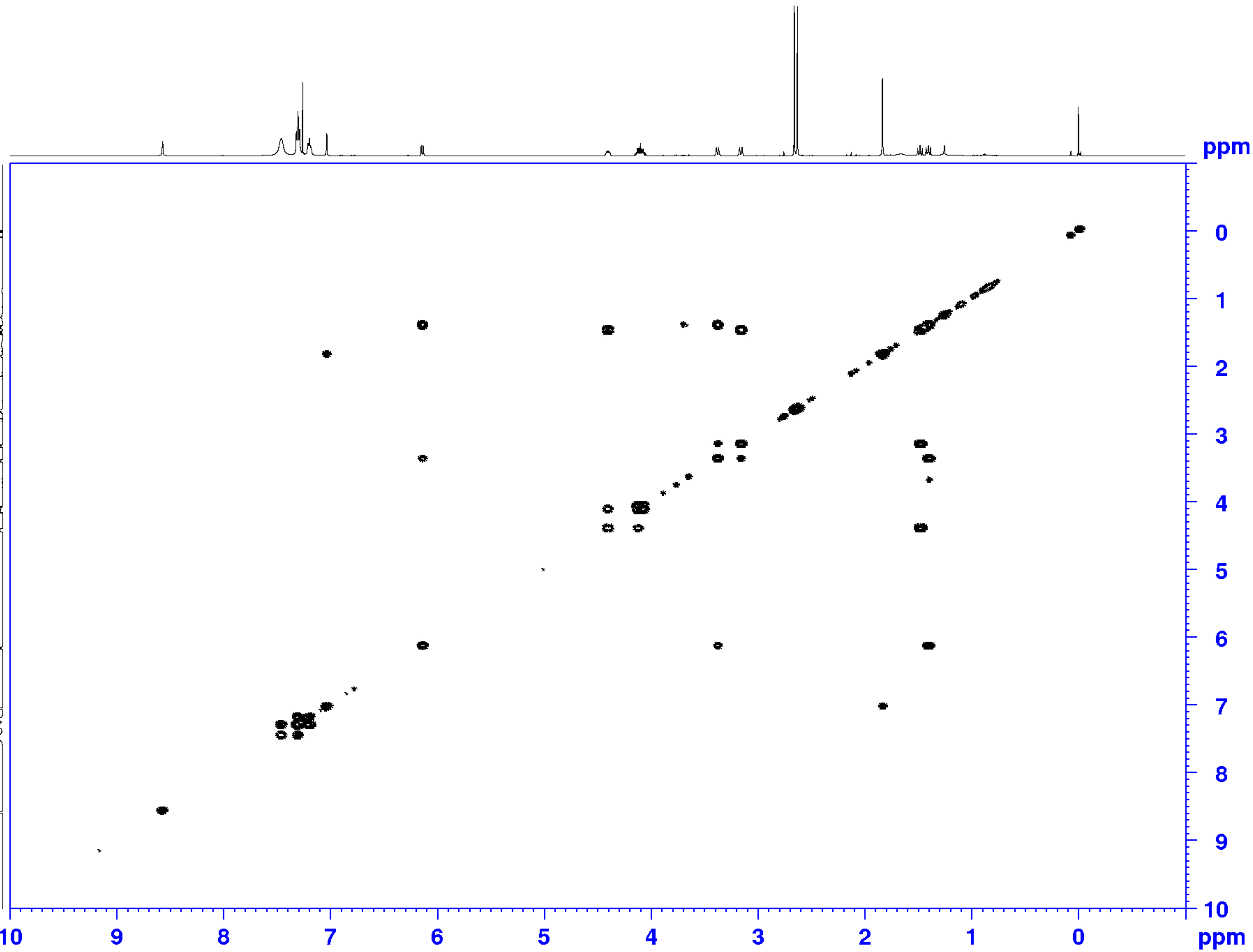

HSQC ($CDCl_3$) of (*S*p)-**13**

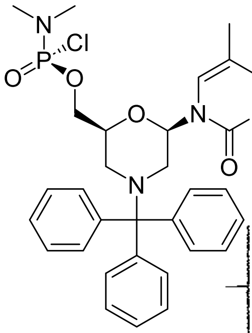

(*S*p)-**13**

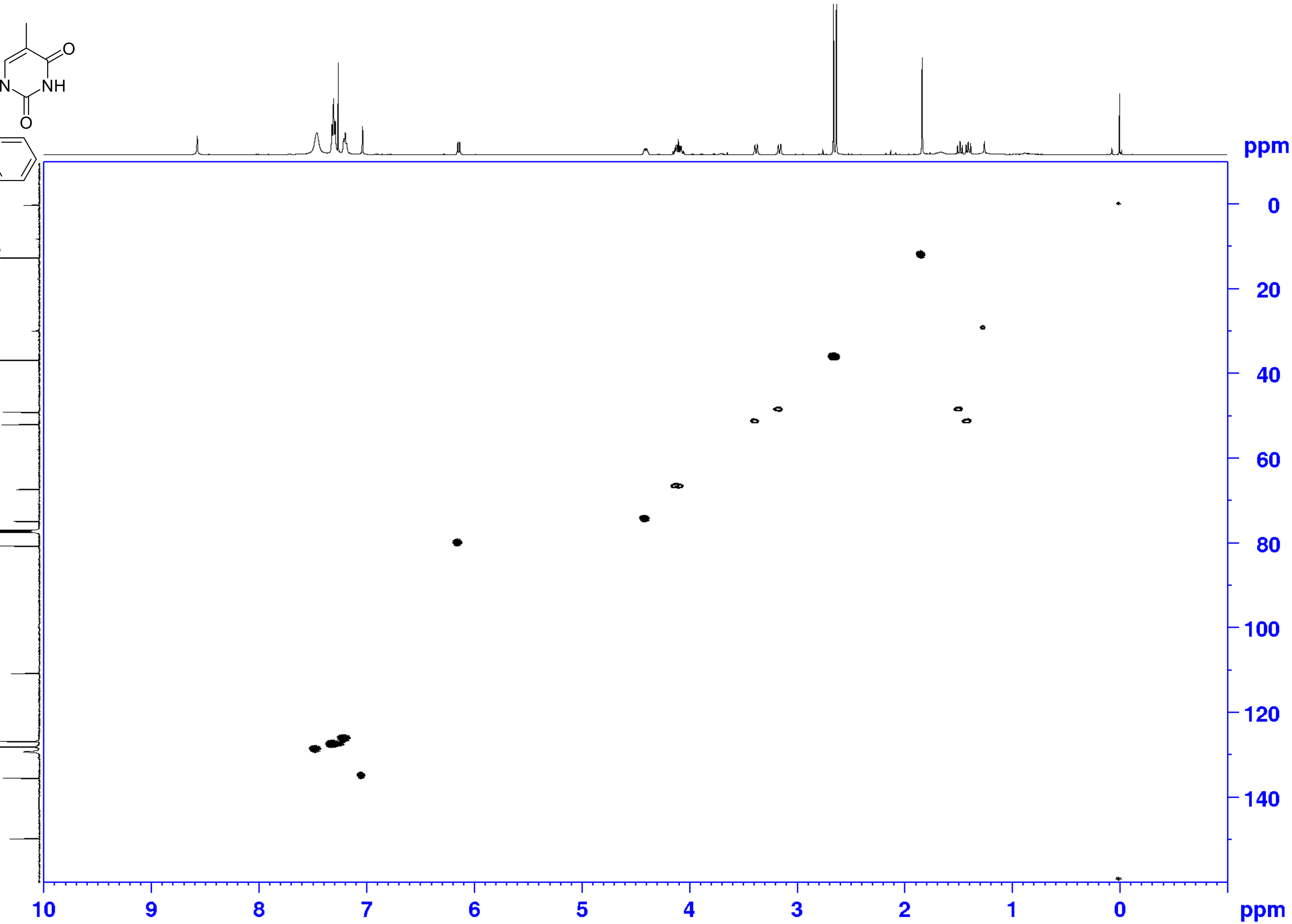

HMBC ($CDCl_3$) of (*S*p)-**13**

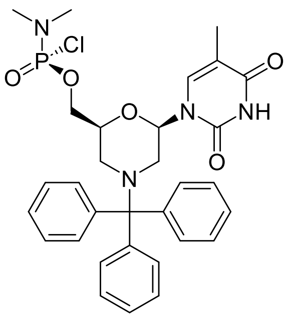

(*S*p)-**13**

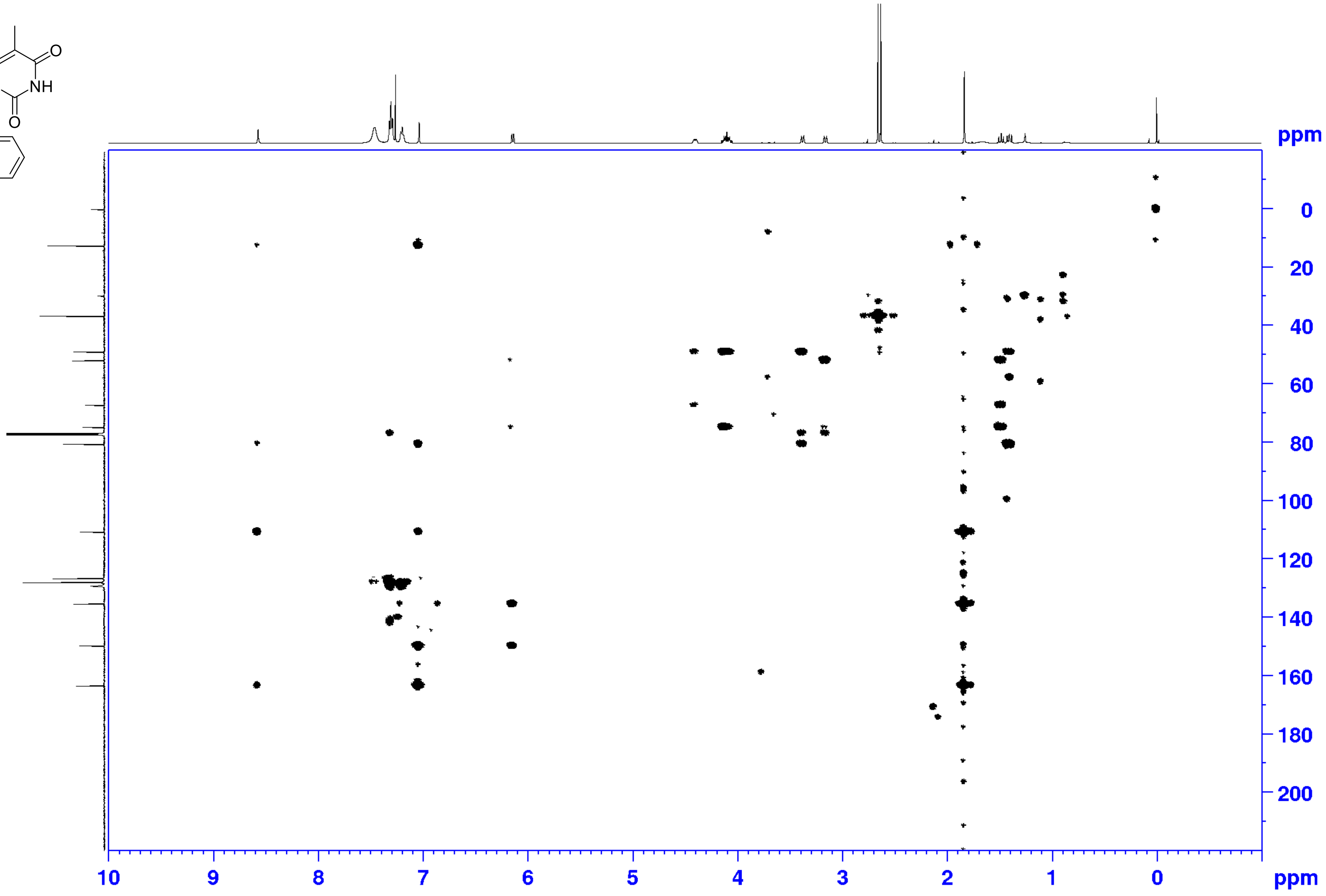

^{1}H NMR (500 MHz, CDCl$_3$) of (*R*p)-**16**

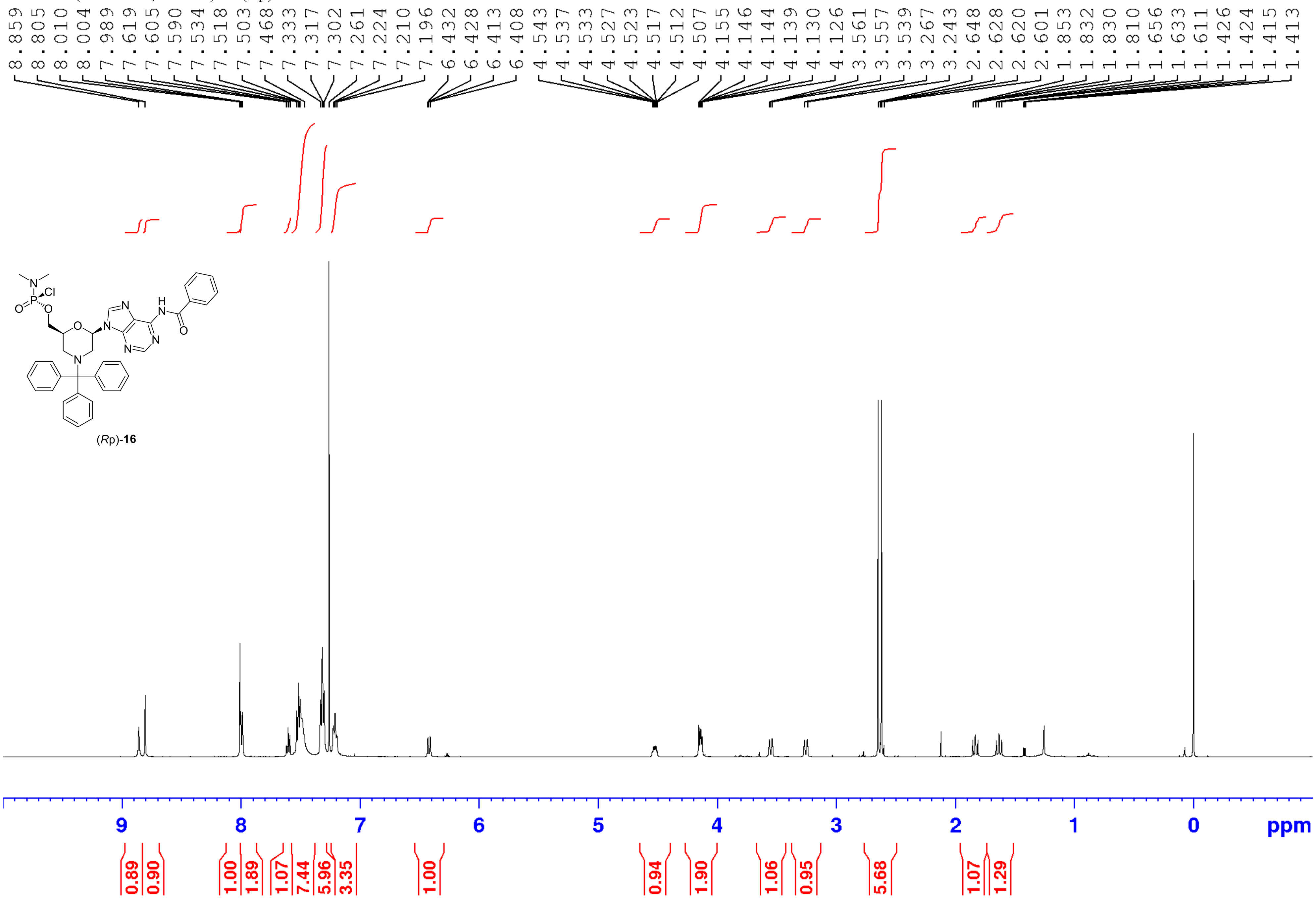

^{13}C {^{1}H} NMR (126 MHz, $CDCl_3$) of (*R*p)-**16**

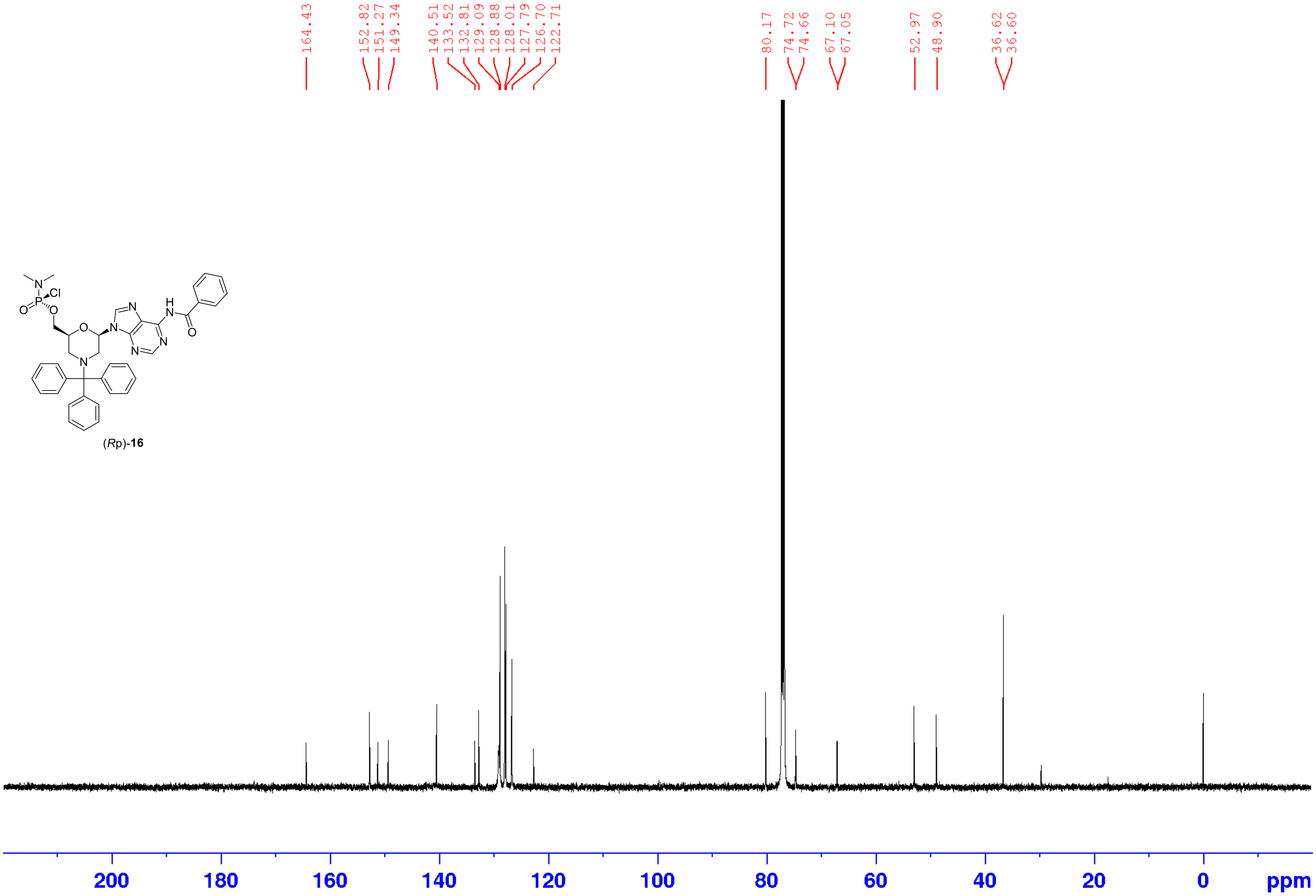

(*R*p)-**16**

^{31}P {^{1}H} NMR (202 MHz, $CDCl_3$) of (*R*p)-**16**

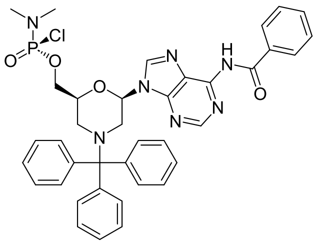

(*R*p)-**16**

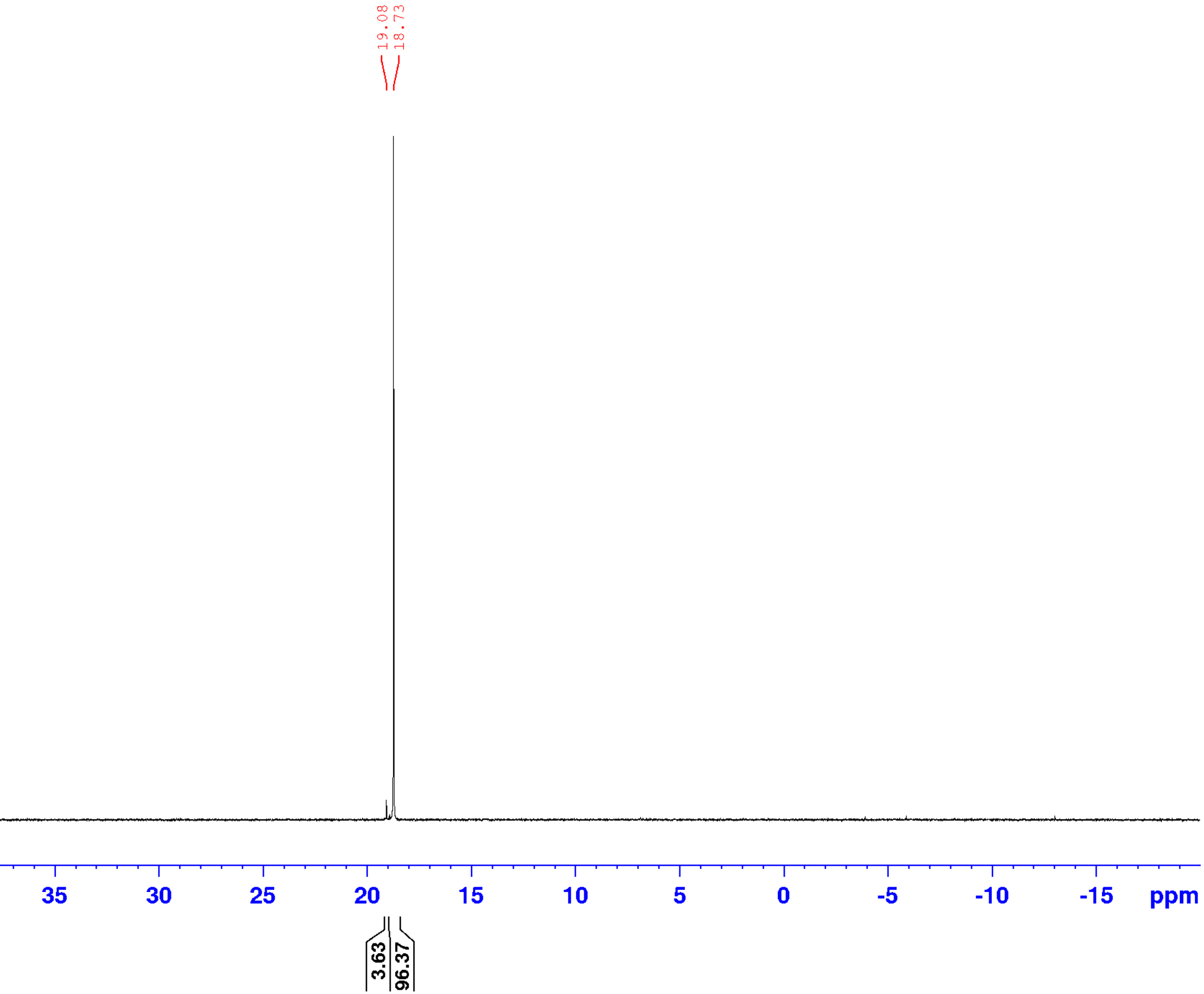

COSY ($CDCl_3$) of (*R*p)-**16**

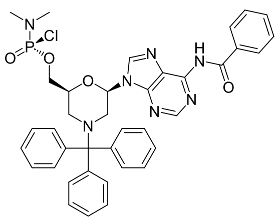

(*R*p)-**16**

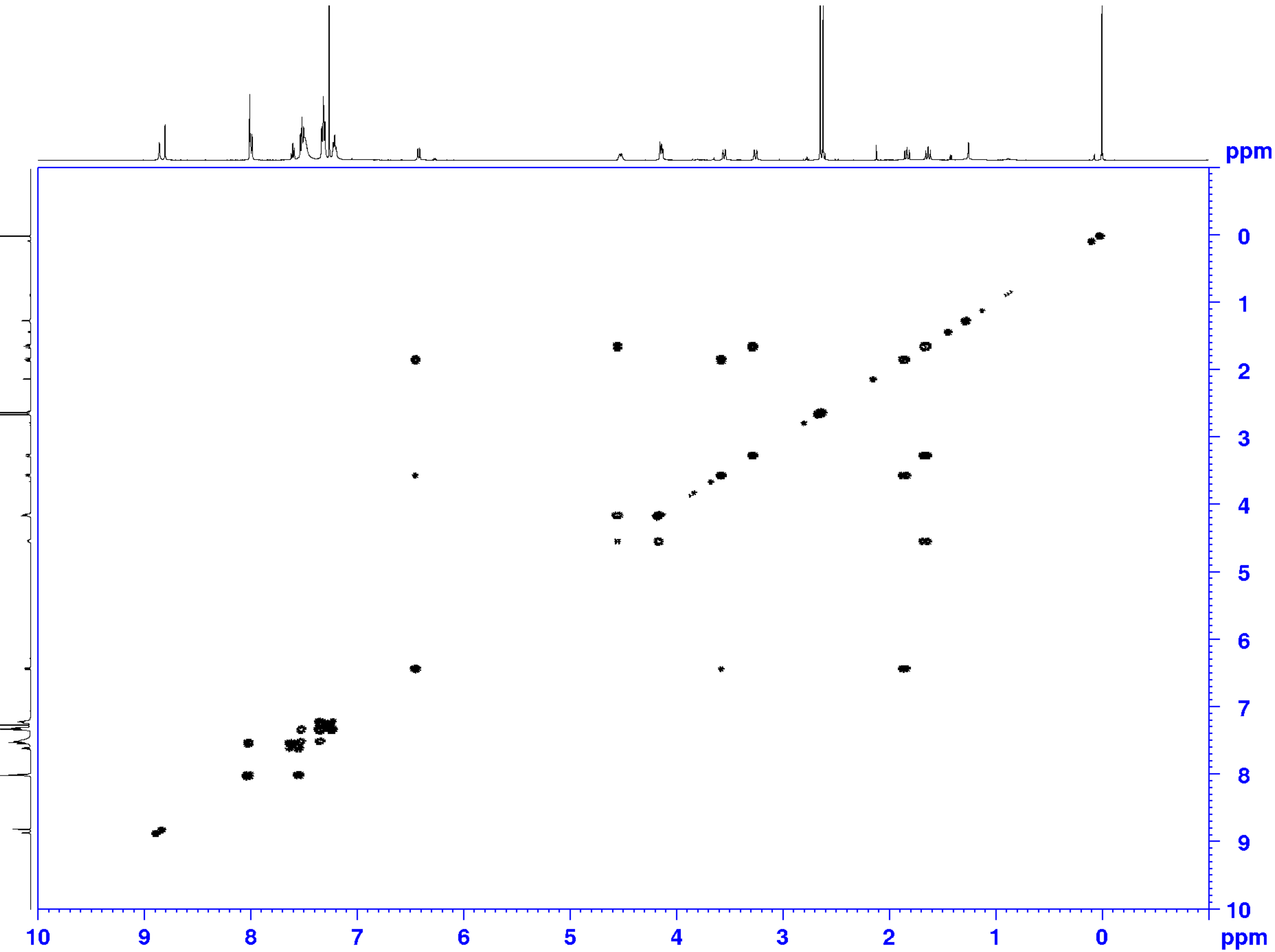

HSQC ($CDCl_3$) of (*R*p)-**16**

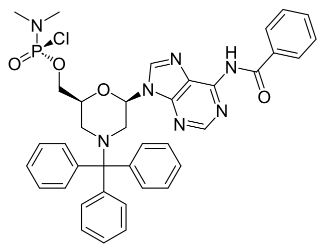

(*R*p)-**16**

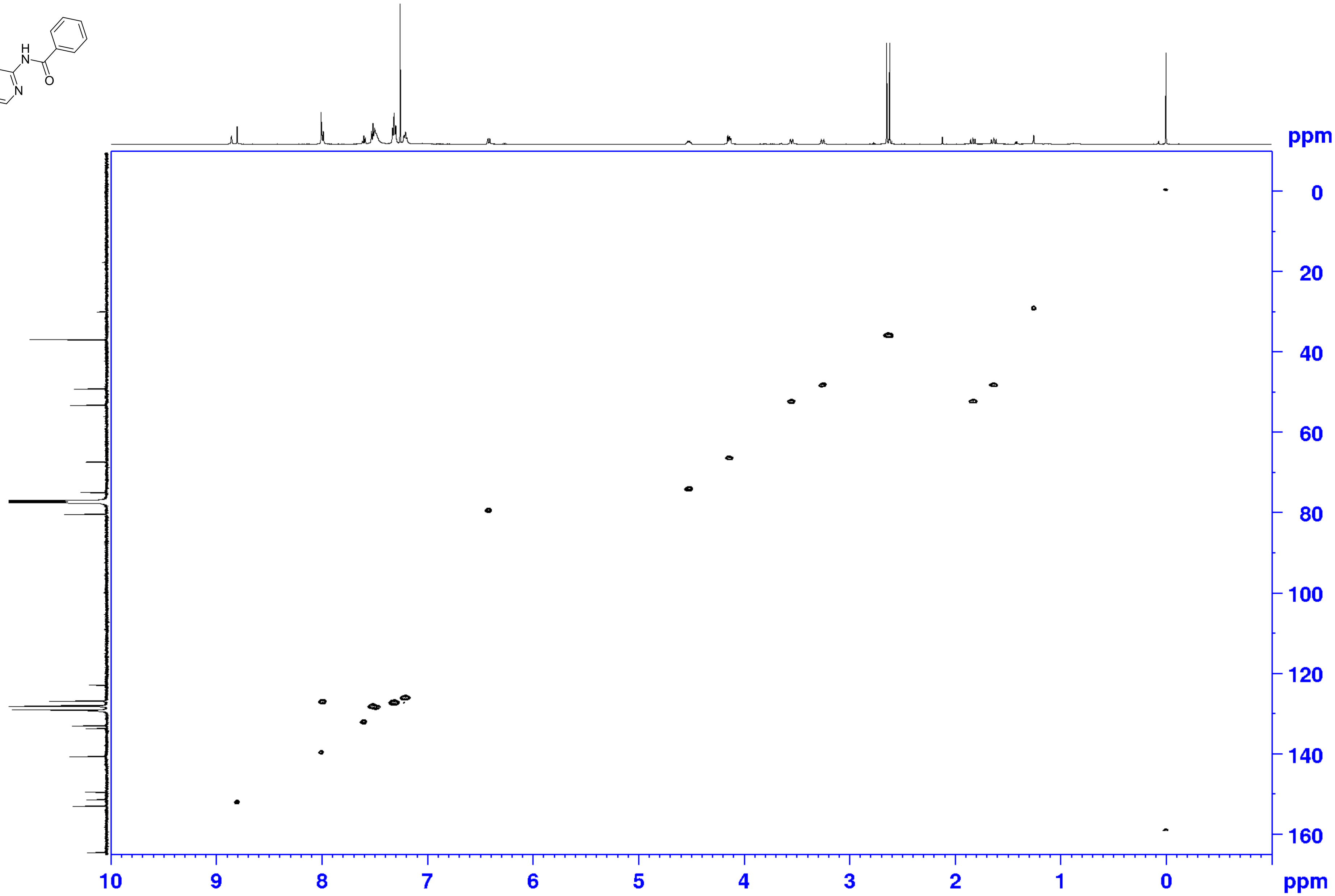

HMBC ($CDCl_3$) of (*R*p)-**16**

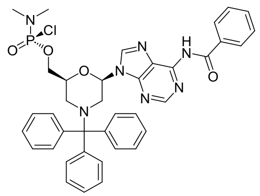

(*R*p)-**16**

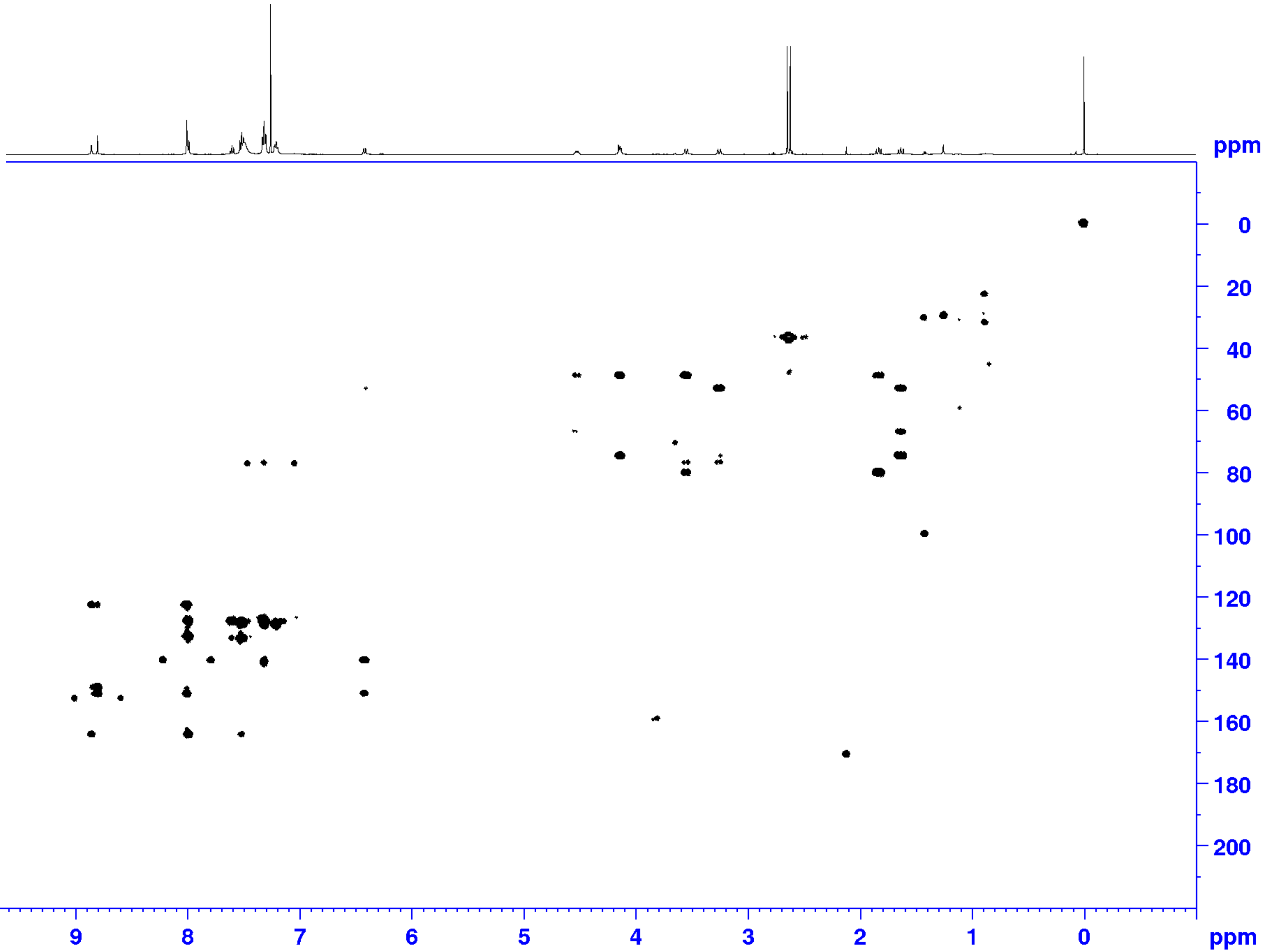

^{1}H NMR (500 MHz, $CDCl_3$) of (*S*p)-**16**

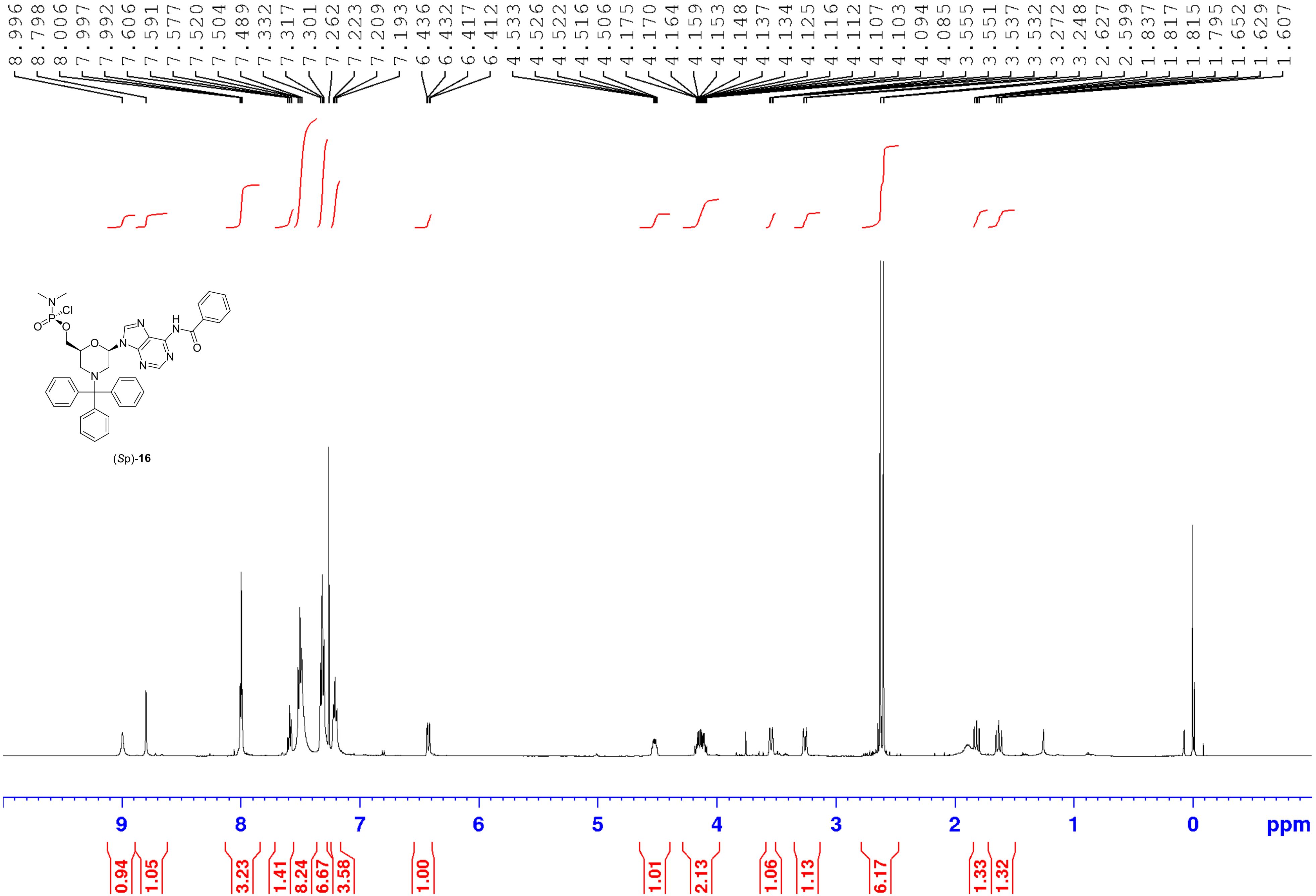

^{13}C {^{1}H} NMR (126 MHz, $CDCl_3$) of (*S*p)-**16**

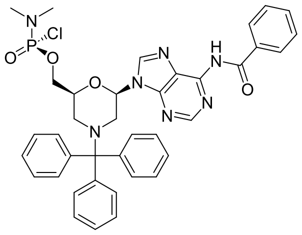

(*S*p)-**16**

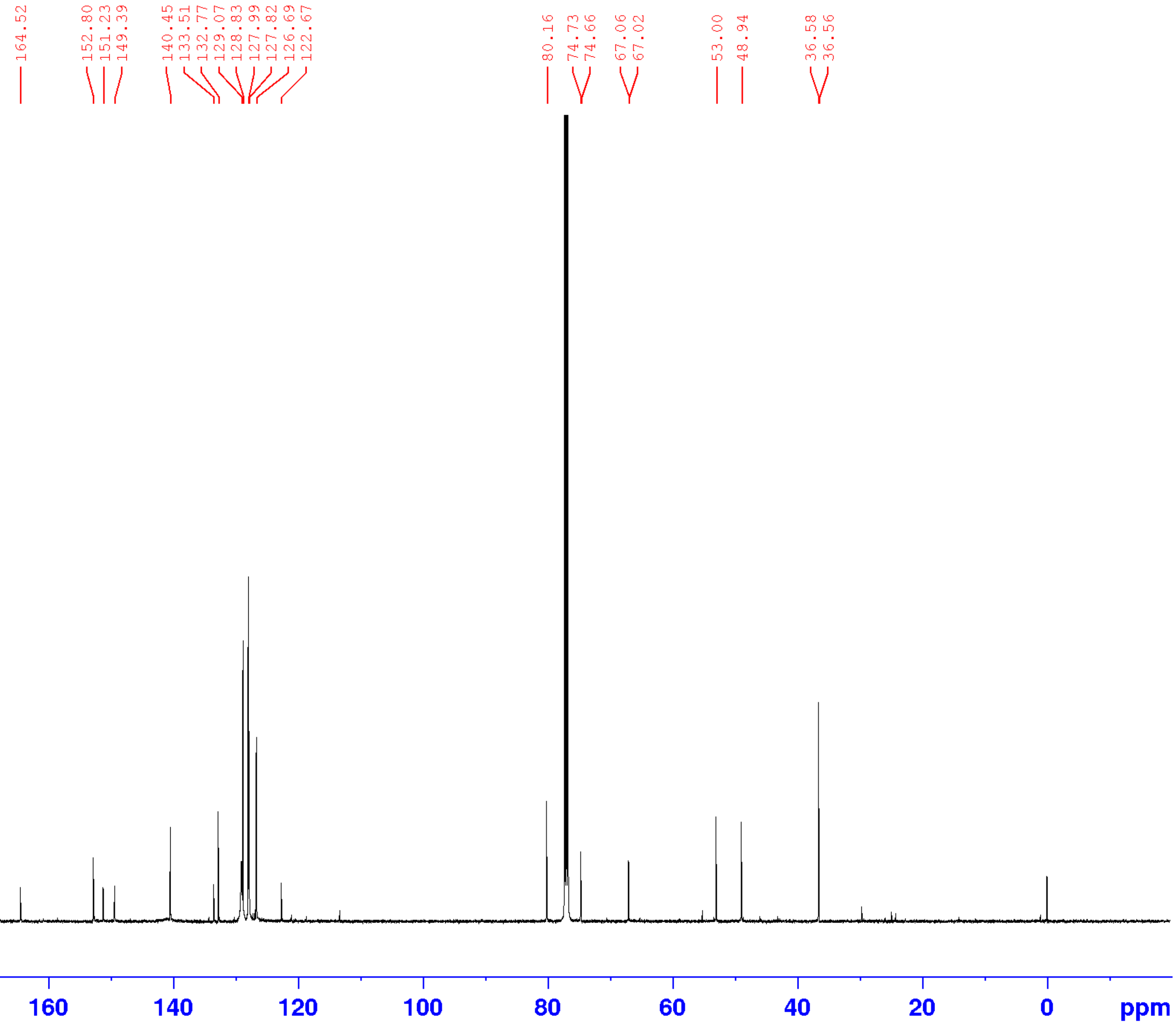

^{31}P {^{1}H} NMR (202 MHz, $CDCl_3$) of (*S*p)-**16**

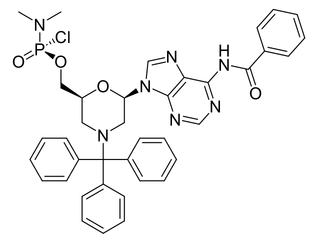

(*S*p)-**16**

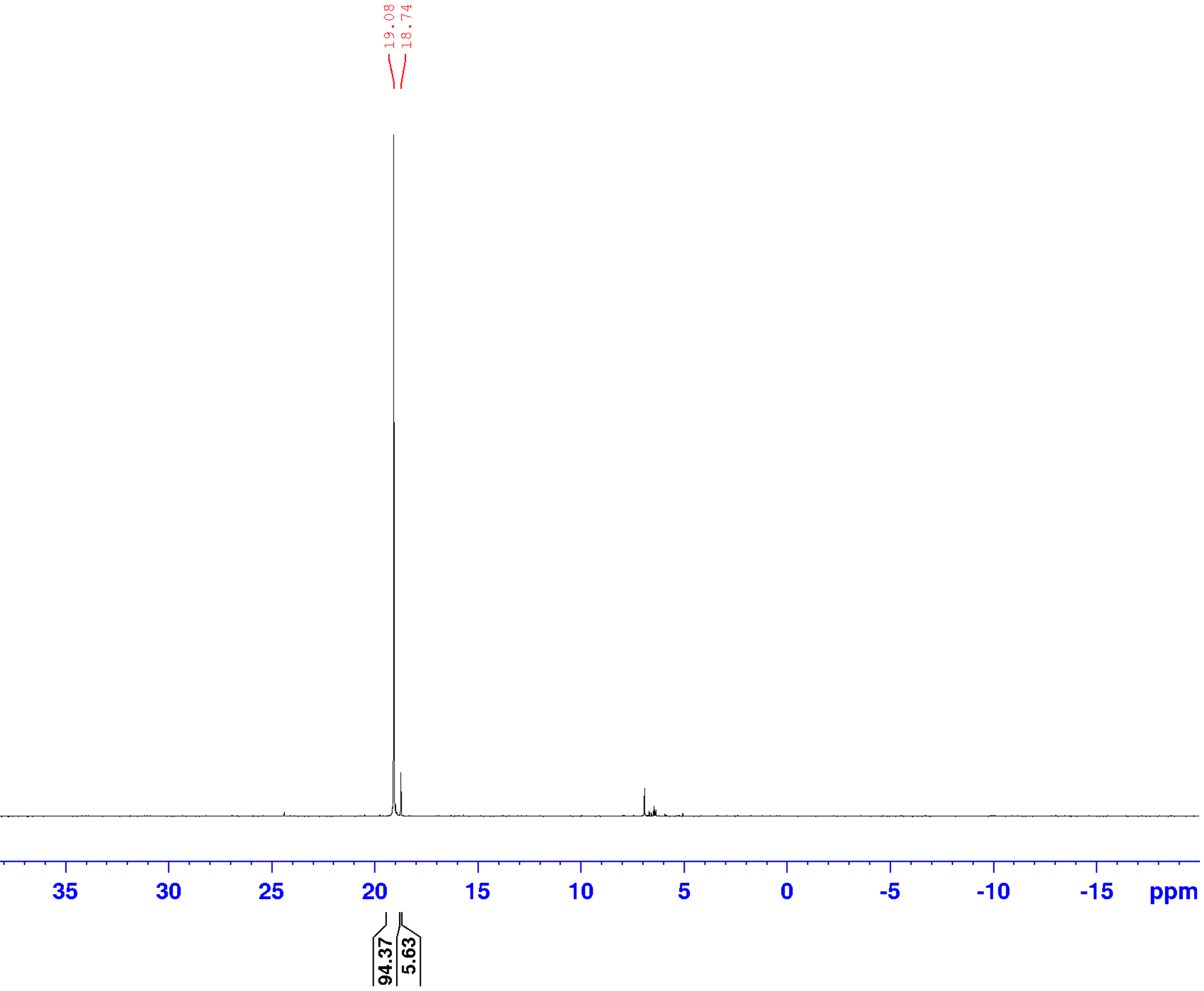

COSY ($CDCl_3$) of (*S*p)-**16**

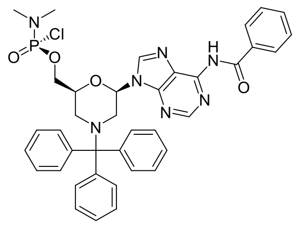

(*S*p)-**16**

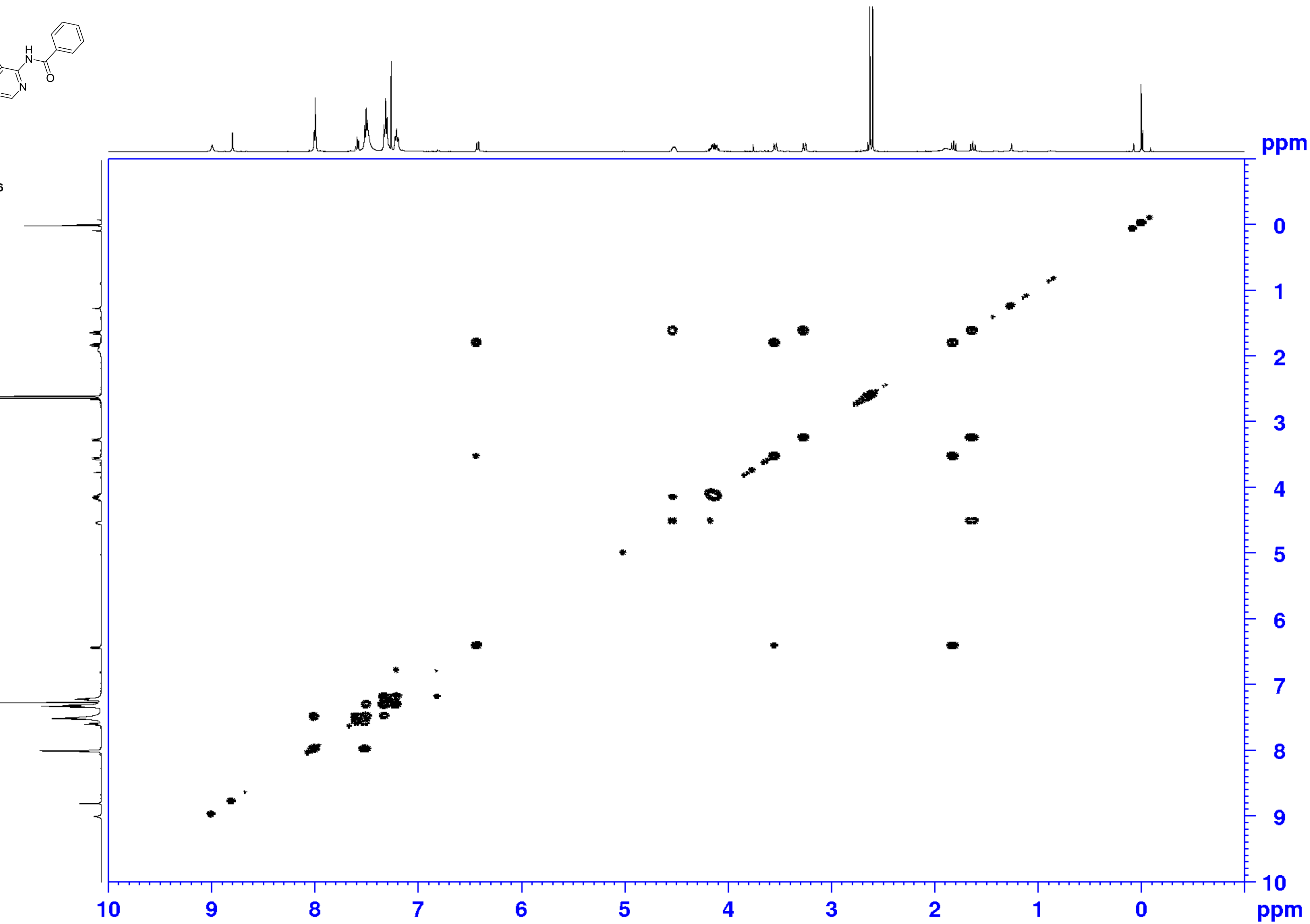

HSQC (CDCl₃) of (*S*p)-**16**

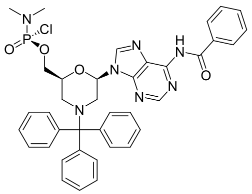

(*S*p)-**16**

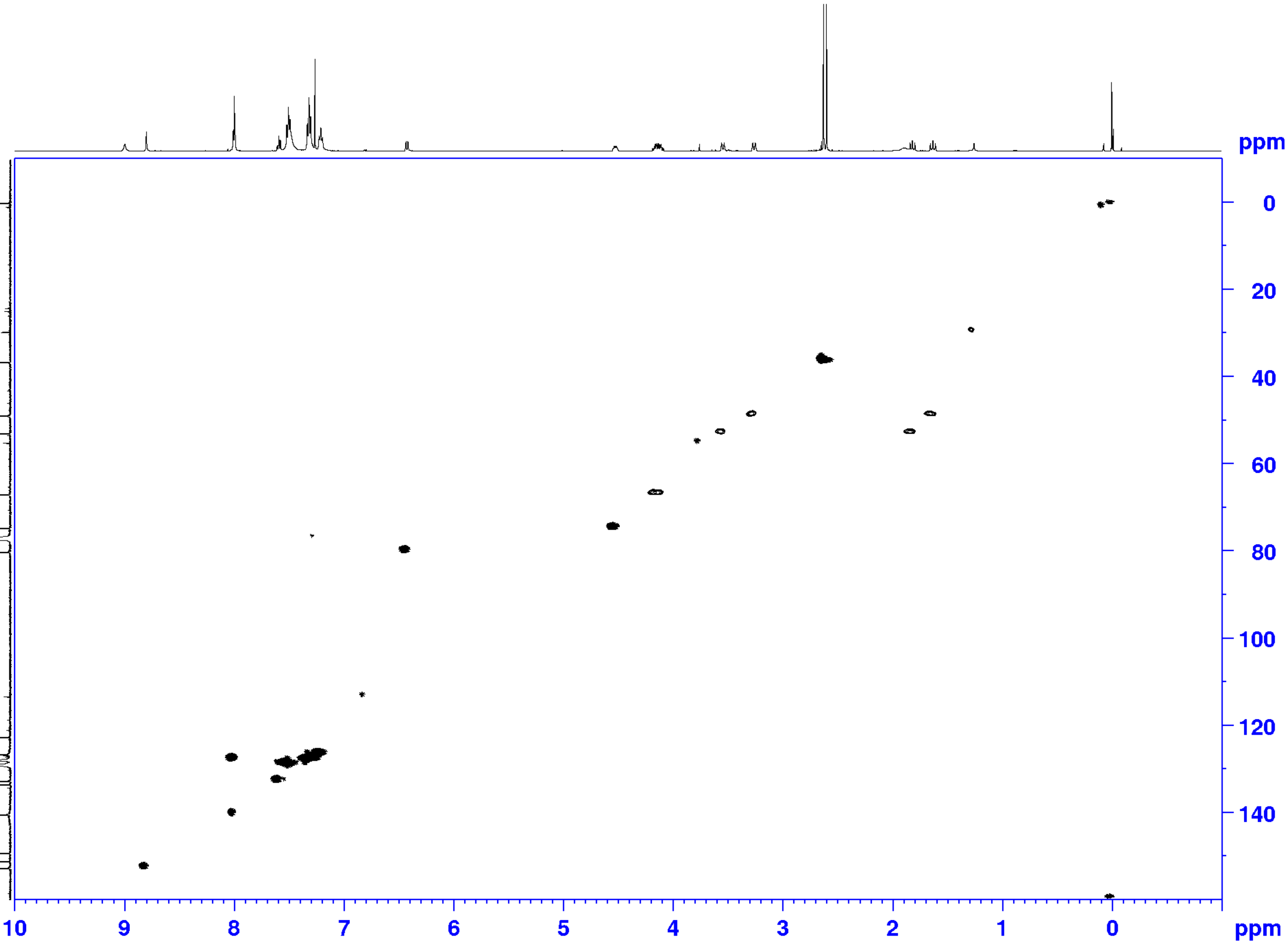

HMBC ($CDCl_3$) of (*S*p)-**16**

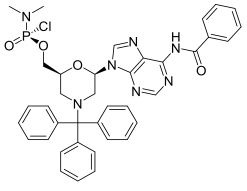

(*S*p)-**16**

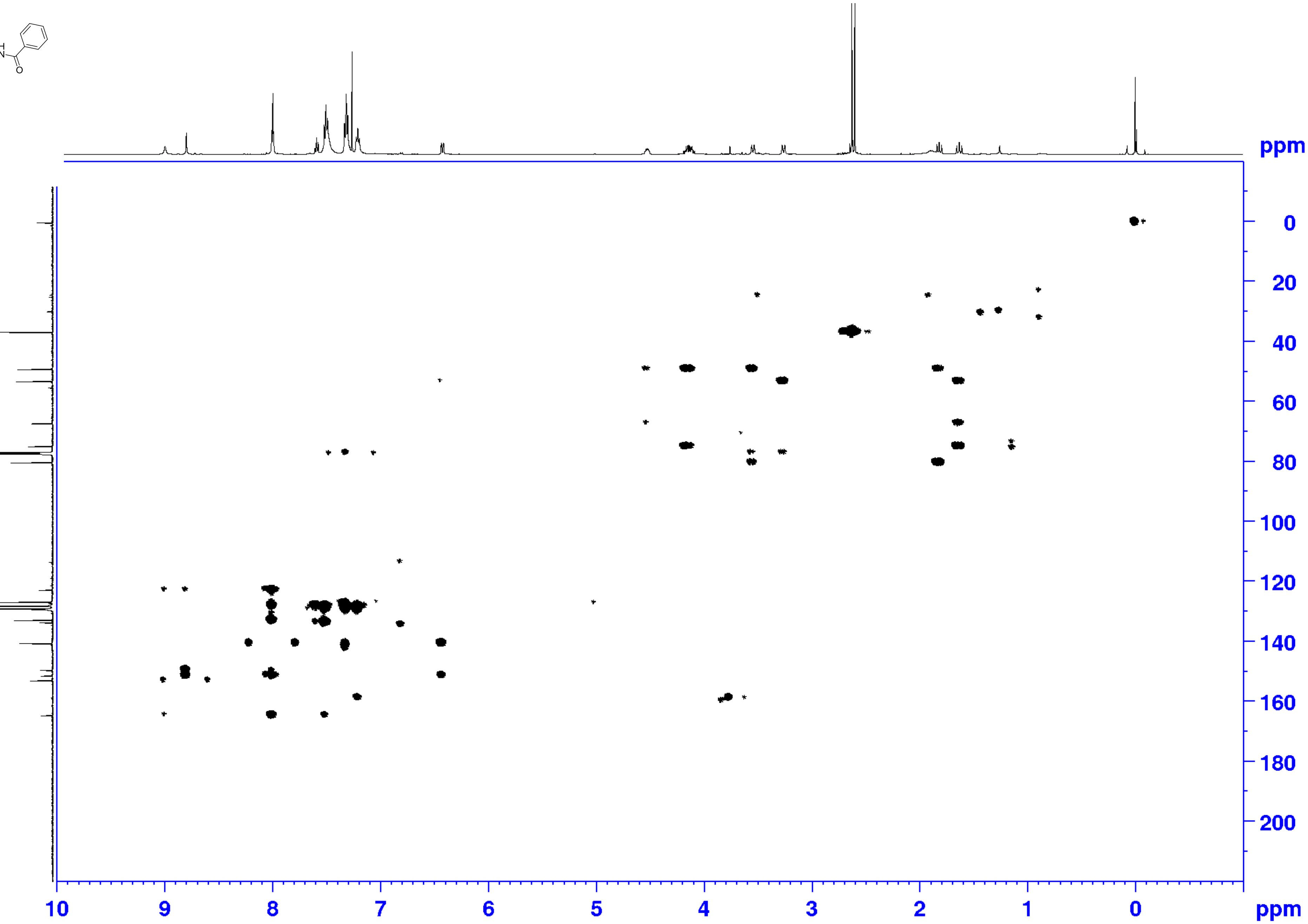

^{1}H NMR (500 MHz, $CDCl_3$) of (*R*p)-**17**

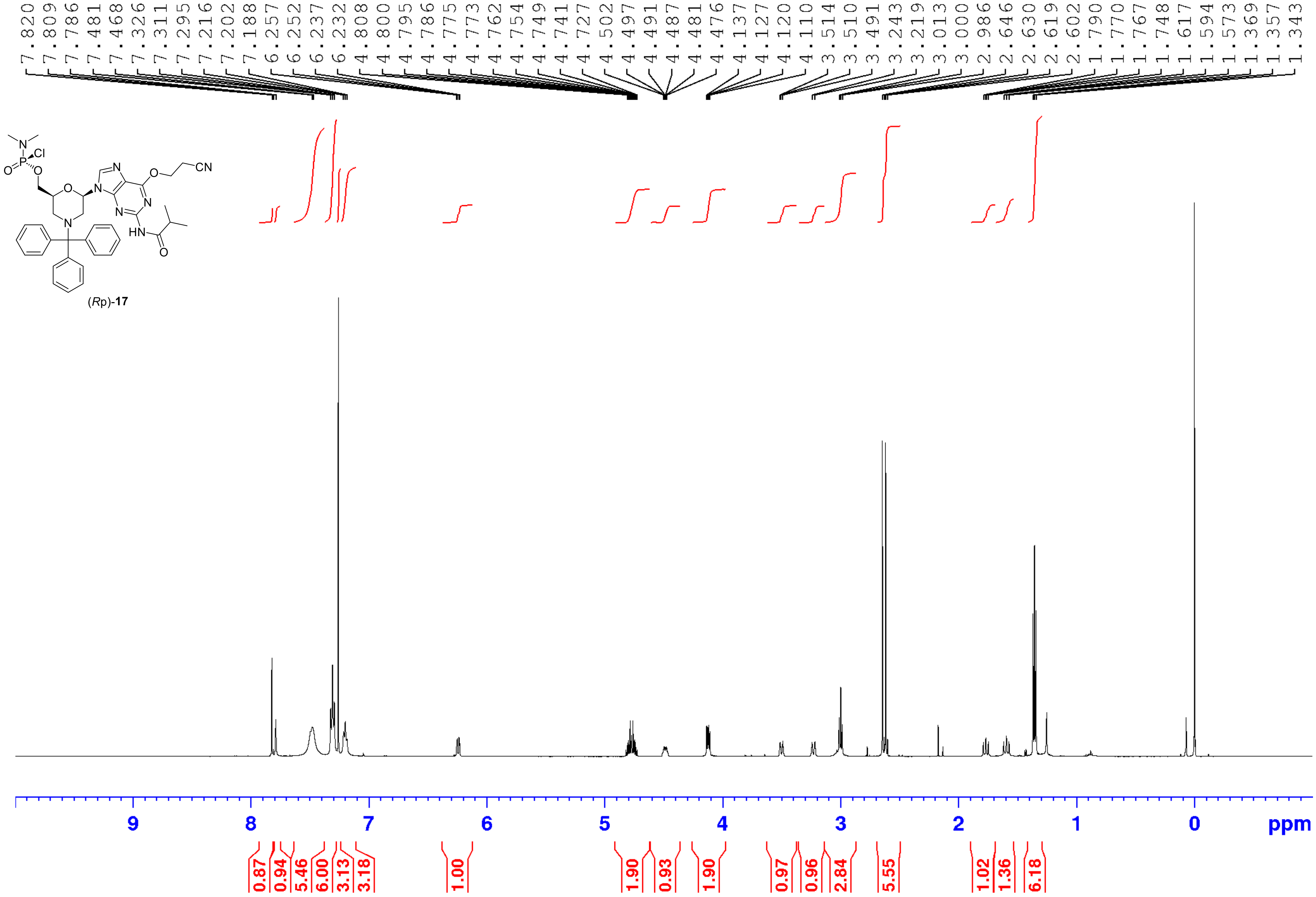

^{13}C {^{1}H} NMR (126 MHz, $CDCl_3$) of (*R*p)-**17**

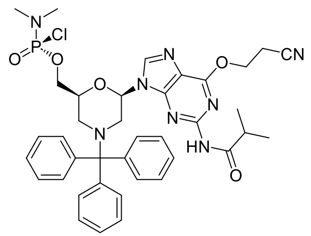

(*R*p)-**17**

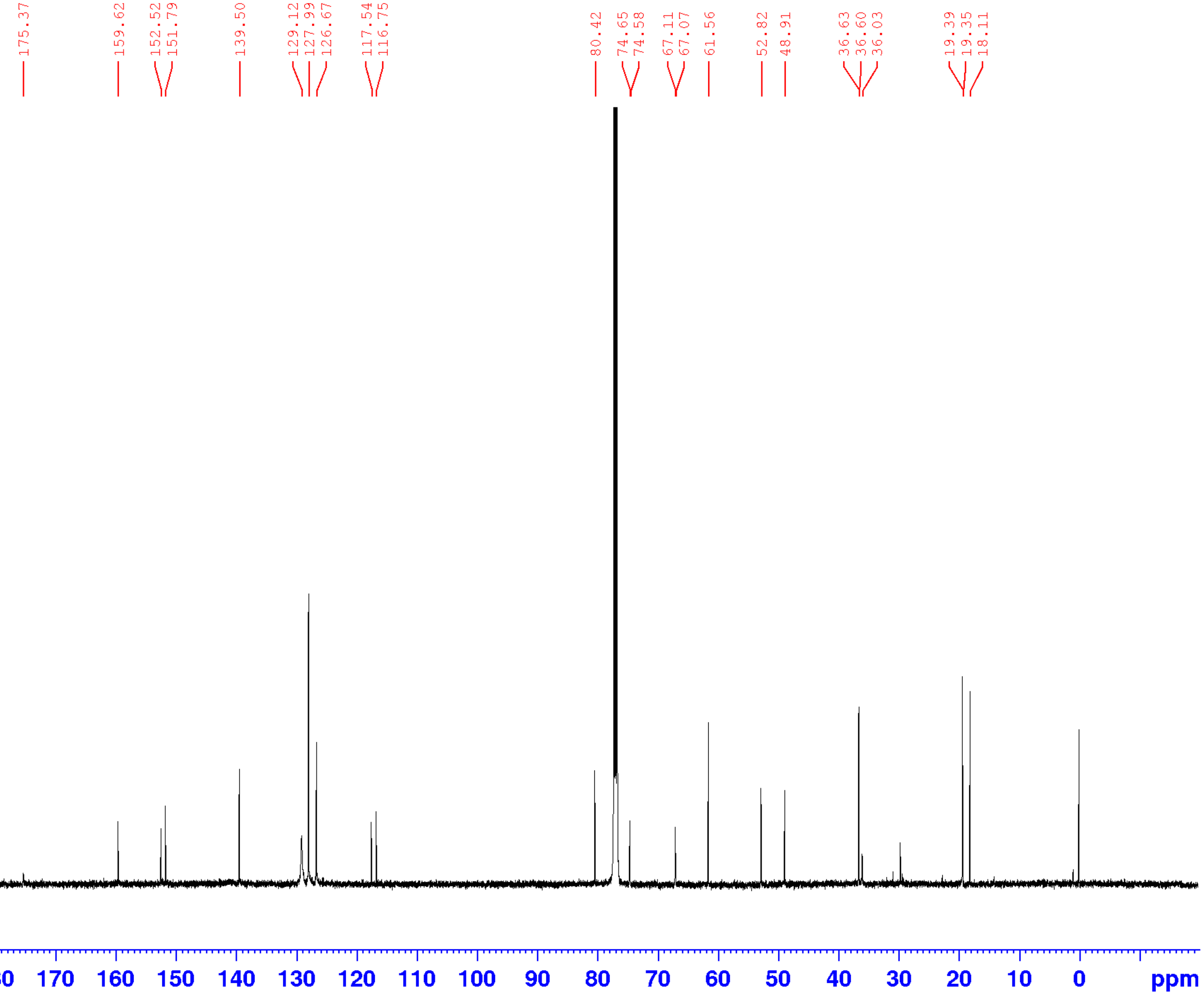

^{31}P {^{1}H} NMR (202 MHz, CDCl$_3$) of (*R*p)-**17**

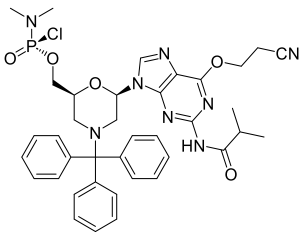

(*R*p)-**17**

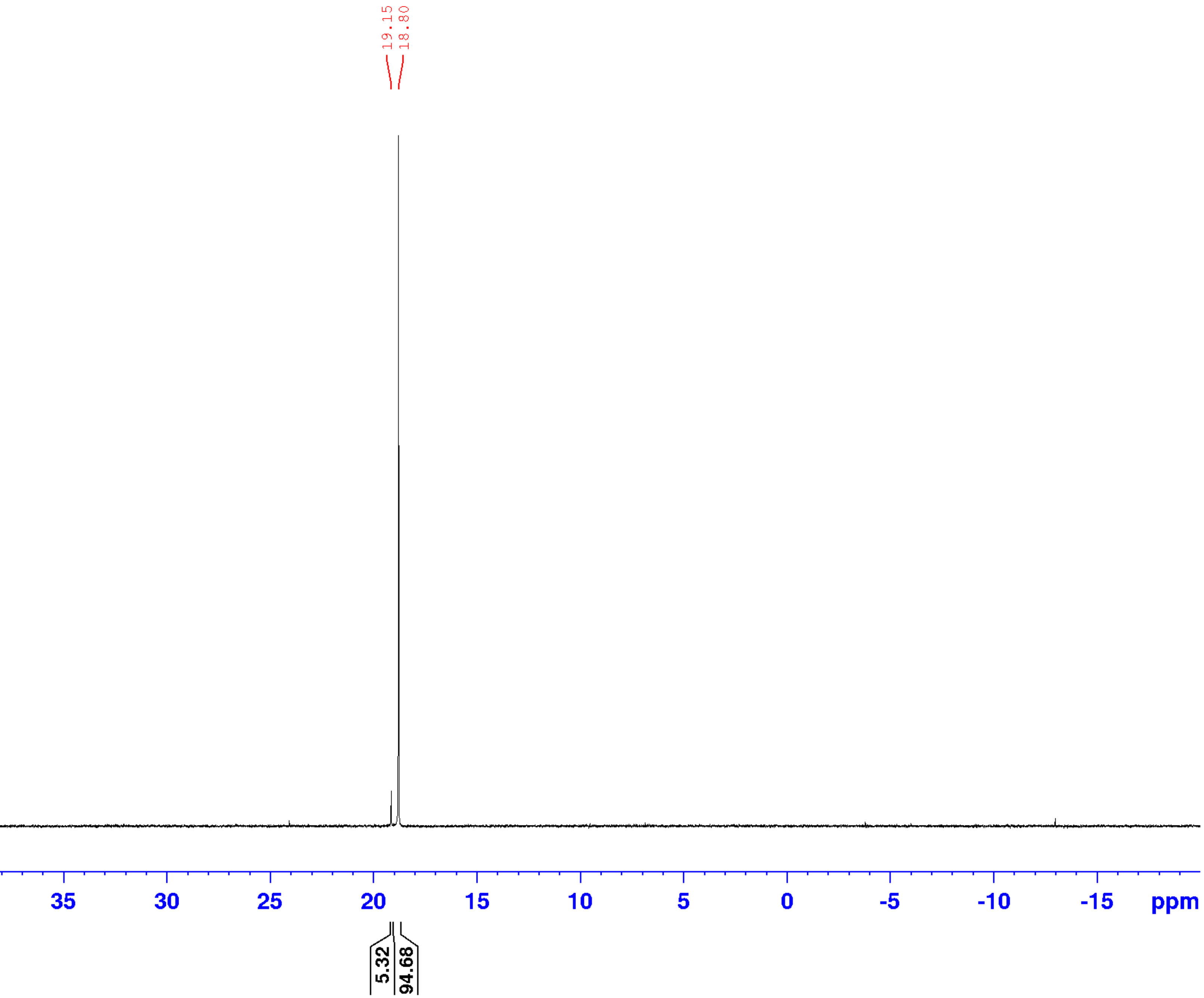

COSY ($CDCl_3$) of (*R*p)-**17**

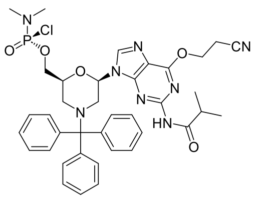

(*R*p)-**17**

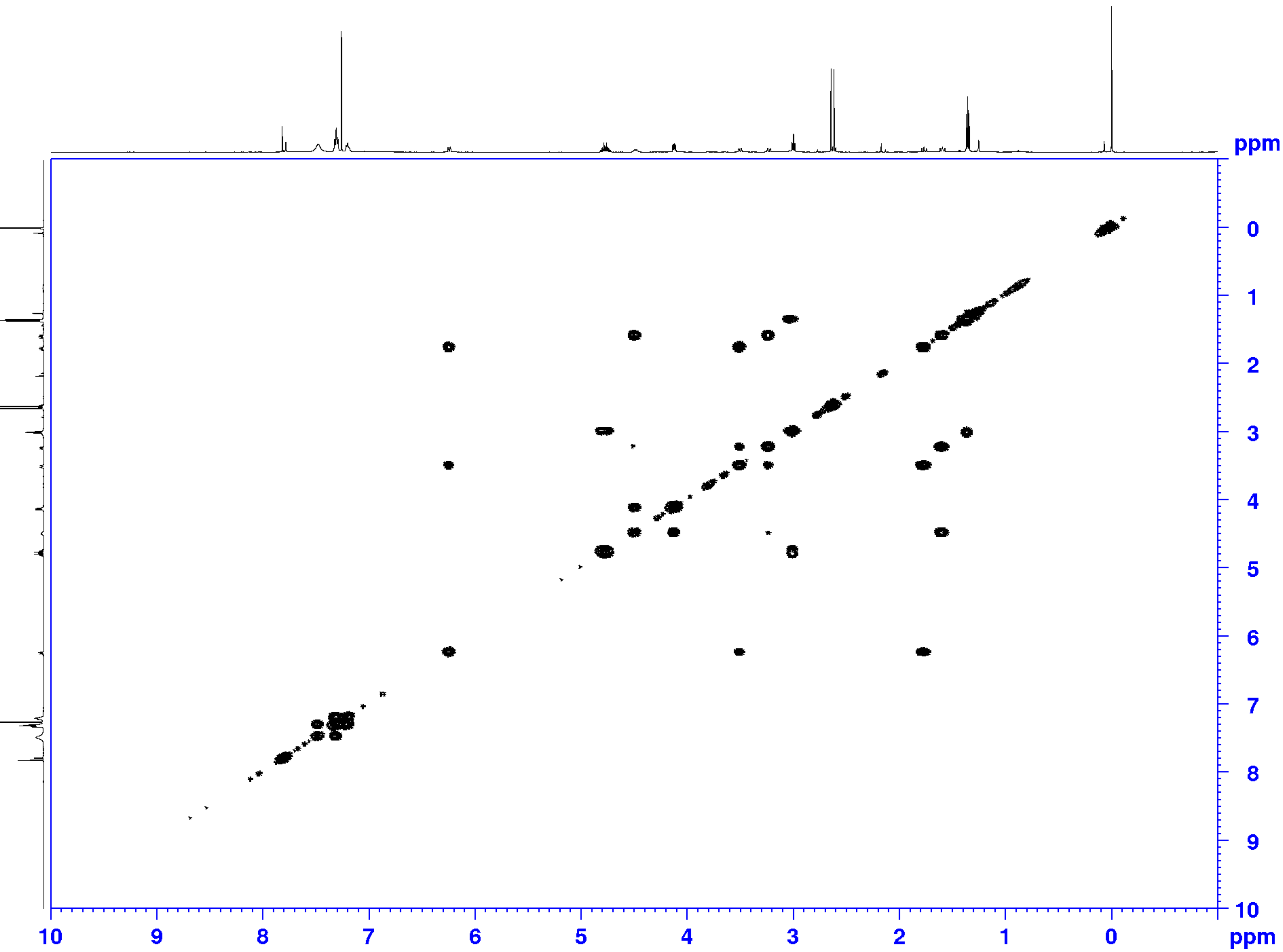

HSQC ($CDCl_3$) of (*R*p)-**17**

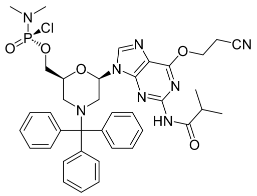

(*R*p)-**17**

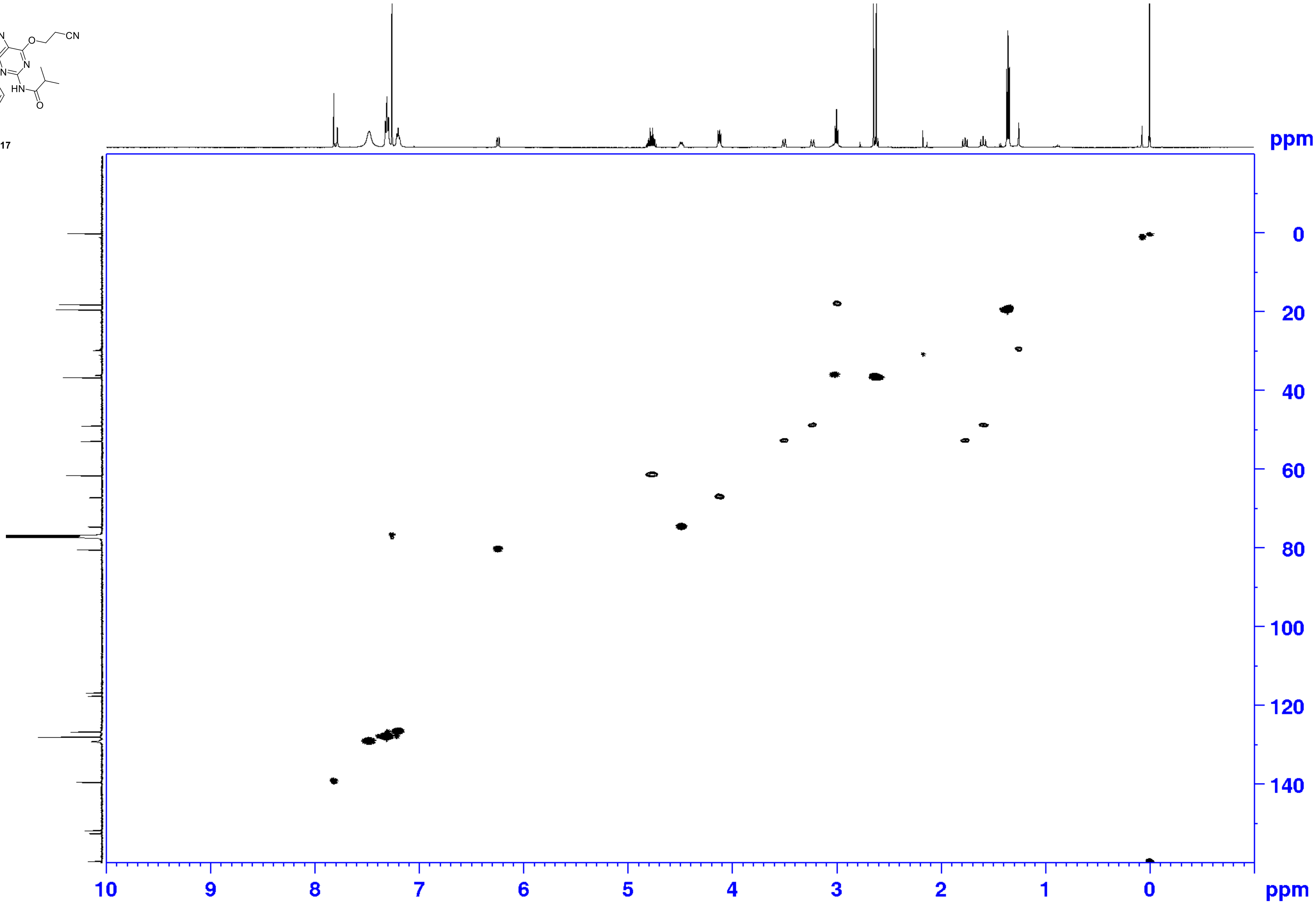

HMBC (CDCl$_3$) of (*R*p)-**17**

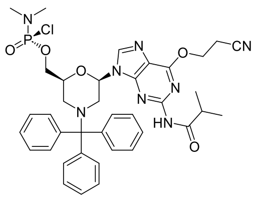

(*R*p)-**17**

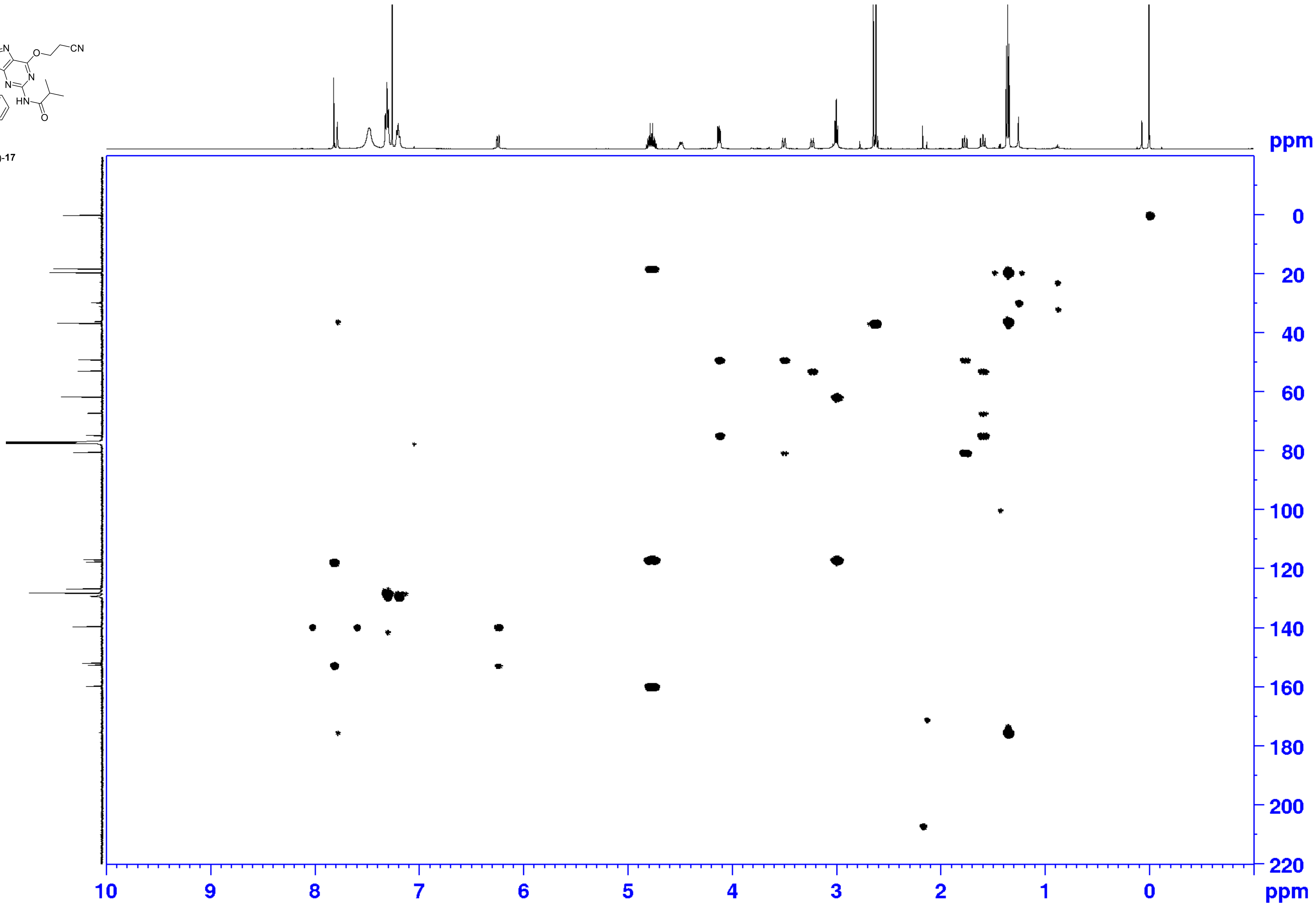

^{1}H NMR (500 MHz, CDCl$_3$) of (*S*p)-**17**

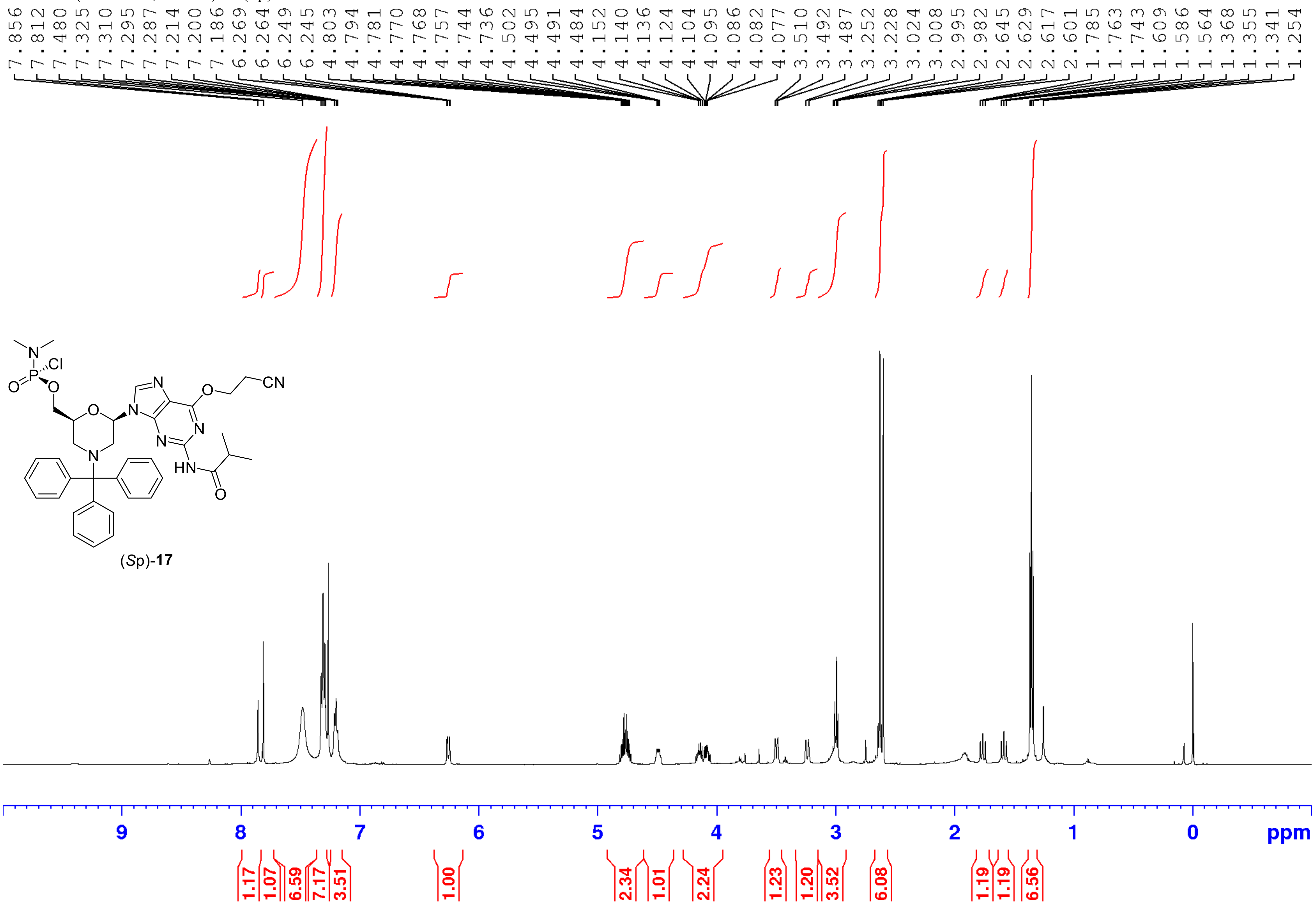

$^{13}C\ \{^{1}H\}$ NMR (126 MHz, $CDCl_3$) of (*S*p)-**17**

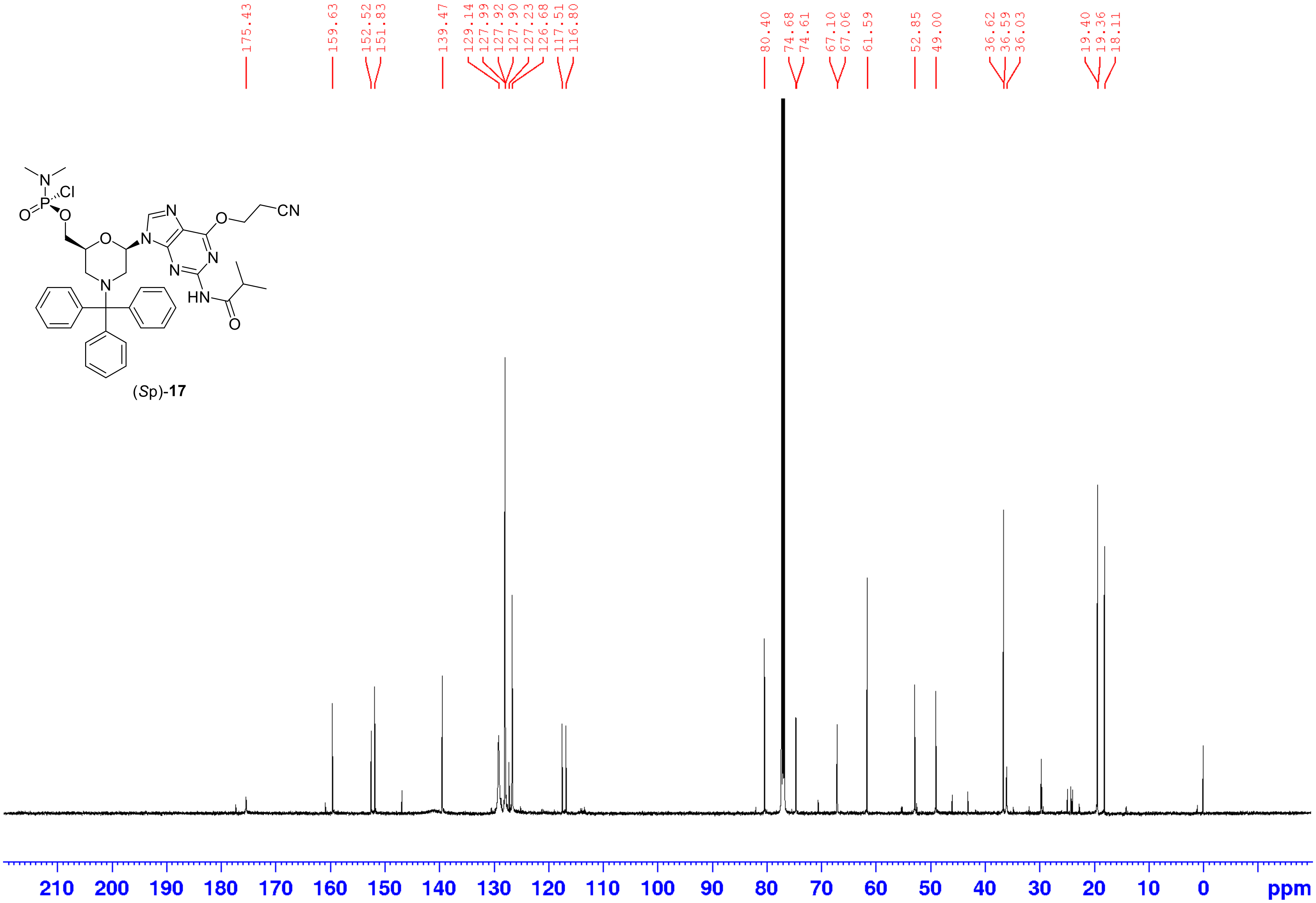

^{31}P {^{1}H} NMR (202 MHz, $CDCl_3$) of (*S*p)-**17**

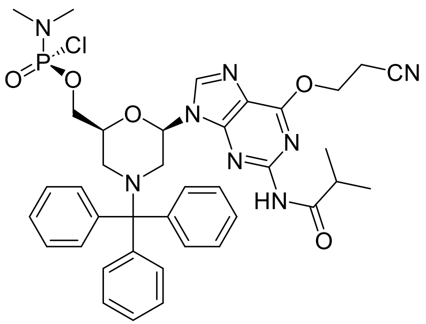

(*S*p)-**17**

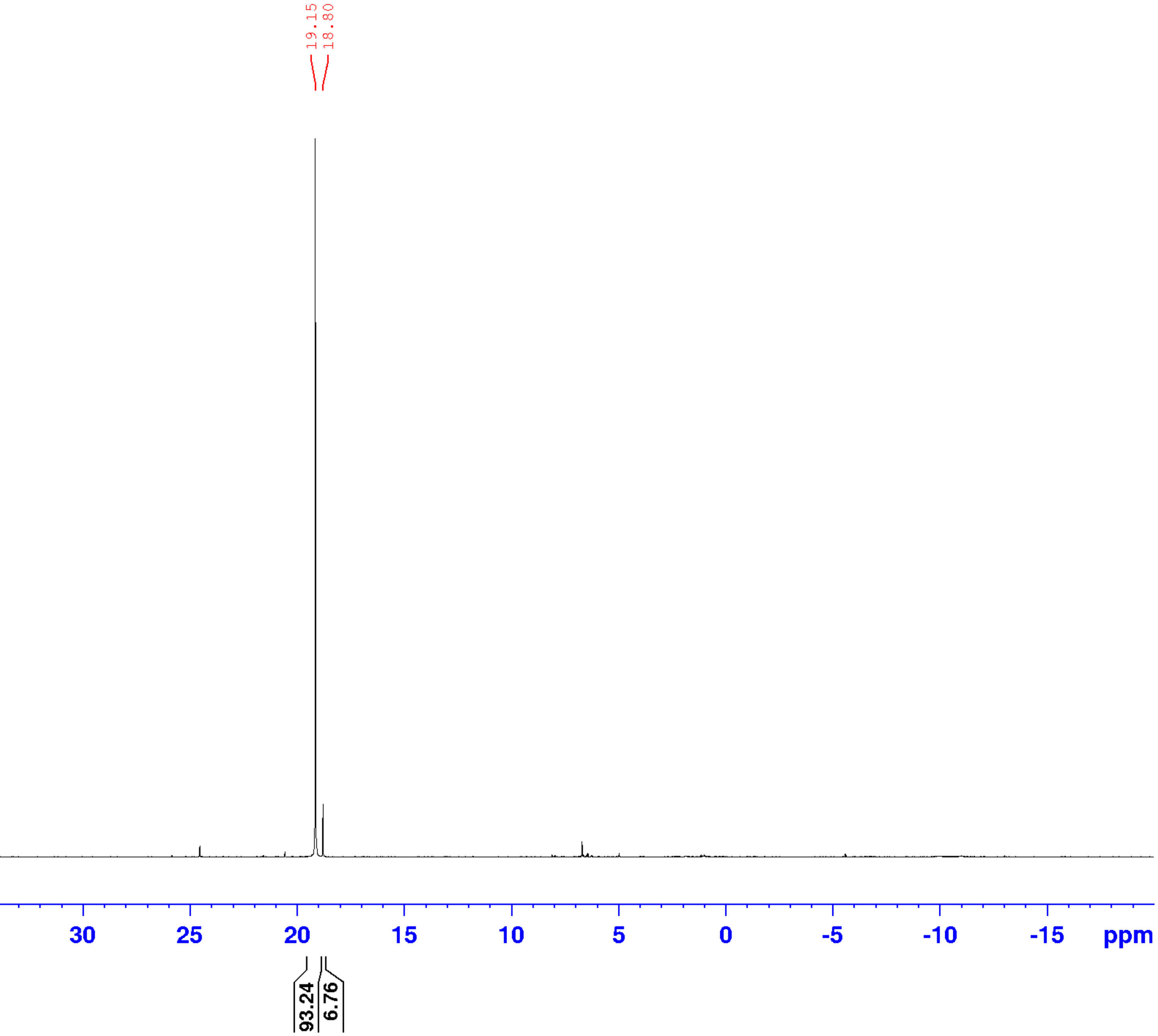

COSY ($CDCl_3$) of (*S*p)-**17**

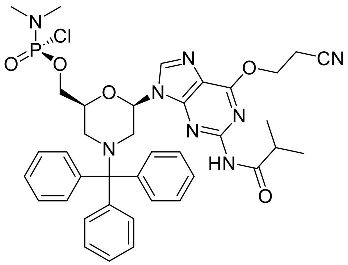

(*S*p)-**17**

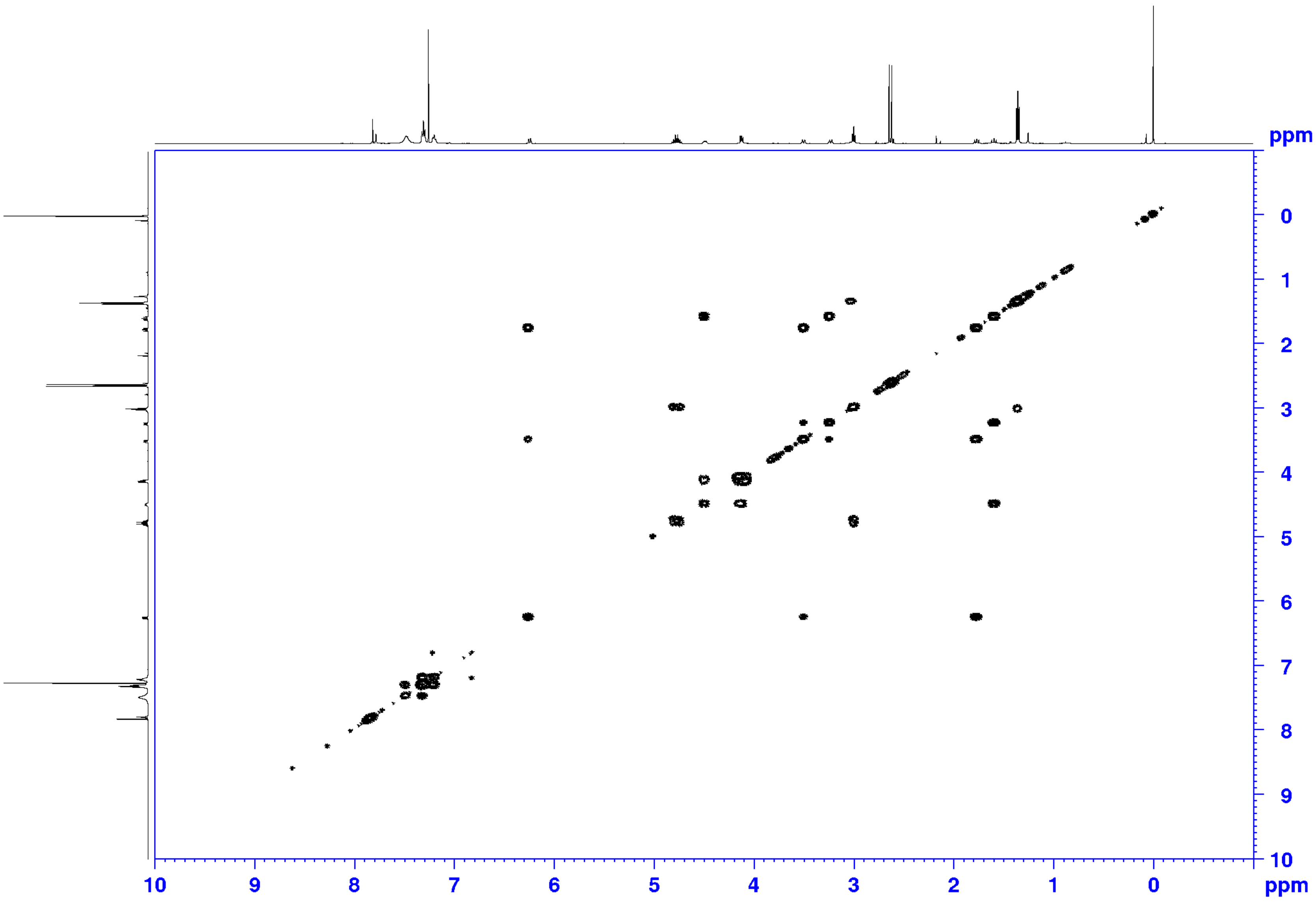

HSQC ($CDCl_3$) of (*S*p)-**17**

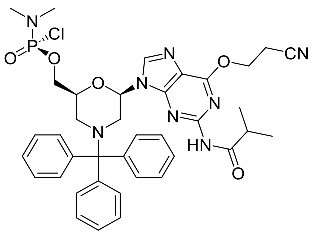

(*S*p)-**17**

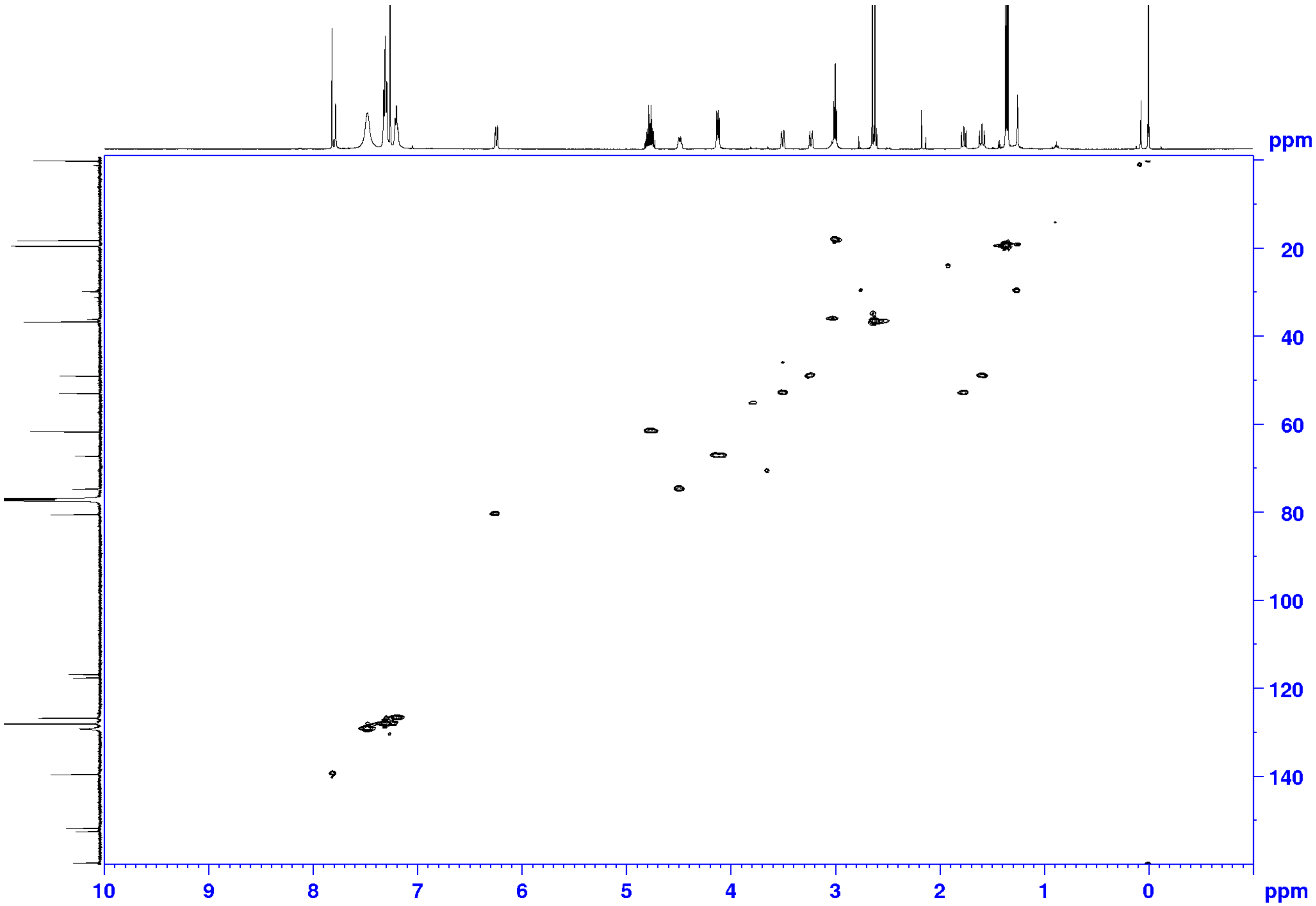

HMBC ($CDCl_3$) of (*S*p)-**17**

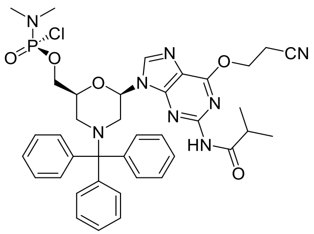

(*S*p)-**17**

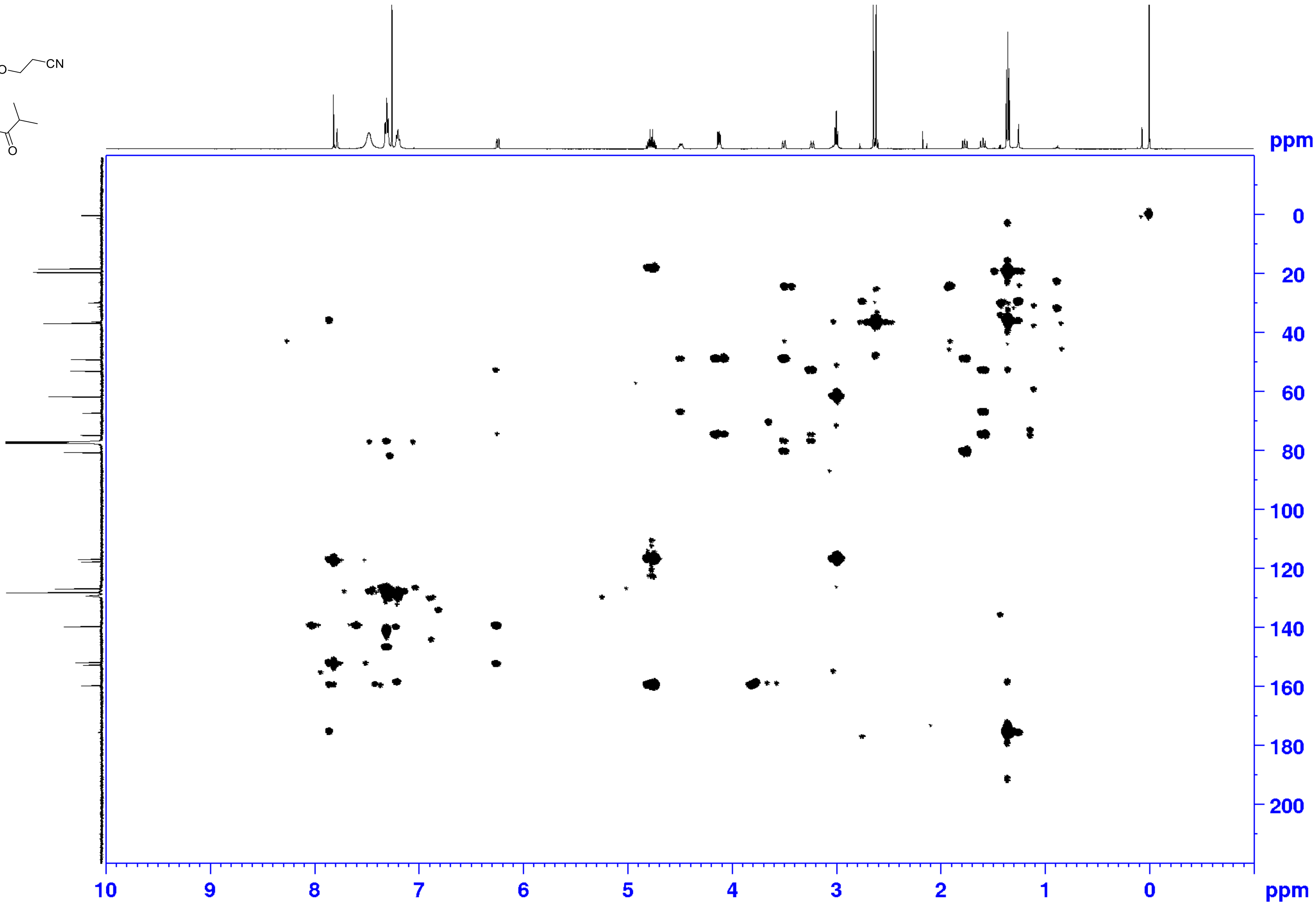

^{1}H NMR (500 MHz, CDCl$_3$) of (*R*p)-**18**

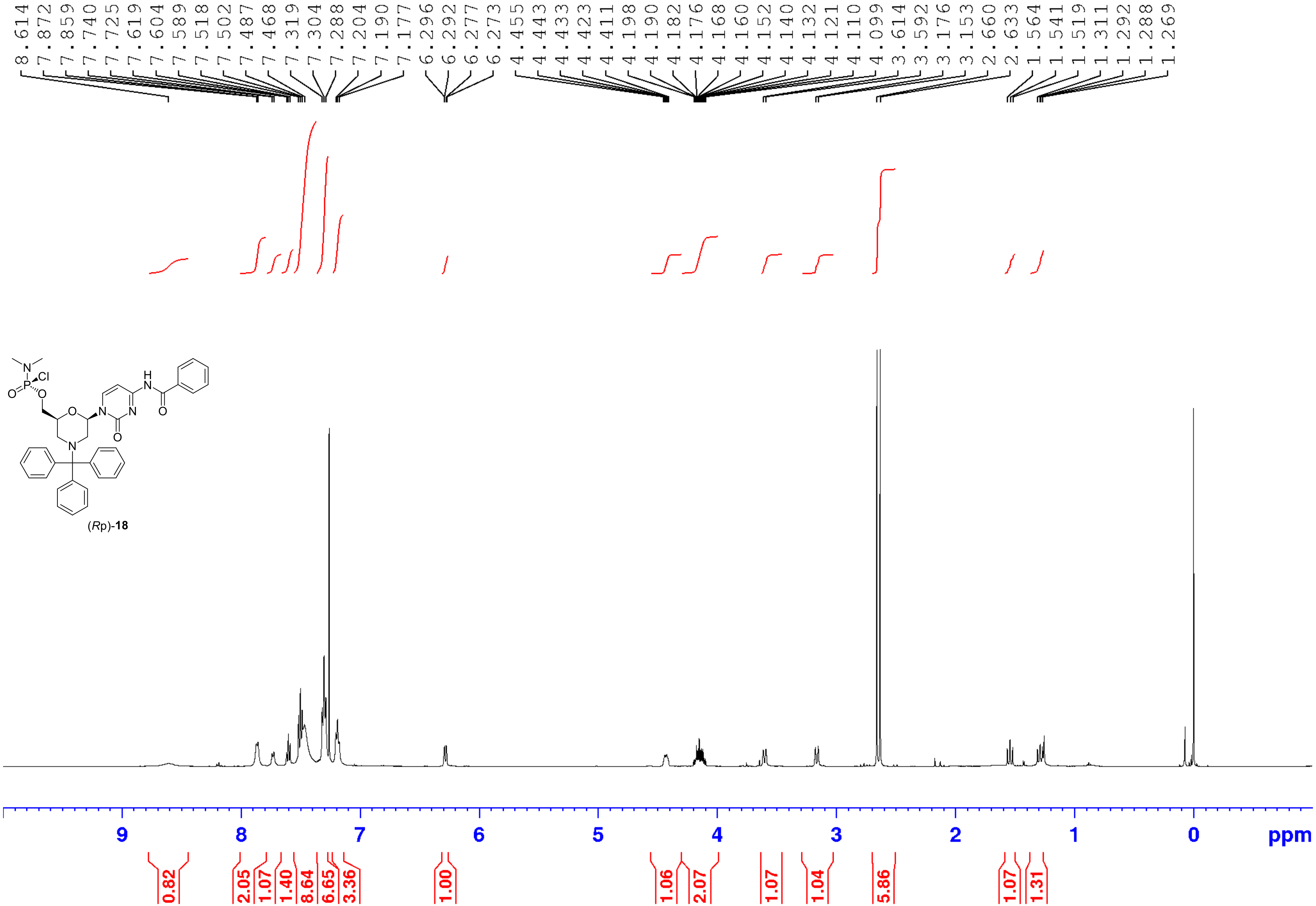

^{13}C {^{1}H} NMR (126 MHz, $CDCl_3$) of (*R*p)-**18**

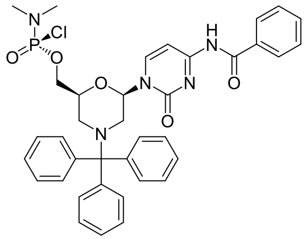

(*R*p)-**18**

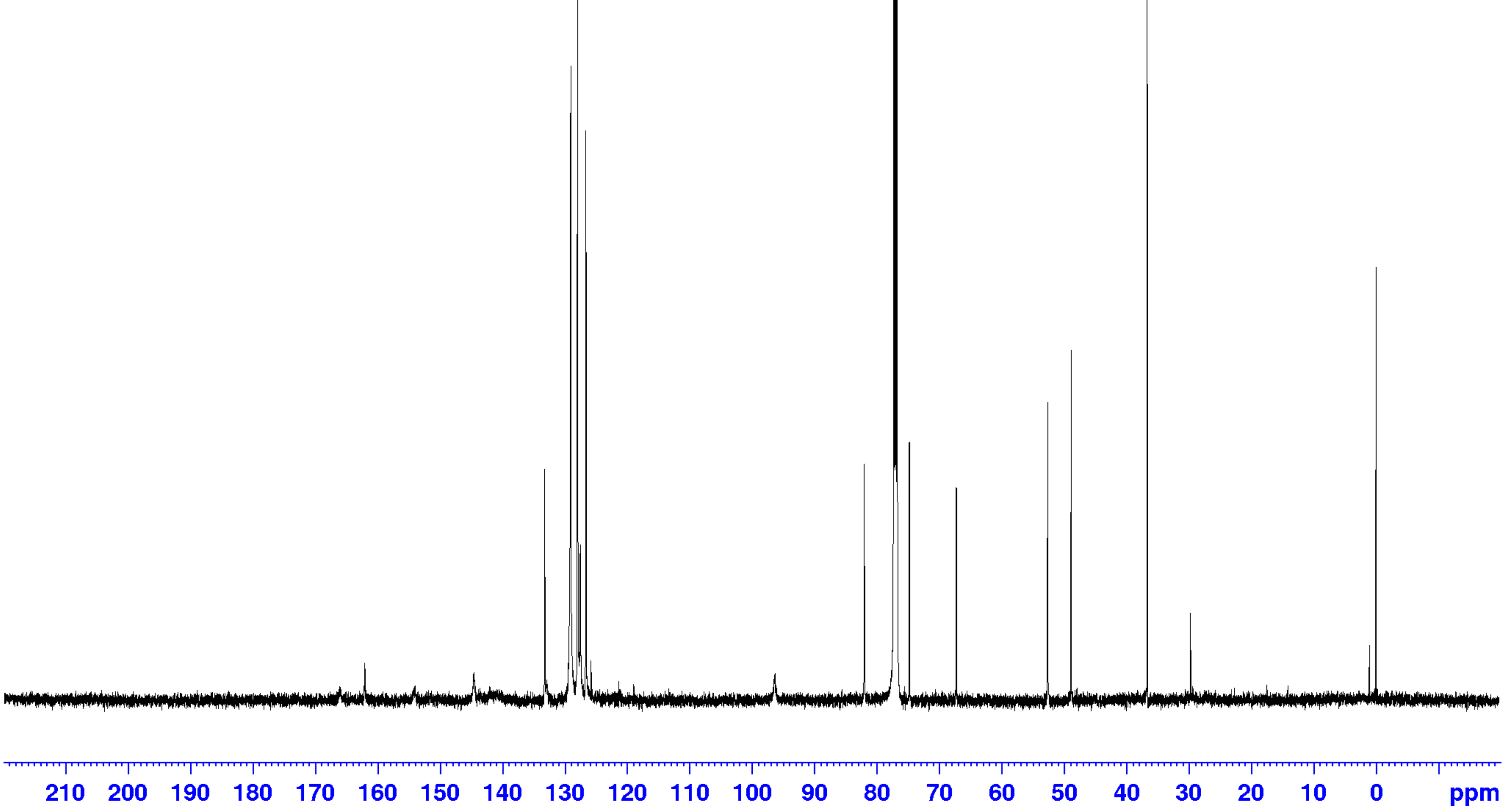

^{31}P {^{1}H} NMR (202 MHz, $CDCl_3$) of (*R*p)-**18**

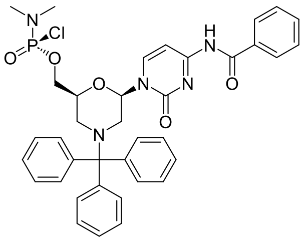

(*R*p)-**18**

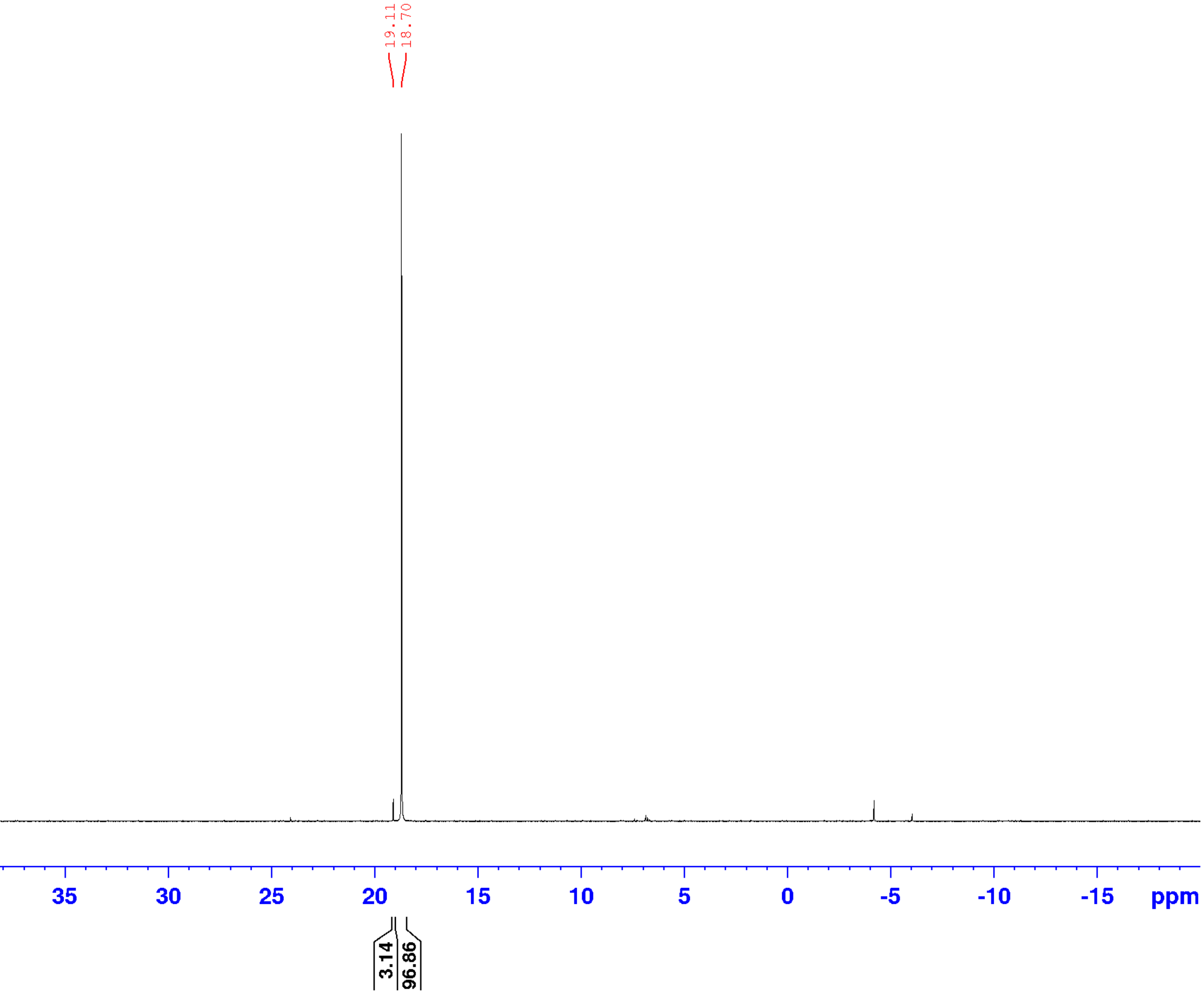

COSY ($CDCl_3$) of (*R*p)-**18**

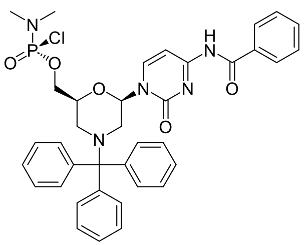

(*R*p)-**18**

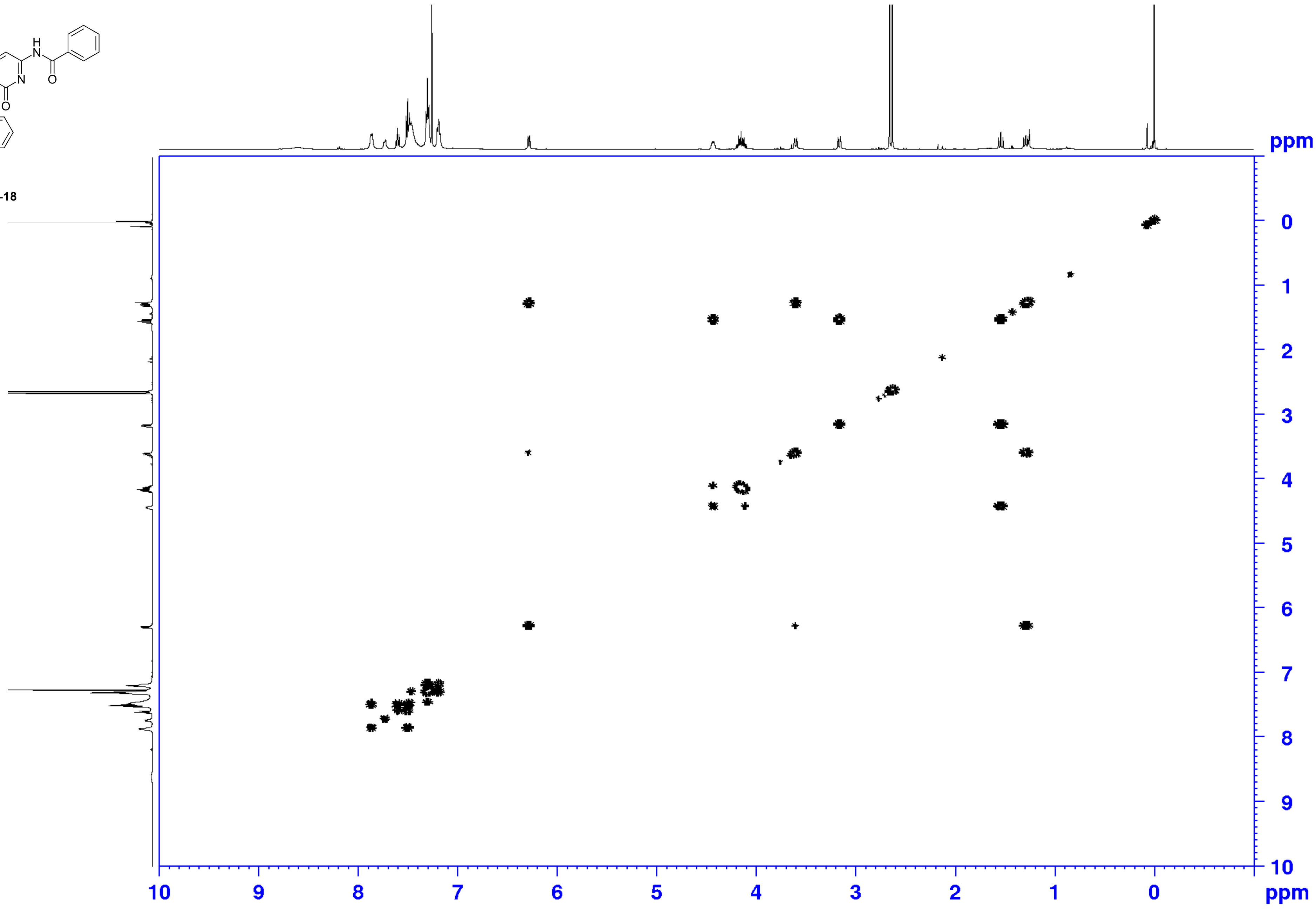

HSQC ($CDCl_3$) of (*R*p)-**18**

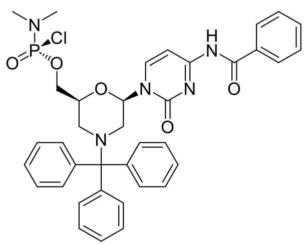

(*R*p)-**18**

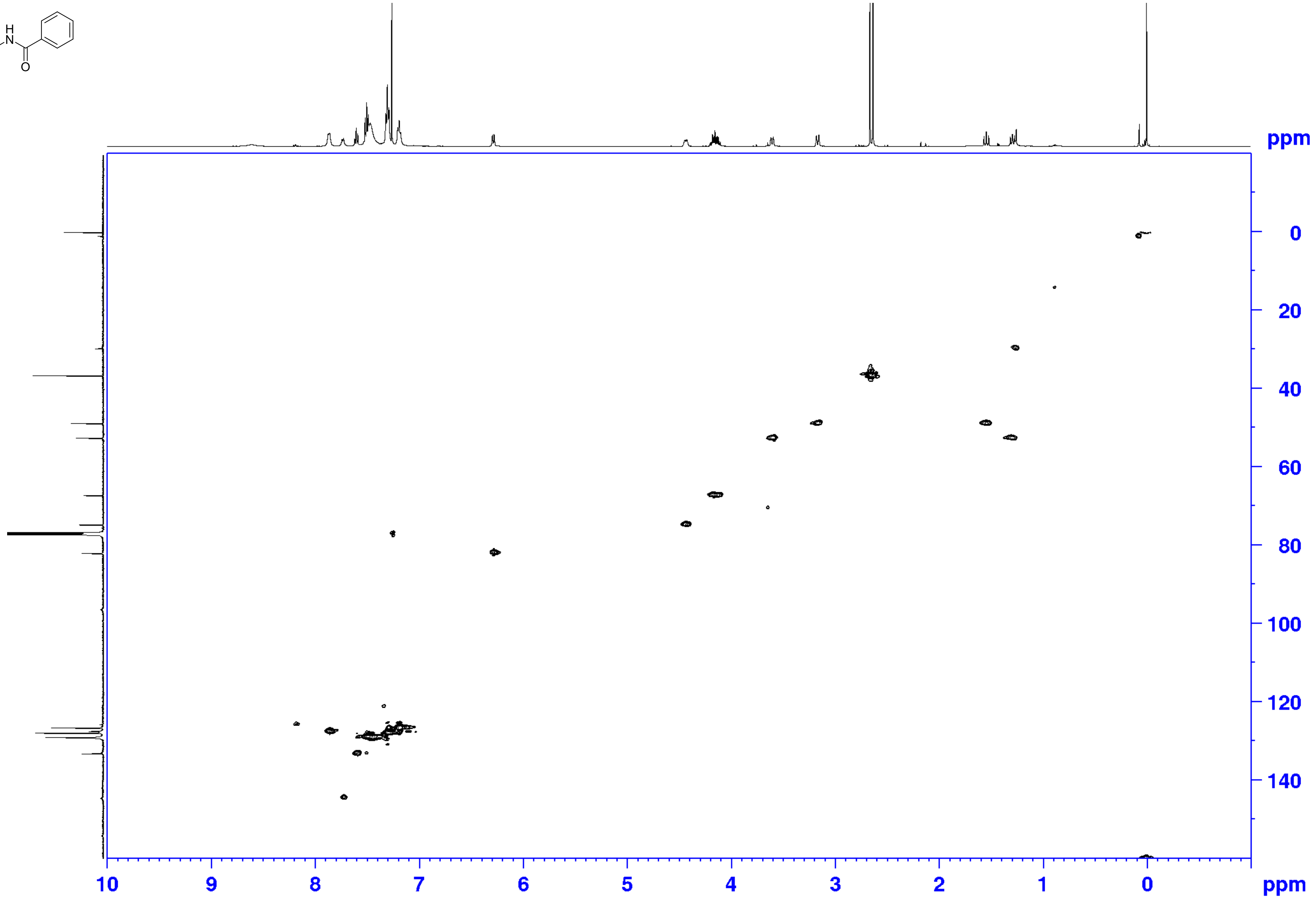

HMBC ($CDCl_3$) of (*R*p)-**18**

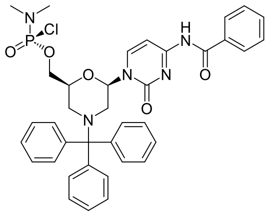

(*R*p)-**18**

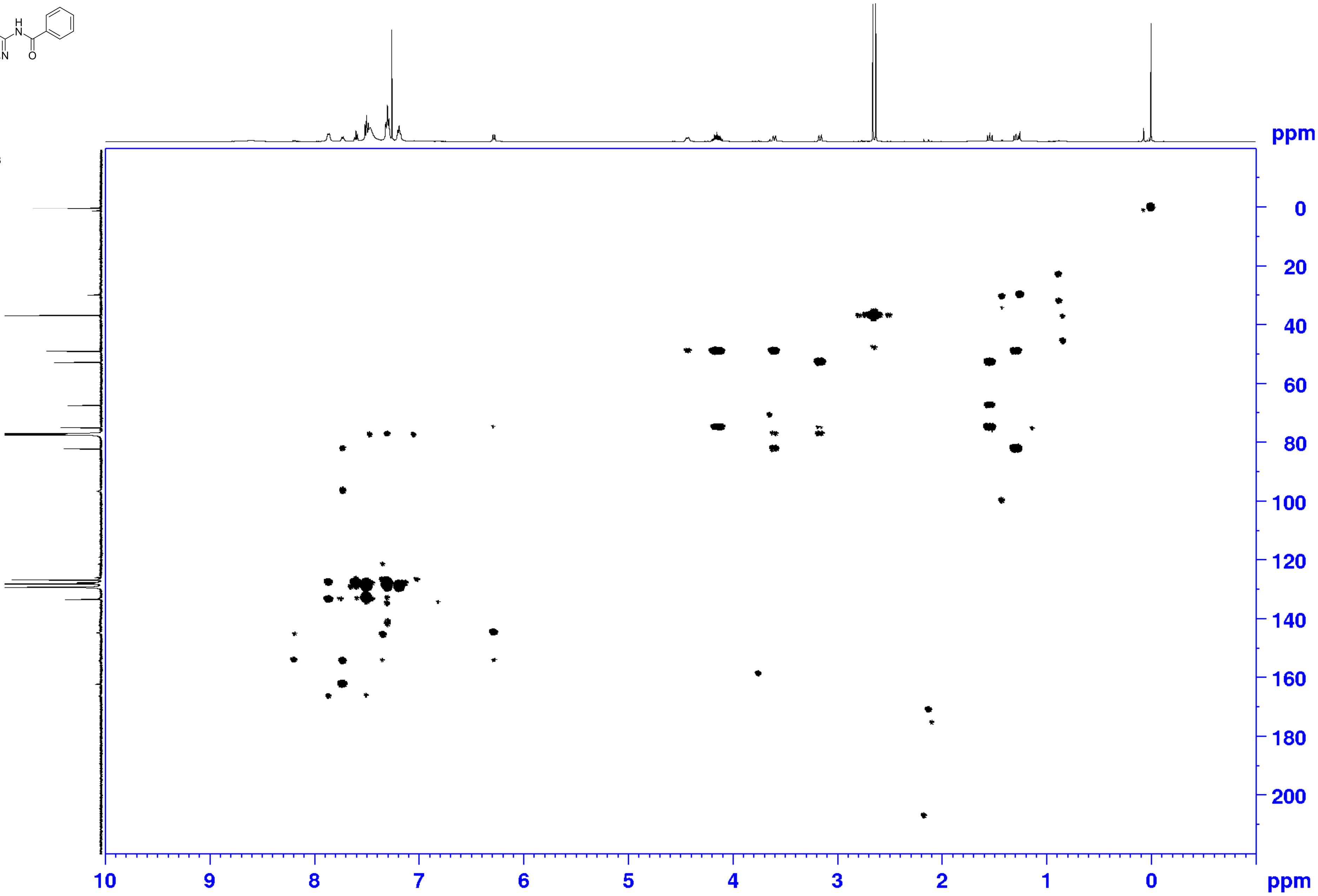

^{1}H NMR (500 MHz, $CDCl_3$) of (*S*p)-**18**

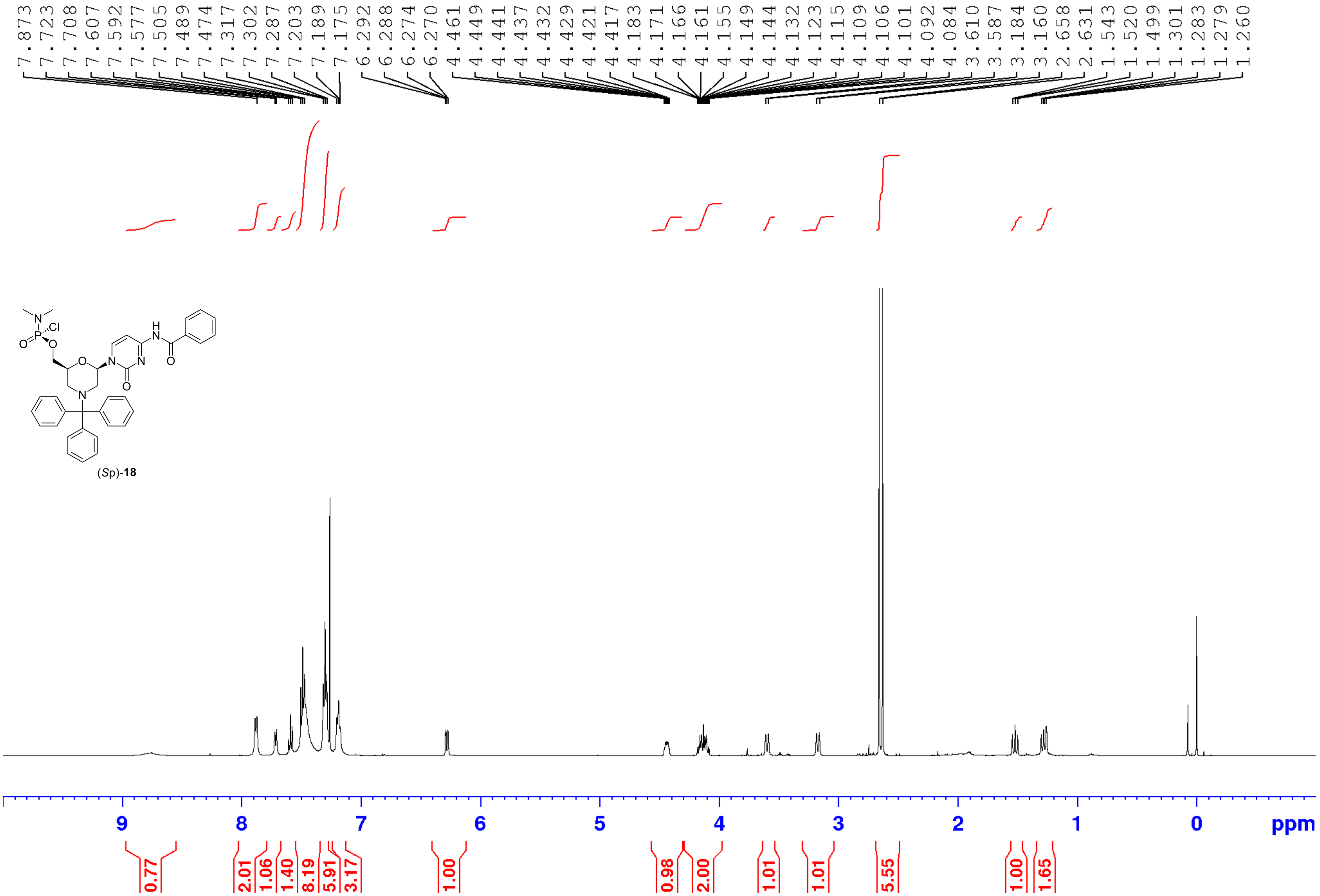

(*S*p)-**18**

^{13}C {^{1}H} NMR (126 MHz, $CDCl_3$) of (*S*p)-**18**

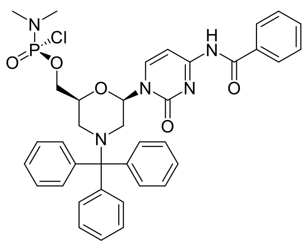

(*S*p)-**18**

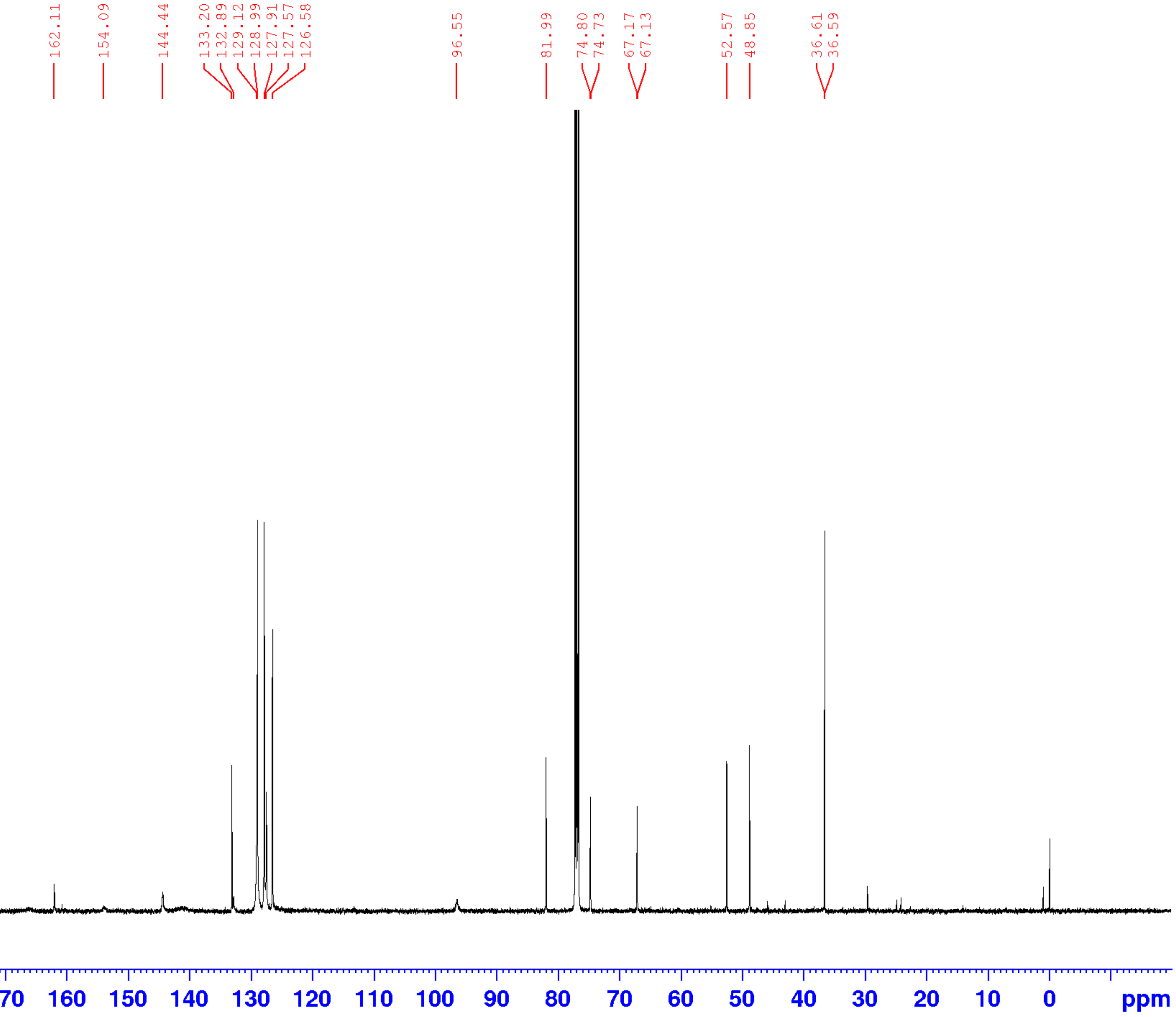

^{31}P {^{1}H} NMR (202 MHz, $CDCl_3$) of (*S*p)-**18**

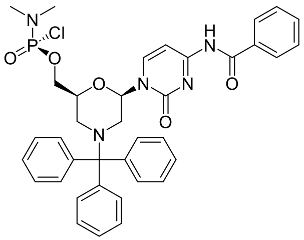

(*S*p)-**18**

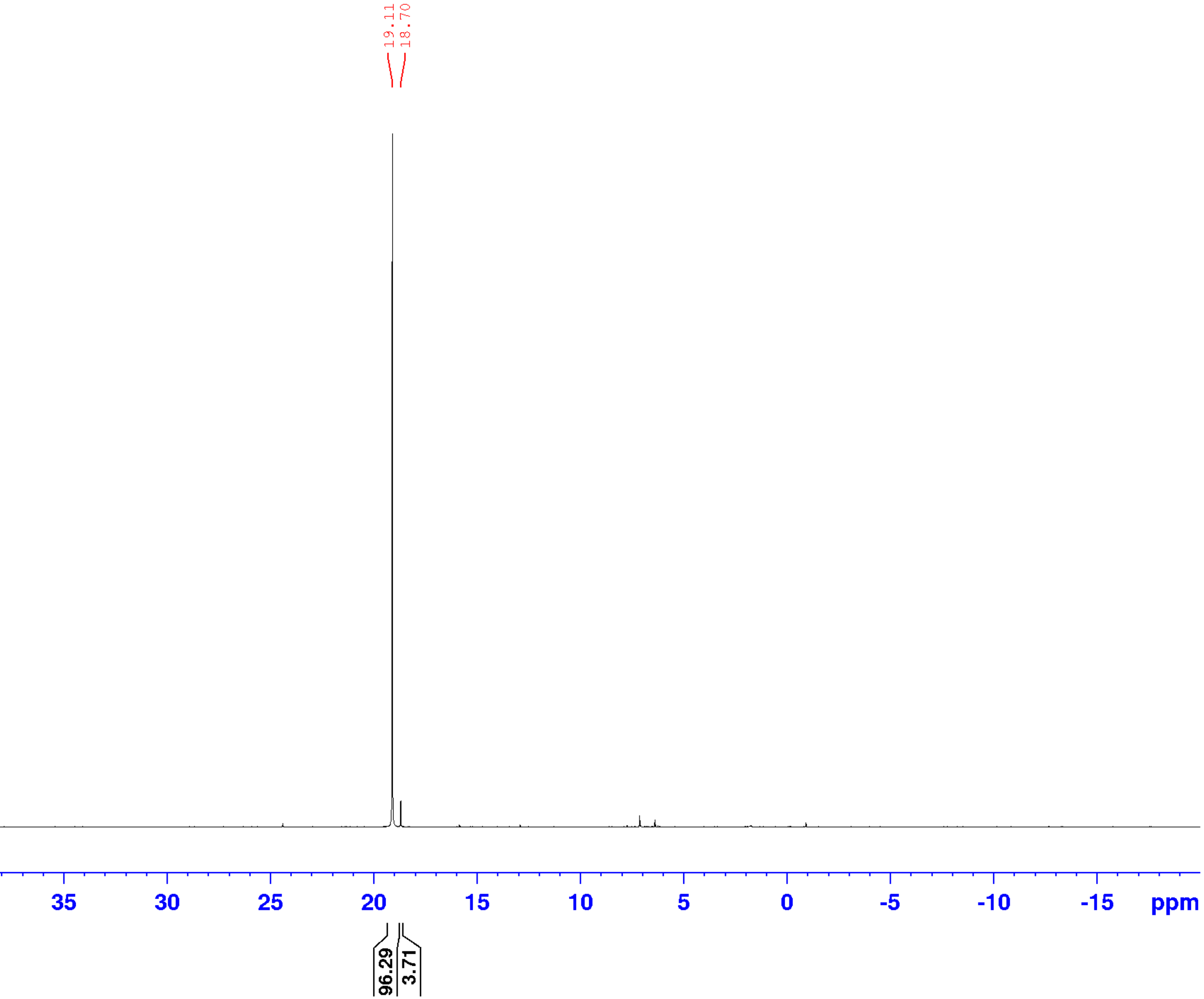

COSY ($CDCl_3$) of (*S*p)-**18**

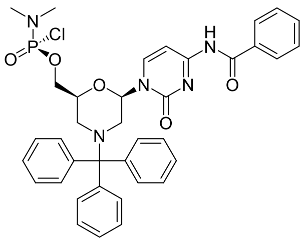

(*S*p)-**18**

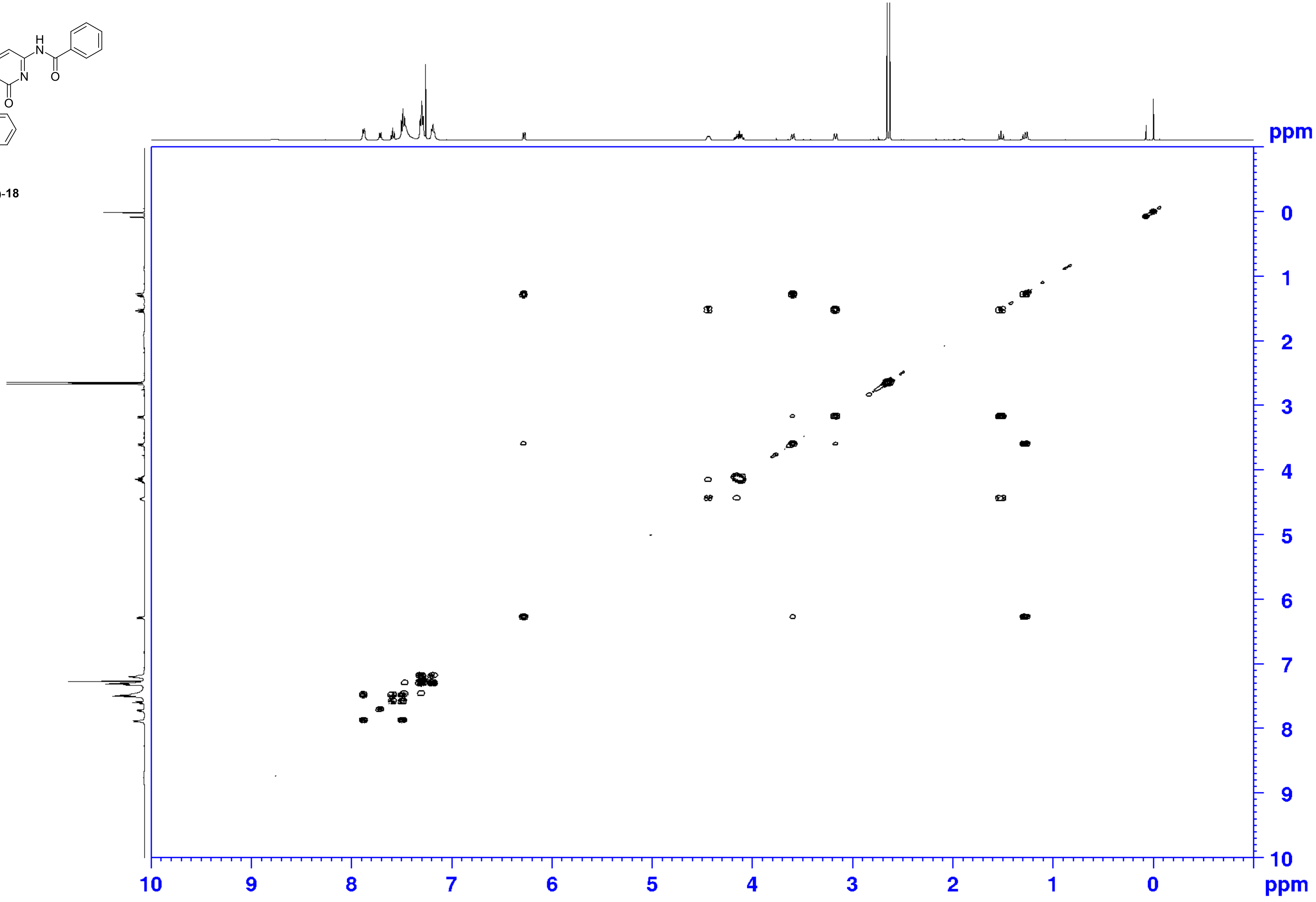

HSQC ($CDCl_3$) of (*S*p)-**18**

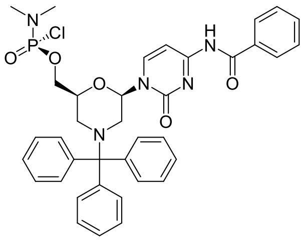

(*S*p)-**18**

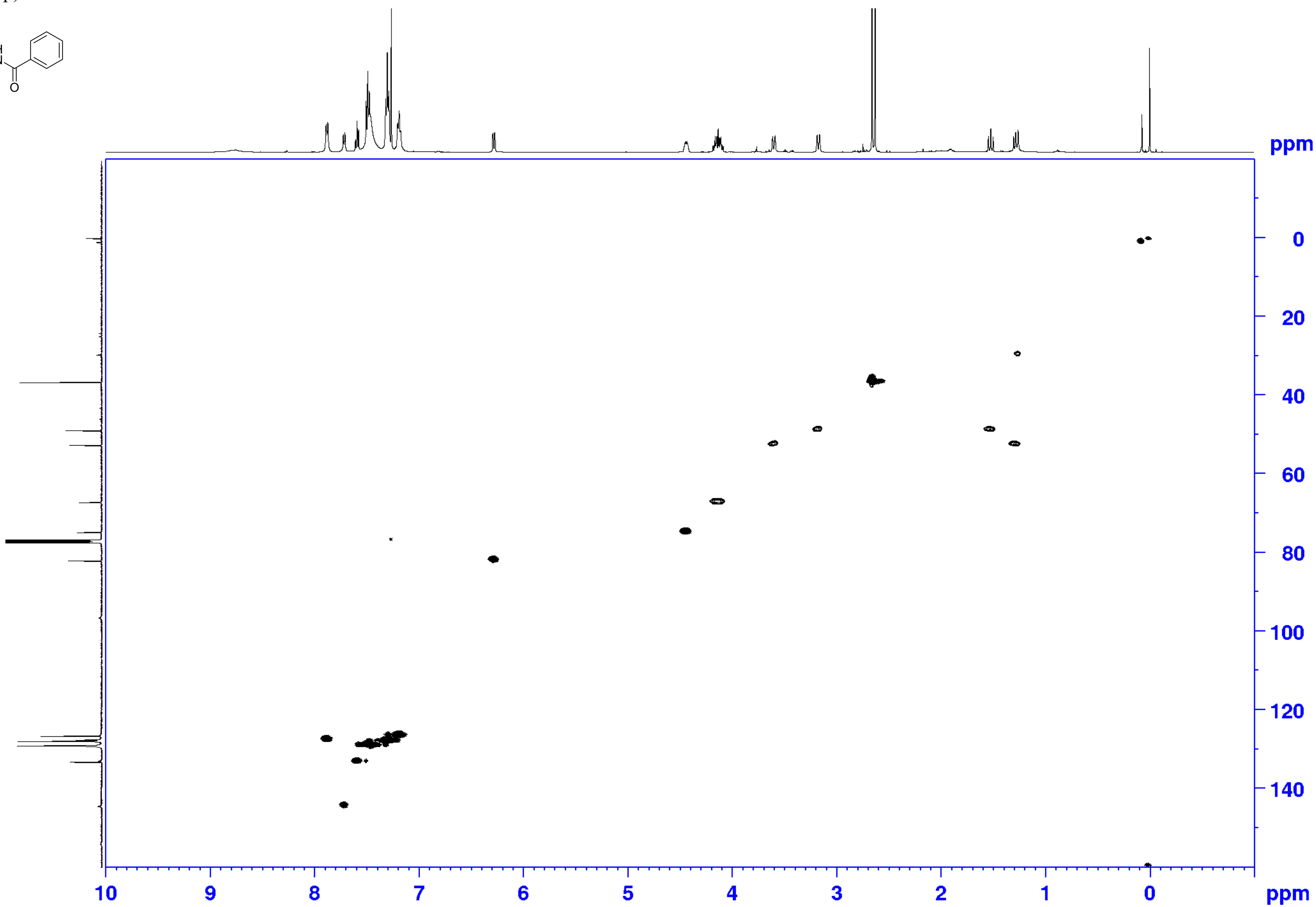

HMBC ($CDCl_3$) of (*S*p)-**18**

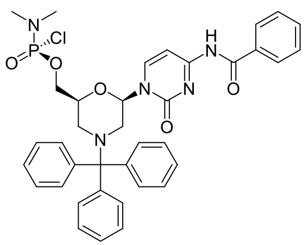

(*S*p)-**18**

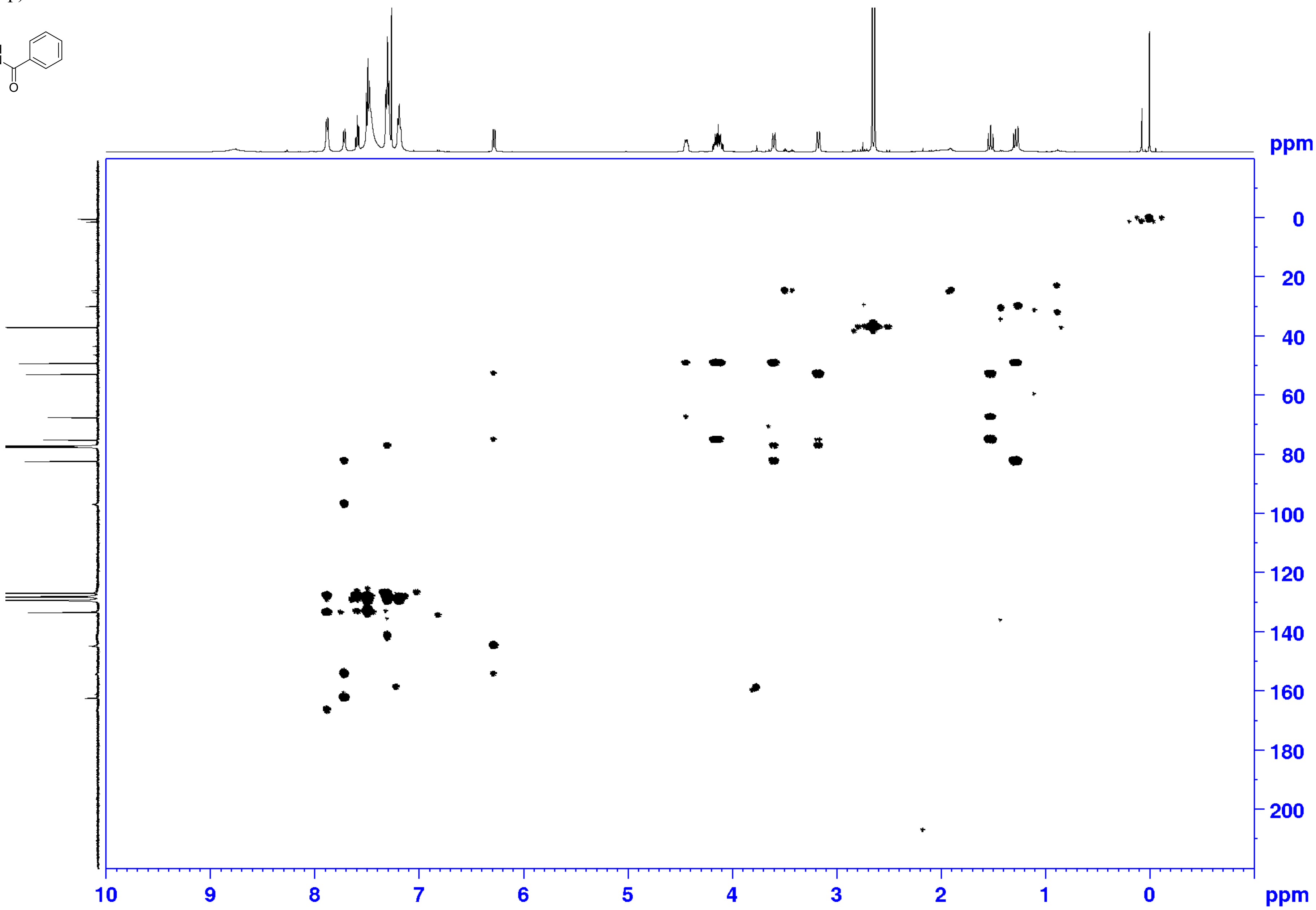

^{1}H NMR (500 MHz, $CDCl_3$) of (*R*p)-**18**

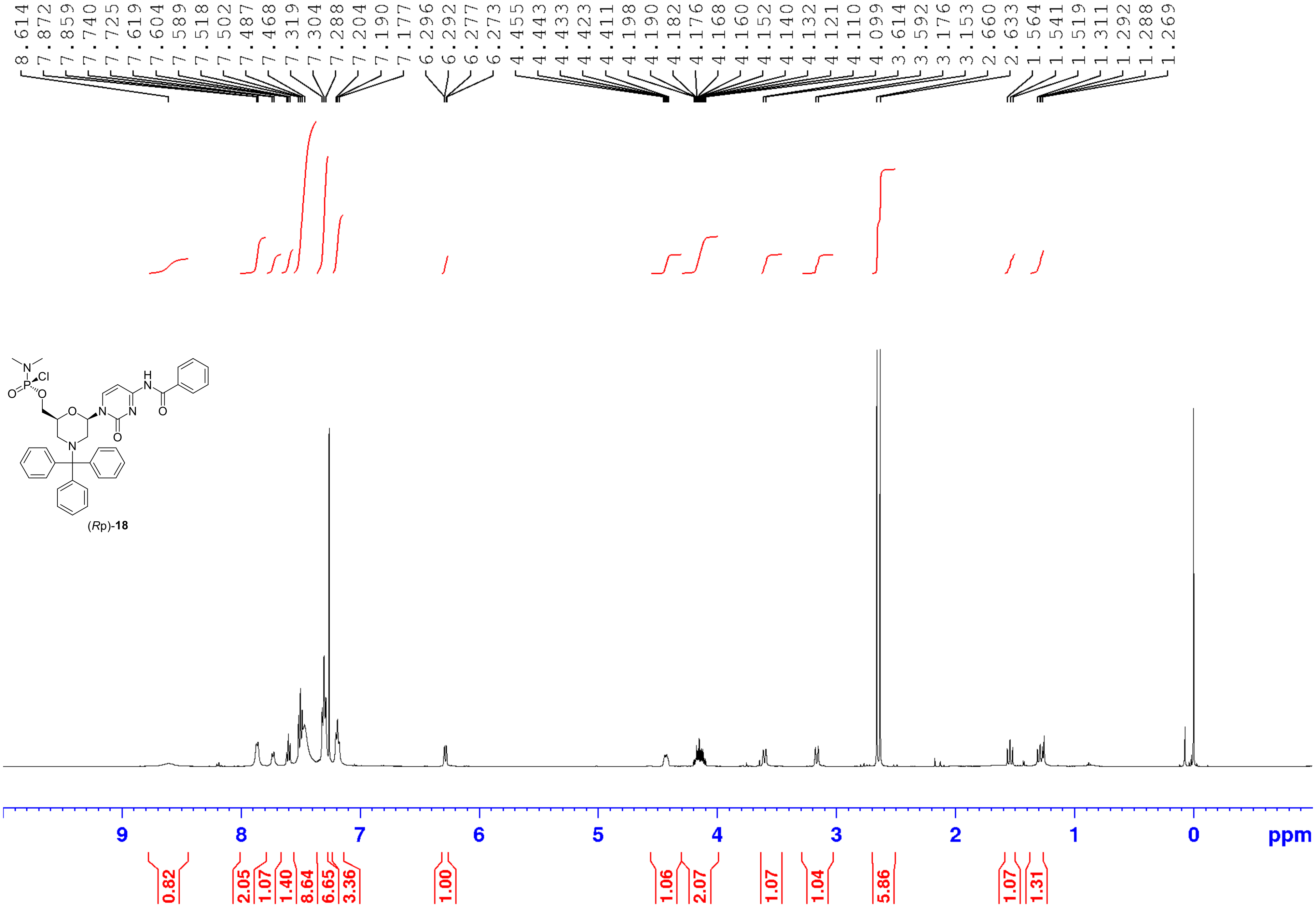

$^{13}C\ \{^1H\}$ NMR (126 MHz, $CDCl_3$) of (*R*p)-**18**

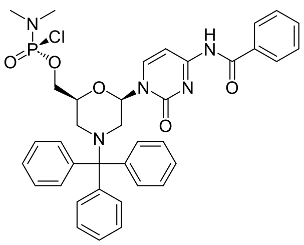

(*R*p)-**18**

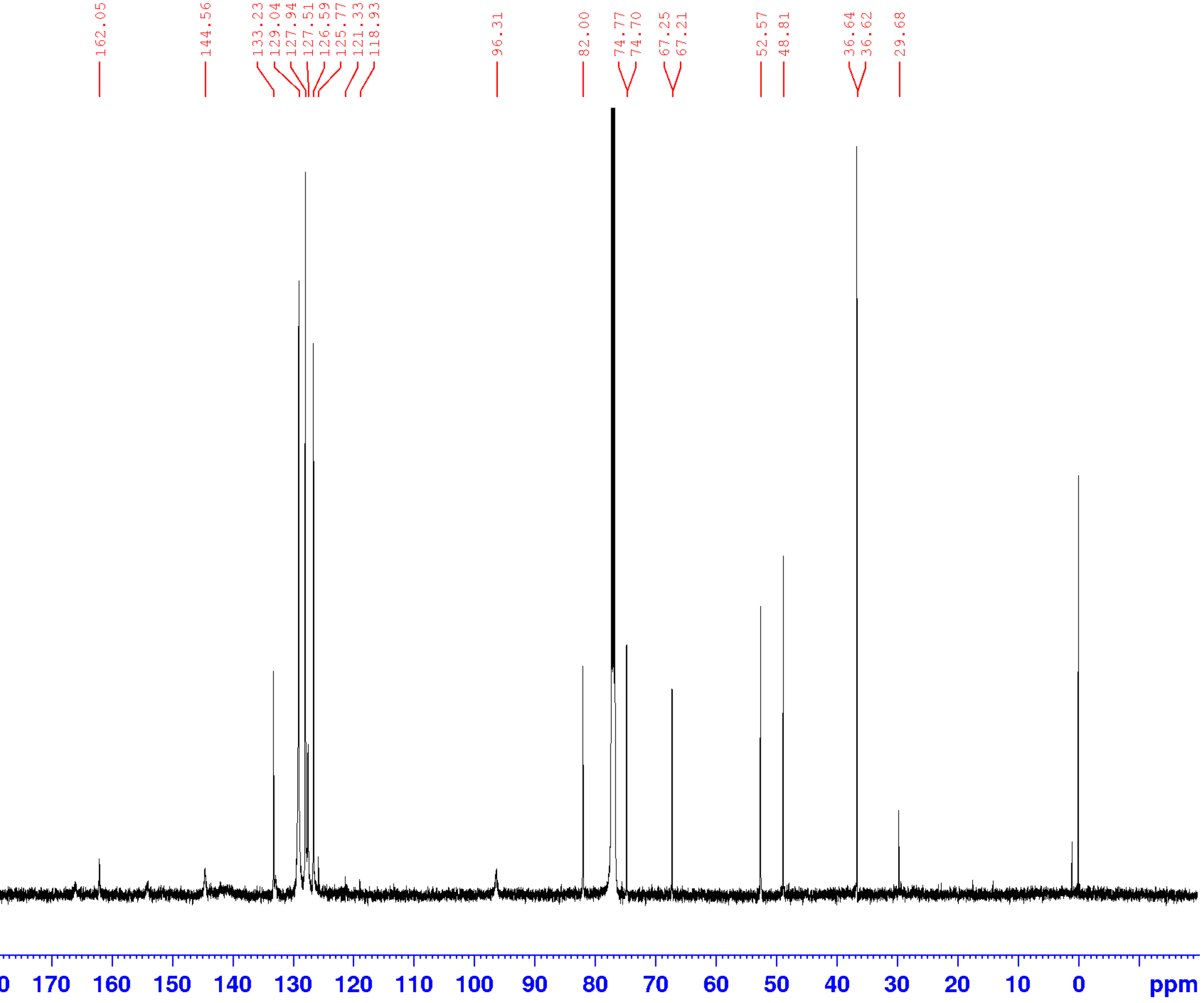

^{31}P {^{1}H} NMR (202 MHz, $CDCl_3$) of (*R*p)-**18**

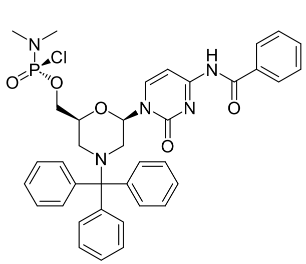

(*R*p)-**18**

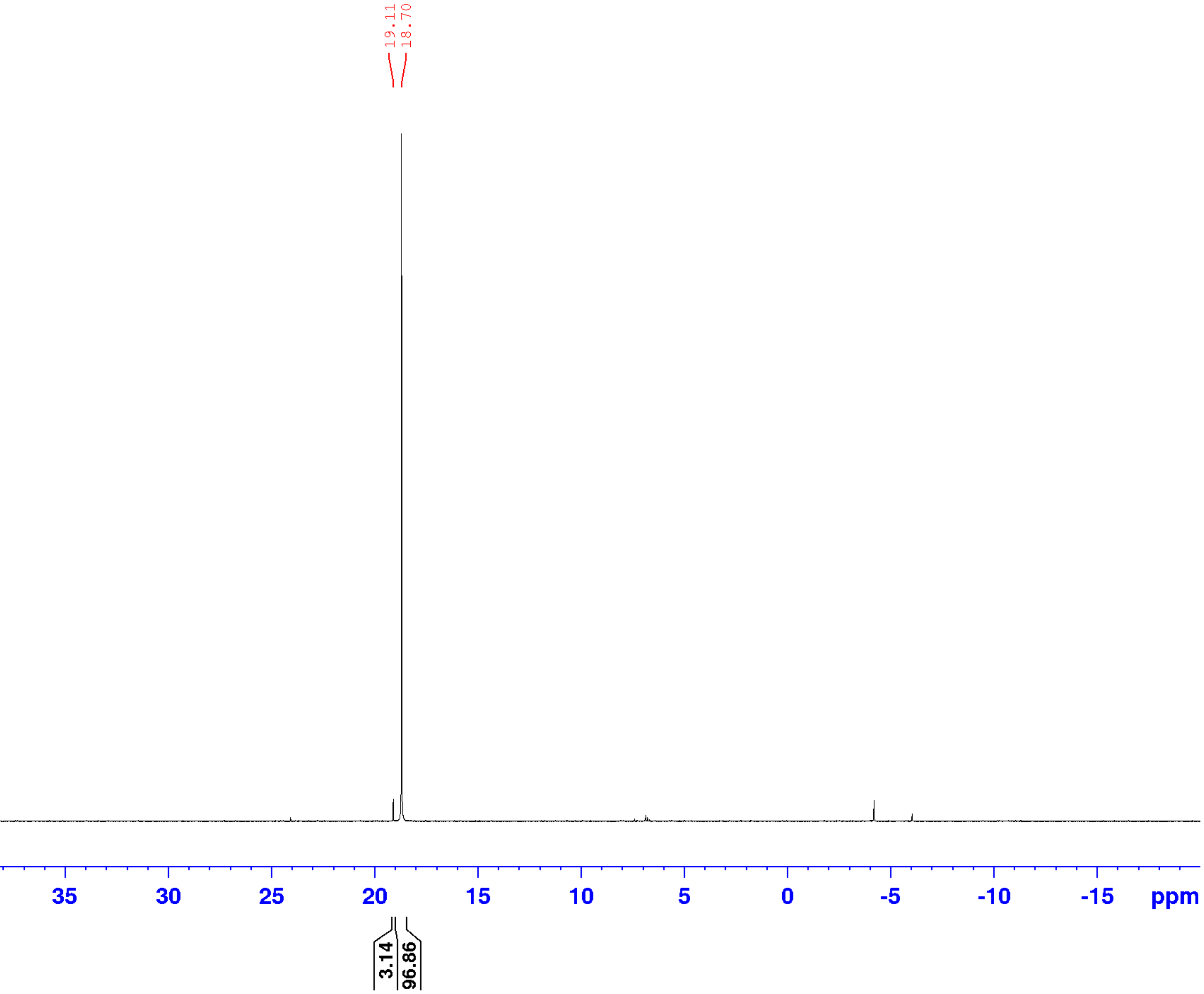

COSY (CDCl$_3$) of (*R*p)-**18**

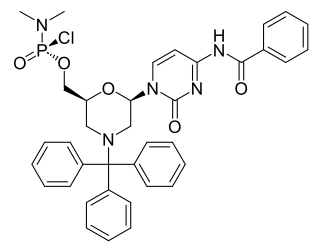

(*R*p)-**18**

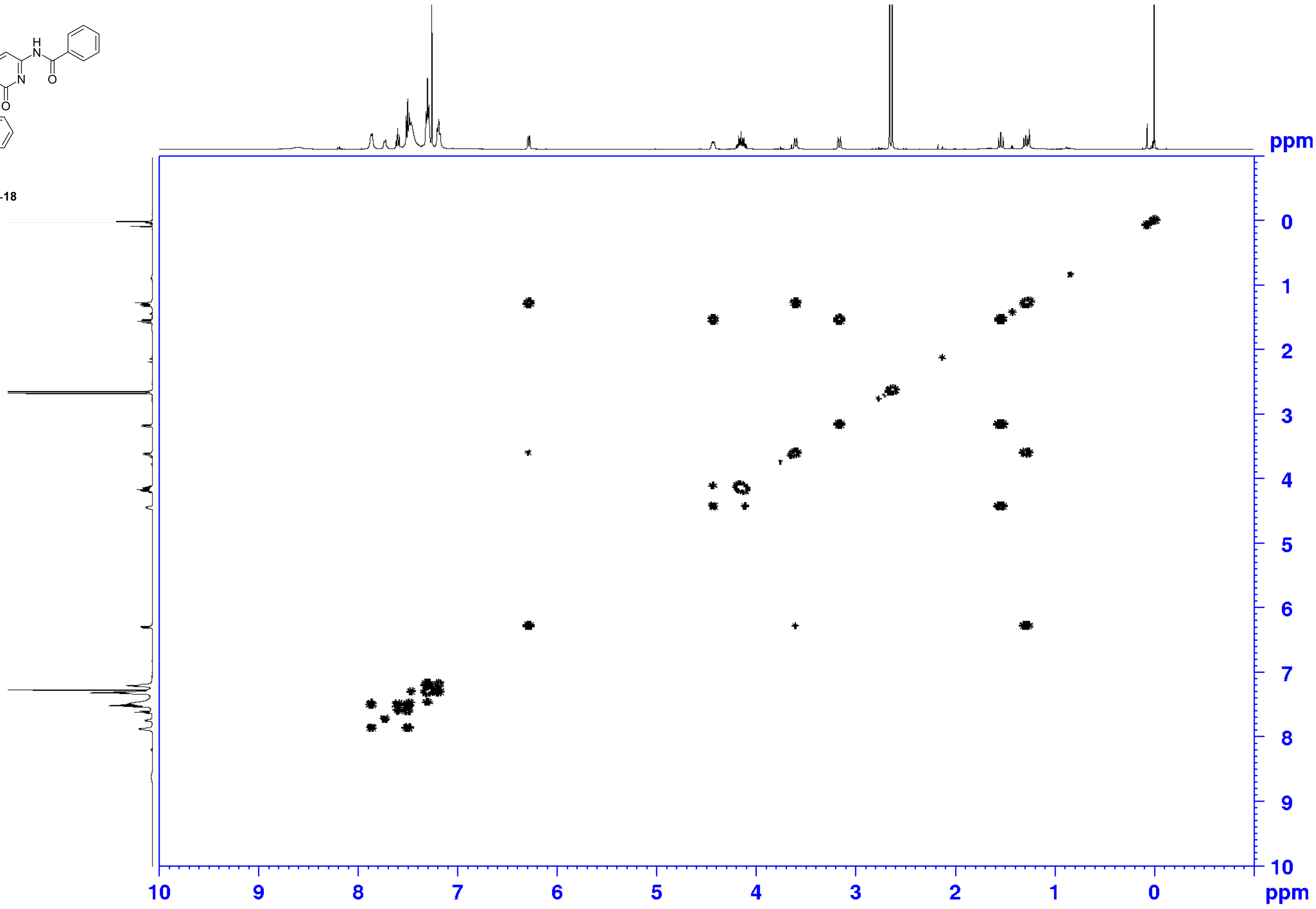

HSQC ($CDCl_3$) of (*R*p)-**18**

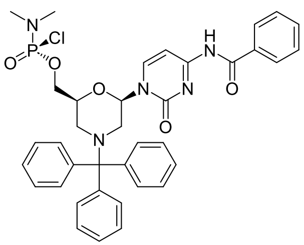

(*R*p)-**18**

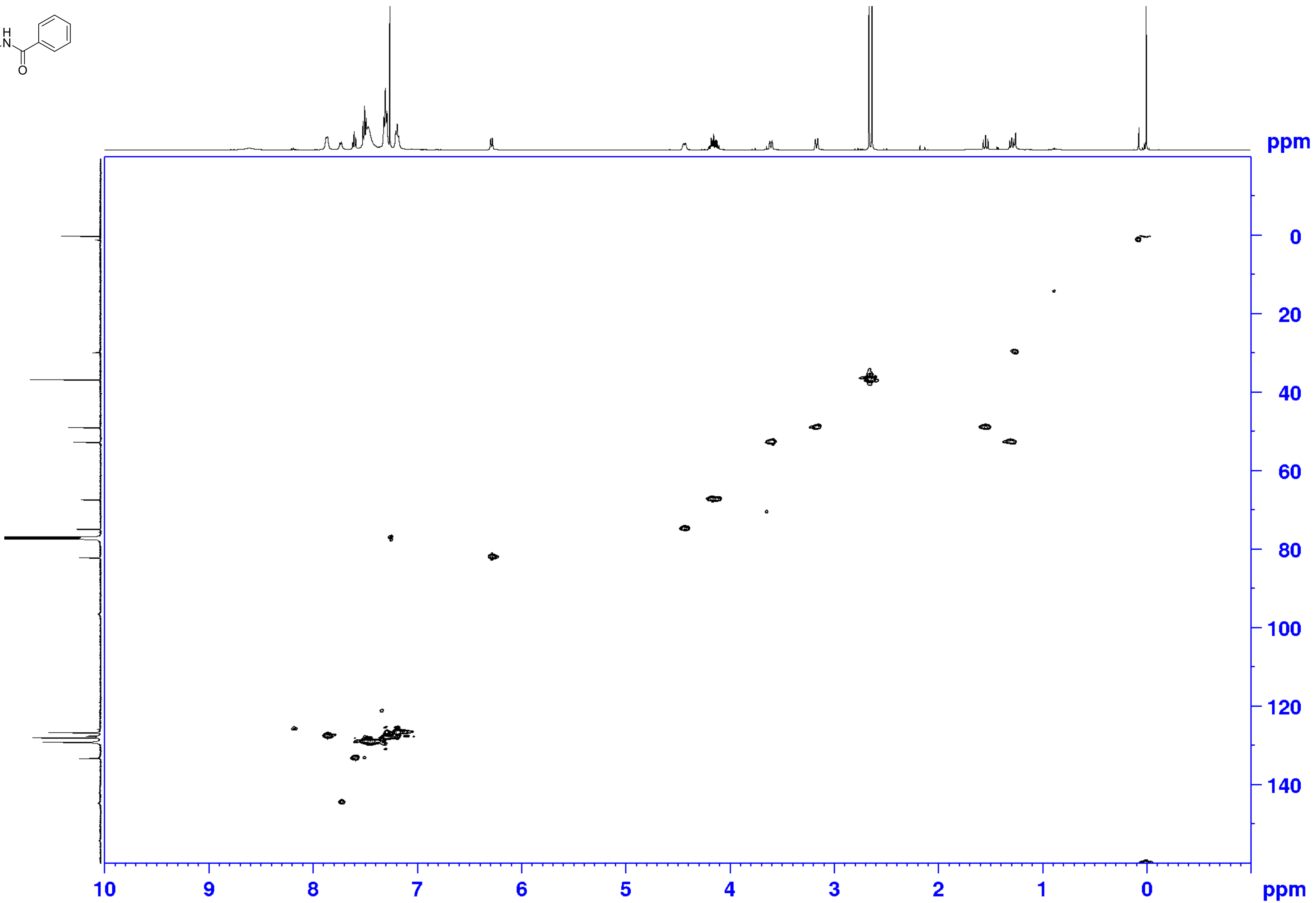

HMBC ($CDCl_3$) of (*R*p)-**18**

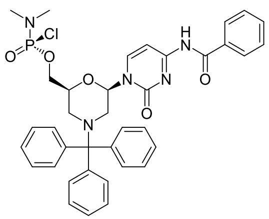

(*R*p)-**18**

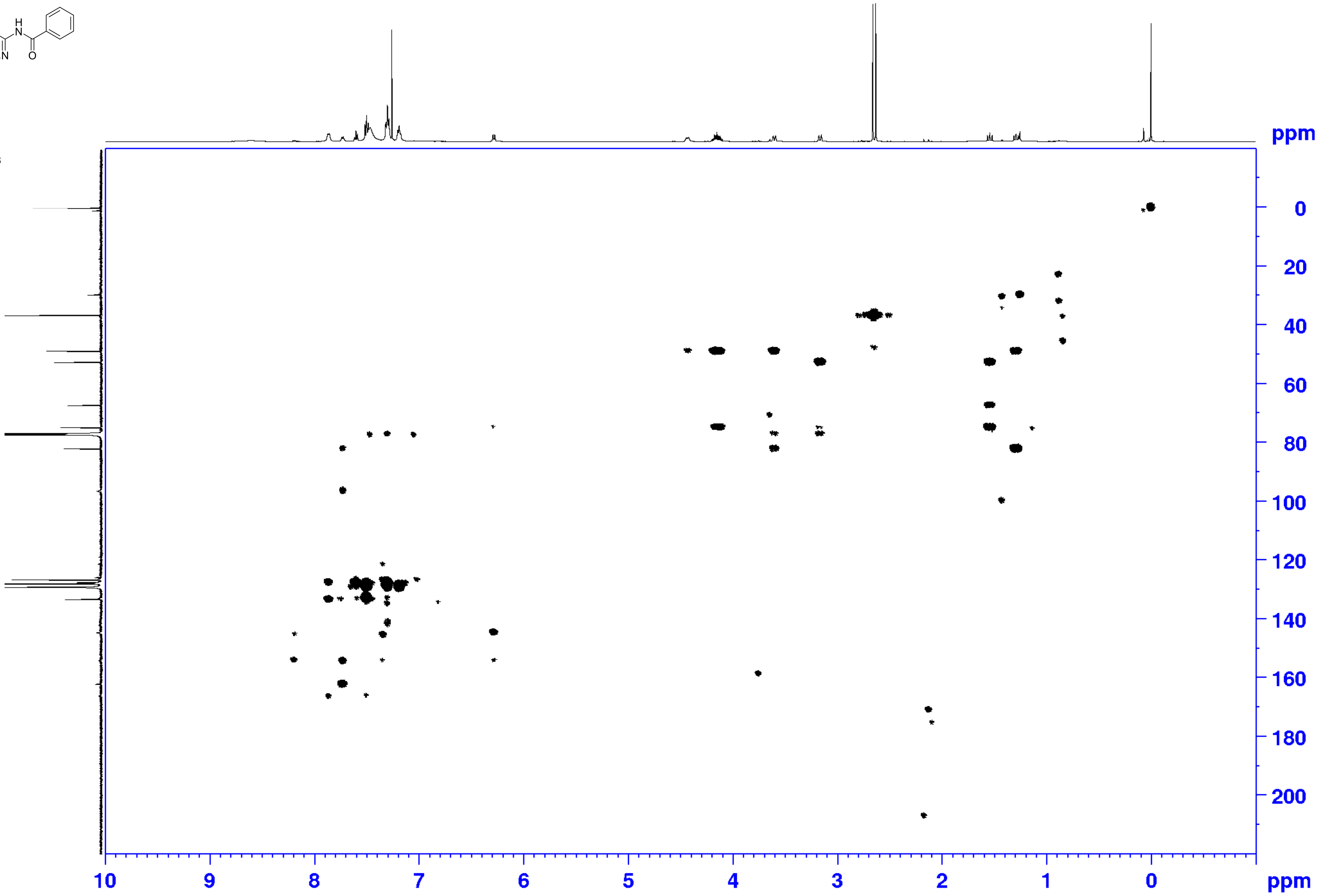

^{1}H NMR (400 MHz, $CDCl_3$) of **19**

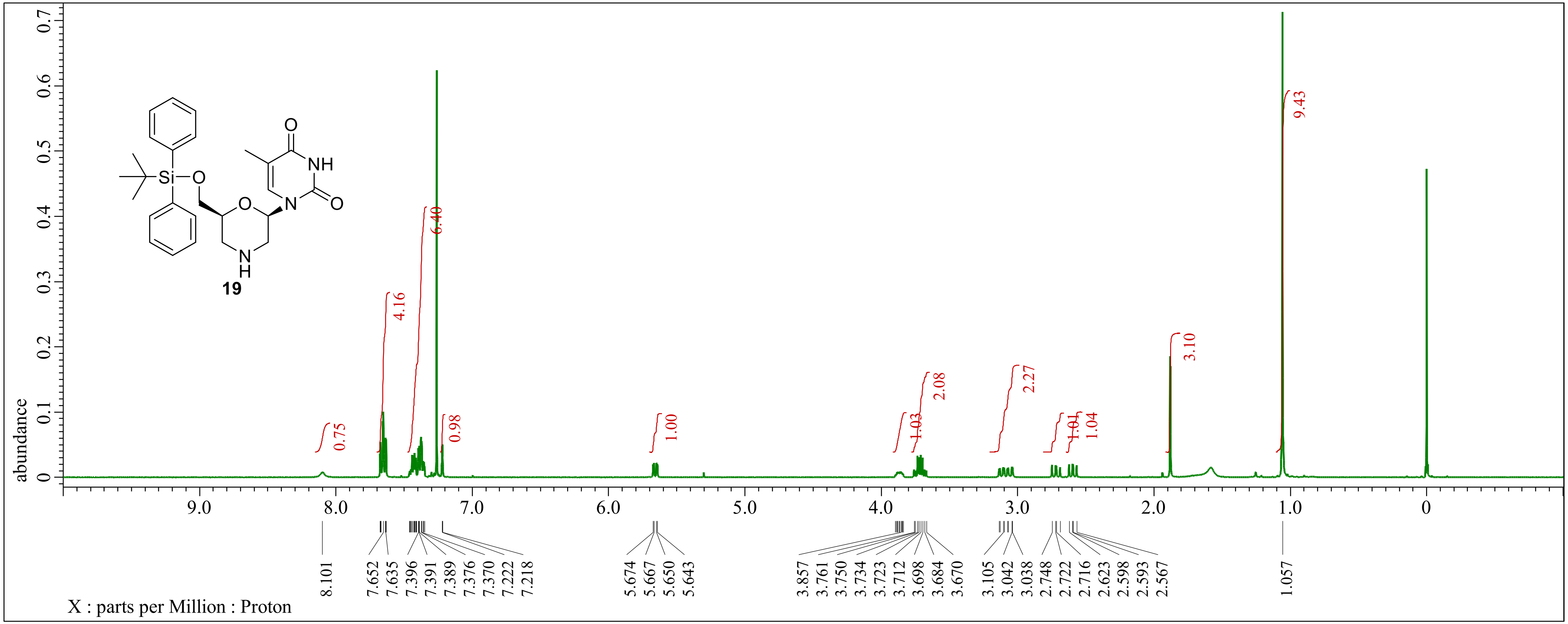

^{1}H NMR (400 MHz, $CDCl_3$) of **20**

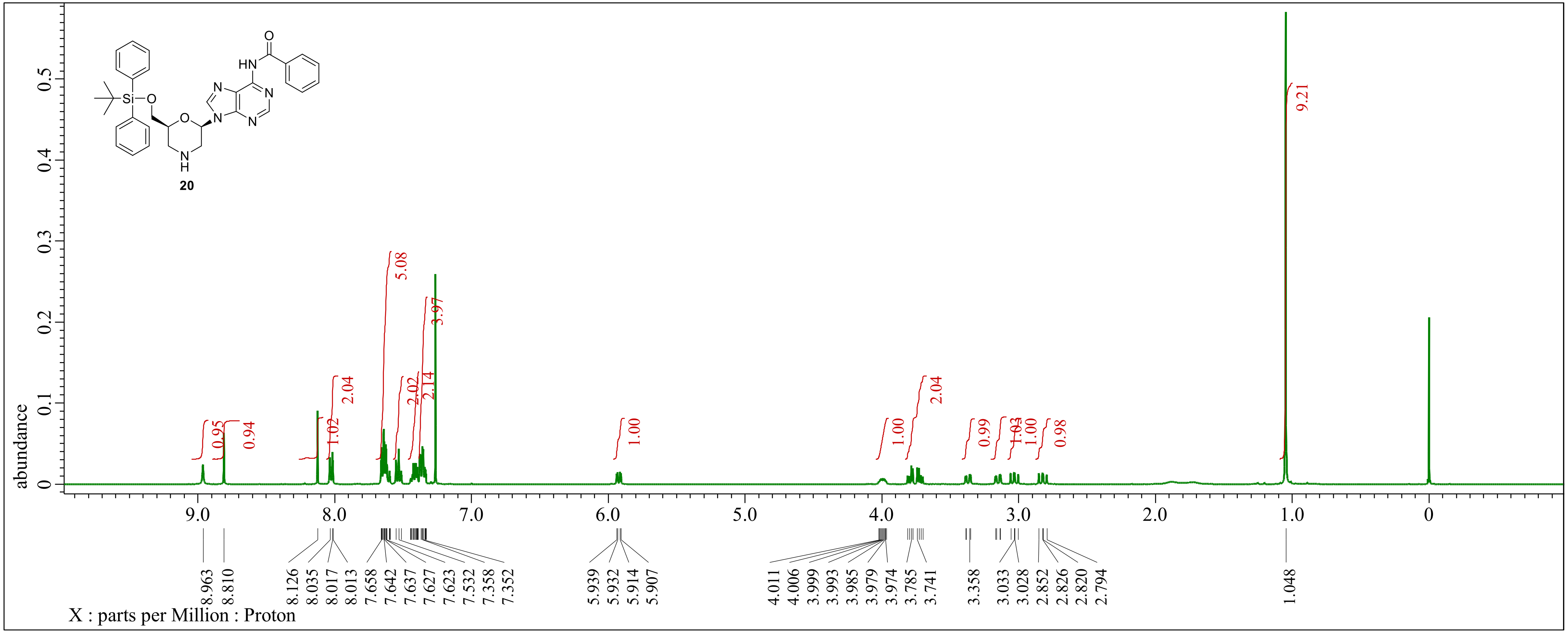

^{1}H NMR (400 MHz, $CDCl_3$) of **21**

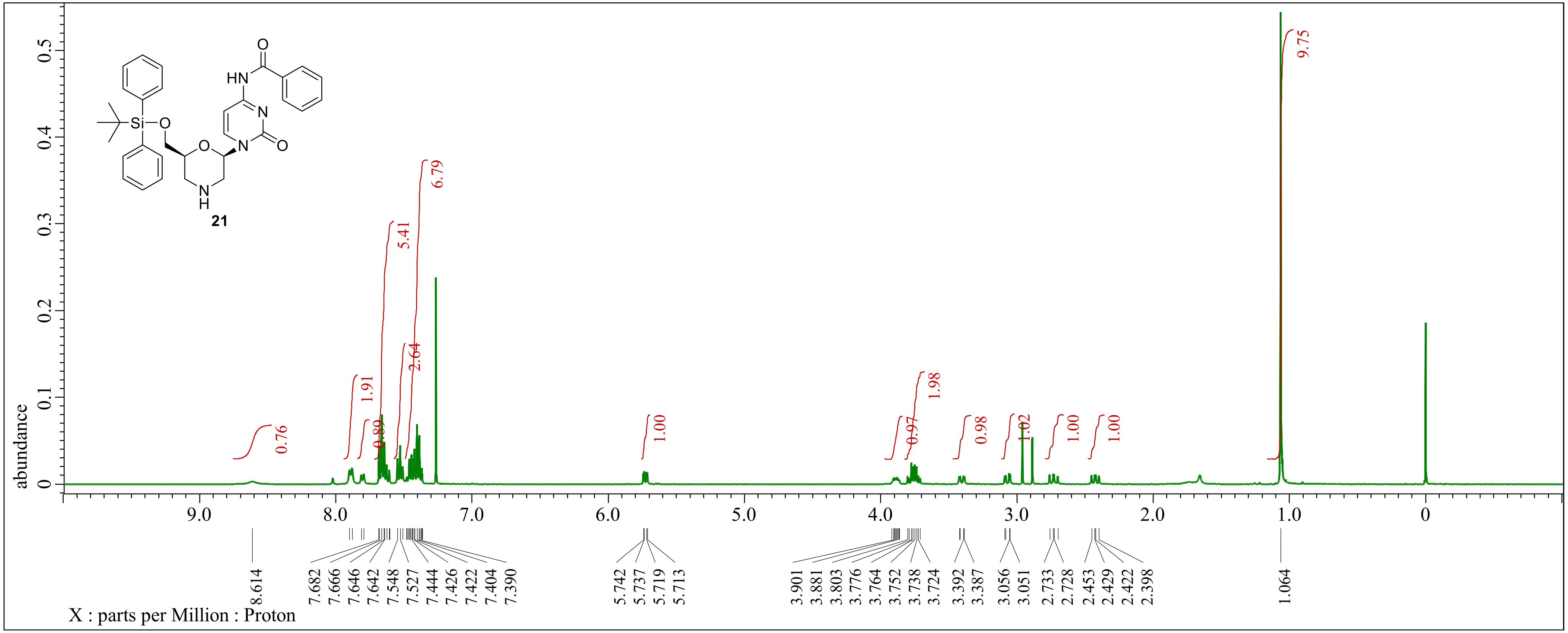

^{1}H NMR (400 MHz, $CDCl_3$) of **22**

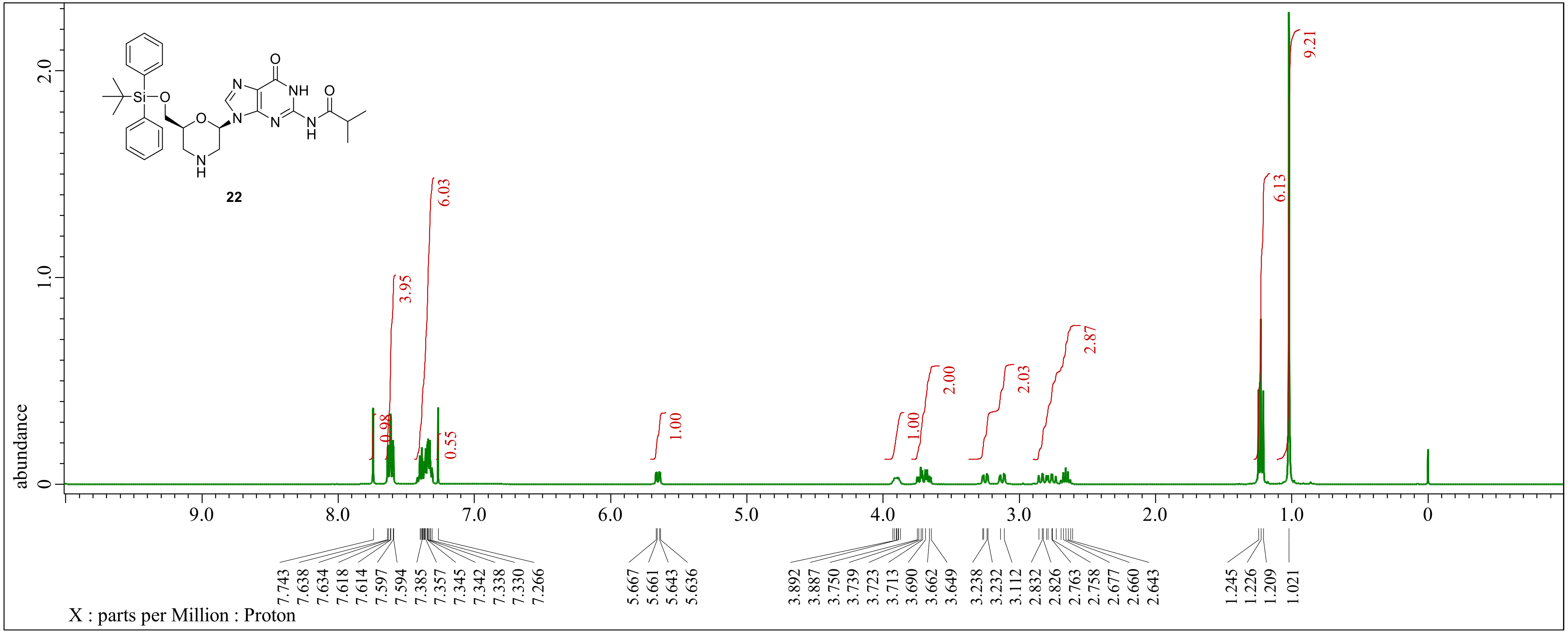

^{13}C {^{1}H} NMR (101 MHz, $CDCl_3$) of **22**

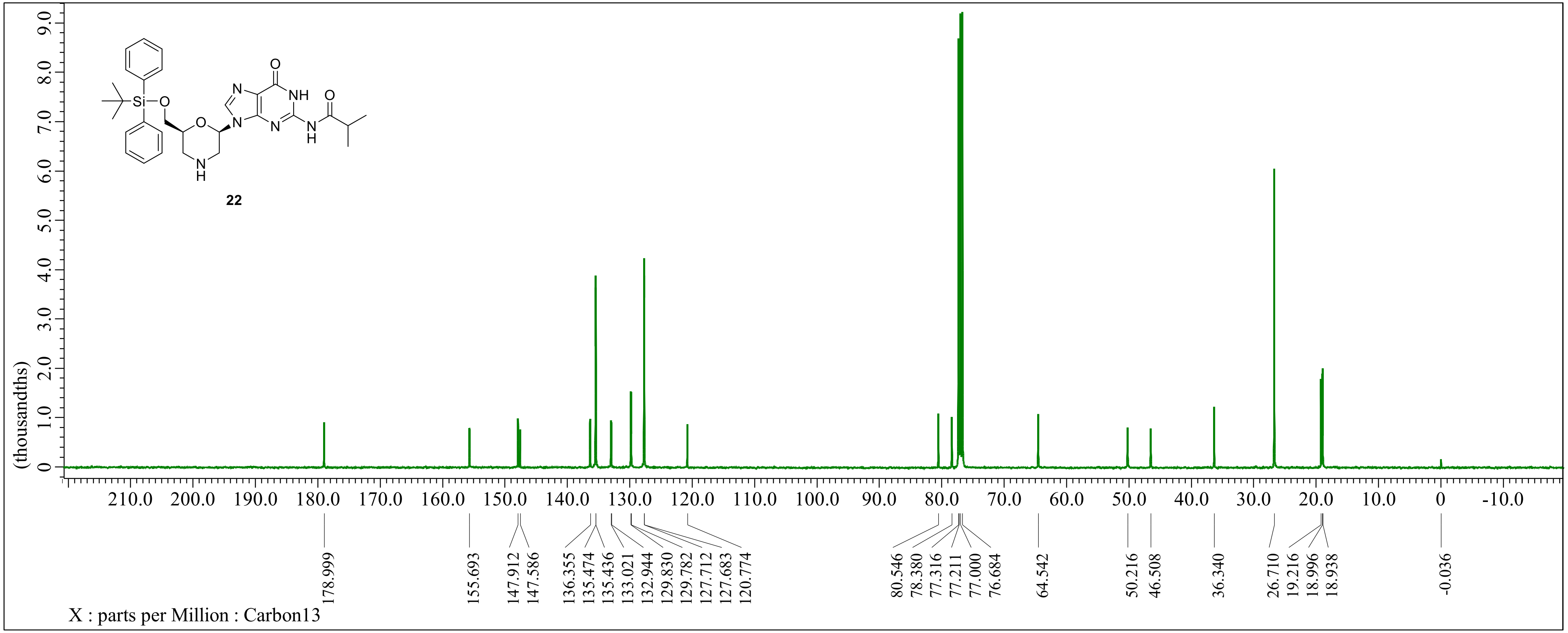

COSY of **22**

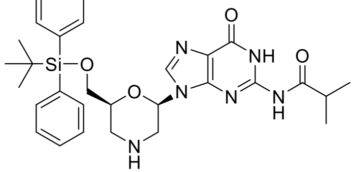

22

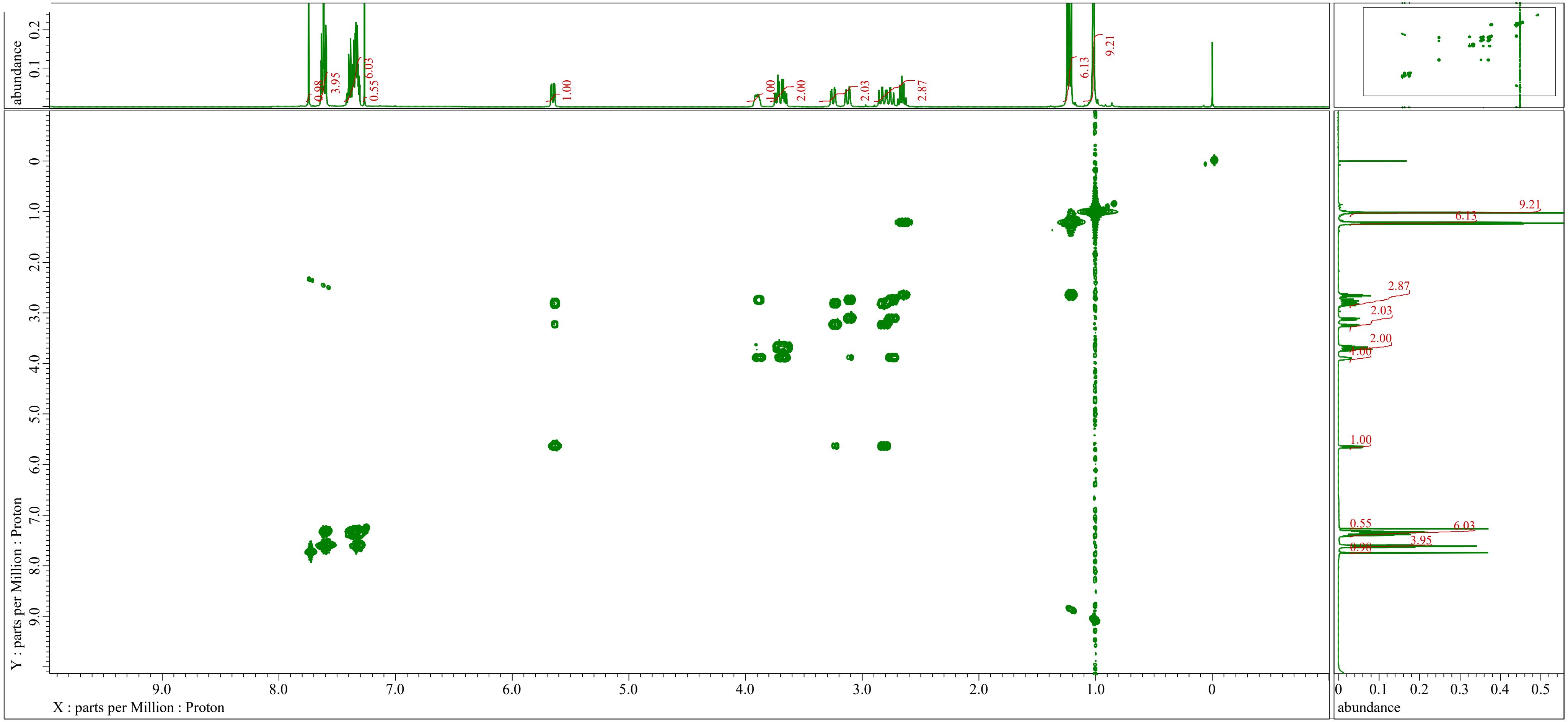

abundance
0.1
0.2
0.98
3.95
6.03
0.55
1.00
1.00
2.00
2.03
2.87
6.13
9.21
Y : parts per Million : Proton
X : parts per Million : Proton
9.0
8.0
7.0
6.0
5.0
4.0
3.0
2.0
1.0
0
0.3
0.4
0.5

HSQC of **22**

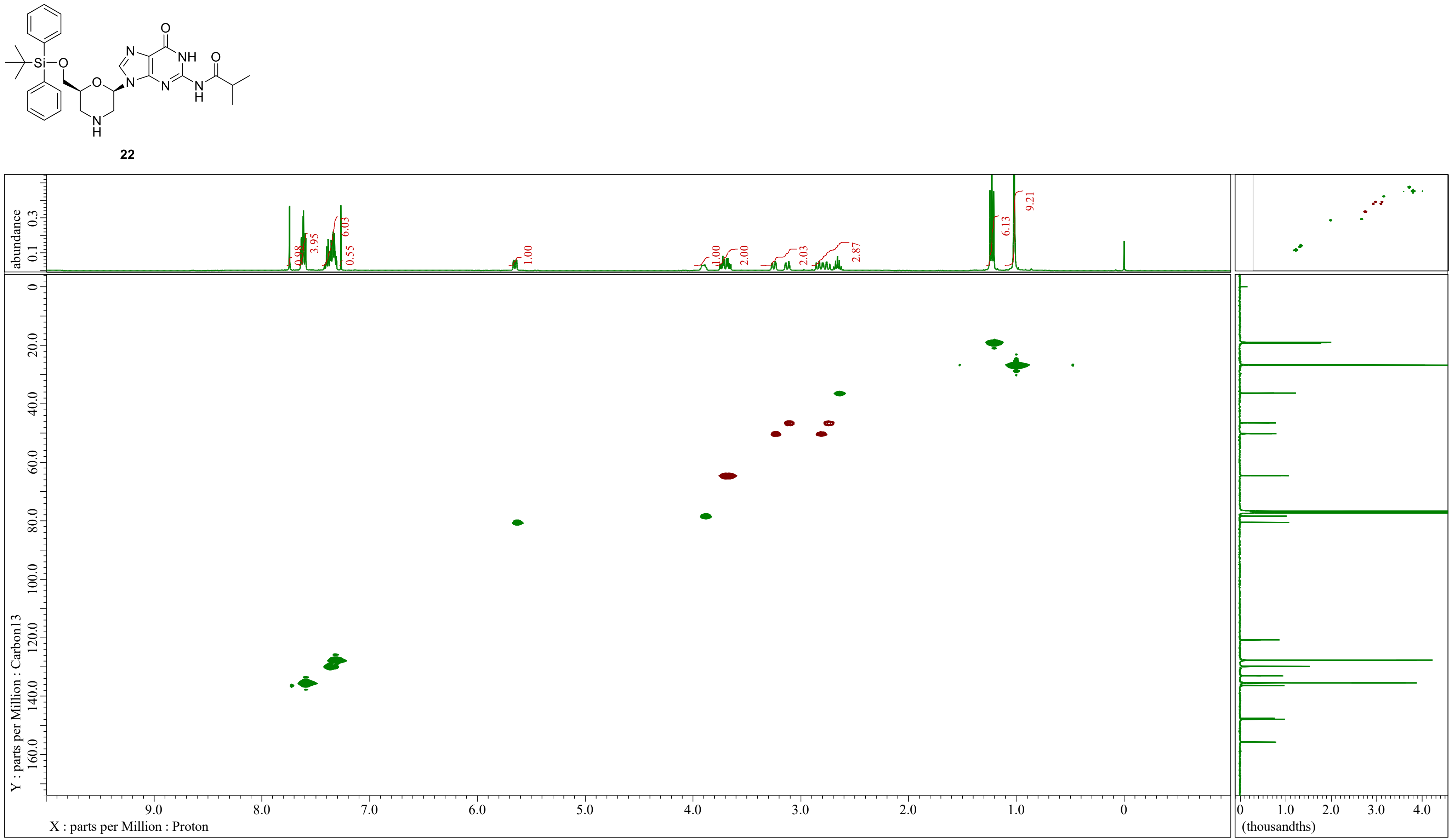

Si
O
N
NH
O
N
N
N
H
22
abundance
0.1
0.3
0.98
3.95
6.03
0.55
1.00
1.00
2.00
2.03
2.87
6.13
9.21
Y : parts per Million : Carbon13
0
20.0
40.0
60.0
80.0
100.0
120.0
140.0
160.0
9.0
8.0
7.0
6.0
5.0
4.0
3.0
2.0
1.0
0
X : parts per Million : Proton
0
1.0
2.0
3.0
4.0
(thousandths)

HMBC of **22**

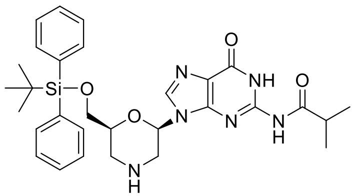

22

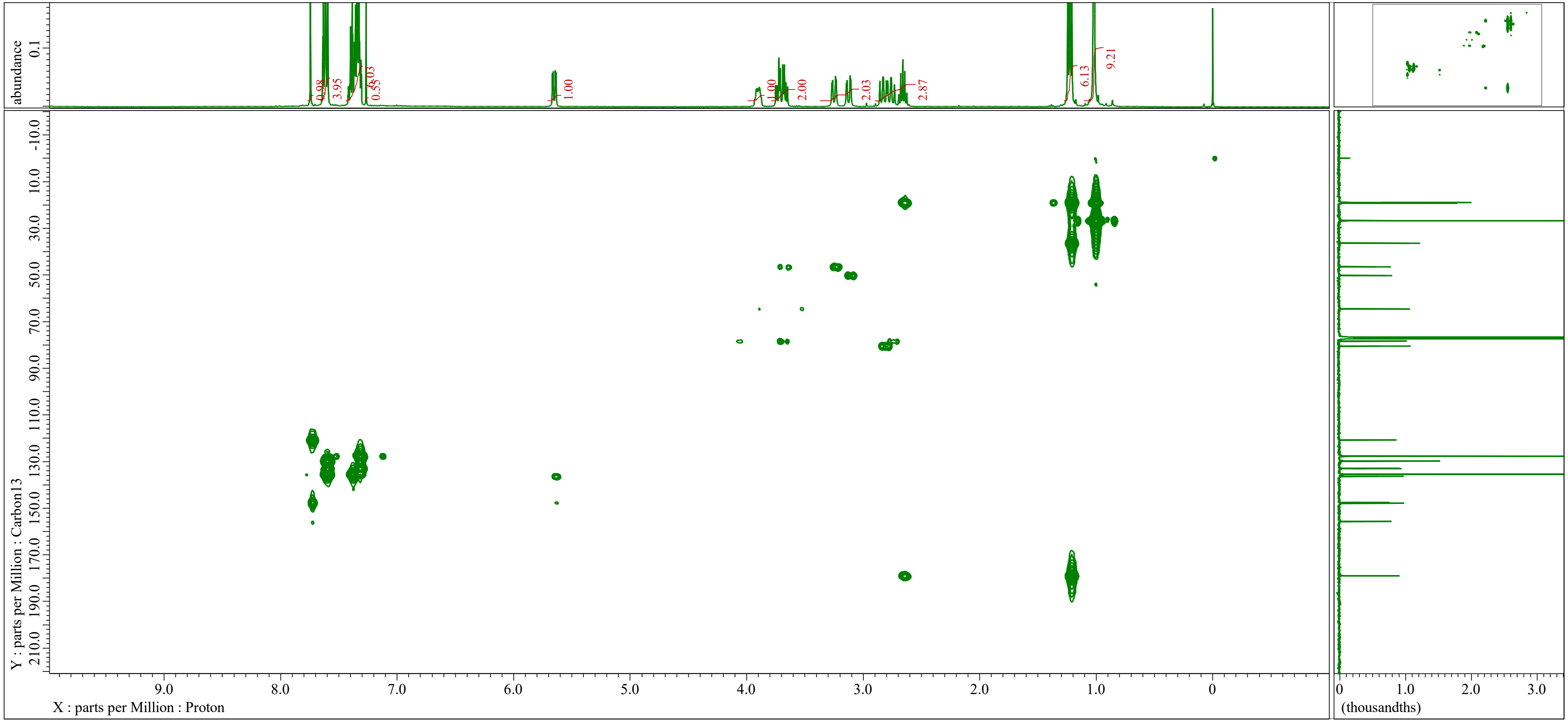

abundance
0.1
0.98
3.95
0.55
1.03
1.00
1.00
2.00
2.03
2.87
6.13
9.21
Y : parts per Million : Carbon13
-10.0
10.0
30.0
50.0
70.0
90.0
110.0
130.0
150.0
170.0
190.0
210.0
9.0
8.0
7.0
6.0
5.0
4.0
3.0
2.0
1.0
0
X : parts per Million : Proton
0
1.0
2.0
3.0
(thousandths)

^{1}H NMR (500 MHz, $CDCl_3$) of (*S*p)-**23**

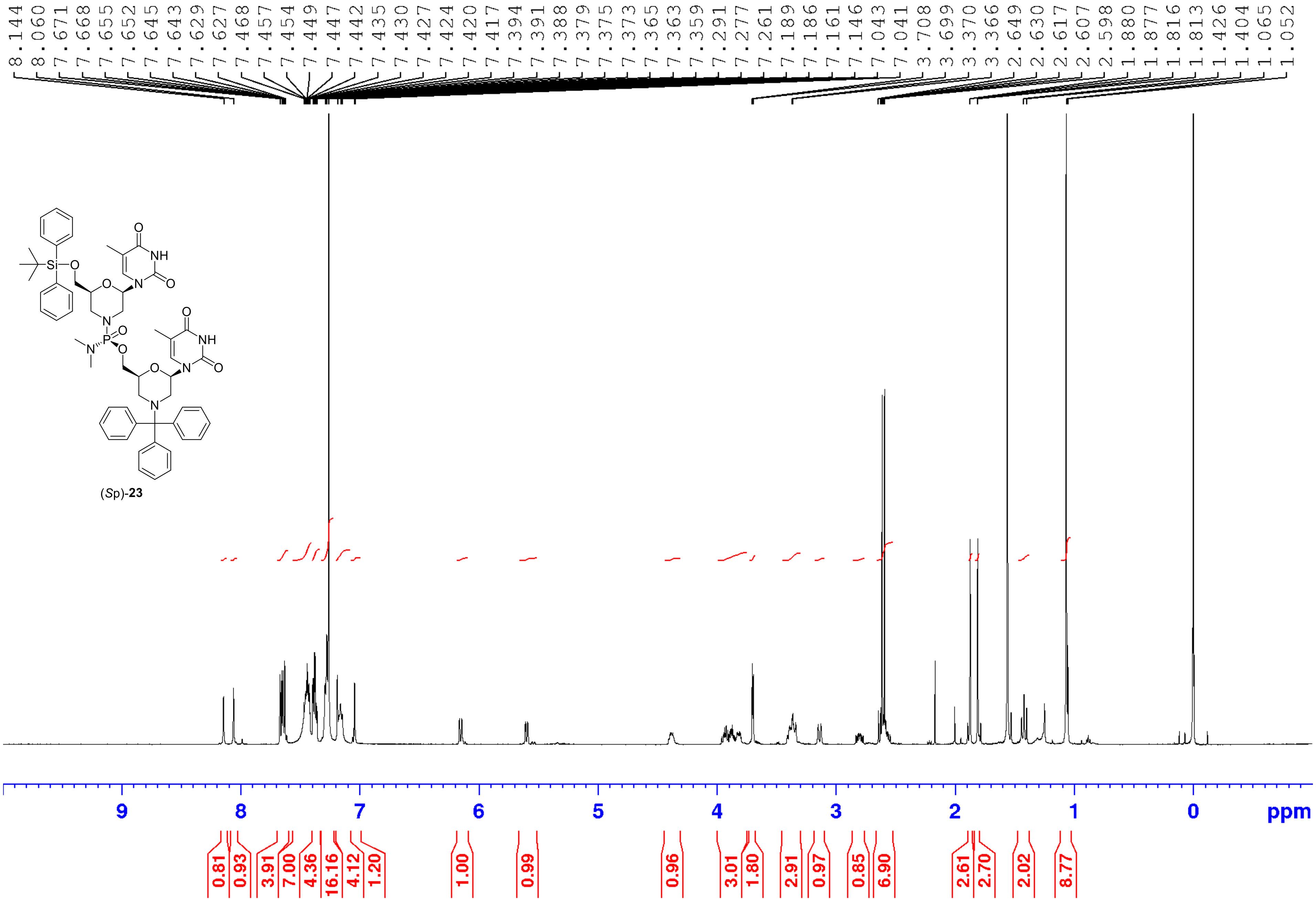

$^{13}C\ \{^{1}H\}$ NMR (126 MHz, $CDCl_3$) of (*S*p)-**23**

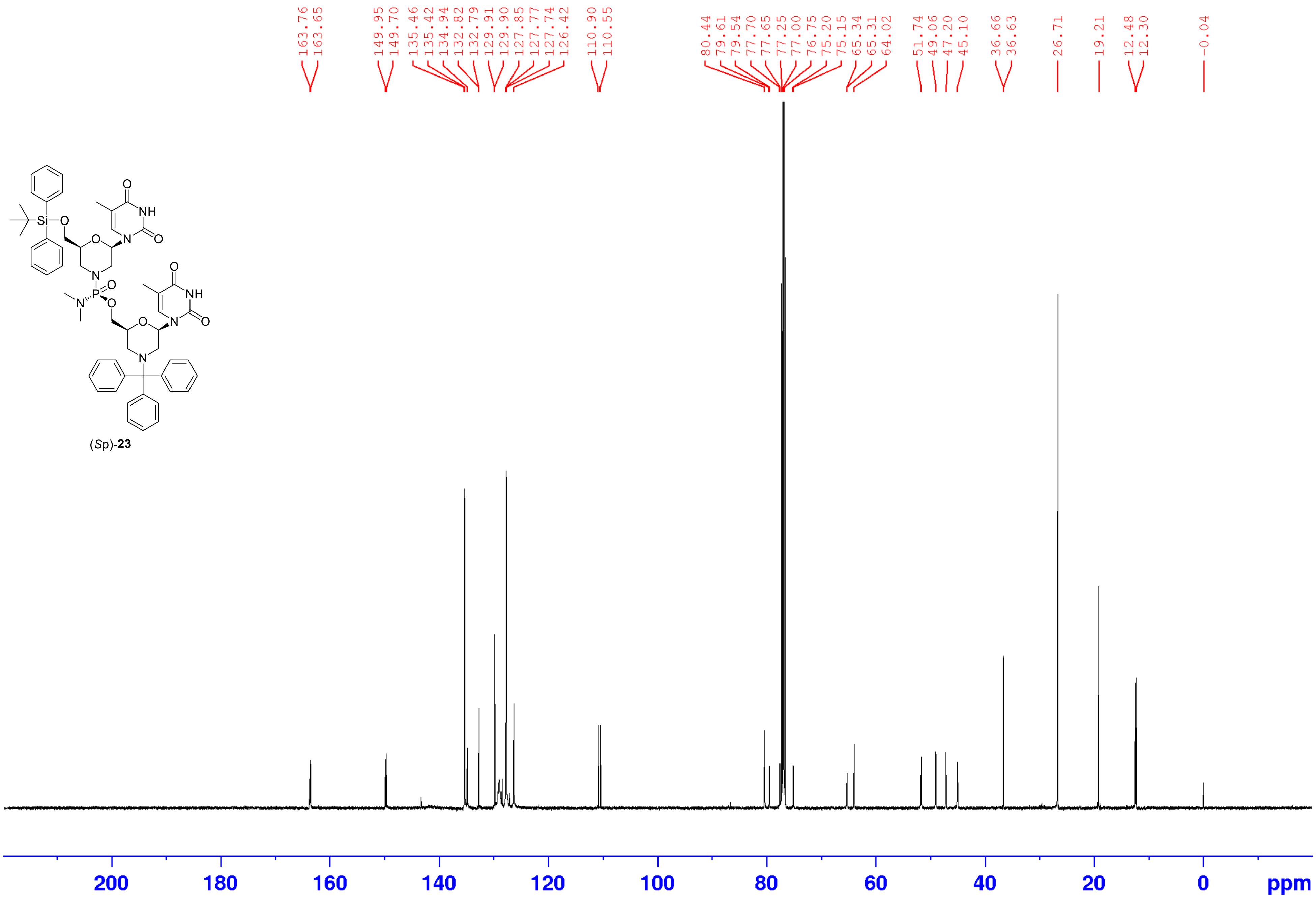

$^{31}P\ \{^{1}H\}$ NMR (202 MHz, $CDCl_3$) of (*S*p)-**23**

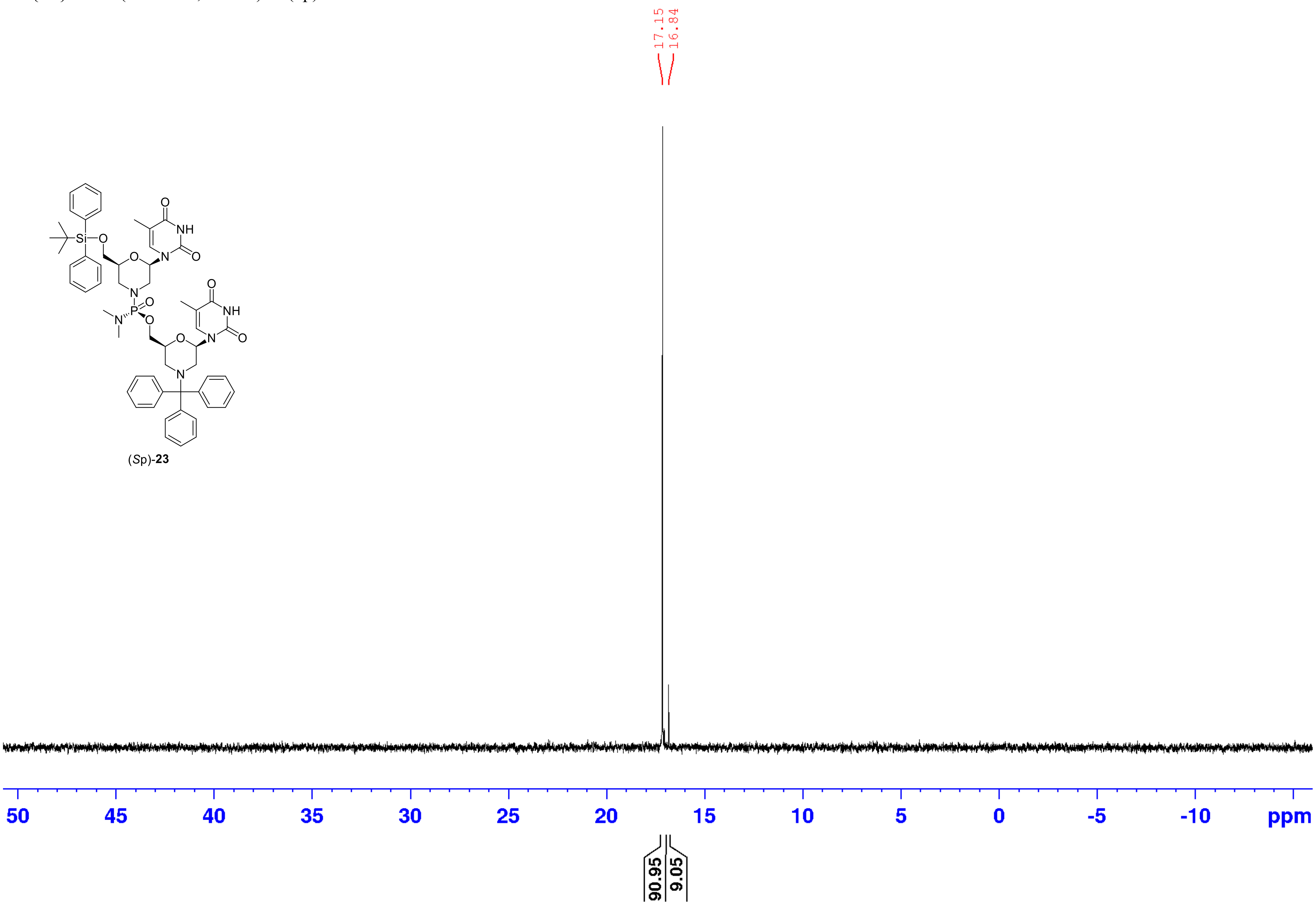

COSY ($CDCl_3$) of (*S*p)-**23**

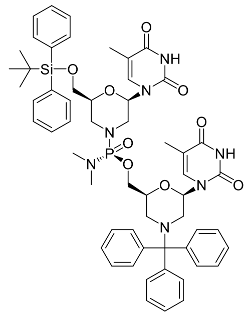

(*S*p)-**23**

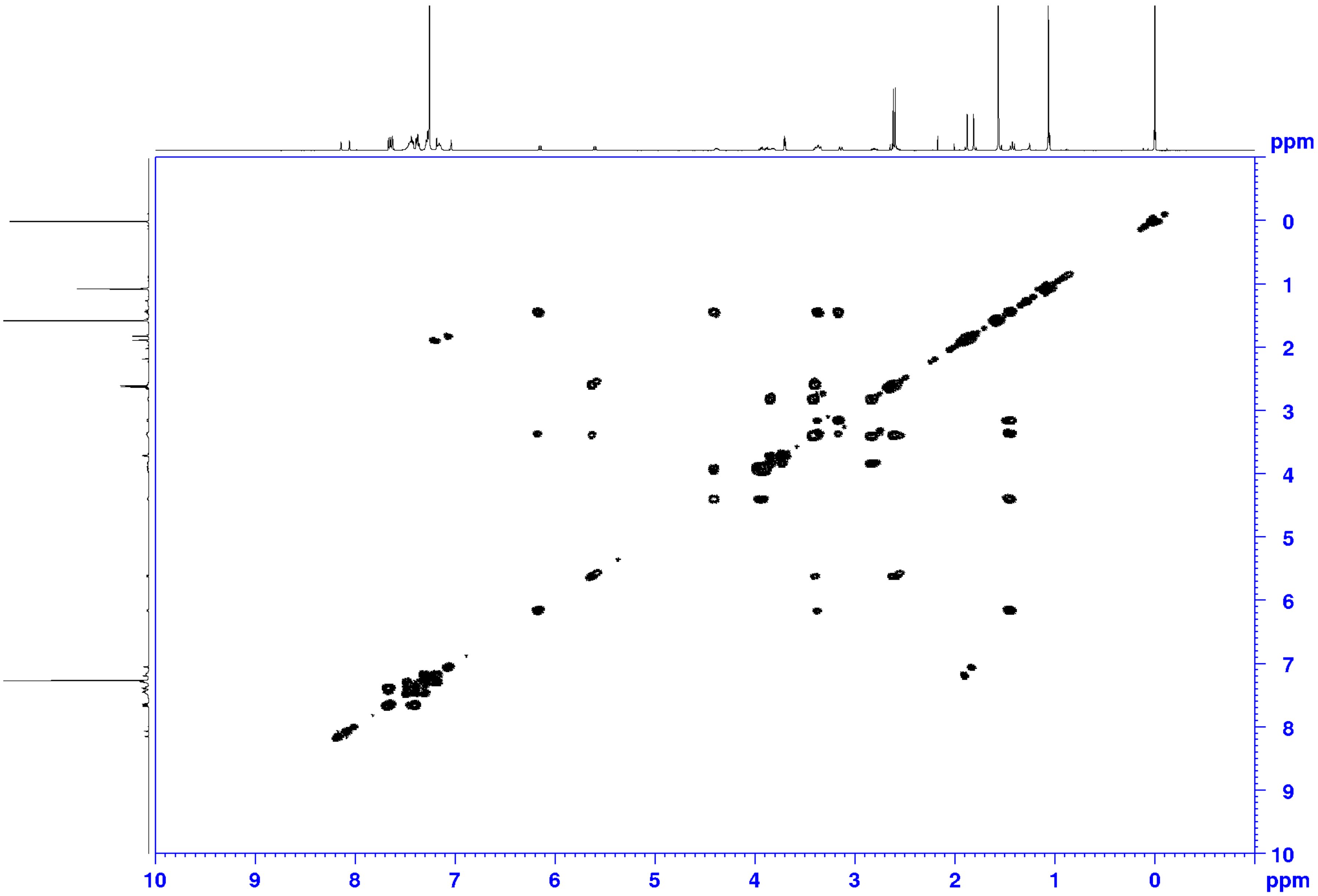

HSQC ($CDCl_3$) of (*S*p)-**23**

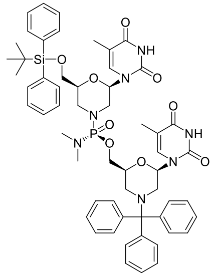

(*S*p)-**23**

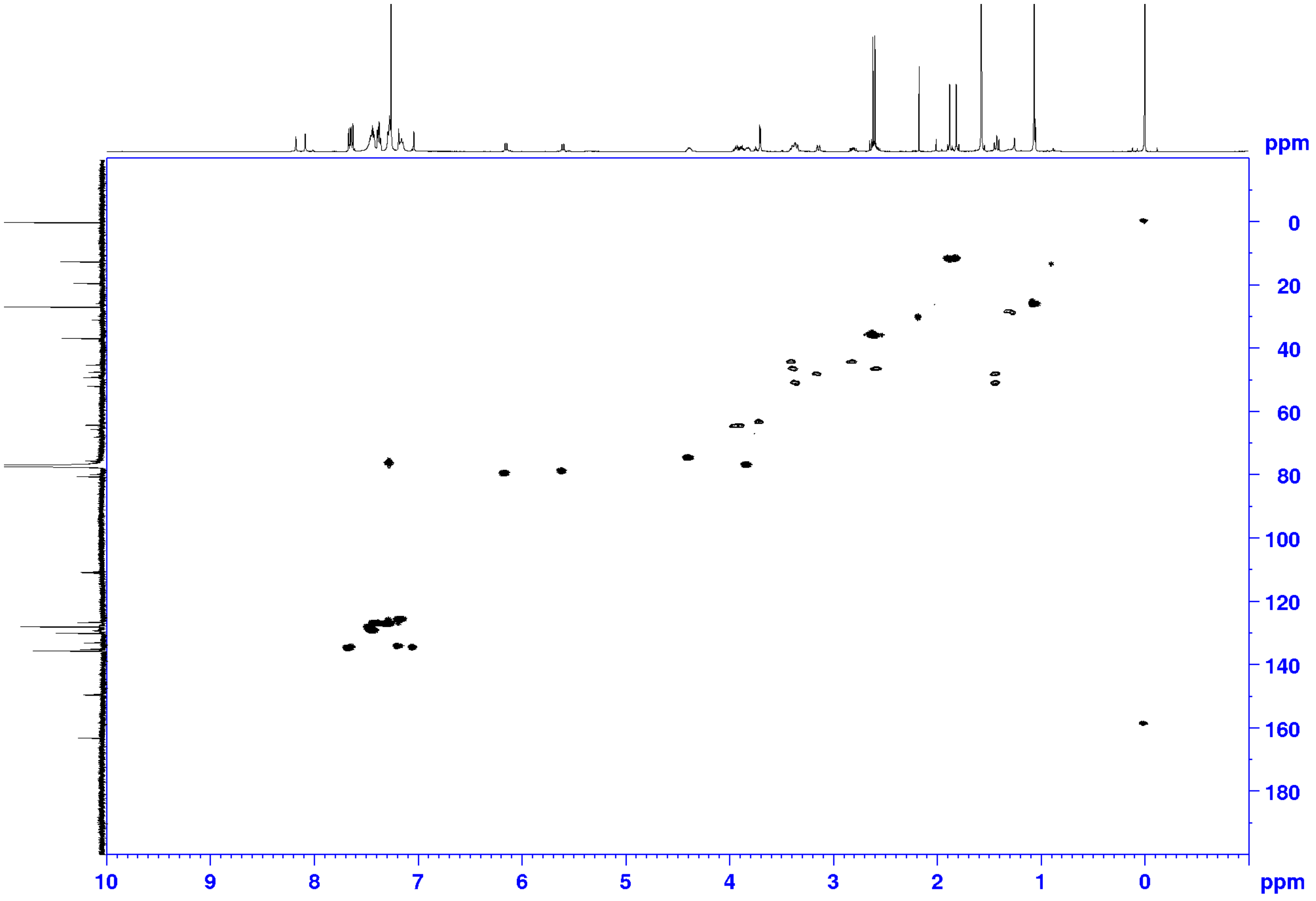

HMBC ($CDCl_3$) of (*S*p)-**23**

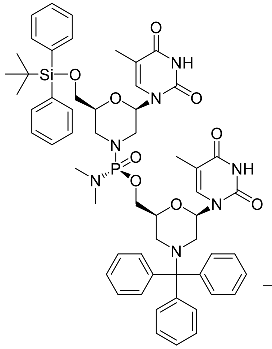

(*S*p)-**23**

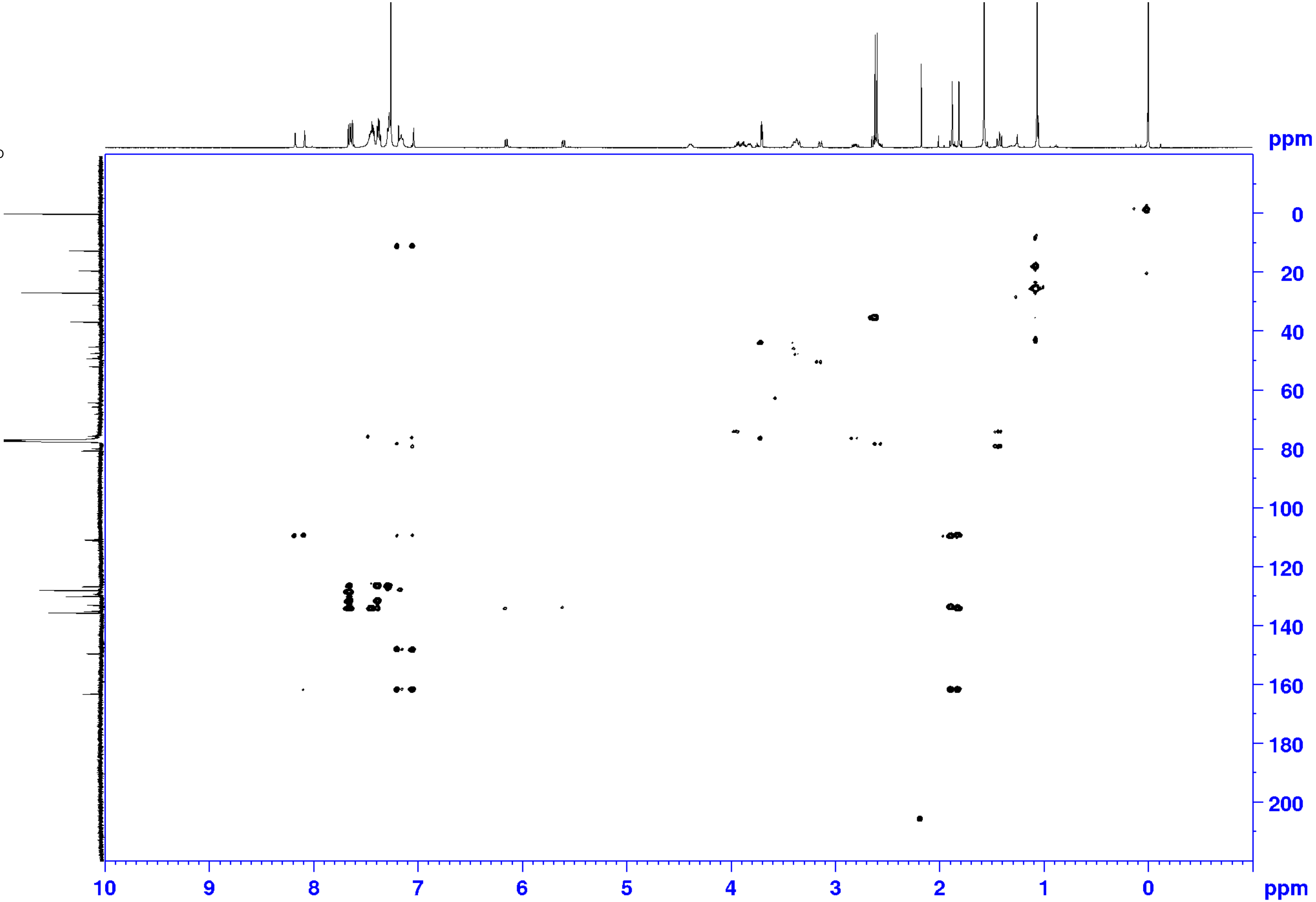

^{1}H NMR (500 MHz, $CDCl_3$) of (*R*p)-**23**

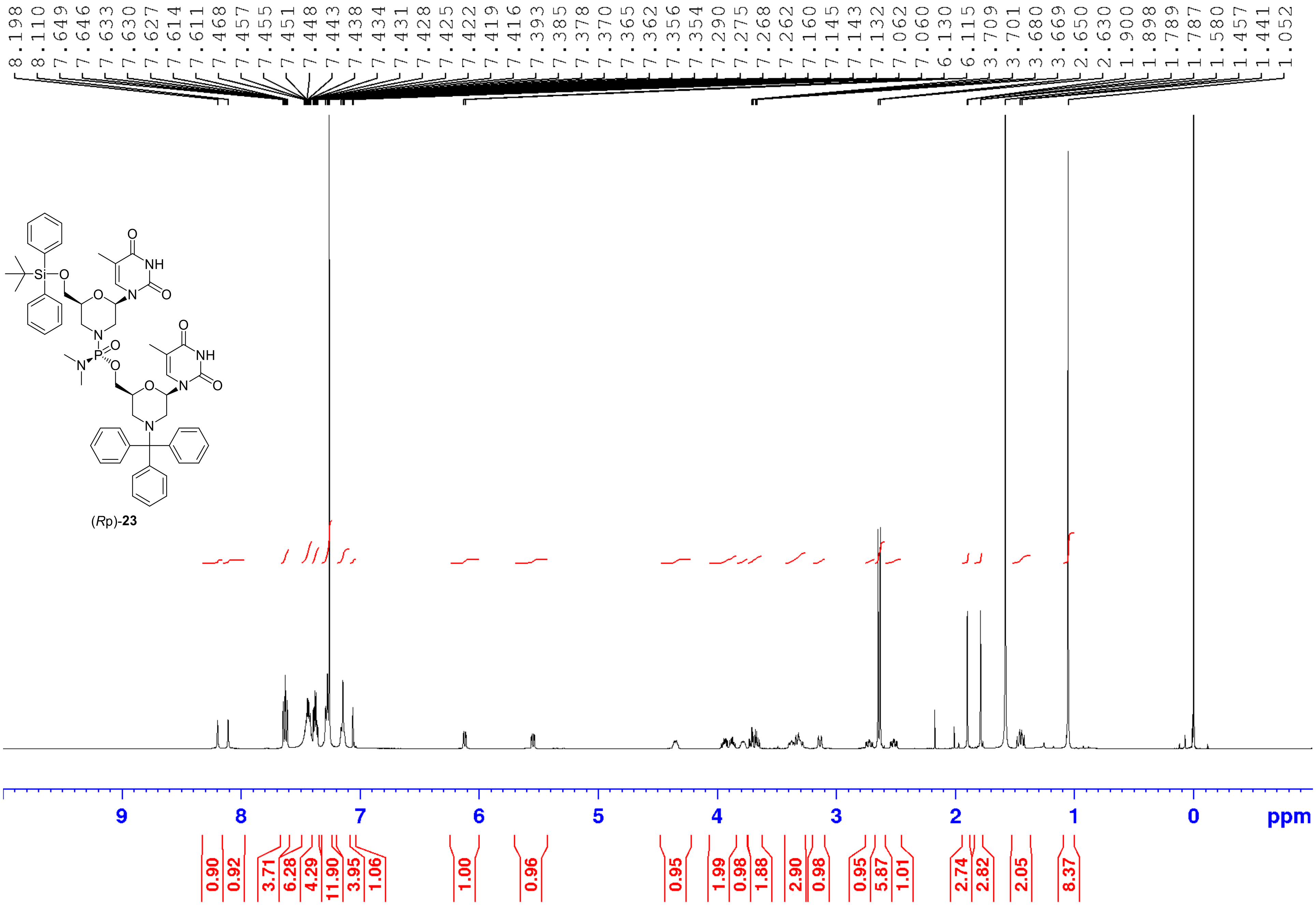

$^{13}C\{^{1}H\}$ NMR (126 MHz, CDCl$_3$) of (*R*p)-**23**

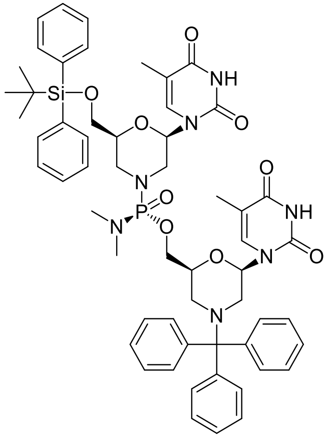

(*R*p)-**23**

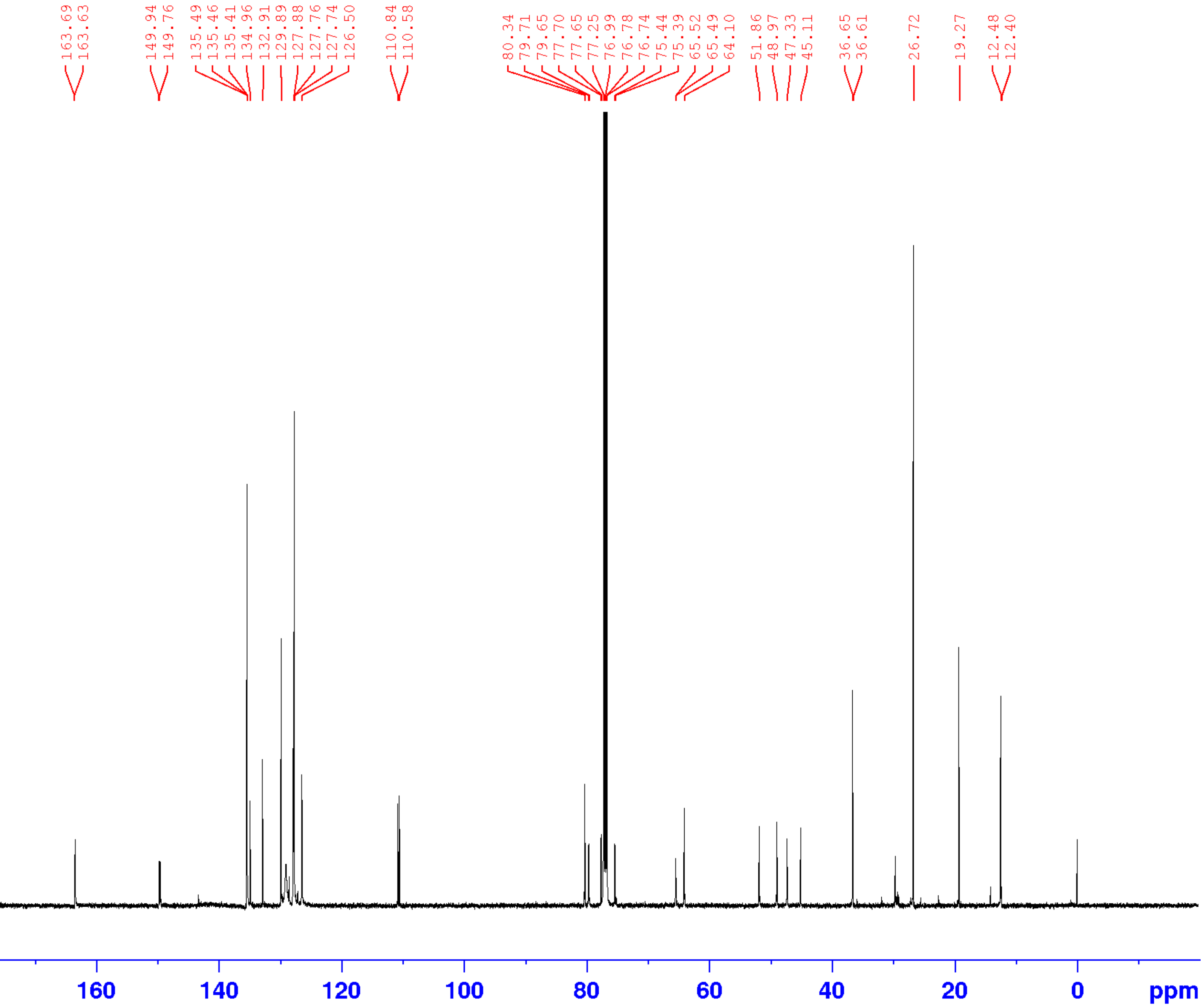

^{31}P {^{1}H} NMR (202 MHz, $CDCl_3$) of (*R*p)-**23**

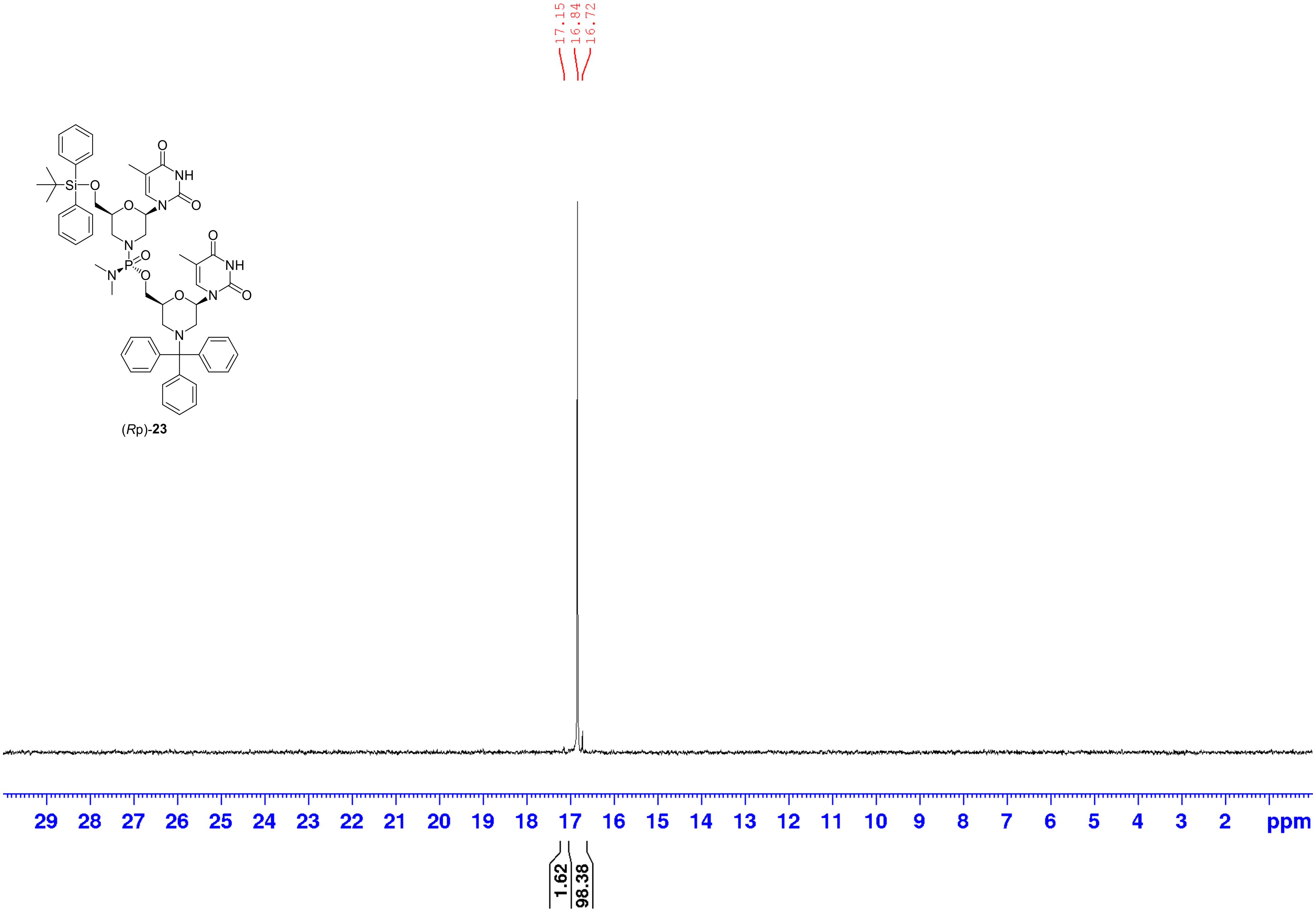

COSY ($CDCl_3$) of (*R*p)-**23**

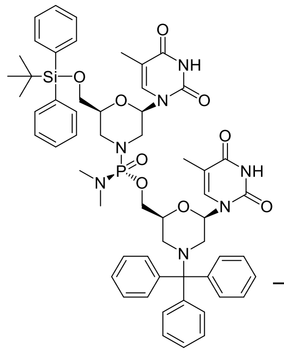

(*R*p)-**23**

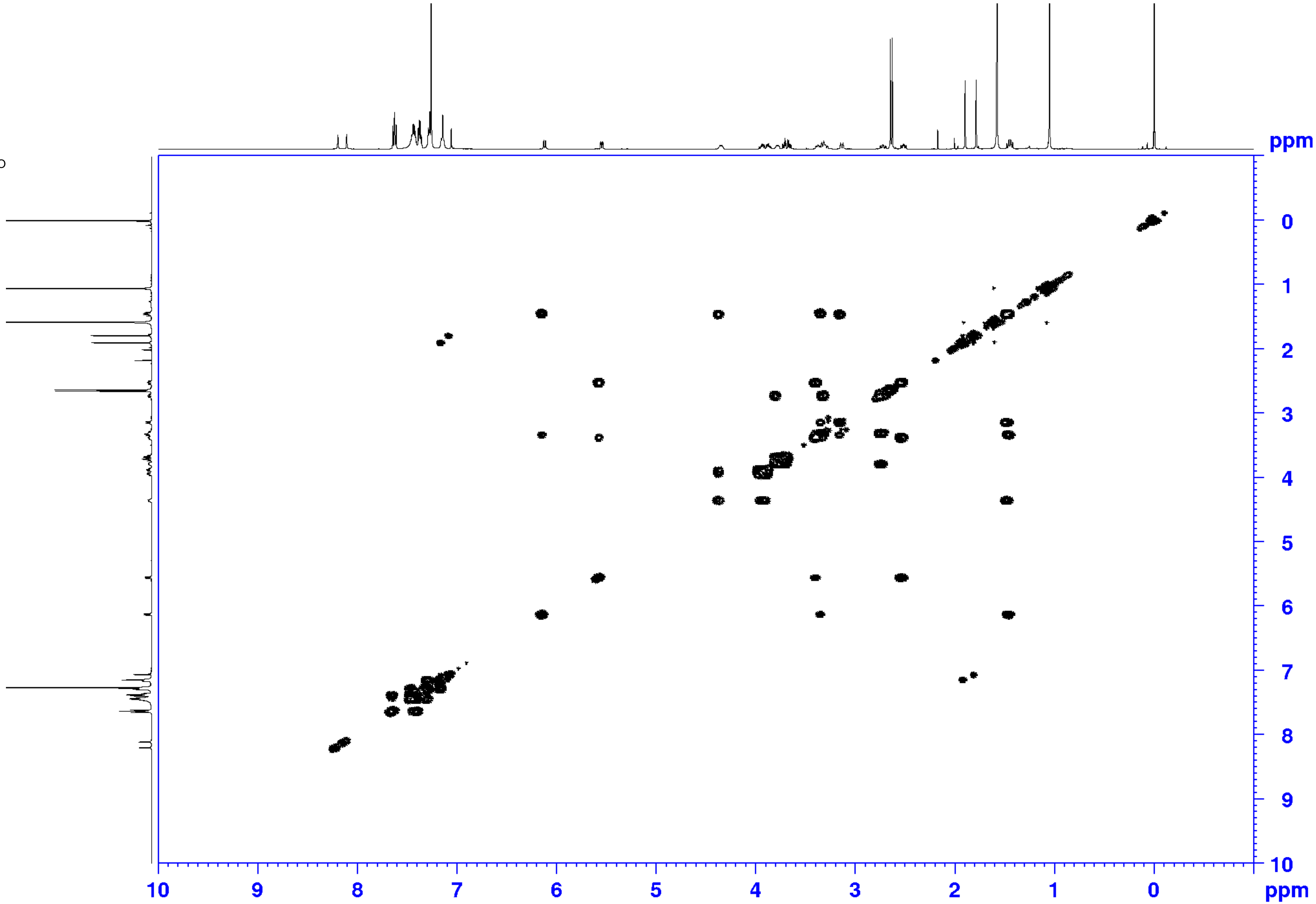

HSQC ($CDCl_3$) of (*R*p)-**23**

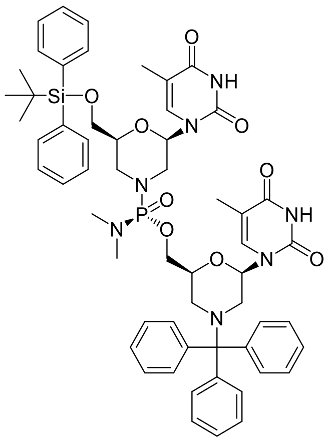

(*R*p)-**23**

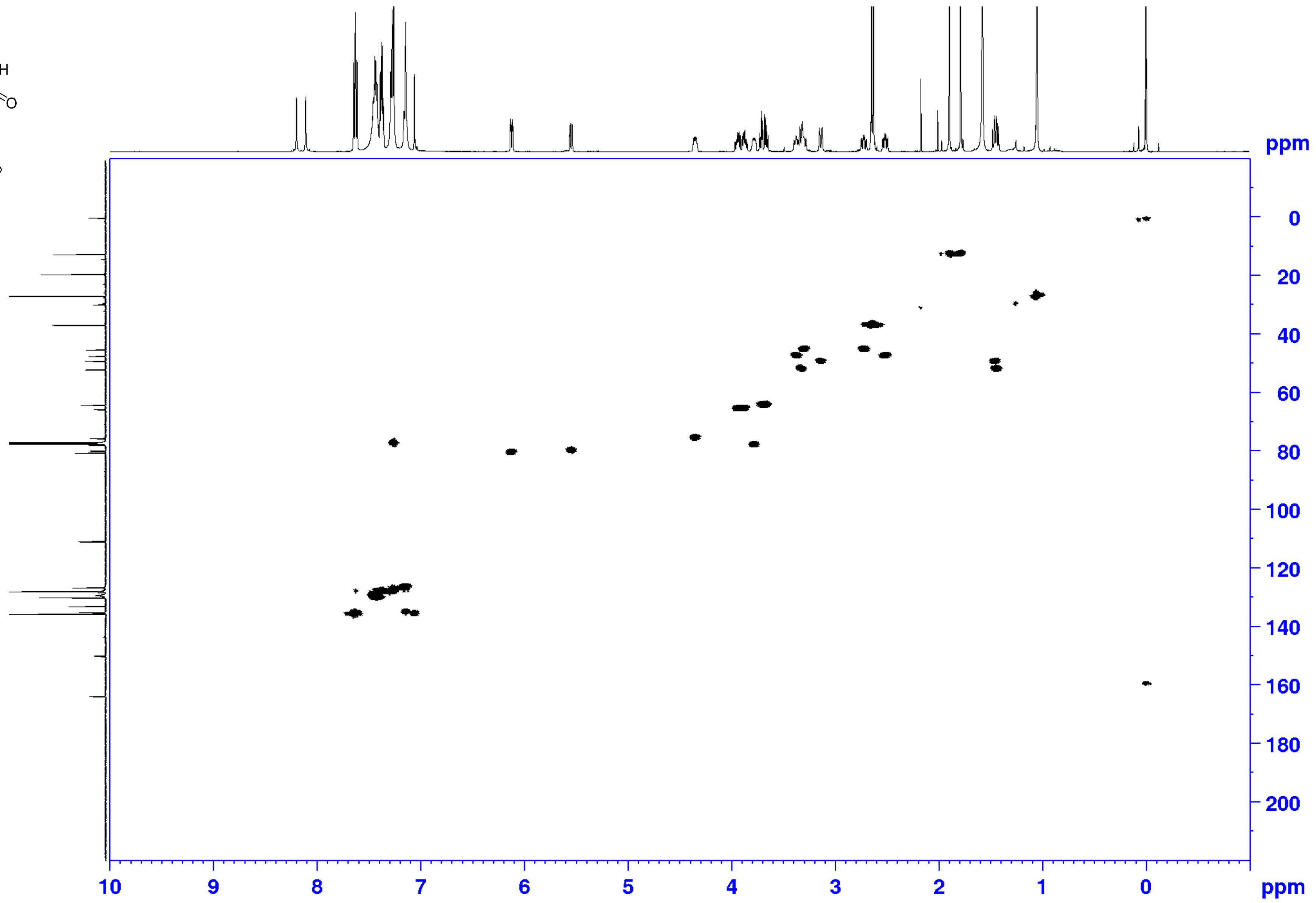

HMBC ($CDCl_3$) of (*R*p)-**23**

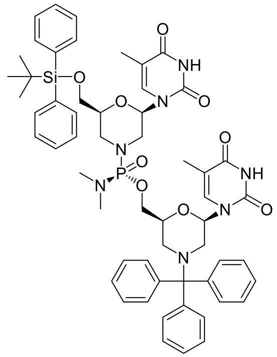

(*R*p)-**23**

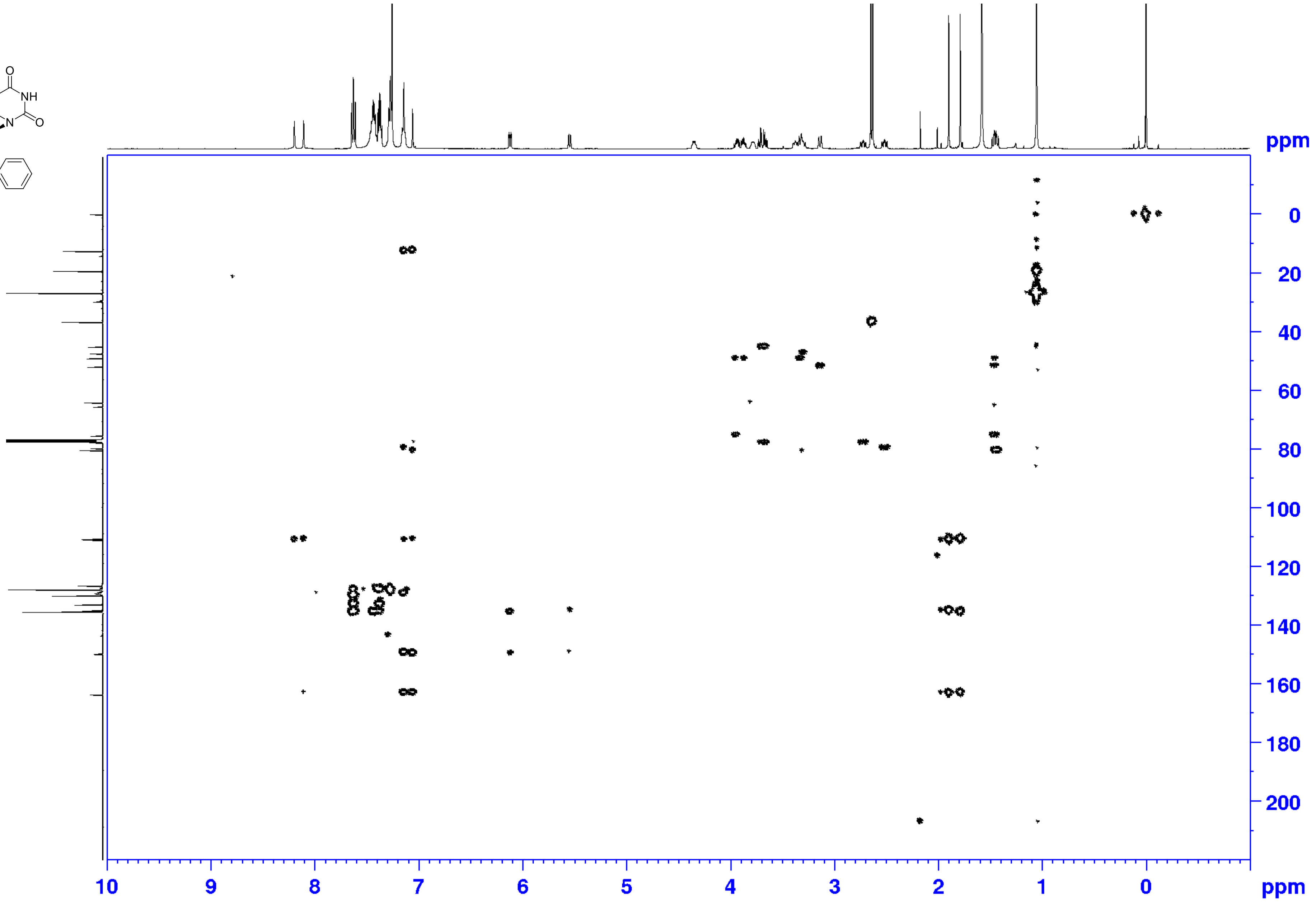

^{1}H NMR (500 MHz, $CDCl_3$) of (*S*p)-**24**

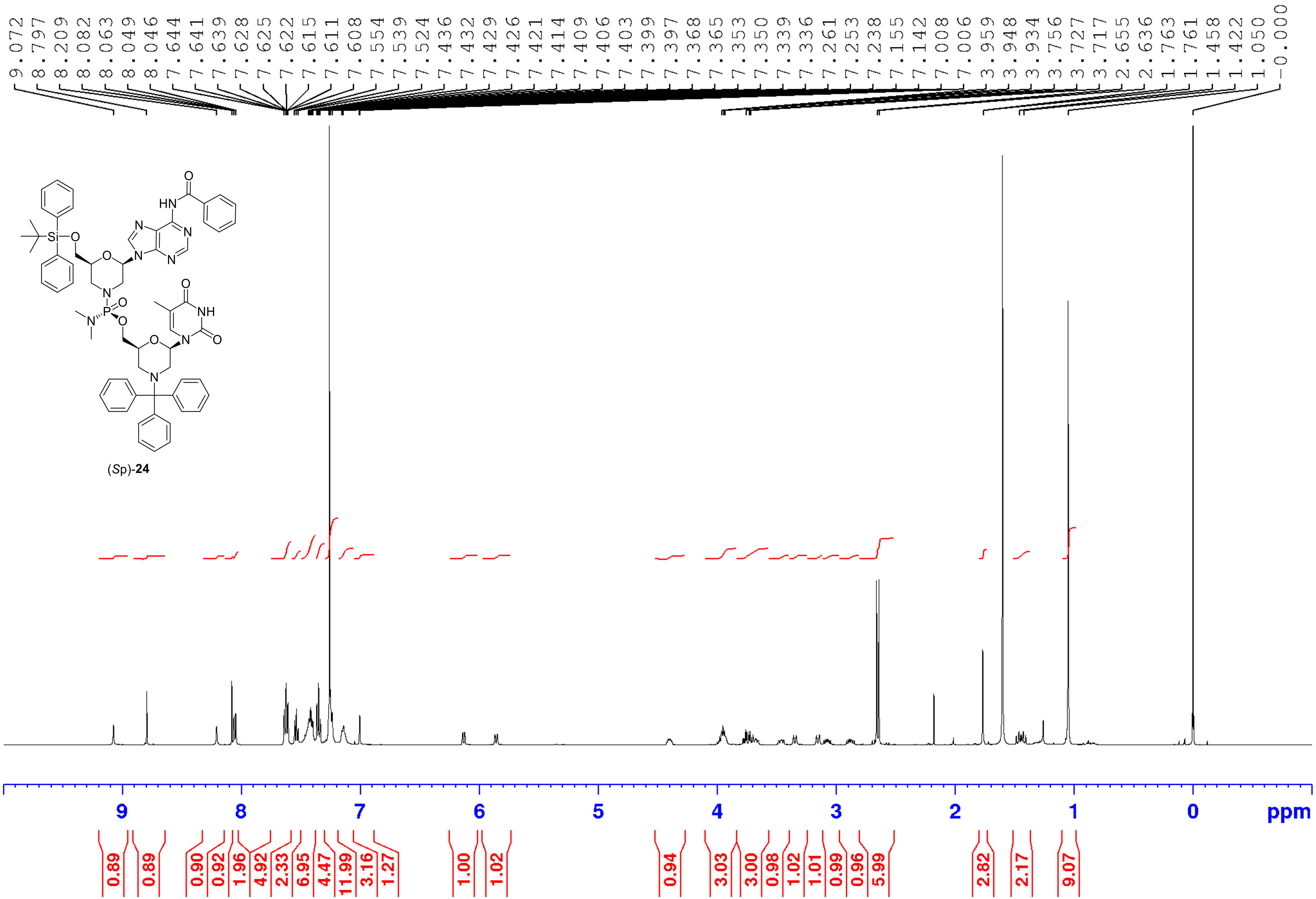

^{13}C {^{1}H} NMR (126 MHz, CDCl$_3$) of (*R*p)-**23**

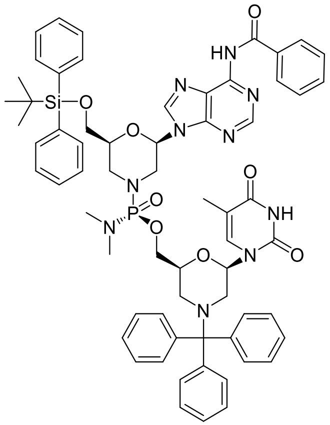

(*S*p)-**24**

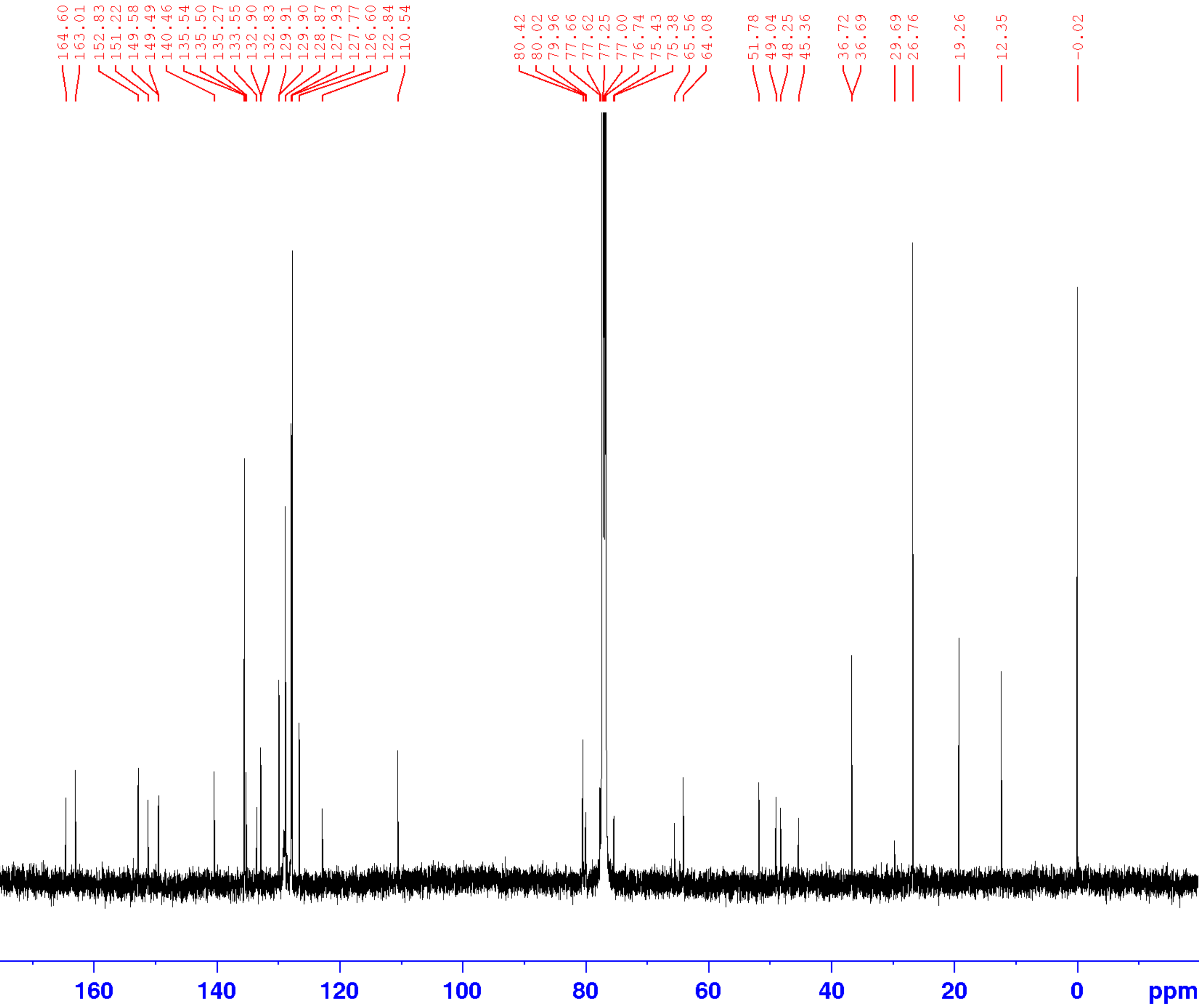

^{31}P {^{1}H} NMR (202 MHz, $CDCl_3$) of (*S*p)-**24**

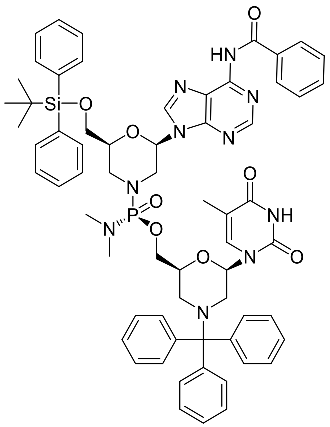

(*S*p)-**24**

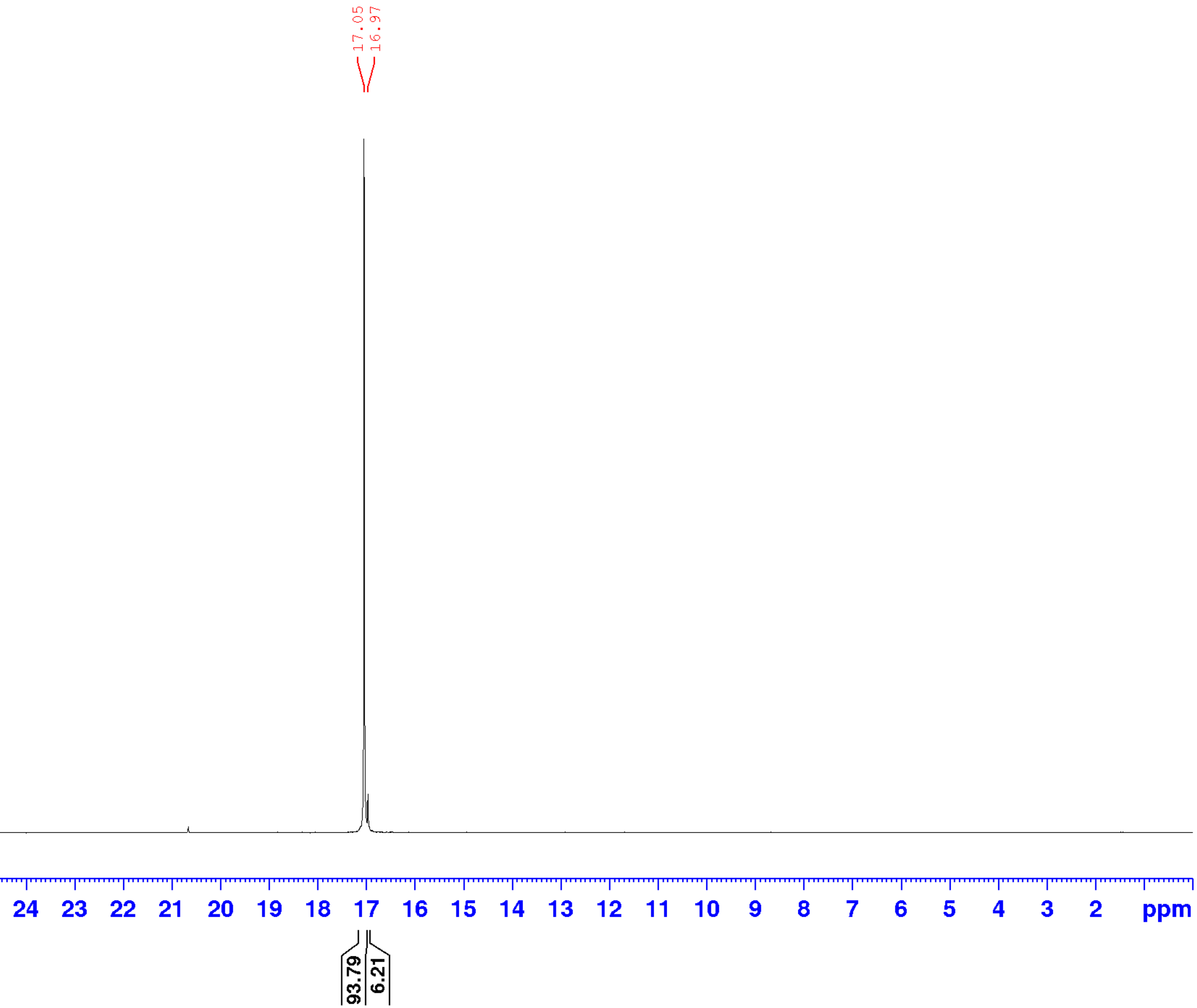

COSY ($CDCl_3$) of (*S*p)-**24**

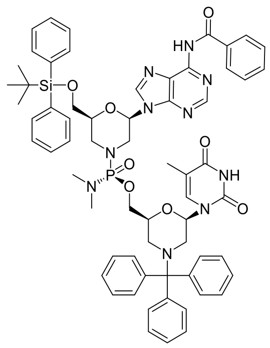

(*S*p)-**24**

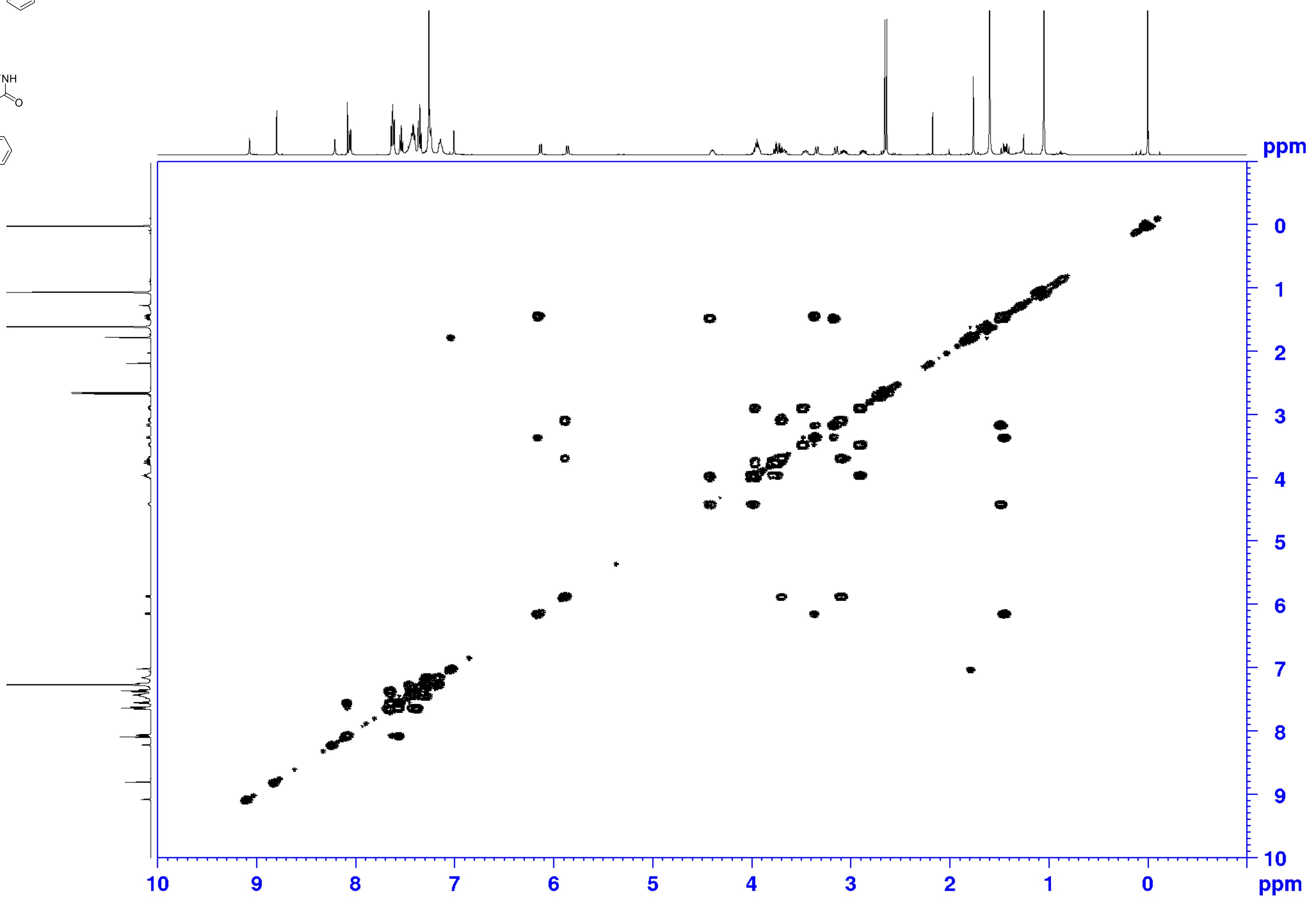

HSQC ($CDCl_3$) of (*S*p)-**24**

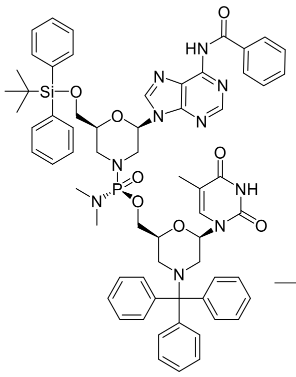

(*S*p)-**24**

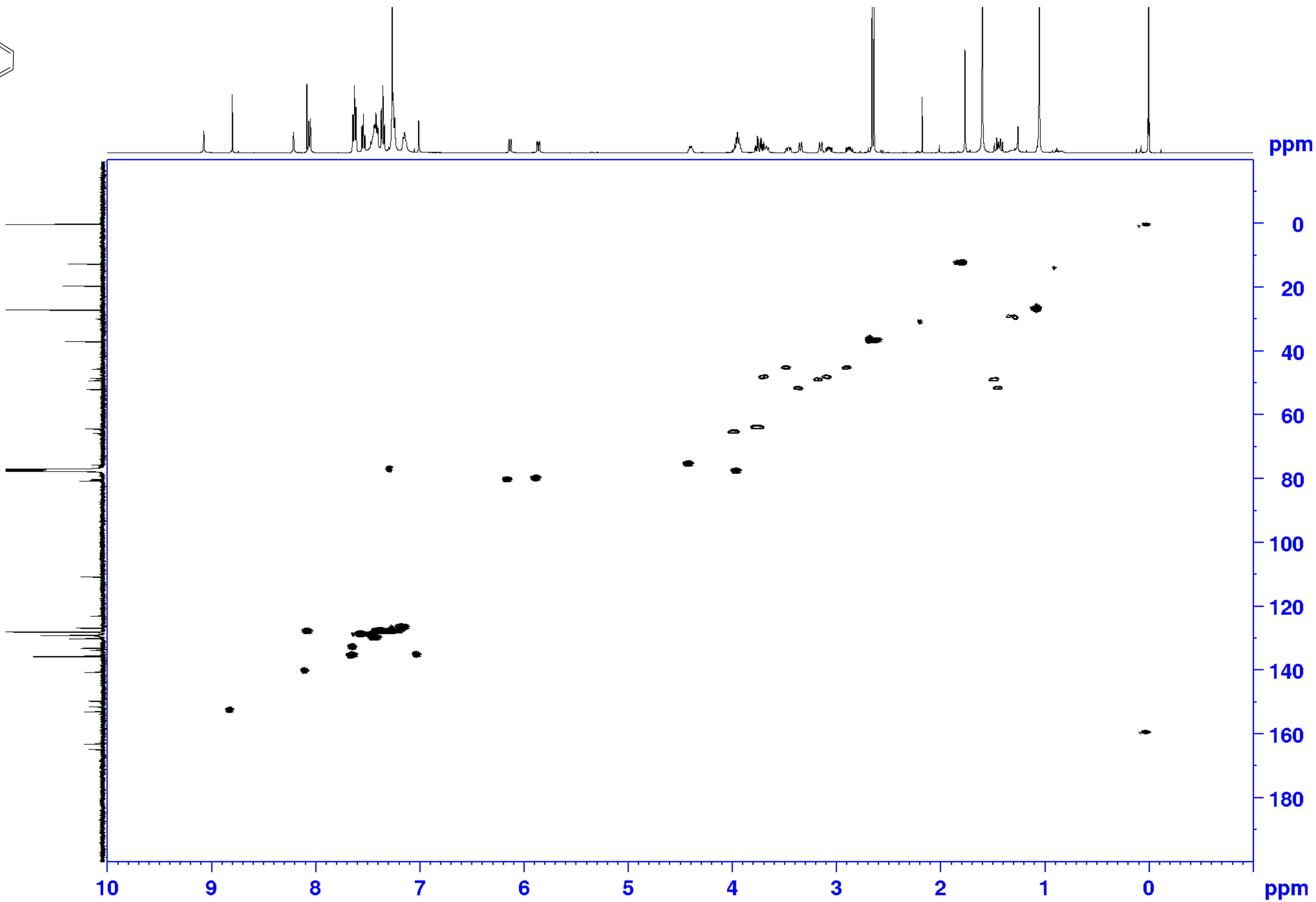

HMBC ($CDCl_3$) of (*S*p)-**24**

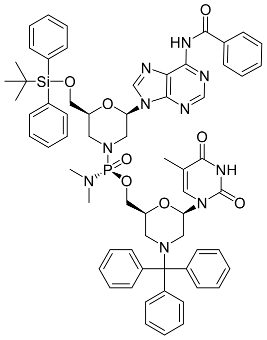

(*S*p)-**24**

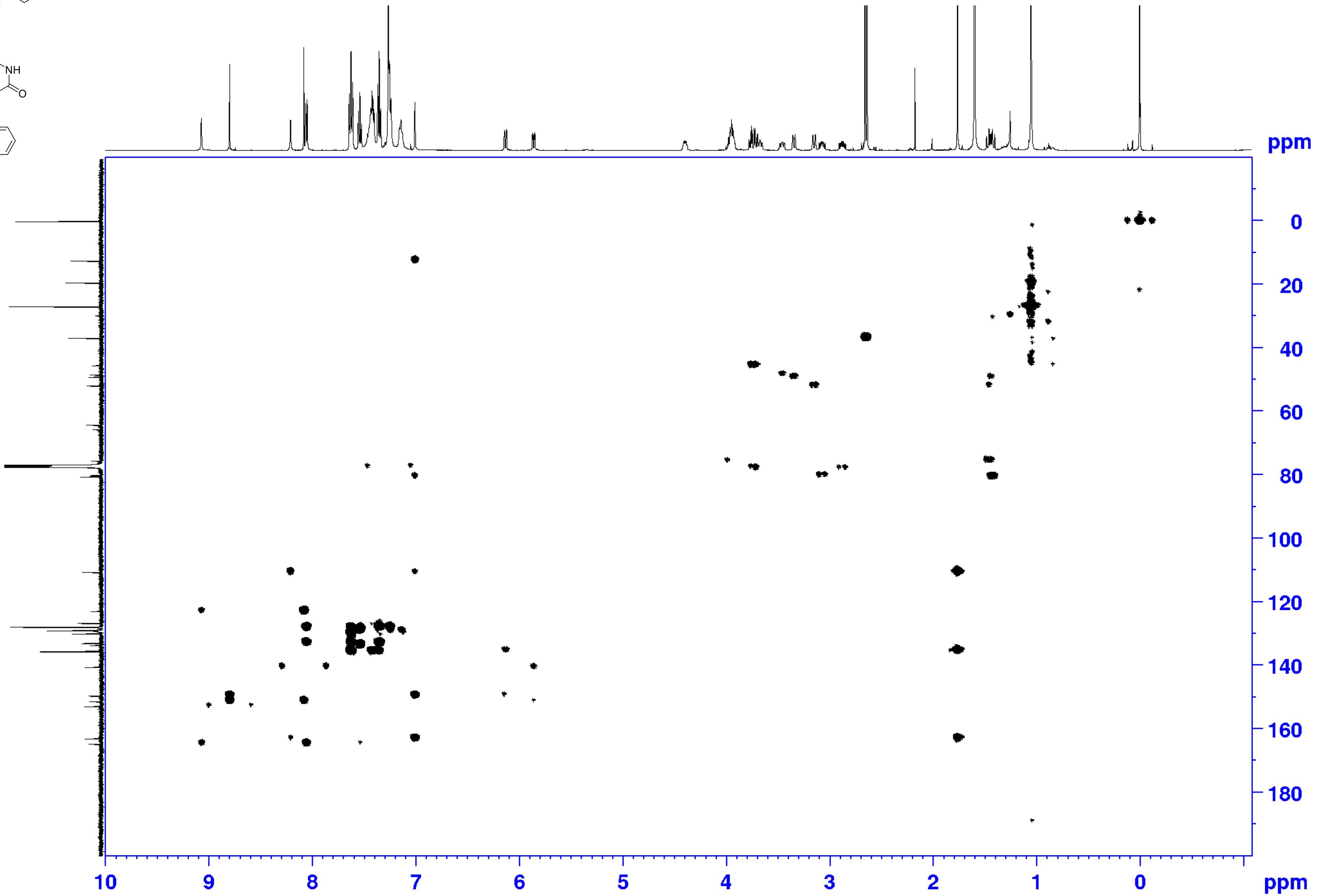

^{1}H NMR (500 MHz, $CDCl_3$) of (*R*p)-**24**

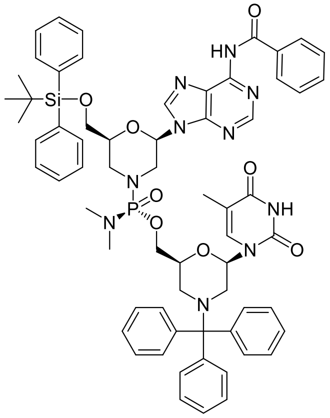

(*R*p)-**24**

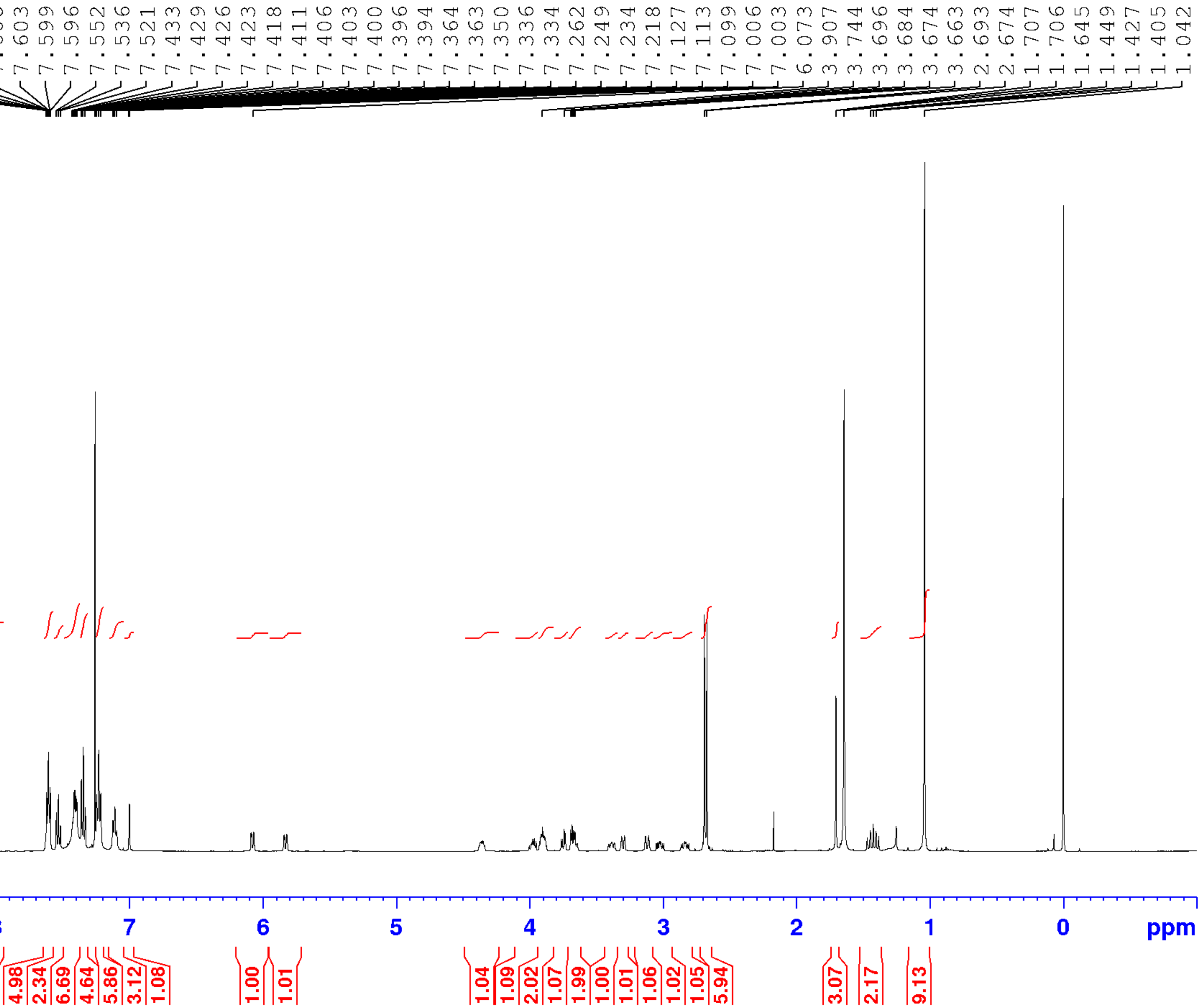

^{13}C {^{1}H} NMR (126 MHz, $CDCl_3$) of (*R*p)-**24**

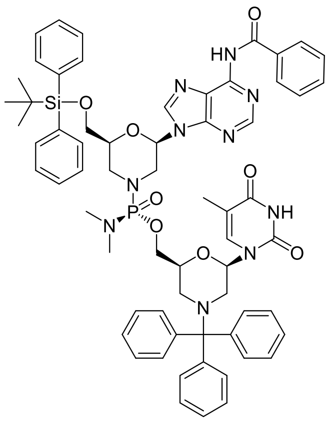

(*R*p)-**24**

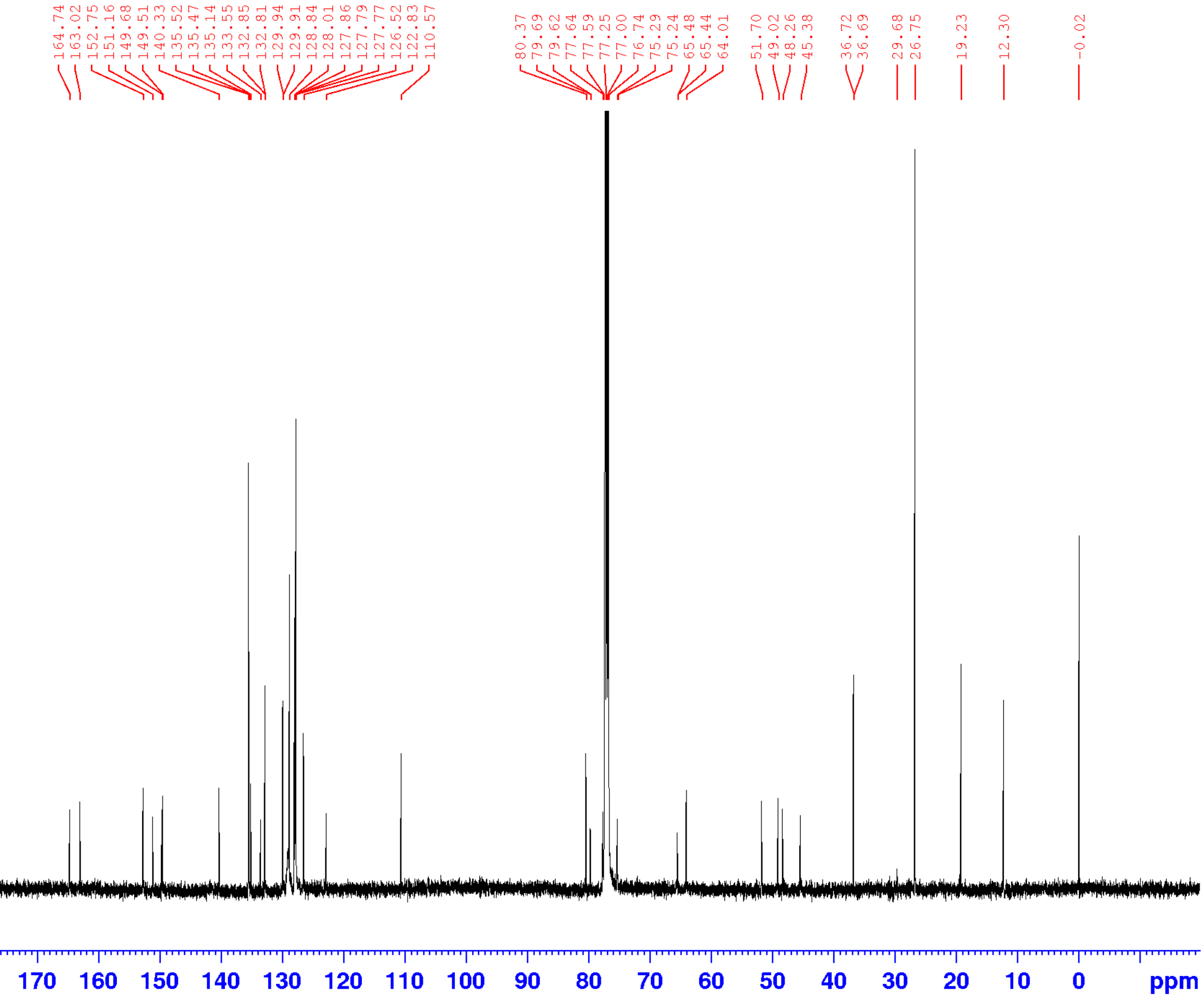

^{31}P {^{1}H} NMR (202 MHz, $CDCl_3$) of (*R*p)-**24**

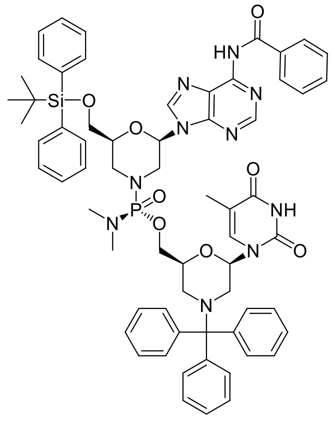

(*R*p)-**24**

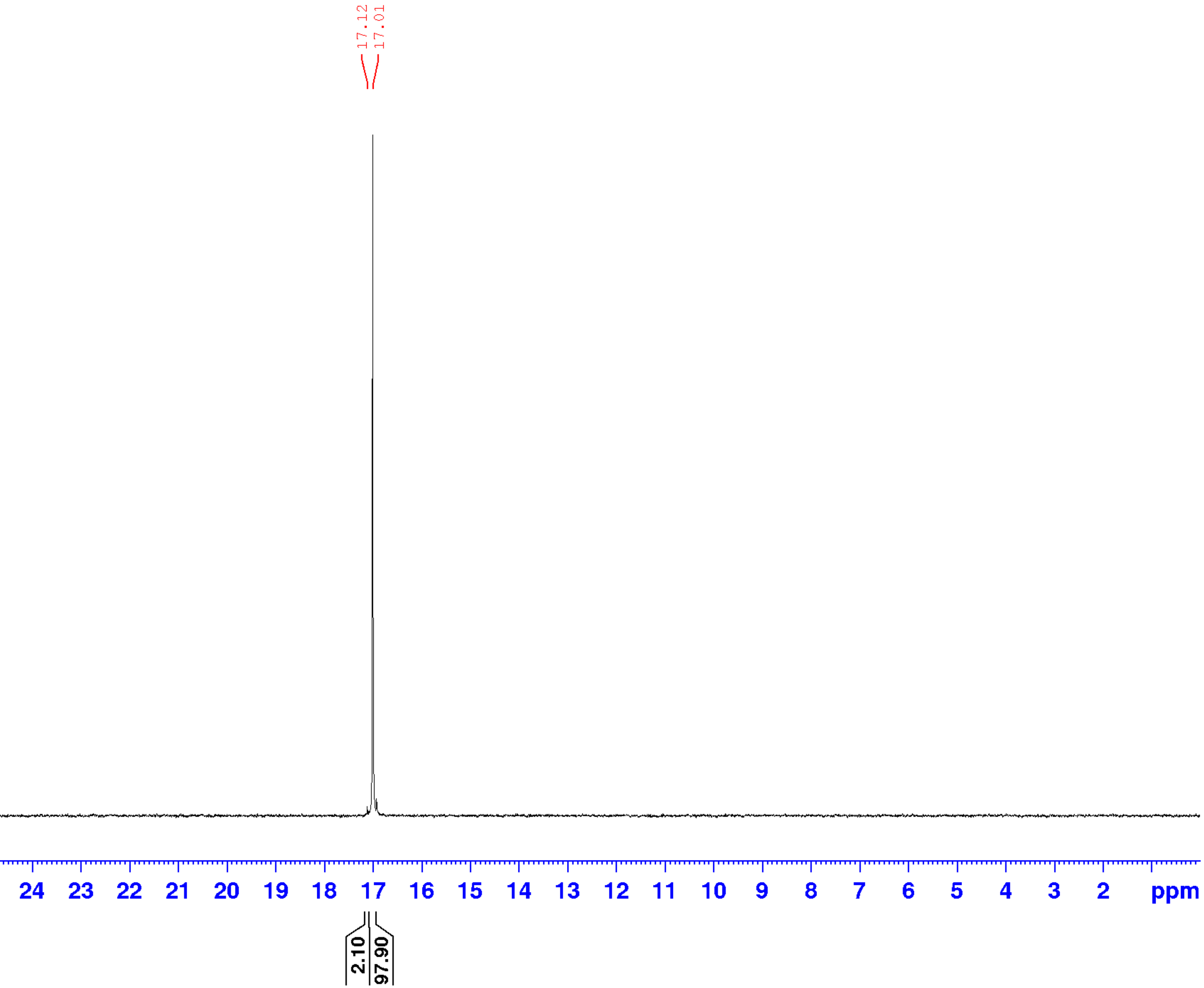

COSY ($CDCl_3$) of (*R*p)-**24**

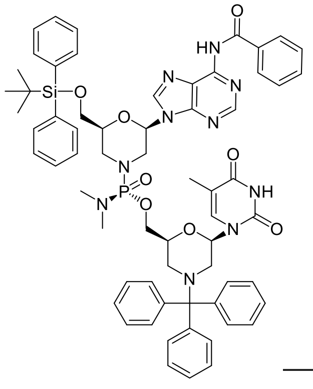

(*R*p)-**24**

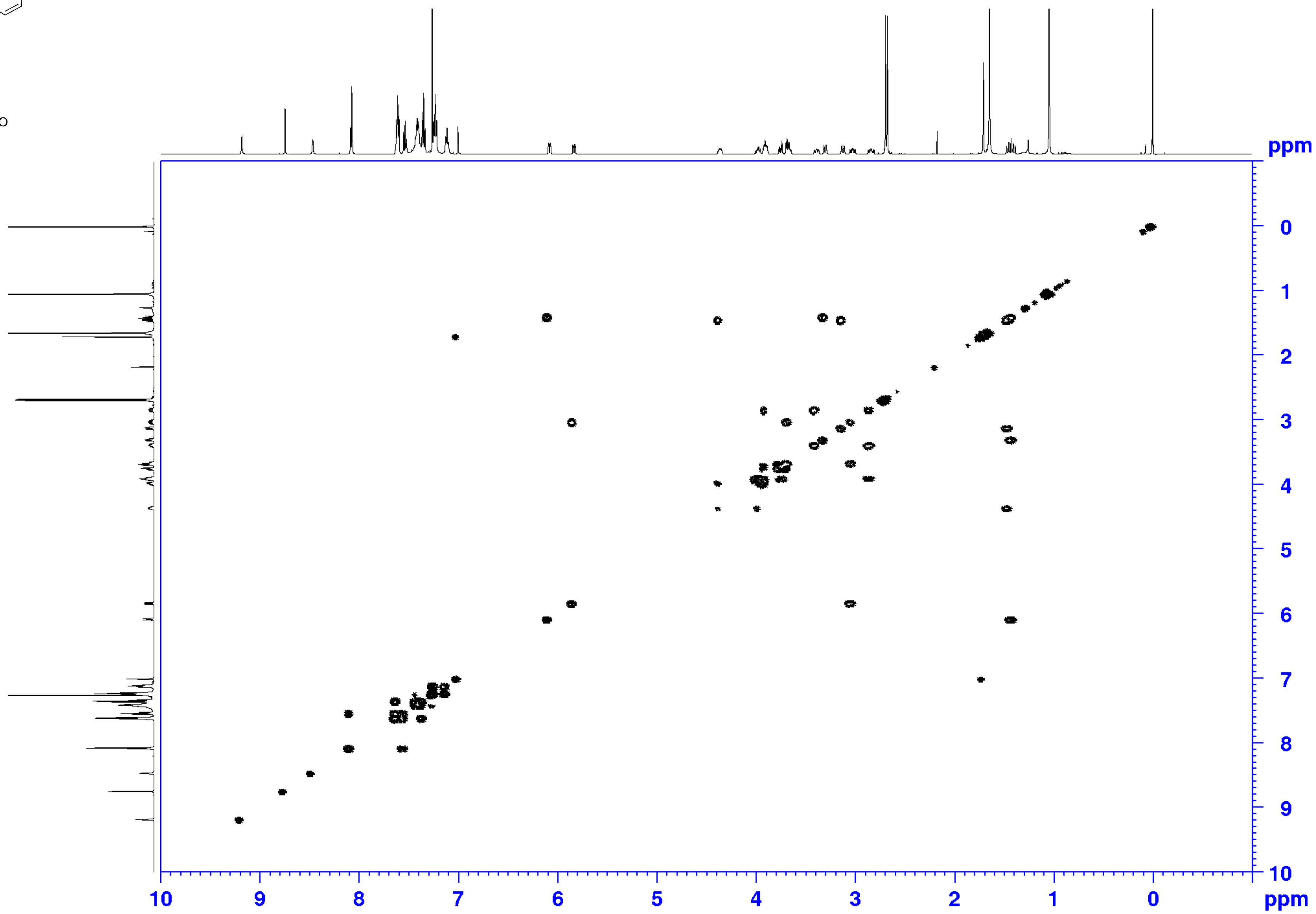

HSQC ($CDCl_3$) of (*R*p)-**24**

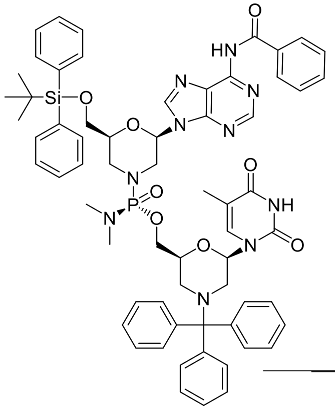
(*R*p)-**24**

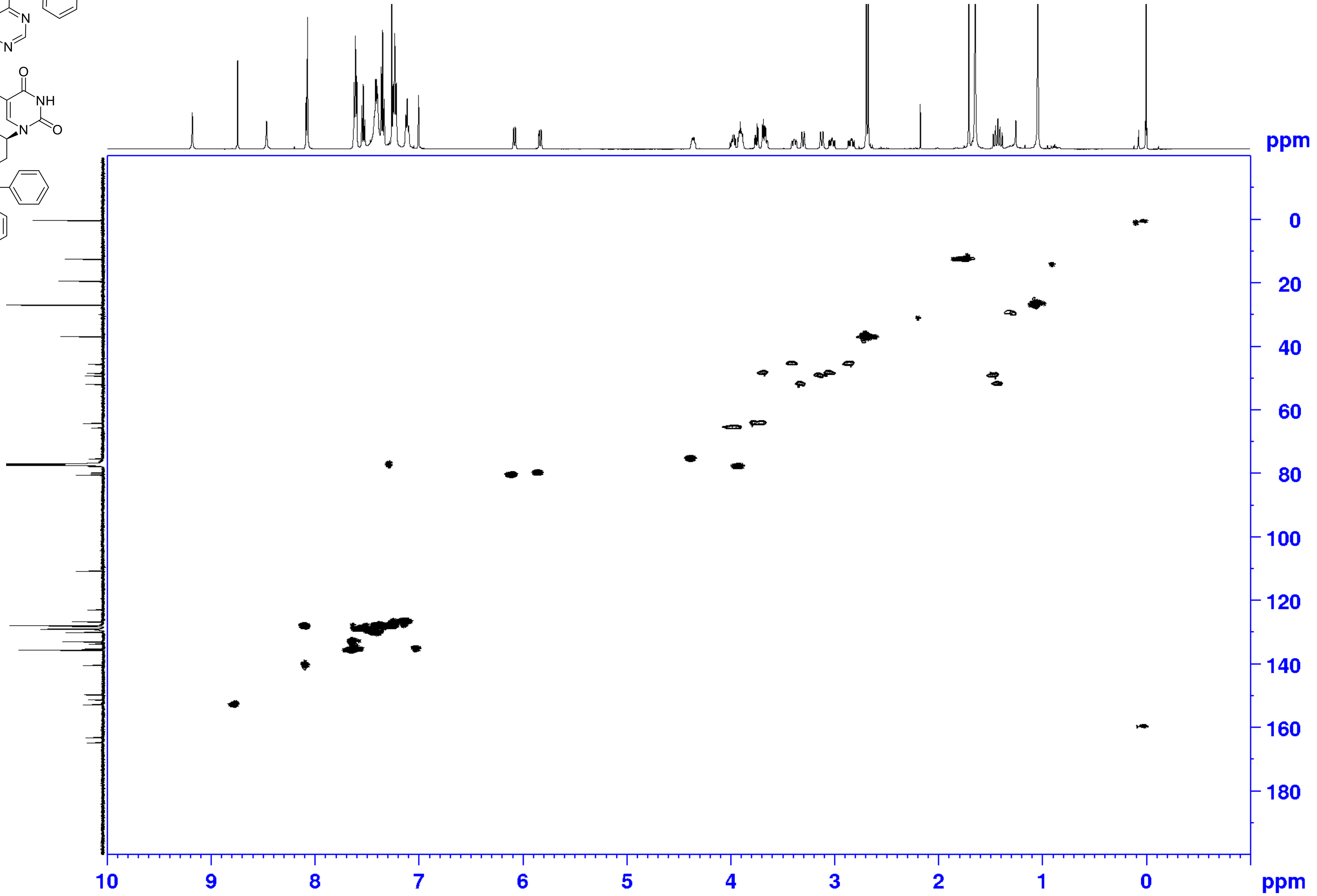

HMBC ($CDCl_3$) of (*R*p)-**24**

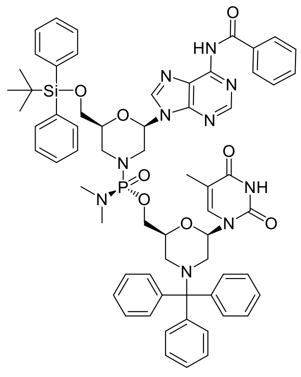

(*R*p)-**24**

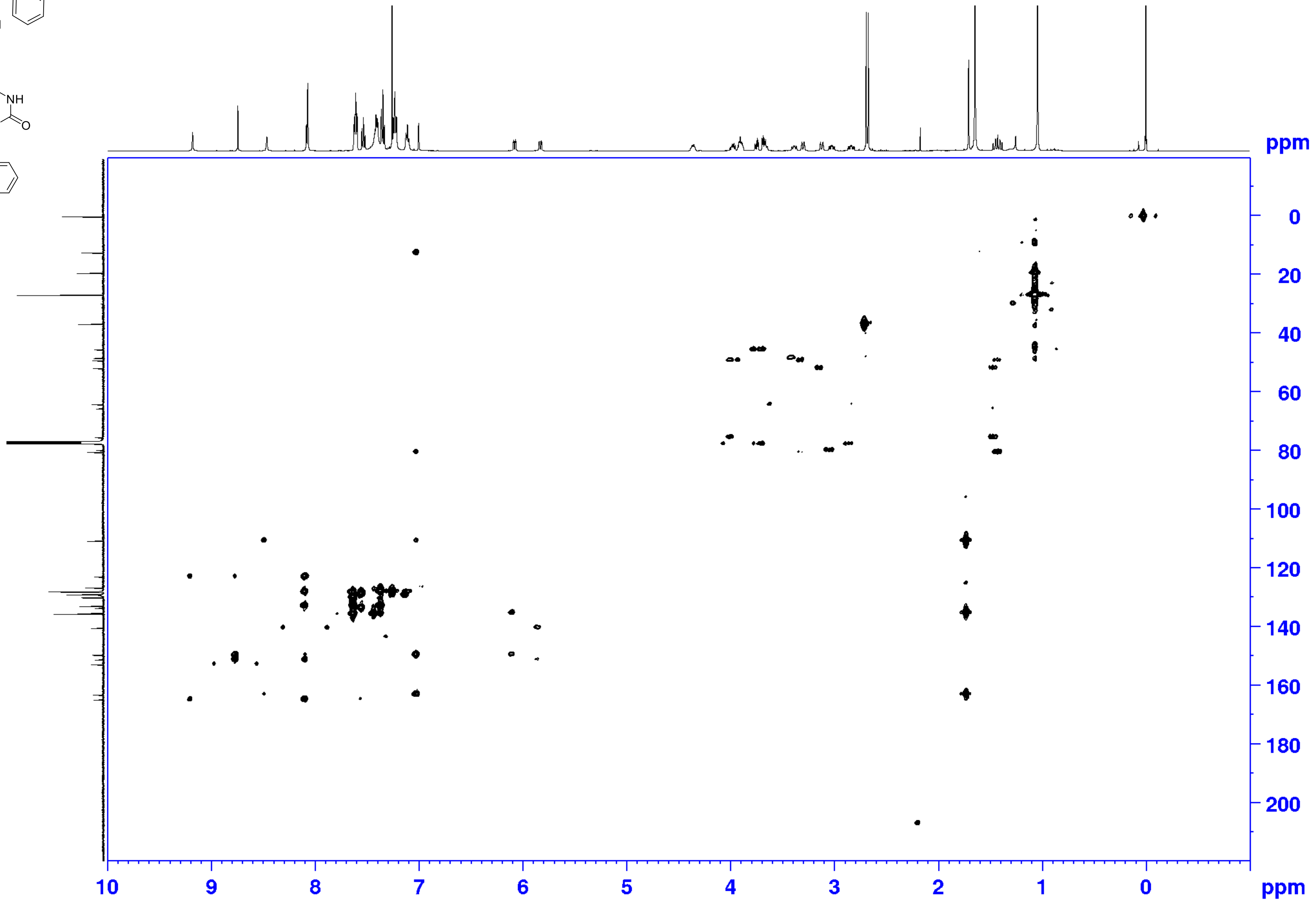

^{1}H NMR (500 MHz, $CDCl_3$) of (*S*p)-**25**

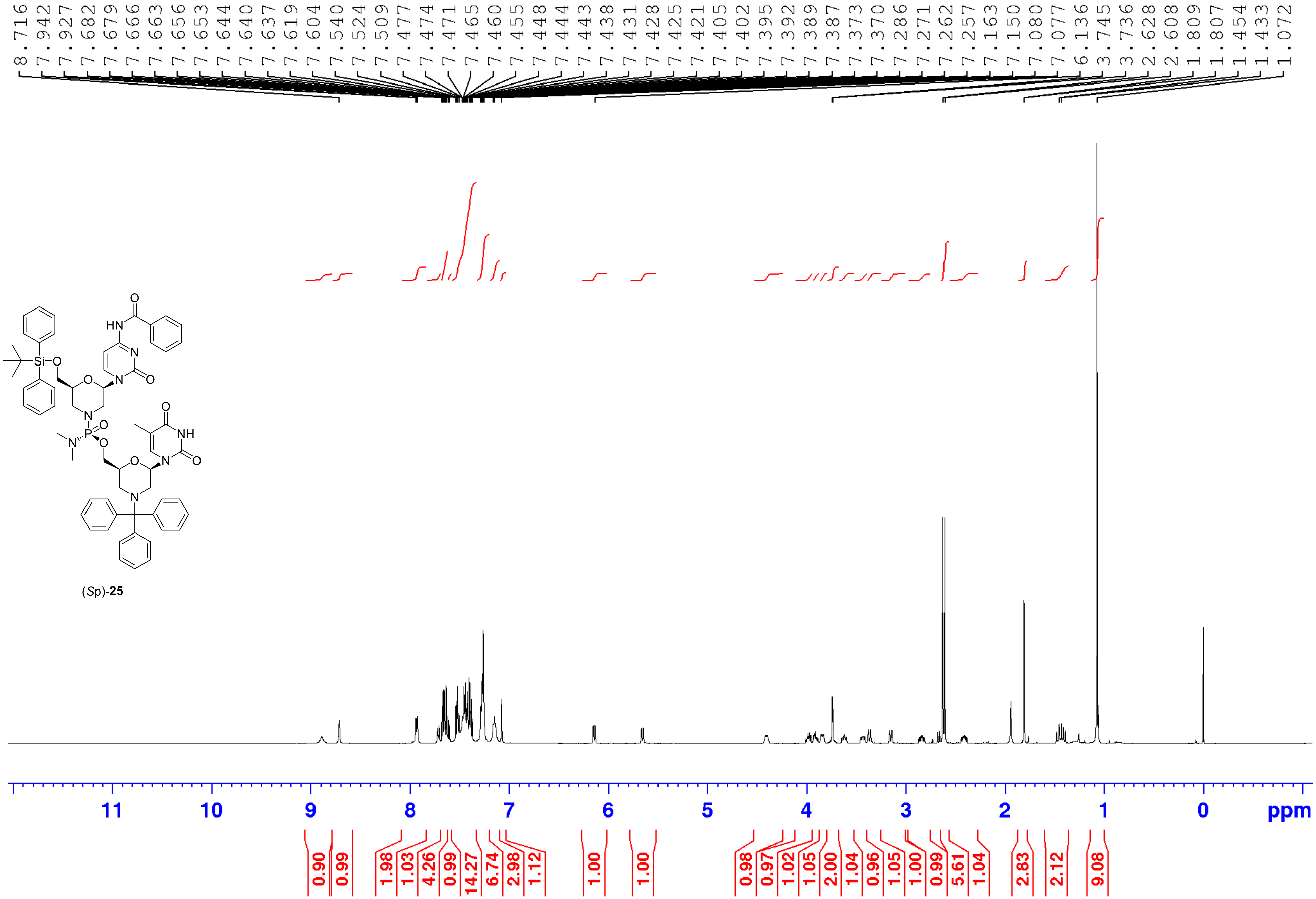

(*S*p)-**25**

^{13}C {^1H} NMR (126 MHz, $CDCl_3$) of (*S*p)-**25**

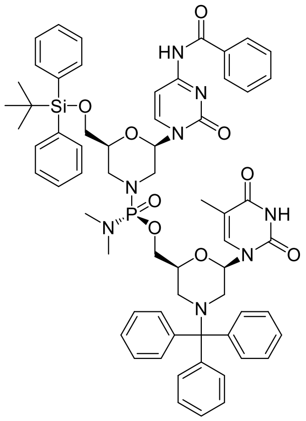

(*S*p)-**25**

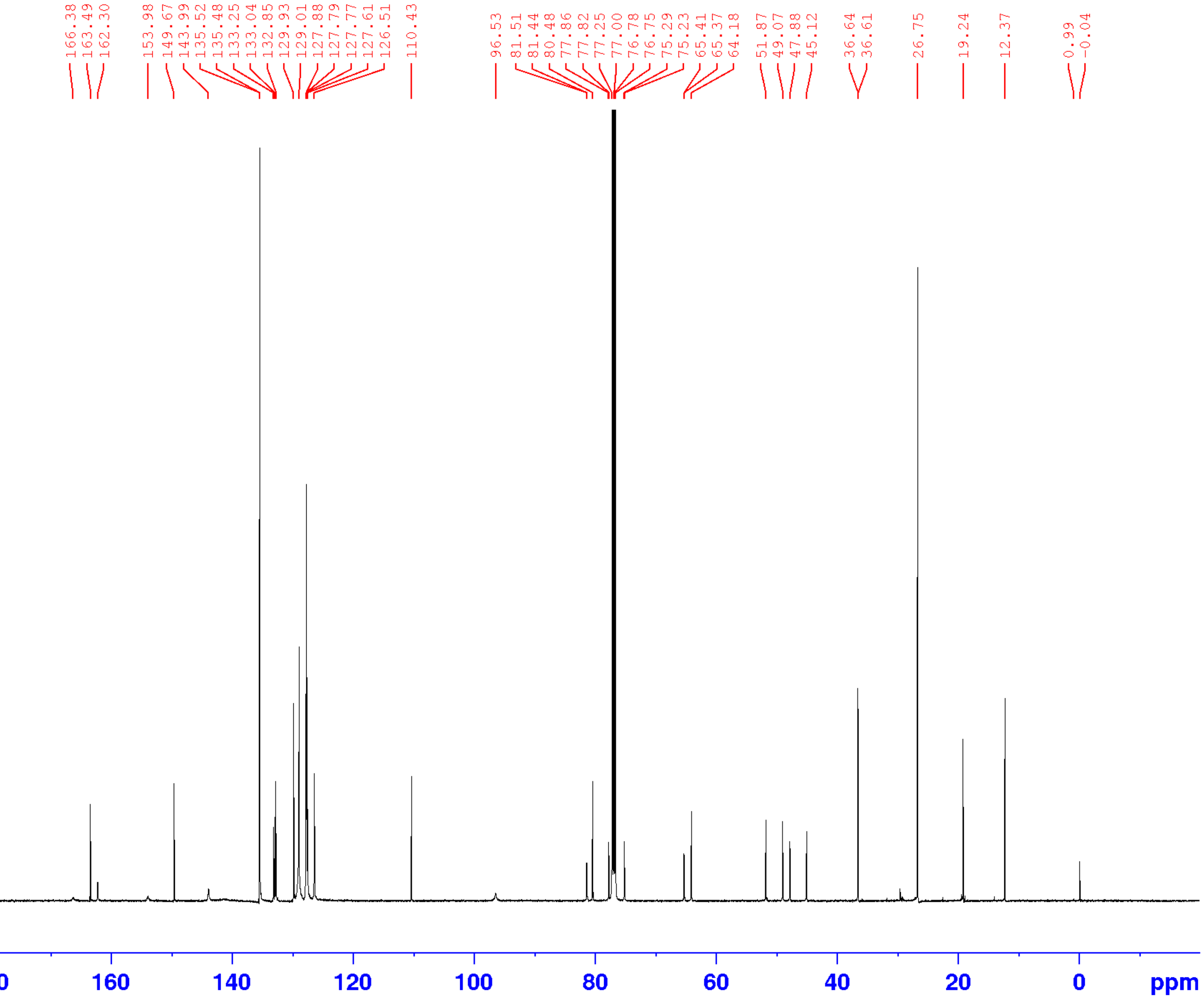

^{31}P {^{1}H} NMR (202 MHz, $CDCl_3$) of (*S*p)-**25**

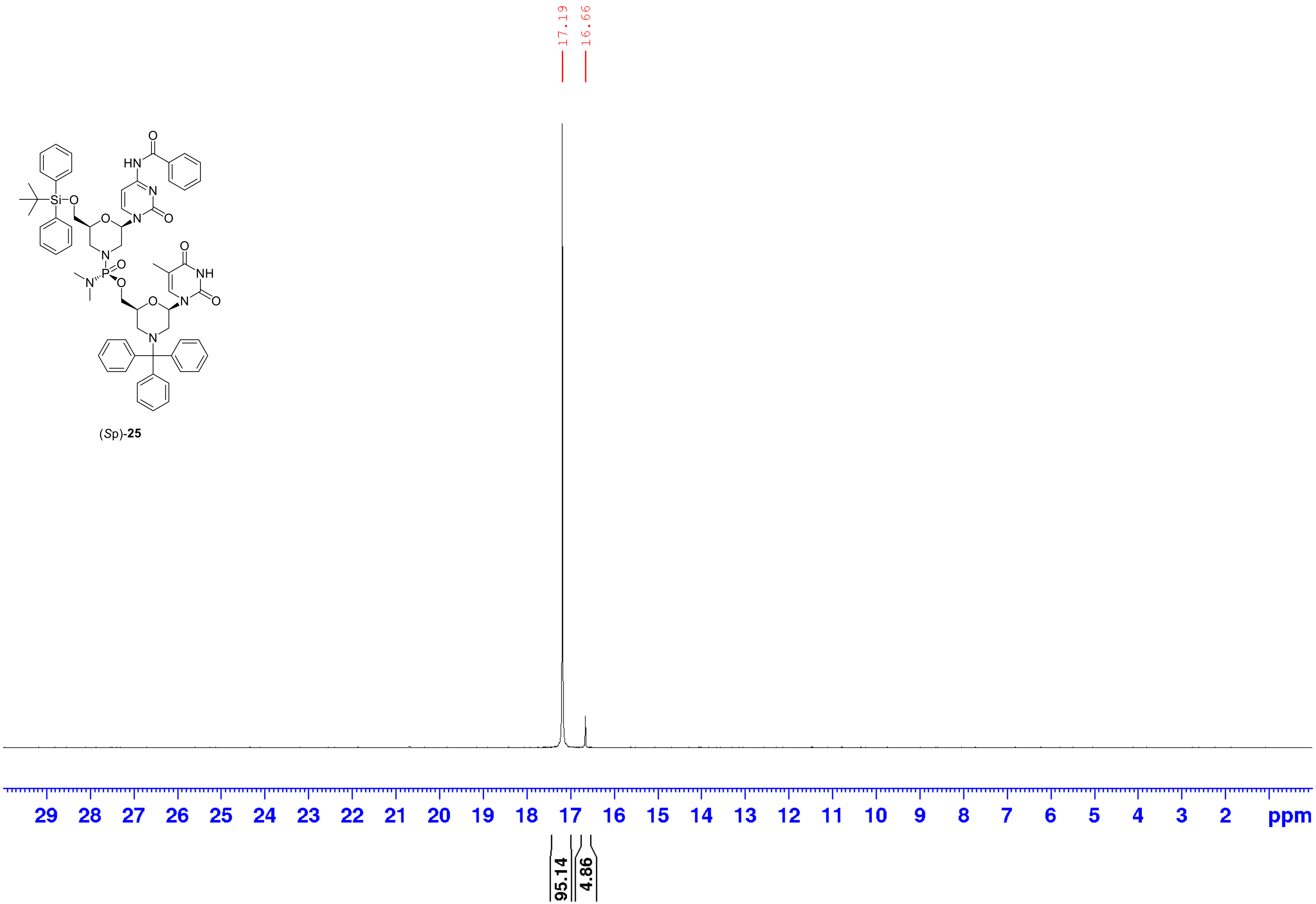

COSY ($CDCl_3$) of (*S*p)-**25**

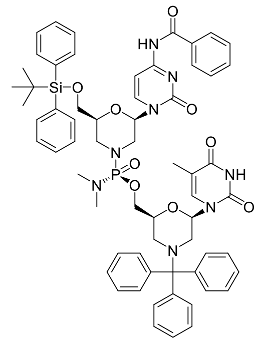

(*S*p)-**25**

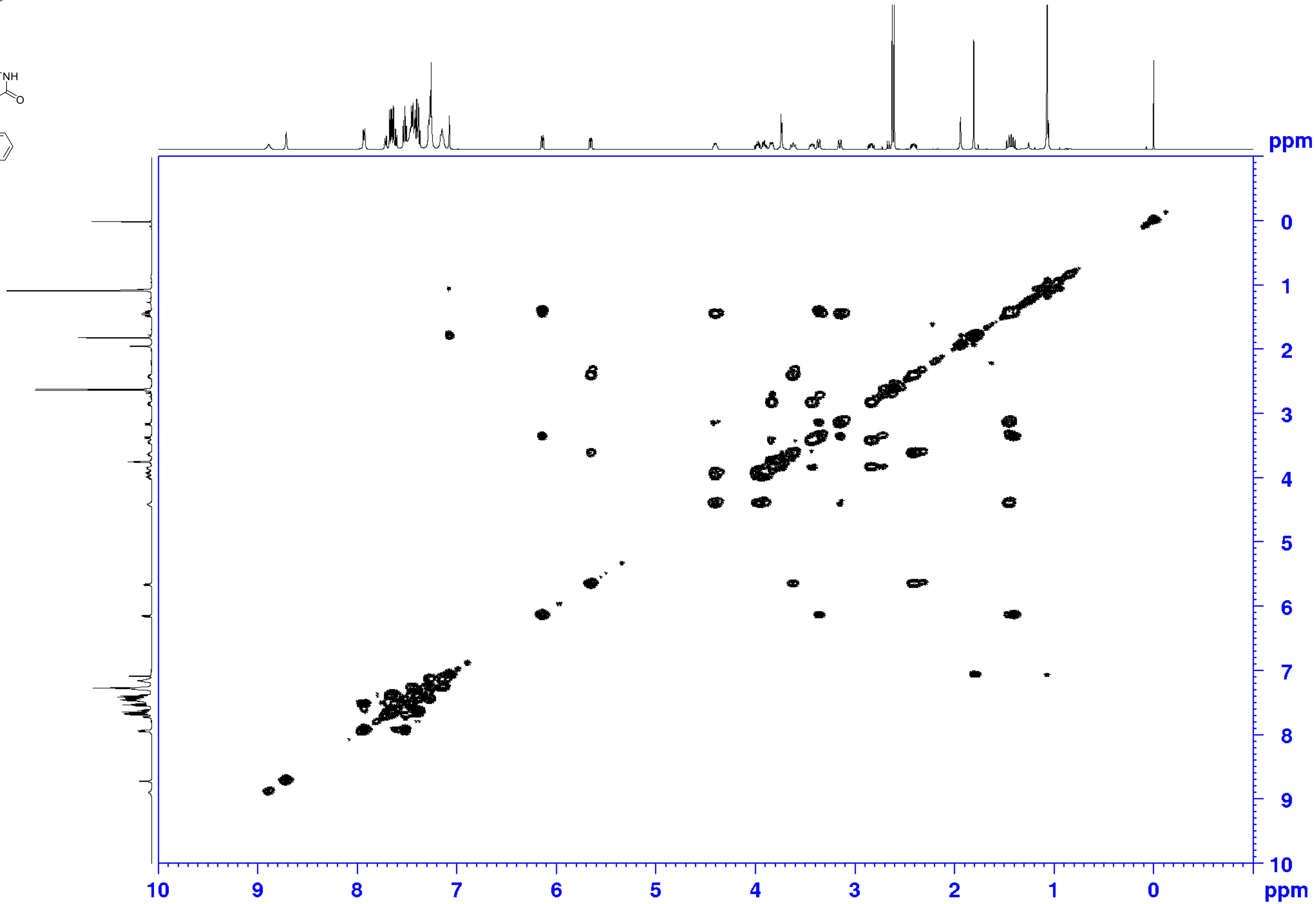

HSQC ($CDCl_3$) of (*S*p)-**25**

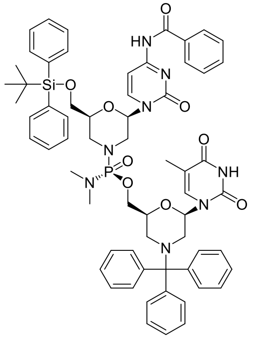

(*S*p)-**25**

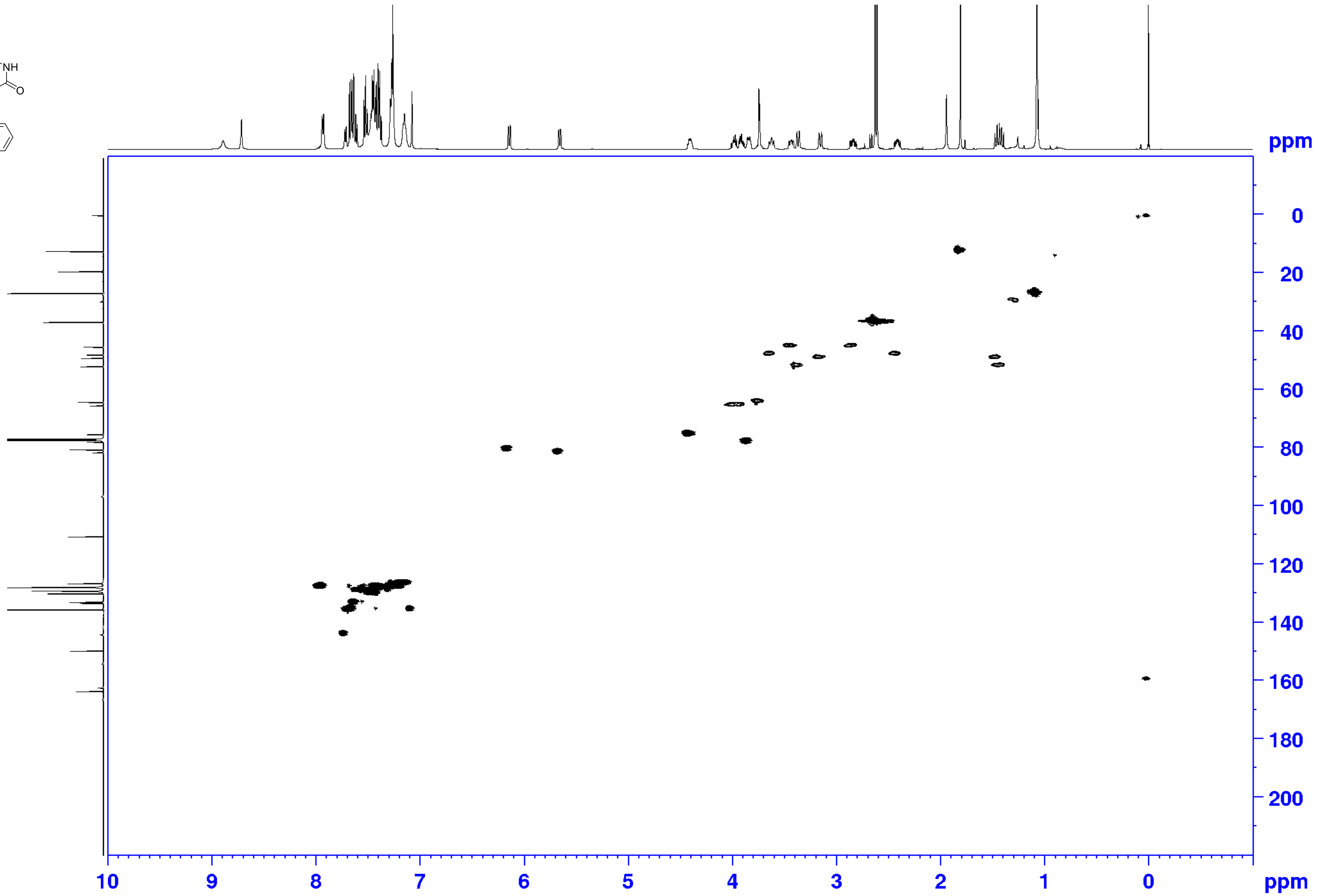

HMBC ($CDCl_3$) of (*S*p)-**25**

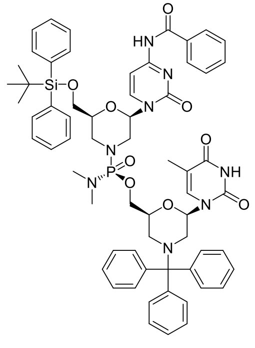

(*S*p)-**25**

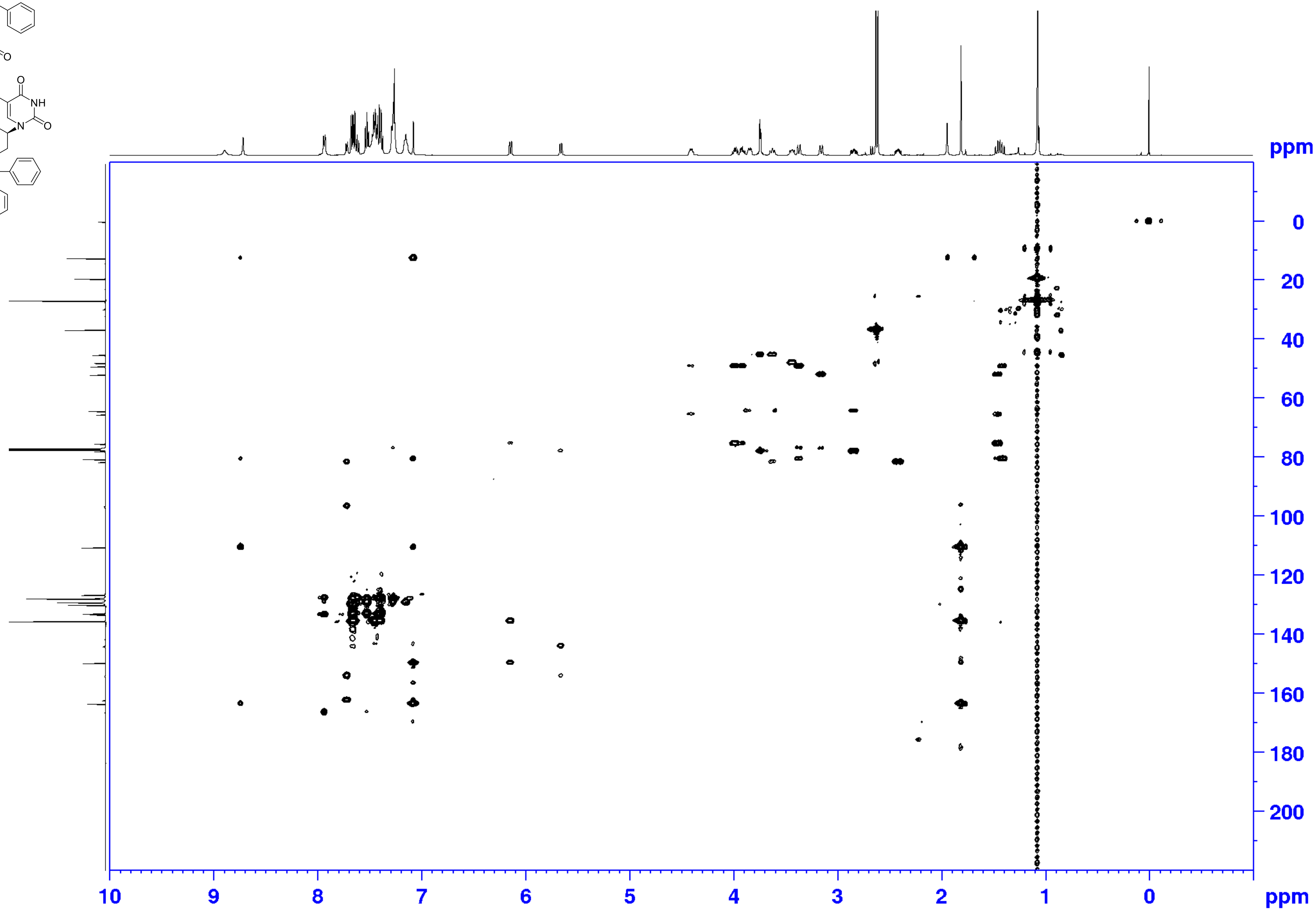

^{1}H NMR (500 MHz, CDCl$_3$) of (*R*p)-**25**

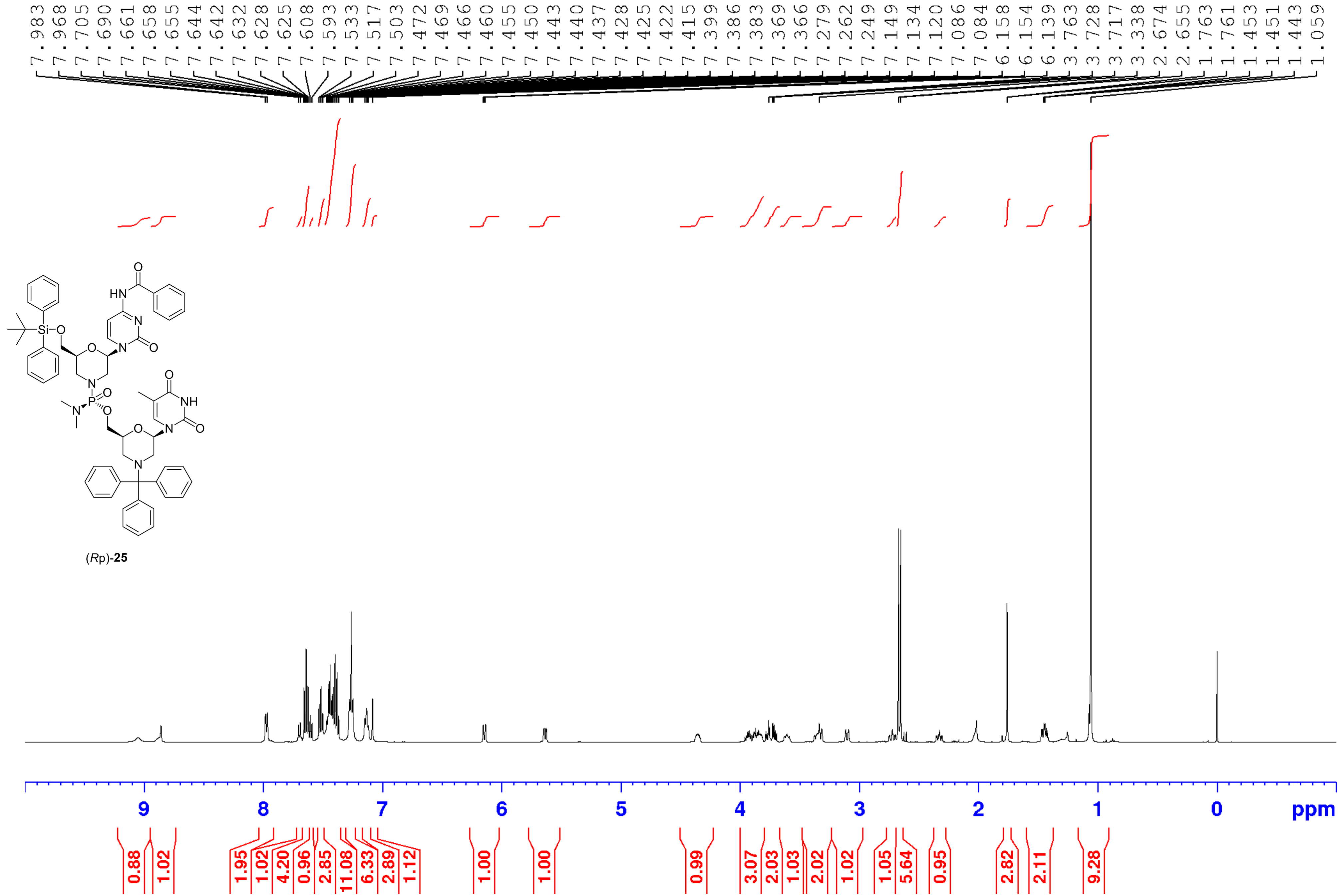

$^{13}C\{^1H\}$ NMR (126 MHz, $CDCl_3$) of (*R*p)-**25**

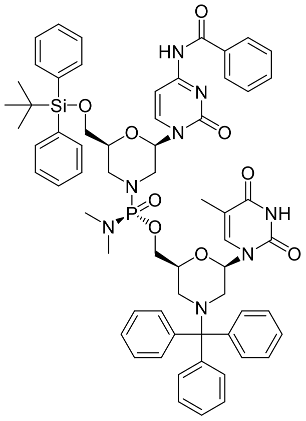

(*R*p)-**25**

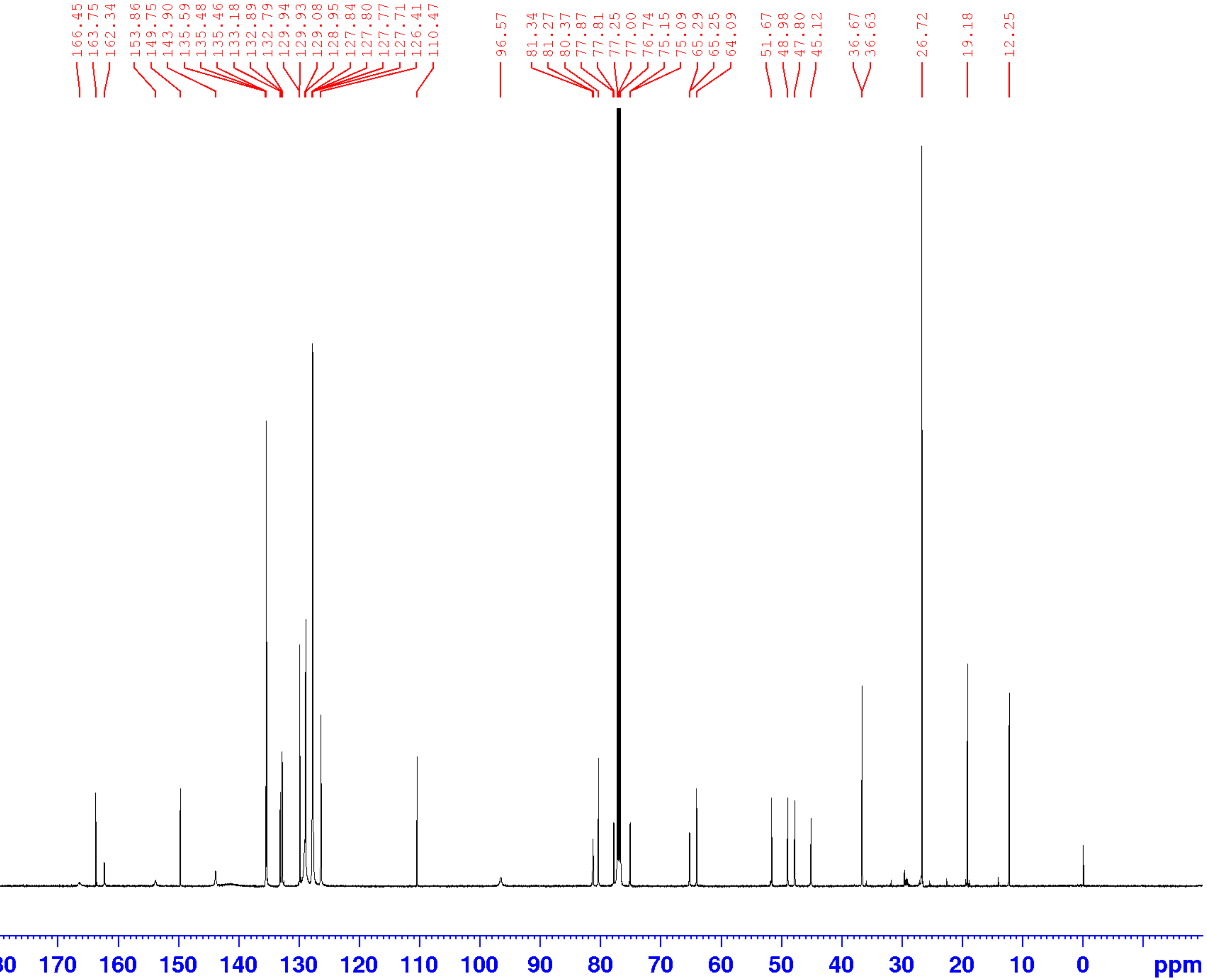

^{31}P {^{1}H} NMR (202 MHz, $CDCl_3$) of (*R*p)-**25**

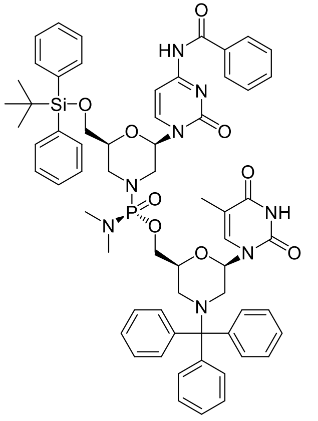

(*R*p)-**25**

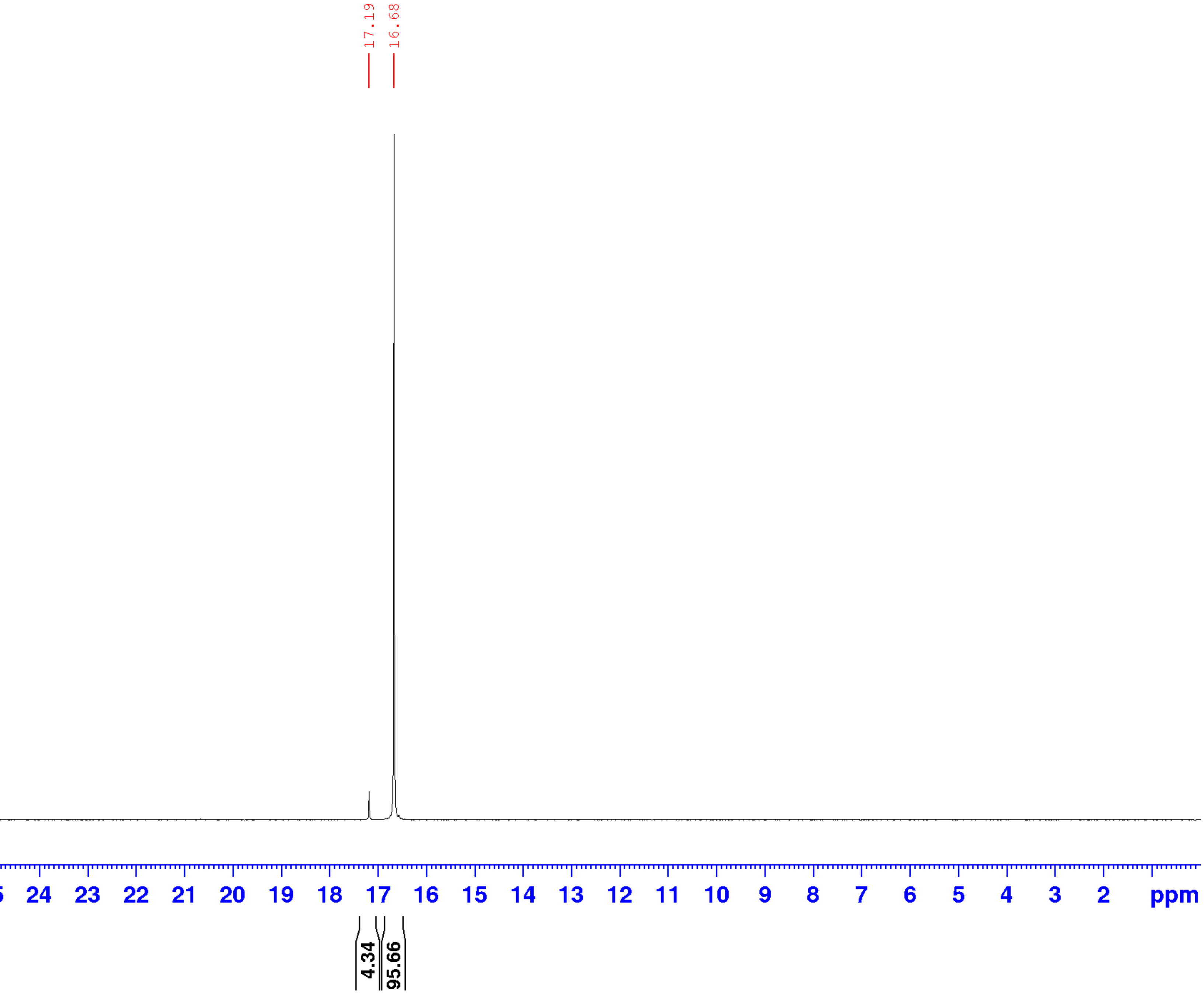

COSY ($CDCl_3$) of (*R*p)-**25**

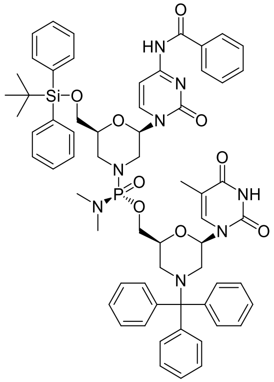

(*R*p)-**25**

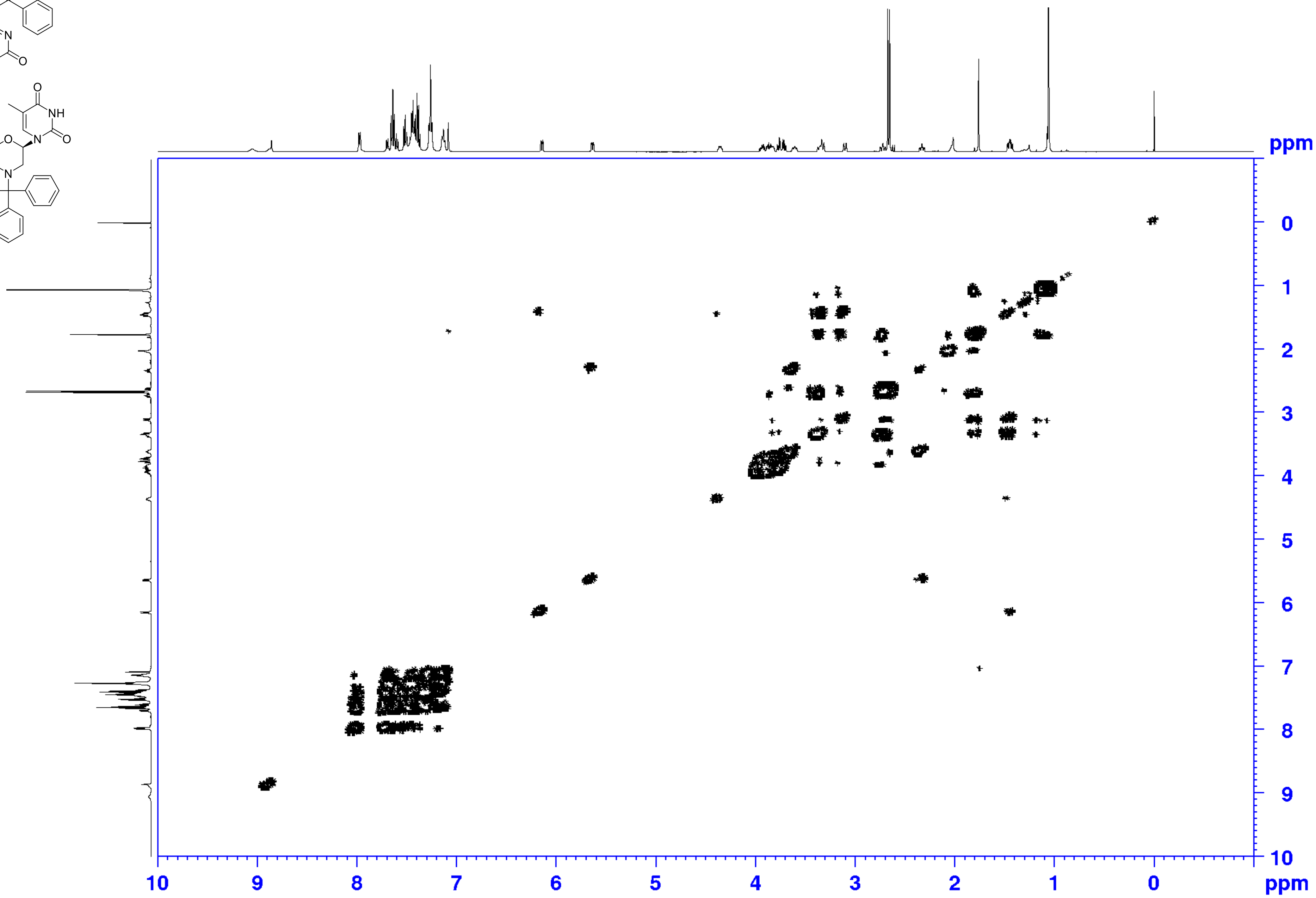

HSQC ($CDCl_3$) of (*R*p)-**25**

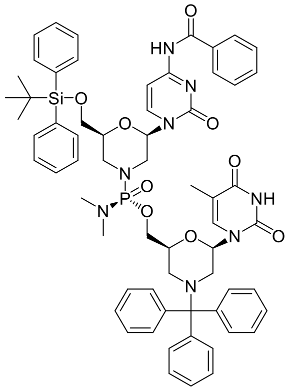

(*R*p)-**25**

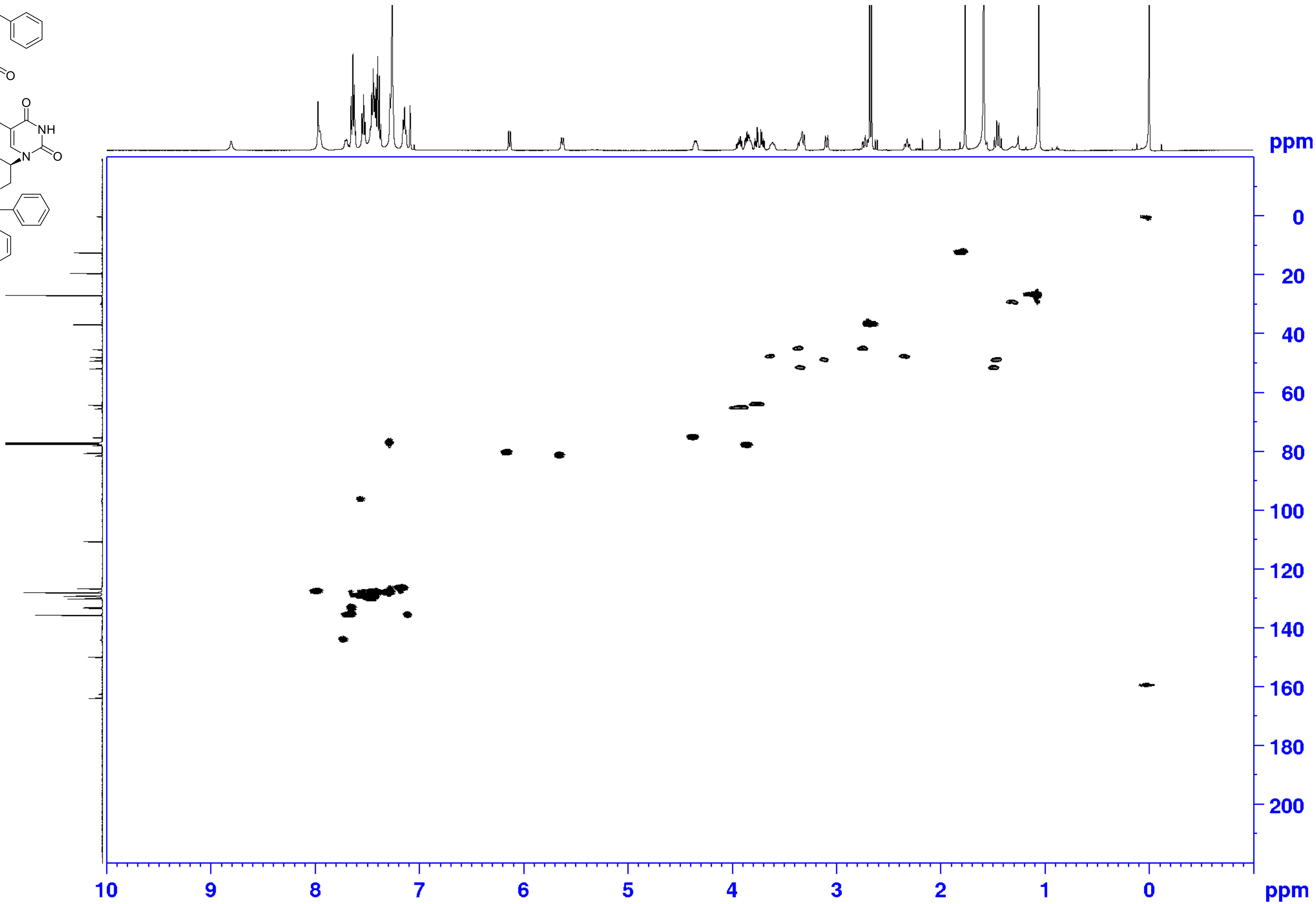

HMBC ($CDCl_3$) of (*R*p)-**25**

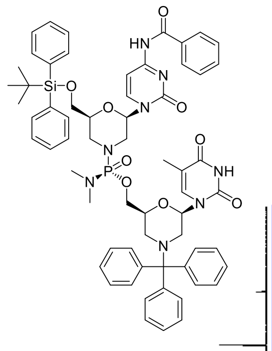

(*R*p)-**25**

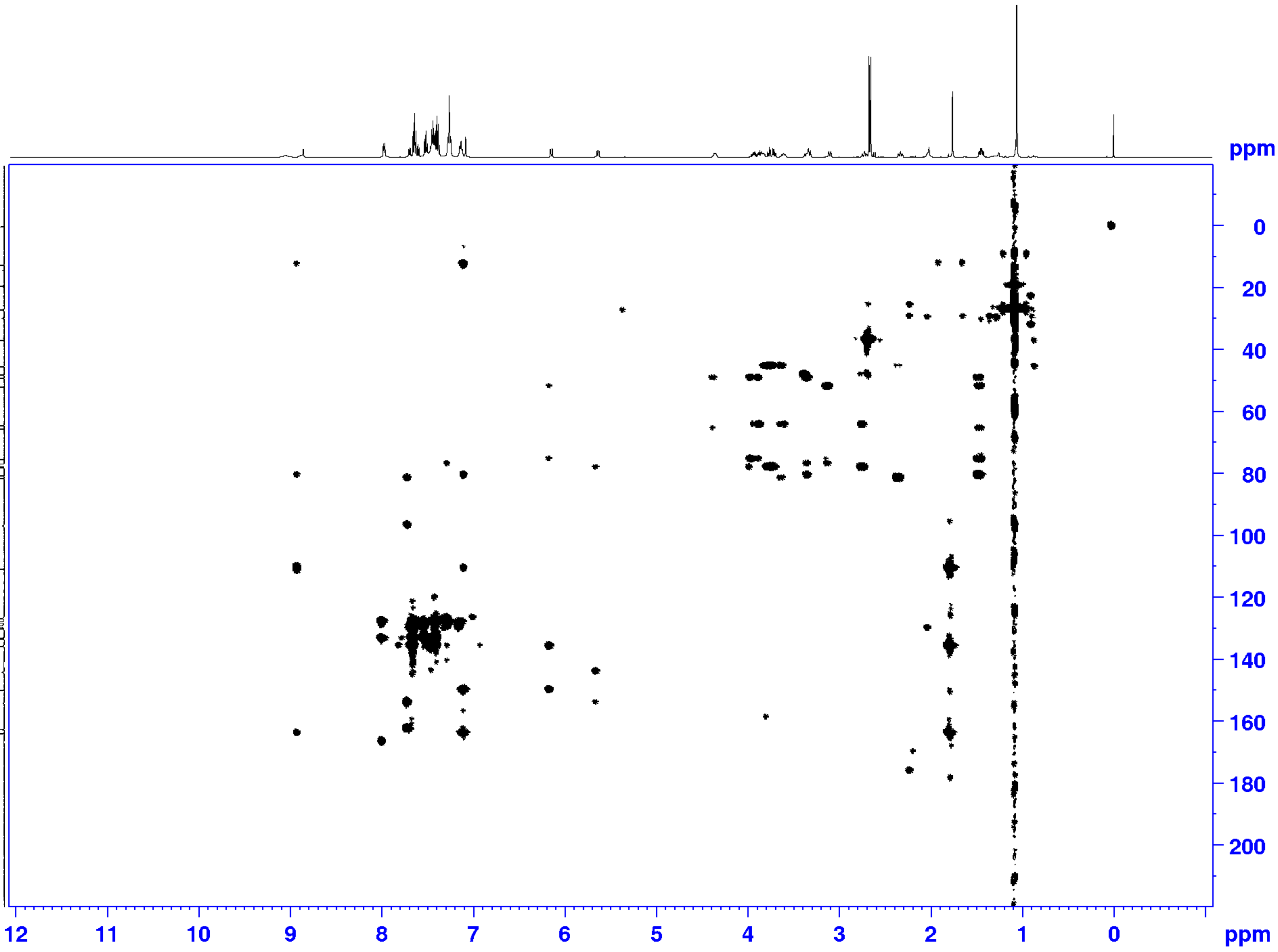

^{1}H NMR (500 MHz, $CDCl_3$) of (*S*p)-**26**

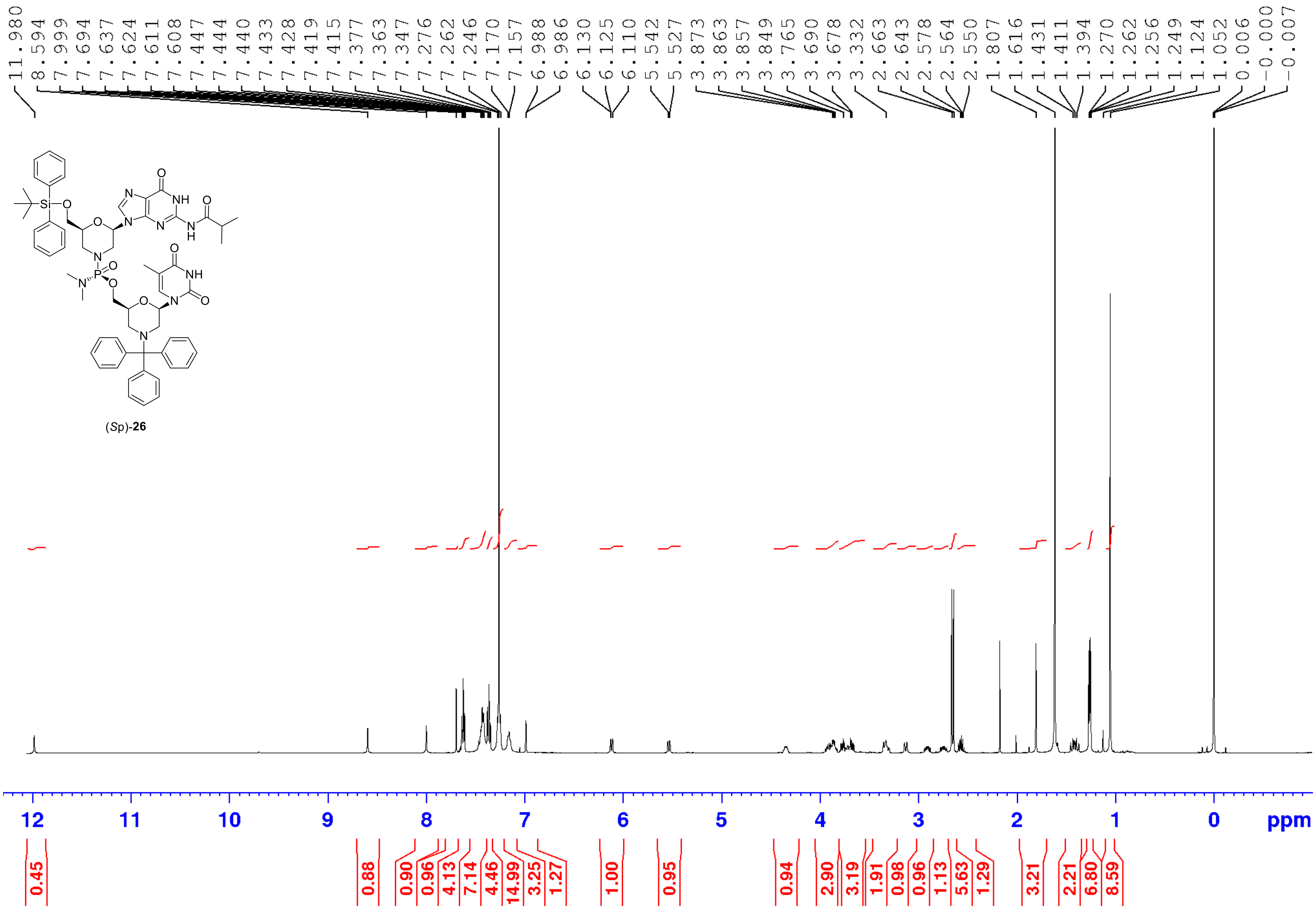

^{13}C {^{1}H} NMR (126 MHz, $CDCl_3$) of (*S*p)-**26**

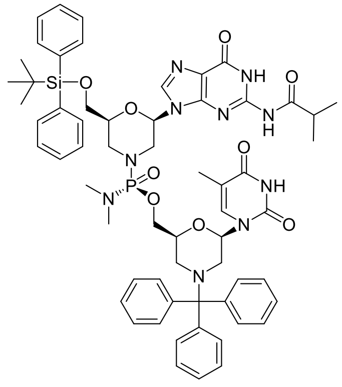

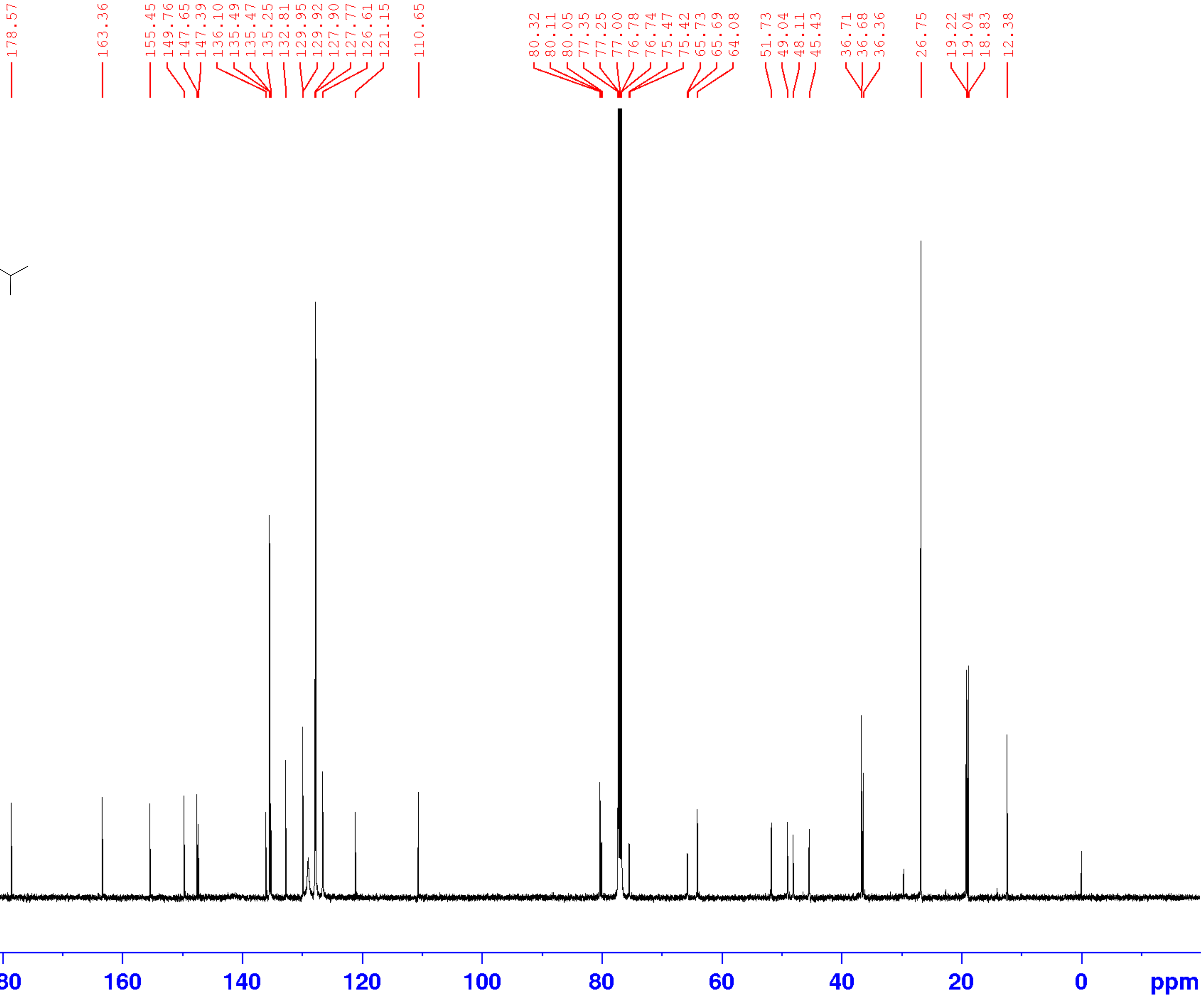

$^{31}P\ \{^{1}H\}$ NMR (202 MHz, $CDCl_3$) of (*S*p)-**26**

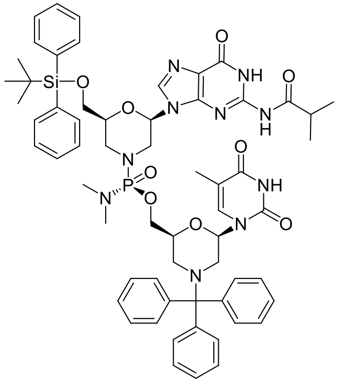

(*S*p)-**26**

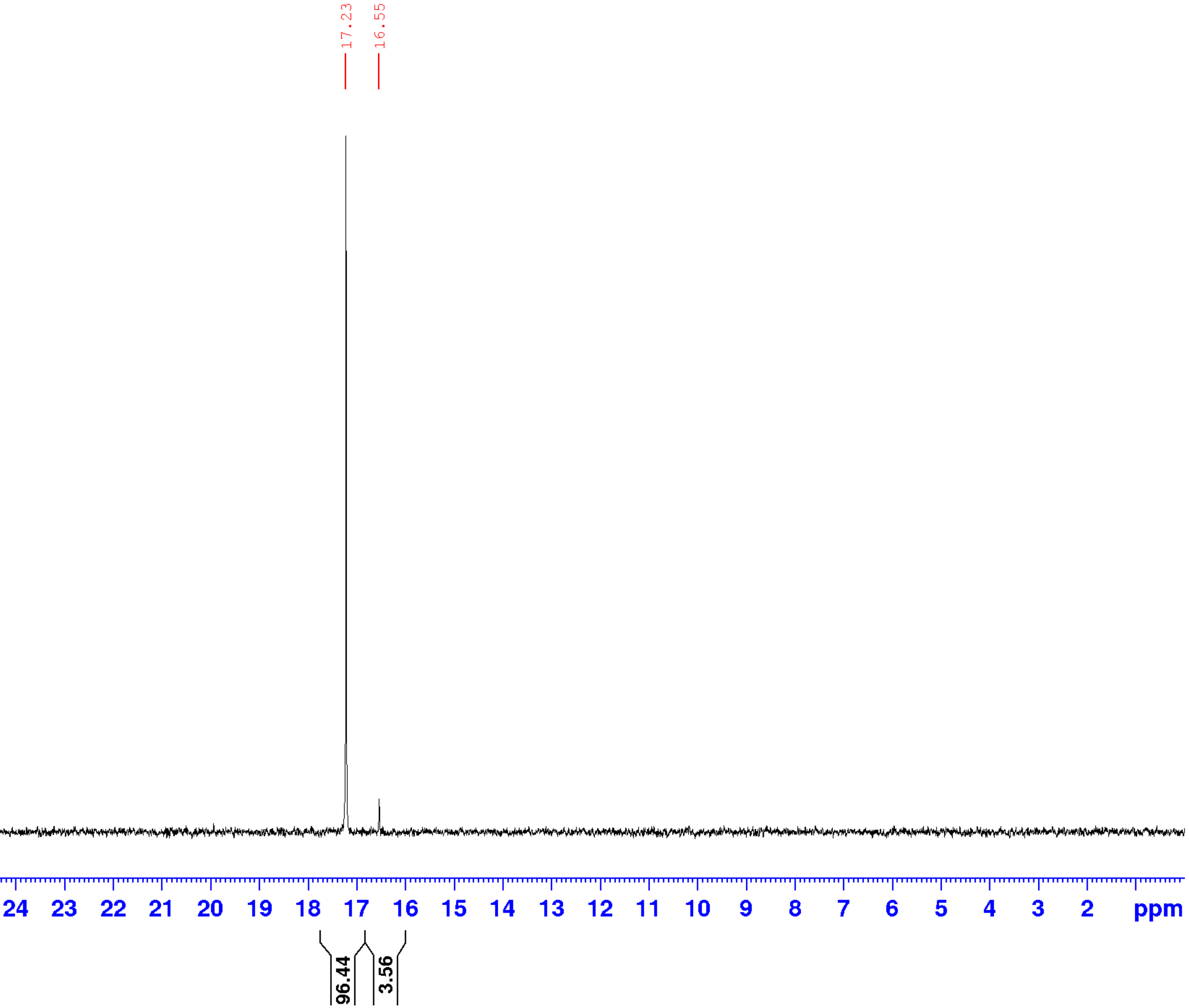

COSY ($CDCl_3$) of (*S*p)-**26**

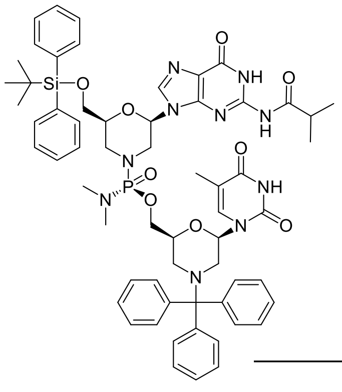

(*S*p)-**26**

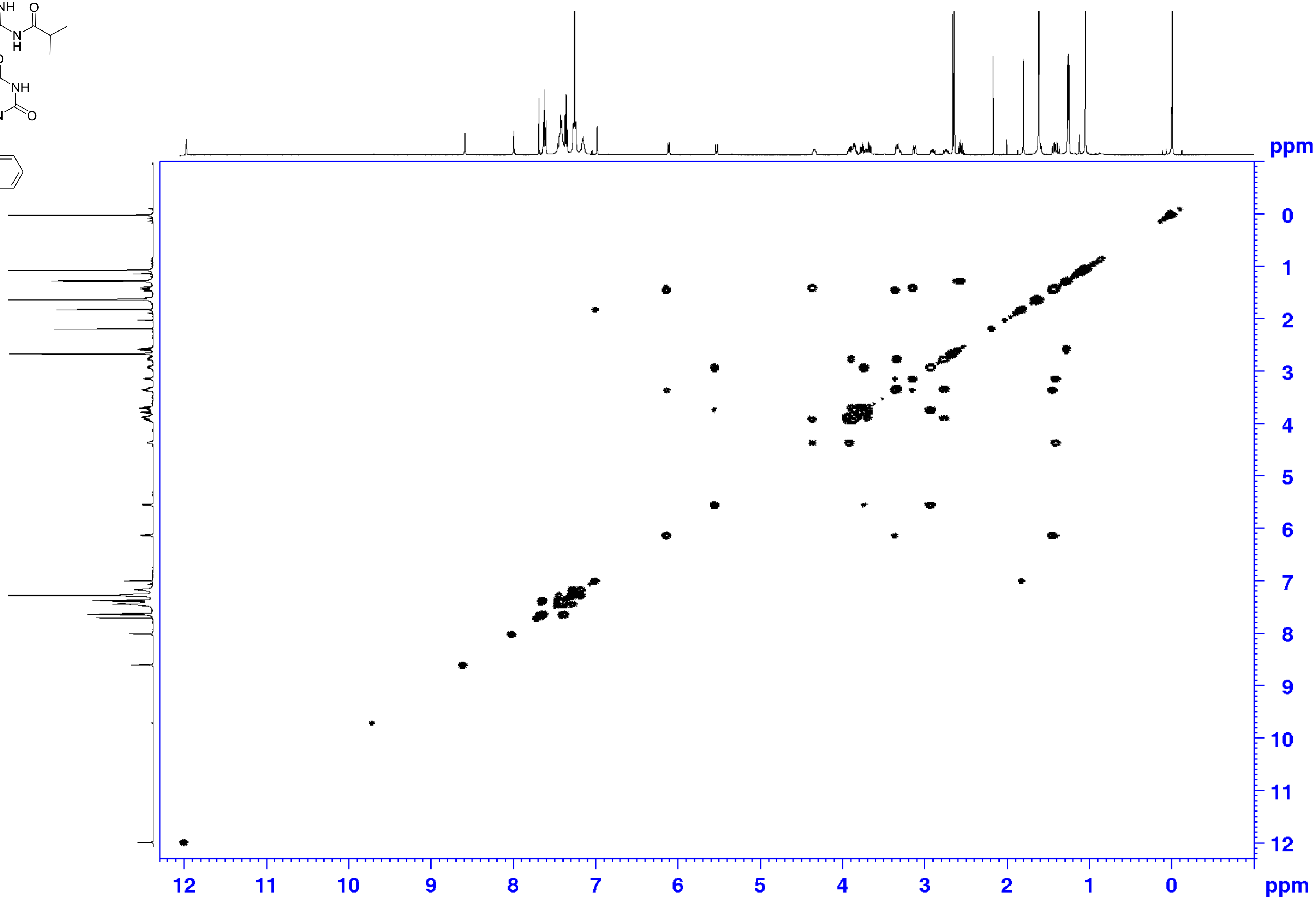

HSQC ($CDCl_3$) of (*S*p)-**26**

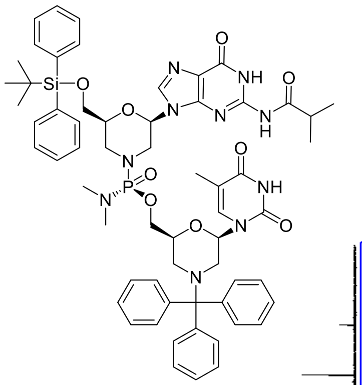

(*S*p)-**26**

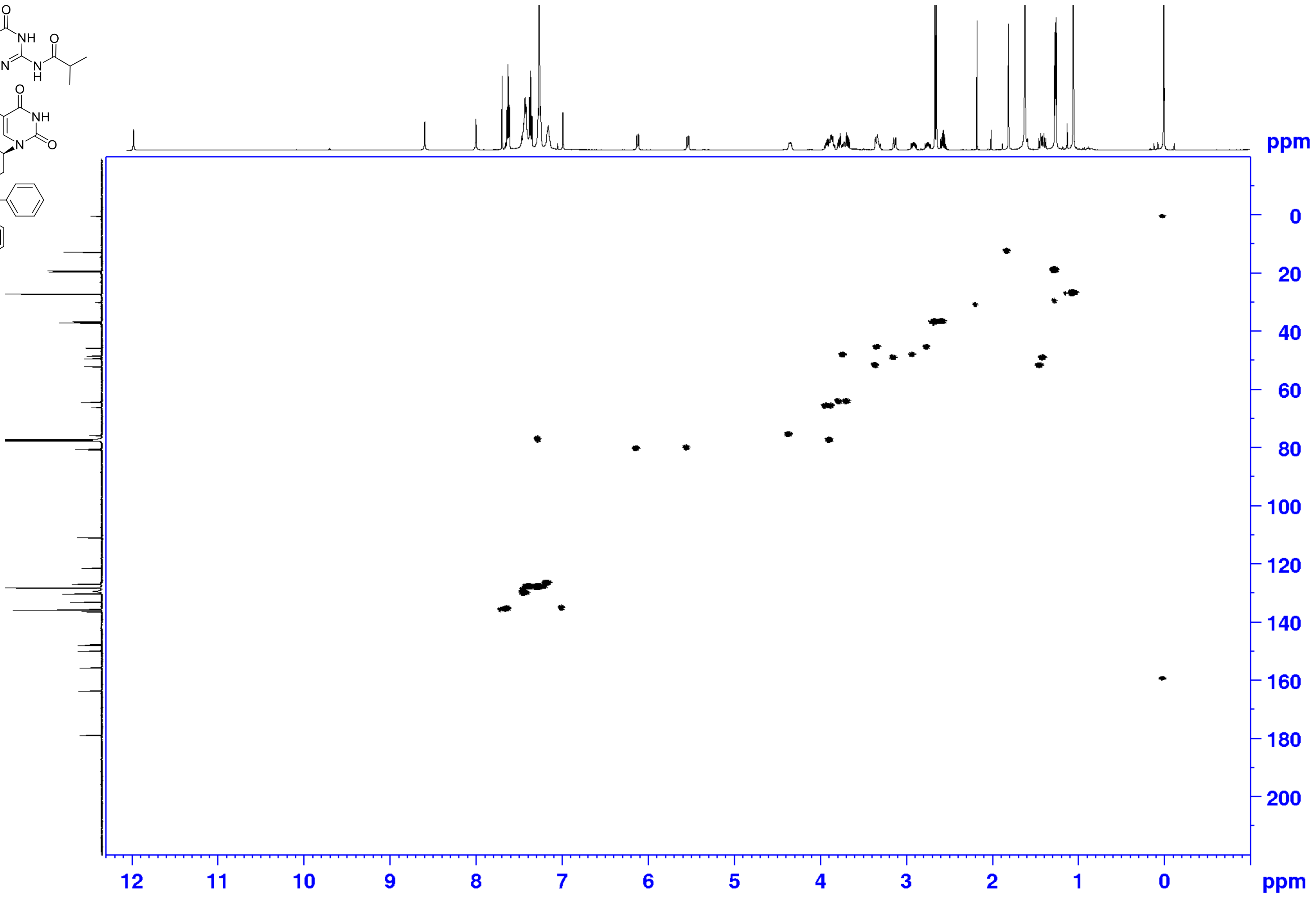

HMBC ($CDCl_3$) of (*S*p)-**26**

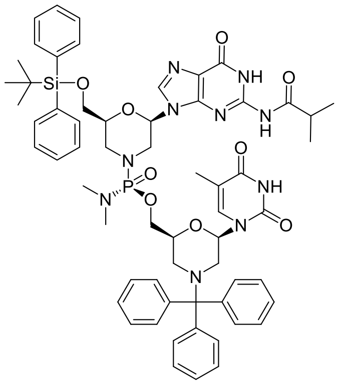

(*S*p)-**26**

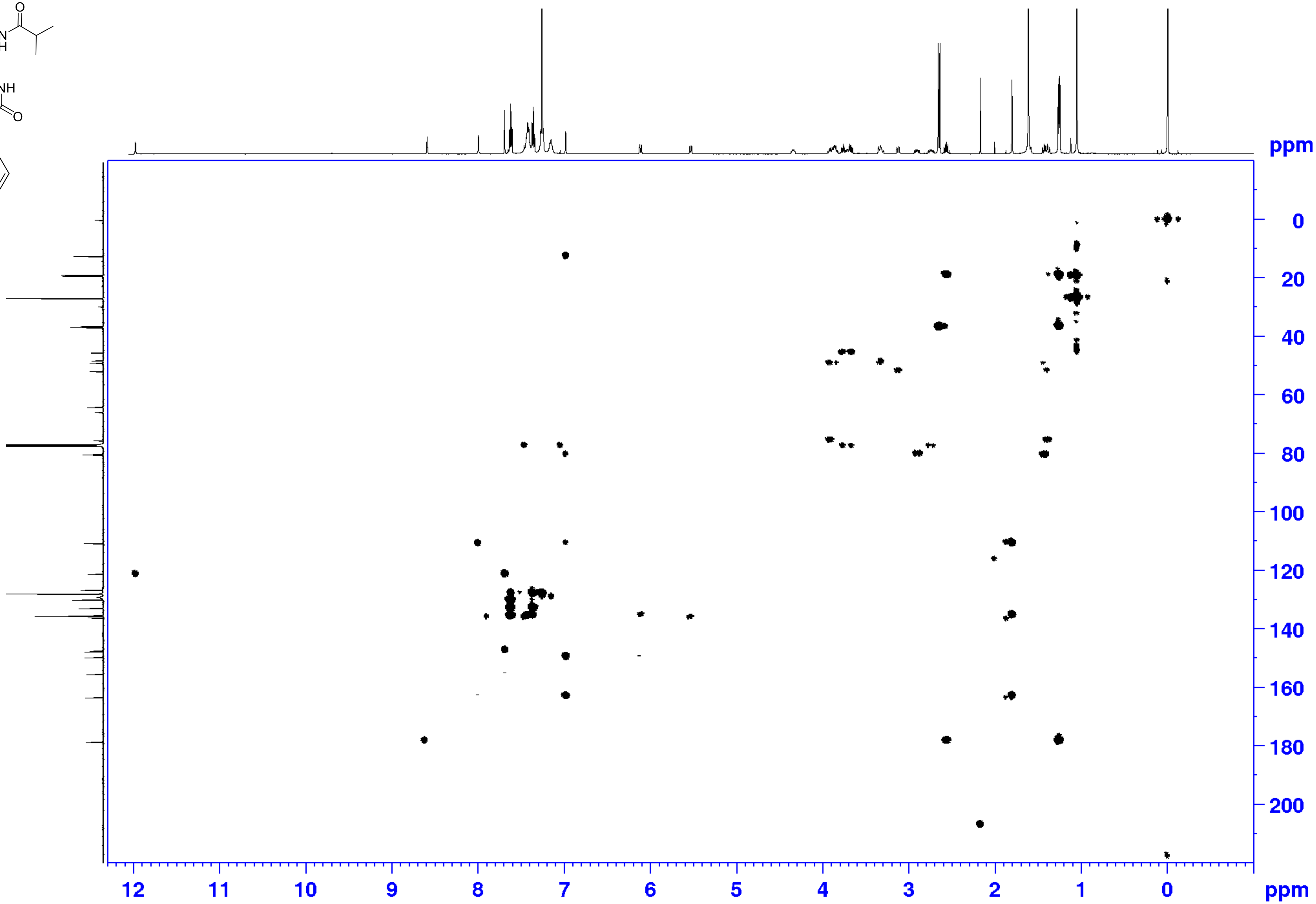

^{1}H NMR (500 MHz, $CDCl_3$) of (*R*p)-**26**

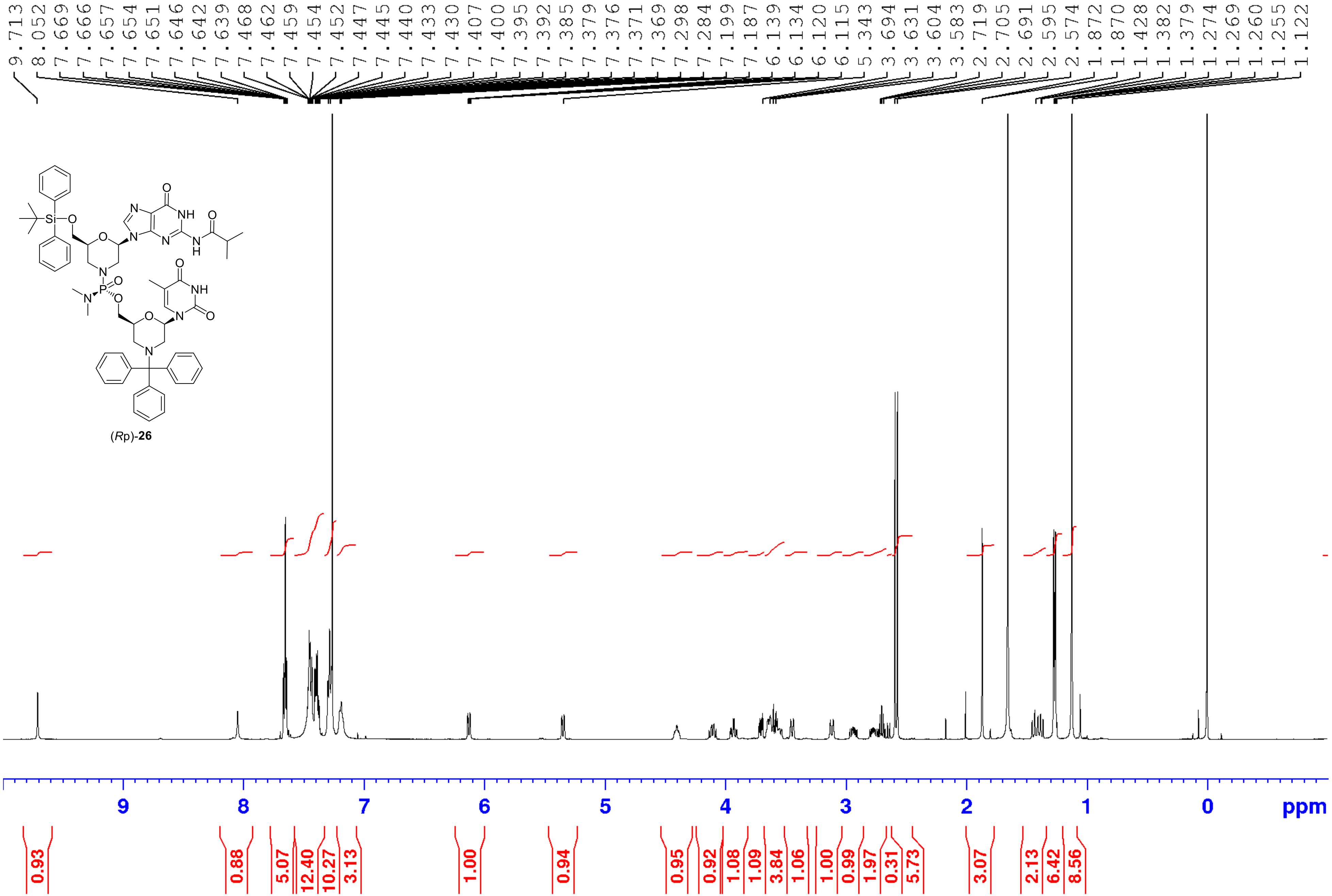

^{13}C {^{1}H} NMR (126 MHz, $CDCl_3$) of (*R*p)-**26**

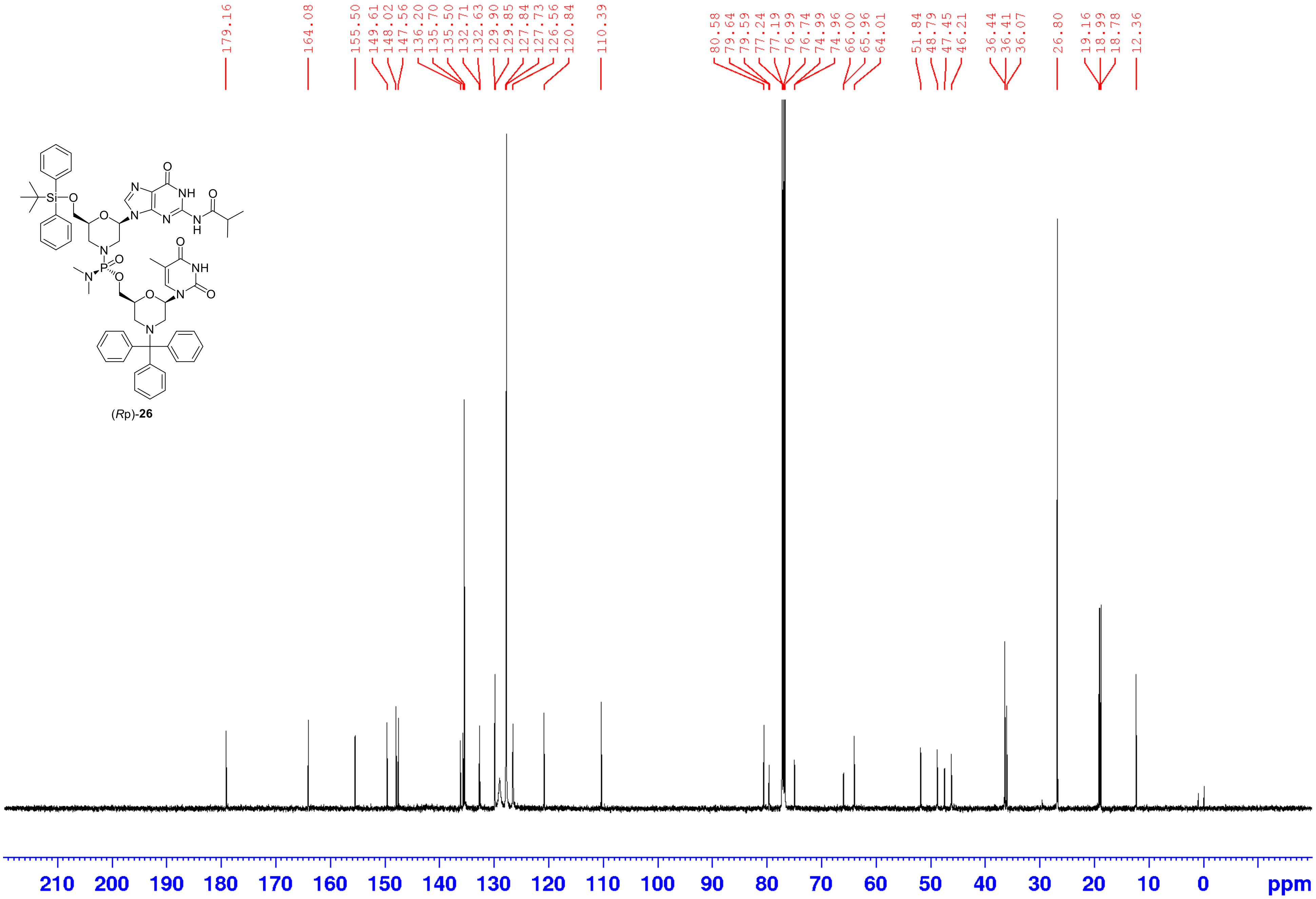

^{31}P {^{1}H} NMR (202 MHz, $CDCl_3$) of (*R*p)-**26**

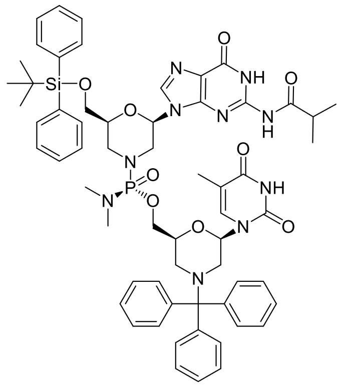

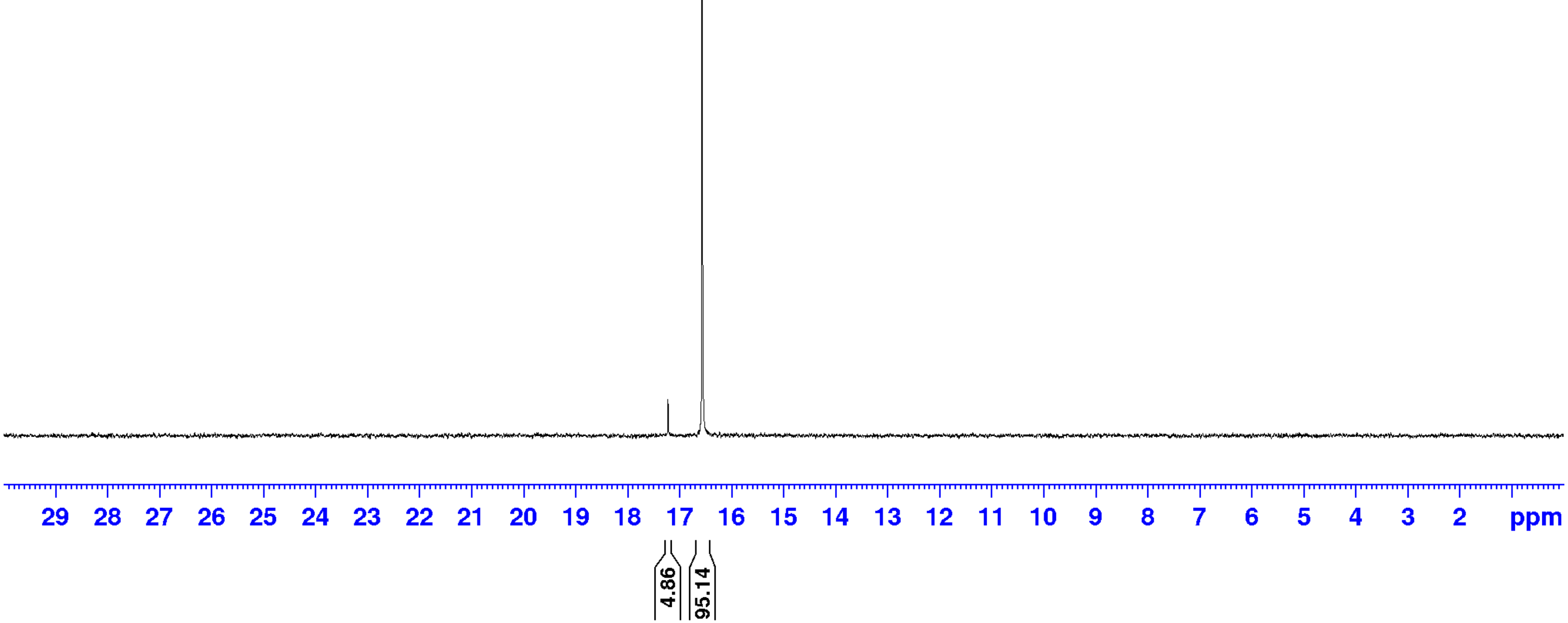

COSY ($CDCl_3$) of (*R*p)-**26**

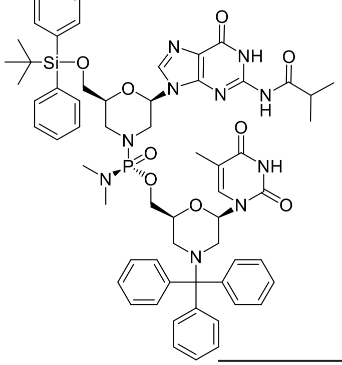

(*R*p)-**26**

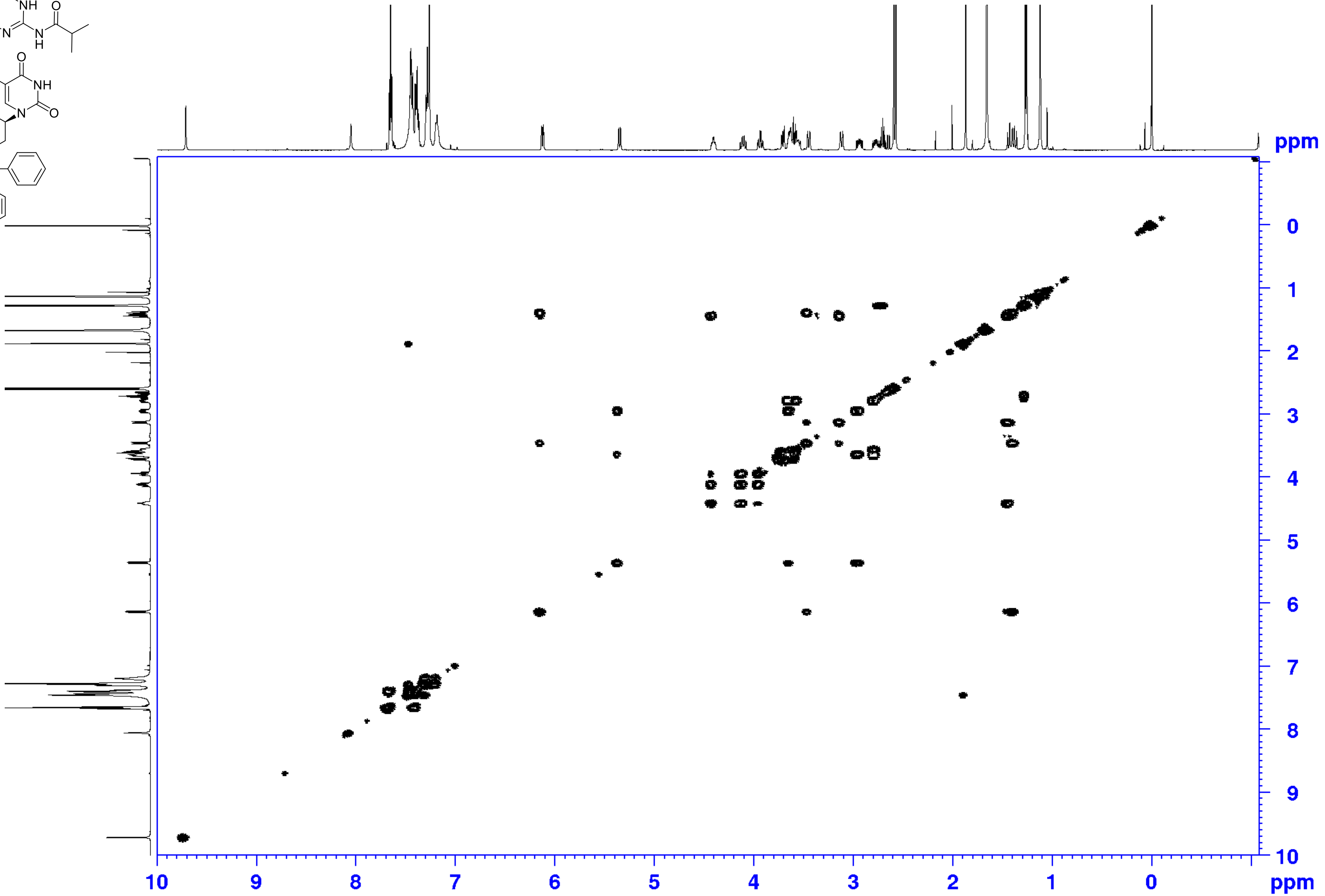

HSQC ($CDCl_3$) of (*R*p)-**26**

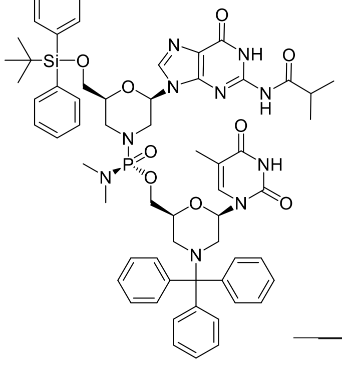

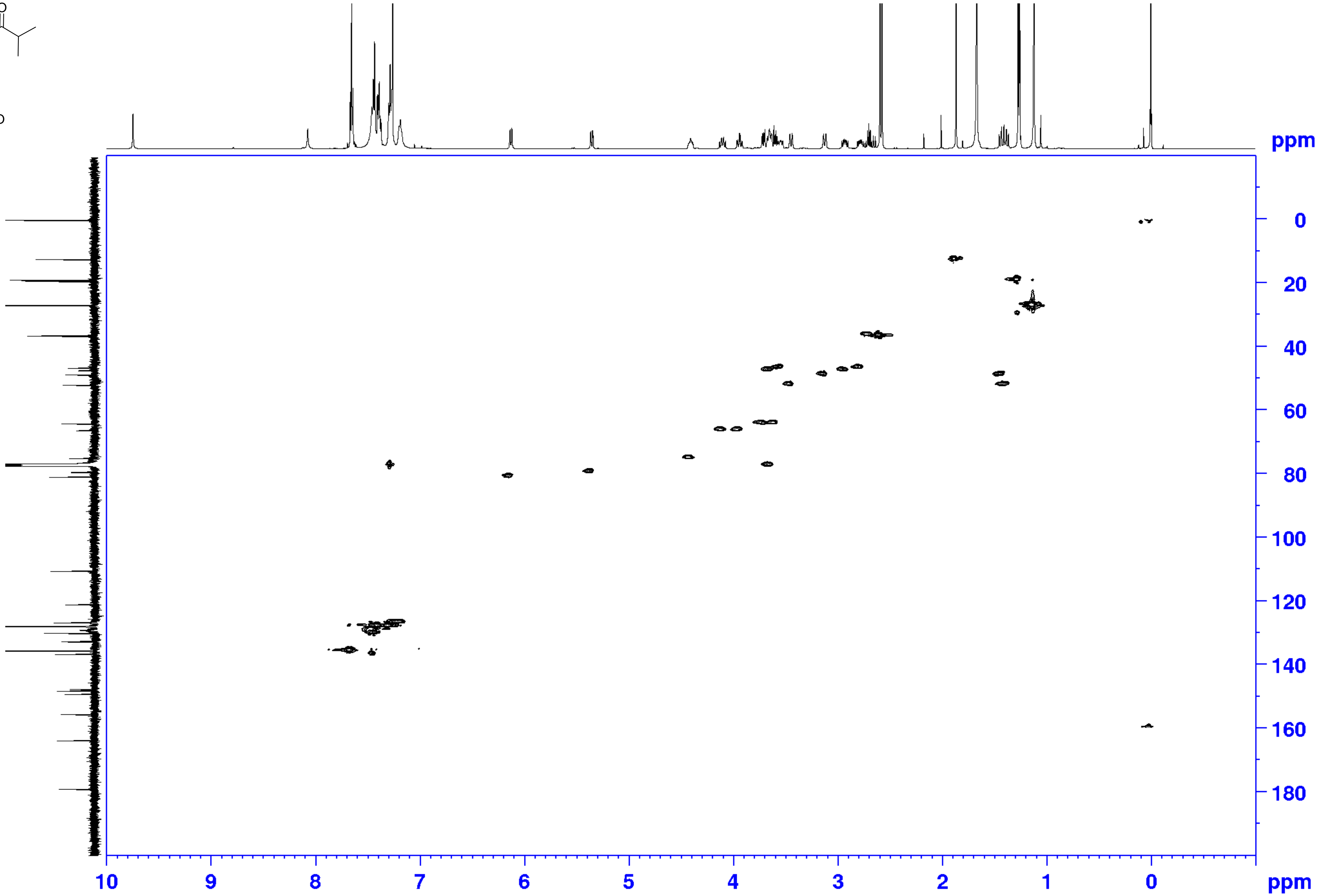

HMBC (CDCl$_3$) of (*R*p)-**26**

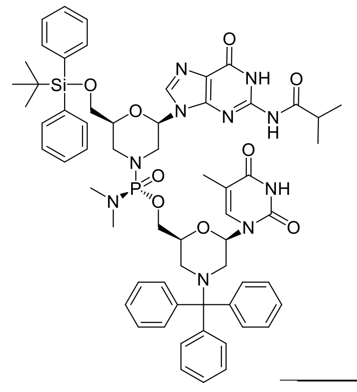

(*R*p)-**26**

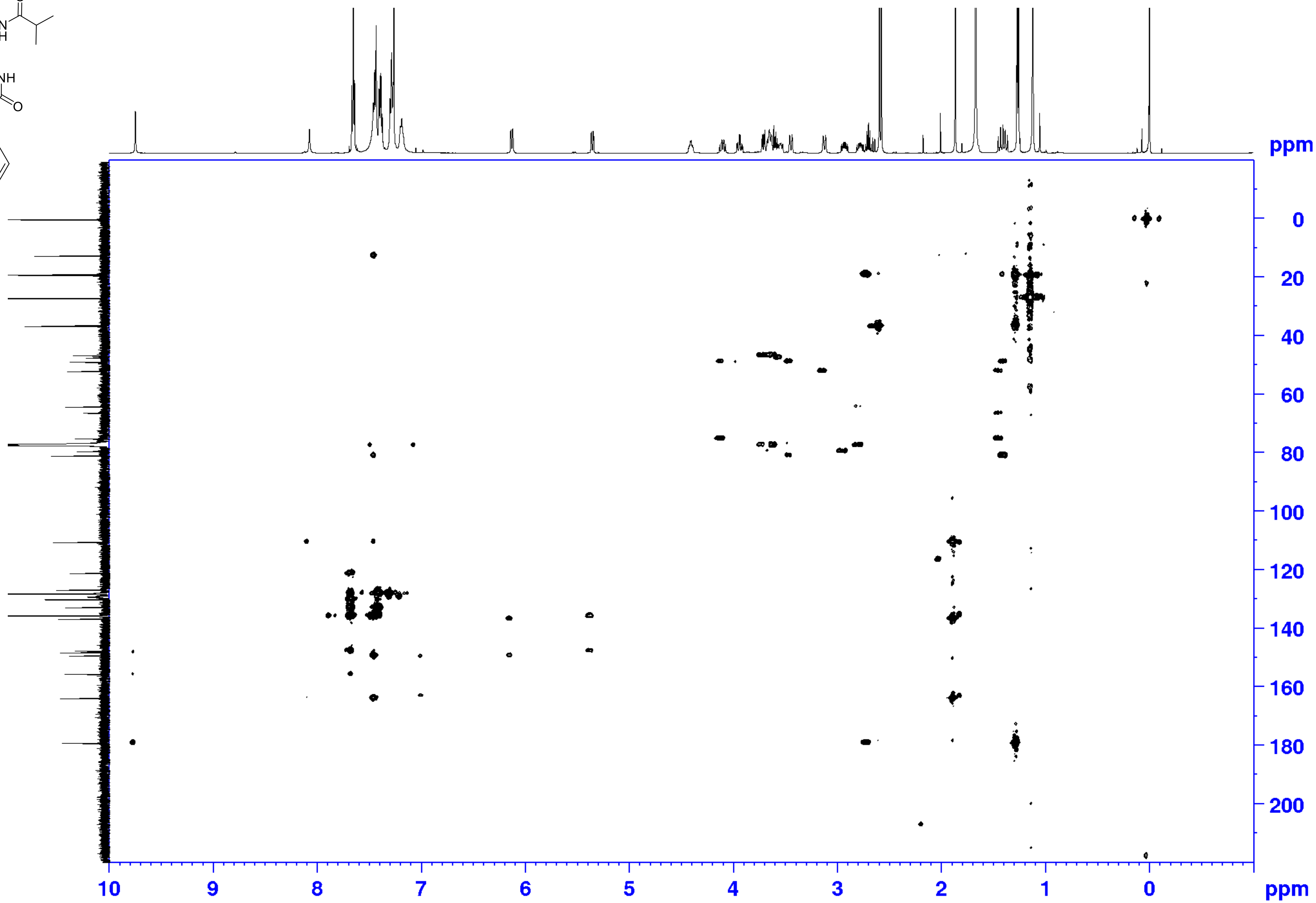

^{1}H NMR (600 MHz, D_2O) of (*S*p)-**27**

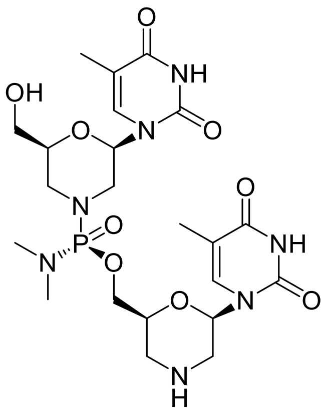

(*S*p)-**27**

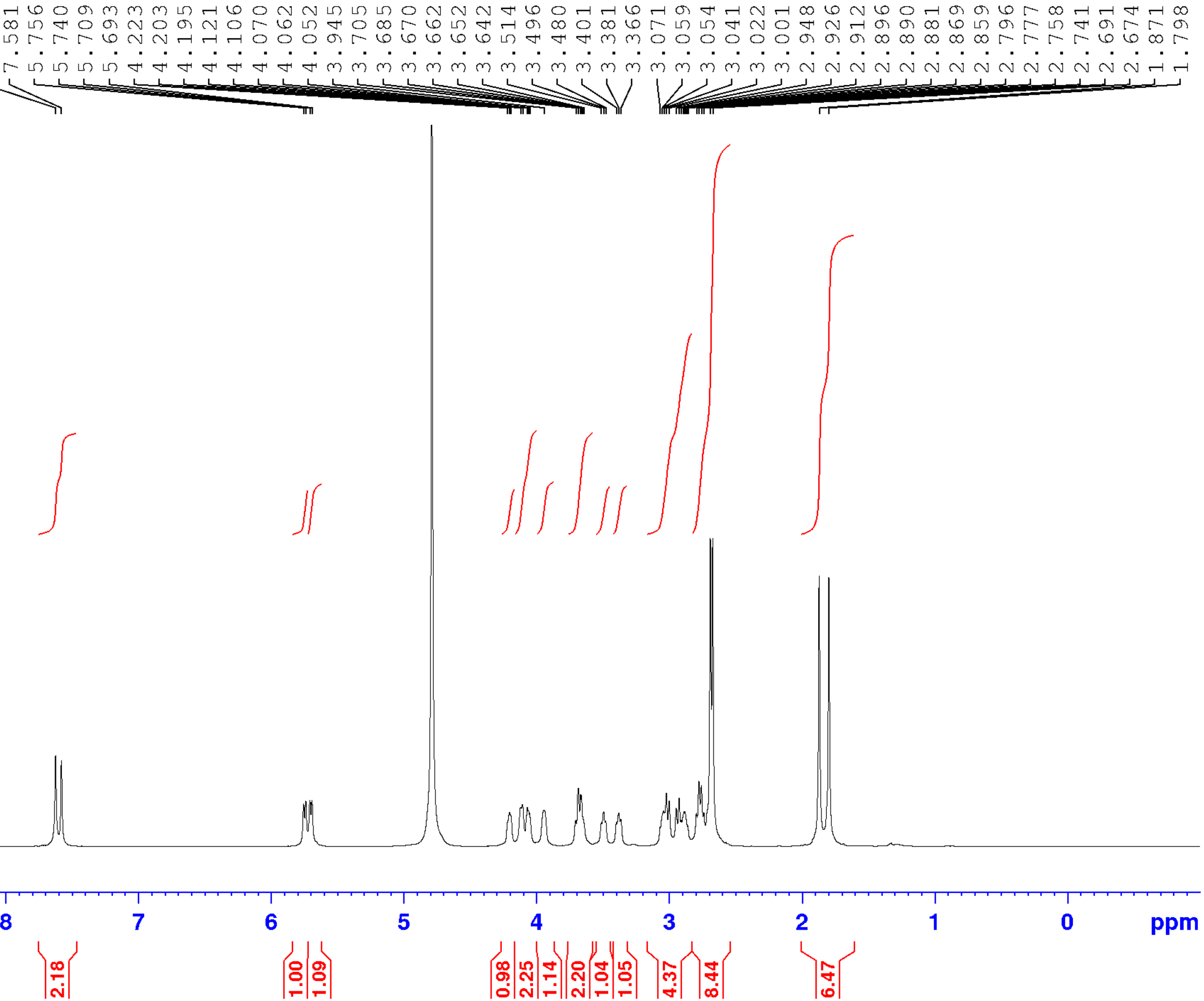

^{13}C {^{1}H} NMR (126 MHz, D_2O) of (*S*p)-**27**

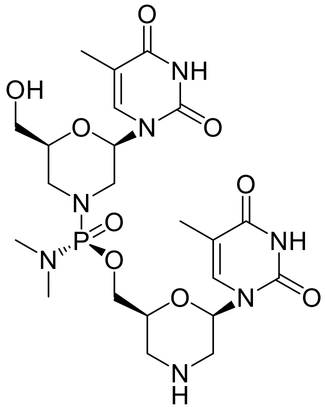

(*S*p)-**27**

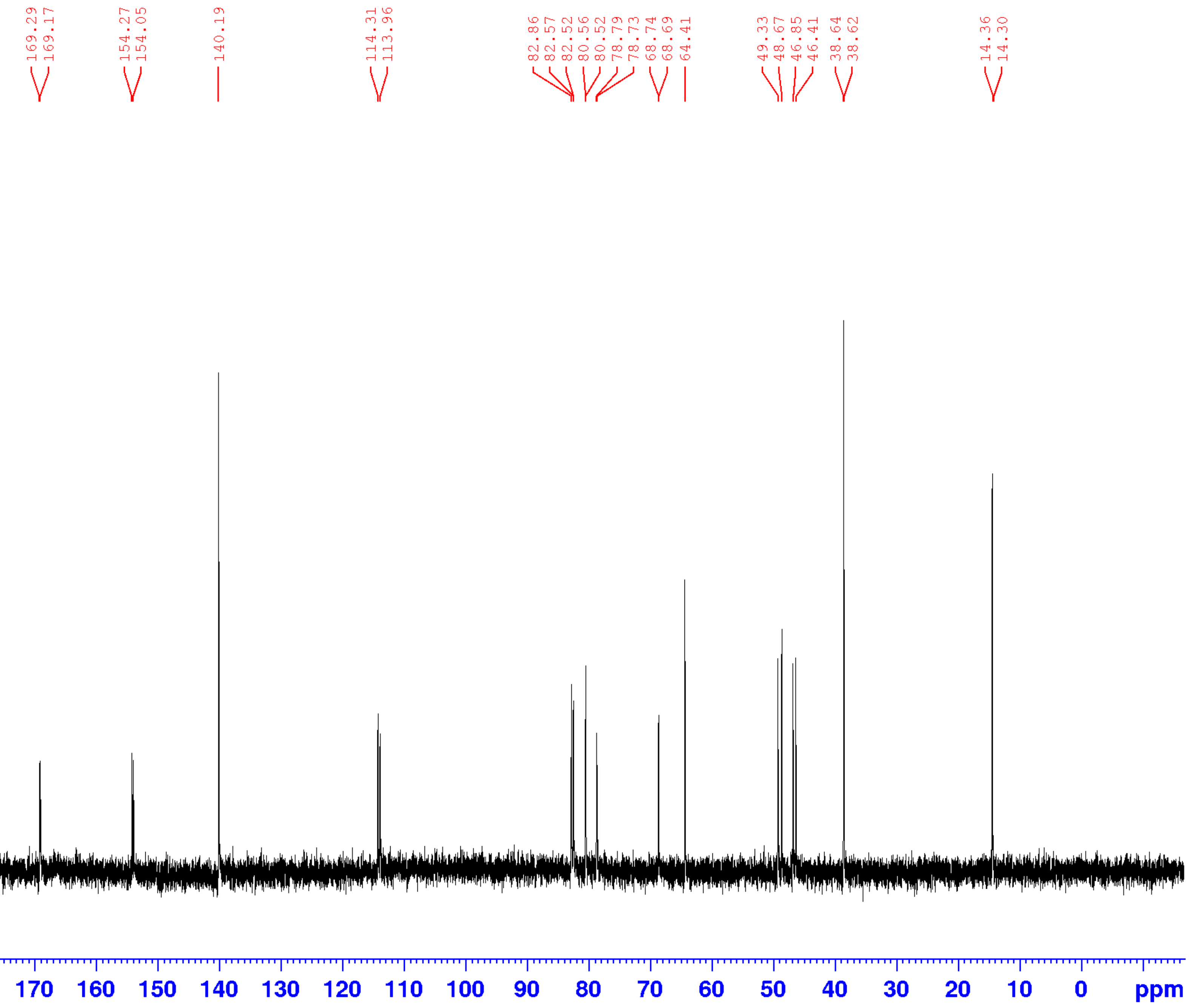

^{31}P {^{1}H} NMR (202 MHz, D_2O) of (*S*p)-**27**

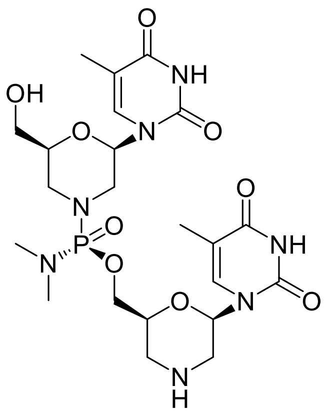

(*S*p)-**27**

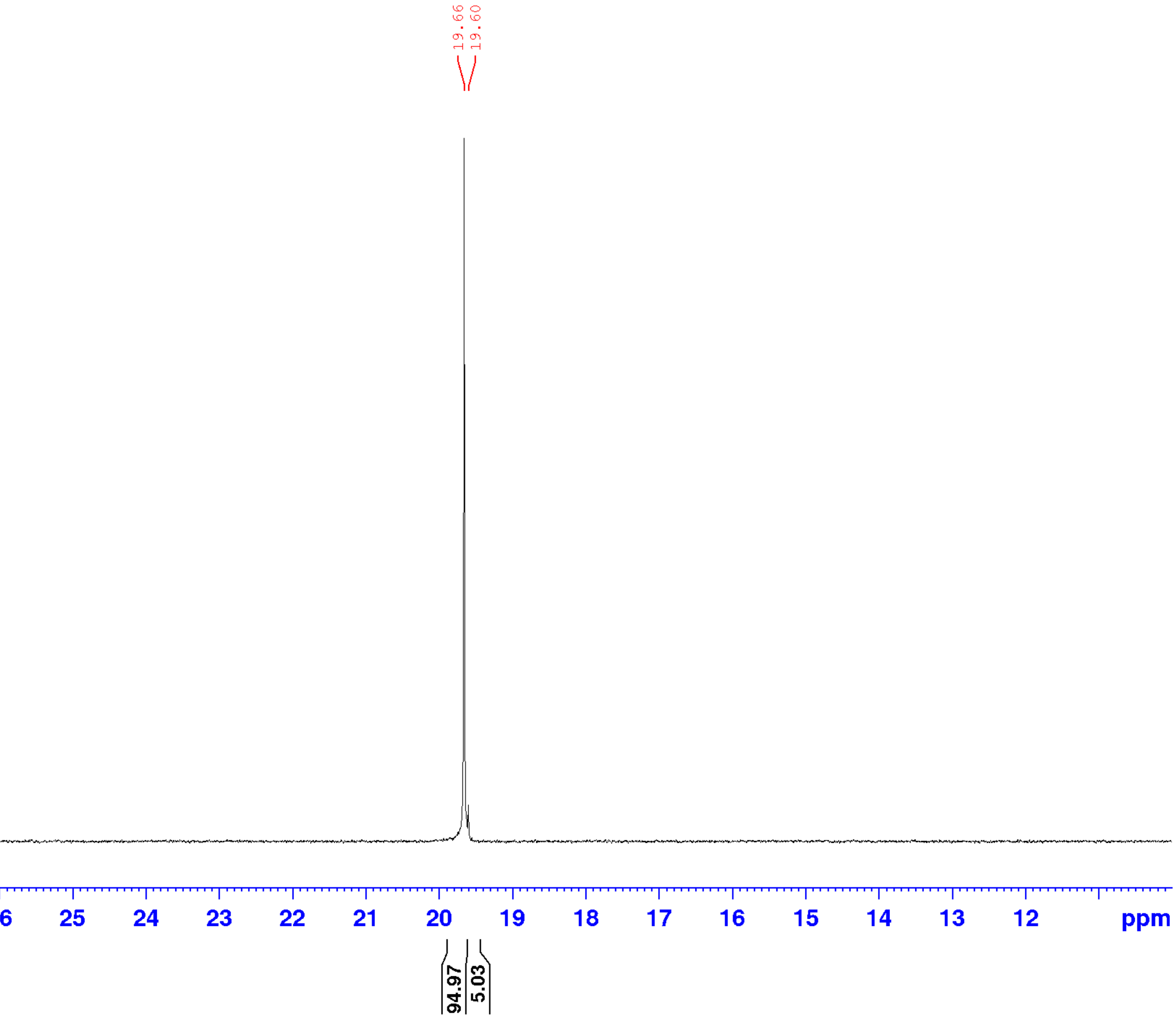

COSY (D_2O) of (*S*p)-**27**

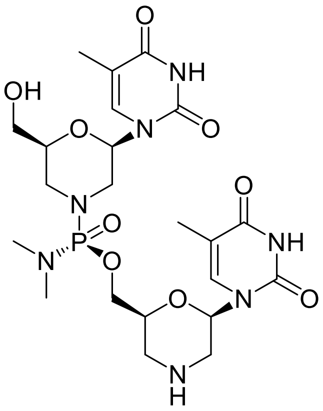

(*S*p)-**27**

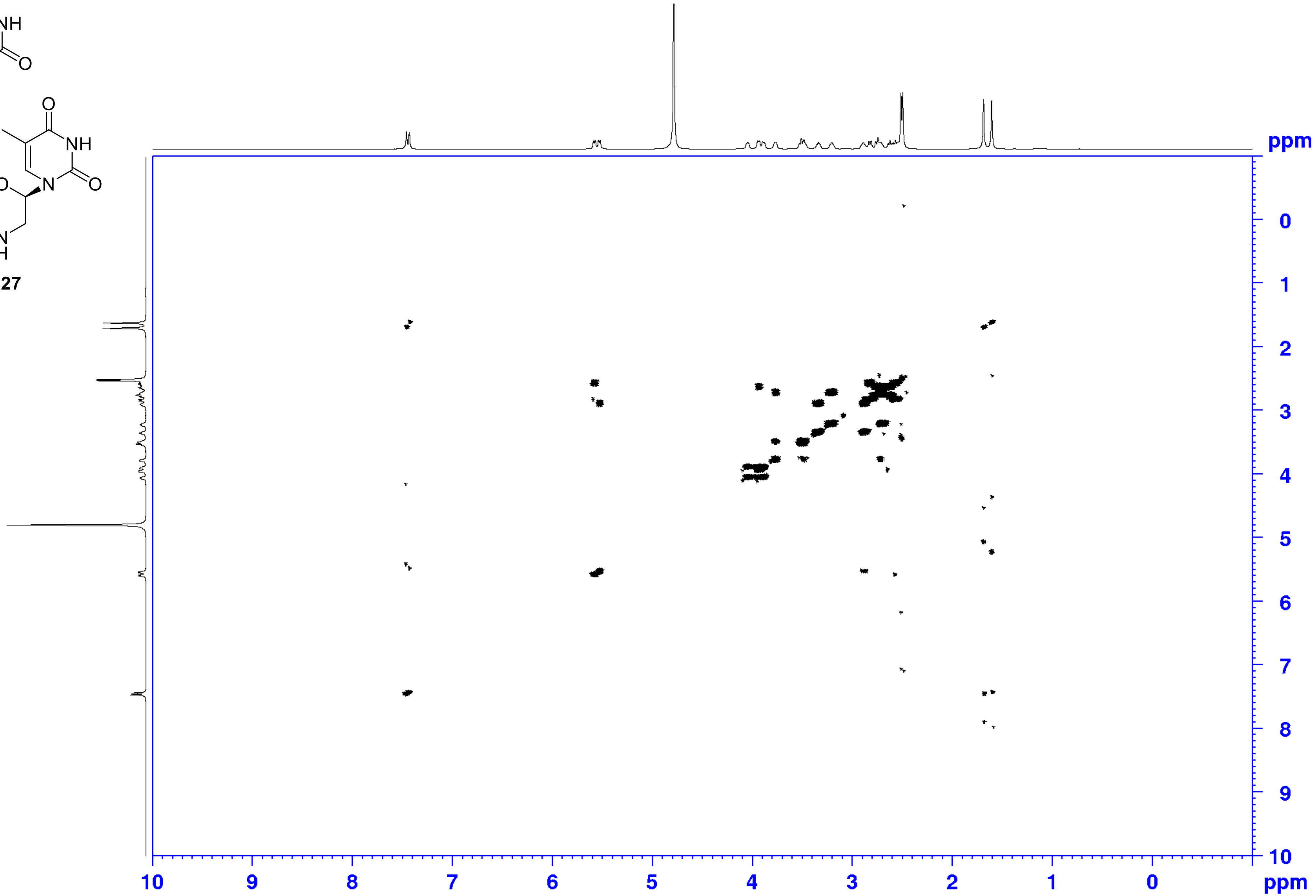

HSQC (D_2O) of (*S*p)-**27**

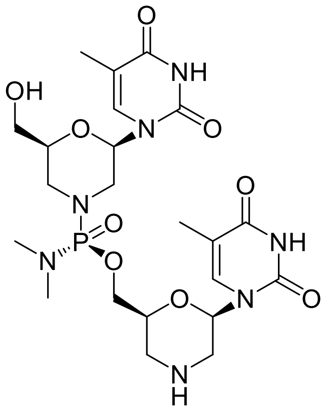

(*S*p)-**27**

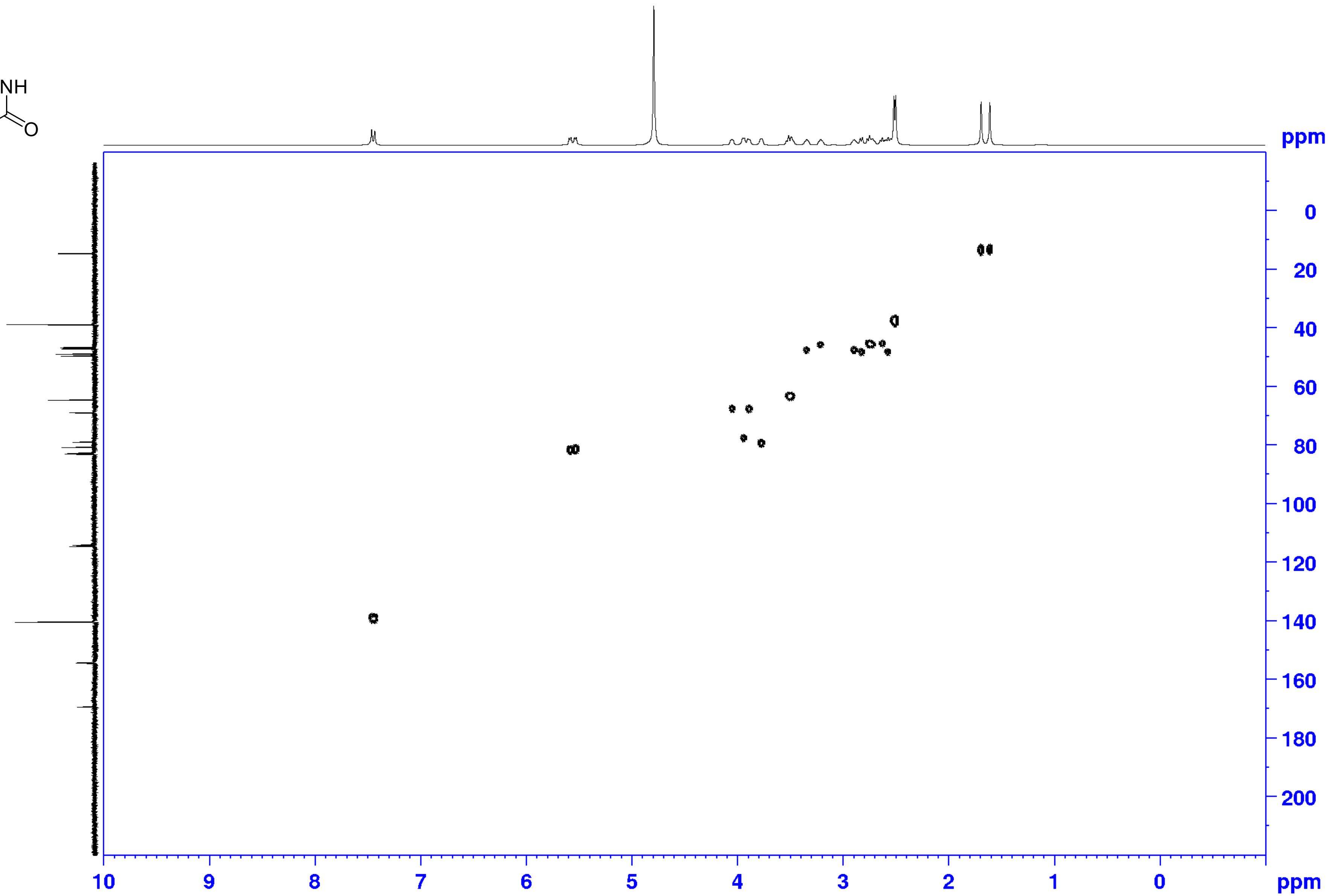

HMBC (D_2O) of (*S*p)-**27**

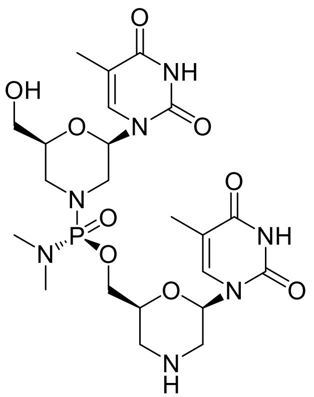

(*S*p)-**27**

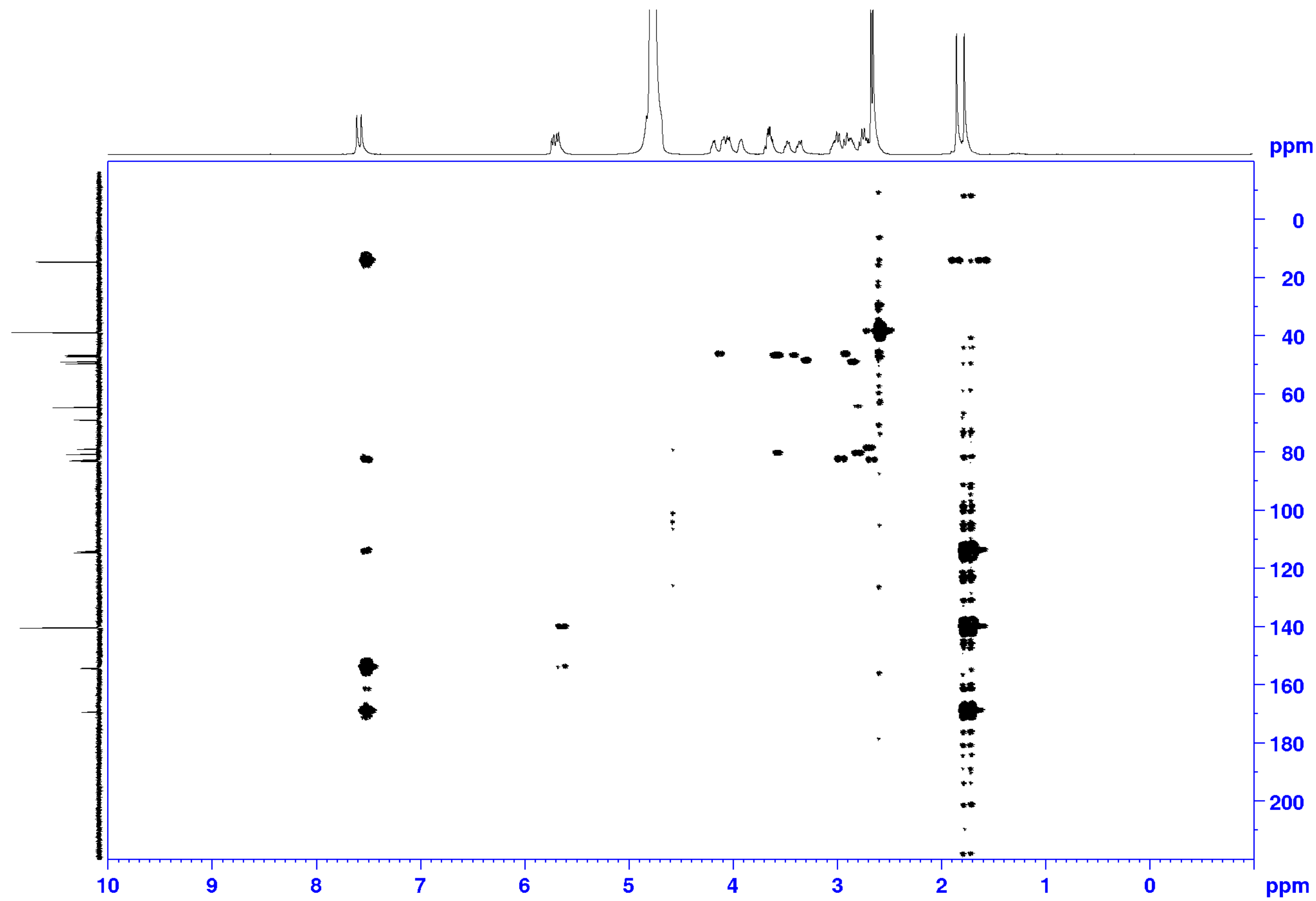

^{1}H NMR (500 MHz, D_2O) of (*R*p)-**27**

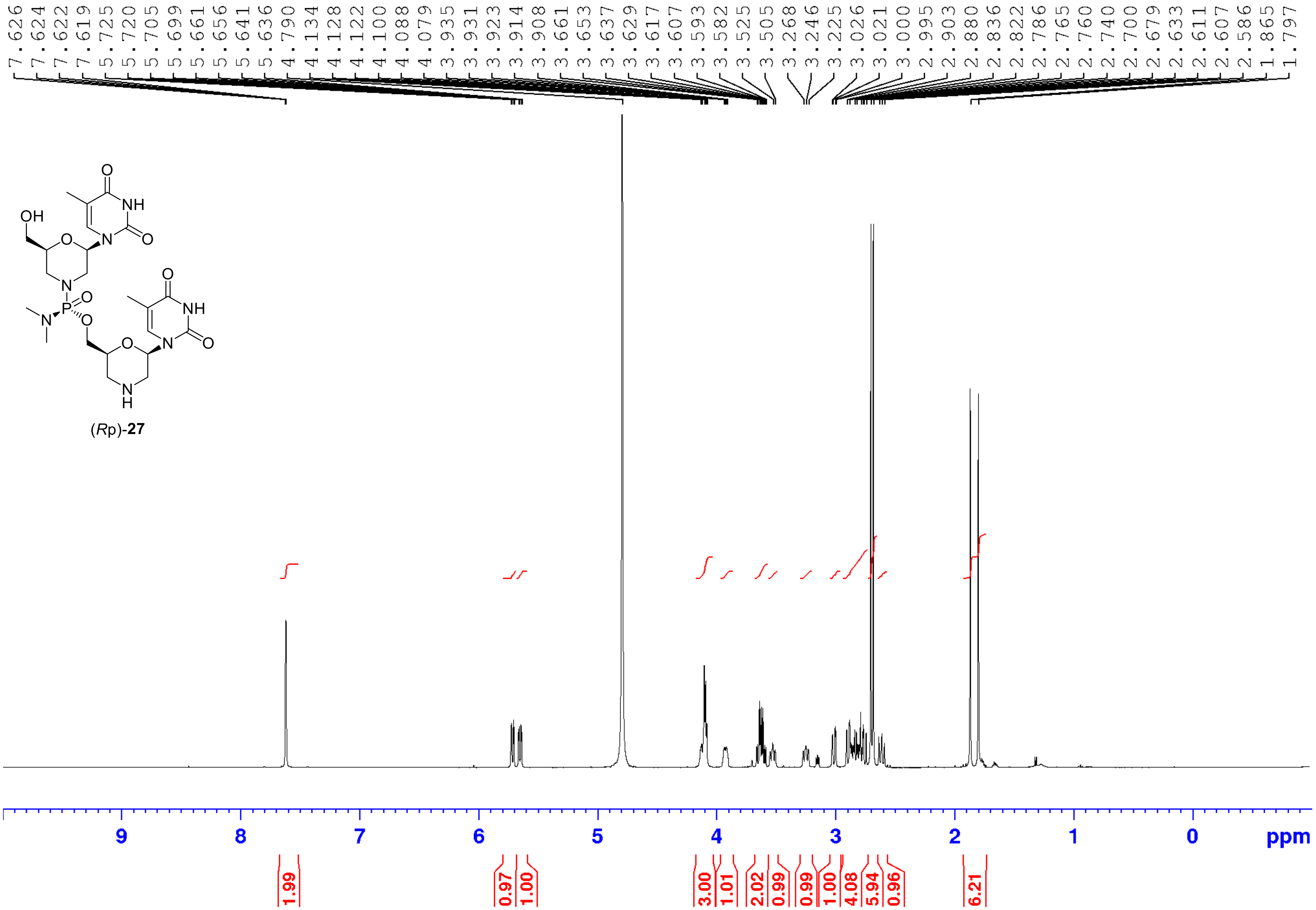

^{13}C {^{1}H} NMR (126 MHz, D_2O) of (*R*p)-**27**

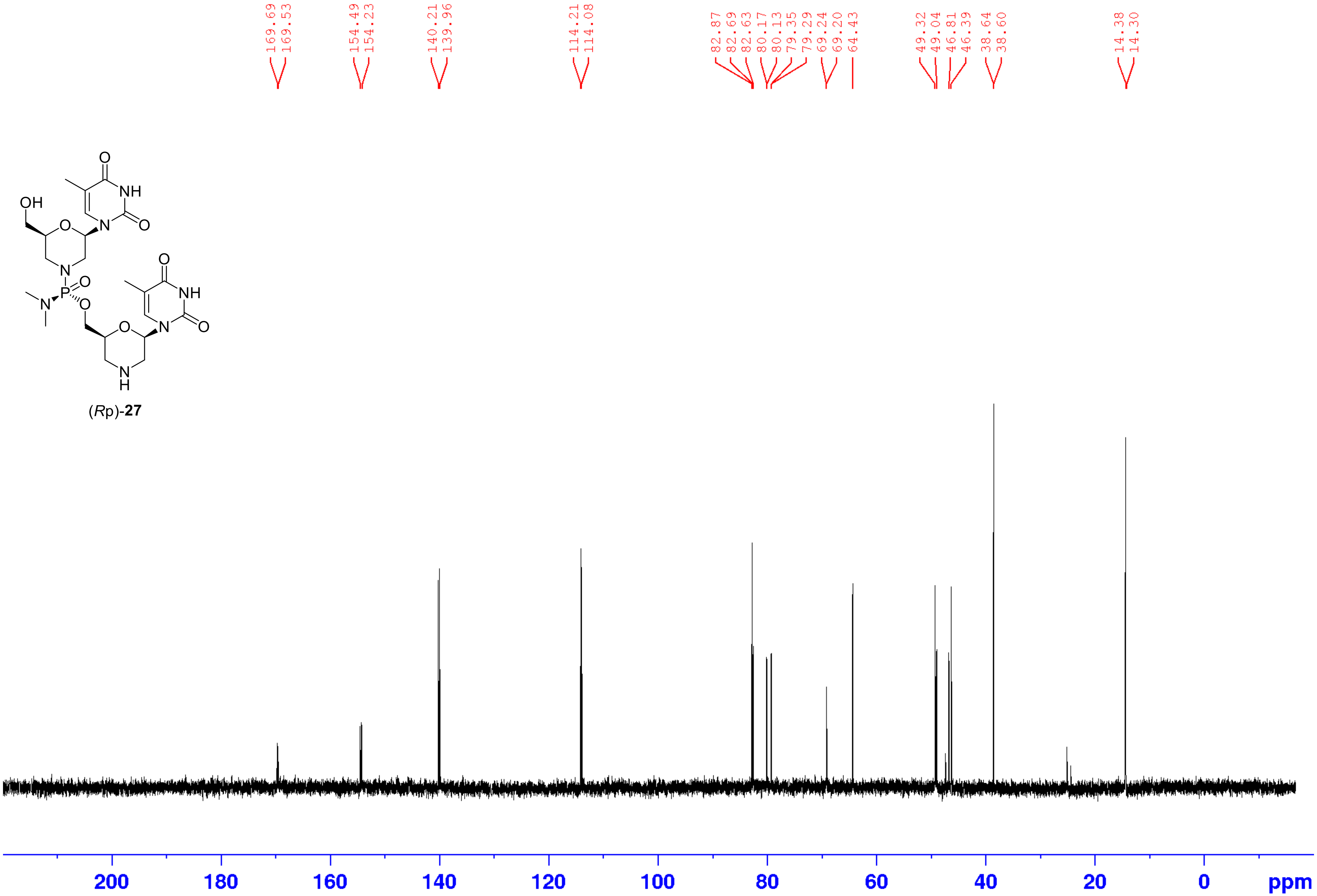

^{31}P {^{1}H} NMR (202 MHz, D_2O) of (*R*p)-**27**

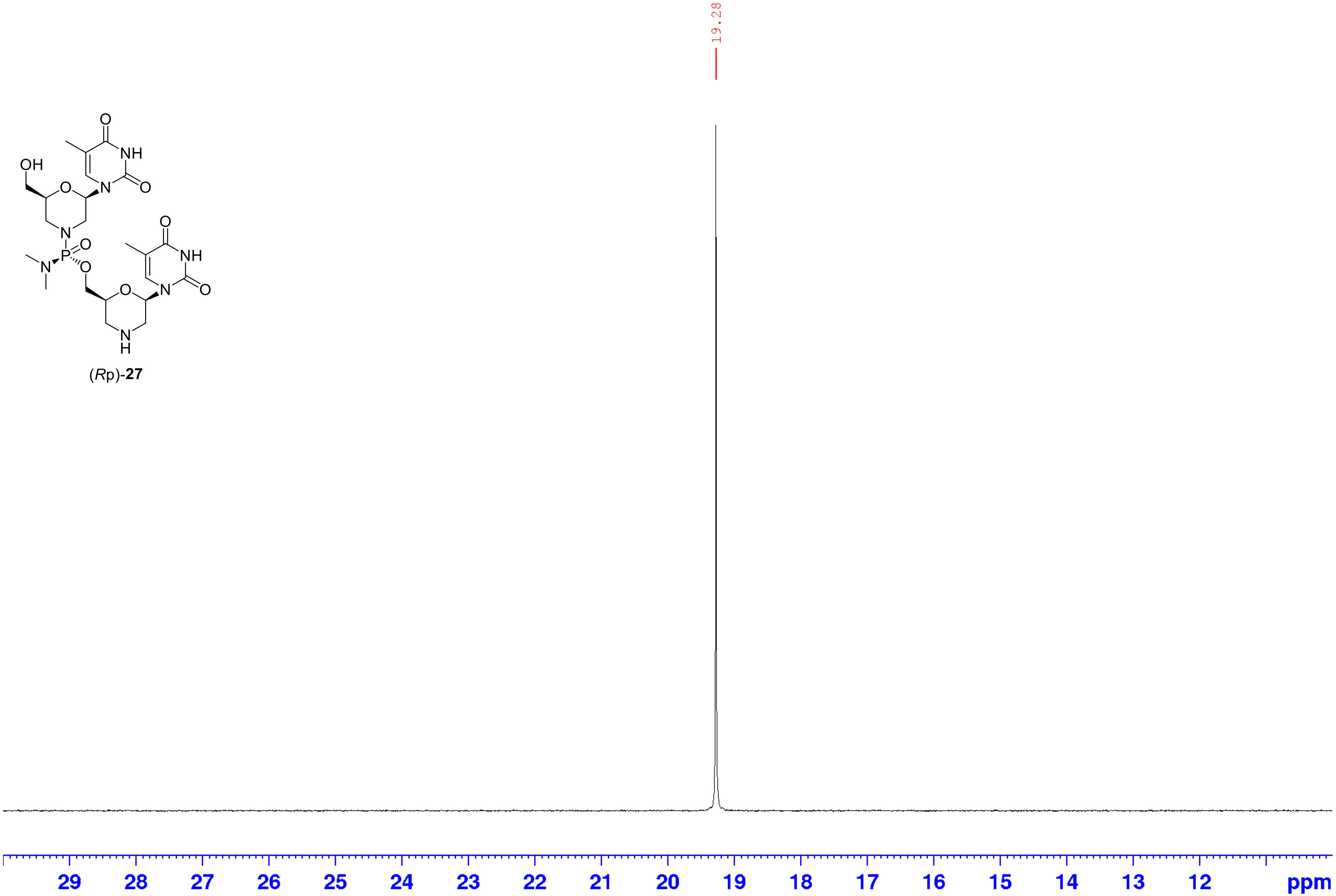

(*R*p)-**27**

COSY (D_2O) of (*R*p)-**27**

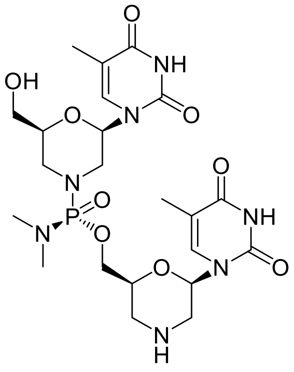

(*R*p)-**27**

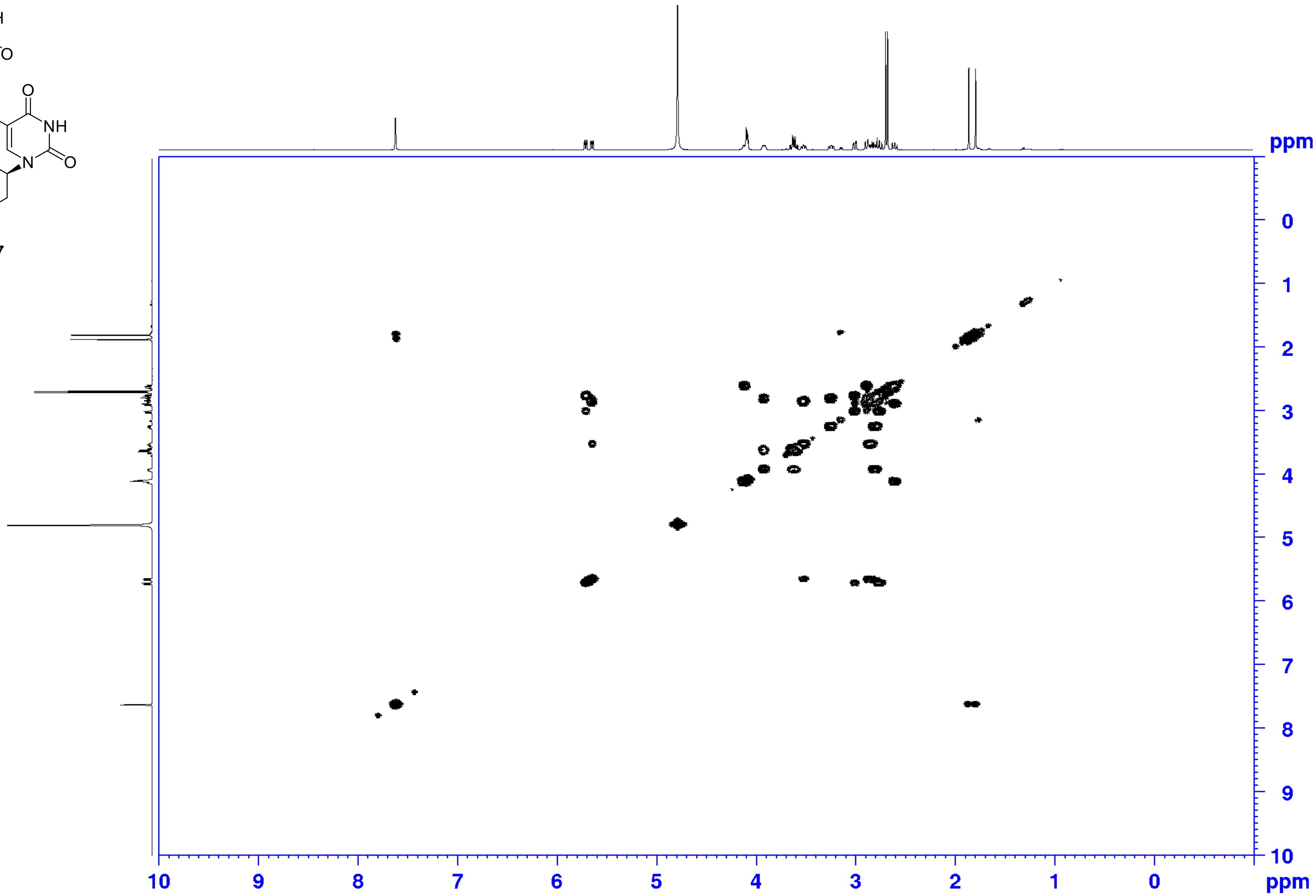

HSQC (D_2O) of (*R*p)-**27**

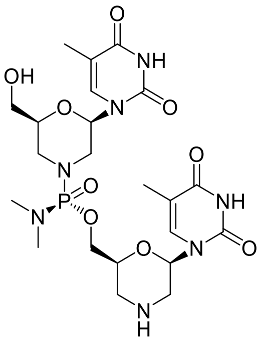

(*R*p)-**27**

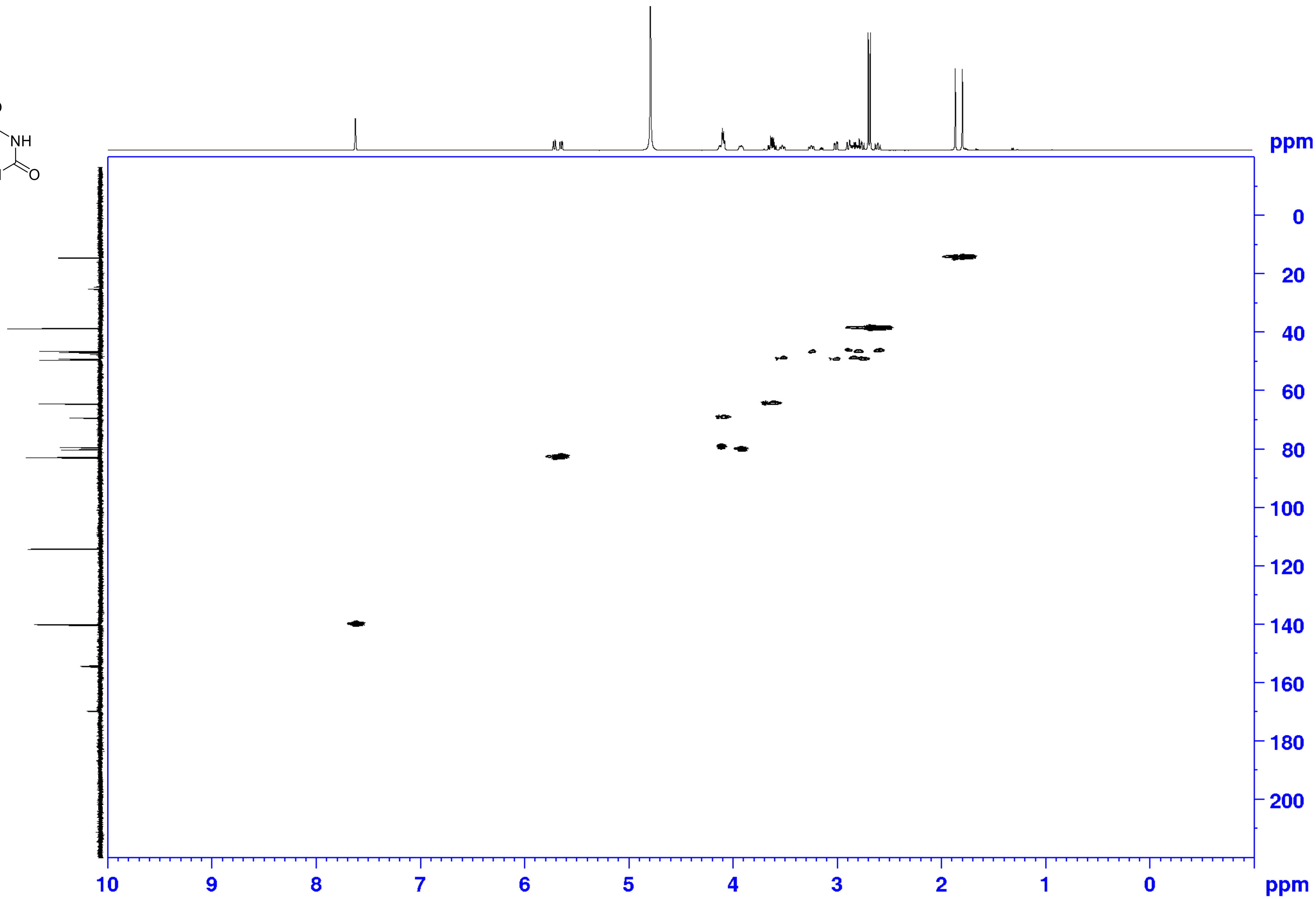

HMBC (D_2O) of (*R*p)-**27**

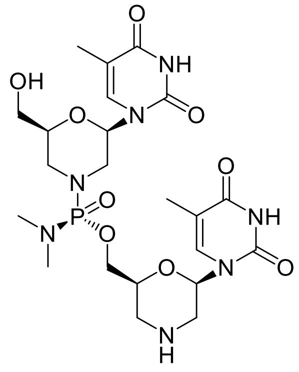

(*R*p)-**27**

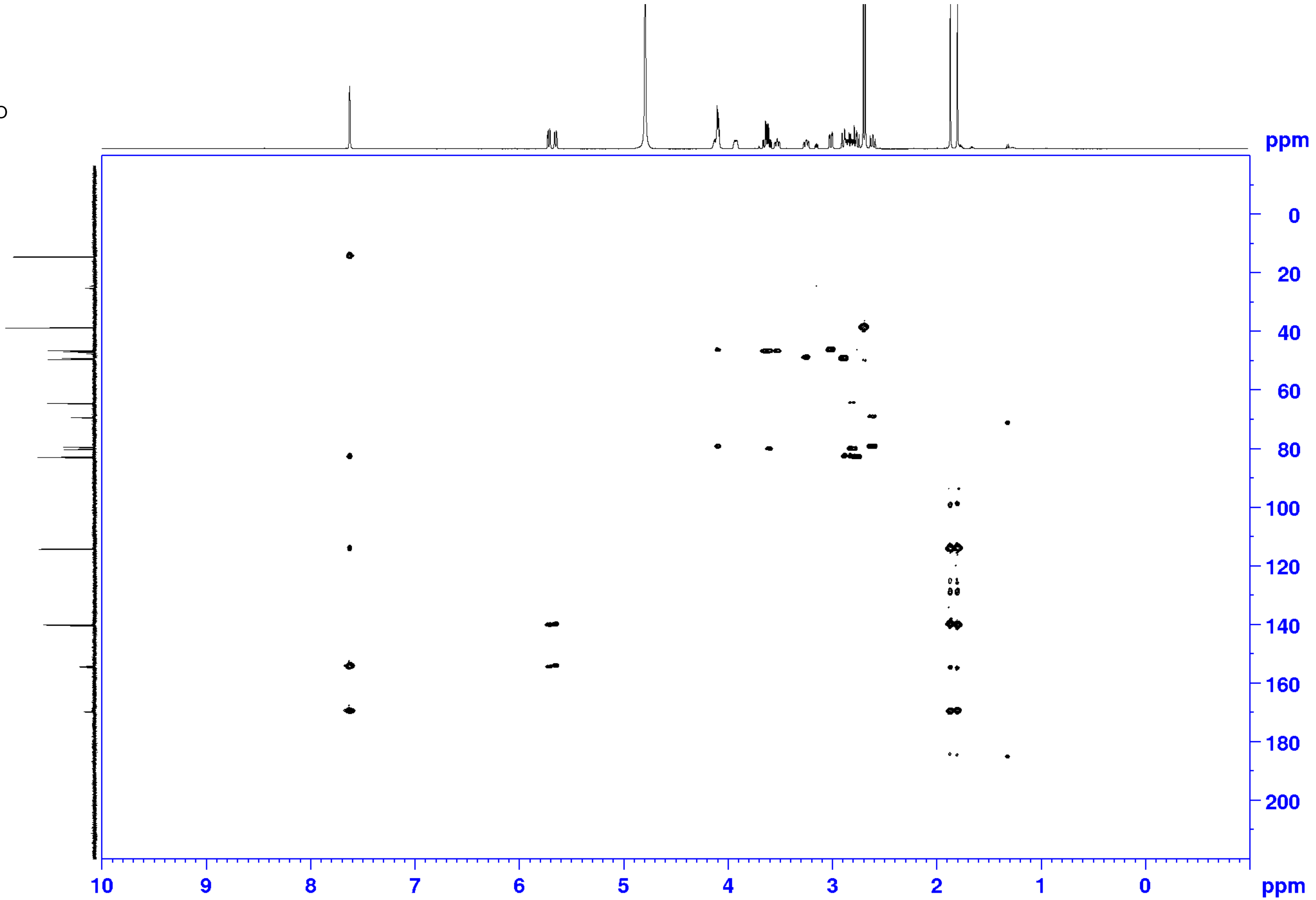

^{1}H NMR (600 MHz, D_2O) of (*S*p)-**28**

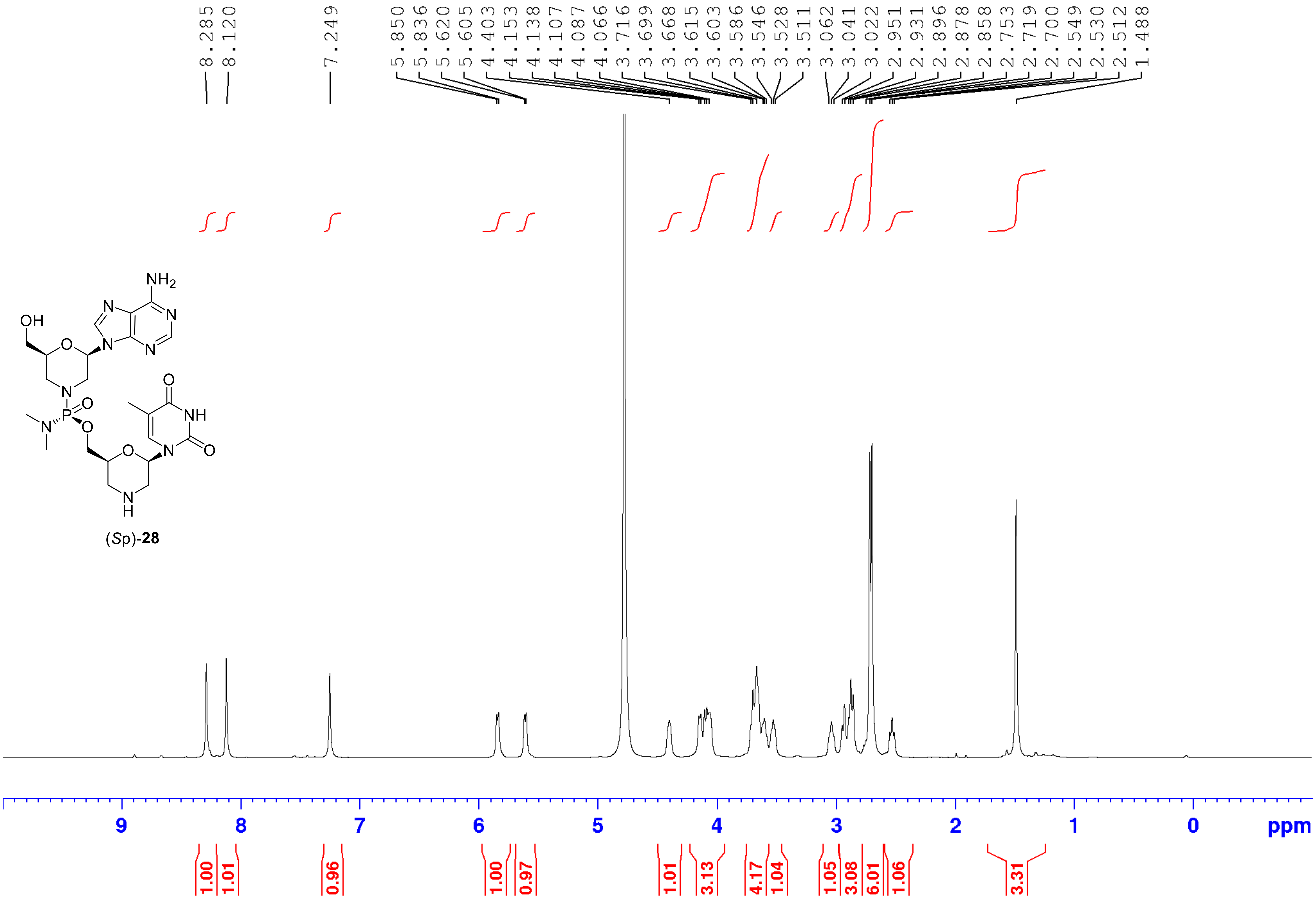

^{13}C {^{1}H} NMR (126 MHz, D_2O) of (*S*p)-**28**

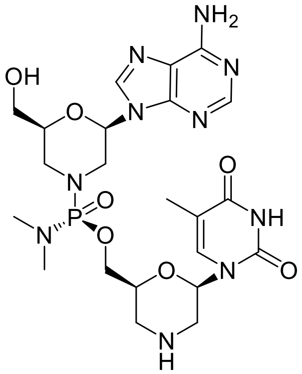

(*S*p)-**28**

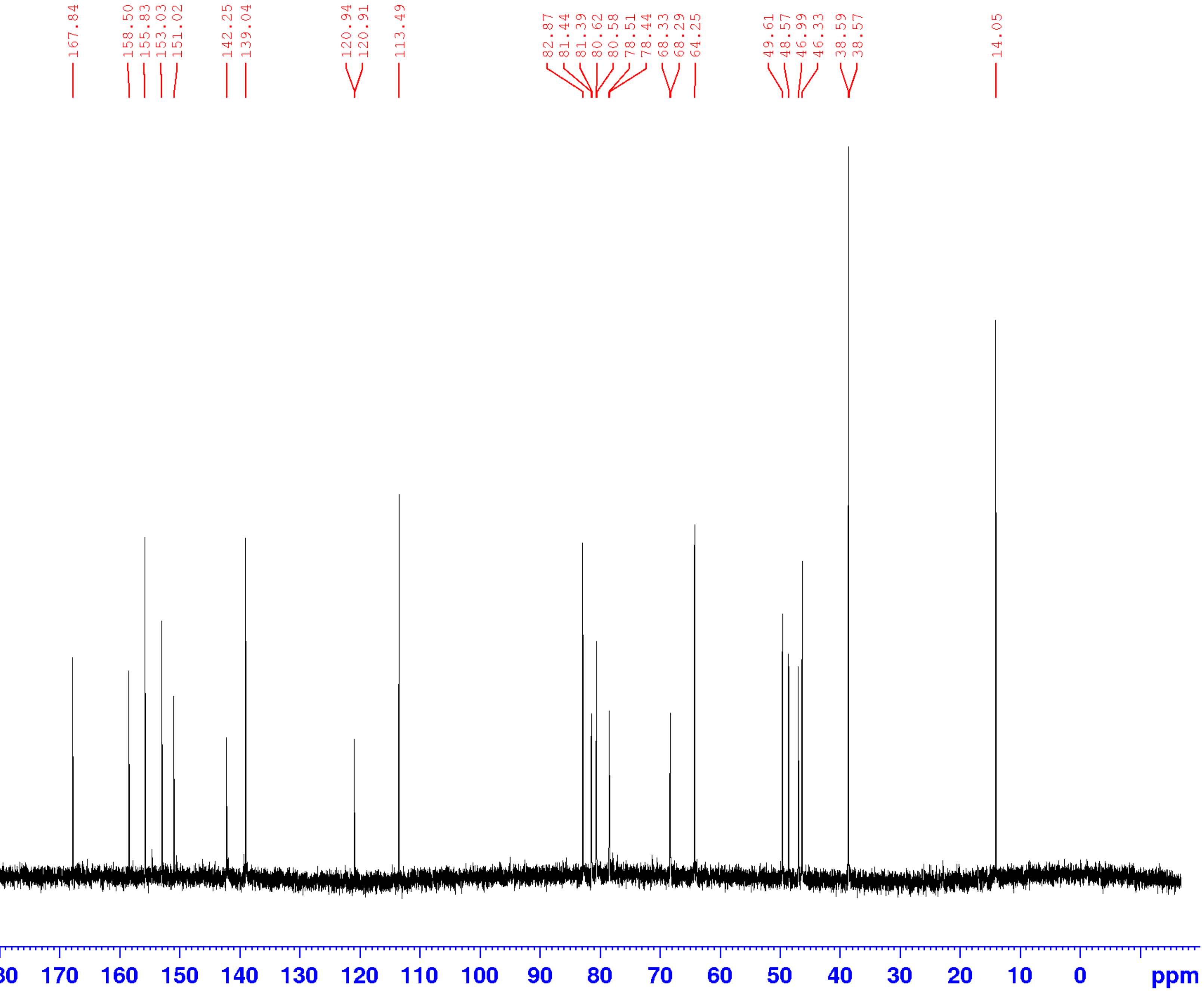

^{31}P {^{1}H} NMR (202 MHz, D_2O) of (*S*p)-**28**

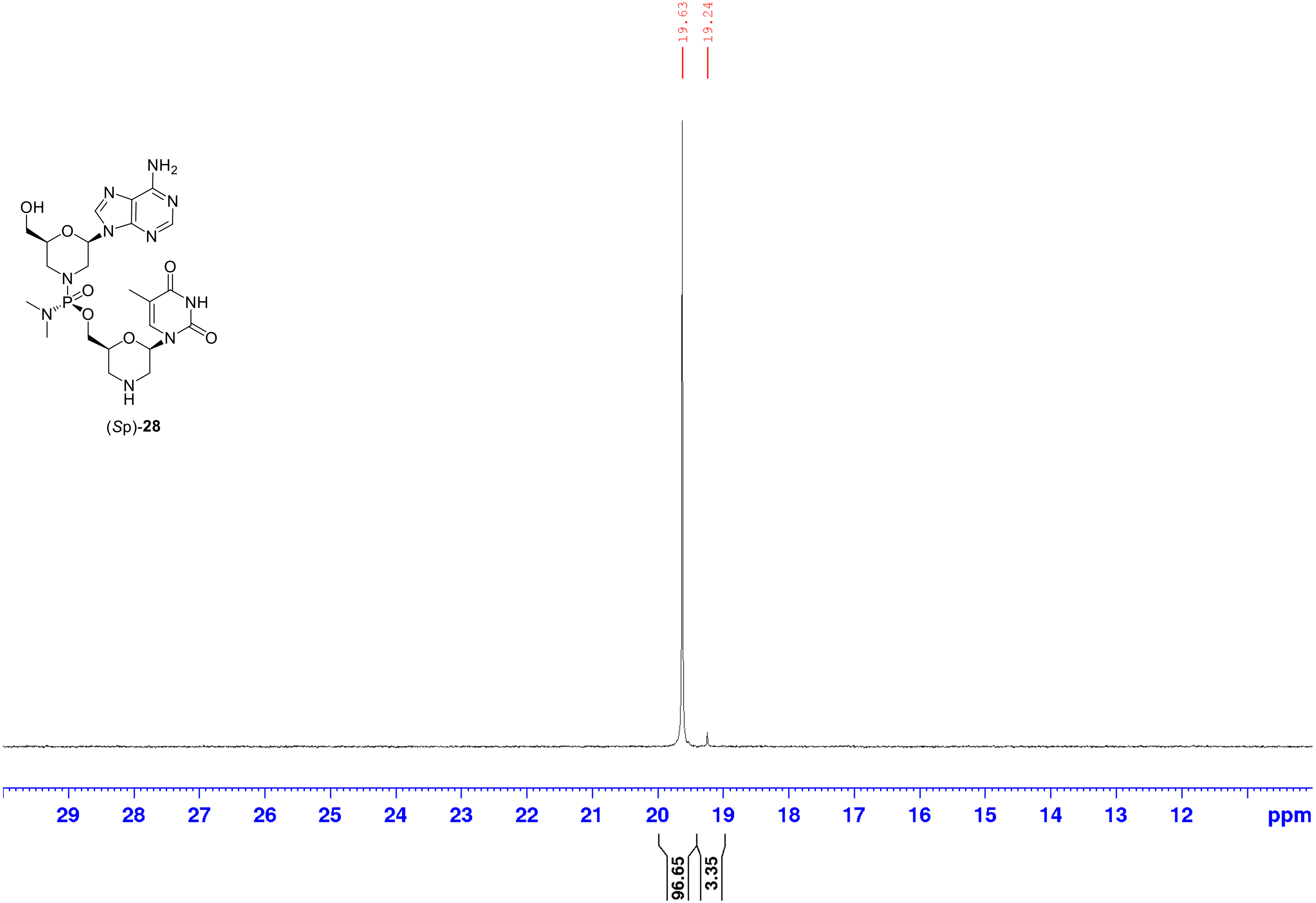

COSY (D_2O) of (*S*p)-**28**

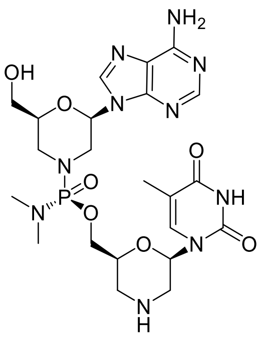

(*S*p)-**28**

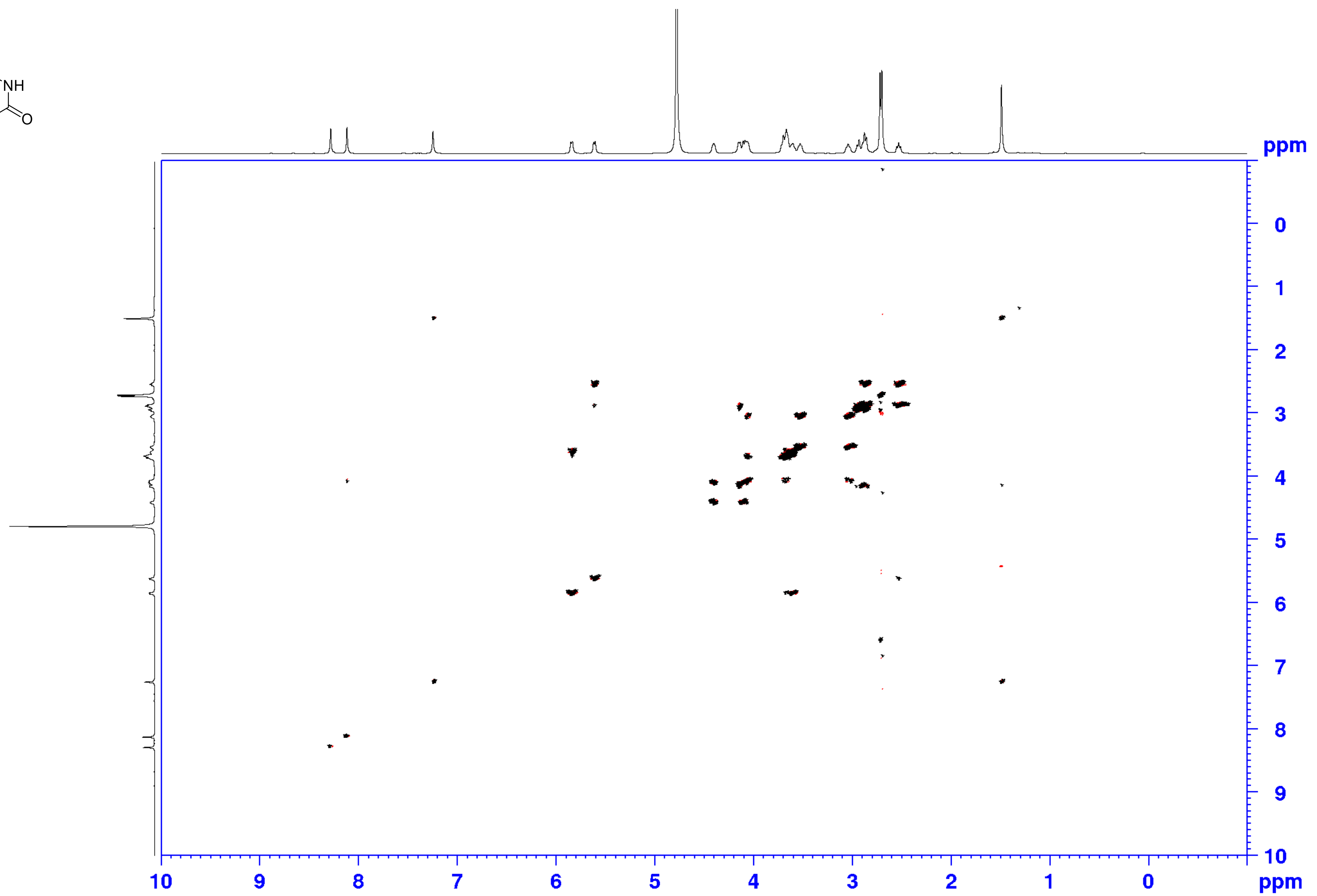

HSQC (D_2O) of (*S*p)-**28**

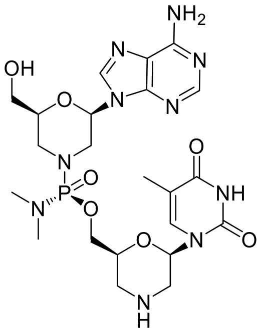

(*S*p)-**28**

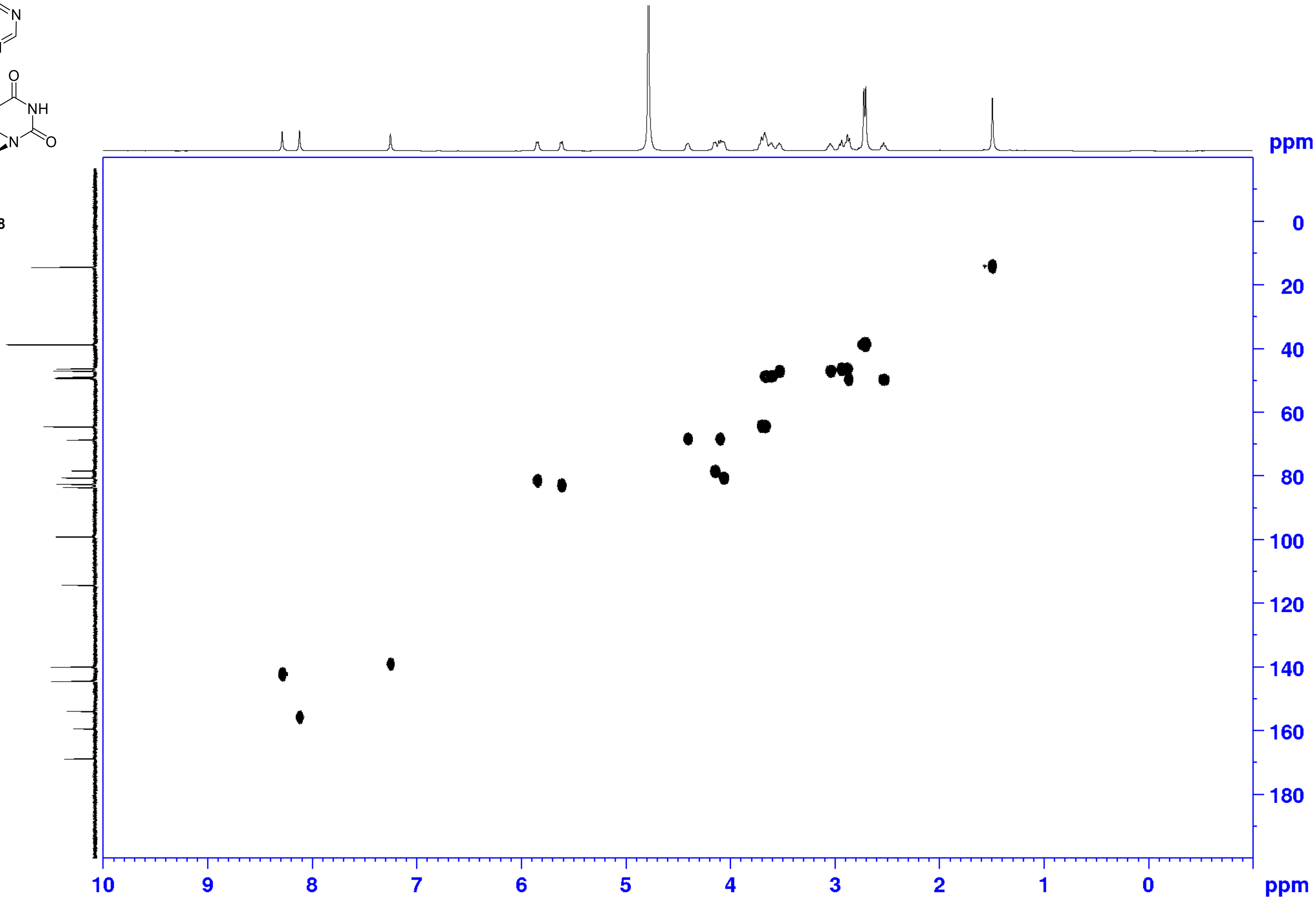

HMBC (D_2O) of (*S*p)-**28**

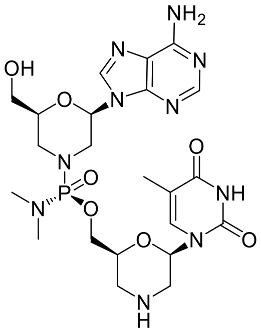

(*S*p)-**28**

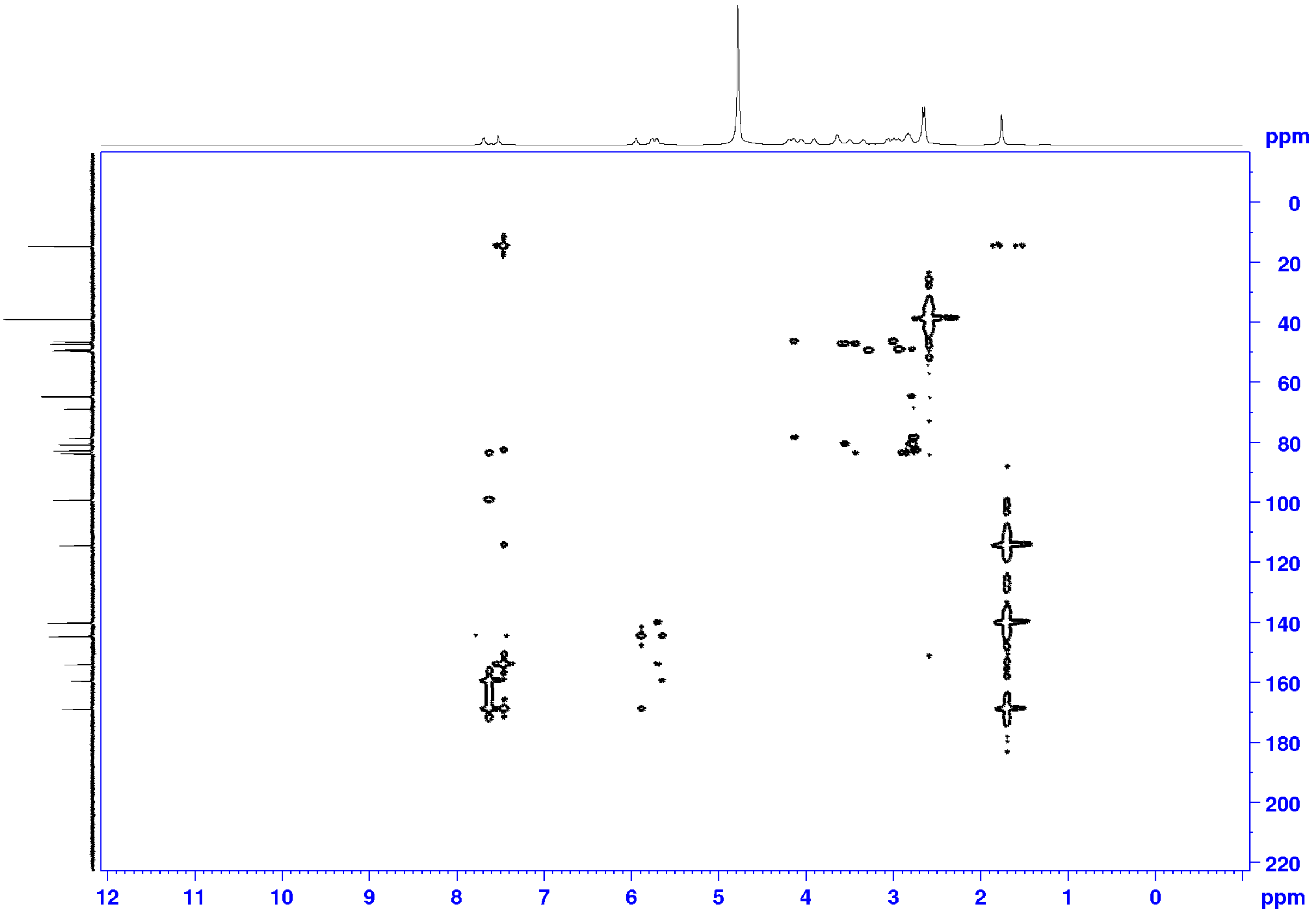

^{1}H NMR (600 MHz, D_2O) of (*R*p)-**28**

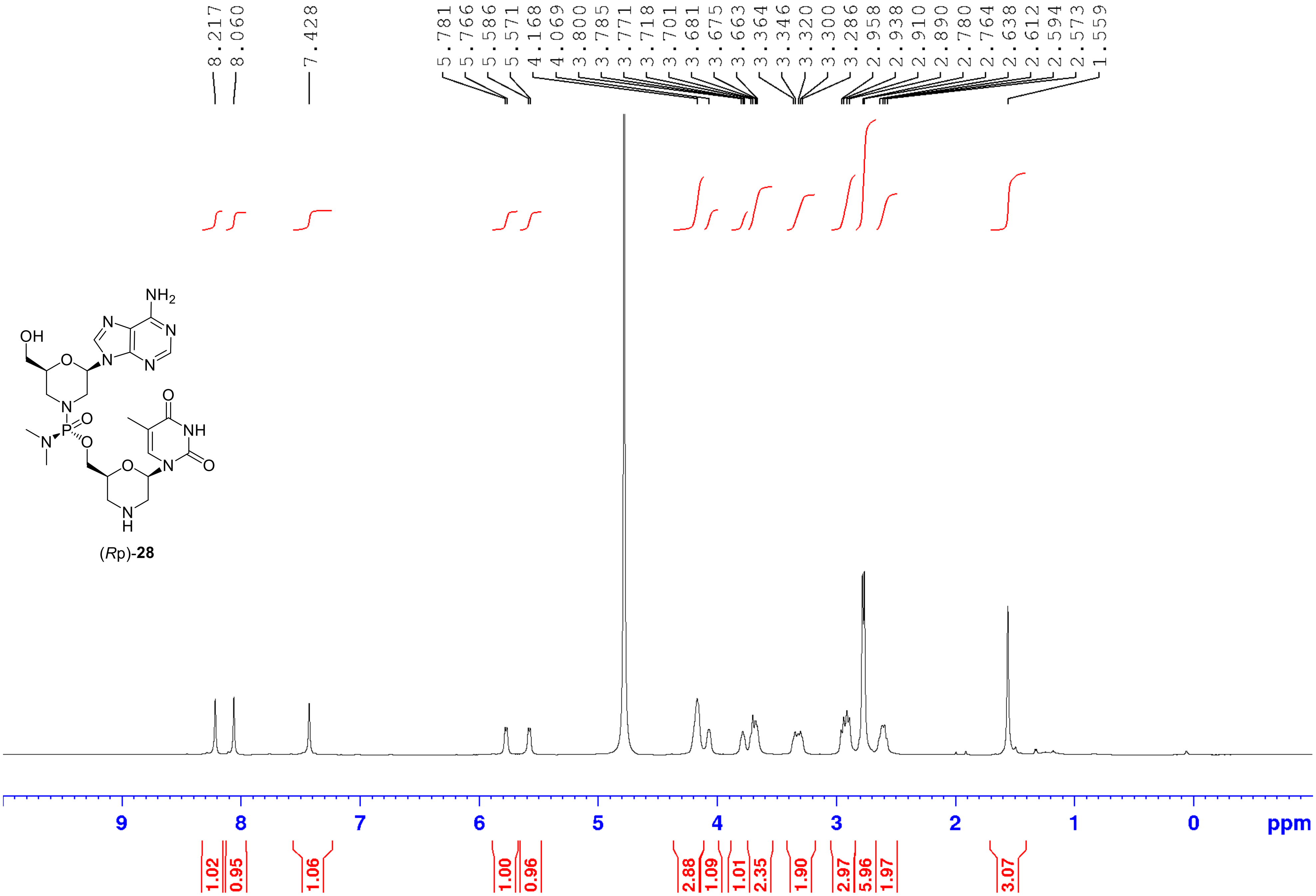

(*R*p)-**28**

$^{13}C\ \{^{1}H\}$ NMR (126 MHz, D_2O) of (*R*p)-**28**

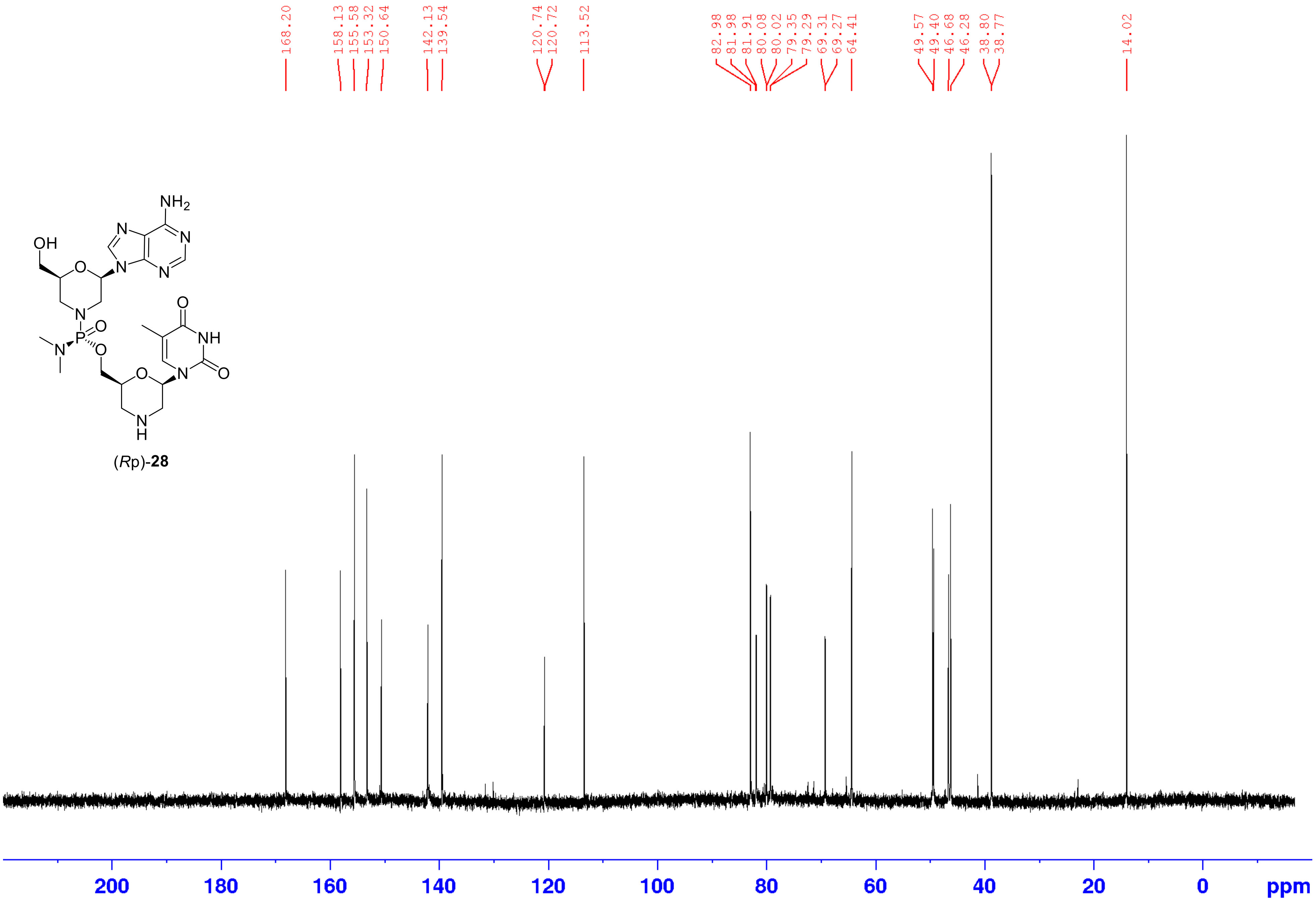

$^{31}P\ \{^1H\}$ NMR (202 MHz, D_2O) of (*R*p)-**28**

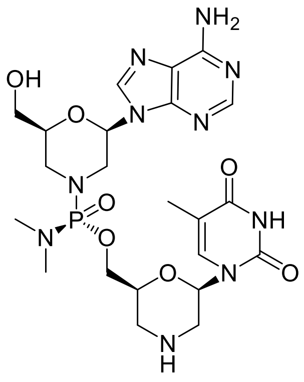

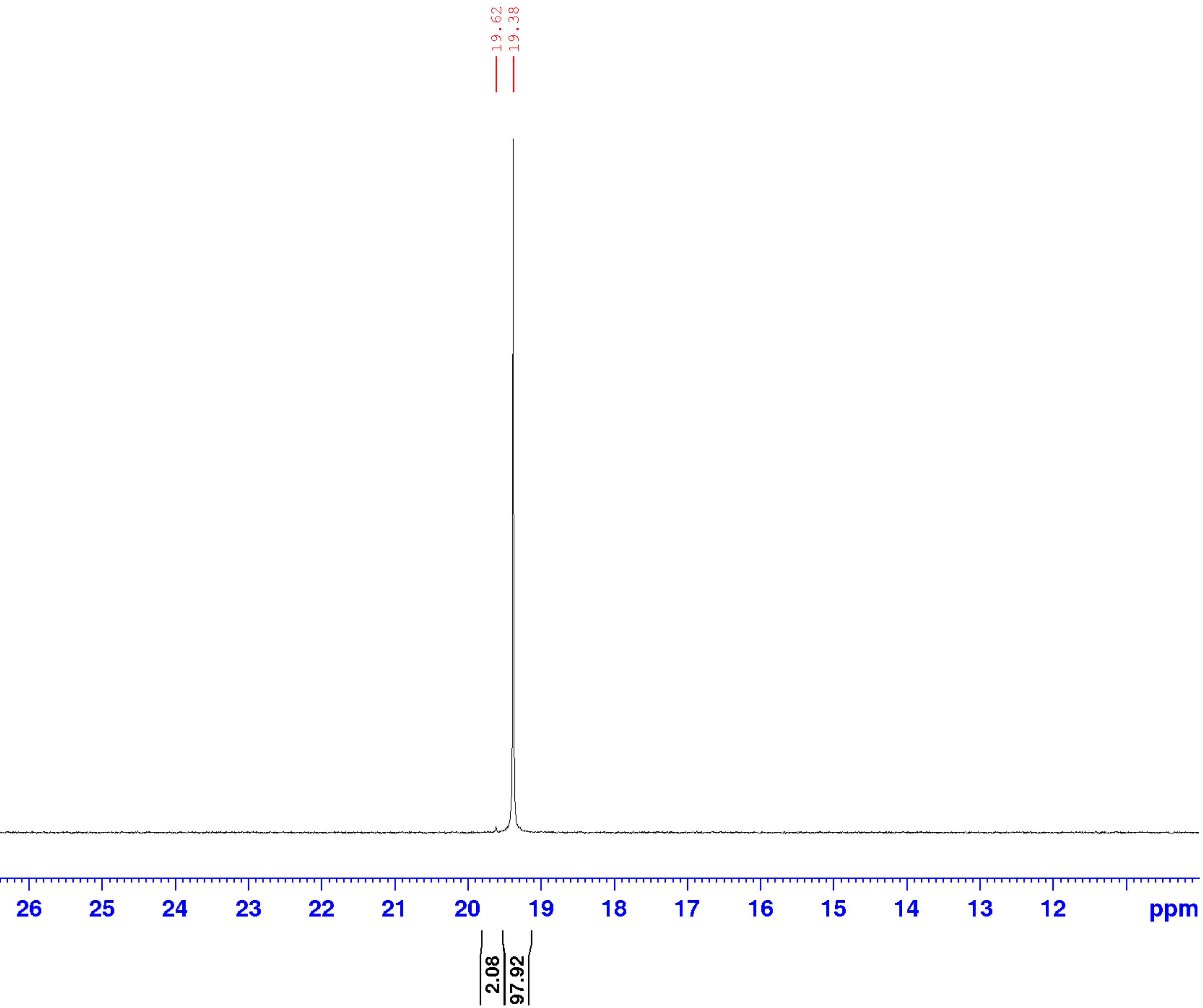

COSY (D_2O) of (*R*p)-**28**

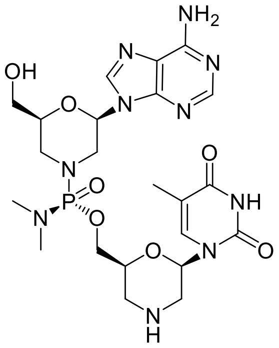

(*R*p)-**28**

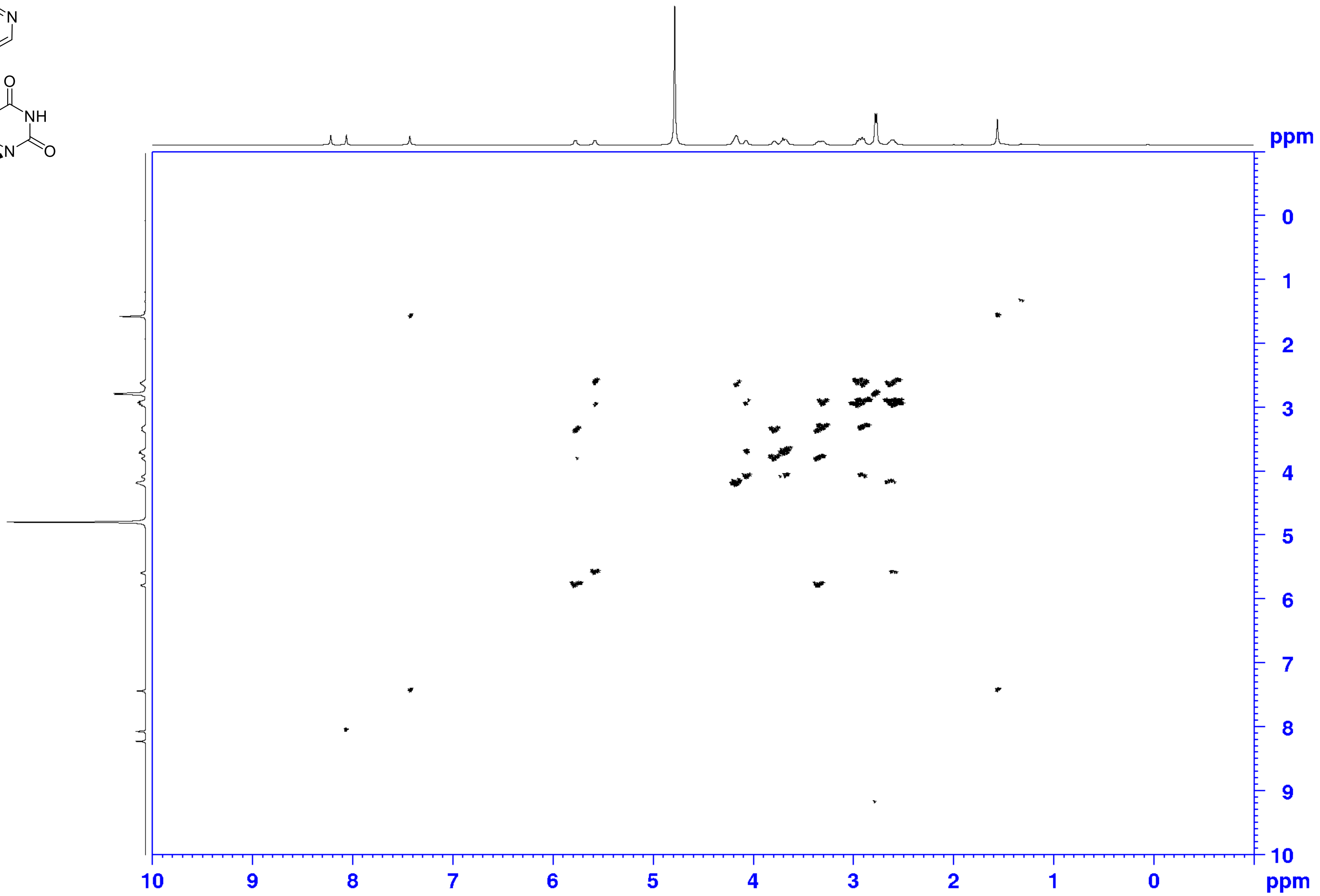

HSQC (D_2O) of (*R*p)-**28**

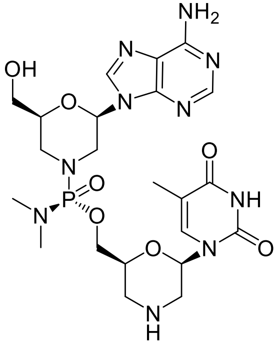

(*R*p)-**28**

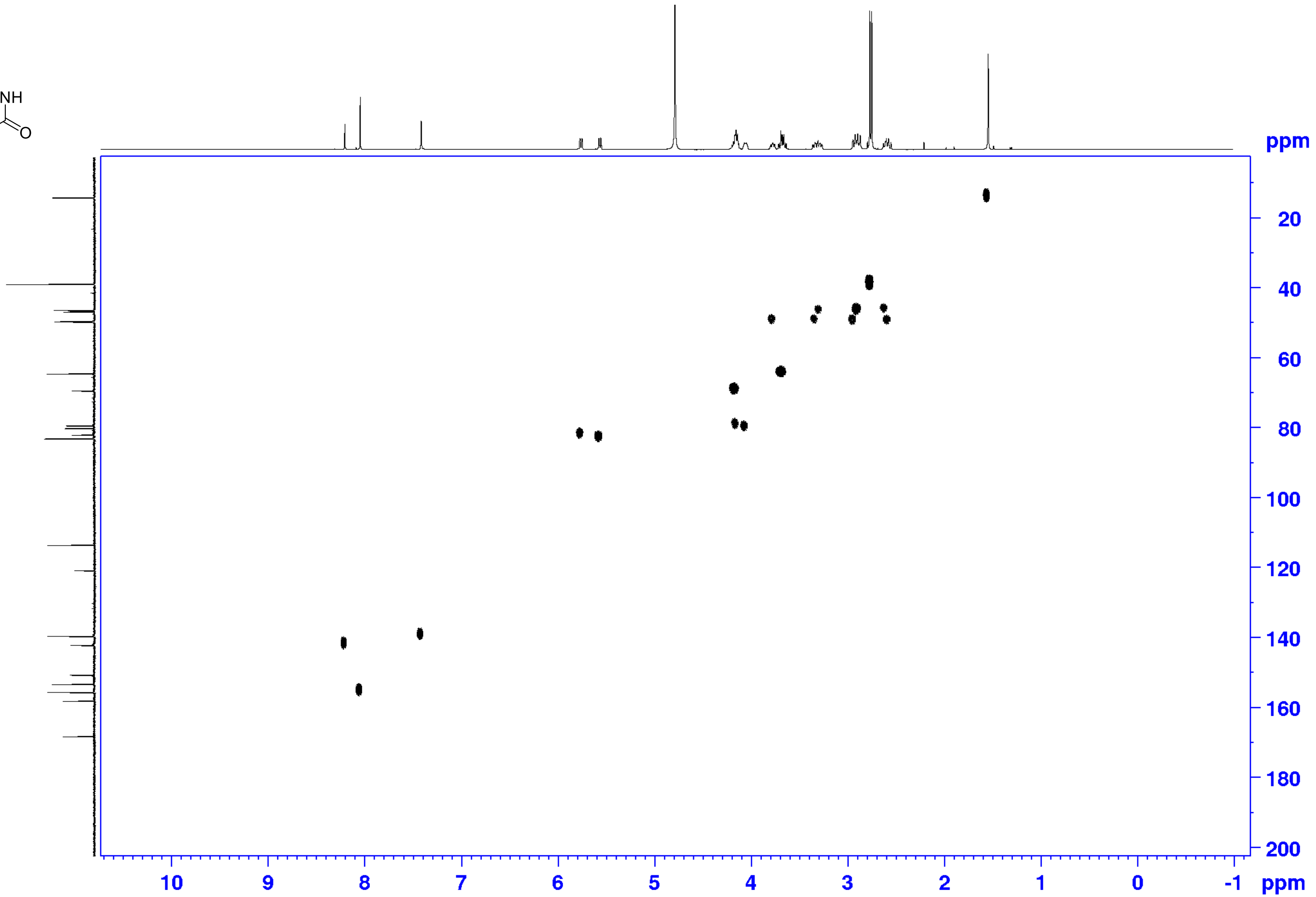

HMBC (D_2O) of (*R*p)-**28**

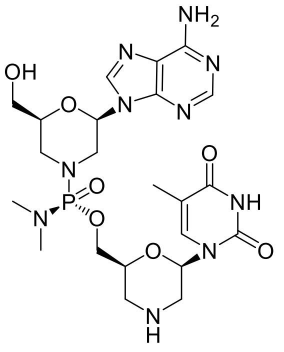

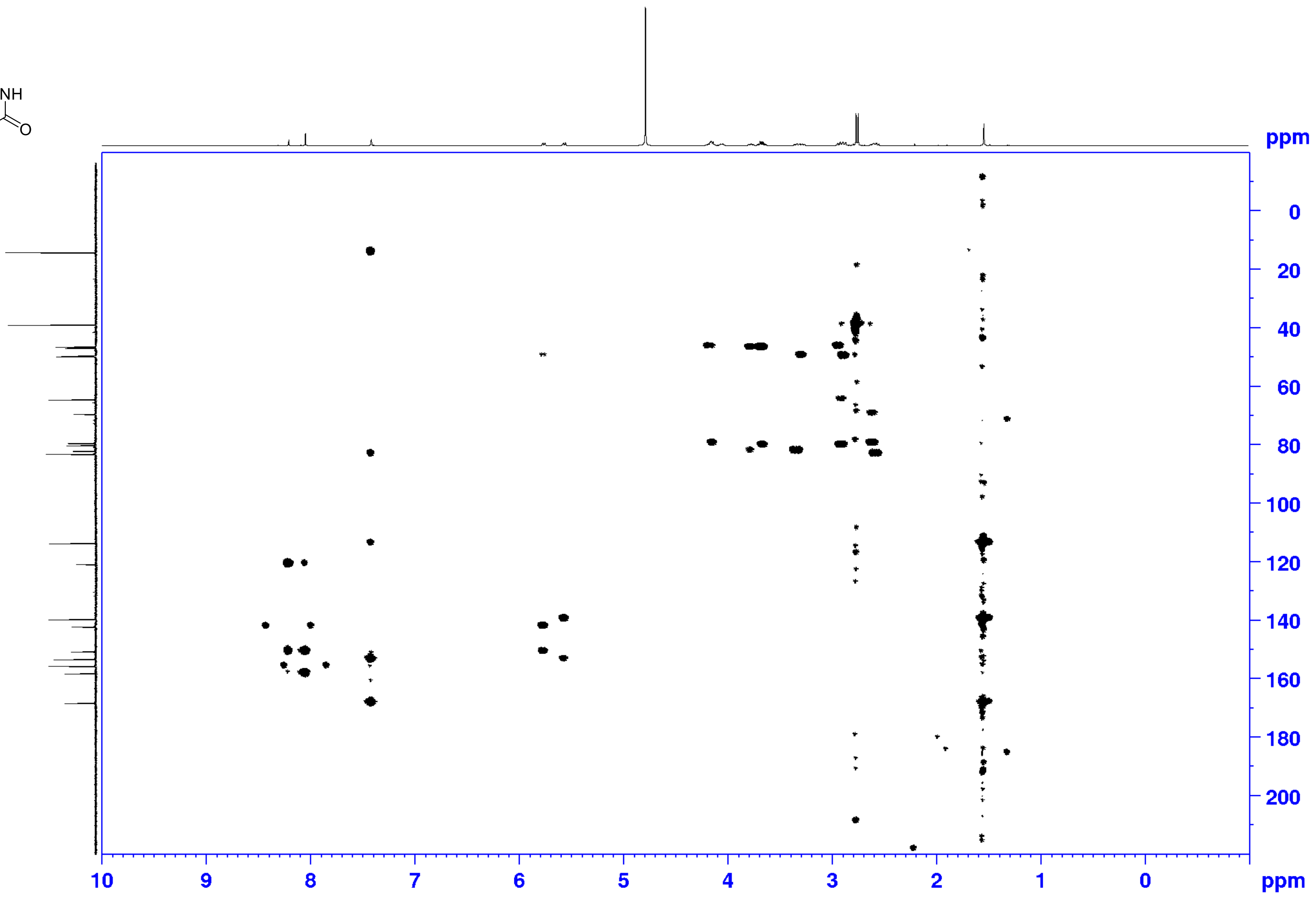

^{1}H NMR (600 MHz, D_2O) of (*S*p)-**29**

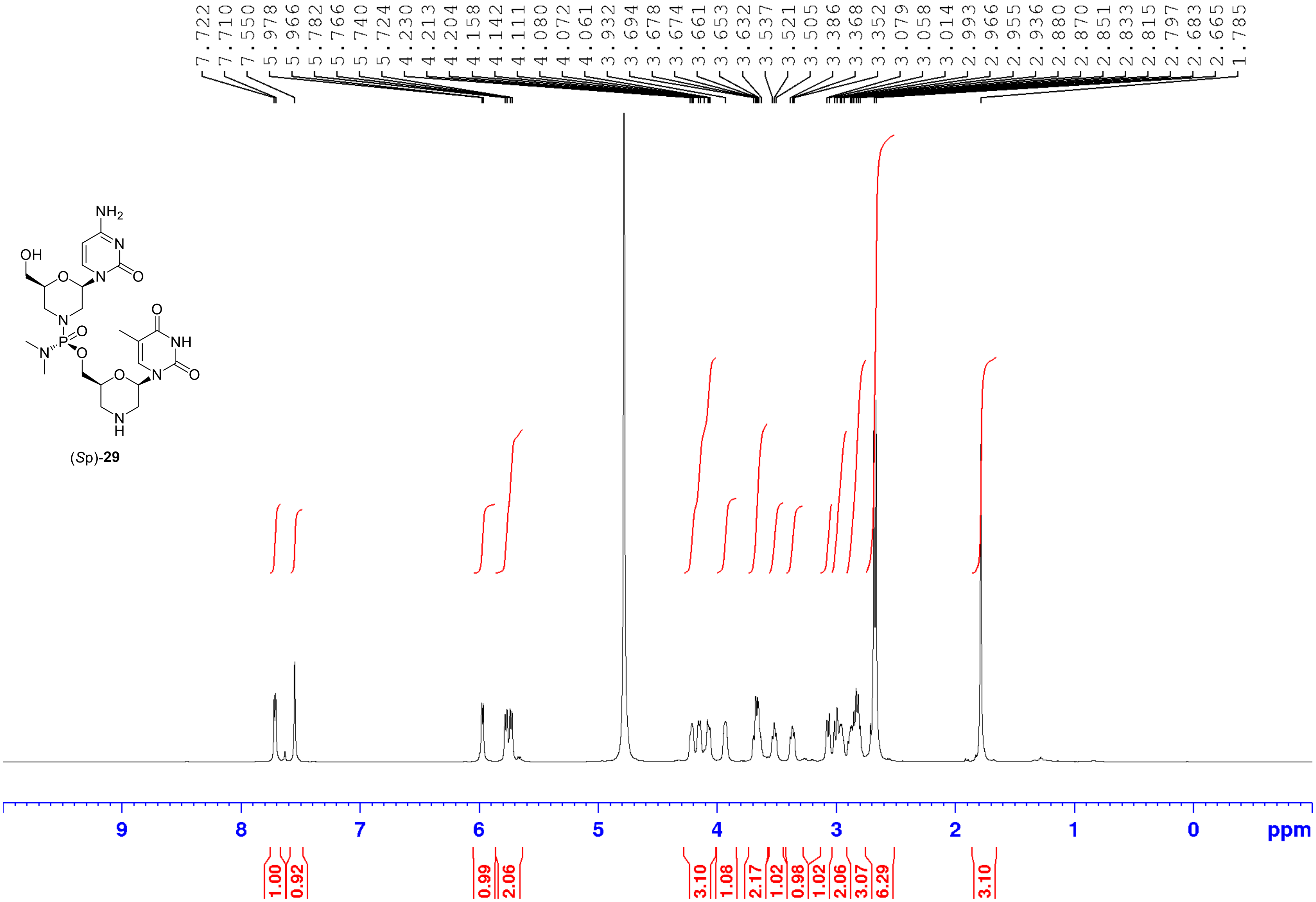

$^{13}C\{^1H\}$ NMR (126 MHz, D_2O) of (*S*p)-**29**

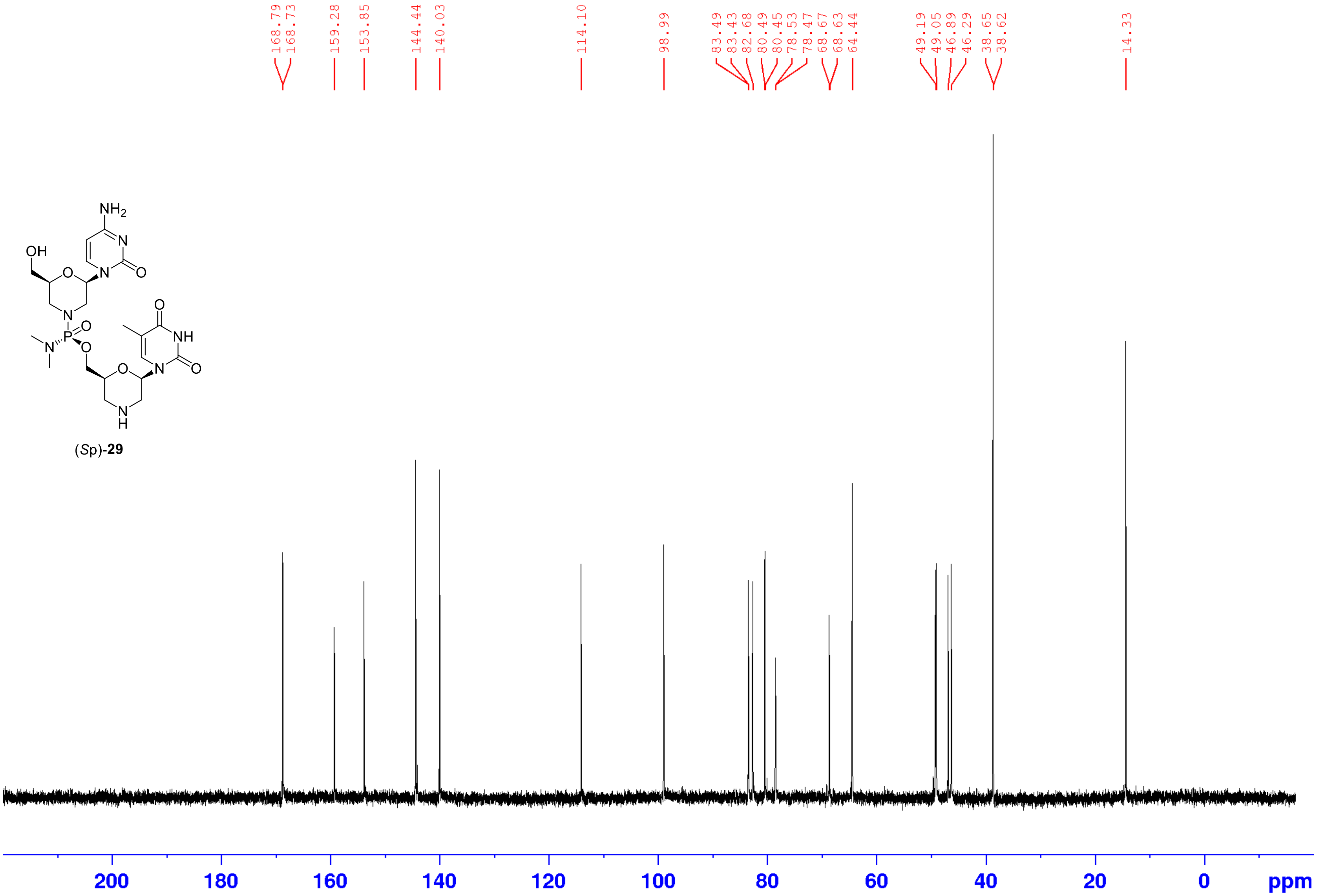

(*S*p)-**29**

$^{31}P\{^{1}H\}$ NMR (202 MHz, D_2O) of (*S*p)-**29**

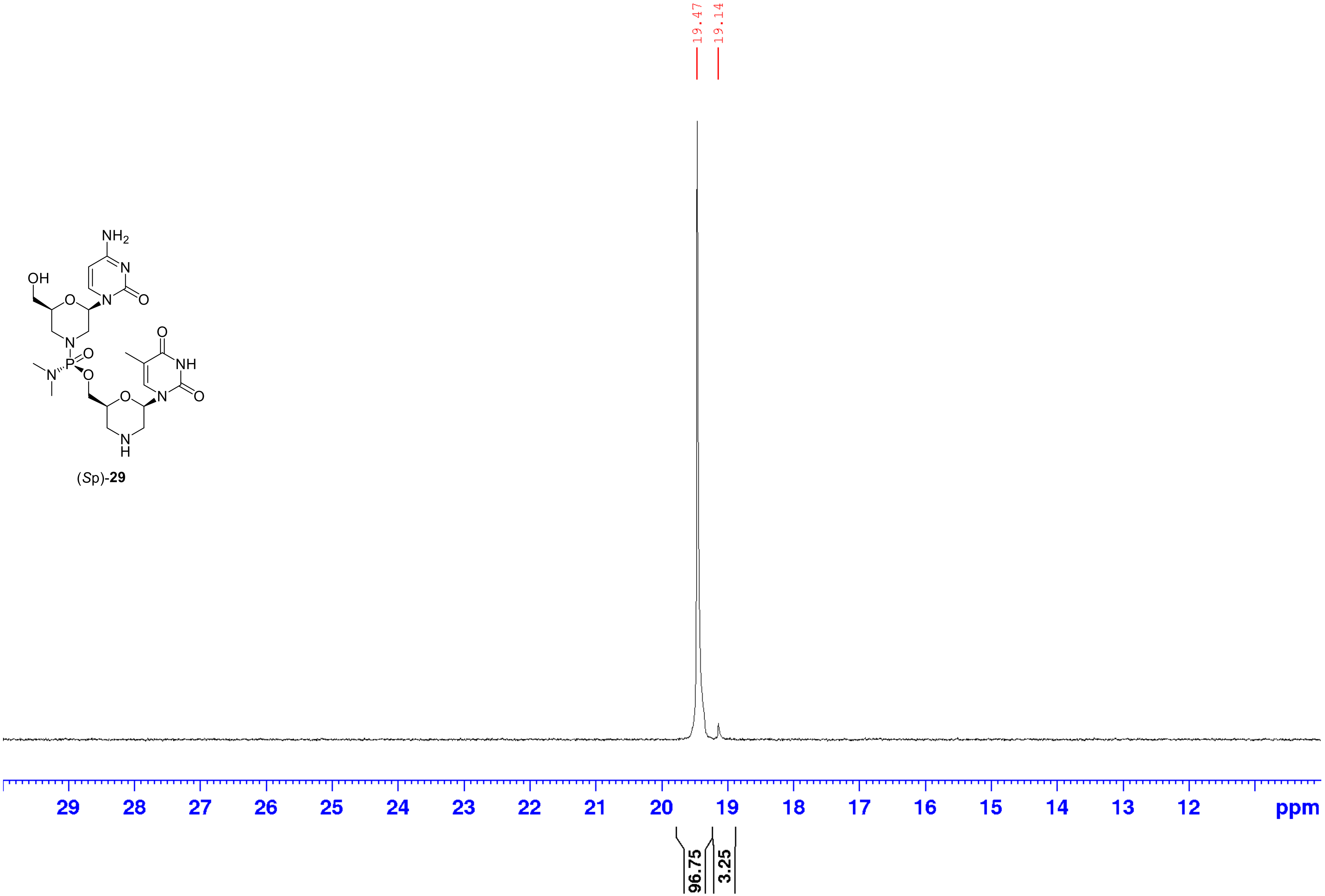

(*S*p)-**29**

COSY (D_2O) of (*S*p)-**29**

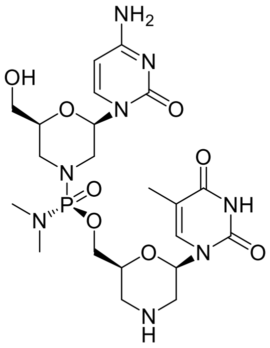

(*S*p)-**29**

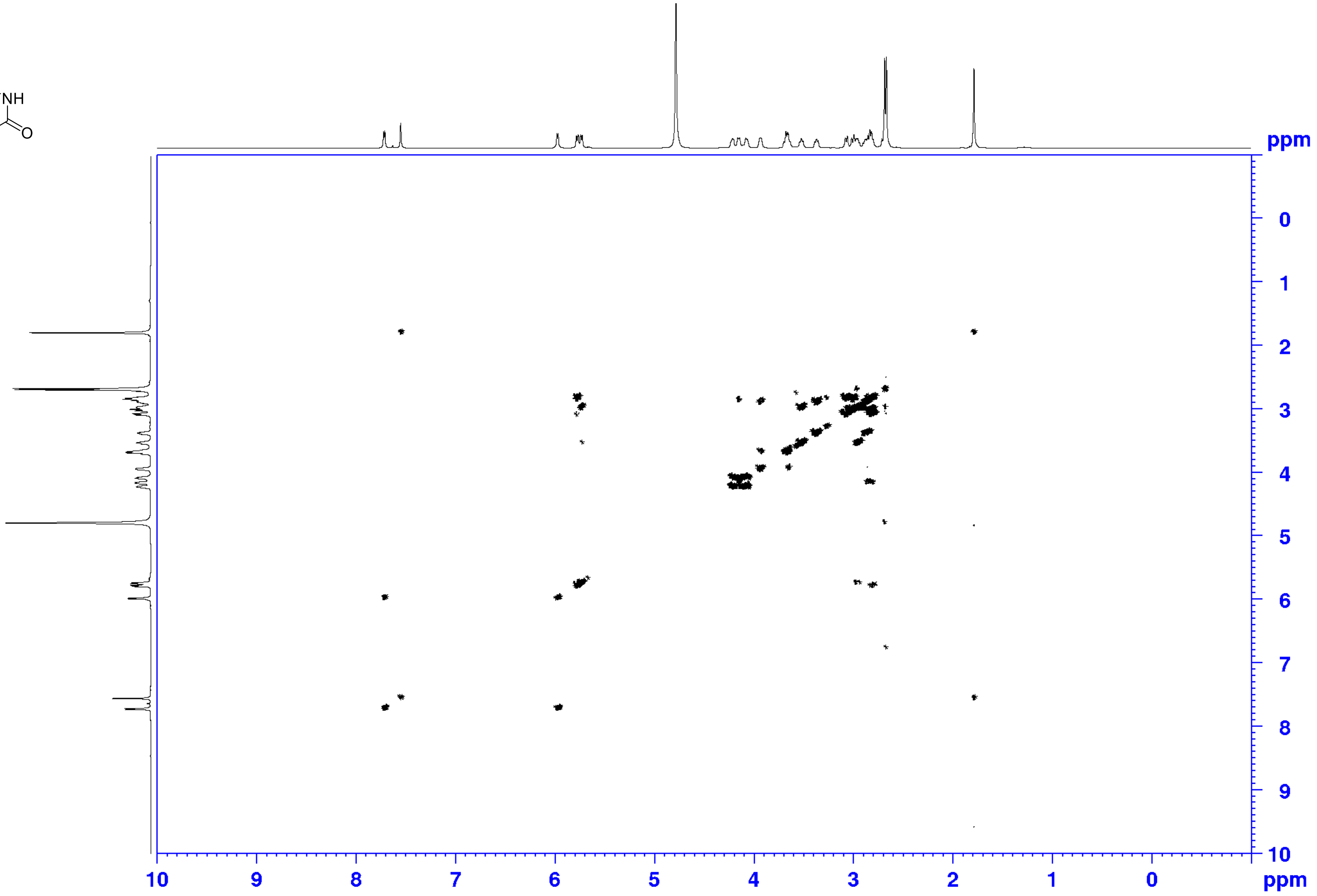

HSQC (D_2O) of (*S*p)-**29**

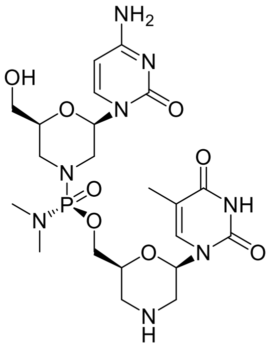

(*S*p)-**29**

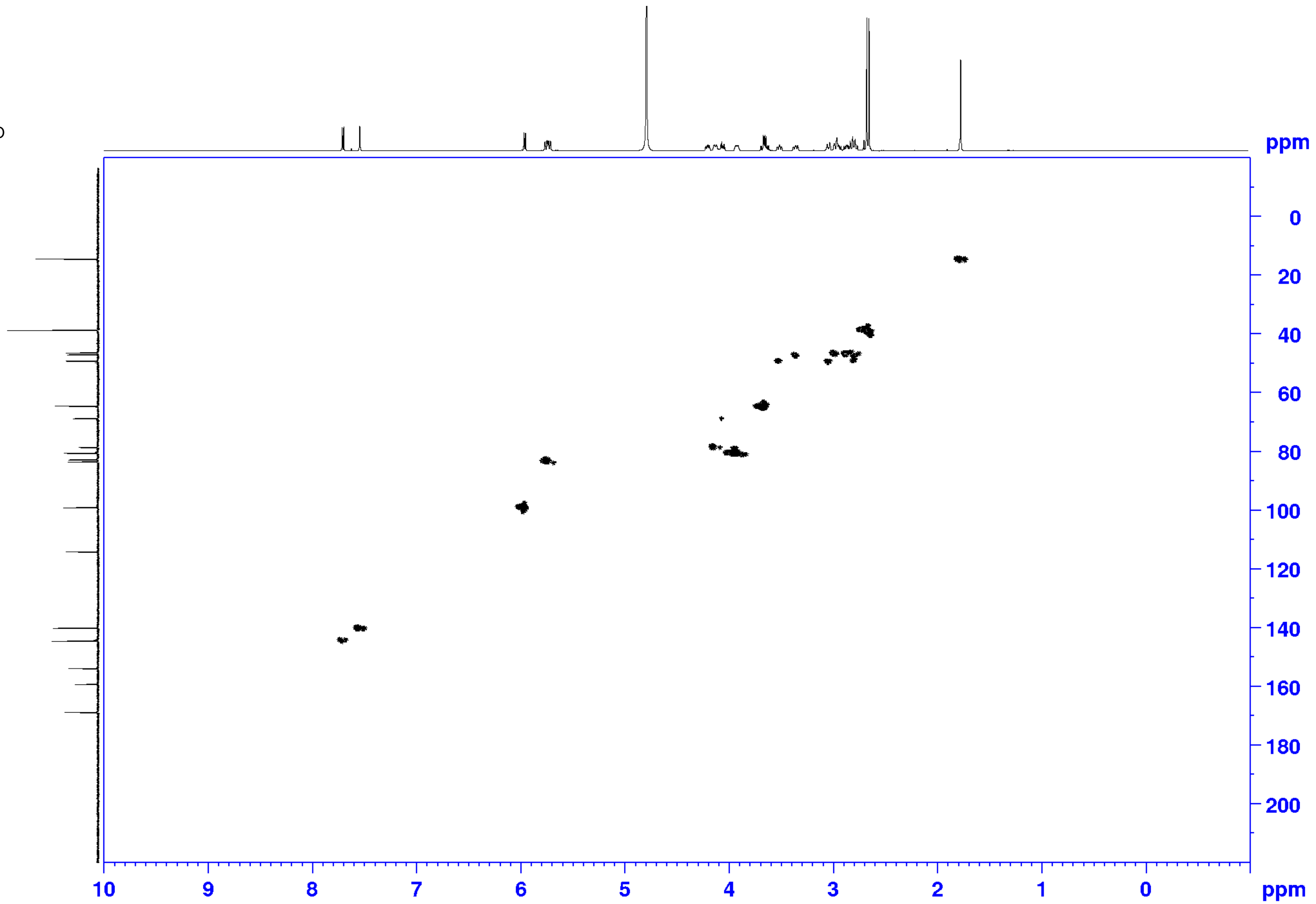

HMBC (D_2O) of (*S*p)-**29**

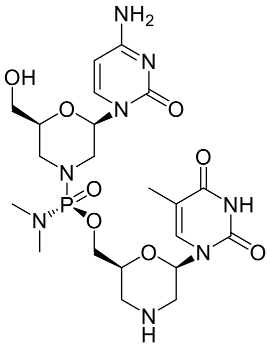

(*S*p)-**29**

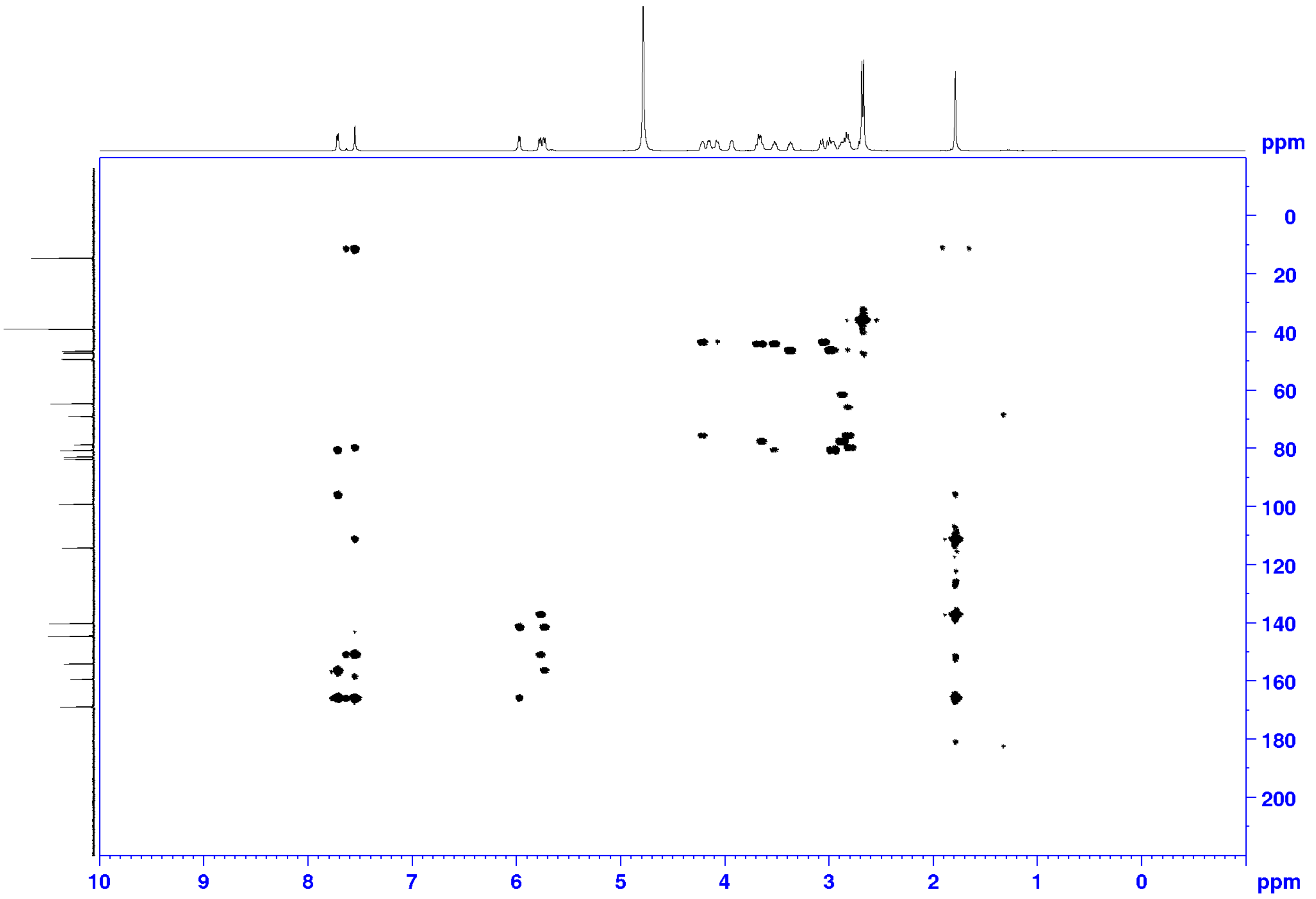

^{1}H NMR (600 MHz, D_2O) of (*R*p)-**29**

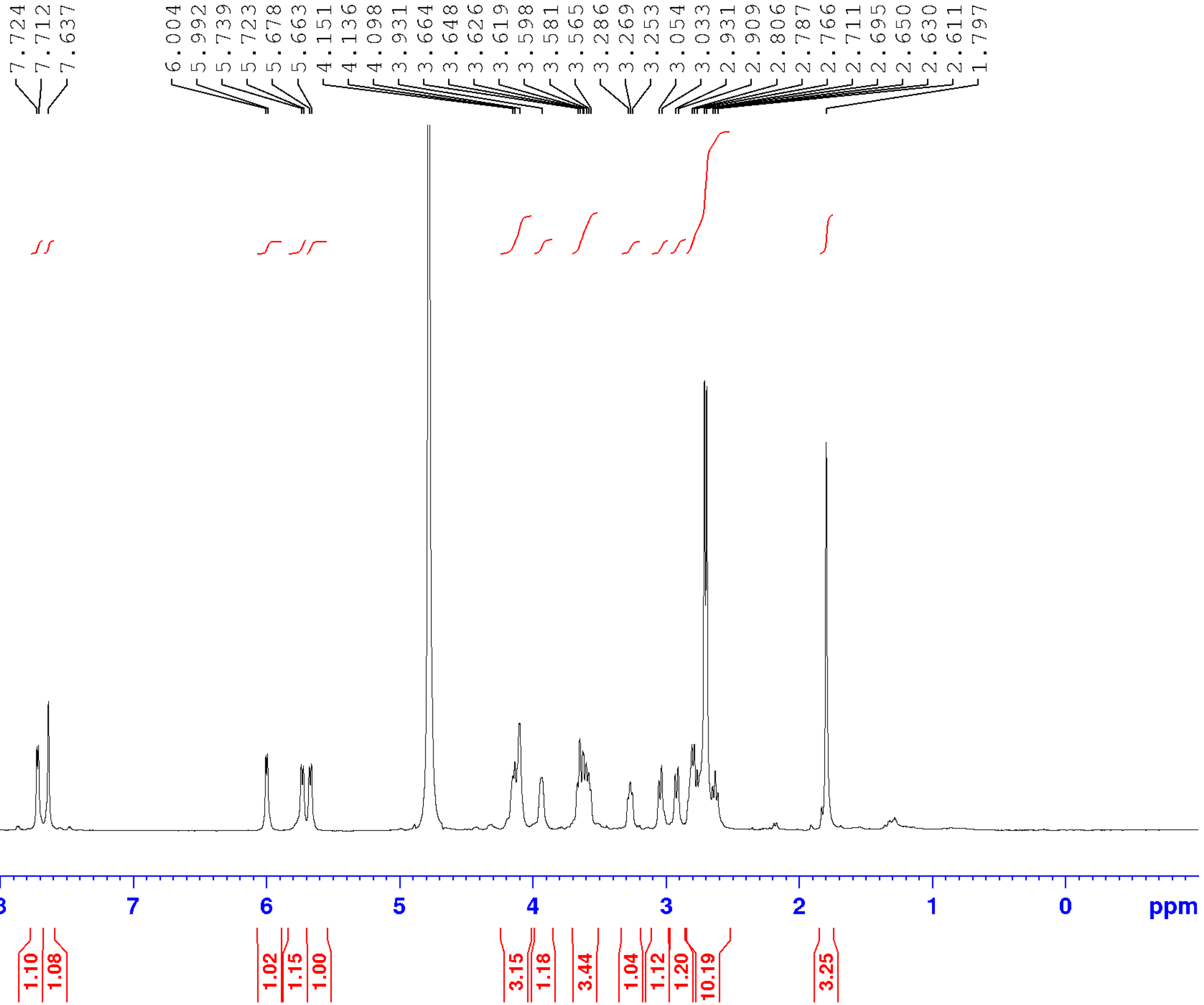

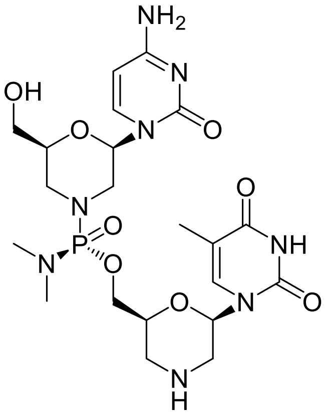

(*R*p)-**29**

$^{13}C\{^{1}H\}$ NMR (126 MHz, D_2O) of (*R*p)-**29**

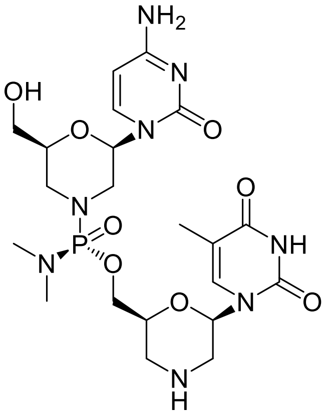

(*R*p)-**29**

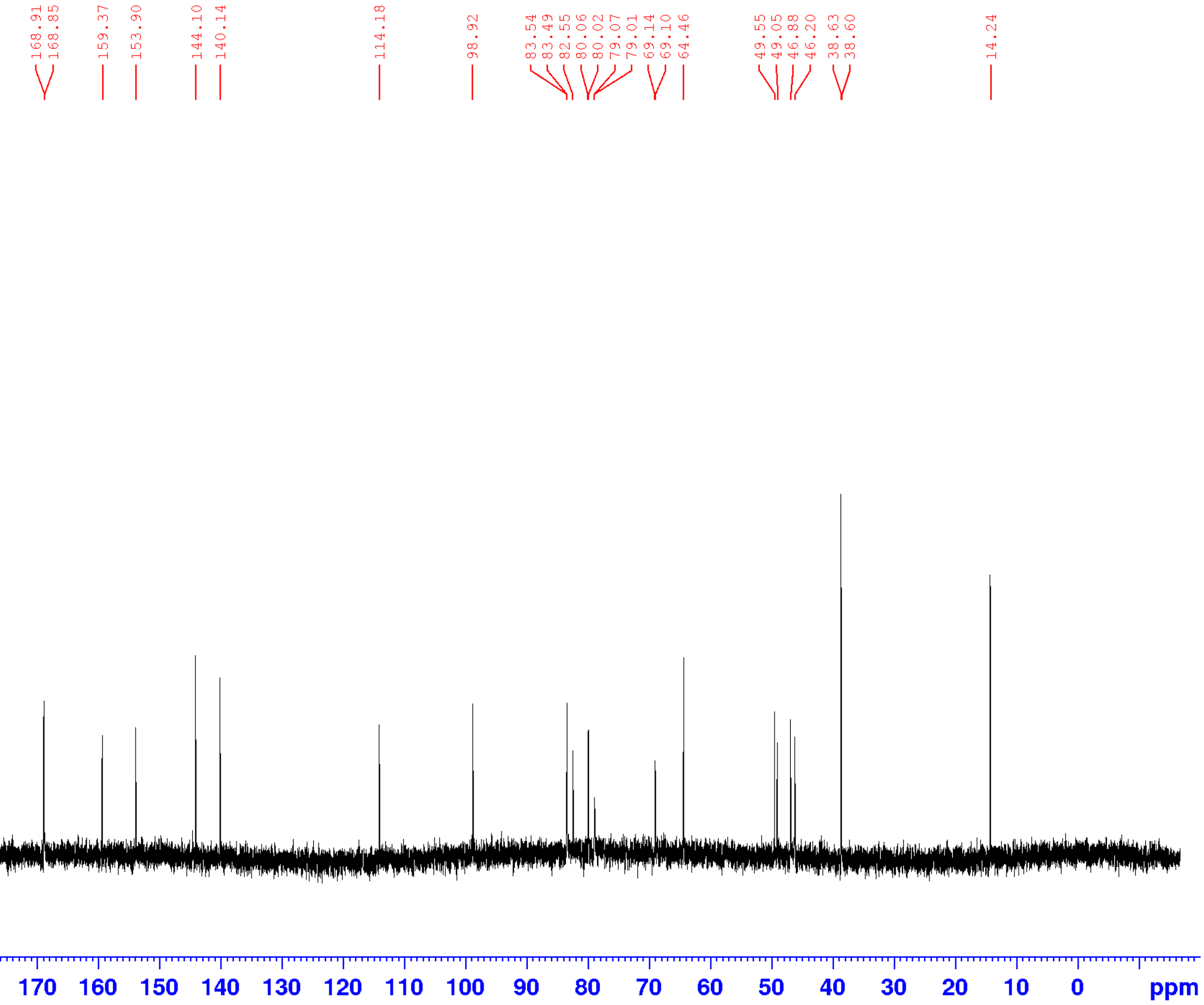

^{31}P {^{1}H} NMR (202 MHz, D_2O) of (*R*p)-**29**

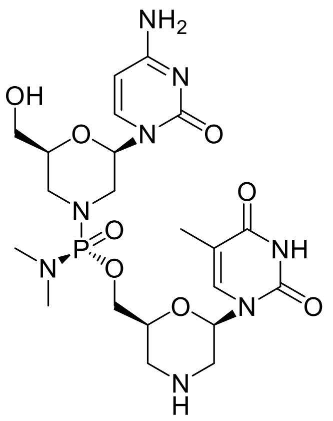

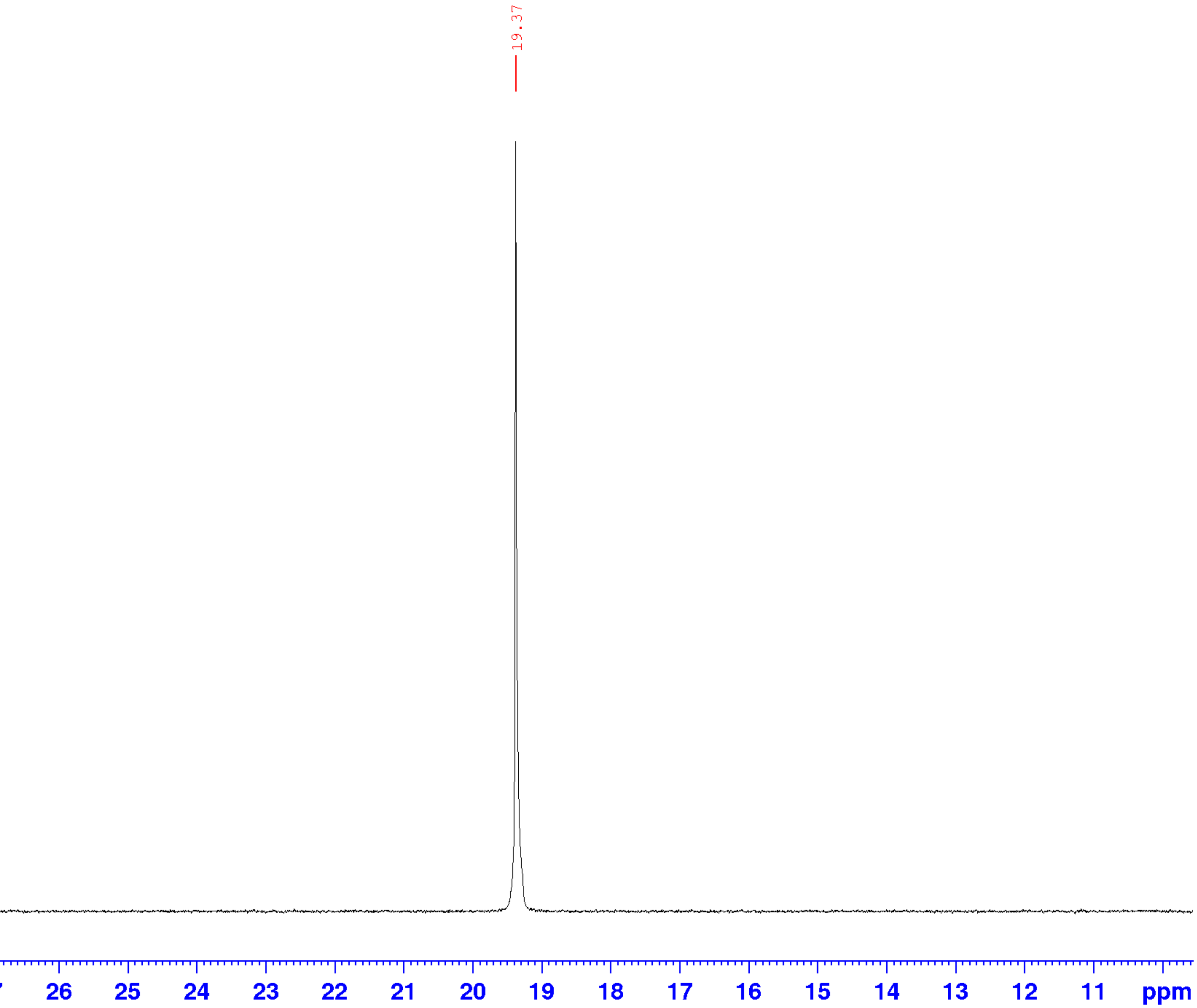

COSY (D_2O) of (*R*p)-**29**

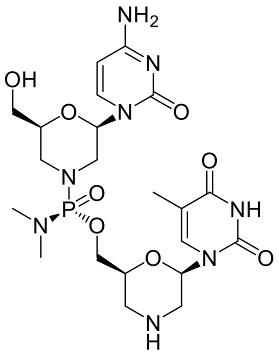

(*R*p)-**29**

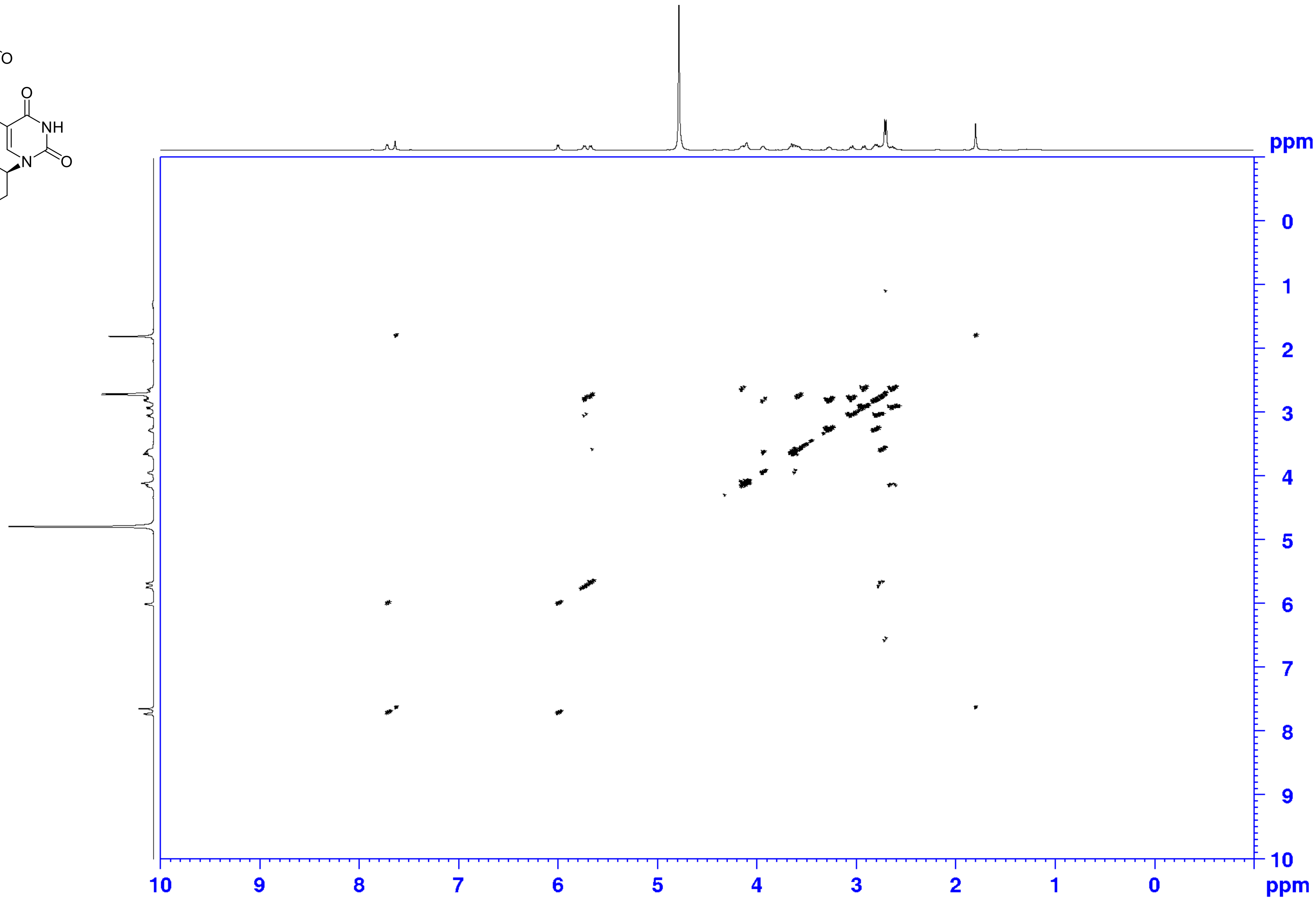

HSQC (D_2O) of (*R*p)-**29**

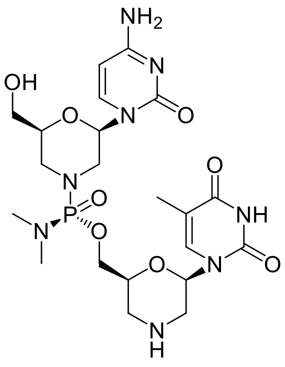

(*R*p)-**29**

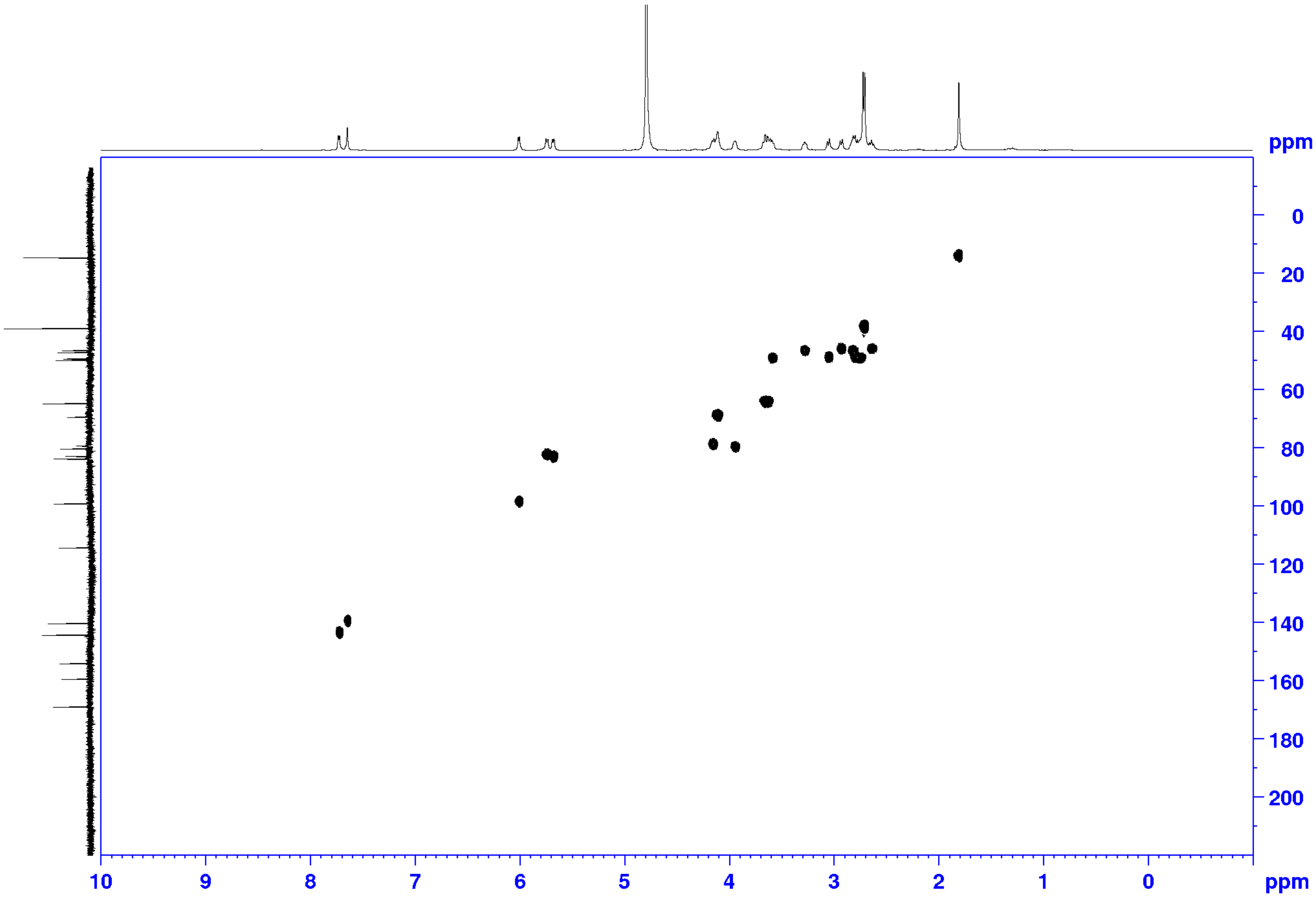

HMBC (D_2O) of (*R*p)-**29**

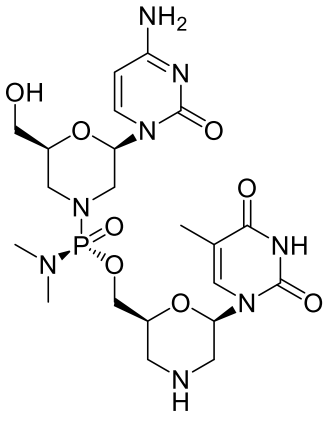

(*R*p)-**29**

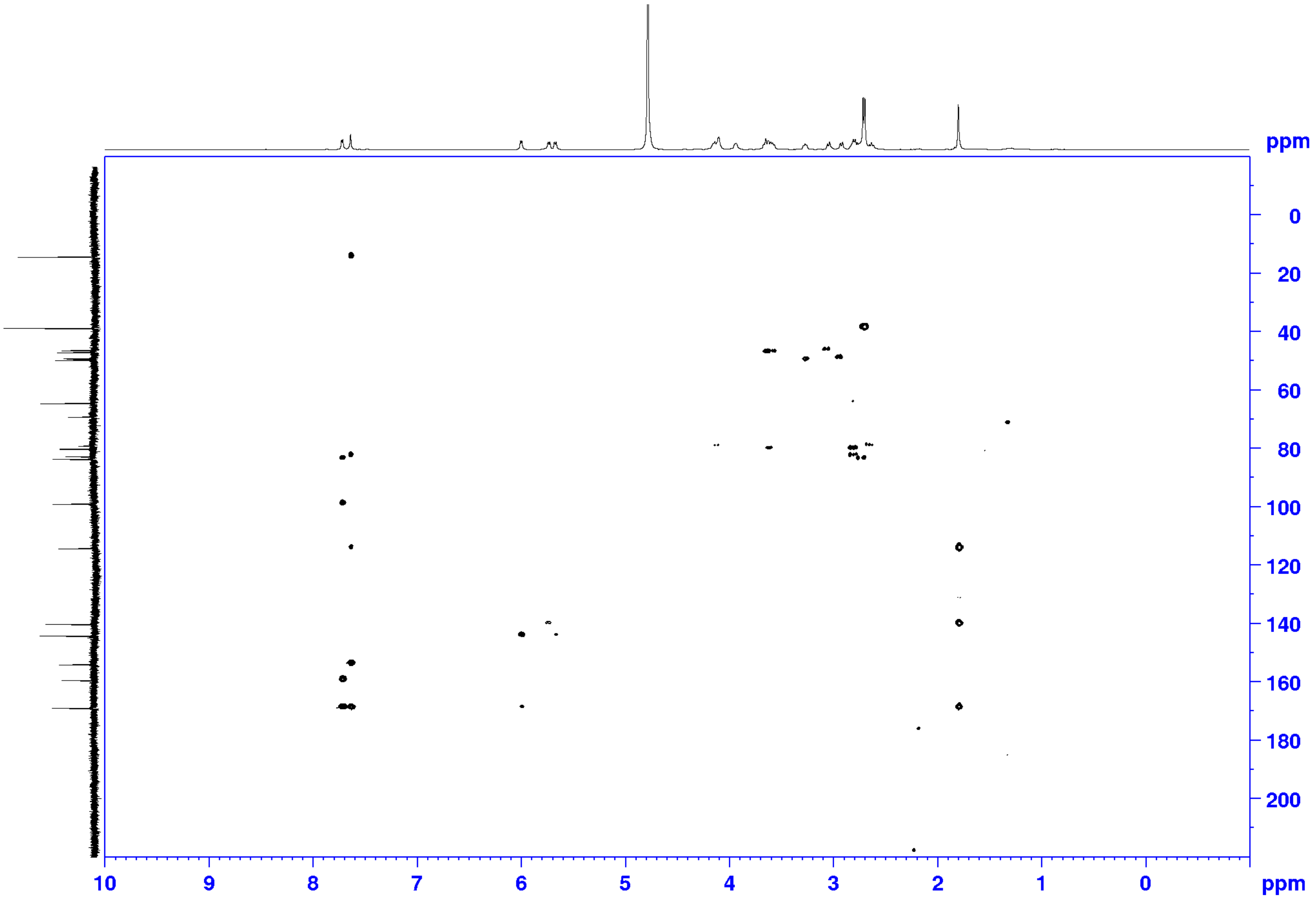

^{1}H NMR (600 MHz, D_2O) of (*S*p)-**30**

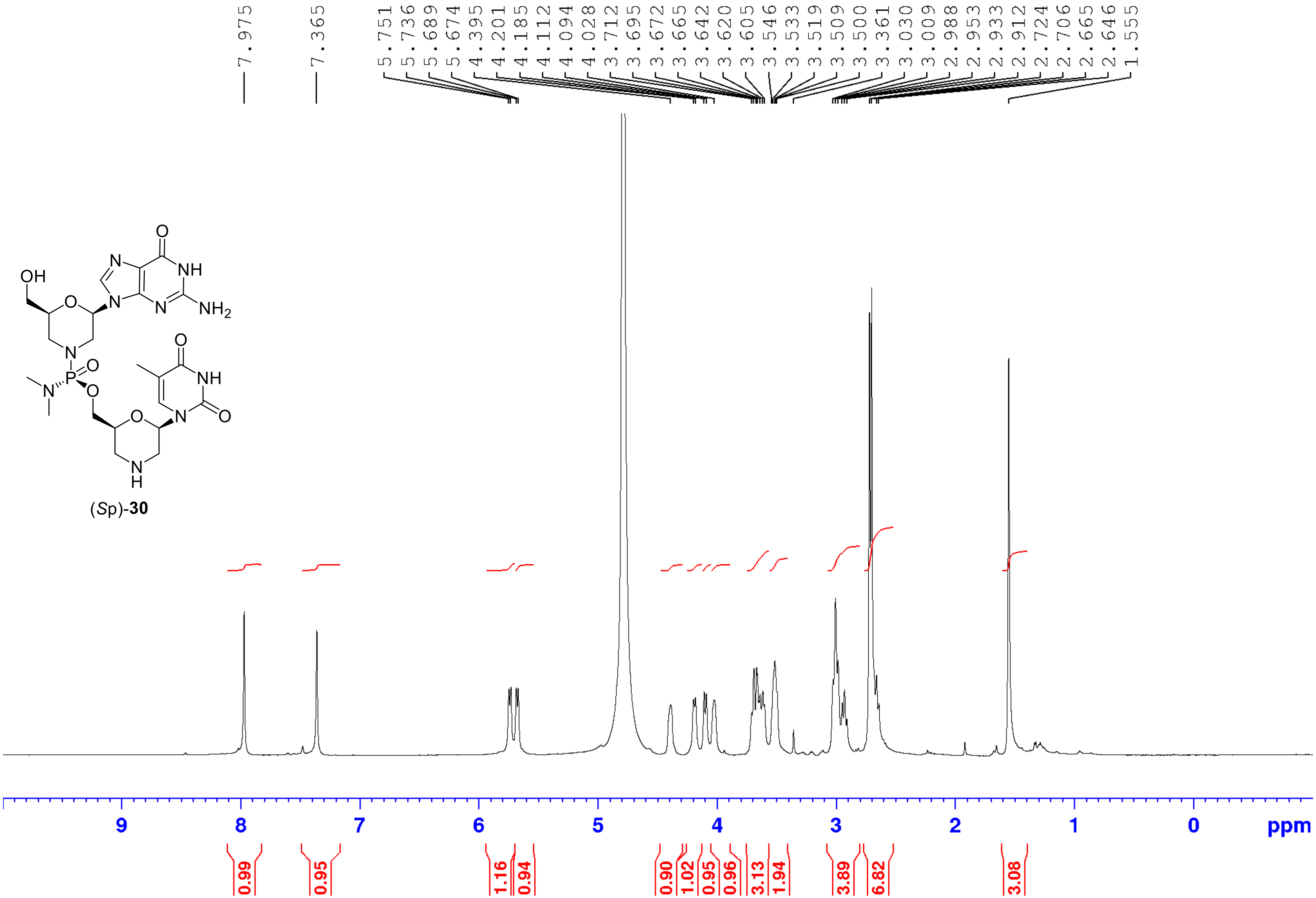

(*S*p)-**30**

$^{13}C\ \{^{1}H\}$ NMR (126 MHz, D_2O) of (*S*p)-**30**

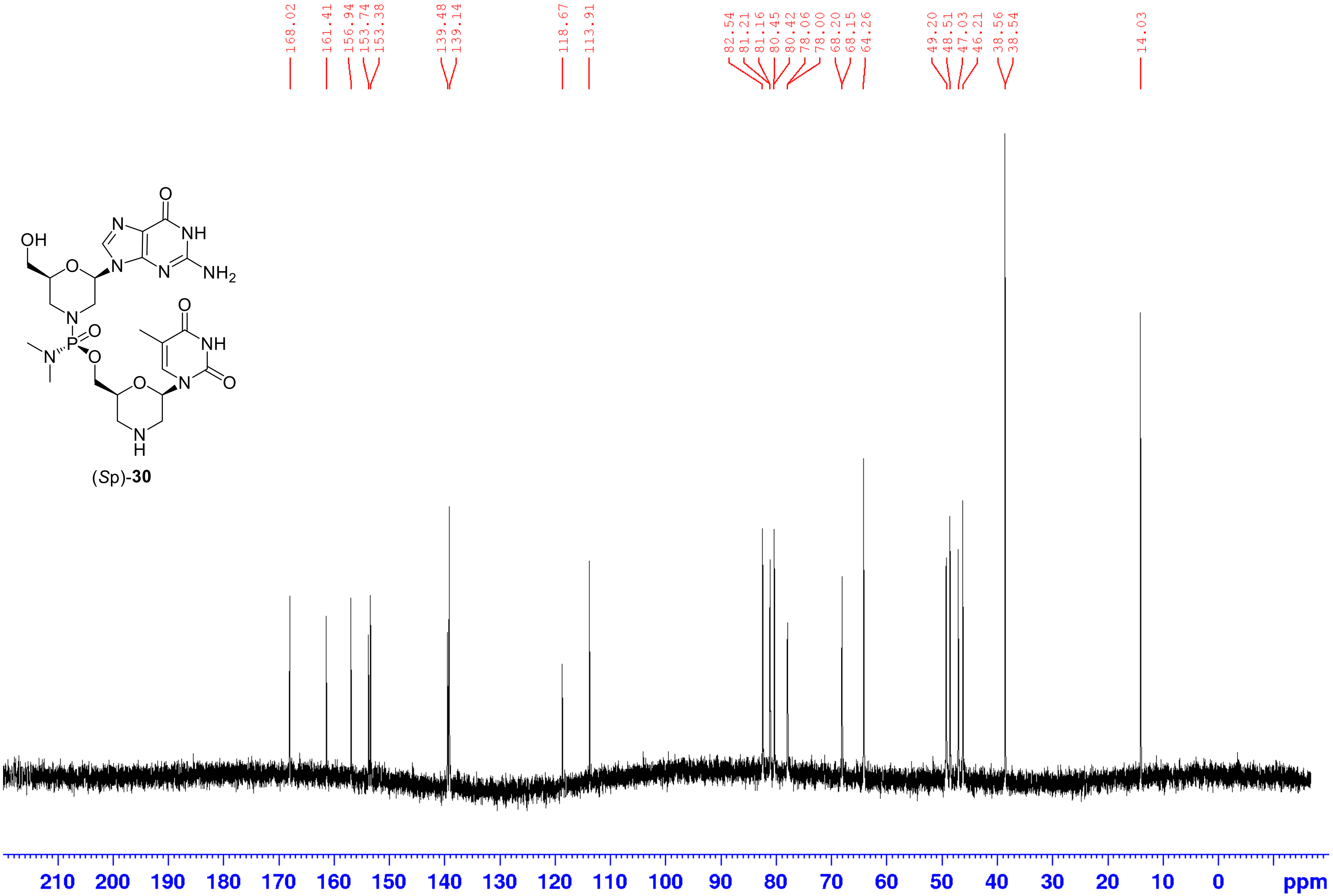

(*S*p)-**30**

$^{31}P\{^1H\}$ NMR (202 MHz, D_2O) of (*S*p)-**30**

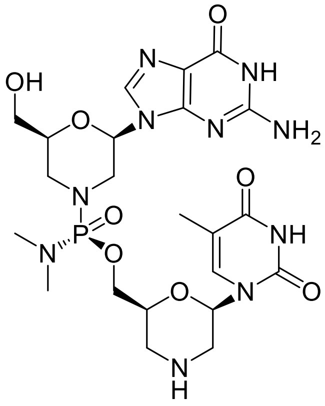

(*S*p)-**30**

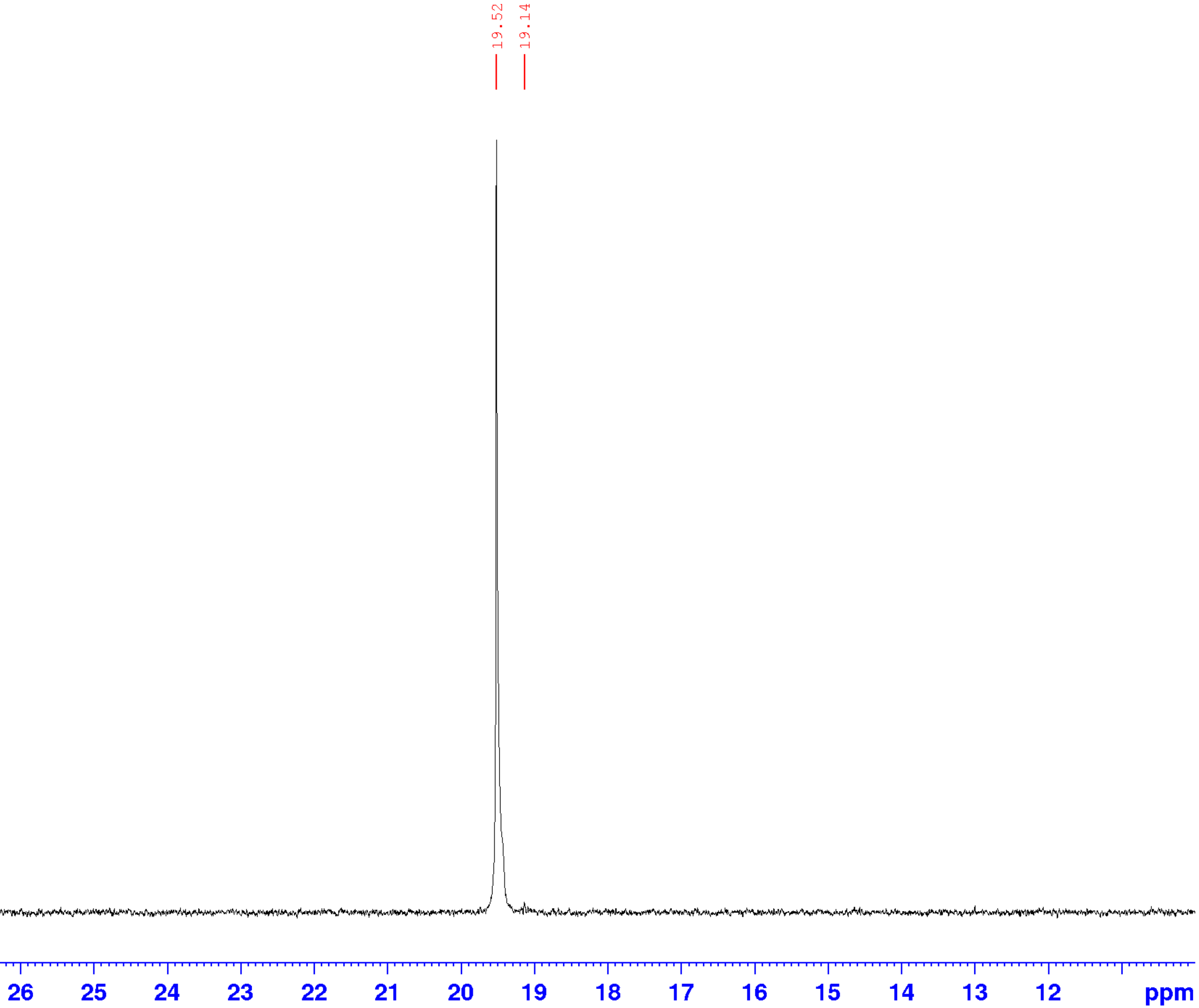

COSY (D_2O) of (*S*p)-**30**

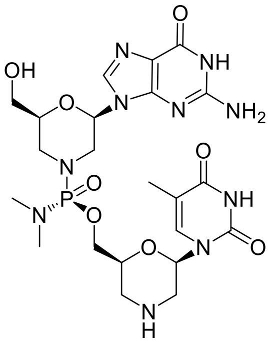

(*S*p)-**30**

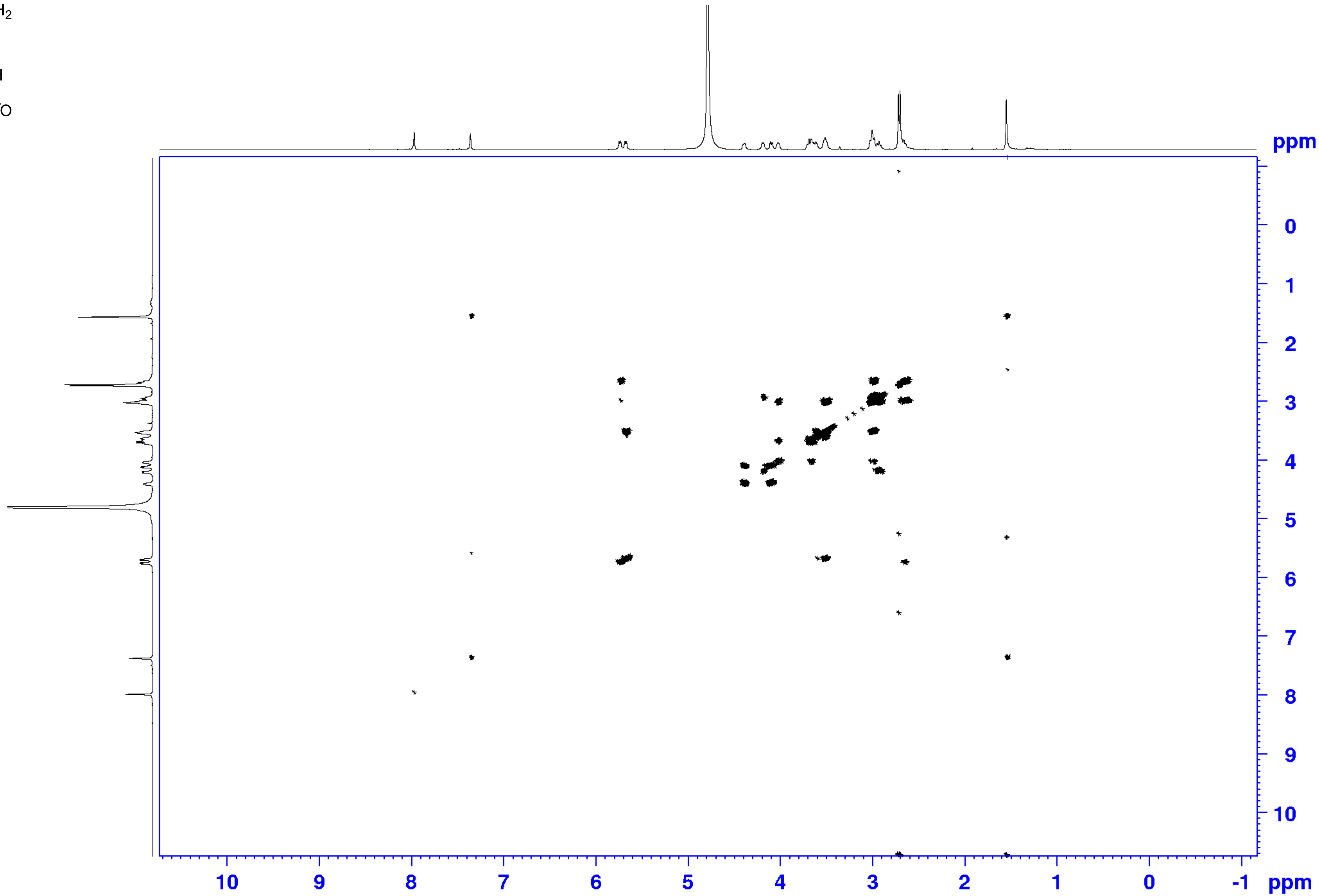

HSQC (D_2O) of (*S*p)-**30**

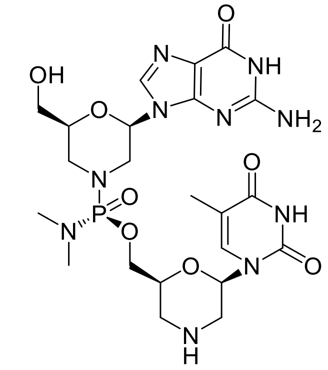

(*S*p)-**30**

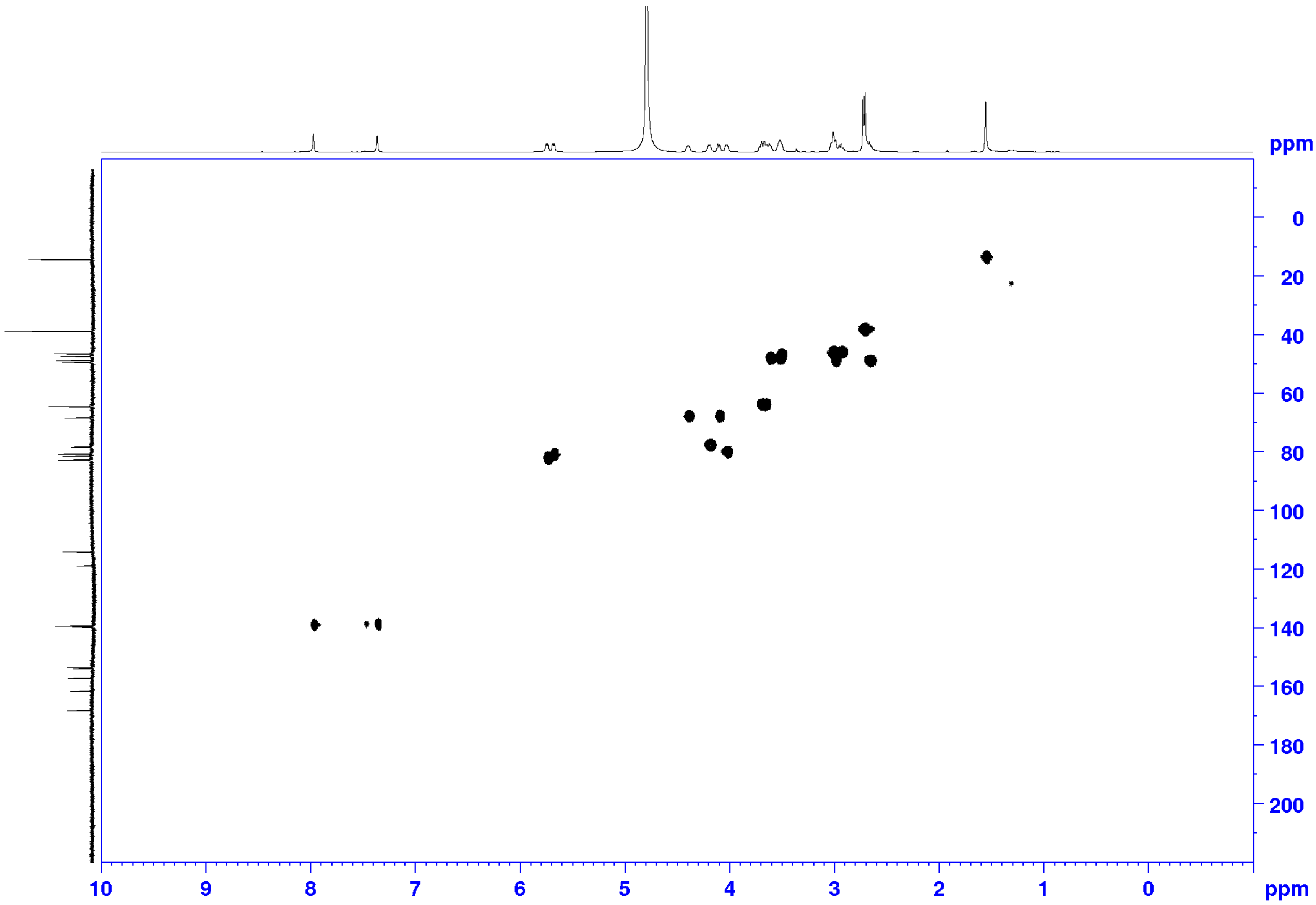

HMBC (D_2O) of (*S*p)-**30**

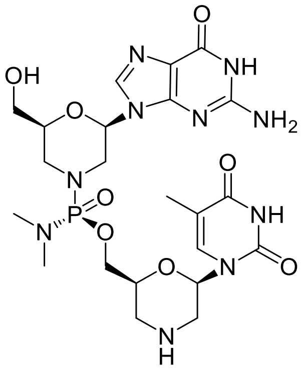

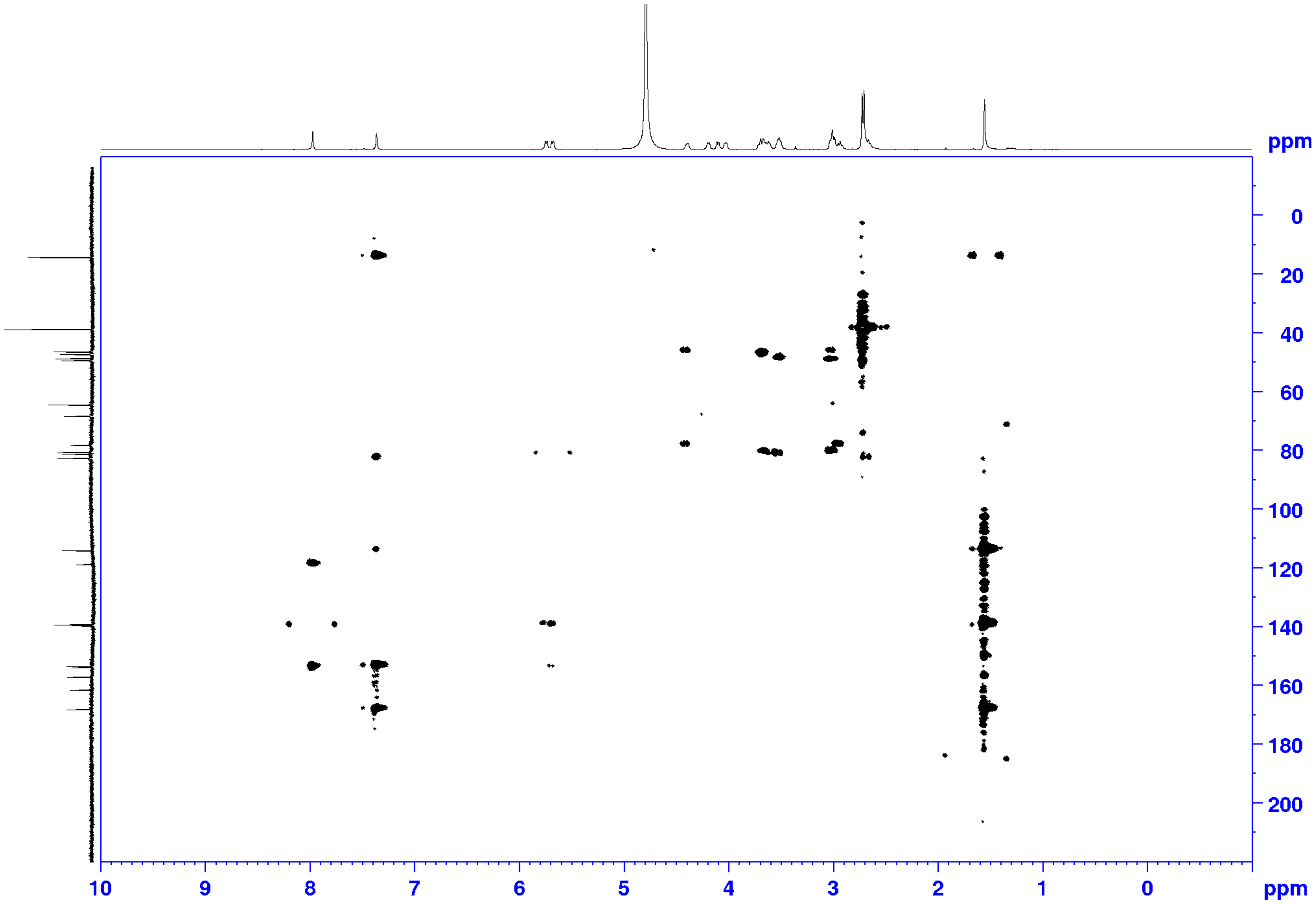

^{1}H NMR (600 MHz, D_2O) of (*R*p)-**30**

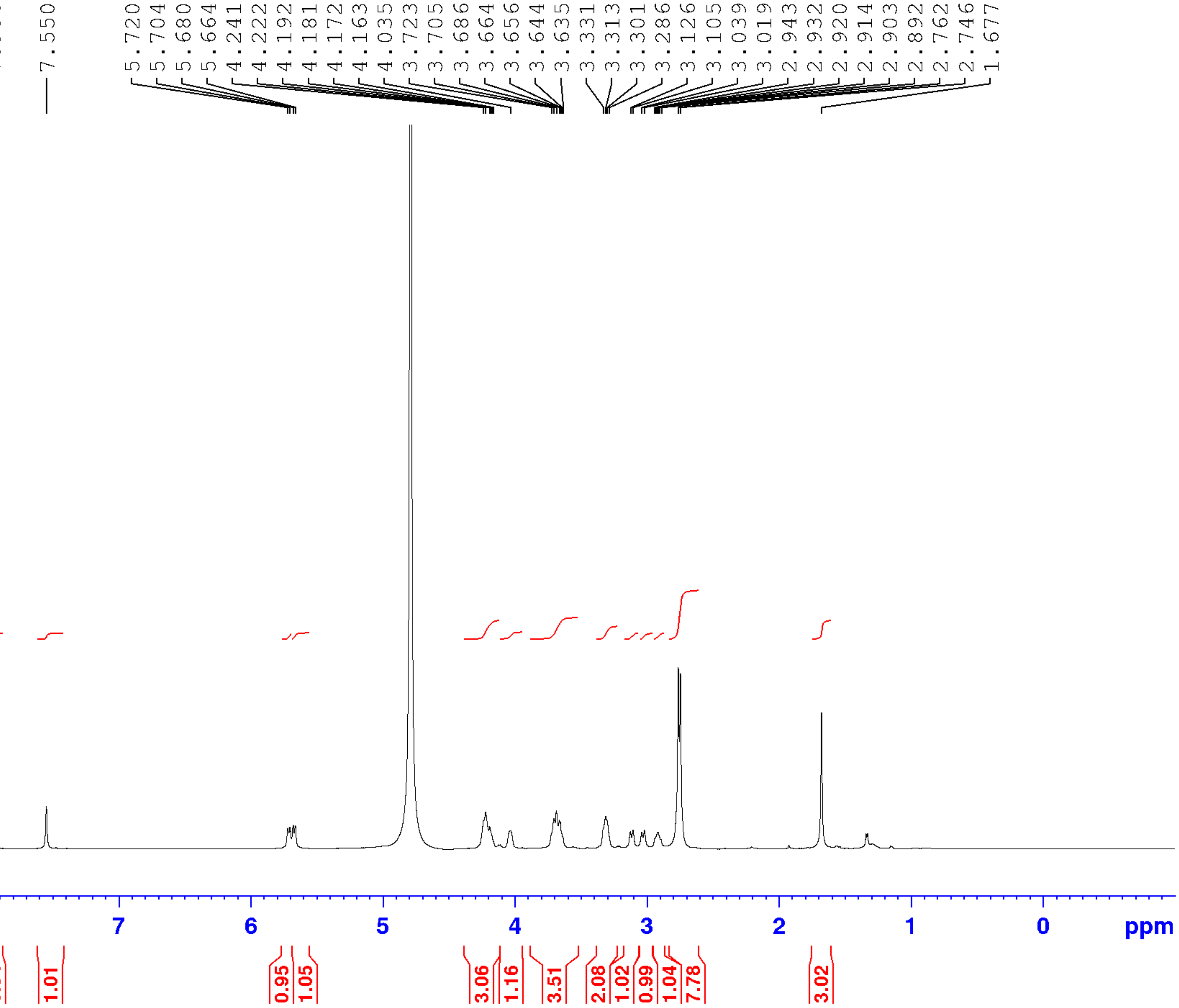

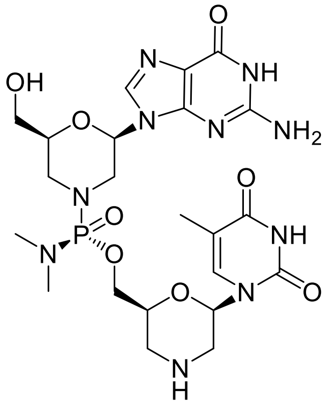

(*R*p)-**30**

^{13}C {^{1}H} NMR (126 MHz, D_2O) of (*R*p)-**30**

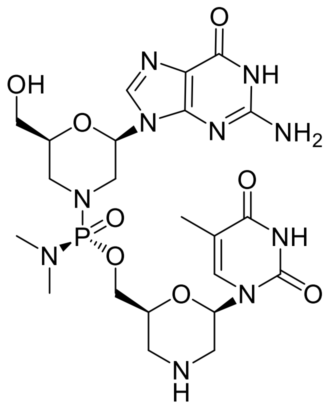

(*R*p)-**30**

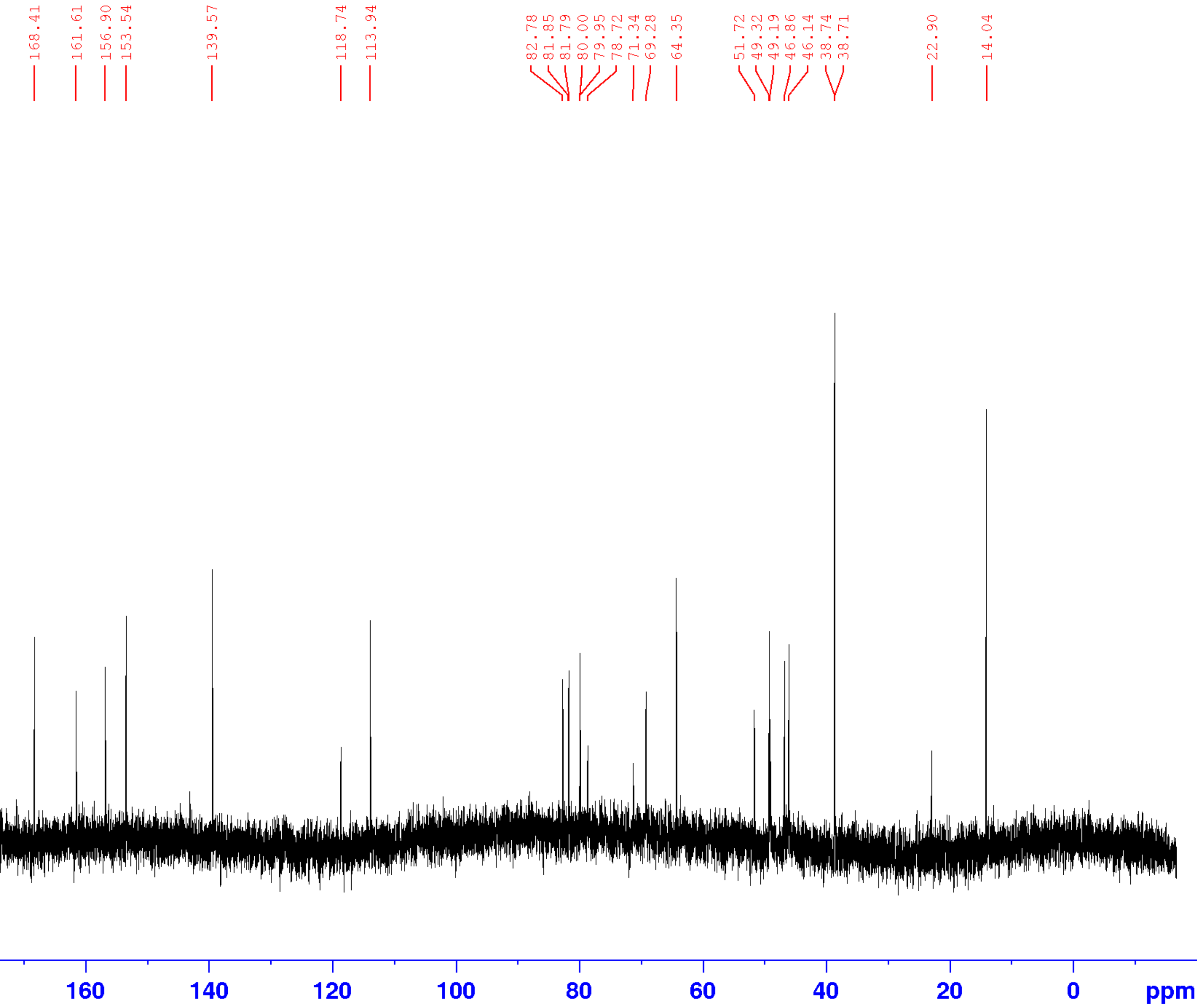

$^{31}P\ \{^{1}H\}$ NMR (202 MHz, D_2O) of (*R*p)-**30**

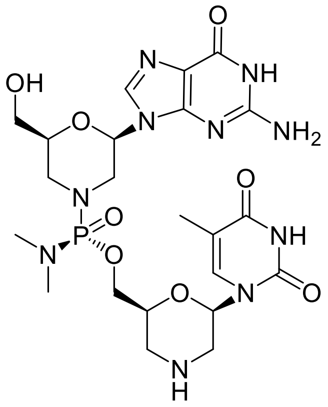

(*R*p)-**30**

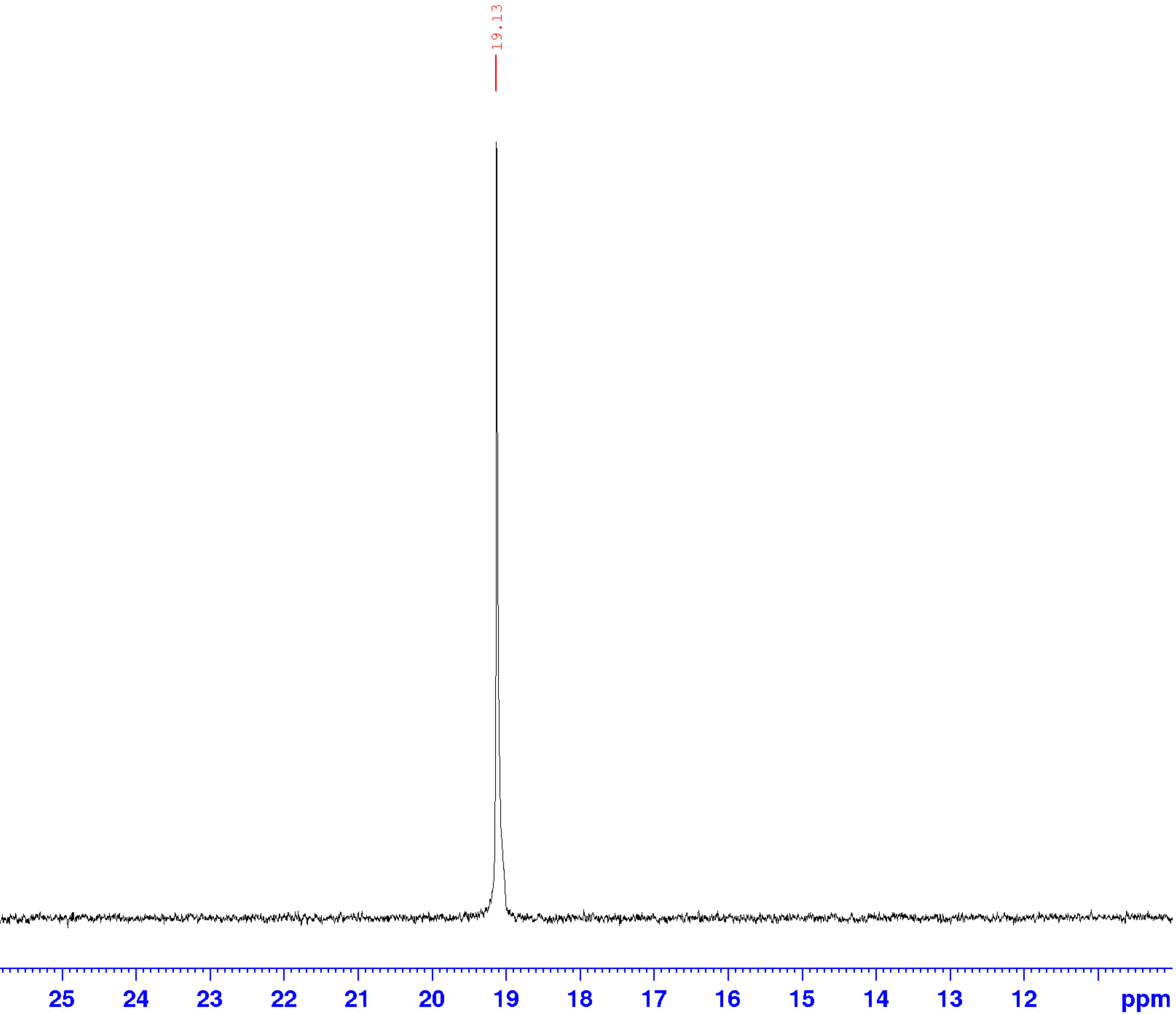

COSY (D_2O) of (*R*p)-**30**

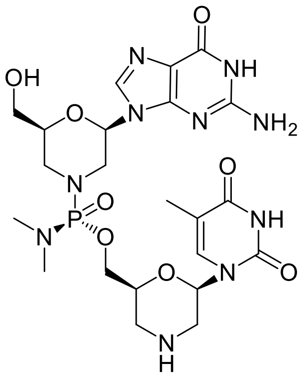

(*R*p)-**30**

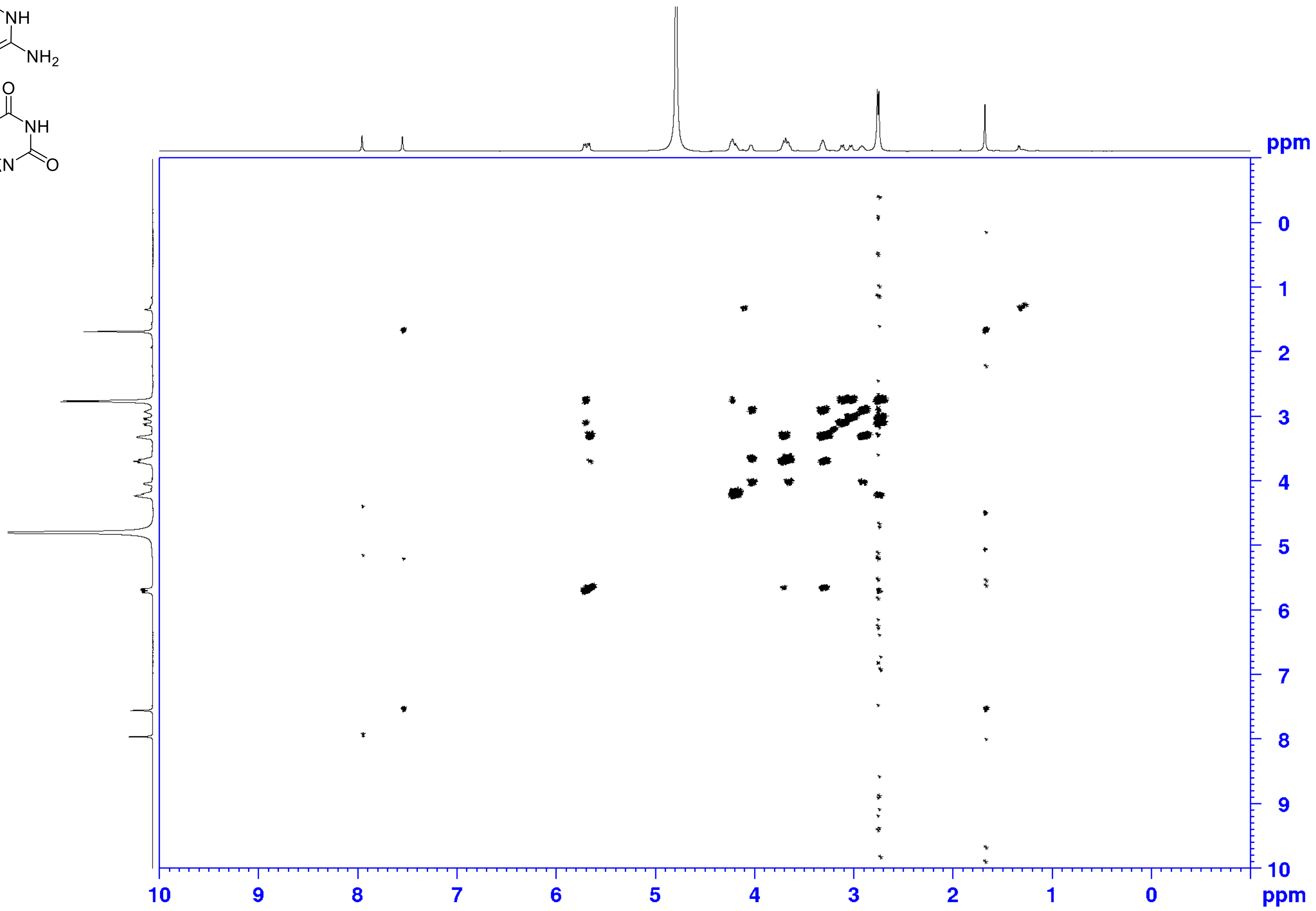

HSQC (D_2O) of (*R*p)-**30**

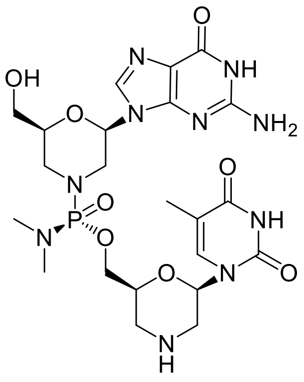

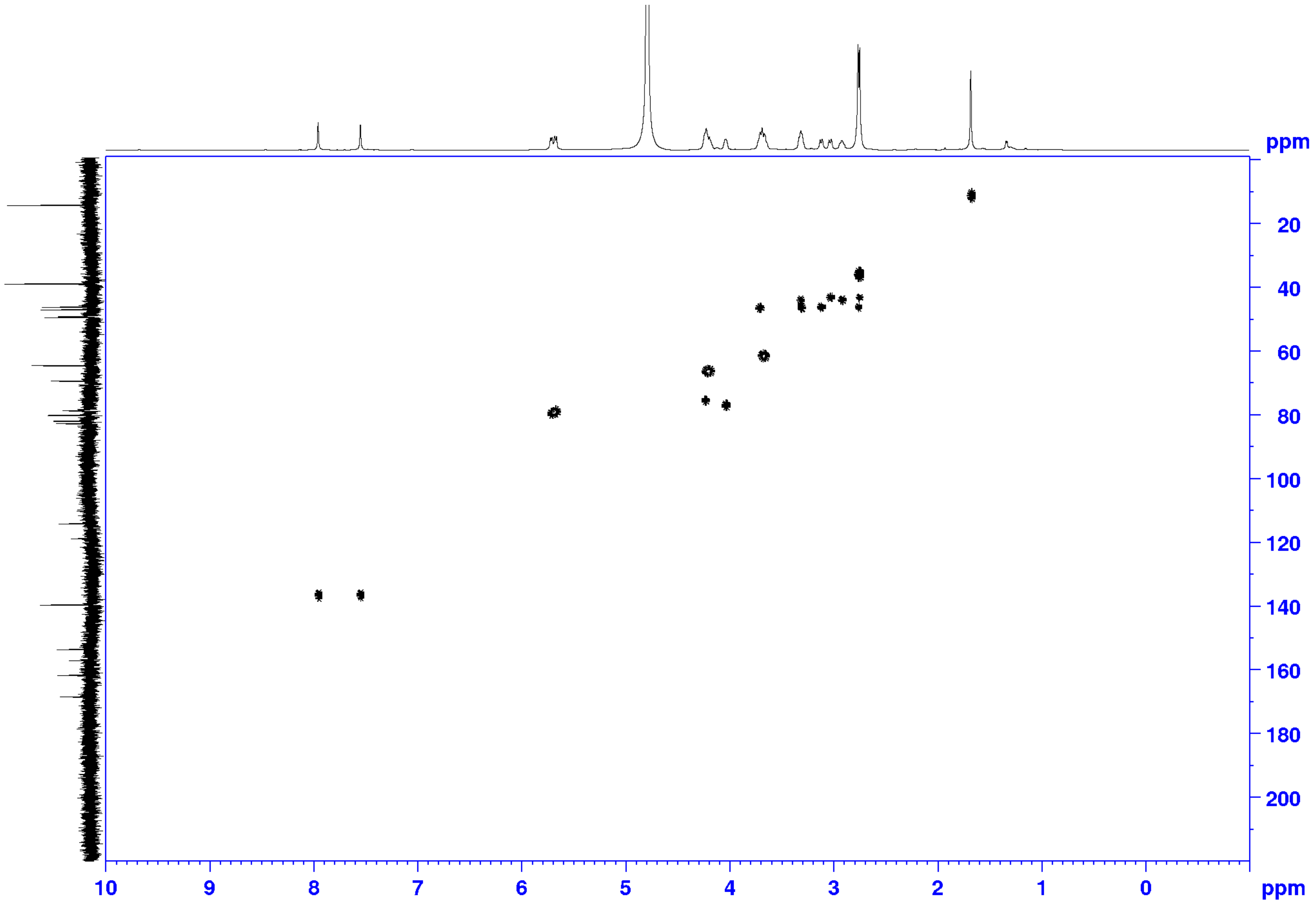

HMBC (D_2O) of (*R*p)-**30**

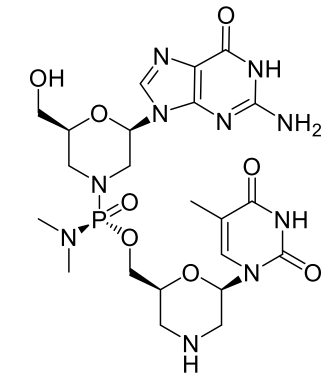

(*R*p)-**30**

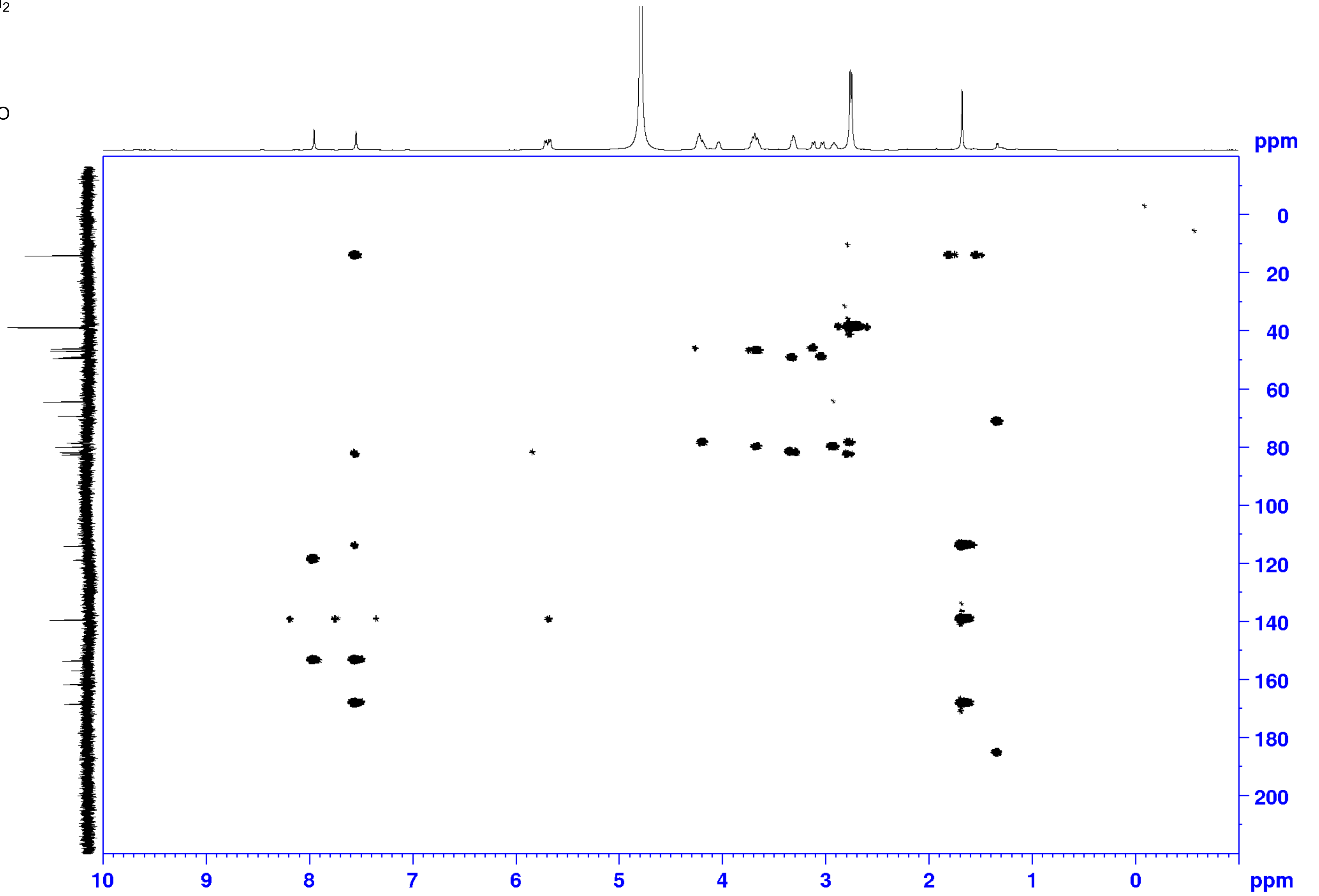

5. References

(1) Pattanayak, S.; Paul, S.; Nandi, B.; Sinha, S. Improved Protocol for the Synthesis of Flexibly Protected Morpholino Monomers from Unprotected Ribonucleosides. *Nucleosides Nucleotides Nucleic Acids* **2012**, *31* (11), 763–782. https://doi.org/10.1080/15257770.2012.724491.

(2) Tsurusaki, T.; Sato, K.; Imai, H.; Hirai, K.; Takahashi, D.; Wada, T. Convergent Synthesis of Phosphorodiamidate Morpholino Oligonucleotides (PMOs) by the *H*-Phosphonate Approach. *Sci Rep* **2023**, *13* (1), 12576. https://doi.org/10.1038/s41598-023-38698-2.
